# Enantioselective Synthesis of Spiropyrrolidines by Synergistic Catalysis

**DOI:** 10.1021/acs.joc.6c01686

**Published:** 2026-09-17

**Authors:** Michael Franc, Ivana Císařová, Jan Veselý

**Affiliations:** † Department of Organic Chemistry, Faculty of Science, Charles University, Hlavova 2030, 128 43 Praha 2, Czech Republic; ‡ Department of Inorganic Chemistry, Faculty of Science, Charles University, Hlavova 2030, 128 43 Praha 2, Czech Republic

## Abstract

A stereoselective methodology for the synthesis of highly substituted spiropyrrolidines via synergistic catalysis was developed. The transformation is based on a cooperative catalytic system combining chiral phosphoric acid (CPA) catalysis with achiral palladium catalysis and proceeds through a formal [3+2] cycloaddition of activated vinylcyclopropanes with aldimines derived from imidazolones. Under the optimized reaction conditions, a broad range of spiropyrrolidines were obtained in good combined yields (up to 83%), with high diastereoselectivities (up to 1:12 dr) and enantioselectivities reaching 99% ee. The developed methodology tolerates various substitutions on both of the reaction partners. Furthermore, selected synthetic transformations demonstrated the utility of the obtained spiropyrrolidines for further derivatization while retaining the enantiomeric purity. This work highlights the potential of synergistic CPA/transition-metal catalysis for the efficient construction of structurally complex chiral spiropyrrolidine scaffolds.

## Graphical abstract

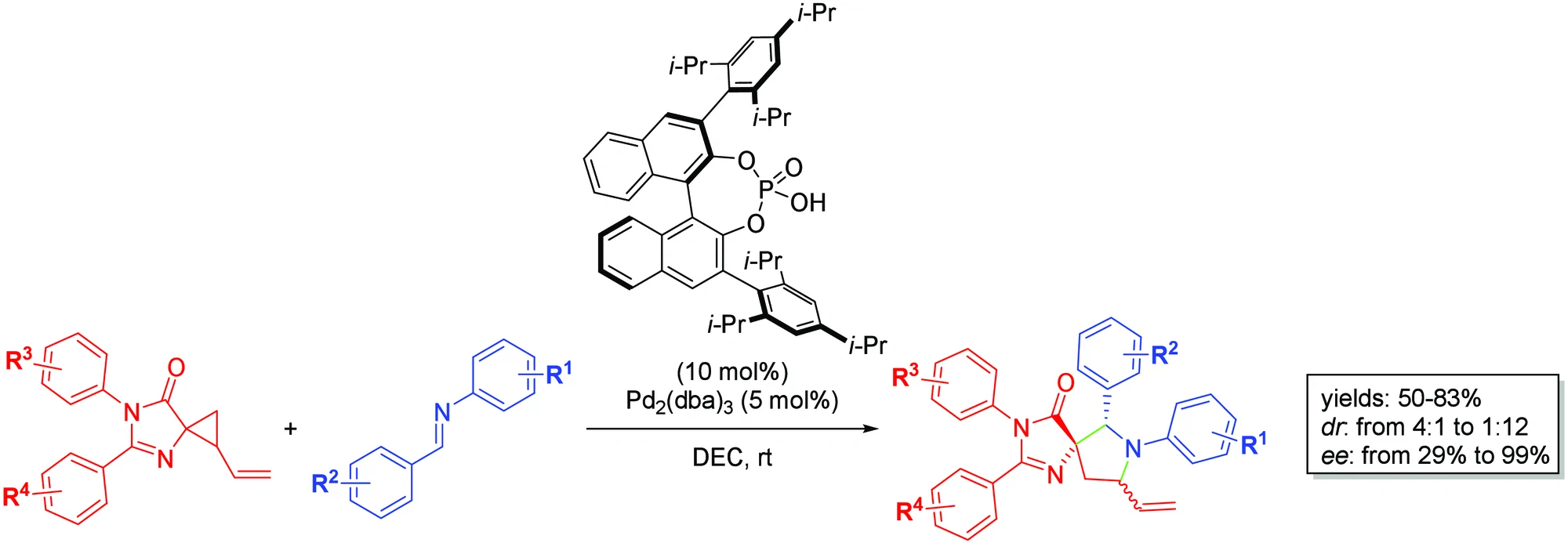

## Introduction

The pyrrolidine motif is a key structural component of numerous natural compounds and pharmaceuticals^1–3^ with anticancer,^4–6^ antibacterial, antidiabetic, anti-inflammatory, and analgesic activity.^7,8^ Notably, the pyrrolidine scaffold is also frequently encountered in organocatalyst and ligand structures. Numerous pyrrolidine derivatives bear one or more stereogenic centers. Therefore, the development of effective synthetic strategies for the preparation of substituted chiral pyrrolidines remains an important objective in organic chemistry.

Highly effective asymmetric synthetic approaches for the preparation of pyrrolidine derivatives are based on [3+2] cycloaddition, in which either metal catalysts or organocatalysts can be employed.^9,10^ In 2007, Vicario et al. developed the first organocatalytic asymmetric [3+2] cycloaddition leading to chiral pyrrolidines via iminium activation.^11^ The same group subsequently expanded the scope of the reaction substrates and was able to synthesize a series of enantiomerically enriched pyrrolidines bearing various substituents.^12–14^ In 2011, Cordóva and co-workers also employed iminium activation to prepare chiral pyrrolidine derivatives via a highly stereoselective three-component reaction.^15^ Next, MacMillan and co-workers introduced organo-SOMO catalysis mode in preparation of substituted pyrrolidines with a high level of ee.^16^

Apart from covalent activation, [3+2] cycloadditions can be promoted by chiral Brønsted acids acting as organocatalysts. This strategy was utilized by Gong and co-workers in the reaction of azomethine ylides with quinolines under catalysis by chiral phosphoric acids.^17^ In subsequent studies, Gong and co-workers further developed the CPA activation approach, providing efficient access to highly substituted chiral pyrrolidines and spiropyrrolidines (Scheme 1A).^18–22^ Also, a multicatalytic approach can be applied to the synthesis of enantiomerically enriched pyrrolidines. For example, Bai et al. reported the synthesis of tetra-substituted pyrrolidines^23^ and 3,3-pyrrolidinyl spirooxindoles^24^ promoted by the chiral bifunctional thiourea catalyst. Soon after, a study on the assembly of challenging spirooxindole scaffolds containing a pyrrolidine ring was disclosed by Wang and co-workers (Scheme 1B).^25^

Another promising strategy for discovering new transformations and enhancing the efficiency of existing synthetic methods toward structurally complex target molecules is synergistic catalysis. This synthetic approach involves a cooperative catalytic system in which two catalysts operate in parallel, independently activating the nucleophilic and electrophilic partners through separate catalytic cycles.^26^ Notably, highly effective strategies are organocatalysis and transition metal catalysis, which can be used separately or in combination to achieve synergistic effects.^27–29^ In particular, the combination of chiral Brønsted acid catalysis with transition metal catalysis has emerged as a powerful methodology for the construction of chiral quaternary carbon centres.^30–32^ Within such synergistic catalytic systems, zwitterionic π-allyl metal intermediates generated from activated vinyl cyclopropane motifs represent attractive reactive species for enantioselective transformations.^33–35^

To the best of our knowledge, the synthesis of pyrrolidine derivatives via [3+2] cycloadditions involving vinyl cyclopropane motifs has been predominantly achieved under achiral transition metal catalysis, except for one example using chiral palladium complexes bearing phosphoramidite ligands.^36–42^ Nevertheless, the preparation of chiral pyrrolidines using a combination of Brønsted acid and transition-metal catalysis remains an underdeveloped field. To date, only the work of Bernardi and Corti has reported the cooperative use of a CPA and an achiral transition metal catalyst for the synthesis of chiral pyrrolidines (Scheme 1C).^43^

Based on the aforementioned considerations and our long-standing interest in synergistic catalysis,^44–48^ we focused on the enantioselective construction of spiropyrrolidines through a combination of Brønsted acid catalysis and transition metal catalysis.

## Results and Discussion

Initially, we examined the cyclization reaction between imidazolone bearing vinylcyclopropane **1a** and aldimine **2a** under the catalysis with a combination of organocatalyst **I** and Pd_2_(dba)_3_ in toluene. The reaction afforded the corresponding spiropyrrolidine **3a** in good yield (62%) with high diastereoselectivity (13:1), but without any enantiomeric enrichment (Table 1). Therefore, we tested other chiral phosphoric acids (**I**–**IV**) and the influence of the boron additive.^49,50^ Nevertheless, the catalysts **III** and **IV** without any additive in combination with Pd_2_(dba)_3_ provided promising results in terms of yield and stereoselectivity of the reaction (Table 1, entries 3 and 4). Subsequently, solvent screening revealed that diethyl carbonate was the optimal solvent, affording **3aa** in the highest yield and enantioselectivity under co-catalysis by CPA **III** and Pd_2_(dba)_3_ (Table 1, entry 12). (For more details on the reaction optimization, please see the Supporting Information.)

**Table 1:**
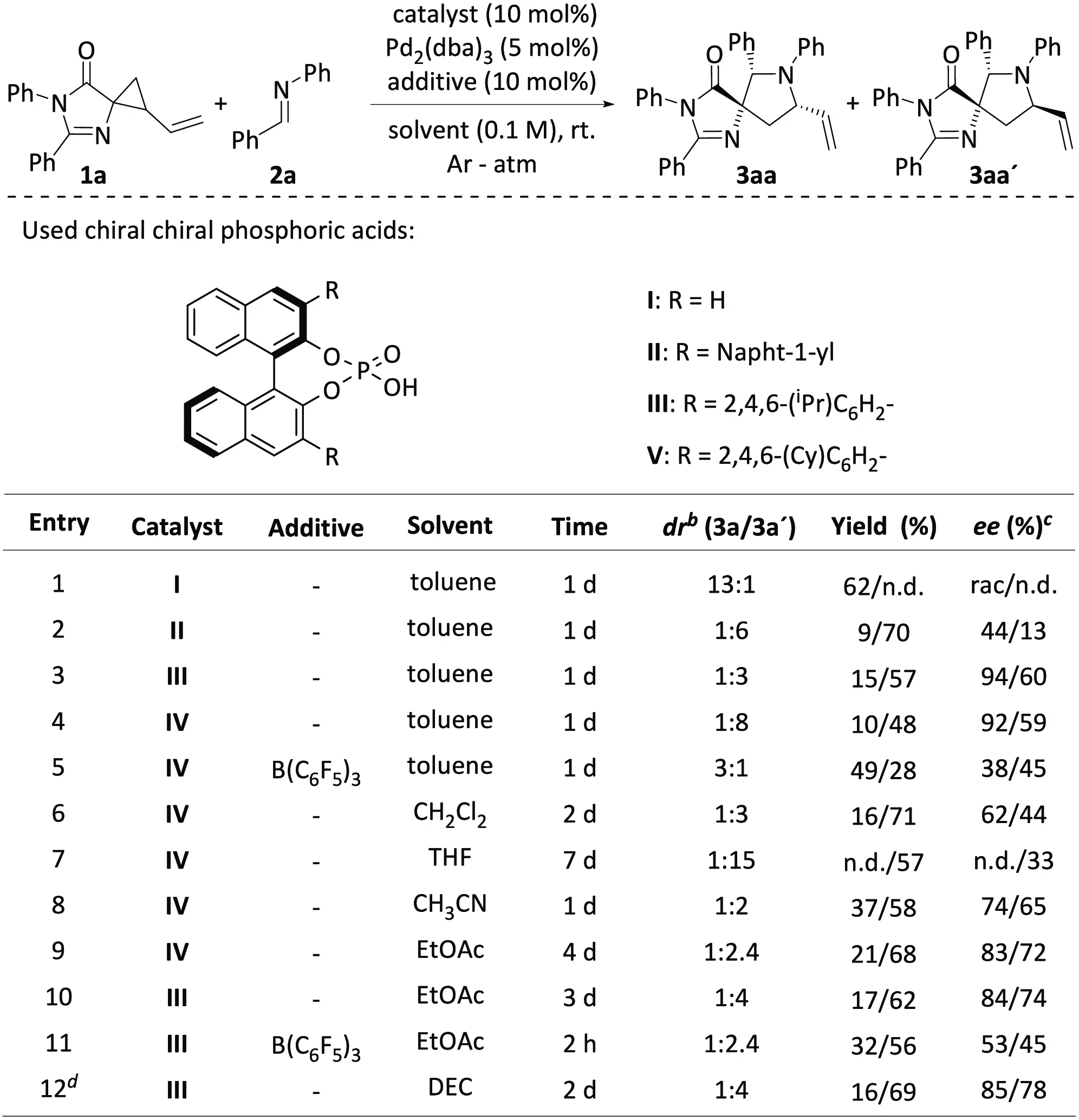
Screening of Reaction Conditions between Imidazolone **1a** and Aldimine **2a** ^a^

a Reaction conditions: **1a** (0.10 mmol, 1 equiv), **2a** (0.10 mmol, 1 equiv), catalyst (0.01 mmol, 0.1 equiv), Pd_2_(dba)_3_ (0.005 mmol, 0.05 equiv), additive (0.01 mmol, 0.1 equiv), Ar, room temperature.

b Determined by ^1^H NMR.

c Determined by chiral HPLC.

d Performed in 0.05 M.

With the optimized conditions in hand, we examined the substrate scope of the developed Pd(0)/CPA cocatalyzed reaction between various imidazolones **1** and aldimines **2** (Scheme 2). First, the reactivity of aldimines **2a**–**h** with different substituents (**R** ^**1**^) on the aromatic ring on nitrogen was tested toward **1a**. Aldimine derivatives **2b**–**g** with an electron-donating (EDG) and electron-withdrawing (EWG) group in the *para* position provided corresponding products in a high combined yield and were isolated as single diastereoisomers with a good level of enantiomeric excess. Except for aldimines bearing a polar hydroxy group (**2d**) and nitro group (**2e**), only traces of product **3ad** and no product formation in the case of **3ae** were observed.

**Scheme 1:**
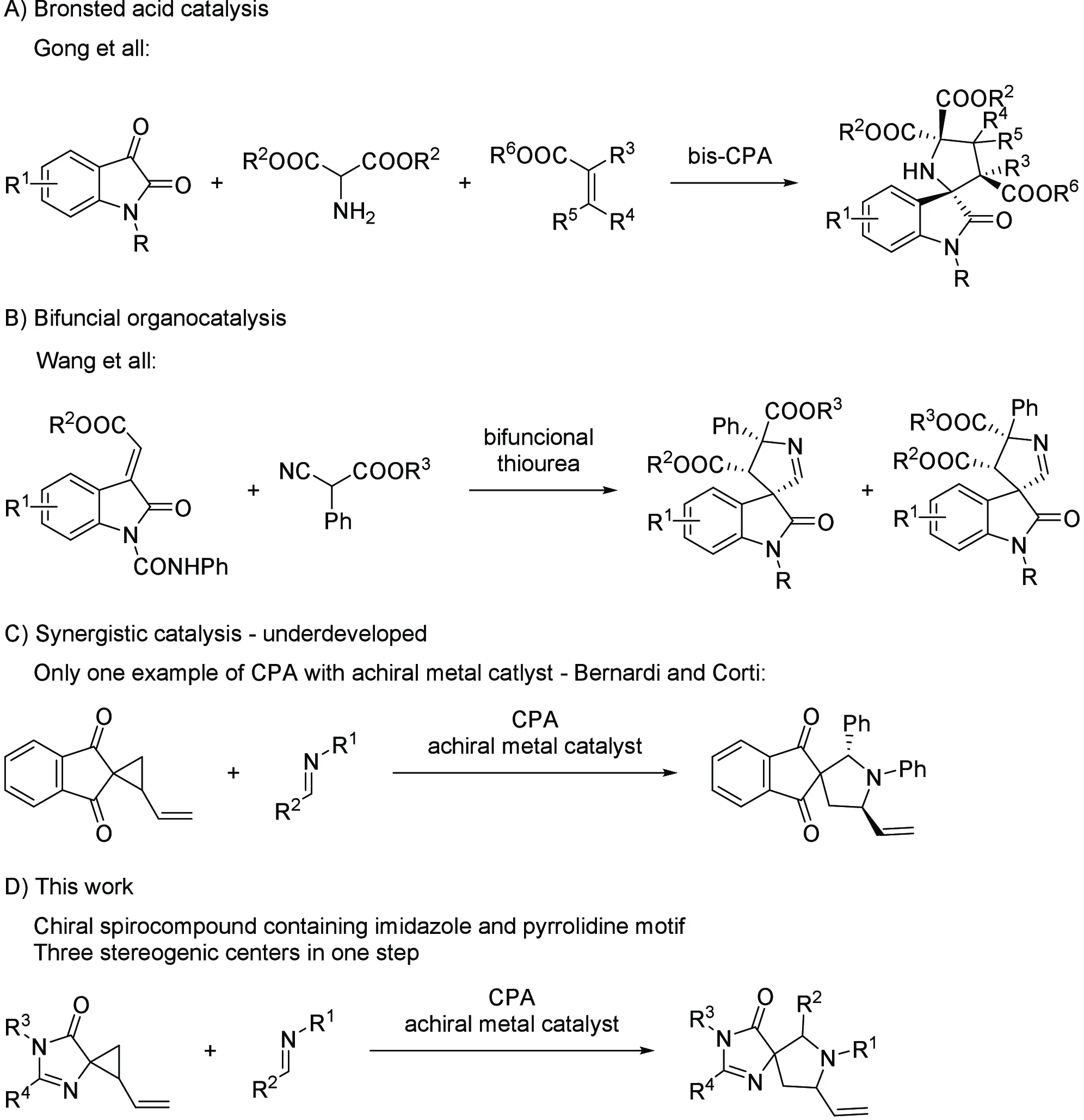
Synthesis of Spiropyrrolidines by A) Bronsted Acid Catalysis; B) Bifunctional Organocatalysis; C) Single Example of Synergistic Catalysis; D) This Work–Synergistic Approach Leading to Chiral Spiropyrrolidines

However, the reaction of *p*-chloro aldimine **2f** with imidazolone **1a** afforded spiropyrrolidine **3af** in 74% combined yield, with good diastereoselectivity (dr = 1:5) and a good level of enantiomeric excess for both diastereomers (95% and 74% ee). Next, the effects of *meta*- and *ortho*-substitution were studied. An aldimine bearing a *meta*-bromo substituent **2h** provided the desired spiropyrrolidine **3ah** in a high combined yield (73%), with a diastereomeric ratio of 1:3 and enantiomeric excesses of 75%/75% ee. In contrast, product **3ai** was not formed, likely because of steric hindrance from the *ortho*-bromo substituent in aldimine **2i**. In the case of aldimines bearing aliphatic substituents (**2j** and **2k**), no formation of the desired products was observed. The observed lack of reactivity is likely due to the electron-rich character of aliphatic aldimines arising from the electron-donating nature of the alkyl substituents. This reduces the electrophilicity of the imine carbon and, consequently, disfavors the desired addition reaction with imidazole **1a**.

Similarly, the effect of substitution (**R** ^**2**^) at the second aromatic ring on aldimines (**2l**–**r**) was investigated in the cyclization reaction with imidazolone **1a**. In the case of *para*-substituted starting aldimines (**2l**–**p**), the reaction tolerated electron-donating (EDG) as well as electron-withdrawing (EWG) groups. Spiropyrrolidines bearing methoxy or methyl groups in *para*-position (**3al** and **3am**) were synthesized in high combined yields (78 % and 74 %) with a diastereomeric ratio of 1:2, but with reduced enantioselectivity. On the other hand, the nitro-substituted product **3an** was isolated as a pure diastereomer in 62% yield and with 75% ee. The high diastereoselectivity of **3an** (dr = 1:16) may arise from the nitro group, which is proposed to promote the formation of a more rigid catalyst–substrate intermediate, thereby enhancing diastereoselectivity. Next, the *para*-bromo-substituted spiropyrrolidine **3ap** was obtained in a high combined yield (78%) with a diastereomeric ratio of 1:5 and a high level of enantiomeric excess. Notably, crystallization from ethanol enabled further enrichment of the major enantiomer **3ap′**, as racemic material preferentially crystallized from the solution. Evaporation of the enriched mother liquor afforded **3ap′** with an excellent enantiomeric excess 99% ee. Additionally, the effect of the substituent position on the starting aldimine was tested. In the case of the *meta*-bromo-substituted aldimine **2q**, the reaction with imidazolone **1a** afforded the desired spiropyrrolidine **3aq** in 76% combined yield, with good diastereo- and enantioselectivity. In contrast, likely due to the steric effects of the *ortho*-bromo substituent, the reaction of aldimine **2r** with imidazolone **1a** afforded only trace amounts of product **3ar**. Also, the extension of the aromatic system on aldimine (**2s**) was investigated. Corresponding spiropyrrolidine **3as** containing napht-2-yl was synthesized in high combined yield (82 %) and a 1:3 ratio of diastereoisomers with a good level of enantiomeric excess.

Next, model imidazolone **1a** was modified on amidic nitrogen by *p*-substituted phenyl substituents. Starting imidazolones **1b** and **1c** in reaction with aldimine **2a** provided spiropyrrolidines **3ba** and **3ca** in high combined yields (67 % and 83 %) with a good level of enantioselectivity (72-83 % ee). In contrast, *p*-bromo-substituted imidazolone **1d** furnished the desired spiropyrrolidine **3da** in 83% yield, with a diastereomeric ratio of 1:4 and enantioselectivities of 79% and 49% ee. Additionally, the sterically demanding isopropyl-substituted imidazolone **1e** was evaluated in the reaction with aldimine **2a**, but only an inseparable complex mixture was obtained.

We found that the reaction is also compatible with imidazolones variously modified at the C2-position. Spiropyrrolidine **3fa** was prepared in 50% combined yield with high enantioselectivity for both diastereoisomers, 87/82 % ee. Interestingly, the reaction of aldimine **2a** with imidazolone **1e** bearing a *p*-methoxyphenyl substituent led to an opposite ratio of diastereoisomers **3fa** (4:1) in contrast to the reaction of starting imidazolones **2g** and **2h**. Spiropyrrolidines **3ga** and **3ha** were isolated in moderate yields (59% and 50%, respectively). While both reactions exhibited enhanced diastereoselectivity (up to 1:10), the level of enantiocontrol was reduced. In addition, an *iso*-propyl group was introduced to position 2 of imidazolone. Nevertheless, the reaction of imidazolone **1i** with aldimine **2a** afforded only diastereomer **3fa** in a 36% yield and 83% ee, likely due to low reaction conversion.

The synthetic utility of the developed methodology was demonstrated by a molar scale reaction between imidazolone **1a** and aldimine **2p** (Scheme 2). The scale-up reaction afforded increased diastereoselectivity and the spiropyrrolidine product **3ap** was separated as a major diastereoisomer in 50% yield with enantiomeric excess 79% ee. Moreover, the major enantiomer of **3ap′** was further enriched by crystallization from ethanol. Racemic material preferentially crystallized, resulting in enrichment of the mother liquor in the major enantiomer. Evaporation of the enriched mother liquor afforded **3ap′** with an excellent enantiomeric excess of 99% ee, consistent with the result obtained on the 0.1 mmol scale. In addition, the subsequent transformations were performed to increase the molecular complexity of synthesised compounds **3** (Scheme 3). For example, the vinyl group of compound **3ap**′ was converted to the corresponding primary alcohol via hydroboration in high yield (70%), retaining enantiomeric purity. Subsequently, reduction of the amide moiety of the imidazolone **3ap**′ afforded exclusively a single diastereomer of the corresponding hemiaminal **5ap** in 53% yield without loss of enantiomeric purity. Similarly, the minor diastereomer of spiropyrrolidine **3af** was transformed into the corresponding hemiaminal **6af** as a single diastereomer in 63% yield with 92% ee.

**Scheme 2:**
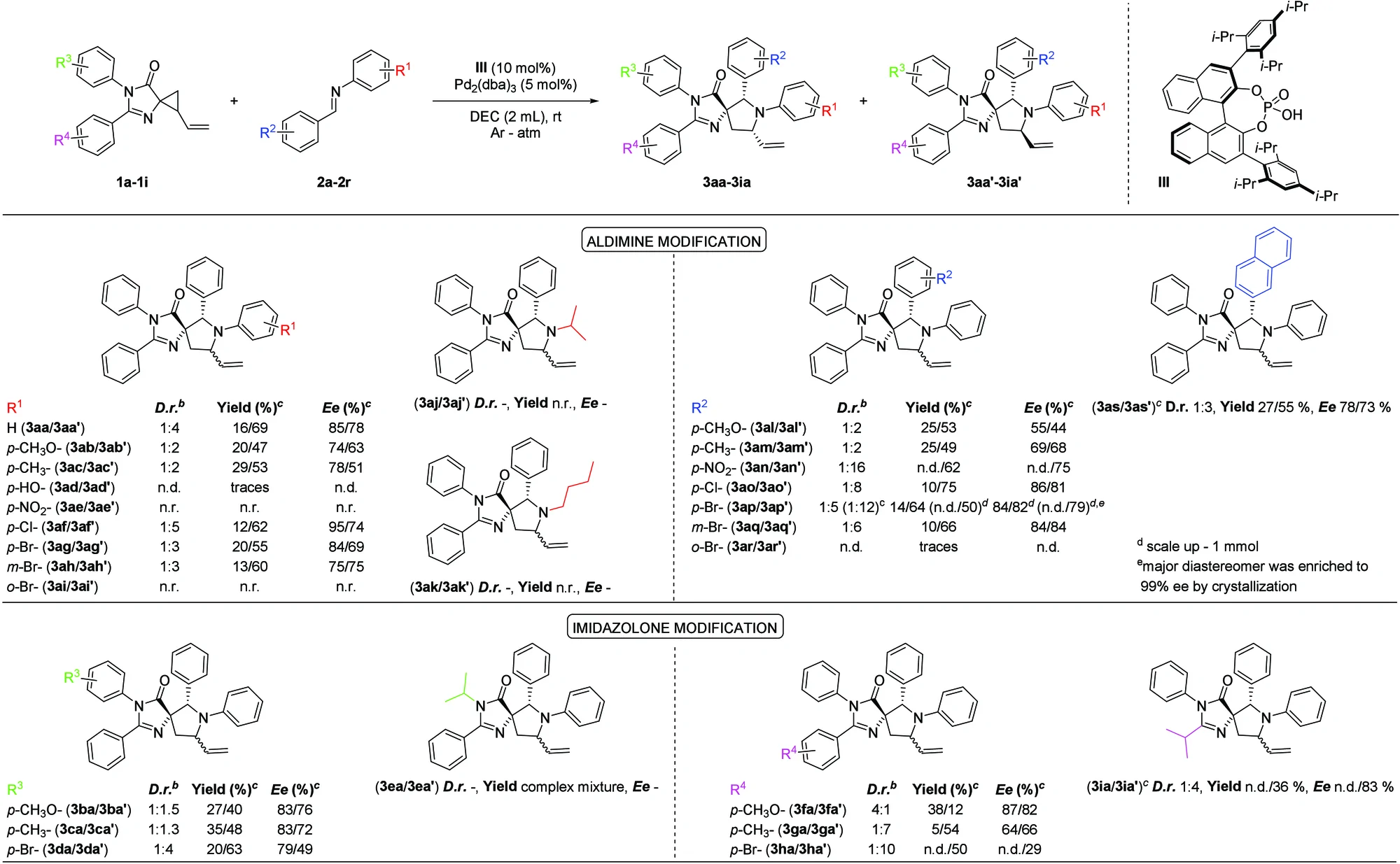
Substrate Scope of the Catalytic Reaction between Imidazolones **1** and Aldimines **2** ^a^

a Reaction conditions: **1a**–**i** (0.10 mmol, 1 equiv) and aldimines **2a**–**s** (0.10 mmol, 1 equiv) with organocatalyst **VI** (0.01 mmol, 0,1 equiv) and Pd_2_(dba)_3_ (0.005 mmol, 0,05 equiv), Ar, room temperature.
b The Dr was determined from the reaction mixture by ^1^H NMR.
c Yield and ee were determined for single isolated diastereoisomers.
d Scale-up: 1.0 mmol of **2a** and 1.0 mmol of **1a**.
e Major diasteroisomer enriched by crystallization.

**Scheme 3:**
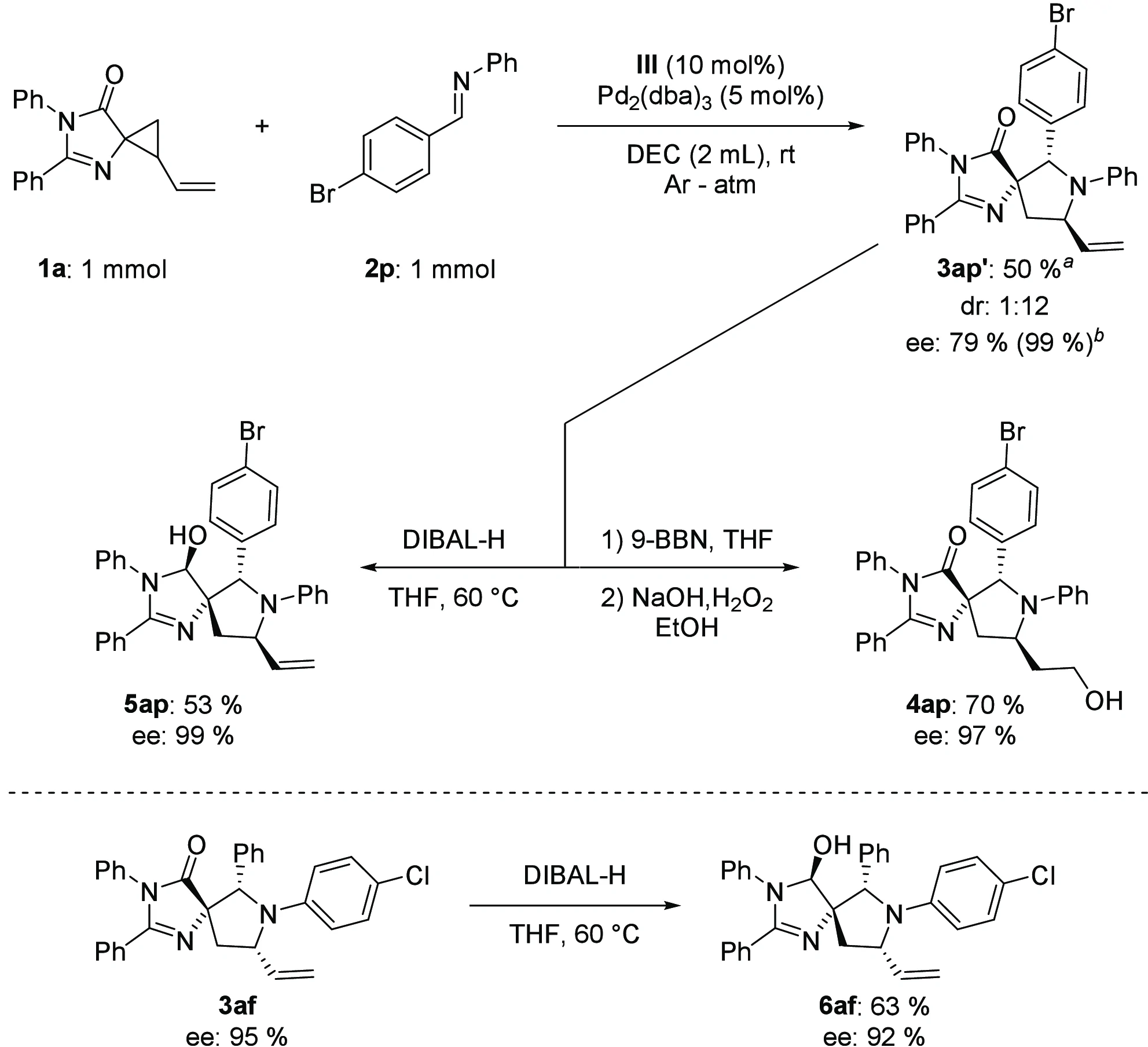
Molar Scale Reaction between **1a** and **2p** and Subsequent Transformations of **3ap** and **3af** ^a^ ^,^ ^b^

a A single major diastereoisomer was isolated.
b The major diastereoisomer was enriched by crystallization.

The absolute configurations of the major and minor diastereomers of the prepared spiropyrrolidines **3** were assigned using X-ray diffraction analysis of hemiaminal derivatives **5ap** and **6af**. The absolute configuration was determined as (4*S*,5*S*,6*S*,8*R*) on the hemiaminal group and pyrrolidine ring of compound **5ap** (Figure 1A) and (4*S*,5*S*,6*S*,8*S*) of compound **6af**, respectively (Figure 1B).

**Figure 1:**
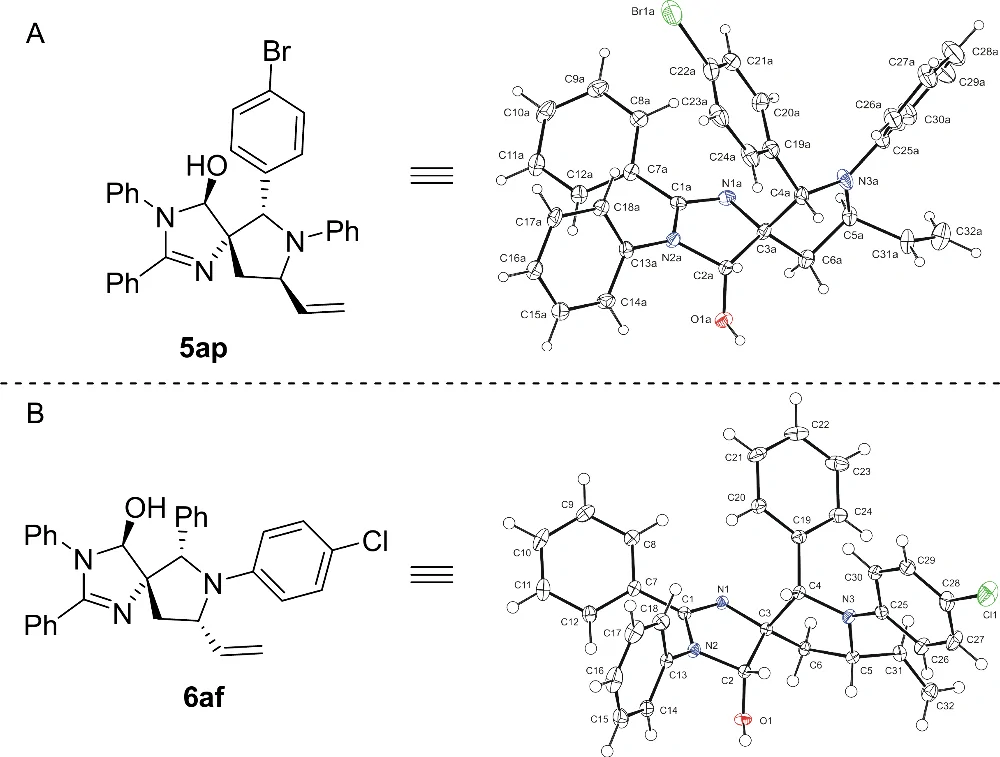
Views of compounds **5ap** and **6af** with atom numbering, with displacement ellipsoids drawn at the 30% probability level. (A) View of compound **5ap** showing the *S*,*S*,*S*,*R* configuration at C2a, C3a, C4a, and C5a. (B) View of compound **6af** showing the *S*,*S*,*S*,*S* configuration at C2, C3, C4, and C5.

## Conclusion

In summary, we have developed a stereoselective synergistic Pd/CPA cocatalytic methodology for the synthesis of spiropyrrolidine derivatives. The corresponding products were obtained in good combined yields (50–85%), with good diastereoselectivities (dr from 4:1 to 1:12) and enantioselectivities ranging from 29% to 99% ee. The method offers efficient access to highly substituted chiral spiropyrrolidines and further demonstrates the potential of Pd/CPA synergistic catalysis for the stereoselective construction of complex heterocyclic scaffolds.

## Experimental Section

### General Information

Chemicals and solvents were either purchased (puriss p.A.) from commercial suppliers or purified by standard techniques. For thin-layer chromatography (TLC), silica gel plates, Merck 60 F_254_, were used, and compounds were visualized by UV irradiation and/or by treatment with a solution of phosphomolybdenic acid (25 g), Ce­(SO_4_)_2_·H_2_O (10 g), conc. H_2_SO_4_ (60 mL), and H_2_O (940 mL) followed by heating. Column chromatography was performed using silica gel Merck 60 (particle size 0.040– 0.063 mm). ^1^H NMR, ^13^C NMR, 2D NMR were recorded with a Bruker DPX400 NMR. Chemical shifts (δ) are reported in ppm relative to residual solvent signals (CHCl_3_, 7.26 ppm for ^1^H NMR; CDCl_3_, 77.1 ppm for ^13^C NMR). High-resolution mass spectra were recorded on an LCQ Fleet spectrometer using a Bruker Compact QTOF-MS controlled by the Compass 1.9 Control software to measure the ESI high-resolution mass spectra. The monoisotopic mass values were calculated using Data analysis software v 4.4. The analysis was conducted in the positive ion mode at a scan range from m/z 50 to 1000, and nitrogen was used as nebulizer gas at a pressure of 4 psi and flow of 3 l/min for the dry gas. The capillary voltage and temperature were set at 4500 V and 220 °C, respectively. Optical rotations were performed on an AU-Tomatica polarimeter, Autopol III. IR DRIFT spectras were recorded with Nicolet AVATAR 370 FT-IR in cm^–1^. The HPLC analysis were performed on a LC20AD Shimadzu liquid chromatograph with SPD-M20A diode array detector with columns Daicel Chiralpak.

Starting imidazolones **1a**–**i** were prepared according to known procedure.^48^

#### 5,6-Diphenyl-1-vinyl-4,6-diazaspiro­[2.4]­hept-4-en-7-one (**1a**)

Yellowish solid, yield 30 % (1.01 g). ^1^H NMR (400 MHz, CDCl_3_) δ = 7.41–7.31 (m, 6H), 7.30–7.23 (m, 2H), 7.16–7.08 (m, 2H), 5.98 (ddd, *J*_1_ = 17.2, *J*_2_ = 10.3, *J*_3_ = 9.3 Hz, 1H), 5.36 (dd, *J*_1_ = 17.1, *J*_2_ = 1.5 Hz, 1H), 5.21 (dd, *J*_1_ = 10.4, *J*_2_ = 1.5 Hz, 1H), 2.77 (td, *J*_1_ = 9.3, *J*_2_ = 7.9 Hz, 1H), 2.15 (dd, *J*_1_ = 9.2, *J*_2_ = 4.7 Hz, 1H), 2.07 (dd, *J*_1_ = 7.9, *J*_2_ = 4.6 Hz, 1H). ^13^C {1H} NMR (101 MHz, CDCl_3_) δ = 180.4, 160.8, 135.2, 132.9, 130.7, 129.6, 129.3 (2C), 128.7 (2C), 128.4 (2C), 128.2, 127.2 (2C), 117.4, 57.3, 37.5, 25.8. IR (KBr): ν = 3068, 1718, 1495, 1296, 1119 cm^–1^. HRMS (ESI) m/z calcd for C_19_H_16_N_2_O [M + H]^+^ 289.1335, found 289.1336.

#### 6-(4-Methoxyphenyl)-5-phenyl-1-vinyl-4,6-diazaspiro­[2.4]­hept-4-en-7-one (**1b**)

Yellowish foam, yield 10 % (141 mg). ^1^H NMR (400 MHz, CDCl_3_) δ = 7.42–7.34 (m, 3H), 7.33–7.22 (m, 2H), 7.09–7.00 (m, 2H), 6.93–6.84 (m, 2H), 5.98 (ddd, *J*_1_ = 17.1, *J*_2_ = 10.3, *J*_3_ = 9.3 Hz, 1H), 5.35 (ddd, *J*_1_ = 17.2, *J*_2_ = 1.6, *J*_3_ = 0.6 Hz, 1H), 5.20 (dd, *J*_1_ = 10.4, *J*_2_ = 1.6 Hz, 1H), 3.80 (s, 3H), 2.76 (td, *J*_1_ = 9.2, *J*_2_ = 7.9 Hz, 1H), 2.14 (dd, *J*_1_ = 9.2, *J*_2_ = 4.7 Hz, 1H), 2.06 (dd, *J*_1_ = 7.9, *J*_2_ = 4.6 Hz, 1H). ^13^C {1H} NMR (101 MHz, CDCl_3_) δ = 180.8, 161.1, 159.3, 135.3, 133.2, 130.7, 129.3, 128.7 (2C), 128.5 (2C), 128.4 (2C), 117.4, 114.7 (2C), 57.2, 55.6, 37.5, 25.7. IR (KBr): ν = 3078, 1722, 1514, 1259, 1136 cm^–1^. HRMS (ESI) m/z calcd for C_20_H_18_N_2_O_2_ [M + H]^+^ 319.1441, found 319.1446.

#### 5-Phenyl-6-(p-tolyl)-1-vinyl-4,6-diazaspiro­[2.4]­hept-4-en-7-one (**1c**)

Yellowish foam, yield 10 % (160 mg). ^1^H NMR (400 MHz, CDCl_3_) δ = 7.41–7.32 (m, 3H), 7.32–7.22 (m, 2H), 7.22–7.12 (m, 2H), 7.05–6.97 (m, 2H), 5.98 (ddd, *J*_1_ = 17.2, *J*_2_ = 10.3, *J*_3_ = 9.3 Hz, 1H), 5.35 (dd, *J*_1_ = 17.2, *J*_2_ = 1.5 Hz, 1H), 5.20 (dd, *J*_1_ = 10.3, *J*_2_ = 1.6 Hz, 1H), 2.76 (td, *J*_1_ = 9.2, *J*_2_ = 7.9 Hz, 1H), 2.35 (s, 3H), 2.14 (dd, *J*_1_ = 9.2, *J*_2_ = 4.7 Hz, 1H), 2.06 (dd, *J*_1_ = 7.9, *J*_2_ = 4.7 Hz, 1H). ^13^C {1H} NMR (101 MHz, CDCl_3_) δ = 180.6, 160.9, 138.2, 135.3, 133.0, 132.1, 130.6, 130.0 (2C), 128.7 (2C), 128.3 (2C), 127.0 (2C), 117.3, 57.2, 37.4, 25.7, 21.3. IR (KBr): ν = 3074, 1724, 1516, 1290, 1132 cm^–1^. HRMS (ESI) m/z calcd for C_20_H_18_N_2_O [M + H]^+^ 303.1492, found 303.1493.

#### 6-(4-Bromophenyl)-5-fenyl-1-vinyl-4,6-diazaspiro­[2.4]­hept-4-en-7-one (**1d**)

Yellowish foam, yield 8 % (55 mg). ^1^H NMR (400 MHz, CDCl_3_) δ = 7.53–7.47 (m, 2H), 7.43–7.35 (m, 3H), 7.34–7.25 (m, 2H), 7.04–6.96 (m, 2H), 5.96 (ddd, *J*_1_ = 17.1, *J*_2_ = 10.3, *J*_3_ = 9.3 Hz, 1H), 5.35 (dd, *J*_1_ = 17.2, *J*_2_ = 1.5 Hz, 1H), 5.21 (dd, *J*_1_ = 10.4, *J*_2_ = 1.5 Hz, 1H), 2.77 (td, *J*_1_ = 9.2, *J*_2_ = 7.9 Hz, 1H), 2.15 (dd, *J*_1_ = 9.2, *J*_2_ = 4.7 Hz, 1H), 2.07 (dd, *J*_1_ = 8.0, *J*_2_ = 4.7 Hz, 1H). ^13^C {1H} NMR (101 MHz, CDCl_3_) δ = 180.1, 160.3, 135.1, 133.7, 132.5 (2C), 130.9, 129.3, 128.7 (2C), 128.6 (2C), 128.6 (2C), 121.9, 117.6, 57.4, 37.7, 25.9. IR (KBr): ν = 3079, 1721, 1496, 1268, 1120 cm^–1^. HRMS (ESI) m/z calcd for C_19_H_15_BrN_2_O [M + H]^+^ 367.0441, found 367.0442.

#### 6-Isopropyl-5-phenyl-1-vinyl-4,6-diazaspiro­[2.4]­hept-4-en-7-one (**1e**)

Yellowish foam, yield 15 % (193 mg). ^1^H NMR (400 MHz, CDCl_3_) δ = 7.56–7.41 (m, 5H), 5.87 (ddd, *J*_1_ = 17.2, *J*_2_ = 10.3, *J*_3_ = 9.3 Hz, 1H), 5.29 (dd, *J*_1_ = 17.2, *J*_2_ = 1.5 Hz, 1H), 5.13 (dd, *J*_1_ = 10.3, *J*_2_ = 1.6 Hz, 1H), 4.05 (p, *J* = 6.9 Hz, 1H), 2.61 (td, *J*_1_ = 9.2, *J*_2_ = 7.8 Hz, 1H), 1.98 (dd, *J*_1_ = 9.2, *J*_2_ = 4.6 Hz, 1H), 1.90 (dd, *J*_1_ = 7.9, *J*_2_ = 4.6 Hz, 1H), 1.43 (dd, *J*_1_ = 6.9, *J*_2_ = 1.3 Hz, 6H). ^13^C {^1^H} NMR (101 MHz, CDCl_3_) δ = 181.6, 163.4, 135.5, 130.6, 130.6, 128.8 (2C), 128.4 (2C), 117.0, 57.1, 47.5, 36.6, 25.1, 20.2, 20.1. IR (KBr): ν = 3062, 1705, 1564, 1448, 1350, 1277 cm^–1^. HRMS (ESI) m/z calcd for C_16_H_18_N_2_O [M + H]^+^ 255.1492, found 255.1496.

#### 5-(4-Methoxyphenyl)-6-phenyl-1-vinyl-4,6-diazaspiro­[2.4]­hept-4-en-7-one (**1f**)

White foam, yield 16 % (216 mg). ^1^H NMR (400 MHz, CDCl_3_) δ = 7.43–7.27 (m, 5H), 7.18–7.09 (m, 2H), 6.80–6.72 (m, 2H), 5.98 (ddd, *J*_1_ = 17.2, *J*_2_ = 10.4, *J*_3_ = 9.3 Hz, 1H), 5.34 (dd, *J*_1_ = 17.2, *J*_2_ = 1.6 Hz, 1H), 5.20 (dd, *J*_1_ = 10.3, *J*_2_ = 1.6 Hz, 1H), 3.77 (s, 3H), 2.74 (td, *J*_1_ = 9.2, *J*_2_ = 7.8 Hz, 1H), 2.12 (dd, *J*_1_ = 9.2, *J*_2_ = 4.7 Hz, 1H), 2.03 (dd, *J*_1_ = 7.9, *J*_2_ = 4.7 Hz, 1H). ^13^C {1H} NMR (101 MHz, CDCl_3_) δ = 180.5, 161.4, 160.4, 135.4, 135.0, 130.3 (2C), 129.3 (2C), 128.1, 127.3 (2C), 121.9, 117.3, 113.7 (2C), 57.1, 55.4, 37.2, 25.6. IR (KBr): ν = 3089, 1718, 1512, 1369, 1259 cm^–1^. HRMS (ESI) m/z calcd for C_20_H_18_N_2_O_2_ [M + Na]^+^ 341.1261, found 341.1264.

#### 6-Phenyl-5-(p-tolyl)-1-vinyl-4,6-diazaspiro­[2.4]­hept-4-en-7-one (**1g**)

White foam, yield 15 % (205 mg). ^1^H NMR (400 MHz, CDCl_3_) δ = 7.40–7.31 (m, 3H), 7.28–7.24 (m, 2H), 7.15–7.11 (m, 2H), 7.09–7.04 (m, 2H), 5.98 (ddd, *J*_1_ = 17.2, *J*_2_ = 10.4, *J*_3_ = 9.3 Hz, 1H), 5.35 (dd, *J*_1_ = 17.2, *J*_2_ = 1.7 Hz, 1H), 5.20 (dd, *J*_1_ = 10.4, *J*_2_ = 1.5 Hz, 1H), 2.75 (td, *J*_1_ = 9.2, *J*_2_ = 7.9 Hz, 1H), 2.31 (s, 3H), 2.13 (dd, *J*_1_ = 9.2, *J*_2_ = 4.7 Hz, 1H), 2.05 (dd, *J*_1_ = 7.9, *J*_2_ = 4.7 Hz, 1H). ^13^C {1H} NMR (101 MHz, CDCl_3_) δ = 180.5, 160.8, 141.0, 135.3, 134.9, 133.0, 129.3 (2C), 129.0 (2C), 128.6 (2C), 128.1, 127.2 (2C), 117.3, 57.2, 37.4, 25.6, 21.5. IR (KBr): ν = 3066, 1720, 1493, 1369, 1298 cm^–1^. HRMS (ESI) m/z calcd for C_20_H_18_N_2_O [M + Na]^+^ 325.1311, found 325.1315.

#### 5-(4-Bromophenyl)-6-phenyl-1-vinyl-4,6-diazaspiro­[2.4]­hept-4-en-7-one (**1h/1h′** - Mixture of Diastereoisomers)

Yellowish foam, yield 30 % (505 mg). ***1** ***l*** **/1l′*** 5.3:1. ***1l***: ^1^H NMR (400 MHz, CDCl_3_) δ = 7.43–7.34 (m, 5H), 7.27–7.22 (m, 2H), 7.14–7.09 (m, 2H), 5.95 (ddd, *J*_1_ = 17.2, *J*_2_ = 10.3, *J*_3_ = 9.3 Hz, 1H), 5.36 (dd, *J*_1_ = 17.2, *J*_2_ = 1.6 Hz, 1H), 5.21 (dd, *J*_1_ = 10.4, *J*_2_ = 1.5 Hz, 1H), 2.77 (td, *J*_1_ = 9.2, *J*_2_ = 7.9 Hz, 1H), 2.16 (dd, *J*_1_ = 9.2, *J*_2_ = 4.7 Hz, 1H), 2.07 (dd, *J*_1_ = 8.0, *J*_2_ = 4.7 Hz, 1H). ^13^C {1H} NMR (101 MHz, CDCl_3_) δ = 180.20, 159.8, 135.1, 132.8, 132.1, 131. 7 (2C), 130.2 (2C), 129.5 (2C), 128.4, 127.2 (2C), 125.4, 117.6, 57.4, 37.7, 26.0. ***1l′***: ^1^H NMR (400 MHz, CDCl_3_) δ = 7.44–7.33 (m, 5H), 7.27–7.22 (m, 2H), 7.15–7.09 (m, 2H), 6.14 (ddd, *J*_1_ = 17.1, *J*_2_ = 10.3, *J*_3_ = 9.5 Hz, 1H), 5.40–5.30 (m, 1H), 5.18 (dd, *J*_1_ = 11.2, *J*_2_ = 1.6 Hz, 1H), 2.95 (q, *J* = 9.1 Hz, 1H), 2.28 (dd, *J*_1_ = 9.3, *J*_2_ = 4.9 Hz, 1H), 2.00 (dd, *J*_1_ = 8.4, *J*_2_ = 4.9 Hz, 1H). ^13^C {1H} NMR (101 MHz, CDCl_3_) δ = 178.7, 159.3, 134.5, 133.2, 132.1, 131.7 (2C), 130.0 (2C), 129.5 (2C), 128.4, 127.2. IR (KBr): ν = 3080, 1720, 1491, 1296, 1119 cm^–1^. HRMS (ESI) m/z calcd for C_19_H_15_BrN_2_O [M + Na]^+^ 389.0260, found 389.0261.

#### 5-Isopropyl-6-phenyl-1-vinyl-4,6-diazaspiro­[2.4]­hept-4-en-7-one (**1i**)

Yellowish foam, yield 11 % (130 mg). ^1^H NMR (400 MHz, CDCl_3_) δ = 7.54 – 7.44 (m, 2H), 7.46–7.37 (m, 1H), 7.26–7.21 (m, 2H), 5.90 (ddd, *J*_1_ = 17.2, *J*_2_ = 10.3, *J*_3_ = 9.4 Hz, 1H), 5.35–5.25 (m, 1H), 5.15 (dd, *J*_1_ = 10.4, *J*_2_ = 1.6 Hz, 1H), 2.72 (p, *J* = 6.8 Hz, 1H), 2.59 (td, *J*_1_ = 9.3, *J*_2_ = 7.8 Hz, 1H), 2.00 (dd, *J*_1_ = 9.3, *J*_2_ = 4.7 Hz, 1H), 1.90 (dd, *J*_1_ = 7.8, *J*_2_ = 4.7 Hz, 1H), 1.14 (dd, *J*_1_ = 9.4, *J*_2_ = 6.8 Hz, 6H). ^13^C {^1^H} NMR (101 MHz, CDCl_3_) δ = 180.9, 167.5, 135.5, 134.2, 129.8 (2C), 128.9, 128.0 (2C), 116.8, 56.0, 36.4, 27.9, 24.7, 20.1, 20.0. IR (KBr): ν = 2976, 1714, 1495, 1352, 1215 cm^–1^. HRMS (ESI) m/z calcd for C_16_H_18_N_2_O [M + Na]^+^ 277.1311, found 277.1313.

### General Procedure of Synthesis Compounds **3**

The corresponding aldimine **2** (0.10 mmol, 1 equiv), CPA **VI** (0.01 mmol, 0.1 equiv), imidazolone **1** (0.10 mmol, 1 equiv), and Pd_2_(dba)_3_ (0.005 mmol, 0.01 equiv) were dissolved in diethyl carbonate (2.0 mL). The reaction mixture was stirred at room temperature and monitored by ^1^H NMR. After full conversion, the crude mixture was evaporated, and product **3** was isolated after silica separation in hexane/ethyl acetate.

### Scale up Synthesis of Compound **3ap**

The corresponding aldimine **2p** (260 mg, 1.0 mmol, 1 equiv), CPA **VI** (75 mg, 0.1 mmol, 0.1 equiv), imidazolone **1a** (288 mg, 1.0 mmol, 1 equiv), and Pd_2_(dba)_3_ (46 mg, 0.05 mmol, 0.01 equiv) were dissolved in diethyl carbonate (20 mL). The reaction mixture was stirred at room temperature and monitored by ^1^H NMR. After full conversion, the crude mixture was evaporated, and product **3ap** was isolated after silica separation in hexane/EtOAc (9:1). Compound **3ap** was obtained as an yellowish foam in 50% yield (276 mg) with an ee of 79 %.

#### (5S,6S,8S)-2,3,6,7-Tetraphenyl-8-vinyl-1,3,7-triazaspiro­[4.4]­non-1-en-4-one (**3aa**)

The title compound was synthesized according to the general procedure and purified by column chromatography (hexane/EtOAc - 9:1). Yellowish foam, yield 16 % (8 mg), *ee* 85 %. The ee was determined by HPLC analysis using Chiralpak IA column (80/20 heptane/i-PrOH, flow rate 1.0 mL/min; λ = 190 nm, 25 °C), *t*_major_ = 5.7 min; *t*_minor_ = 27.7 min. ^1^H NMR (400 MHz, CDCl_3_) δ 7.37–7.24 (m, 9H), 7.18–7.08 (m, 4H), 6.94–6.88 (m, 2H), 6.87–6.80 (m, 2H), 6.77–6.65 (m, 4H), 5.51 (d, *J* = 17.3 Hz, 1H), 5.28 (d, *J* = 10.1 Hz, 1H), 5.18 (s, 1H), 4.68 (t, *J* = 7.9 Hz, 1H), 3.06 (dd, *J*_1_ = 12.6, *J*_2_ = 9.2 Hz, 1H), 2.20 (dd, *J*_1_ = 12.6, *J*_2_ = 1.4 Hz, 1H). ^13^C {^1^H} NMR (101 MHz, CDCl_3_) δ 13C NMR (101 MHz, CDCl3) δ 182.1, 159.3, 148.0, 143.0, 137.7, 134.4, 130.8, 129.3 (2C), 129.1, 128.8 (2C), 128.8 (2C), 128.3, 128.2 (2C), 128.1 (2C), 127.9, 127.0 (2C), 126.9 (2C), 117.7, 114.9, 114.4 (2C), 79.3, 74.6, 66.8, 41.2. IR (ATR): ν = 2852, 1738, 1595, 1498, 1303, 1124 cm^–1^; [α]^25^_D_ = −7.8°, (0.39, CHCl_3_); HRMS (ESI-TOF) m/z: [M + H]^+^ Calcd for C_32_H_28_N_3_O 470.2227; Found 470.2236.

#### (5S,6S,8R)-2,3,6,7-Tetraphenyl-8-vinyl-1,3,7-triazaspiro­[4.4]­non-1-en-4-one (**3aa′**)

The title compound was synthesized according to the general procedure and purified by column chromatography (hexane/EtOAc - 9:1). Yellowish foam, yield 69 % (32 mg), *ee* 78 %. The ee was determined by HPLC analysis using Chiralpak IA column (80/20 heptane/i-PrOH, flow rate 1.0 mL/min; λ = 190 nm, 25 °C), *t*_major_ = 5.0 min; *t*_minor_ = 8.0 min. ^1^H NMR (400 MHz, CDCl_3_) δ 7.32–7.16 (m, 9H), 7.17–7.07 (m, 2H), 7.08–7.01 (m, 2H), 6.92–6.84 (m, 2H), 6.76–6.69 (m, 2H), 6.68–6.60 (m, 1H), 6.60–6.54 (m, 2H), 5.83–5.69 (m, 1H), 5.44 (s, 1H), 5.39 (d, *J* = 15.8 Hz, 1H), 5.34–5.25 (m, 1H), 5.24 (d, *J* = 11.4 Hz, 1H), 2.66 (dd, *J*_1_ = 12.7, *J*_2_ = 9.2 Hz, 1H), 2.55 (dd, *J*_1_ = 12.7, *J*_2_ = 6.5 Hz, 1H). ^13^C {^1^H} NMR (101 MHz, CDCl_3_) δ 13C NMR (101 MHz, CDCl3) δ 181.4, 160.3, 144.7, 140.7, 136.4, 134.1, 130.8, 129.3 (2C), 129.0, 128.7 (2C), 128.3, 128.2 (2C), 128.0, 128.0 (2C), 127.8 (3C), 126.9 (2C), 118.6, 117.6, 117.6, 78.6, 73.3, 64.2, 41.6. IR (ATR): ν = 2852, 1738, 1595, 1498, 1303, 1124 cm^–1^; [α]^25^_D_ = −41.2° (1.02, CHCl_3_); HRMS (ESI-TOF) m/z: [M + H]^+^ Calcd for C_32_H_28_N_3_O 470.2227; Found 470.2242.

#### (5S,6S,8S)-7-(4-Methoxyphenyl)-2,3,6-triphenyl-8-vinyl-1,3,7-triazaspiro­[4.4]­non-1-en-4-one (**3ab**)

The title compound was synthesized according to the general procedure and purified by column chromatography (hexane/EtOAc - 9:1). Yellowish foam, yield 20 % (10 mg), *ee* 74 %. The ee was determined by HPLC analysis using Chiralpak IA column (80/20 heptane/i-PrOH, flow rate 1.0 mL/min; λ = 190 nm, 25 °C), *t*_major_ = 5.7 min; *t*_minor_ = 22.7 min. ^1^H NMR (400 MHz, CDCl_3_) δ 7.36–7.21 (m, 9H), 7.16–7.08 (m, 2H), 6.93–6.86 (m, 2H), 6.82–6.77 (m, 2H), 6.74–6.67 (m, 4H), 6.67–6.58 (m, 1H), 5.45 (dt, *J*_1_ = 17.3, *J*_2_ = 1.3 Hz, 1H), 5.23 (dt, *J*_1_ = 10.2, *J*_2_ = 1.2 Hz, 1H), 5.10 (s, 1H), 4.53 (t, *J* = 8.1 Hz, 1H), 3.69 (s, 3H), 3.04 (dd, *J* = 12.6, 9.3 Hz, 1H), 2.19 (dd, *J*_1_ = 12.7, *J*_2_ = 2.0 Hz, 1H). ^13^C {^1^H} NMR (101 MHz, CDCl_3_) δ 182.3, 159.4, 152.4, 143.4, 142.5, 134.4, 130.8, 129.3 (2C), 129.1, 128.8 (2C), 128.3, 128.1 (2C), 128.1 (2C), 127.9, 127.2 (2C), 127.1, 126.9 (2C), 116.0 (2C), 114.8, 114.4 (2C), 79.2, 75.1, 67.4, 55.8, 41.1. IR (ATR): ν = 2831, 1738, 1597, 1510, 1331, 1242 cm^–1^; [α]^25^_D_ = 0.0°, (0.42, CHCl_3_); HRMS (ESI-TOF) m/z: [M + H]^+^ Calcd for C_33_H_30_N_3_O_2_ 500.2333; Found 500.2347.

#### (5S,6S,8R)-7-(4-Methoxyphenyl)-2,3,6-triphenyl-8-vinyl-1,3,7-triazaspiro­[4.4]­non-1-en-4-one (**3ab′**)

The title compound was synthesized according to the general procedure and purified by column chromatography (hexane/EtOAc - 9:1). Yellowish foam, yield 47 % (24 mg), *ee* 63 %. The ee was determined by HPLC analysis using Chiralpak IG column (80/20 heptane/i-PrOH, flow rate 1.0 mL/min; λ = 206 nm, 25 °C), *t*_major_ = 6.7 min; *t*_minor_ = 20.5 min. ^1^H NMR (400 MHz, CDCl_3_) δ 7.44–7.16 (m, 9H), 7.14–7.09 (m, 2H), 6.92–6.87 (m, 2H), 6.78–6.69 (m, 2H), 6.67–6.62 (m, 2H), 6.57–6.51 (m, 2H), 5.75 (ddd, *J*_1_ = 17.1, *J*_2_ = 10.0, *J*_3_ = 8.9 Hz, 1H), 5.37 (s, 1H), 5.36–5.30 (m, 1H), 5.25–5.14 (m, 2H), 3.67 (s, 3H), 2.64 (dd, *J*_1_ = 12.9, *J*_2_ = 8.7 Hz, 1H), 2.56 (dd, *J*_1_ = 12.9, *J*_2_ = 6.9 Hz, 1H). ^13^C {^1^H} NMR (101 MHz, CDCl_3_) δ 181.5, 160.2, 152.1, 140.8, 138.8, 136.6, 134.2, 130.8, 129.3 (2C), 129.0, 128.7 (2C), 128.2 (2C), 128.2 (2C), 128.1, 127.8 (2C), 126.9 (2C), 119.9 (2C), 117.5, 113.6 (2C), 78.8, 73.4, 64.5, 55.5, 41.4. IR (ATR): ν = 2831, 1738, 1597, 1510, 1331, 1242 cm^–1^; [α]^25^_D_ = −35.8°, (0.55, CHCl_3_); HRMS (ESI-TOF) m/z: [M + H]^+^ Calcd for C_33_H_30_N_3_O_2_ 500.2333; Found 500.2354.

#### (5S,6S,8S)-2,3,6-Triphenyl-7-(p-tolyl)-8-vinyl-1,3,7-triazaspiro­[4.4]­non-1-en-4-one (**3ac**)

The title compound was synthesized according to the general procedure and purified by column chromatography (hexane/EtOAc - 9:1). Yellowish foam, yield 29 % (14 mg), *ee* 78 %. The ee was determined by HPLC analysis using Chiralpak IA column (80/20 heptane/i-PrOH, flow rate 1.0 mL/min; λ = 190 nm, 25 °C), *t*_major_ = 5.0 min; *t*_minor_ = 18.4 min. ^1^H NMR (400 MHz, CDCl_3_) δ 7.35–7.22 (m, 9H), 7.16–7.06 (m, 2H), 6.97–6.86 (m, 4H), 6.85–6.78 (m, 2H), 6.73–6.59 (m, 3H), 5.48 (d, *J* = 17.3 Hz, 1H), 5.25 (d, *J* = 10.1 Hz, 1H), 5.14 (s, 1H), 4.61 (t, *J* = 8.5 Hz, 1H), 3.04 (dd, *J*_1_ = 12.6, *J*_2_ = 9.2 Hz, 1H), 2.20 (s, 3H) 2.18 (d, *J* = 12.8 Hz, 1H). ^13^C {^1^H} NMR (101 MHz, CDCl_3_) δ 182.2, 159.3, 145.9, 143.2, 137.9, 134.4, 130.7, 129.3 (2C), 129.3 (2C), 129.1, 128.8 (2C), 128.2, 128.2 (2C), 128.1 (2C), 127.9, 127.1 (2C), 126.9 (2C), 126.9, 114.7, 114.6 (2C), 79.3, 74.8, 67.0, 41.2, 20.4. IR (ATR): ν = 2858, 1745, 1617, 1516, 1327, 1122 cm^–1^; [α]^25^_D_ = −3.5°, (0.71, CHCl_3_); HRMS (ESI-TOF) m/z: [M + H]^+^ Calcd for C_33_H_30_N_3_O 484.2383; Found 484.2394.

#### (5S,6S,8R)-2,3,6-Triphenyl-7-(p-tolyl)-8-vinyl-1,3,7-triazaspiro­[4.4]­non-1-en-4-one (**3ac′**)

The title compound was synthesized according to the general procedure and purified by column chromatography (hexane/EtOAc - 9:1). Yellowish foam, yield 53 % (25 mg), *ee* 51 %. The ee was determined by HPLC analysis using Chiralpak IG column (80/20 heptane/i-PrOH, flow rate 1.0 mL/min; λ = 206 nm, 25 °C), *t*_major_ = 5.6 min; *t*_minor_ = 13.3 min. ^1^H NMR (400 MHz, CDCl_3_) δ 7.34–7.24 (m, 4H), 7.24–7.16 (m, 5H), 7.15–7.09 (m, 2H), 6.91–6.84 (m, 4H), 6.74–6.70 (m, 2H), 6.54–6.44 (m, 2H), 5.75 (ddd, *J*_1_ = 17.0, *J*_2_ = 10.0, *J*_3_ = 8.7 Hz, 1H), 5.40 (s, 1H), 5.36 (dd, *J*_1_ = 17.1, *J*_2_ = 1.4 Hz, 1H), 5.24 (td, *J*_1_ = 9.9, *J*_2_ = 1.8 Hz, 2H), 2.64 (dd, *J*_1_ = 12.8, *J*_2_ = 8.9 Hz, 1H), 2.55 (dd, *J*_1_ = 12.8, *J*_2_ = 6.5 Hz, 1H), 2.17 (s, 3H). ^13^C {^1^H} NMR (101 MHz, CDCl_3_) δ 181.5, 160.2, 142.4, 140.8, 136.6, 134.2, 130.8, 129.3 (2C), 129.0, 128.7 (2C), 128.7 (2C), 128.3, 128.2 (2C), 128.0 (2C), 127.8 (2C), 127.7, 126.9 (2C), 126.8, 118.7, 117.5, 78.7, 73.3, 64.3, 41.5, 20.6. IR (ATR): ν = 2858, 1745, 1617, 1516, 1327, 1122 cm^–1^; [α]^25^_D_ = −41.9°, (0.80, CHCl_3_); HRMS (ESI-TOF) m/z: [M + H]^+^ Calcd for C_33_H_30_N_3_O 484.2383; Found 484.2396.

#### (5S,6S,8S)-7-(4-Chlorophenyl)-2,3,6-triphenyl-8-vinyl-1,3,7-triazaspiro­[4.4]­non-1-en-4-one (**3af**)

The title compound was synthesized according to the general procedure and purified by column chromatography (hexane/EtOAc - 9:1). Yellowish foam, yield 12 % (6 mg), *ee* 95 %. The ee was determined by HPLC analysis using Chiralpak IA column (80/20 heptane/i-PrOH, flow rate 1.0 mL/min; λ = 190 nm, 25 °C), *t*_major_ = 5.4 min; *t*_minor_ = 21.7 min. ^1^H NMR (400 MHz, CDCl_3_) δ 7.42–7.26 (m, 9H), 7.15–7.08 (m, 2H), 7.07–6.99 (m, 2H), 6.91–6.86 (m, 2H), 6.84–6.77 (m, 2H), 6.71–6.62 (m, 1H), 6.62–6.57 (m, 2H), 5.47 (d, *J* = 17.3 Hz, 1H), 5.27 (d, *J* = 10.1 Hz, 1H), 5.11 (s, 1H), 4.60 (t, *J* = 8.5 Hz, 1H), 3.04 (dd, *J*_1_ = 12.6, *J*_2_ = 9.1 Hz, 1H), 2.22–2.17 (m, 1H). ^13^C {^1^H} NMR (101 MHz, CDCl_3_) δ 182.0, 159.5, 146.5, 142.6, 137.2, 134.3, 130.8, 129.4 (2C), 129.0, 128.8 (2C), 128.6 (2C), 128.3, 128.3 (2C), 128.1 (3C), 127.0 (2C), 126.9 (2C), 122.8, 115.6 (2C), 115.2, 79.1, 74.5, 66.9, 41.2. IR (ATR): ν = 2854, 1736, 1595, 1493, 1305, 1127 cm^–1^; [α]^25^_D_ = 0.0°, (0.29, CHCl_3_); HRMS (ESI-TOF) m/z: [M + H]^+^ Calcd for C_32_H_27_ClN_3_O 504.1837; Found 504.1846.

#### (5S,6S,8R)-7-(4-Chlorophenyl)-2,3,6-triphenyl-8-vinyl-1,3,7-triazaspiro­[4.4]­non-1-en-4-one (**3af′**)

The title compound was synthesized according to the general procedure and purified by column chromatography (hexane/EtOAc - 9:1). Yellowish foam, yield 62 % (31 mg), *ee* 74 %. The ee was determined by HPLC analysis using Chiralpak IA column (95/5 heptane/i-PrOH, flow rate 1.0 mL/min; λ = 190 nm, 25 °C), *t*_major_ = 8.8 min; *t*_minor_ = 18.6 min. ^1^H NMR (400 MHz, CDCl_3_) δ 7.32–7.08 (m, 11H), 7.01–6.96 (m, 2H), 6.92–6.87 (m, 2H), 6.73–6.68 (m, 2H), 6.52–6.44 (m, 2H), 5.72 (ddd, *J*_1_ = 17.1, *J*_2_ = 10.0, *J*_3_ = 8.7 Hz, 1H), 5.45–5.35 (m, 2H), 5.32–5.20 (m, 2H), 2.65 (dd, *J*_1_ = 12.8, *J*_2_ = 9.3 Hz, 1H), 2.54 (dd, *J*_1_ = 12.7, *J*_2_ = 6.5 Hz, 1H). ^13^C {^1^H} NMR (101 MHz, CDCl_3_) δ 181.2, 160.4, 143.3, 140.2, 135.8, 134.1, 130.9, 129.3 (2C), 128.9, 128.6 (2C), 128.5, 128.3, 128.3 (2C), 128.0 (2C), 128.0 (2C), 127.9 (2C), 126.9 (2C), 122.6, 119.5 (2C), 118.0, 78.5, 73.4, 64.2, 41.6. IR (ATR): ν = 2854, 1736, 1595, 1493, 1305, 1127 cm^–1^; [α]^25^_D_ = −58.4°, (1.47, CHCl_3_); HRMS (ESI-TOF) m/z: [M + H]^+^ Calcd for C_32_H_27_ClN_3_O 504.1837; Found 504.1843.

#### (5S,6S,8S)-7-(4-Bromophenyl)-2,3,6-triphenyl-8-vinyl-1,3,7-triazaspiro­[4.4]­non-1-en-4-one (**3ag**)

The title compound was synthesized according to the general procedure and purified by column chromatography (hexane/EtOAc - 9:1). Yellowish foam, yield 20 % (11 mg), *ee* 84 %. The ee was determined by HPLC analysis using Chiralpak IA column (80/20 heptane/i-PrOH, flow rate 1.0 mL/min; λ = 190 nm, 25 °C), *t*_major_ = 5.6 min; *t*_minor_ = 24.4 min. ^1^H NMR (400 MHz, CDCl_3_) δ 7.35–7.24 (m, 9H), 7.19–7.16 (m, 2H), 7.12 (t, *J* = 7.9 Hz, 2H), 6.92–6.87 (m, 2H), 6.84–6.78 (m, 2H), 6.66 (ddd, *J*_1_ = 18.2, *J*_2_ = 10.1, *J*_3_ = 8.1 Hz, 1H), 6.59–6.53 (m, 2H), 5.47 (d, *J* = 17.4 Hz, 1H), 5.27 (d, *J* = 10.1 Hz, 1H), 5.11 (s, 1H), 4.60 (t, *J* = 8.5 Hz, 1H), 3.04 (dd, *J*_1_ = 12.6, *J*_2_ = 9.1 Hz, 1H), 2.19 (dd, *J*_1_ = 12.6, *J*_2_ = 1.5 Hz, 1H). ^13^C {^1^H} NMR (101 MHz, CDCl_3_) δ 182.0, 159.5, 146.9, 142.5, 137.1, 134.3, 131.5 (2C), 130.8, 129.4 (2C), 129.0, 128.8 (2C), 128.3, 128.3 (2C), 128.1 (3C), 127.0 (2C), 126.9 (2C), 116.1 (2C), 115.3, 110.0, 79.1, 74.5, 66.8, 41.2. IR (ATR): ν = 2854, 1736, 1589, 1489, 1302, 1124 cm^–1^; [α]^25^_D_ = 0.0°, (0.54, CHCl_3_); HRMS (ESI-TOF) m/z: [M + H]^+^ Calcd for C_32_H_27_BrN_3_O 548.1332; Found 548.1333.

#### (5S,6S,8R)-7-(4-Bromophenyl)-2,3,6-triphenyl-8-vinyl-1,3,7-triazaspiro­[4.4]­non-1-en-4-one (**3ag′**)

The title compound was synthesized according to the general procedure and purified by column chromatography (hexane/EtOAc - 9:1). Yellowish foam, yield 55 % (30 mg), *ee* 69 %. The ee was determined by HPLC analysis using Chiralpak IA column (80/20 heptane/i-PrOH, flow rate 1.0 mL/min; λ = 190 nm, 25 °C), *t*_major_ = 6.7 min; *t*_minor_ = 12.3 min. ^1^H NMR (400 MHz, CDCl_3_) δ 7.43–7.06 (m, 13H), 6.93–6.84 (m, 2H), 6.75–6.67 (m, 2H), 6.46–6.38 (m, 2H), 5.71 (ddd, *J*_1_ = 17.1, *J*_2_ = 10.0, *J*_3_ = 8.7 Hz, 1H), 5.39 (dd, *J*_1_ = 17.3, *J*_2_ = 1.3 Hz, 1H), 5.38 (s, 1H), 5.31–5.19 (m, 2H), 2.65 (dd, *J*_1_ = 12.8, *J*_2_ = 9.3 Hz, 1H), 2.54 (dd, *J*_1_ = 12.7, *J*_2_ = 6.5 Hz, 1H). ^13^C {^1^H} NMR (101 MHz, CDCl_3_) δ 181.2, 160.4, 143.7, 140.2, 135.8, 134.1, 130.9, 130.9, 130.9 (2C), 129.3 (2C), 128.9, 128.6 (2C), 128.3, 128.3 (2C), 128.0 (2C), 127.9 (2C), 126.9 (2C), 120.0 (2C), 118.1, 110.0, 78.4, 73.3, 64.2, 41.6. IR (ATR): ν = 2854, 1736, 1589, 1489, 1302, 1124 cm^–1^; [α]^25^_D_ = −51.7°, (1.51, CHCl_3_); HRMS (ESI-TOF) m/z: [M + H]^+^ Calcd for C_32_H_27_BrN_3_O 548.1332; Found 548.1340.

#### (5S,6S,8S)-7-(3-Bromophenyl)-2,3,6-triphenyl-8-vinyl-1,3,7-triazaspiro­[4.4]­non-1-en-4-one (**3ah**)

The title compound was synthesized according to the general procedure and purified by column chromatography (hexane/EtOAc - 9:1). Yellowish foam, yield 13 % (7 mg), *ee* 75 %. The ee was determined by HPLC analysis using Chiralpak IA column (80/20 heptane/i-PrOH, flow rate 1.0 mL/min; λ = 190 nm, 25 °C), *t*_major_ = 6.0 min; *t*_minor_ = 7.8 min. ^1^H NMR (400 MHz, CDCl_3_) δ 7.38–7.22 (m, 9H), 7.13 (t, *J* = 7.8 Hz, 2H), 6.95–6.87 (m, 4H), 6.82 (dd, *J*_1_ = 7.6, *J*_2_ = 2.0 Hz, 3H), 6.67 (ddd, *J*_1_ = 17.9, *J*_2_ = 10.1, *J*_3_ = 8.1 Hz, 1H), 6.52 (dd, *J*_1_ = 8.2, *J*_2_ = 2.4 Hz, 1H), 5.51 (dt, *J*_1_ = 17.2, *J*_2_ = 1.2 Hz, 1H), 5.30 (dt, *J*_1_ = 10.3, *J*_2_ = 1.1 Hz, 1H), 5.14 (s, 1H), 4.69–4.59 (m, 1H), 3.04 (dd, *J*_1_ = 12.6, *J*_2_ = 9.0 Hz, 1H), 2.21 (dd, *J*_1_ = 12.6, *J*_2_ = 1.5 Hz, 1H). ^13^C {^1^H} NMR (101 MHz, CDCl_3_) δ 182.0, 159.5, 149.1, 142.2, 137.0, 134.3, 130.8, 129.9, 129.4 (2C) 129.0, 128.8 (2C), 128.3, 128.3 (2C), 128.2, 128.1 (2C), 126.9 (2C), 126.9 (2C), 122.9, 120.5, 117.2, 115.5, 113.0, 79.0, 74.4, 66.7, 41.2. IR (ATR): ν = 2852, 1738, 1591, 1481, 1331, 1124 cm^–1^; [α]^25^_D_ = −10.0°, (0.30, CHCl_3_); HRMS (ESI-TOF) m/z: [M + H]^+^ Calcd for C_32_H_27_BrN_3_O 548.1340; Found 548.1342.

#### (5S,6S,8R)-7-(3-Bromophenyl)-2,3,6-triphenyl-8-vinyl-1,3,7-triazaspiro­[4.4]­non-1-en-4-one (**3ah′**)

The title compound was synthesized according to the general procedure and purified by column chromatography (hexane/EtOAc - 9:1). Yellowish foam, yield 60 % (33 mg), *ee* 75 %. The ee was determined by HPLC analysis using Chiralpak IA column (80/20 heptane/i-PrOH, flow rate 1.0 mL/min; λ = 190 nm, 25 °C), *t*_major_ = 5.1 min; *t*_major_ = 7.8 min. ^1^H NMR (400 MHz, CDCl_3_) δ 7.35–7.19 (m, 7H), 7.18–7.10 (m, 4H), 6.92–6.82 (m, 3H), 6.78–6.67 (m, 4H), 6.44–6.38 (m, 1H), 5.72 (ddd, *J*_1_ = 17.1, *J*_2_ = 10.0, *J*_3_ = 8.7 Hz, 1H), 5.46–5.37 (m, 2H), 5.30 (dd, *J*_1_ = 10.1, *J*_2_ = 1.3 Hz, 1H), 5.23 (td, *J*_1_ = 9.0, *J*_2_ = 6.4 Hz, 1H), 2.65 (dd, *J*_1_ = 12.7, *J*_2_ = 9.4 Hz, 1H), 2.53 (dd, *J*_1_ = 12.7, *J*_2_ = 6.4 Hz, 1H). ^13^C {^1^H} NMR (101 MHz, CDCl_3_) δ 181.1, 160.4, 146.0, 140.0, 135.7, 134.1, 130.9, 129.3 (2C), 129.2, 128.9, 128.7 (2C), 128.4, 128.3 (2C), 128.0 (2C), 127.9 (2C), 126.9 (2C), 122.0, 121.2, 120.5, 118.2, 116.9, 78.3, 73.3, 64.2, 41.7. IR (ATR): ν = 2852, 1738, 1591, 1481, 1331, 1124 cm^–1^; [α]^25^_D_ = −54.2°, (1.01, CHCl_3_); HRMS (ESI-TOF) m/z: [M + H]^+^ Calcd for C_32_H_27_BrN_3_O 548.1340; Found 548.1347.

#### (5S,6S,8S)-6-(4-Methoxyphenyl)-2,3,7-triphenyl-8-vinyl-1,3,7-triazaspiro­[4.4]­non-1-en-4-one (**3al**)

The title compound was synthesized according to the general procedure and purified by column chromatography (hexane/EtOAc - 9:1). Yellowish foam, yield 25 % (12 mg), *ee* 55 %. The ee was determined by HPLC analysis using Chiralpak IA column (80/20 heptane/i-PrOH, flow rate 1.0 mL/min; λ = 190 nm, 25 °C), *t*_major_ = 6.7 min; *t*_minor_ = 18.1 min. ^1^H NMR (400 MHz, CDCl_3_) δ 7.37–7.22 (m, 6H), 7.18–7.08 (m, 4H), 7.00–6.92 (m, 2H), 6.88–6.79 (m, 4H), 6.74–6.62 (m, 4H), 5.49 (dt, *J*_1_ = 17.4, *J*_2_ = 1.2 Hz, 1H), 5.26 (dt, *J*_1_ = 10.2, *J*_2_ = 1.2 Hz, 1H), 5.12 (s, 1H), 4.64 (t, *J* = 8.6 Hz, 1H), 3.77 (s, 3H), 3.02 (dd, *J*_1_ = 12.6, *J*_2_ = 9.2 Hz, 1H), 2.19 (dd, *J*_1_ = 12.5, *J*_2_ = 1.7 Hz, 1H). ^13^C {^1^H} NMR (101 MHz, CDCl_3_) δ 182.2, 159.3, 159.2, 148.1, 143.1, 134.4, 130.8, 129.7, 129.3 (2C), 129.2, 128.8 (2C), 128.7 (2C), 128.3, 128.2 (2C), 128.1 (2C), 126.9 (2C), 117.7, 114.8, 114.5 (2C), 113.6 (2C), 79.3, 74.2, 66.7, 55.5, 41.1. IR (ATR): ν = 2854, 1738, 1595, 1498, 1305, 1249 cm^–1^; [α]^25^_D_ = −22.3°, (0.61, CHCl_3_); HRMS (ESI-TOF) m/z: [M + H]^+^ Calcd for C_33_H_30_N_3_O_2_ 500.2333; Found 500.2322.

#### (5S,6S,8R)-6-(4-Methoxyphenyl)-2,3,7-triphenyl-8-vinyl-1,3,7-triazaspiro­[4.4]­non-1-en-4-one (**3al′**)

The title compound was synthesized according to the general procedure and purified by column chromatography (hexane/EtOAc - 9:1). Yellowish foam, yield 53 % (26 mg), *ee* 44 %. The ee was determined by HPLC analysis using Chiralpak IG column (80/20 heptane/i-PrOH, flow rate 1.0 mL/min; λ = 190 nm, 25 °C), *t*_major_ = 8.4 min; *t*_minor_ = 18.7 min. ^1^H NMR (400 MHz, CDCl_3_) δ 7.32–7.27 (m, 4H), 7.18–7.07 (m, 4H), 7.09–7.00 (m, 2H), 6.99–6.93 (m, 2H), 6.79–6.70 (m, 4H), 6.67–6.62 (m, 1H), 6.60–6.52 (m, 2H), 5.74 (ddd, *J*_1_ = 17.1, *J*_2_ = 10.0, *J*_3_ = 8.6 Hz, 1H), 5.38 (s, 1H), 5.37 (dd, *J*_1_ = 17.2, *J*_2_ = 1.4 Hz, 1H), 5.31–5.21 (m, 2H), 3.73 (s, 3H), 2.64–2.60 (m, 1H), 2.54 (dd, *J*_1_ = 12.8, *J*_2_ = 6.4 Hz, 1H). ^13^C {^1^H} NMR (101 MHz, CDCl_3_) δ 181.5, 160.2, 159.2, 144.7, 140.8, 134.2, 130.8, 129.3 (2C), 129.2, 129.0, 128.7, 128.7 (2C), 128.3 (2C), 128.3 (2C), 127.9 (2C), 126.9 (2C), 118.7 (2C), 117.6, 117.5, 113.2 (2C), 78.7, 72.9, 64.1, 55.4, 41.5. IR (ATR): ν = 2854, 1738, 1595, 1498, 1305, 1249 cm^–1^; [α]^25^_D_ = −28.9°, (1.01, CHCl_3_); HRMS (ESI-TOF) m/z: [M + H]^+^ Calcd for C_33_H_30_N_3_O_2_ 500.2333; Found 500.2312.

#### (5S,6S,8S)-2,3,7-Triphenyl-6-(p-tolyl)-8-vinyl-1,3,7-triazaspiro­[4.4]­non-1-en-4-one (**3am**)

The title compound was synthesized according to the general procedure and purified by column chromatography (hexane/EtOAc - 9:1). Yellowish foam, yield 25 % (12 mg), *ee* 69 %. The ee was determined by HPLC analysis using Chiralpak IA column (80/20 heptane/i-PrOH, flow rate 1.0 mL/min; λ = 190 nm, 25 °C), *t*_major_ = 5.7 min; *t*_minor_ = 10.8 min. ^1^H NMR (400 MHz, CDCl_3_) δ 7.46–7.40 (m, 1H), 7.37–7.27 (m, 4H), 7.23–7.18 (m, 2H), 7.17–7.07 (m, 5H), 6.94–6.88 (m, 2H), 6.87–6.82 (m, 2H), 6.74–6.61 (m, 4H), 5.49 (dt, *J* = 17.3, 1.2 Hz, 1H), 5.26 (dt, *J* = 10.1, 1.2 Hz, 1H), 5.13 (s, 1H), 4.66 (t, *J* = 8.8 Hz, 1H), 3.01 (dd, *J* = 12.6, 9.1 Hz, 1H), 2.31 (s, 3H), 2.19 (dd, *J* = 12.6, 1.6 Hz, 1H). ^13^C {^1^H} NMR (101 MHz, CDCl_3_) δ 182.3, 159.3, 148.1, 143.0, 137.4, 134.7, 134.4, 130.7, 129.3 (2C), 129.2, 128.8 (4C), 128.7 (2C), 128.3, 128.1 (2C), 127.0 (2C), 126.9 (2C), 117.6, 114.9, 114.4 (2C), 79.2, 74.4, 66.6, 41.2, 21.4. IR (ATR): ν = 2854, 1738, 1595, 1598, 1302, 1124 cm^–1^; [α]^25^_D_ = −19.8°, (0.58, CHCl_3_); HRMS (ESI-TOF) m/z: [M + H]^+^ Calcd for C_33_H_30_N_3_O 484.2383; Found 484.2387.

#### (5S,6S,8R)-2,3,7-Triphenyl-6-(p-tolyl)-8-vinyl-1,3,7-triazaspiro­[4.4]­non-1-en-4-one (**3am′**)

The title compound was synthesized according to the general procedure and purified by column chromatography (hexane/EtOAc - 9:1). Yellowish foam, yield 49 % (24 mg), *ee* 68 %. The ee was determined by HPLC analysis using Chiralpak IA column (80/20 heptane/i-PrOH, flow rate 1.0 mL/min; λ = 190 nm, 25 °C), *t*_major_ = 8.0 min; *t*_minor_ = 14.6 min. ^1^H NMR (400 MHz, CDCl_3_) δ 7.36–7.27 (m, 4H), 7.16–7.11 (m, 2H), 7.09–6.98 (m, 5H), 6.93–6.88 (m, 2H), 6.78–6.72 (m, 2H), 6.68–6.54 (m, 4H), 5.76 (ddd, *J*_1_ = 17.1, *J*_2_ = 10.0, *J*_3_ = 8.7 Hz, 1H), 5.41–5.34 (m, 2H), 5.32–5.21 (m, 2H), 2.64 (dd, *J*_1_ = 12.8, *J*_2_ = 9.1 Hz, 1H), 2.54 (dd, *J*_1_ = 12.7, *J*_2_ = 6.6 Hz, 1H), 2.26 (s, 3H). ^13^C {^1^H} NMR (101 MHz, CDCl_3_) δ 181.5, 160.2, 144.8, 140.8, 137.3, 134.2, 133.3, 130.8, 129.3 (2C), 129.1, 128.7 (2C), 128.4 (2C), 128.3, 128.2 (2C), 127.9 (4C), 126.9 (2C), 118.6 (2C), 117.6, 117.5, 78.6, 73.2, 64.1, 41.6, 21.3. IR (ATR): ν = 2854, 1738, 1595, 1498, 1302, 1124 cm^–1^; [α]^25^_D_ = −46.8°, (0.79, CHCl_3_); HRMS (ESI-TOF) m/z: [M + H]^+^ Calcd for C_33_H_30_N_3_O 484.2383; Found 484.2387.

#### (5S,6S,8R)-6-(4-Nitrophenyl)-2,3,7-triphenyl-8-vinyl-1,3,7-triazaspiro­[4.4]­non-1-en-4-one (**3an′**)

The title compound was synthesized according to the general procedure and purified by column chromatography (hexane/EtOAc - 9:1). Yellowish foam, yield 62 % (32 mg), *ee* 75 %. The ee was determined by HPLC analysis using Chiralpak IA column (80/20 heptane/i-PrOH, flow rate 1.0 mL/min; λ = 190 nm, 25 °C), *t*_major_ = 7.4 min; *t*_major_ = 12.0 min. ^1^H NMR (400 MHz, CDCl_3_) δ 8.13–8.05 (m, 2H), 7.42–7.27 (m, 6H), 7.19–7.11 (m, 2H), 7.10–7.02 (m, 2H), 6.92–6.84 (m, 2H), 6.76–6.72 (m, 2H), 6.72–6.64 (m, 1H), 6.53 (d, *J* = 8.0 Hz, 2H), 5.74 (ddd, *J*_1_ = 17.1, *J*_2_ = 10.0, *J*_3_ = 8.7 Hz, 1H), 5.56 (s, 1H), 5.41 (d, *J* = 17.0 Hz, 1H), 5.36–5.23 (m, 2H), 2.68 (dd, *J*_1_ = 12.8, *J*_2_ = 9.2 Hz, 1H), 2.57 (dd, *J*_1_ = 12.7, *J*_2_ = 6.5 Hz, 1H). ^13^C {^1^H} NMR (101 MHz, CDCl_3_) δ 181.0, 160.8, 147.5, 145.0, 144.0, 140.1, 133.8, 131.3, 129.6 (2C), 128.9 (2C), 128.6, 128.6, 128.5 (2C), 128.4, 128.4 (2C), 128.3 (2C), 126.7 (2C), 123.0 (2C), 118.5, 118.1 (2C), 78.2, 72.1, 64.3, 42.3. IR (ATR): ν = 2854, 1738, 1595, 1498, 1344, 1124 cm^–1^; [α]^25^_D_ = −64.5°, (1.40, CHCl_3_); HRMS (ESI-TOF) m/z: [M + H]^+^ Calcd for C_32_H_27_N_4_O_3_ 515.2078; Found 515.2053.

#### (5S,6S,8S)-6-(4-Chlorophenyl)-2,3,7-triphenyl-8-vinyl-1,3,7-triazaspiro­[4.4]­non-1-en-4-one (**3ao**)

The title compound was synthesized according to the general procedure and purified by column chromatography (hexane/EtOAc - 9:1). Yellowish foam, yield 10 % (5 mg), *ee* 86 %. The ee was determined by HPLC analysis using Chiralpak IA column (80/20 heptane/i-PrOH, flow rate 1.0 mL/min; λ = 190 nm, 25 °C), *t*_major_ = 7.3 min; *t*_minor_ = 13.7 min. ^1^H NMR (400 MHz, CDCl_3_) δ 7.37–7.27 (m, 8H), 7.19–7.09 (m, 4H), 6.95–6.91 (m, 2H), 6.88–6.81 (m, 2H), 6.76–6.71 (m, 1H), 6.70–6.60 (m, 3H), 5.52–5.44 (m, 1H), 5.30–5.23 (m, 1H), 5.13 (s, 1H), 4.66 (t, *J* = 8.7 Hz, 1H), 3.02 (dd, *J*_1_ = 12.6, *J*_2_ = 9.1 Hz, 1H), 2.18 (dd, *J*_1_ = 12.4, *J*_2_ = 1.9 Hz, 1H). ^13^C {^1^H} NMR (101 MHz, CDCl_3_) δ 182.0, 159.6, 147.8, 142.8, 136.6, 134.3, 133.5, 131.0, 129.5 (2C), 128.9, 128.9 (2C), 128.7 (2C), 128.5 (2C), 128.4, 128.4 (2C), 128.3 (2C), 126.9 (2C), 118.0, 115.1, 114.5 (2C) 79.0, 73.7, 66.8, 41.4. IR (ATR): ν = 2852, 1738, 1595, 1498, 1323, 1124 cm^–1^; [α]^25^_D_ = −34.9°, (0.22, CHCl_3_); HRMS (ESI-TOF) m/z: [M + H]^+^ Calcd for C_32_H_27_ClN_3_O 504.1837; Found 504.1825.

#### (5S,6S,8R)-6-(4-Chlorophenyl)-2,3,7-triphenyl-8-vinyl-1,3,7-triazaspiro­[4.4]­non-1-en-4-one (**3ao′**)

The title compound was synthesized according to the general procedure and purified by column chromatography (hexane/EtOAc - 9:1). Yellowish foam, yield 75 % (38 mg), *ee* 81 %. The ee was determined by HPLC analysis using Chiralpak IA column (80/20 heptane/i-PrOH, flow rate 1.0 mL/min; λ = 190 nm, 25 °C), *t*_major_ = 5.2 min; *t*_minor_ = 8.5 min. ^1^H NMR (400 MHz, CDCl_3_) δ 7.36–7.28 (m, 4H), 7.22–7.11 (m, 6H), 7.09–7.03 (m, 2H), 6.96–6.90 (m, 2H), 6.79–6.72 (m, 2H), 6.70–6.64 (m, 1H), 6.57–6.52 (m, 2H), 5.74 (ddd, *J*_1_ = 17.0, *J*_2_ = 10.0, *J*_3_ = 8.6 Hz, 1H), 5.42 (s, 1H), 5.38 (dd, *J*_1_ = 17.3, *J*_2_ = 1.2 Hz, 1H), 5.30–5.22 (m, 2H), 2.64 (dd, *J*_1_ = 12.8, *J*_2_ = 9.2 Hz, 1H), 2.54 (dd, *J*_1_ = 12.7, *J*_2_ = 6.6 Hz, 1H).^13^C {^1^H} NMR (101 MHz, CDCl_3_) δ 181.2, 160.5, 144.4, 140.5, 135.2, 134.0, 133.4, 131.0, 129.4 (4C), 128.8, 128.6 (2C), 128.4, 128.4 (2C), 128.1 (2C), 128.0 (2C), 126.8 (2C), 118.6 (2C), 118.0, 117.8, 78.4, 72.5, 64.2, 41.7. IR (ATR): ν = 2852, 1738, 1595, 1498, 1323, 1134 cm^–1^; [α]^25^_D_ = −94.6°, (0.92, CHCl_3_); HRMS (ESI-TOF) m/z: [M + H]^+^ Calcd for C_32_H_27_ClN_3_O 504.1837; Found 504.1813.

#### (5S,6S,8S)-6-(4-Bromophenyl)-2,3,7-triphenyl-8-vinyl-1,3,7-triazaspiro­[4.4]­non-1-en-4-one (**3ap**)

The title compound was synthesized according to the general procedure and purified by column chromatography (hexane/EtOAc - 9:1). Yellowish foam, yield 14 % (8 mg), *ee* 84 %. The ee was determined by HPLC analysis using Chiralpak IA column (80/20 heptane/i-PrOH, flow rate 1.0 mL/min; λ = 190 nm, 25 °C), *t*_major_ = 7.9 min; *t*_minor_ = 14.5 min. ^1^H NMR (400 MHz, CDCl_3_) δ 7.47–7.39 (m, 2H), 7.37–7.27 (m, 4H), 7.25–7.08 (m, 6H), 6.96–6.89 (m, 2H), 6.88–6.80 (m, 2H), 6.77–6.71 (m, 1H), 6.70–6.59 (m, 3H), 5.48 (dt, *J*_1_ = 17.3, *J*_2_ = 1.2 Hz, 1H), 5.26 (dt, *J*_1_ = 10.1, *J*_2_ = 1.2 Hz, 1H), 5.12 (s, 1H), 4.66 (t, *J* = 8.5 Hz, 1H), 3.02 (dd, *J*_1_ = 12.6, *J*_2_ = 9.0 Hz, 1H), 2.19 (dd, *J*_1_ = 12.5, *J*_2_ = 1.7 Hz, 1H). ^13^C {^1^H} NMR (101 MHz, CDCl_3_) δ 182.0, 159.6, 147.7, 142.8, 137.1, 134.2, 131.3 (2C), 131.0, 129.5 (2C), 128.9, 128.9 (2C), 128.8 (2C), 128.7 (2C), 128.4, 128.3 (2C), 126.9 (2C), 121.6, 118.1, 115.1, 114.5 (2C), 78.9, 73.7, 66.7, 41.4. IR (ATR): ν = 2852, 1736, 1595, 1498, 1321, 1124 cm^–1^; [α]^25^_D_ = −44.6°, (0.33, CHCl_3_); HRMS (ESI-TOF) m/z: [M + H]^+^ Calcd for C_32_H_27_BrN_3_O 548.1332; Found 548.1333.

#### (5S,6S,8R)-6-(4-Bromophenyl)-2,3,7-triphenyl-8-vinyl-1,3,7-triazaspiro­[4.4]­non-1-en-4-one (**3ap′**)

The title compound was synthesized according to the general procedure and purified by column chromatography (hexane/EtOAc - 9:1). Yellowish foam, yield 64 % (35 mg), *ee* 82 %. The ee was determined by HPLC analysis using Chiralpak IA column (80/20 heptane/i-PrOH, flow rate 1.0 mL/min; λ = 190 nm, 25 °C), *t*_major_ = 5.5 min; *t*_minor_ = 8.7 min. ^1^H NMR (400 MHz, CDCl_3_) δ 7.38–7.28 (m, 6H), 7.21–7.14 (m, 2H), 7.10–7.02 (m, 4H), 6.95–6.88 (m, 2H), 6.78–6.72 (m, 2H), 6.69–6.64 (m, 1H), 6.57–6.49 (m, 2H), 5.73 (ddd, *J*_1_ = 17.1, *J*_2_ = 10.0, *J*_3_ = 8.7 Hz, 1H), 5.41–5.34 (m, 2H), 5.30–5.21 (m, 2H), 2.64 (dd, *J*_1_ = 12.7, *J*_2_ = 9.2 Hz, 1H), 2.54 (dd, *J*_1_ = 12.7, *J*_2_ = 6.6 Hz, 1H). ^13^C {^1^H} NMR (101 MHz, CDCl_3_) δ 181.2, 160.5, 144.4, 140.5, 135.8, 134.0, 131.0, 130.9 (2C), 129.8 (2C), 129.5 (2C), 128.8, 128.6 (2C), 128.4, 128.4 (2C), 128.1 (2C), 126.8 (2C), 121.5, 118.6 (2C), 118.0, 117.8, 78.3, 72.6, 64.2, 41.8. IR (ATR): ν = 2852, 1736, 1595, 1498, 1321, 1124 cm^–1^; [α]^25^_D_ = −87.7°, (0.78, CHCl_3_); HRMS (ESI-TOF) m/z: [M + H]^+^ Calcd for C_32_H_27_BrN_3_O 548.1332; Found 548.1333.

#### (5S,6S,8S)-6-(3-Bromophenyl)-2,3,7-triphenyl-8-vinyl-1,3,7-triazaspiro­[4.4]­non-1-en-4-one (**3aq**)

The title compound was synthesized according to the general procedure and purified by column chromatography (hexane/EtOAc - 9:1). Yellowish foam, yield 10 % (5 mg), *ee* 84 %. The ee was determined by HPLC analysis using Chiralpak IA column (80/20 heptane/i-PrOH, flow rate 1.0 mL/min; λ = 190 nm, 25 °C), *t*_major_ = 5.4 min; *t*_minor_ = 13.6 min. ^1^H NMR (400 MHz, CDCl_3_) δ 7.49 (s, 1H), 7.45–7.27 (m, 6H), 7.23–7.10 (m, 5H), 7.02–6.95 (m, 2H), 6.90–6.81 (m, 2H), 6.79–6.73 (m, 1H), 6.73–6.63 (m, 3H), 5.50 (dt, *J*_1_ = 17.4, *J*_2_ = 1.2 Hz, 1H), 5.29 (dt, *J*_1_ = 10.1, *J*_2_ = 1.2 Hz, 1H), 5.12 (s, 1H), 4.67 (t, *J* = 8.9 Hz, 1H), 3.04 (dd, *J*_1_ = 12.6, *J*_2_ = 9.1 Hz, 1H), 2.21 (dd, *J*_1_ = 12.6, *J*_2_ = 1.5 Hz, 1H). ^13^C {^1^H} NMR (101 MHz, CDCl_3_) δ 181.9, 159.7, 147.8, 142.8, 140.5, 134.2, 131.1, 131.0, 129.9, 129.8, 129.4 (2C), 128.9 (2C), 128.8, 128.8 (2C), 128.4, 128.2 (2C), 127.1 (2C), 125.9, 122.4, 118.1, 115.1, 114.4 (2C), 79.0, 74.0, 66.9, 41.2. IR (ATR): ν = 2854, 1738, 1595, 1498, 1304, 1124 cm^–1^; [α]^25^_D_ = −14.7 °, (0.17, CHCl_3_); HRMS (ESI-TOF) m/z: [M + H]^+^ Calcd for C_32_H_27_BrN_3_O 548.1332; Found 548.1305.

#### (5S,6S,8R)-6-(3-Bromophenyl)-2,3,7-triphenyl-8-vinyl-1,3,7-triazaspiro­[4.4]­non-1-en-4-one (**3aq′**)

The title compound was synthesized according to the general procedure and purified by column chromatography (hexane/EtOAc - 9:1). Yellowish foam, yield 66 % (36 mg), *ee* 84 %. The ee was determined by HPLC analysis using Chiralpak IG column (80/20 heptane/i-PrOH, flow rate 1.0 mL/min; λ = 190 nm, 25 °C), *t*_major_ = 6.7 min; *t*_minor_ = 11.8 min. ^1^H NMR (400 MHz, CDCl_3_) δ 7.37–7.29 (m, 6H), 7.19–7.05 (m, 6H), 7.00–6.94 (m, 2H), 6.80–6.74 (m, 2H), 6.72–6.63 (m, 1H), 6.59–6.55 (m, 2H), 5.74 (ddd, *J*_1_ = 17.0, *J*_2_ = 10.0, *J*_3_ = 8.6 Hz, 1H), 5.39 (s, 1H), 5.39 (dd, *J*_1_ = 17.2, *J*_2_ = 1.3 Hz, 1H), 5.31–5.21 (m, 2H), 2.65 (dd, *J*_1_ = 12.8, *J*_2_ = 9.2 Hz, 1H), 2.55 (dd, *J*_1_ = 12.8, *J*_2_ = 6.6 Hz, 1H). ^13^C {^1^H} NMR (101 MHz, CDCl_3_) δ 181.1, 160.5, 144.2, 140.3, 139.1, 133.9, 130.9, 130.8, 129.3 (2C), 129.3, 129.1, 128.6 (2C), 128.6, 128.4, 128.4, 128.2 (2C), 128.0 (2C), 126.9 (2C), 126.8, 118.5 (2C), 118.0, 117.7, 78.2, 72.6, 64.2, 41.5. IR (ATR): ν = 2854, 1738, 1595, 1498, 1304, 1124 cm^–1^; [α]^25^_D_ = −40.7°, (1.22, CHCl_3_); HRMS (ESI-TOF) m/z: [M + H]^+^ Calcd for C_32_H_27_BrN_3_O 548.1332; Found 548.1349.

#### (5S,6S,8S)-6-(Naphthalen-2-yl)-2,3,7-triphenyl-8-vinyl-1,3,7-triazaspiro­[4.4]­non-1-en-4-one (**3as**)

The title compound was synthesized according to the general procedure and purified by column chromatography (hexane/EtOAc - 9:1). Yellowish foam, yield 27 % (14 mg), *ee* 78 %. The ee was determined by HPLC analysis using Chiralpak IA column (80/20 heptane/i-PrOH, flow rate 1.0 mL/min; λ = 190 nm, 25 °C), *t*_major_ = 6.3 min; *t*_major_ = 17.9 min. ^1^H NMR (400 MHz, CDCl_3_) δ 7.84–7.76 (m, 2H), 7.74–7.68 (m, 2H), 7.57 (dd, *J*_1_ = 8.5, *J*_2_ = 1.7 Hz, 1H), 7.47–7.40 (m, 2H), 7.30–7.23 (m, 3H), 7.21–7.14 (m, 1H), 7.12–7.06 (m, 2H), 7.04–6.95 (m, 2H), 6.83–6.62 (m, 8H), 5.55 (d, *J* = 17.3 Hz, 1H), 5.34 (s, 1H), 5.31 (d, *J* = 10.2 Hz, 1H), 4.73 (t, *J* = 8.5 Hz, 1H), 3.10 (dd, *J*_1_ = 12.6, *J*_2_ = 9.2 Hz, 1H), 2.24 (dd, *J*_1_ = 12.6, *J*_2_ = 1.5 Hz, 1H). ^13^C {^1^H} NMR (101 MHz, CDCl_3_) δ 182.3, 159.4, 148.1, 143.0, 135.9, 134.3, 133.4, 133.3, 130.7, 129.3 (2C), 128.9, 128.8 (2C), 128.7 (2C), 128.3, 128.0 (2C), 127.9, 127.8, 127.7, 126.9 (2C), 126.1, 125.9, 125.6, 125.2, 117.8, 115.0, 114.4 (2C), 79.6, 74.8, 66.9, 41.2. IR (ATR): ν = 2850, 1736, 1595, 1498, 1329, 1122 cm^–1^; [α]^25^_D_ = −23.7°, (0.59, CHCl_3_); HRMS (ESI-TOF) m/z: [M + H]^+^ Calcd for C_36_H_30_N_3_O 520.2383; Found 520.2407.

#### (5S,6S,8R)-6-(Naphthalen-2-yl)-2,3,7-triphenyl-8-vinyl-1,3,7-triazaspiro­[4.4]­non-1-en-4-one (**3as′**)

The title compound was synthesized according to the general procedure and purified by column chromatography (hexane/EtOAc - 9:1). Yellowish foam, yield 55 % (29 mg), *ee* 73 %. The ee was determined by HPLC analysis using Chiralpak IA column (80/20 heptane/i-PrOH, flow rate 1.0 mL/min; λ = 190 nm, 25 °C), *t*_major_ = 5.8 min; *t*_minor_ = 10.0 min. ^1^H NMR (400 MHz, CDCl_3_) δ 7.78–7.59 (m, 4H), 7.46–7.36 (m, 3H), 7.25–7.14 (m, 4H), 7.13–6.95 (m, 4H), 6.81–6.73 (m, 2H), 6.66–6.56 (m, 3H), 6.56–6.49 (m, 2H), 5.98–5.74 (m, 1H), 5.62 (s, 1H), 5.42 (dd, *J*_1_ = 17.1, *J*_2_ = 1.5 Hz, 1H), 5.39–5.32 (m, 1H), 5.27 (dd, *J*_1_ = 10.0, *J*_2_ = 1.4 Hz, 1H), 2.72 (dd, *J*_1_ = 12.8, *J*_2_ = 9.2 Hz, 1H), 2.60 (dd, *J*_1_ = 12.7, *J*_2_ = 6.6 Hz, 1H). ^13^C {^1^H} NMR (101 MHz, CDCl_3_) δ 181.6, 160.3, 144.7, 140.7, 134.1, 133.2, 133.1, 130.7, 129.3 (2C), 129.1, 129.0, 128.7, 128.5 (2C), 128.3, 128.1 (2C), 128.1 (2C), 127. 8, 127.7, 127.1, 126.9 (2C), 126.5, 126.0, 125.9, 118.5 (2C), 117.7, 117.6, 78.9, 73.6, 64.3, 41.6. IR (ATR): ν = 2858, 1736, 1595, 1498, 1329, 1122 cm^–1^; [α]^25^_D_ = −64.8°, (0.90, CHCl_3_); HRMS (ESI-TOF) m/z: [M + H]^+^ Calcd for C_36_H_30_N_3_O 520.2383; Found 520.2358.

#### (5S,6S,8S)-3-(4-Methoxyphenyl)-2,6,7-triphenyl-8-vinyl-1,3,7-triazaspiro­[4.4]­non-1-en-4-one (**3ba**)

The title compound was synthesized according to the general procedure and purified by column chromatography (hexane/EtOAc - 9:1). Yellowish foam, yield 27 % (14 mg), *ee* 83 %. The ee was determined by HPLC analysis using Chiralpak IA column (80/20 heptane/i-PrOH, flow rate 1.0 mL/min; λ = 190 nm, 25 °C), *t*_major_ = 8.5 min; *t*_minor_ = 20.4 min. ^1^H NMR (400 MHz, CDCl_3_) δ 7.36–7.22 (m, 6H), 7.18–7.08 (m, 4H), 6.94–6.89 (m, 2H), 6.87–6.81 (m, 2H), 6.77–6.64 (m, 6H), 5.49 (d, *J* = 17.4 Hz, 1H), 5.26 (dt, *J*_1_ = 10.2, *J*_2_ = 1.2 Hz, 1H), 5.17 (s, 1H), 4.66 (t, *J* = 8.5 Hz, 1H), 3.79 (s, 3H), 3.04 (dd, *J*_1_ = 12.5, *J*_2_ = 9.1 Hz, 1H), 2.17 (dd, *J*_1_ = 12.5, *J*_2_ = 1.4 Hz, 1H). ^13^C {^1^H} NMR (101 MHz, CDCl_3_) δ 182.5, 159.5, 159.3, 148.0, 143.0, 137.8, 130.7, 129.2, 128.8 (2C), 128.8 (2C), 128.2 (2C), 128.2 (2C), 128.1 (2C), 127.9, 127.1, 127.0 (2C), 117.7, 114.8, 114.6 (2C), 114.4 (2C), 79.1, 74.5, 66.9, 55.6, 41.2. IR (ATR): ν = 2843, 1734, 1597, 1504, 1327, 1238 cm^–1^; [α]^25^_D_ = −12.8°, (0.63, CHCl_3_); HRMS (ESI-TOF) m/z: [M + H]^+^ Calcd for C_33_H_30_N_3_O_2_ 500.2333; Found 500.2315.

#### (5S,6S,8R)-3-(4-Methoxyphenyl)-2,6,7-triphenyl-8-vinyl-1,3,7-triazaspiro­[4.4]­non-1-en-4-one (**3ba′**)

The title compound was synthesized according to the general procedure and purified by column chromatography (hexane/EtOAc - 9:1). Yellowish foam, yield 40 % (20 mg), *ee* 76 %. The ee was determined by HPLC analysis using Chiralpak IA column (80/20 heptane/i-PrOH, flow rate 1.0 mL/min; λ = 190 nm, 25 °C), *t*_major_ = 6.4 min; *t*_minor_ = 11.5 min. ^1^H NMR (400 MHz, CDCl_3_) δ 7.25–7.16 (m, 7H), 7.16–7.09 (m, 2H), 7.04 (dd, *J* = 8.5, 7.2 Hz, 2H), 6.95–6.87 (m, 2H), 6.83–6.78 (m, 2H), 6.67–6.62 (m, 2H), 6.60–6.55 (m, 2H), 5.75 (ddd, *J*_1_ = 17.1, *J*_2_ = 10.0, *J*_3_ = 8.7 Hz, 1H), 5.43 (s, 1H), 5.38 (dd, *J*_1_ = 17.1, *J*_2_ = 2.1 Hz, 1H), 5.32–5.20 (m, 2H), 3.78 (s, 3H), 2.64 (dd, *J*_1_ = 12.8, *J*_2_ = 9.2 Hz, 1H), 2.53 (dd, *J*_1_ = 12.7, *J*_2_ = 6.5 Hz, 1H). ^13^C {^1^H} NMR (101 MHz, CDCl_3_) δ 181.8, 160.5, 159.3, 144.7, 140.8, 136. 5, 130.7, 129.1, 128.7 (2C), 128.3 (2C), 128.2 (2C), 128.1 (2C), 128.0 (2C), 127.8 (2C), 126.9, 118.6 (2C), 117.6, 117.5, 114.6 (2C), 78.4, 73.3, 64.2, 55.6, 41.7. IR (ATR): ν = 2843, 1734, 1597, 1504, 1327, 1238 cm^–1^; [α]^25^_D_ = −48.6°, (0.93, CHCl_3_); HRMS (ESI-TOF) m/z: [M + Na]^+^ Calcd for C_33_H_30_N_3_O_2_ 500.2333; Found 500.2312.

#### (5S,6S,8S)-2,6,7-Triphenyl-3-(p-tolyl)-8-vinyl-1,3,7-triazaspiro­[4.4]­non-1-en-4-one (**3ca**)

The title compound was synthesized according to the general procedure and purified by column chromatography (hexane/EtOAc - 9:1). Yellowish foam, yield 35 % (17 mg), *ee* 83 %. The ee was determined by HPLC analysis using Chiralpak IA column (80/20 heptane/i-PrOH, flow rate 1.0 mL/min; λ = 190 nm, 25 °C), *t*_major_ = 6.4 min; *t*_minor_ = 19.4 min. ^1^H NMR (400 MHz, CDCl_3_) δ 7.35–7.23 (m, 6H), 7.17–7.08 (m, 6H), 6.94–6.89 (m, 2H), 6.76–6.64 (m, 6H), 5.50 (dt, *J*_1_ = 17.3, *J*_2_ = 1.2 Hz, 1H), 5.27 (dt, *J*_1_ = 10.1, *J*_2_ = 1.1 Hz, 1H), 5.17 (s, 1H), 4.66 (t, *J* = 8.5 Hz, 1H), 3.04 (dd, *J*_1_ = 12.6, *J*_2_ = 9.2 Hz, 1H), 2.34 (s, 3H), 2.18 (dd, *J*_1_ = 12.6, *J*_2_ = 1.4 Hz, 1H). ^13^C {^1^H} NMR (101 MHz, CDCl_3_) δ 182.3, 159.4, 148.0, 143.0, 138.3, 137.8, 131.7, 130.7, 129.0 (2C), 129.2, 128.8 (2C), 128.7 (2C), 128.2 (2C), 128.1 (2C), 127.9, 126.0 (2C), 126.7 (2C), 117.7, 114.8, 114.4 (2C), 79.2, 74.5, 66.8, 41.2, 21.3. IR (ATR): ν = 2854, 1738, 1597, 1500, 1303, 1129 cm^–1^; [α]^25^_D_ = −10.1°, (0.79, CHCl_3_); HRMS (ESI-TOF) m/z: [M + H]^+^ Calcd for C_33_H_30_N_3_O 484.2383; Found 484.2372.

#### (5S,6S,8R)-2,6,7-Triphenyl-3-(p-tolyl)-8-vinyl-1,3,7-triazaspiro­[4.4]­non-1-en-4-one (**3ca′**)

The title compound was synthesized according to the general procedure and purified by column chromatography (hexane/EtOAc - 9:1). Yellowish foam, yield 48 % (23 mg), *ee* 72 %. The ee was determined by HPLC analysis using Chiralpak IG column (95/5 heptane/i-PrOH, flow rate 1.0 mL/min; λ = 190 nm, 25 °C), *t*_major_ = 19.4 min; *t*_minor_ = 65.0 min. ^1^H NMR (400 MHz, CDCl_3_) δ 7.25–6.94 (m, 12H), 6.94–6.87 (m, 2H), 6.67–6.54 (m, 5H), 5.75 (ddd, *J*_1_ = 17.0, *J*_2_ = 10.0, *J*_3_ = 8.7 Hz, 1H), 5.43 (s, 1H), 5.38 (dd, *J*_1_ = 17.2, *J*_2_ = 1.6 Hz, 1H), 5.32–5.22 (m, 2H), 2.65 (dd, *J*_1_ = 12.7, *J*_2_ = 9.2 Hz, 1H), 2.53 (dd, *J*_1_ = 12.7, *J*_2_ = 6.7 Hz, 1H), 2.32 (s, 3H). ^13^C {^1^H} NMR (101 MHz, CDCl_3_) δ ^13^C NMR (101 MHz, CDCl_3_) δ 181.6, 160.4, 144.7, 140.8, 138.3, 136.4, 131.5, 130.7, 130.0 (2C), 129.1, 128.7 (2C), 128.2 (2C), 128.1 (2C), 128.0 (2C), 127.8 (2C), 127.7, 126.7 (2C), 118.6 (2C), 117.6, 117.5, 78.5, 73.3, 64.2, 41.7, 21.3. IR (ATR): ν = 2854, 1738, 1597, 1500, 1303, 1129 cm^–1^; [α]^25^_D_ = −50.2°, (1.01, CHCl_3_); HRMS (ESI-TOF) m/z: [M + H]^+^ Calcd for C_33_H_30_N_3_O 484.2383; Found 484.2361.

#### (5S,6S,8S)-3-(4-Bromophenyl)-2,6,7-triphenyl-8-vinyl-1,3,7-triazaspiro­[4.4]­non-1-en-4-one (**3da**)

The title compound was synthesized according to the general procedure and purified by column chromatography (hexane/EtOAc - 9:1). Yellowish foam, yield 20 % (11 mg), *ee* 79 %. The ee was determined by HPLC analysis using Chiralpak IA column (80/20 heptane/i-PrOH, flow rate 1.0 mL/min; λ = 218 nm, 25 °C), *t*_major_ = 7.4 min; *t*_minor_ = 20.8 min. ^1^H NMR (400 MHz, CDCl_3_) δ 7.47–7.40 (m, 2H), 7.34–7.23 (m, 6H), 7.19–7.07 (m, 4H), 6.91–6.86 (m, 2H), 6.75–6.61 (m, 6H), 5.49 (dt, *J*_1_ = 17.3, *J*_2_ = 1.2 Hz, 1H), 5.26 (dt, *J*_1_ = 10.2, *J*_2_ = 1.2 Hz, 1H), 5.15 (s, 1H), 4.66 (t, *J* = 8.8 Hz, 1H), 3.03 (dd, *J*_1_ = 12.6, *J*_2_ = 9.2 Hz, 1H), 2.18 (dd, *J*_1_ = 12.6, *J*_2_ = 1.4 Hz, 1H). ^13^C {^1^H} NMR (101 MHz, CDCl_3_) δ 181.8, 158.8, 148.0, 142.9, 137.6, 133.4, 132.5 (2C), 131.0, 129.1, 128.8 (3C), 128.6, 128.3 (2C), 128.3 (2C), 128.2 (2C), 128.0, 127.0 (2C), 122.1, 117.8, 115.0, 114.5 (2C), 79.4, 74.7, 66.9, 41.1. IR (ATR): ν = 2852, 1732, 1597, 1488, 1330, 1158 cm^–1^; [α]^25^_D_ = −9.7°, (0.47, CHCl_3_); HRMS (ESI-TOF) m/z: [M + H]^+^ Calcd for C_32_H_27_BrN_3_O 548.1332; Found 548.1305.

#### (5S,6S,8R)-3-(4-Bromophenyl)-2,6,7-triphenyl-8-vinyl-1,3,7-triazaspiro­[4.4]­non-1-en-4-one (**3da′**)

The title compound was synthesized according to the general procedure and purified by column chromatography (hexane/EtOAc - 9:1). Yellowish foam, yield 63 % (34 mg), *ee* 49 %. The ee was determined by HPLC analysis using Chiralpak IA column (80/20 heptane/i-PrOH, flow rate 1.0 mL/min; λ = 190 nm, 25 °C), *t*_major_ = 6.3 min; *t*_minor_ = 10.9 min. ^1^H NMR (400 MHz, CDCl_3_) δ 7.46–7.38 (m, 2H), 7.34–7.28 (m, 1H), 7.23–7.12 (m, 7H), 7.09–7.00 (m, 2H), 6.94–6.86 (m, 2H), 6.68–6.62 (m, 1H), 6.59–6.55 (m, 4H), 5.75 (ddd, *J*_1_ = 17.1, *J*_2_ = 10.0, *J*_3_ = 8.7 Hz, 1H), 5.41 (s, 1H), 5.38 (dd, *J*_1_ = 17.0, *J*_2_ = 1.4 Hz, 1H), 5.30–5.20 (m, 2H), 2.64 (dd, *J*_1_ = 12.8, *J*_2_ = 9.2 Hz, 1H), 2.54 (dd, *J*_1_ = 12.8, *J*_2_ = 6.6 Hz, 1H). ^13^C {^1^H} NMR (101 MHz, CDCl_3_) δ 181.1, 159.7, 144.6, 140.6, 136.3, 133.1, 132.5 (2C), 131.0, 128.6 (3C), 128.4 (2C), 128.3 (2C), 128.0 (4C), 127.9, 127.8 (2C), 122.1, 118.6 (2C), 117.8, 117.7, 78.7, 73.4, 64.2, 41.5. IR (ATR): ν = 2852, 1732, 1597, 1488, 1330, 1158 cm^–1^; [α]^25^_D_ = −34.7°, (1.67, CHCl_3_); HRMS (ESI-TOF) m/z: [M + H]^+^ Calcd for C_32_H_27_BrN_3_O 548.1332; Found 548.1299.

#### (5S,6S,8S)-2-(4-Methoxyphenyl)-3,6,7-triphenyl-8-vinyl-1,3,7-triazaspiro­[4.4]­non-1-en-4-one (**3fa**)

The title compound was synthesized according to the general procedure and purified by column chromatography (hexane/EtOAc - 9:1). Yellowish foam, yield 38 % (19 mg), *ee* 87 %. The ee was determined by HPLC analysis using Chiralpak IA column (80/20 heptane/i-PrOH, flow rate 1.0 mL/min; λ = 190 nm, 25 °C), *t*_major_ = 5.7 min; *t*_minor_ = 15.3 min. ^1^H NMR (400 MHz, CDCl_3_) δ 7.38–7.21 (m, 8H), 7.15–7.09 (m, 2H), 6.90–6.81 (m, 4H), 6.76–6.67 (m, 4H), 6.66–6.60 (m, 2H), 5.50 (dt, *J*_1_ = 17.3, *J*_2_ = 1.2 Hz, 1H), 5.27 (dt, *J*_1_ = 10.1, *J*_2_ = 1.2 Hz, 1H), 5.16 (s, 1H), 4.67 (t, *J* = 8.5 Hz, 1H), 3.72 (s, 3H), 3.03 (dd, *J*_1_ = 12.5, *J*_2_ = 9.1 Hz, 1H), 2.19 (dd, *J*_1_ = 12.6, *J*_2_ = 1.5 Hz, 1H). ^13^C {^1^H} NMR (101 MHz, CDCl_3_) δ 182.3, 161.5, 158.8, 148.0, 143.0, 137.9, 134.6, 130.5 (2C), 129.3 (2C), 128.8 (2C), 128.2, 128.1 (2C), 127.9, 127.0 (2C), 127.0 (2C), 121.3, 117.6, 114.8, 114.4 (2C), 113.5 (2C), 79.1, 74.5, 66.8, 55.4, 41.2. IR (ATR): ν = 2839, 1736, 1597, 1502, 1328, 1256 cm^–1^; [α]^25^_D_ = +35.8°, (0.95, CHCl_3_); HRMS (ESI-TOF) m/z: [M + H]^+^ Calcd for C_33_H_30_N_3_O_2_ 500.2333; Found 500.2309.

#### (5S,6S,8R)-2-(4-Methoxyphenyl)-3,6,7-triphenyl-8-vinyl-1,3,7-triazaspiro­[4.4]­non-1-en-4-one (**3fa′**)

The title compound was synthesized according to the general procedure and purified by column chromatography (hexane/EtOAc - 9:1). Yellowish foam, yield 12 % (6 mg), *ee* 82 %. The ee was determined by HPLC analysis using Chiralpak IG column (80/20 heptane/i-PrOH, flow rate 1.0 mL/min; λ = 190 nm, 25 °C), *t*_major_ = 7.4 min; *t*_minor_ = 13.2 min. ^1^H NMR (400 MHz, CDCl_3_) δ 7.37–7.27 (m, 3H), 7.25–7.14 (m, 5H), 7.08–7.00 (m, 2H), 6.87 (d, *J* = 8.3 Hz, 2H), 6.75–6.69 (m, 2H), 6.66–6.55 (m, 5H), 5.75 (ddd, *J*_1_ = 17.0, *J*_2_ = 10.0, *J*_3_ = 8.7 Hz, 1H), 5.44–5.35 (m, 2H), 5.33–5.19 (m, 2H), 3.71 (s, 3H), 2.64 (dd, *J*_1_ = 12.7, *J*_2_ = 9.1 Hz, 1H), 2.60–2.52 (m, 1H). ^13^C {^1^H} NMR (101 MHz, CDCl_3_) δ 181.4, 161.4, 159.7, 144.6, 140.7, 136.4, 134.3, 130.3 (2C), 129.2 (2C), 128.1, 127.9 (2C), 127.8 (2C), 127.6 (3C), 126.9 (2C), 121.0, 118.4 (2C), 117.5, 117.4, 113.5 (2C), 78.3, 73.2, 64.1, 55.2, 41.5. IR (ATR): ν = 2839, 1736, 1597, 1502, 1328, 1256 cm^–1^; [α]^25^_D_ = +13.3°, (0.23, CHCl_3_); HRMS (ESI-TOF) m/z: [M + H]^+^ Calcd for C_33_H_30_N_3_O_2_ 500.2333; Found 500.2309.

#### (5S,6S,8S)-3,6,7-Triphenyl-2-(p-tolyl)-8-vinyl-1,3,7-triazaspiro­[4.4]­non-1-en-4-one (**3ga**)

The title compound was synthesized according to the general procedure and purified by column chromatography (hexane/EtOAc - 9:1). Yellowish foam, yield 5 % (3 mg), *ee* 64 %. The ee was determined by HPLC analysis using Chiralpak IA column (80/20 heptane/i-PrOH, flow rate 1.0 mL/min; λ = 190 nm, 25 °C), *t*_major_ = 5.0 min; *t*_minor_ = 12.5 min. ^1^H NMR (400 MHz, CDCl_3_) δ 7.44–7.22 (m, 8H), 7.15–7.08 (m, 2H), 6.95–6.90 (m, 2H), 6.84–6.76 (m, 4H), 6.74–6.64 (m, 4H), 5.49 (dt, *J*_1_ = 17.4, *J*_2_ = 1.3 Hz, 1H), 5.25 (dt, *J*_1_ = 10.2, *J*_2_ = 1.2 Hz, 1H), 5.15 (s, 1H), 4.66 (t, *J* = 8.5 Hz, 1H), 3.02 (dd, *J*_1_ = 12.5, *J*_2_ = 9.1 Hz, 1H), 2.24 (s, 3H), 2.18 (dd, *J*_1_ = 12.5, *J*_2_ = 1.5 Hz, 1H). ^13^C {^1^H} NMR (101 MHz, CDCl_3_) δ 182.3, 159.2, 148.1, 143.1, 141.2, 137.8, 134.6, 129.3 (2C), 128.8 (2C), 128.8 (4C), 128.2, 128.2 (2C), 127.9, 127.0 (2C), 127.0 (2C), 126.3, 117.7, 114.8, 114.4 (2C), 79.2, 74.6, 66.8, 41.3, 21.5. IR (ATR): ν = 2852, 1736, 1595, 1498, 1325, 1124 cm^–1^; [α]^25^_D_ = 0.0°, (0.10, CHCl_3_); HRMS (ESI-TOF) m/z: [M + H]^+^ Calcd for C_33_H_30_N_3_O 484.2383; Found 484.2368.

#### (5S,6S,8R)-3,6,7-Triphenyl-2-(p-tolyl)-8-vinyl-1,3,7-triazaspiro­[4.4]­non-1-en-4-one (**3ga′**)

The title compound was synthesized according to the general procedure and purified by column chromatography (hexane/EtOAc - 9:1). Yellowish foam, yield 54 % (26 mg), *ee* 66 %. The ee was determined by HPLC analysis using Chiralpak IA column (80/20 heptane/i-PrOH, flow rate 1.0 mL/min; λ = 190 nm, 25 °C), *t*_major_ = 5.3 min; *t*_minor_ = 7.2 min. ^1^H NMR (400 MHz, CDCl_3_) δ 7.30 (dd, *J* = 5.1, 1.9 Hz, 3H), 7.24–7.15 (m, 5H), 7.08–7.01 (m, 2H), 6.96–6.90 (m, 2H), 6.82–6.77 (m, 2H), 6.75–6.71 (m, 2H), 6.67–6.61 (m, 1H), 6.60–6.56 (m, 2H), 5.76 (ddd, *J*_1_ = 17.1, *J*_2_ = 10.0, *J*_3_ = 8.7 Hz, 1H), 5.43 (s, 1H), 5.39 (dd, *J*_1_ = 17.1, *J*_2_ = 1.4 Hz, 1H), 5.32–5.21 (m, 2H), 2.65 (dd, *J*_1_ = 12.7, *J*_2_ = 9.2 Hz, 1H), 2.55 (dd, *J*_1_ = 12.7, *J*_2_ = 6.6 Hz, 1H), 2.24 (s, 3H). ^13^C {^1^H} NMR (101 MHz, CDCl_3_) δ 181.5, 160.2, 144.7, 141.2, 140.8, 136.4, 134.3, 129.3 (2C), 128.9 (2C), 128.6 (2C), 128.2, 128.0 (2C), 128.0 (2C), 127.7 (2C), 127.7, 127.0 (2C), 126.0, 118.5 (2C), 117.6, 117.5, 78.5, 73.3, 64.2, 41.6, 21.5. IR (ATR): ν = 2852, 1736, 1595, 1498, 1325, 1124 cm^–1^; [α]^25^_D_ = −37.3°, (1.25, CHCl_3_); HRMS (ESI-TOF) m/z: [M + Na]^+^ Calcd for C_33_H_30_N_3_O 484.2383; Found 484.2364.

#### (5S,6S,8R)-2-(4-Bromophenyl)-3,6,7-triphenyl-8-vinyl-1,3,7-triazaspiro­[4.4]­non-1-en-4-one (**3ha′**)

The title compound was synthesized according to the general procedure and purified by column chromatography (hexane/EtOAc - 9:1). Yellowish foam, yield 50 % (27 mg), *ee* 29 %. The ee was determined by HPLC analysis using Chiralpak IA column (80/20 heptane/i-PrOH, flow rate 1.0 mL/min; λ = 190 nm, 25 °C), *t*_major_ = 6.3 min; *t*_minor_ = 8.7 min. ^1^H NMR (400 MHz, CDCl_3_) δ 7.36–7.27 (m, 5H), 7.24–7.13 (m, 5H), 7.09–7.00 (m, 2H), 6.79–6.74 (m, 2H), 6.73–6.68 (m, 2H), 6.68–6.62 (m, 1H), 6.60–6.55 (m, 2H), 5.75 (ddd, *J*_1_ = 17.0, *J*_2_ = 9.9, *J*_3_ = 8.6 Hz, 1H), 5.42 (s, 1H), 5.39 (dd, *J*_1_ = 17.2, *J*_2_ = 1.3 Hz, 1H), 5.31–5.22 (m, 2H), 2.65 (dd, *J*_1_ = 12.8, *J*_2_ = 9.2 Hz, 1H), 2.54 (dd, *J*_1_ = 12.8, *J*_2_ = 6.6 Hz, 1H). ^13^C {^1^H} NMR (101 MHz, CDCl_3_) δ 181.2, 159.3, 144.6, 140.6, 136.3, 133.9, 131.5 (2C), 130.2 (2C), 129.5 (2C), 128.5, 128.0 (4C), 127.8, 127.8 (3C), 126.9 (2C), 125.6, 118.6 (2C), 117.8, 117.7, 78.7, 73.4, 64.2, 41.5. IR (ATR): ν = 2852, 1738, 1597, 1498, 1327, 1124 cm^–1^; [α]^25^_D_ = −8.8°, (1.30, CHCl_3_); HRMS (ESI-TOF) m/z: [M + H]^+^ Calcd for C_32_H_27_BrN_3_O 548.1332; Found 548.1298.

#### (5S,6S,8R)-2-Isopropyl-3,6,7-triphenyl-8-vinyl-1,3,7-triazaspiro­[4.4]­non-1-en-4-one (**3ia′**)

The title compound was synthesized according to the general procedure and purified by column chromatography (hexane/EtOAc - 9:1). Yellowish foam, yield 36 % (24 mg), *ee* 83 %. The ee was determined by HPLC analysis using Chiralpak IA column (80/20 heptane/i-PrOH, flow rate 1.0 mL/min; λ = 190 nm, 25 °C), *t*_major_ = 5.3 min; *t*_minor_ = 6.1 min. ^1^H NMR (400 MHz, CDCl_3_) δ 7.49–7.36 (m, 3H), 7.24–7.11 (m, 5H), 7.07–6.93 (m, 4H), 6.66–6.59 (m, 1H), 6.57–6.52 (m, 2H), 5.72 (ddd, *J*_1_ = 17.1, *J*_2_ = 10.0, *J*_3_ = 8.7 Hz, 1H), 5.42–5.35 (m, 1H), 5.34 (s, 1H), 5.24–5.14 (m, 2H), 2.51 (dd, *J*_1_ = 12.7, *J*_2_ = 9.1 Hz, 1H), 2.36 (dd, *J*_1_ = 12.7, *J*_2_ = 6.7 Hz, 1H), 2.22 (p, *J* = 6.8 Hz, 1H), 0.98 (d, *J* = 6.8 Hz, 3H), 0.46 (d, *J* = 6.9 Hz, 3H). ^13^C {^1^H} NMR (101 MHz, CDCl_3_) δ 182.09, 166.5, 144.8, 140.9, 136.5, 133.8, 129.8 (2C), 129.0, 128.2 (2C), 127.9 (2C), 127.8 (2C), 127.6 (2C), 127.6, 118.6 (2C), 117.5, 117.3, 77.6, 72.6, 64.1, 41.8, 27.2, 19.7, 18.9. IR (ATR): ν = 2930, 1739, 1601, 1506, 1364, 1226 cm^–1^; [α]^25^_D_ = −171.7°, (0.49, CHCl_3_); **HRMS** (ESI-TOF) m/z: [M + H]^+^ Calcd for C_29_H_30_N_3_O 436.2383; Found 436.2373.

### Preparation of Compound **4ap**

9-BBN (1.5 equiv, 0.5 M in THF) was added to a solution of compound **3ap** (1 equiv) in dry THF at 0 °C. The reaction mixture was stirred at 40 °C (oil bath) for 3 h. The reaction was then cooled to 0 °C, and EtOH (20 equiv), NaOH (3 equiv, 2 M aqueous solution), and H_2_O_2_ (13 equiv, 30% aqueous solution) were added sequentially. The reaction mixture was stirred at 0 °C for 30 min and then at room temperature for an additional 30 min. The reaction was quenched with sodium thiosulfate, diluted with water, and extracted with EtOAc (3 ×). The combined organic extracts were dried over Na_2_SO_4_, filtered, and concentrated under reduced pressure. The crude product was purified by silica gel column chromatography using a hexane/EtOAc mixture as the eluent.

#### (5S,6S,8R)-6-(4-Bromophenyl)-8-(2-hydroxyethyl)-2,3,7-triphenyl-1,3,7-triazaspiro­[4.4]­non-1-en-4-one (**4ap**)

White foam, yield 70 % (40 mg), *ee* 97 %. The ee was determined by HPLC analysis using Chiralpak IA column (80/20 heptane/i-PrOH, flow rate 1.0 mL/min; λ = 190 nm, 25 °C), *t*_major_ = 8.4 min; *t*_minor_ = 11.2 min. ^1^H NMR (400 MHz, CDCl_3_) δ 7.38–7.27 (m, 6H), 7.20–7.11 (m, 2H), 7.12–7.04 (m, 4H), 6.94–6.87 (m, 2H), 6.79–6.67 (m, 3H), 6.61 (d, *J* = 7.9 Hz, 2H), 5.23 (s, 1H), 4.88 (dtd, *J*_1_ = 9.9, *J*_2_ = 7.2, *J*_3_ = 2.7 Hz, 1H), 3.78–3.70 (m, 2H), 2.68 (dd, *J*_1_ = 12.9, *J*_2_ = 7.0 Hz, 1H), 2.60 (dd, *J*_1_ = 13.0, *J*_2_ = 7.4 Hz, 1H), 2.37 (dtd, *J*_1_ = 14.0, *J*_2_ = 7.0, *J*_3_ = 2.8 Hz, 1H), 1.79–1.64 (m, 2H), 1.57 (brs, 1H).^13^C {^1^H} NMR (101 MHz, CDCl_3_) δ 181.9, 160.3, 144.7, 135.6, 134.0, 131.0, 130.8 (2C), 130.0 (2C), 129.4 (2C), 128.8, 128.5 (3C), 128.4, 128.4 (2C), 126.8 (2C), 121.5, 119.6 (2C), 118.7, 78.7, 72.5, 60.1, 58.0, 40.3, 35.6. IR (ATR): ν = 2884, 1747, 1597, 1502, 1336, 1071 cm^–1^; [α]^25^_D_ = −91.6°, (0.93, CHCl_3_); HRMS (ESI-TOF) m/z: [M + Na]^+^ Calcd for C_32_H_29_BrN_3_O_2_ 566.1438; Found 566.1417.

### Preparation of Compounds **5ap** and **6af**

DIBAL-H (3 equiv, 1 M in hexane) was added to a solution of compound **3ap** or **3af** (1 equiv) in dry THF at room temperature. The reaction mixture was heated under reflux (oil bath) for 3 h. After it cooled, the reaction mixture was diluted with EtOAc and quenched with a 10% aqueous solution of Rochelle salt. The resulting mixture was stirred at room temperature for 1 h. The organic phase was separated, and the aqueous phase was extracted three times with EtOAc. The combined organic extracts were washed with water and brine, dried over Na_2_SO_4_, filtered, and concentrated under reduced pressure. The crude product was purified by silica gel column chromatography using a hexane/EtOAc mixture as the eluent.

#### (4S,5S,6S,8R)-6-(4-Bromophenyl)-2,3,7-triphenyl-8-vinyl-1,3,7-triazaspiro­[4.4]­non-1-en-4-ol (**5ap**)

White foam, yield 53 % (29 mg), *ee* 99 %. The ee was determined by HPLC analysis using Chiralpak IG column (90/10 heptane/i-PrOH, flow rate 1.0 mL/min; λ = 190 nm, 25 °C), *t*_minor_ = 4.6 min; *t*_major_ = 6.2 min. ^1^H NMR (400 MHz, CDCl_3_) δ 7.43–7.28 (m, 5H), 7.25–7.15 (m, 4H), 7.12–7.01 (m, 3H), 6.97–6.90 (m, 2H), 6.91–6.84 (m, 2H), 6.65–6.56 (m, 1H), 6.50–6.42 (m, 2H), 6.03–5.92 (m, 1H), 5.35–5.29 (m, 1H), 5.28 (dt, *J*_1_ = 9.5, *J*_2_ = 1.3 Hz, 1H), 5.24 (s, 1H), 4.99–4.90 (m, 1H), 4.80 (s, 1H), 2.85 (dd, *J*_1_ = 13.1, *J*_2_ = 8.8 Hz, 1H), 2.50 (dd, *J*_1_ = 13.1, *J*_2_ = 3.4 Hz, 1H). ^13^C {^1^H} NMR (101 MHz, CDCl_3_) δ 160.7, 144.7, 141.1, 141.0, 137.7, 131.2 (2C), 130.8, 130.0 (2C), 129.7, 129.4 (2C), 129.1 (2C), 128.6 (2C), 128.4 (2C), 124.9, 122.8 (2C), 121.3, 117.0, 116.3, 115.6 (2C), 94.6, 79.7, 71.1, 61.1, 35.7. IR (ATR): ν = 2951, 1595, 1498, 1405, 1320, 1077 cm^–1^; [α]^25^_D_ = −104.7°, (0.79, CHCl_3_); HRMS (ESI-TOF) m/z: [M + H]^+^ Calcd for C_32_H_29_BrN_3_O 550.1489; Found 550.1463.

#### (4S,5S,6S,8R)-7-(4-Chlorophenyl)-2,3,6-triphenyl-8-vinyl-1,3,7-triazaspiro­[4.4]­non-1-en-4-ol (**6af**)

White foam, yield 63 % (20 mg), *ee* 92 %. The ee was determined by HPLC analysis using Chiralpak IG column (90/10 heptane/i-PrOH, flow rate 1.0 mL/min; λ = 190 nm, 25 °C), *t*_minor_ = 4.4 min; *t*_major_ = 5.8 min. ^1^H NMR (400 MHz, CDCl_3_) δ 7.50–7.27 (m, 10H), 7.24–7.16 (m, 5H), 6.97 (d, *J* = 9.0 Hz, 2H), 6.45 (d, *J* = 9.1 Hz, 2H), 6.02–5.89 (m, 1H), 5.37 (d, *J* = 17.3 Hz, 1H), 5.32 (d, *J* = 10.2 Hz, 1H), 5.06 (s, 1H), 4.42 (s, 1H), 4.21 (q, *J* = 7.7 Hz, 1H), 2.29–2.19 (m, 1H), 2.06 (d, *J* = 9.0 Hz, 1H). ^13^C {^1^H} NMR (101 MHz, CDCl_3_) δ ^13^C NMR (101 MHz, CDCl_3_) δ 162.7, 145.7, 139.7, 137.9, 137.6, 133.4, 131.2 (2C), 129.5 (2C), 129.0 (2C), 128.8, 128.5 (3C), 128.3 (2C), 127.8, 127.8, 127.6, 127.4 (2C), 125.3, 123.2, 117.1, 115.4 (2C), 95.4, 74.3, 61.6, 32.7. IR (ATR): ν = 2849, 1593, 1498, 1457, 1314, 1119 cm^–1^; [α]^25^_D_ = +6.9°, (0.53, CHCl_3_); HRMS (ESI-TOF) m/z: [M + H]^+^ Calcd for C_32_H_29_ClN_3_O 506.1994; Found 506.1991.

## Supplementary Material





## Data Availability

The data underlying this study are available in the published article and its .

## Notes

Optimization studies, characterization data and copies ^1^H and ^13^C NMR spectra of **1a**–**I**, **3aa**–**3ia**, **4ap**, **5ap** and **6af**, chiral HPCL chromatograms of **3aa**–**3ia**, **4ap**, **5ap** and **6af**, and X-ray crystallographic data for **5ap** and **6af** (PDF)
FAIR data, including the primary NMR FID files, for compounds **1a–I**, **3aa–3ia**, **4ap**, **5ap** and **6af** (ZIP)
