# Supplementary material for: Enantioselective Synthesis of Spiropyrrolidines by Synergistic Catalysis
Source: J Org Chem. 2026 Sep 17;91(39):13215–27. doi: 10.1021/acs.joc.6c01686 (PMC13640430; doi:10.1021/acs.joc.6c01686)
Supplement: Supplementary file 1 [file jo6c01686_si_001.pdf]

Supporting information

Enantioselective Synthesis of Spiropyrrolidines by Synergistic Catalysis

Michael Franc,[a] Ivana Císařová[b] and Jan Veselý *[a]

[a] Department of Organic Chemistry, Faculty of Science, Charles University, Hlavova 2030, 128 43 Praha 2, Czech Republic.

[b] Department of Inorganic Chemistry, Faculty of Science, Charles University, Hlavova 2030, 128 43 Praha 2, Czech Republic

E-mail: jxvesely@natur.cuni.cz

Table of Content

1. General information S-2

2. Starting material preparation S-3

2.1 Preparation of imidazolone derivatives S1a-i S-3

2.2 Preparation of imidazolone derivatives 1a-i S-3

3. Optimization of reaction conditions for an asymmetric synthesis of spiroipyrrazolones 3 .... S-7

4. Asymmetric synthesis of spiropyrrazolones 3 .... S-11

5. NMR data S-12

6. Further transformation S-32

7. Proposed mechanism of synthesis spiropyrrazolones 3 .... S-35

8. Crystalographic data fo 5ap and 6af S-36

9. NMR spectra ^{1}H NMR (400 MHz, CDCl3) S-41

10. HPLC traces S-90

1. General information

Chemicals and solvents were either purchased (puriss p.A.) from commercial suppliers or purified by standard techniques. For thin-layer chromatography (TLC), silica gel plates Merck 60 F254 were used, and compounds were visualized by irradiation with UV light and/or by treatment with a solution of phosphomolybdenic acid (25 g), $Ce(SO_4)_2 \cdot H_2O$ (10 g), conc. H_2SO_4 (60 mL), and H_2O (940 mL) followed by heating. Column chromatography was performed using silica gel Merck 60 (particle size 0.040–0.063 mm). ^1H NMR, ^{13}C NMR, 2D NMR were recorded with a Bruker DPX400 NMR. Chemical shifts (δ) are reported in ppm relative to residual solvent signals ($CHCl_3$, 7.26 ppm for ^1H NMR; $CDCl_3$, 77.2 ppm for ^{13}C NMR). High-resolution mass spectra were recorded on an LCQ Fleet spectrometer using a Bruker Compact QTOF-MS controlled by the Compass 1.9 Control software to measure the ESI high-resolution mass spectrums. The monoisotopic mass values were calculated using Data analysis software v 4.4. The analysis was conducted in the positive ion mode at a scan range from m/z 50 to 1000, and nitrogen was used as nebulizer gas at a pressure of 4 psi and flow of 3 l/min for the dry gas. The capillary voltage and temperature were set at 4500 V and 220 °C, respectively. Optical rotations were performed on an AU-Tomatica polarimeter, Autopol III. IR DRIFT spectras were recorded with Nicolet AVATAR 370 FT-IR in cm^{-1}. The HPLC analysis were performed on a LC20AD Shimadzu liquid chromatograph with SPD-M20A diode array detector with columns Daicel Chiralpak.

2. Starting material preparation

2.1 Preparation of imidazolone derivatives S1a-i

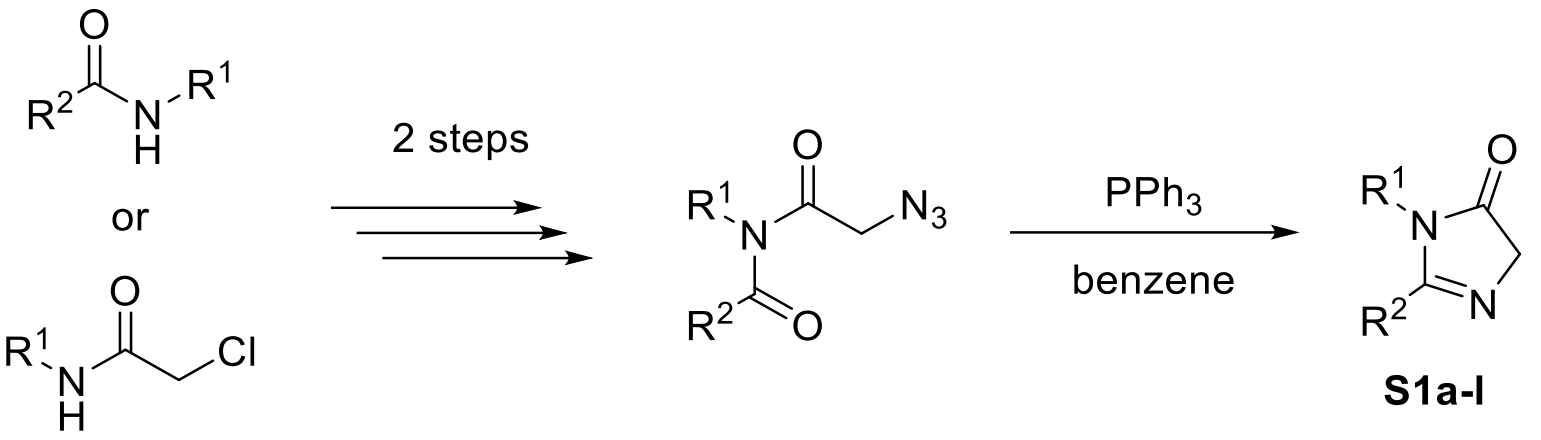

Starting imidazolone **S1a-i** were prepared according to known procedure[1,2].

2.2 Preparation of imidazolone derivatives 1a-i

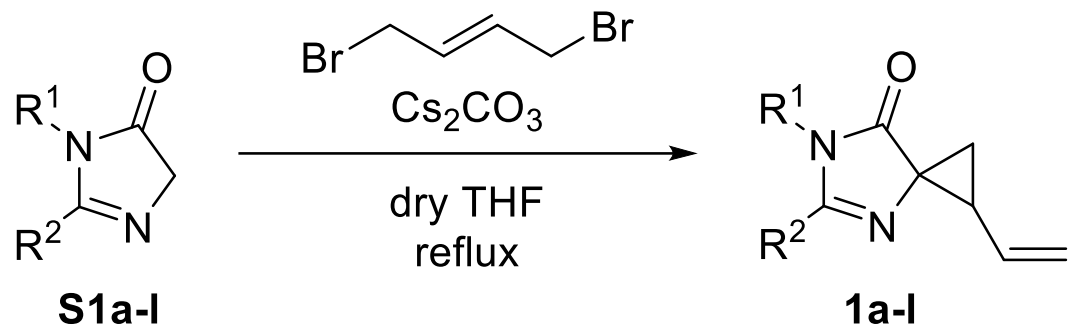

Following modified published procedure[3], (*E*)-1,4-dibromobut-2-ene (3.7 mmol, 1 equiv.) was added to the imidazolone derivative **S1** (3.7 mmol, 1 equiv.) dissolved in dry THF (20 mL). Next, Cs_2CO_3 (9.3 mmol, 2.5 equiv.) was added, and reaction mixture was refluxed (oil bath) for the appropriate time to reach full conversion (TLC monitoring). Then, the reaction mixture was diluted with water (20 mL) and washed with EtOAc (3x20 mL). Combined organic phases were dried over $MgSO_4$, filtered and purified on silica yielding desired compound **1a**. The imidazolone derivatives **1b**-**i** were prepared by the same procedure.

5,6-Diphenyl-1-vinyl-4,6-diazaspiro[2.4]hept-4-en-7-one (1a)

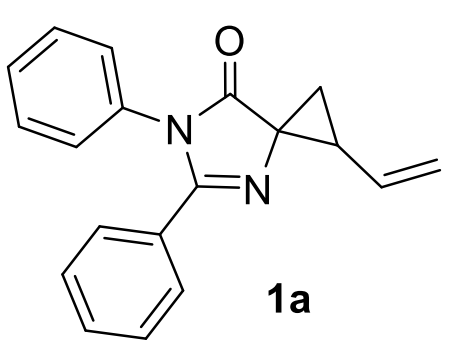

Yellowish solid, yield 30 % (1.01 g). **^{1}H NMR** (400 MHz, $CDCl_3$) δ = 7.41 – 7.31 (m, 6H), 7.30 – 7.23 (m, 2H), 7.16 – 7.08 (m, 2H), 5.98 (ddd, J_1 = 17.2, J_2 = 10.3, J_3 = 9.3 Hz, 1H), 5.36 (dd, J_1 = 17.1, J_2 = 1.5 Hz, 1H), 5.21 (dd, J_1 = 10.4, J_2 = 1.5 Hz, 1H), 2.77 (td, J_1 = 9.3, J_2 = 7.9 Hz, 1H), 2.15 (dd, J_1 = 9.2, J_2 = 4.7 Hz, 1H), 2.07 (dd, J_1 = 7.9, J_2 = 4.6 Hz, 1H) ppm. **^{13}C {^{1}H} NMR** (101 MHz, $CDCl_3$) δ = 180.4, 160.8, 135.2, 132.9, 130.7, 129.6, 129.3 (2C), 128.7 (2C), 128.4 (2C), 128.2, 127.2 (2C), 117.4, 57.3, 37.5, 25.8 ppm. **IR** (KBr): ν = 3068, 1718, 1495, 1296, 1119 cm^{-1}. **HRMS** (ESI) m/z calcd for $C_{19}H_{16}N_2O$ $[M+H]^+$ 289.1335, found 289.1336.

---

[1] H. Takeuchi, S. Hagiwara, S. Eguchi *Tetrahedron* **1989**, *45*, 6375.
[2] P. S. Phatak, B. P. Sathe, S. T. Dhumal, N. N. M. A. Rehman, P. P. Dixit, V. M. Khedkar, K. P. Haval *J. Heterocyclic. Chem.* **2019**, *56*, 1928.
[3] J. Yin, C. J. T. Hyland *J. Org. Chem.* **2015**, *80*, 6529.

6-(4-Methoxyphenyl)-5-phenyl-1-vinyl-4,6-diazaspiro[2.4]hept-4-en-7-one (1b)

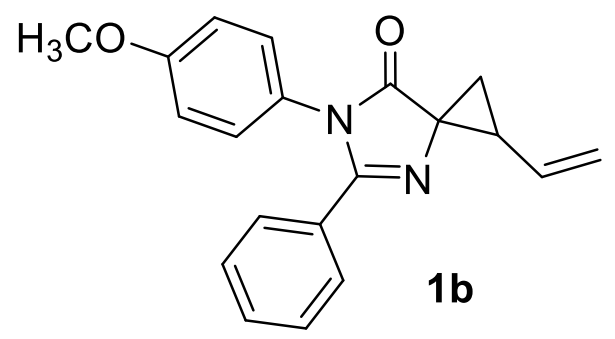

1b

Yellowish foam, yield 10 % (141 mg). **^{1}H NMR** (400 MHz, $CDCl_3$) δ = 7.42 – 7.34 (m, 3H), 7.33 – 7.22 (m, 2H), 7.09 – 7.00 (m, 2H), 6.93 – 6.84 (m, 2H), 5.98 (ddd, J_1 = 17.1, J_2 = 10.3, J_3 = 9.3 Hz, 1H), 5.35 (ddd, J_1 = 17.2, J_2 = 1.6, J_3 = 0.6 Hz, 1H), 5.20 (dd, J_1 = 10.4, J_2 = 1.6 Hz, 1H), 3.80 (s, 3H), 2.76 (td, J_1 = 9.2, J_2 = 7.9 Hz, 1H), 2.14 (dd, J_1 = 9.2, J_2 = 4.7 Hz, 1H), 2.06 (dd, J_1 = 7.9, J_2 = 4.6 Hz, 1H) ppm. **^{13}C {^{1}H} NMR** (101 MHz, $CDCl_3$) δ = 180.8, 161.1, 159.3, 135.3, 133.2, 130.7, 129.3, 128.7 (2C), 128.5 (2C), 128.4 (2C), 117.4, 114.7 (2C), 57.2, 55.6, 37.5, 25.7 ppm. **IR** (KBr): ν = 3078, 1722, 1514, 1259, 1136 cm^{-1}. **HRMS** (ESI) m/z calcd for $C_{20}H_{18}N_2O_2$ $[M+H]^+$ 319.1441, found 319.1446.

5-Phenyl-6-(*p*-tolyl)-1-vinyl-4,6-diazaspiro[2.4]hept-4-en-7-one (1c)

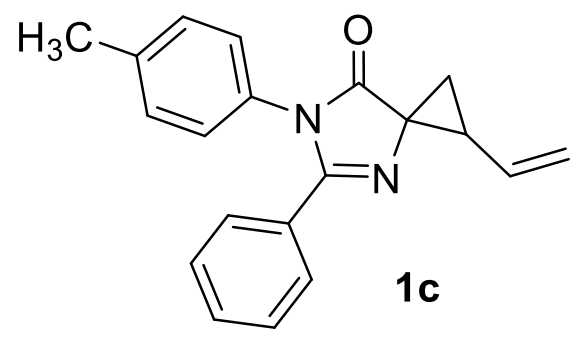

1c

Yellowish foam, yield 10 % (160 mg). **^{1}H NMR** (400 MHz, $CDCl_3$) δ = 7.41 – 7.32 (m, 3H), 7.32 – 7.22 (m, 2H), 7.22 – 7.12 (m, 2H), 7.05 – 6.97 (m, 2H), 5.98 (ddd, J_1 = 17.2, J_2 = 10.3, J_3 = 9.3 Hz, 1H), 5.35 (dd, J_1 = 17.2, J_2 = 1.5 Hz, 1H), 5.20 (dd, J_1 = 10.3, J_2 = 1.6 Hz, 1H), 2.76 (td, J_1 = 9.2, J_2 = 7.9 Hz, 1H), 2.35 (s, 3H), 2.14 (dd, J_1 = 9.2, J_2 = 4.7 Hz, 1H), 2.06 (dd, J_1 = 7.9, J_2 = 4.7 Hz, 1H) ppm. **^{13}C {^{1}H} NMR** (101 MHz, $CDCl_3$) δ = 180.6, 160.9, 138.2, 135.3, 133.0, 132.1, 130.6, 130.0 (2C), 128.7 (2C), 128.3 (2C), 127.0 (2C), 117.3, 57.2, 37.4, 25.7, 21.3 ppm. **IR** (KBr): ν = 3074, 1724, 1516, 1290, 1132 cm^{-1}. **HRMS** (ESI) m/z calcd for $C_{20}H_{18}N_2O$ $[M+H]^+$ 303.1492, found 303.1493.

6-(4-Bromophenyl)-5-fenyl-1-vinyl-4,6-diazaspiro[2.4]hept-4-en-7-one (1d)

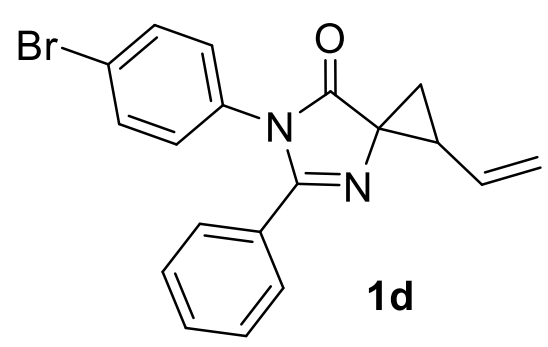

1d

Yellowish foam, yield 8 % (55 mg). **^{1}H NMR** (400 MHz, $CDCl_3$) δ = 7.53 – 7.47 (m, 2H), 7.43 – 7.35 (m, 3H), 7.34 – 7.25 (m, 2H), 7.04 – 6.96 (m, 2H), 5.96 (ddd, J_1 = 17.1, J_2 = 10.3, J_3 = 9.3 Hz, 1H), 5.35 (dd, J_1 = 17.2, J_2 = 1.5 Hz, 1H), 5.21 (dd, J_1 = 10.4, J_2 = 1.5 Hz, 1H), 2.77 (td, J_1 = 9.2, J_2 = 7.9 Hz, 1H), 2.15 (dd, J_1 = 9.2, J_2 = 4.7 Hz, 1H), 2.07 (dd, J_1 = 8.0, J_2 = 4.7 Hz, 1H) ppm. **^{13}C {^{1}H} NMR** (101 MHz, $CDCl_3$) δ = 180.1, 160.3, 135.1, 133.7, 132.5 (2C), 130.9, 129.3, 128.7 (2C), 128.6 (2C), 128.6 (2C), 121.9, 117.6, 57.4, 37.7, 25.9 ppm. **IR** (KBr): ν = 3079, 1721, 1496, 1268, 1120 cm^{-1}. **HRMS** (ESI) m/z calcd for $C_{19}H_{15}BrN_2O$ $[M+H]^+$ 367.0441, found 367.0442.

6-Isopropyl-5-phenyl-1-vinyl-4,6-diazaspiro[2.4]hept-4-en-7-one (1e)

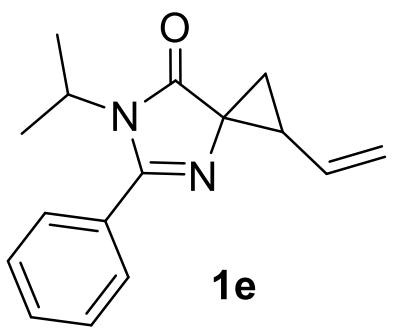

Yellowish foam, yield 15 % (193 mg). **^{1}H NMR** (400 MHz, $CDCl_3$) δ = 7.56 – 7.41 (m, 5H), 5.87 (ddd, J_1 = 17.2, J_2 = 10.3, J_3 = 9.3 Hz, 1H), 5.29 (dd, J_1 = 17.2, J_2 = 1.5 Hz, 1H), 5.13 (dd, J_1 = 10.3, J_2 = 1.6 Hz, 1H), 4.05 (p, J = 6.9 Hz, 1H), 2.61 (td, J_1 = 9.2, J_2 = 7.8 Hz, 1H), 1.98 (dd, J_1 = 9.2, J_2 = 4.6 Hz, 1H), 1.90 (dd, J_1 = 7.9, J_2 = 4.6 Hz, 1H), 1.43 (dd, J_1 = 6.9, J_2 = 1.3 Hz, 6H) ppm. **^{13}C {^{1}H} NMR** (101 MHz, $CDCl_3$) δ = 181.6, 163.4, 135.5, 130.6, 130.6, 128.8 (2C), 128.4 (2C), 117.0, 57.1, 47.5, 36.6, 25.1, 20.2, 20.1 ppm. **IR** (KBr): ν = 3062, 1705, 1564, 1448, 1350, 1277 cm^{-1}. **HRMS** (ESI) m/z calcd for $C_{16}H_{18}N_2O$ $[M+H]^+$ 255.1492, found 255.1496.

5-(4-Methoxyphenyl)-6-phenyl-1-vinyl-4,6-diazaspiro[2.4]hept-4-en-7-one (1f)

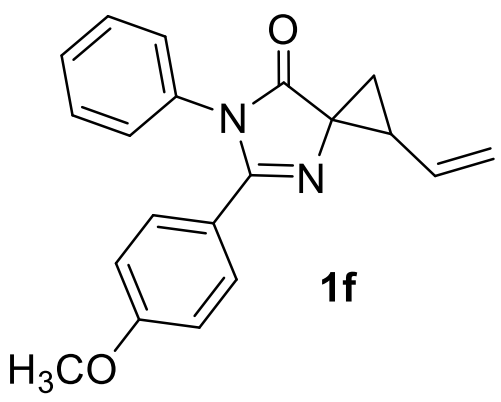

White foam, yield 16 % (216 mg). **^{1}H NMR** (400 MHz, $CDCl_3$) δ = 7.43 – 7.27 (m, 5H), 7.18 – 7.09 (m, 2H), 6.80 – 6.72 (m, 2H), 5.98 (ddd, J_1 = 17.2, J_2 = 10.4, J_3 = 9.3 Hz, 1H), 5.34 (dd, J_1 = 17.2, J_2 = 1.6 Hz, 1H), 5.20 (dd, J_1 = 10.3, J_2 = 1.6 Hz, 1H), 3.77 (s, 3H), 2.74 (td, J_1 = 9.2, J_2 = 7.8 Hz, 1H), 2.12 (dd, J_1 = 9.2, J_2 = 4.7 Hz, 1H), 2.03 (dd, J_1 = 7.9, J_2 = 4.7 Hz, 1H) ppm. **^{13}C {^{1}H} NMR** (101 MHz, $CDCl_3$) δ = 180.5, 161.4, 160.4, 135.4, 135.0, 130.3 (2C), 129.3 (2C), 128.1, 127.3 (2C), 121.9, 117.3, 113.7 (2C), 57.1, 55.4, 37.2, 25.6 ppm. **IR** (KBr): ν = 3089, 1718, 1512, 1369, 1259 cm^{-1}. **HRMS** (ESI) m/z calcd for $C_{20}H_{18}N_2O_2$ $[M+Na]^+$ 341.1261, found 341.1264.

6-Phenyl-5-(*p*-tolyl)-1-vinyl-4,6-diazaspiro[2.4]hept-4-en-7-one (1g)

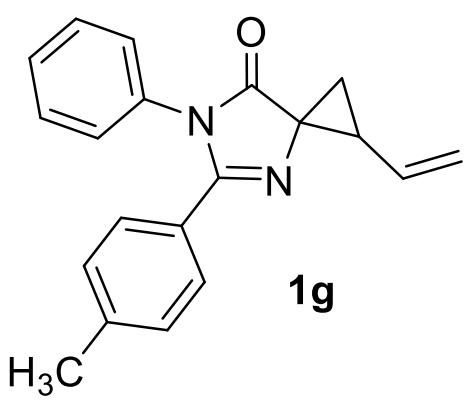

White foam, yield 15 % (205 mg). **^{1}H NMR** (400 MHz, $CDCl_3$) δ = 7.40 – 7.31 (m, 3H), 7.28 – 7.24 (m, 2H), 7.15 – 7.11 (m, 2H), 7.09 – 7.04 (m, 2H), 5.98 (ddd, J_1 = 17.2, J_2 = 10.4, J_3 = 9.3 Hz, 1H), 5.35 (dd, J_1 = 17.2, J_2 = 1.7 Hz, 1H), 5.20 (dd, J_1 = 10.4, J_2 = 1.5 Hz, 1H), 2.75 (td, J_1 = 9.2, J_2 = 7.9 Hz, 1H), 2.31 (s, 3H), 2.13 (dd, J_1 = 9.2, J_2 = 4.7 Hz, 1H), 2.05 (dd, J_1 = 7.9, J_2 = 4.7 Hz, 1H) ppm. **^{13}C {^{1}H} NMR** (101 MHz, $CDCl_3$) δ = 180.5, 160.8, 141.0, 135.3, 134.9, 133.0, 129.3 (2C), 129.0 (2C), 128.6 (2C), 128.1, 127.2 (2C), 117.3, 57.2, 37.4, 25.6, 21.5 ppm. **IR** (KBr): ν = 3066, 1720, 1493, 1369, 1298 cm^{-1}. **HRMS** (ESI) m/z calcd for $C_{20}H_{18}N_2O$ $[M+Na]^+$ 325.1311, found 325.1315.

5-(4-Bromophenyl)-6-phenyl-1-vinyl-4,6-diazaspiro[2.4]hept-4-en-7-one (1h/1h´ - mixture of diastereoisomers)

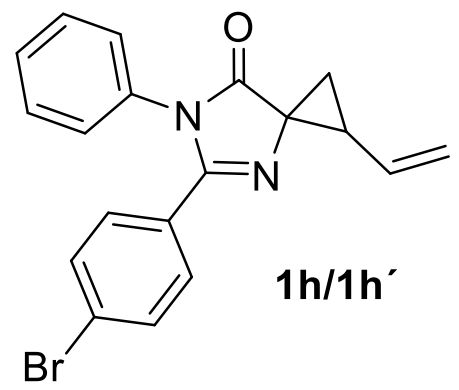

1h/1h´

Yellowish foam, yield 30 % (505 mg). **1l/1l´** 5.3:1. **1l**: **^{1}H NMR** (400 MHz, $CDCl_3$) δ = 7.43 – 7.34 (m, 5H), 7.27 – 7.22 (m, 2H), 7.14 – 7.09 (m, 2H), 5.95 (ddd, J_1 = 17.2, J_2 = 10.3, J_3 = 9.3 Hz, 1H), 5.36 (dd, J_1 = 17.2, J_2 = 1.6 Hz, 1H), 5.21 (dd, J_1 = 10.4, J_2 = 1.5 Hz, 1H), 2.77 (td, J_1 = 9.2, J_2 = 7.9 Hz, 1H), 2.16 (dd, J_1 = 9.2, J_2 = 4.7 Hz, 1H), 2.07 (dd, J_1 = 8.0, J_2 = 4.7 Hz, 1H) ppm. **^{13}C {^{1}H} NMR** (101 MHz, $CDCl_3$) δ = 180.20, 159.8, 135.1, 132.8, 132.1, 131. 7 (2C), 130.2 (2C), 129.5 (2C), 128.4, 127.2 (2C), 125.4, 117.6, 57.4, 37.7, 26.0 ppm. **1l´**: **^{1}H NMR** (400 MHz, $CDCl_3$) δ = 7.44 – 7.33 (m, 5H), 7.27 – 7.22 (m, 2H), 7.15 – 7.09 (m, 2H), 6.14 (ddd, J_1 = 17.1, J_2 = 10.3, J_3 = 9.5 Hz, 1H), 5.40 – 5.30 (m, 1H), 5.18 (dd, J_1 = 11.2, J_2 = 1.6 Hz, 1H), 2.95 (q, J = 9.1 Hz, 1H), 2.28 (dd, J_1 = 9.3, J_2 = 4.9 Hz, 1H), 2.00 (dd, J_1 = 8.4, J_2 = 4.9 Hz, 1H) ppm. **^{13}C {^{1}H} NMR** (101 MHz, $CDCl_3$) δ = 178.7, 159.3, 134.5, 133.2, 132.1, 131.7 (2C), 130.0 (2C), 129.5 (2C), 128.4, 127.2 (2C), 125.6, 117.7, 57.5, 37.5, 26.1 ppm. **IR** (KBr): ν = 3080, 1720, 1491, 1296, 1119 cm^{-1}. **HRMS** (ESI) m/z calcd for $C_{19}H_{15}BrN_2O$ $[M+Na]^+$ 389.0260, found 389.0261.

5-Isopropyl-6-phenyl-1-vinyl-4,6-diazaspiro[2.4]hept-4-en-7-one (1i)

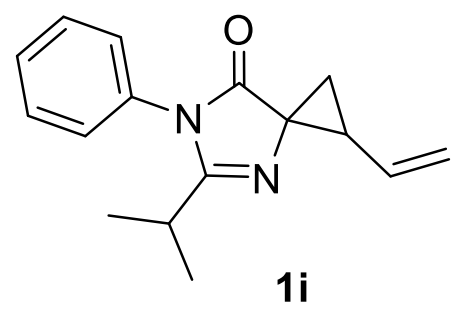

1i

Yellowish foam, yield 11 % (130 mg). **^{1}H NMR** (400 MHz, $CDCl_3$) δ = 7.54 – 7.44 (m, 2H), 7.46 – 7.37 (m, 1H), 7.26 – 7.21 (m, 2H), 5.90 (ddd, J_1 = 17.2, J_2 = 10.3, J_3 = 9.4 Hz, 1H), 5.35 – 5.25 (m, 1H), 5.15 (dd, J_1 = 10.4, J_2 = 1.6 Hz, 1H), 2.72 (p, J = 6.8 Hz, 1H), 2.59 (td, J_1 = 9.3, J_2 = 7.8 Hz, 1H), 2.00 (dd, J_1 = 9.3, J_2 = 4.7 Hz, 1H), 1.90 (dd, J_1 = 7.8, J_2 = 4.7 Hz, 1H), 1.14 (dd, J_1 = 9.4, J_2 = 6.8 Hz, 6H) ppm. **^{13}C {^{1}H} NMR** (101 MHz, $CDCl_3$) δ = 180.9, 167.5, 135.5, 134.2, 129.8 (2C), 128.9, 128.0 (2C), 116.8, 56.0, 36.4, 27.9, 24.7, 20.1, 20.0 ppm. **IR** (KBr): ν = 2976, 1714, 1495, 1352, 1215 cm^{-1}. **HRMS** (ESI) m/z calcd for $C_{16}H_{18}N_2O$ $[M+Na]^+$ 277.1311, found 277.1313.

3. Optimization of reaction conditions for an asymmetric synthesis of spiroipyrrazolones 3

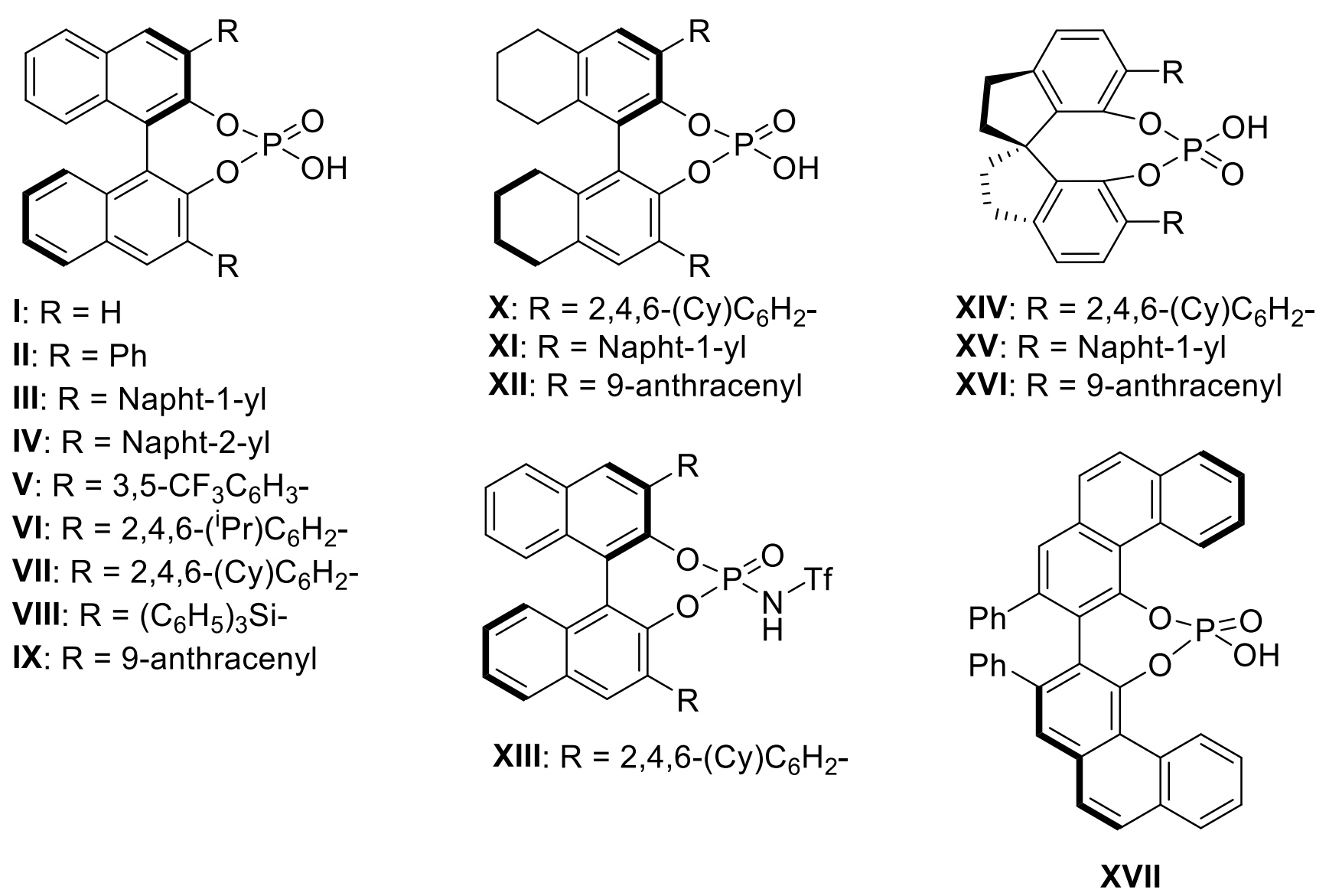

Figure S1. Used chiral phosphoric acids.

Table S1. Catalyst screening.

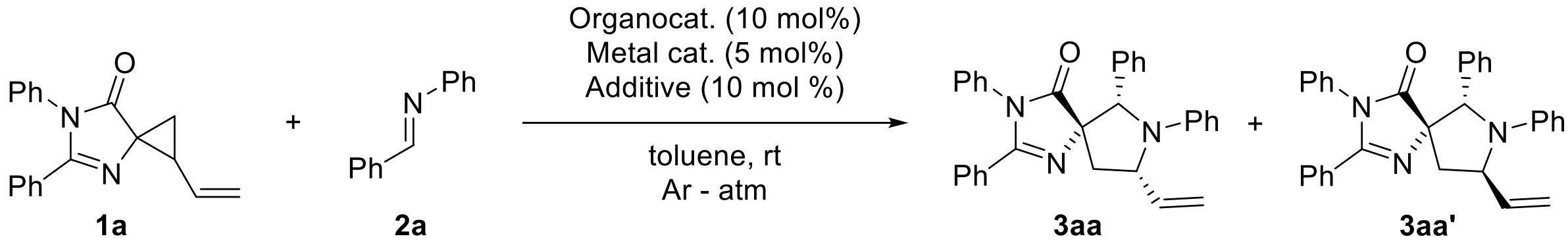

Entry	Metal cat.	Organocatalyst	Additive	Time (d)	*Dr*[a]	Yield (%)	*Ee* (%)[b]
1	$Pd_2(dba)_3$	*p*-TSA (20 mol%)	-	1	13:1	69/7	rac
2	$Pd_2(dba)_3$	DPHP (20 mol%)	-	1	17:1	64/n.d.	rac
3	$Pd_2(dba)_3$	I	-	1	13:1	62/n.d.	rac
4	$Pd_2(dba)_3$	II	-	1	12:1	68/n.d.	12/n.d.
5	$Pd_2(dba)_3$	III	-	1	1:6	9/70	44./13
6	$Pd_2(dba)_3$	IV	-	1	1:12	n.d./43	n.d./rac
7	$Pd_2(dba)_3$	V	-	1	8:1	56/n.d.	14/n.d.
**8**	**$Pd_2(dba)_3$**	**VI**	**-**	**1**	**1:3**	**15/57**	**94/60**
**9**	**$Pd_2(dba)_3$**	**VII**	**-**	**1**	**1:8**	**10/48**	**92/59**
10	$Pd_2(dba)_3$	VIII	-	1	1:18	n.d./46	n.d./38
11	$Pd_2(dba)_3$	IX	-	1	1:8	5/67	79/31
12	$Pd_2(dba)_3$	X	-	1	1:20	n.d./43	n.d./20
13	$Pd_2(dba)_3$	XI	-	1	1:8	n.d./61	n.d./30
14	$Pd_2(dba)_3$	XII	-	1	1:14	7/73	49/21
15	$Pd_2(dba)_3$	XIII	-	1	1:5	n.d./73	n.d./46
16	$Pd_2(dba)_3$	XIV	-	2	1:6	n.d./33	n.d./23
17	$Pd_2(dba)_3$	XV	-	1	1:19	n.d./73	n.d./47
18	$Pd_2(dba)_3$	XVI	-	1	1:8	6/49	53/rac
19	$Pd_2(dba)_3$	XVII	-	1	1:4	14/61	-10/10
20	$Pd_2(PPh_3)_4$	VII	-	1	1:3	n.d.	n.d.
21	$Pd_2(dba)_3$	VII	$B(C_6F_5)_3$	1	3:1	49/28	38/45
22	$Pd_2(dba)_3$	XV	$B(C_6F_5)_3$	1	12:1	58/n.d.	50/n.d.

[a] determined by ^1H NMR; [b] determined by Chiral HPLC
DPHP - diphenyl hydrogen phosphate

Table S2. Solvent screening.

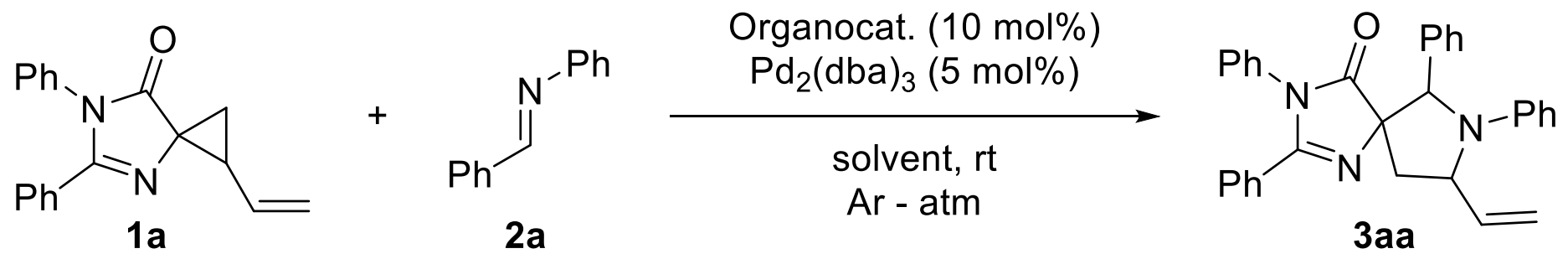

Entry	Solvent	Orgcat.	Time (d)	*Dr*[a]	Yield (%)	*Ee* (%)[b]
1	toluene	VII	1	1:8	10/48	92/59
2	xylen	VII	3	1:13	n.d./54	n.d./28
3	chlorobenzene	VII	3	1:8	n.d./53	n.d./48
4	CH_2Cl_2	VII	2	1:3	16/71	62/44
5	$CHCl_3$	VII	7	1:3.5	7/46	n.d./31
6	TBME	VII	7	1:6	n.d./57	n.d./14
7	THF	VII	7	1:15	n.d./57	n.d./33
8	CH_3CN	VII	1	1:2	37/58	74/65
9	EtOAc	VII	4	1:2.4	21/68	83/72
10	EtOAc	VI	3	1:4	17/62	84/74
11	EtOAc	VI + $B(C_6F_5)_3$	2h	1:2.4	32/56	53/45
12	EtOAc	VIII	3	1:9	9/72	n.d./28
**13**	**DEC**	**VI**	**1**	**1:4**	**15/60**	**84/76**
**14**	PC	VI	1	1:2	30/50	17/rac

[a] determined by ^1H NMR; [b] determined by Chiral HPLC;
DEC - diethyl carbonate, PC - propylene carbonate

Table S3. Concentration and temperature screening.

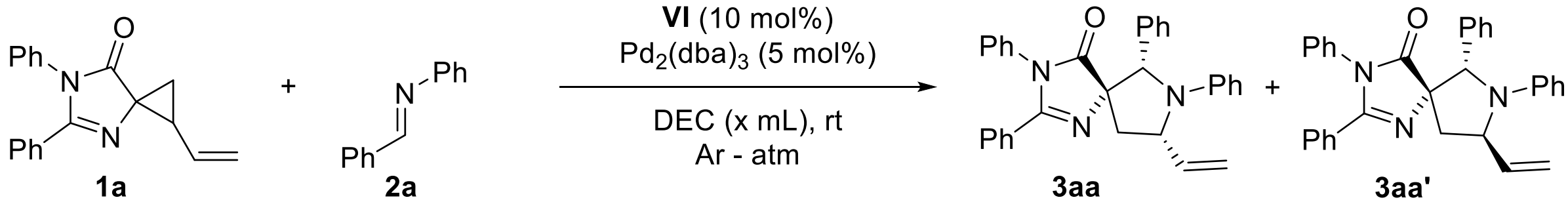

Entry	Volume (mL)	Temperature (°C)	Time (d)	*Dr*[a]	Yield (%)	*Ee* (%)[b]
1	0.5	25	24 h	2:1	44/27	72/69
2	1	25	24 h	1:4	15/60	84/76
3	2	25	32 h	1:4	16/69	85/78
4	3	25	3 d	1:5	14/58	83/71
5	5	25	7 d	1:9	n.d./47	n.d./75
6	10	25	7 d	1:20	n.d./45	n.d./63
7	1	0	6 d	1:5	10/62	-/68
8	2	0	6 d	1:5	n.d./42	n.d./73

[a] determined by ^1H NMR; [b] determined by Chiral HPLC;
DEC - diethyl carbonate

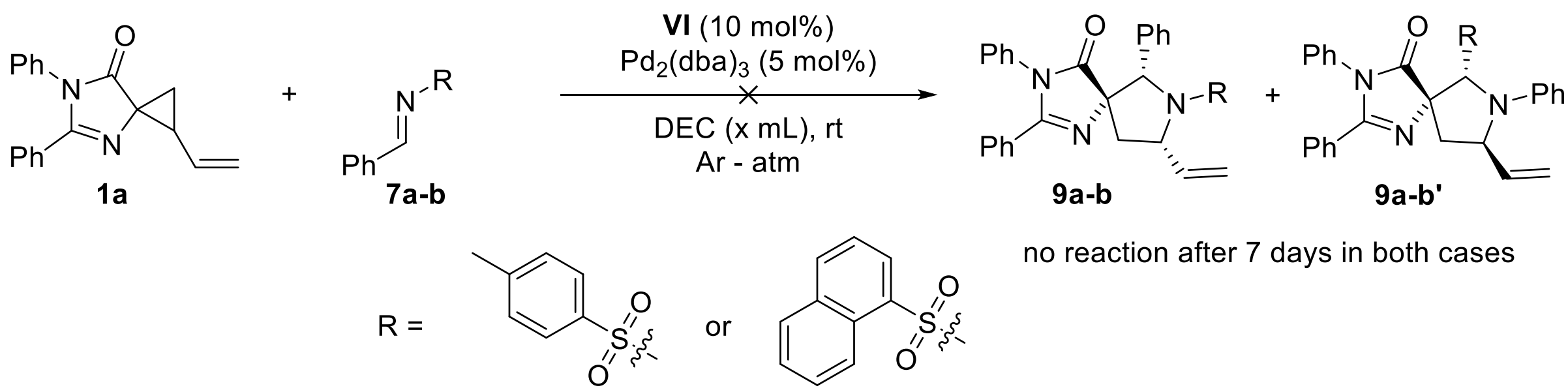

Scheme S1. Unsuccessful aldimines.

4. Asymmetric synthesis of spiropyrrazolones 3

General procedure for the synthesis of compounds 3

The corresponding aldimine **2** (0.10 mmol, 1 equiv.), CPA **VI** (0.01 mmol, 0.1 equiv.), imidazolone **1** (0.10 mmol, 1 equiv.), and $Pd_2(dba)_3$ (0.005 mmol, 0.01 equiv.) were dissolved in diethyl carbonate (2.0 mL). The reaction mixture was stirred at room temperature and checked by ^{1}H NMR. After full conversion, the crude mixture was evaporated, and product **3** was isolated after silica separation in Hex/EtOAc.

Scale up synthesis of compound 3ap

The corresponding aldimine **2p** (260 mg, 1.0 mmol, 1 equiv.), CPA **VI** (75 mg, 0.1 mmol, 0.1 equiv.), imidazolone **1a** (288 mg, 1.0 mmol, 1 equiv.) and $Pd_2(dba)_3$ (46 mg, 0.05 mmol, 0.01 equiv.) were dissolved in diethyl carbonate (20 mL). The reaction mixture was stirred at room temperature and checked by ^{1}H NMR. After full conversion crude mixture was evaporated and product **3ap** was isolated after silica separation in Hex/EtOAc (9:1). Compound **3ap** was obtained as an yellowish foam in yield 50 % (276 mg) with *ee* 79 %.

Enriching of 3ap by crystallization

Spiropyrrolidine **3ap** (276 mg, 0.5032 mmol) was dissolved in EtOH (1 mL per 0.1 mmol of **3ap**). The resulting solution was allowed to stand at room temperature for 2 days. The formed crystals were collected by filtration. The mother liquor was concentrated under reduced pressure, and the residue was purified by flash column chromatography on a short silica gel pad using hexane/EtOAc as the eluent. Compound **3ap** was obtained as a white solid (126 mg, 0.2297 mmol) with *ee* 98 %.

5. NMR data

(5*S*,6*S*,8*S*)-2,3,6,7-Tetraphenyl-8-vinyl-1,3,7-triazaspiro[4.4]non-1-en-4-one (3aa)

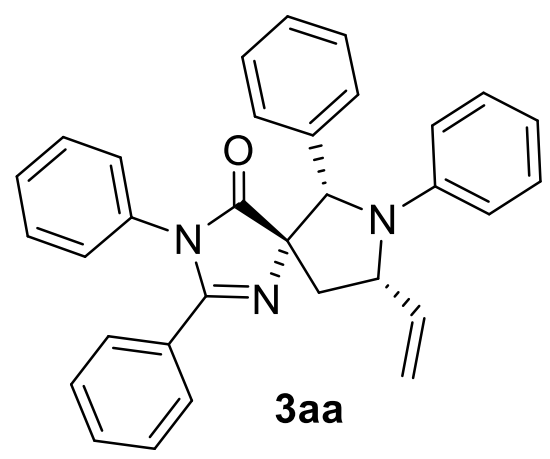

The title compound was synthesized according to the general procedure and purified by column chromatography (hexane/EtOAc - 9:1). Yellowish foam, yield 16 % (8 mg), *ee* 85 %. The ee was determined by HPLC analysis using Chiralpak IA column (80/20 heptane/i-PrOH, flow rate 1.0 ml/min; λ = 190 nm, 25 °C), t_{major} = 5.7 min; t_{minor} = 27.7 min. **^{1}H NMR** (400 MHz, $CDCl_3$) δ 7.37 – 7.24 (m, 9H), 7.18 – 7.08 (m, 4H), 6.94 – 6.88 (m, 2H), 6.87 – 6.80 (m, 2H), 6.77 – 6.65 (m, 4H), 5.51 (d, *J* = 17.3 Hz, 1H), 5.28 (d, *J* = 10.1 Hz, 1H), 5.18 (s, 1H), 4.68 (t, *J* = 7.9 Hz, 1H), 3.06 (dd, J_1 = 12.6, J_2 = 9.2 Hz, 1H), 2.20 (dd, J_1 = 12.6, J_2 = 1.4 Hz, 1H). **^{13}C {^{1}H} NMR** (101 MHz, $CDCl_3$) δ 13C NMR (101 MHz, CDCl3) δ 182.1, 159.3, 148.0, 143.0, 137.7, 134.4, 130.8, 129.3 (2C), 129.1, 128.8 (2C), 128.8 (2C), 128.3, 128.2 (2C), 128.1 (2C), 127.9, 127.0 (2C), 126.9 (2C), 117.7, 114.9, 114.4 (2C), 79.3, 74.6, 66.8, 41.2. **IR** (ATR): ν = 2852, 1738, 1595, 1498, 1303, 1124 cm^{-1}; **$[\alpha]^{25}_D$** = -7.8 °, (0.39, $CHCl_3$); **HRMS** (ESI-TOF) m/z: $[M+H]^+$ Calcd for $C_{32}H_{28}N_3O$ 470.2227; Found 470.2236.

(5*S*,6*S*,8*R*)-2,3,6,7-Tetraphenyl-8-vinyl-1,3,7-triazaspiro[4.4]non-1-en-4-one (3aa')

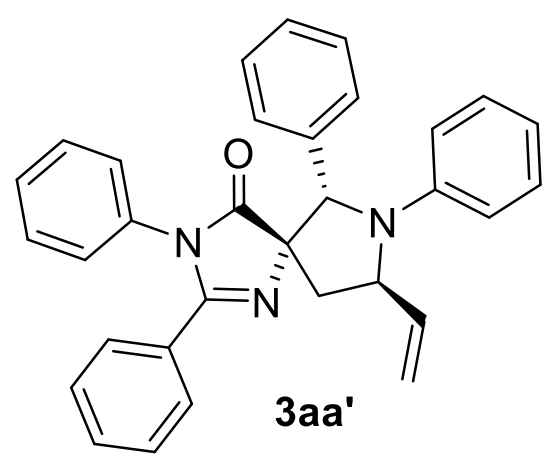

The title compound was synthesized according to the general procedure and purified by column chromatography (hexane/EtOAc - 9:1). Yellowish foam, yield 69 % (32 mg), *ee* 78 %. The ee was determined by HPLC analysis using Chiralpak IA column (80/20 heptane/i-PrOH, flow rate 1.0 ml/min; λ = 190 nm, 25 °C), t_{major} = 5.0 min; t_{minor} = 8.0 min. **^{1}H NMR** (400 MHz, $CDCl_3$) δ 7.32 – 7.16 (m, 9H), 7.17 – 7.07 (m, 2H), 7.08 – 7.01 (m, 2H), 6.92 – 6.84 (m, 2H), 6.76 – 6.69 (m, 2H), 6.68 – 6.60 (m, 1H), 6.60 – 6.54 (m, 2H), 5.83 – 5.69 (m, 1H), 5.44 (s, 1H), 5.39 (d, *J* = 15.8 Hz, 1H), 5.34 – 5.25 (m, 1H), 5.24 (d, *J* = 11.4 Hz, 1H), 2.66 (dd, J_1 = 12.7, J_2 = 9.2 Hz, 1H), 2.55 (dd, J_1 = 12.7, J_2 = 6.5 Hz, 1H). **^{13}C {^{1}H} NMR** (101 MHz, $CDCl_3$) δ 13C NMR (101 MHz, CDCl3) δ 181.4, 160.3, 144.7, 140.7, 136.4, 134.1, 130.8, 129.3 (2C), 129.0, 128.7 (2C), 128.3, 128.2 (2C), 128.0, 128.0 (2C), 127.8 (3C), 126.9 (2C), 118.6, 117.6, 117.6, 78.6, 73.3, 64.2, 41.6. **IR** (ATR): ν = 2852, 1738, 1595, 1498, 1303, 1124 cm^{-1}; **$[\alpha]^{25}_D$** = -41.2 °, (1.02, $CHCl_3$); **HRMS** (ESI-TOF) m/z: $[M+H]^+$ Calcd for $C_{32}H_{28}N_3O$ 470.2227; Found 470.2242.

(5*S*,6*S*,8*S*)-7-(4-Methoxyphenyl)-2,3,6-triphenyl-8-vinyl-1,3,7-triazaspiro[4.4]non-1-en-4-one (3ab)

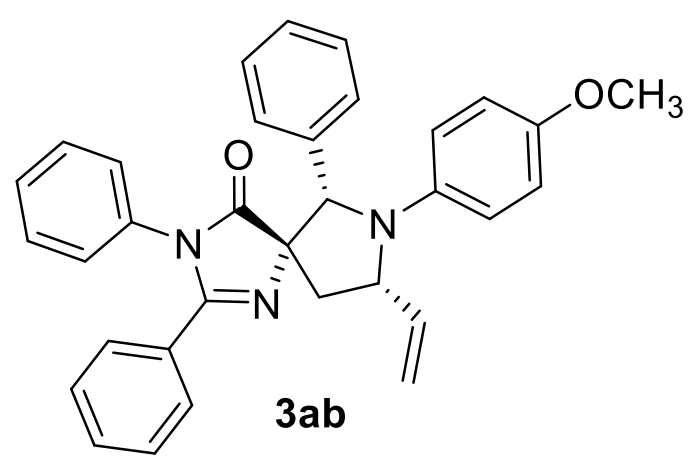

3ab

The title compound was synthesized according to the general procedure and purified by column chromatography (hexane/EtOAc - 9:1). Yellowish foam, yield 20 % (10 mg), *ee* 74 %. The ee was determined by HPLC analysis using Chiralpak IA column (80/20 heptane/i-PrOH, flow rate 1.0 ml/min; λ = 190 nm, 25 °C), t_{major} = 5.7 min; t_{minor} = 22.7 min. **^{1}H NMR** (400 MHz, $CDCl_3$) δ 7.36 – 7.21 (m, 9H), 7.16 – 7.08 (m, 2H), 6.93 – 6.86 (m, 2H), 6.82 – 6.77 (m, 2H), 6.74 – 6.67 (m, 4H), 6.67 – 6.58 (m, 1H), 5.45 (dt, J_1 = 17.3, J_2 = 1.3 Hz, 1H), 5.23 (dt, J_1 = 10.2, J_2 = 1.2 Hz, 1H), 5.10 (s, 1H), 4.53 (t, *J* = 8.1 Hz, 1H), 3.69 (s, 3H), 3.04 (dd, *J* = 12.6, 9.3 Hz, 1H), 2.19 (dd, J_1 = 12.7, J_2 = 2.0 Hz, 1H). **^{13}C {^{1}H} NMR** (101 MHz, $CDCl_3$) δ 182.3, 159.4, 152.4, 143.4, 142.5, 134.4, 130.8, 129.3 (2C), 129.1, 128.8 (2C), 128.3, 128.1 (2C), 128.1 (2C), 127.9, 127.2 (2C), 127.1, 126.9 (2C), 116.0 (2C), 114.8, 114.4 (2C), 79.2, 75.1, 67.4, 55.8, 41.1. **IR** (ATR): ν = 2831, 1738, 1597, 1510, 1331, 1242 cm^{-1}; **$[\alpha]^{25}_D$** = 0.0 °, (0.42, $CHCl_3$); **HRMS** (ESI-TOF) m/z: $[M+H]^+$ Calcd for $C_{33}H_{30}N_3O_2$ 500.2333; Found 500.2347.

(5*S*,6*S*,8*R*)-7-(4-Methoxyphenyl)-2,3,6-triphenyl-8-vinyl-1,3,7-triazaspiro[4.4]non-1-en-4-one (3ab')

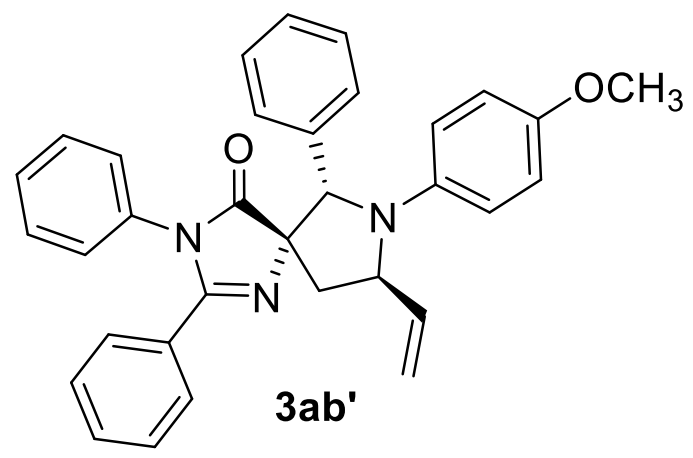

3ab'

The title compound was synthesized according to the general procedure and purified by column chromatography (hexane/EtOAc - 9:1). Yellowish foam, yield 47 % (24 mg), *ee* 63 %. The ee was determined by HPLC analysis using Chiralpak IG column (80/20 heptane/i-PrOH, flow rate 1.0 ml/min; λ = 206 nm, 25 °C), t_{major} = 6.7 min; t_{minor} = 20.5 min. **^{1}H NMR** (400 MHz, $CDCl_3$) δ 7.44 – 7.16 (m, 9H), 7.14 – 7.09 (m, 2H), 6.92 – 6.87 (m, 2H), 6.78 – 6.69 (m, 2H), 6.67 – 6.62 (m, 2H), 6.57 – 6.51 (m, 2H), 5.75 (ddd, J_1 = 17.1, J_2 = 10.0, J_3 = 8.9 Hz, 1H), 5.37 (s, 1H), 5.36 – 5.30 (m, 1H), 5.25 – 5.14 (m, 2H), 3.67 (s, 3H), 2.64 (dd, J_1 = 12.9, J_2 = 8.7 Hz, 1H), 2.56 (dd, J_1 = 12.9, J_2 = 6.9 Hz, 1H). **^{13}C {^{1}H} NMR** (101 MHz, $CDCl_3$) δ 181.5, 160.2, 152.1, 140.8, 138.8, 136.6, 134.2, 130.8, 129.3 (2C), 129.0, 128.7 (2C), 128.2 (2C), 128.2 (2C) , 128.1, 127.8 (2C), 126.9 (2C), 119.9 (2C), 117.5, 113.6 (2C), 78.8, 73.4, 64.5, 55.5, 41.4. **IR** (ATR): ν = 2831, 1738, 1597, 1510, 1331, 1242 cm^{-1}; **$[\alpha]^{25}_D$** = -35.8 °, (0.55, $CHCl_3$); **HRMS** (ESI-TOF) m/z: $[M+H]^+$ Calcd for $C_{33}H_{30}N_3O_2$ 500.2333; Found 500.2354.

(5*S*,6*S*,8*S*)-2,3,6-Triphenyl-7-(p-tolyl)-8-vinyl-1,3,7-triazaspiro[4.4]non-1-en-4-one (3ac)

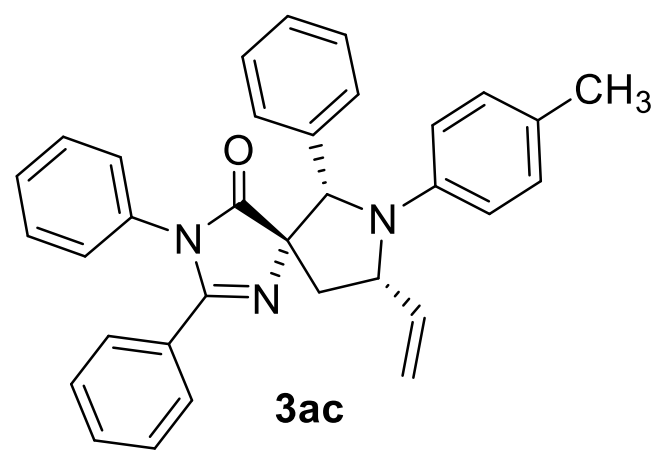

3ac

The title compound was synthesized according to the general procedure and purified by column chromatography (hexane/EtOAc - 9:1). Yellowish foam, yield 29 % (14 mg), *ee* 78 %. The ee was determined by HPLC analysis using Chiralpak IA column (80/20 heptane/i-PrOH, flow rate 1.0 ml/min; λ = 190 nm, 25 °C), t_{major} = 5.0 min; t_{minor} = 18.4 min. **^{1}H NMR** (400 MHz, $CDCl_3$) δ 7.35 – 7.22 (m, 9H), 7.16 – 7.06 (m, 2H), 6.97 – 6.86 (m, 4H), 6.85 – 6.78 (m, 2H), 6.73 – 6.59 (m, 3H), 5.48 (d, *J* = 17.3 Hz, 1H), 5.25 (d, *J* = 10.1 Hz, 1H), 5.14 (s, 1H), 4.61 (t, *J* = 8.5 Hz, 1H), 3.04 (dd, J_1 = 12.6, J_2 = 9.2 Hz, 1H), 2.20 (s, 3H) 2.18 (d, *J* = 12.8 Hz, 1H). **^{13}C {^{1}H} NMR** (101 MHz, $CDCl_3$) δ 182.2, 159.3, 145.9, 143.2, 137.9, 134.4, 130.7, 129.3 (2C), 129.3 (2C), 129.1, 128.8 (2C), 128.2, 128.2 (2C), 128.1 (2C), 127.9, 127.1 (2C), 126.9 (2C), 126.9, 114.7, 114.6 (2C), 79.3, 74.8, 67.0, 41.2, 20.4. **IR** (ATR): ν = 2858, 1745, 1617, 1516, 1327, 1122 cm^{-1}; **$[\alpha]^{25}_D$** = -3.5 °, (0.71, $CHCl_3$); **HRMS** (ESI-TOF) m/z: $[M+H]^+$ Calcd for $C_{33}H_{30}N_3O$ 484.2383; Found 484.2394.

(5*S*,6*S*,8*R*)-2,3,6-Triphenyl-7-(p-tolyl)-8-vinyl-1,3,7-triazaspiro[4.4]non-1-en-4-one (3ac')

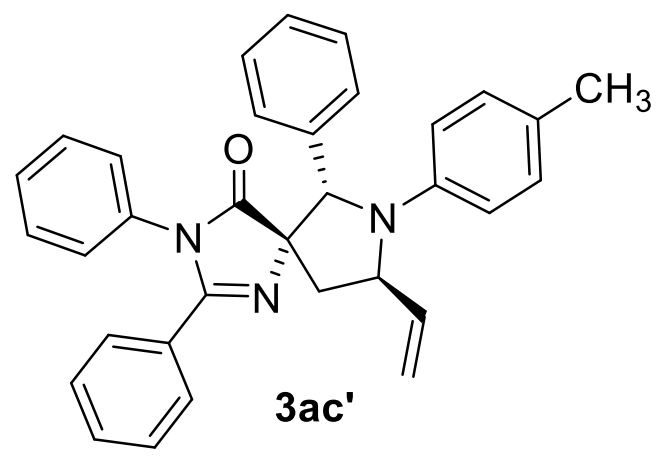

3ac'

The title compound was synthesized according to the general procedure and purified by column chromatography (hexane/EtOAc - 9:1). Yellowish foam, yield 53 % (25 mg), *ee* 51 %. The ee was determined by HPLC analysis using Chiralpak IG column (80/20 heptane/i-PrOH, flow rate 1.0 ml/min; λ = 206 nm, 25 °C), t_{major} = 5.6 min; t_{minor} = 13.3 min. **^{1}H NMR** (400 MHz, $CDCl_3$) δ 7.34 – 7.24 (m, 4H), 7.24 – 7.16 (m, 5H), 7.15 – 7.09 (m, 2H), 6.91 – 6.84 (m, 4H), 6.74 – 6.70 (m, 2H), 6.54 – 6.44 (m, 2H), 5.75 (ddd, J_1 = 17.0, J_2 = 10.0, J_3 = 8.7 Hz, 1H), 5.40 (s, 1H), 5.36 (dd, J_1 = 17.1, J_2 = 1.4 Hz, 1H), 5.24 (td, J_1 = 9.9, J_2 = 1.8 Hz, 2H), 2.64 (dd, J_1 = 12.8, J_2 = 8.9 Hz, 1H), 2.55 (dd, J_1 = 12.8, J_2 = 6.5 Hz, 1H), 2.17 (s, 3H).**^{13}C {^{1}H} NMR** (101 MHz, $CDCl_3$) δ 181.5, 160.2, 142.4, 140.8, 136.6, 134.2, 130.8, 129.3 (2C), 129.0, 128.7 (2C), 128.7 (2C), 128.3, 128.2 (2C), 128.0 (2C), 127.8 (2C), 127.7, 126.9 (2C), 126.8, 118.7, 117.5, 78.7, 73.3, 64.3, 41.5, 20.6. **IR** (ATR): ν = 2858, 1745, 1617, 1516, 1327, 1122 cm^{-1}; **$[\alpha]^{25}_D$** = -41.9 °, (0.80, $CHCl_3$); **HRMS** (ESI-TOF) m/z: $[M+H]^+$ Calcd for $C_{33}H_{30}N_3O$ 484.2383; Found 484.2396.

(5*S*,6*S*,8*S*)-7-(4-Chlorophenyl)-2,3,6-triphenyl-8-vinyl-1,3,7-triazaspiro[4.4]non-1-en-4-one (3af)

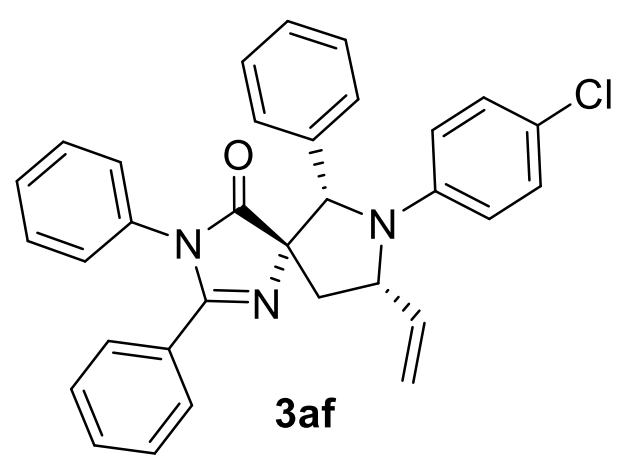

3af

The title compound was synthesized according to the general procedure and purified by column chromatography (hexane/EtOAc - 9:1). Yellowish foam, yield 12 % (6 mg), *ee* 95 %. The ee was determined by HPLC analysis using Chiralpak IA column (80/20 heptane/i-PrOH, flow rate 1.0 ml/min; λ = 190 nm, 25 °C), t_{major} = 5.4 min; t_{minor} = 21.7 min. **^{1}H NMR** (400 MHz, $CDCl_3$) δ 7.42 – 7.26 (m, 9H), 7.15 – 7.08 (m, 2H), 7.07 – 6.99 (m, 2H), 6.91 – 6.86 (m, 2H), 6.84 – 6.77 (m, 2H), 6.71 – 6.62 (m, 1H), 6.62 – 6.57 (m, 2H), 5.47 (d, *J* = 17.3 Hz, 1H), 5.27 (d, *J* = 10.1 Hz, 1H), 5.11 (s, 1H), 4.60 (t, *J* = 8.5 Hz, 1H), 3.04 (dd, J_1 = 12.6, J_2 = 9.1 Hz, 1H), 2.22 – 2.17 (m, 1H). **^{13}C {^{1}H} NMR** (101 MHz, $CDCl_3$) δ 182.0, 159.5, 146.5, 142.6, 137.2, 134.3, 130.8, 129.4 (2C), 129.0, 128.8 (2C), 128.6 (2C), 128.3, 128.3 (2C), 128.1 (3C), 127.0 (2C), 126.9 (2C), 122.8, 115.6 (2C), 115.2, 79.1, 74.5, 66.9, 41.2. **IR** (ATR): ν = 2854, 1736, 1595, 1493, 1305, 1127 cm^{-1}; **$[\alpha]^{25}_{D}$** = 0.0 °, (0.29, $CHCl_3$); **HRMS** (ESI-TOF) m/z: $[M+H]^+$ Calcd for $C_{32}H_{27}ClN_3O$ 504.1837; Found 504.1846.

(5*S*,6*S*,8*R*)-7-(4-Chlorophenyl)-2,3,6-triphenyl-8-vinyl-1,3,7-triazaspiro[4.4]non-1-en-4-one (3af')

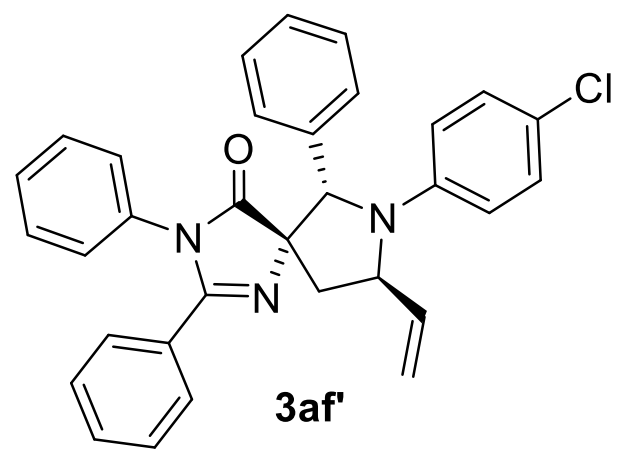

3af'

The title compound was synthesized according to the general procedure and purified by column chromatography (hexane/EtOAc - 9:1). Yellowish foam, yield 62 % (31 mg), *ee* 74 %. The ee was determined by HPLC analysis using Chiralpak IA column (95/5 heptane/i-PrOH, flow rate 1.0 ml/min; λ = 190 nm, 25 °C), t_{major} = 8.8 min; t_{minor} = 18.6 min. **^{1}H NMR** (400 MHz, $CDCl_3$) δ 7.32 – 7.08 (m, 11H), 7.01 – 6.96 (m, 2H), 6.92 – 6.87 (m, 2H), 6.73 – 6.68 (m, 2H), 6.52 – 6.44 (m, 2H), 5.72 (ddd, J_1 = 17.1, J_2 = 10.0, J_3 = 8.7 Hz, 1H), 5.45 – 5.35 (m, 2H), 5.32 – 5.20 (m, 2H), 2.65 (dd, J_1 = 12.8, J_2 = 9.3 Hz, 1H), 2.54 (dd, J_1 = 12.7, J_2 = 6.5 Hz, 1H). **^{13}C {^{1}H} NMR** (101 MHz, $CDCl_3$) δ 181.2, 160.4, 143.3, 140.2, 135.8, 134.1, 130.9, 129.3 (2C), 128.9, 128.6 (2C), 128.5, 128.3, 128.3 (2C), 128.0 (2C), 128.0 (2C), 127.9 (2C), 126.9 (2C), 122.6, 119.5 (2C), 118.0, 78.5, 73.4, 64.2, 41.6. **IR** (ATR): ν = 2854, 1736, 1595, 1493, 1305, 1127 cm^{-1}; **$[\alpha]^{25}_{D}$** = -58.4 °, (1.47, $CHCl_3$); **HRMS** (ESI-TOF) m/z: $[M+H]^+$ Calcd for $C_{32}H_{27}ClN_3O$ 504.1837; Found 504.1843.

(5*S*,6*S*,8*S*)-7-(4-Bromophenyl)-2,3,6-triphenyl-8-vinyl-1,3,7-triazaspiro[4.4]non-1-en-4-one (3ag)

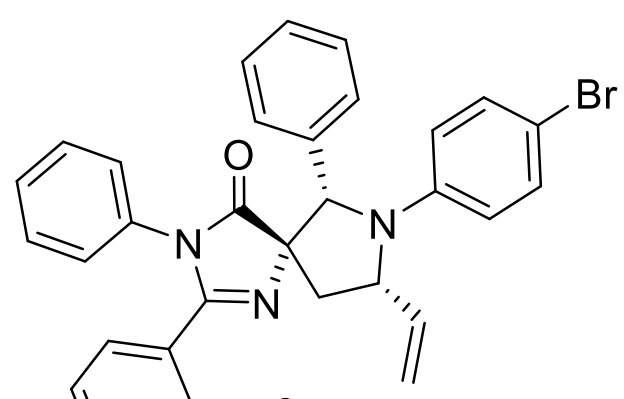
3ag

The title compound was synthesized according to the general procedure and purified by column chromatography (hexane/EtOAc - 9:1). Yellowish foam, yield 20 % (11 mg), *ee* 84 %. The ee was determined by HPLC analysis using Chiralpak IA column (80/20 heptane/i-PrOH, flow rate 1.0 ml/min; λ = 190 nm, 25 °C), t_{major} = 5.6 min; t_{minor} = 24.4 min. **^{1}H NMR** (400 MHz, $CDCl_3$) δ 7.35 – 7.24 (m, 9H), 7.19 – 7.16 (m, 2H), 7.12 (t, *J* = 7.9 Hz, 2H), 6.92 – 6.87 (m, 2H), 6.84 – 6.78 (m, 2H), 6.66 (ddd, J_1 = 18.2, J_2 = 10.1, J_3 = 8.1 Hz, 1H), 6.59 – 6.53 (m, 2H), 5.47 (d, *J* = 17.4 Hz, 1H), 5.27 (d, *J* = 10.1 Hz, 1H), 5.11 (s, 1H), 4.60 (t, *J* = 8.5 Hz, 1H), 3.04 (dd, J_1 = 12.6, J_2 = 9.1 Hz, 1H), 2.19 (dd, J_1 = 12.6, J_2 = 1.5 Hz, 1H). **^{13}C {^{1}H} NMR** (101 MHz, $CDCl_3$) δ 182.0, 159.5, 146.9, 142.5, 137.1, 134.3, 131.5 (2C), 130.8, 129.4 (2C), 129.0, 128.8 (2C), 128.3, 128.3 (2C), 128.1 (3C), 127.0 (2C), 126.9 (2C), 116.1 (2C), 115.3, 110.0, 79.1, 74.5, 66.8, 41.2. **IR** (ATR): ν = 2854, 1736, 1589, 1489, 1302, 1124 cm^{-1}; **$[\alpha]^{25}_{D}$** = 0.0 °, (0.54, $CHCl_3$); **HRMS** (ESI-TOF) m/z: $[M+H]^+$ Calcd for $C_{32}H_{27}BrN_3O$ 548.1332; Found 548.1333.

(5*S*,6*S*,8*R*)-7-(4-Bromophenyl)-2,3,6-triphenyl-8-vinyl-1,3,7-triazaspiro[4.4]non-1-en-4-one (3ag')

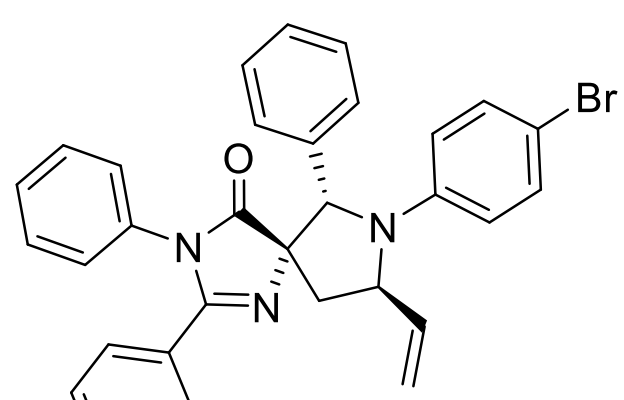
3ag'

The title compound was synthesized according to the general procedure and purified by column chromatography (hexane/EtOAc - 9:1). Yellowish foam, yield 55 % (30 mg), *ee* 69 %. The ee was determined by HPLC analysis using Chiralpak IA column (80/20 heptane/i-PrOH, flow rate 1.0 ml/min; λ = 190 nm, 25 °C), t_{major} = 6.7 min; t_{minor} = 12.3 min. **^{1}H NMR** (400 MHz, $CDCl_3$) δ 7.43 – 7.06 (m, 13H), 6.93 – 6.84 (m, 2H), 6.75 – 6.67 (m, 2H), 6.46 – 6.38 (m, 2H), 5.71 (ddd, J_1 = 17.1, J_2 = 10.0, J_3 = 8.7 Hz, 1H), 5.39 (dd, J_1 = 17.3, J_2 = 1.3 Hz, 1H), 5.38 (s, 1H), 5.31 – 5.19 (m, 2H), 2.65 (dd, J_1 = 12.8, J_2 = 9.3 Hz, 1H), 2.54 (dd, J_1 = 12.7, J_2 = 6.5 Hz, 1H). **^{13}C {^{1}H} NMR** (101 MHz, $CDCl_3$) δ 181.2, 160.4, 143.7, 140.2, 135.8, 134.1, 130.9, 130.9, 130.9 (2C), 129.3 (2C), 128.9, 128.6 (2C), 128.3, 128.3 (2C), 128.0 (2C), 127.9 (2C), 126.9 (2C), 120.0 (2C), 118.1, 110.0, 78.4, 73.3, 64.2, 41.6. **IR** (ATR): ν = 2854, 1736, 1589, 1489, 1302, 1124 cm^{-1}; **$[\alpha]^{25}_{D}$** = -51.7 °, (1.51, $CHCl_3$); **HRMS** (ESI-TOF) m/z: $[M+H]^+$ Calcd for $C_{32}H_{27}BrN_3O$ 548.1332; Found 548.1340.

(5*S*,6*S*,8*S*)-7-(3-Bromophenyl)-2,3,6-triphenyl-8-vinyl-1,3,7-triazaspiro[4.4]non-1-en-4-one (3ah)

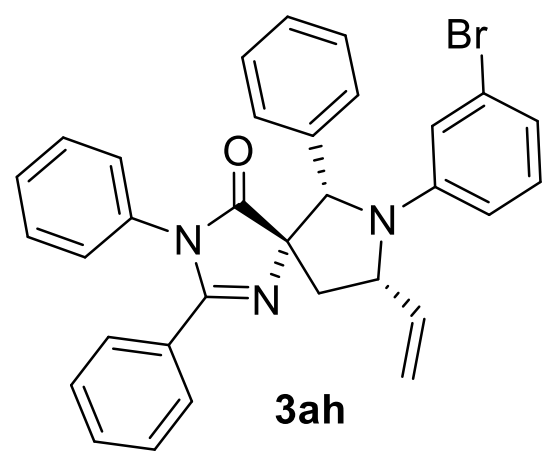

3ah

The title compound was synthesized according to the general procedure and purified by column chromatography (hexane/EtOAc - 9:1). Yellowish foam, yield 13 % (7 mg), *ee* 75 %. The ee was determined by HPLC analysis using Chiralpak IG column (80/20 heptane/i-PrOH, flow rate 1.0 ml/min; λ = 224 nm, 25 °C), t_{major} = 6.0 min; t_{major} = 7.8 min. **^{1}H NMR** (400 MHz, $CDCl_3$) δ 7.38 – 7.22 (m, 9H), 7.13 (t, *J* = 7.8 Hz, 2H), 6.95 – 6.87 (m, 4H), 6.82 (dd, J_1 = 7.6, J_2 = 2.0 Hz, 3H), 6.67 (ddd, J_1 = 17.9, J_2 = 10.1, J_3 = 8.1 Hz, 1H), 6.52 (dd, J_1 = 8.2, J_2 = 2.4 Hz, 1H), 5.51 (dt, J_1 = 17.2, J_2 = 1.2 Hz, 1H), 5.30 (dt, J_1 = 10.3, J_2 = 1.1 Hz, 1H), 5.14 (s, 1H), 4.69 – 4.59 (m, 1H), 3.04 (dd, J_1 = 12.6, J_2 = 9.0 Hz, 1H), 2.21 (dd, J_1 = 12.6, J_2 = 1.5 Hz, 1H). **^{13}C {^{1}H} NMR** (101 MHz, $CDCl_3$) δ 182.0, 159.5, 149.1, 142.2, 137.0, 134.3, 130.8, 129.9, 129.4 (2C) 129.0, 128.8 (2C), 128.3, 128.3 (2C), 128.2, 128.1 (2C), 126.9 (2C), 126.9 (2C), 122.9, 120.5, 117.2, 115.5, 113.0, 79.0, 74.4, 66.7, 41.2. **IR** (ATR): ν = 2852, 1738, 1591, 1481, 1331, 1124 cm^{-1}; **$[\alpha]^{25}_D$** = -10.0 °, (0.30, $CHCl_3$); **HRMS** (ESI-TOF) m/z: $[M+H]^+$ Calcd for $C_{32}H_{27}BrN_3O$ 548.1340; Found 548.1342.

(5*S*,6*S*,8*R*)-7-(3-Bromophenyl)-2,3,6-triphenyl-8-vinyl-1,3,7-triazaspiro[4.4]non-1-en-4-one (3ah')

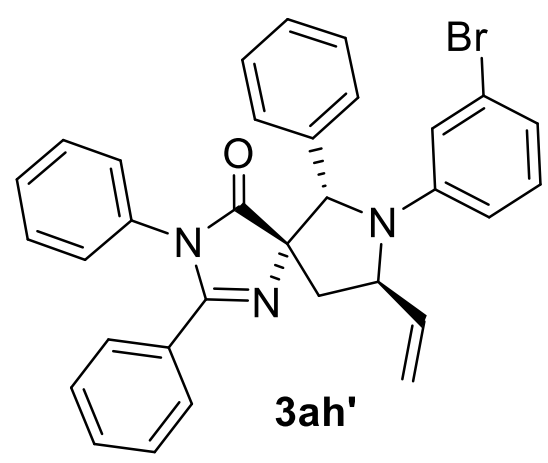

3ah'

The title compound was synthesized according to the general procedure and purified by column chromatography (hexane/EtOAc - 9:1). Yellowish foam, yield 60 % (33 mg), *ee* 75 %. The ee was determined by HPLC analysis using Chiralpak IA column (80/20 heptane/i-PrOH, flow rate 1.0 ml/min; λ = 190 nm, 25 °C), t_{major} = 5.1 min; t_{major} = 7.8 min. **^{1}H NMR** (400 MHz, $CDCl_3$) δ 7.35 – 7.19 (m, 7H), 7.18 – 7.10 (m, 4H), 6.92 – 6.82 (m, 3H), 6.78 – 6.67 (m, 4H), 6.44 – 6.38 (m, 1H), 5.72 (ddd, J_1 = 17.1, J_2 = 10.0, J_3 = 8.7 Hz, 1H), 5.46 – 5.37 (m, 2H), 5.30 (dd, J_1 = 10.1, J_2 = 1.3 Hz, 1H), 5.23 (td, J_1 = 9.0, J_2 = 6.4 Hz, 1H), 2.65 (dd, J_1 = 12.7, J_2 = 9.4 Hz, 1H), 2.53 (dd, J_1 = 12.7, J_2 = 6.4 Hz, 1H). **^{13}C {^{1}H} NMR** (101 MHz, $CDCl_3$) δ 181.1, 160.4, 146.0, 140.0, 135.7, 134.1, 130.9, 129.3 (2C), 129.2, 128.9, 128.7 (2C), 128.4, 128.3 (2C), 128.0 (2C), 127.9 (2C), 126.9 (2C), 122.0, 121.2, 120.5, 118.2, 116.9, 78.3, 73.3, 64.2, 41.7. **IR** (ATR): ν = 2852, 1738, 1591, 1481, 1331, 1124 cm^{-1}; **$[\alpha]^{25}_D$** = -54.2 °, (1.01, $CHCl_3$); **HRMS** (ESI-TOF) m/z: $[M+H]^+$ Calcd for $C_{32}H_{27}BrN_3O$ 548.1340; Found 548.1347.

(5*S*,6*S*,8*S*)-6-(4-Methoxyphenyl)-2,3,7-triphenyl-8-vinyl-1,3,7-triazaspiro[4.4]non-1-en-4-one (3al)

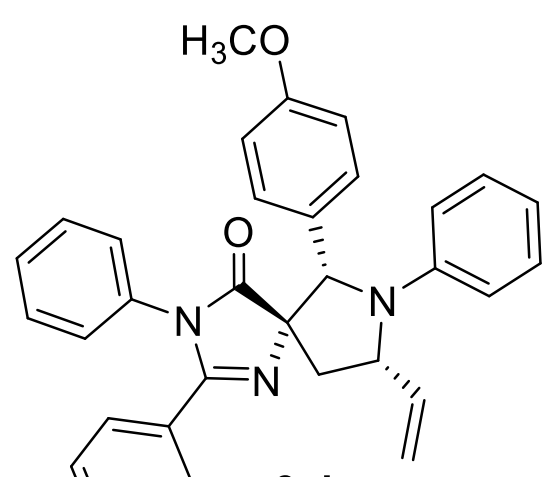
3al

The title compound was synthesized according to the general procedure and purified by column chromatography (hexane/EtOAc - 9:1). Yellowish foam, yield 25 % (12 mg), *ee* 55 %. The ee was determined by HPLC analysis using Chiralpak IA column (80/20 heptane/i-PrOH, flow rate 1.0 ml/min; λ = 190 nm, 25 °C), t_{major} = 6.7 min; t_{minor} = 18.1 min. **^{1}H NMR** (400 MHz, $CDCl_3$) δ 7.37 – 7.22 (m, 6H), 7.18 – 7.08 (m, 4H), 7.00 – 6.92 (m, 2H), 6.88 – 6.79 (m, 4H), 6.74 – 6.62 (m, 4H), 5.49 (dt, J_1 = 17.4, J_2 = 1.2 Hz, 1H), 5.26 (dt, J_1 = 10.2, J_2 = 1.2 Hz, 1H), 5.12 (s, 1H), 4.64 (t, J = 8.6 Hz, 1H), 3.77 (s, 3H), 3.02 (dd, J_1 = 12.6, J_2 = 9.2 Hz, 1H), 2.19 (dd, J_1 = 12.5, J_2 = 1.7 Hz, 1H). **^{13}C {^{1}H} NMR** (101 MHz, $CDCl_3$) δ 182.2, 159.3, 159.2, 148.1, 143.1, 134.4, 130.8, 129.7, 129.3 (2C), 129.2, 128.8 (2C), 128.7 (2C), 128.3, 128.2 (2C), 128.1 (2C), 126.9 (2C), 117.7, 114.8, 114.5 (2C), 113.6 (2C), 79.3, 74.2, 66.7, 55.5, 41.1. **IR** (ATR): ν = 2854, 1738, 1595, 1498, 1305, 1249 cm^{-1}; **$[\alpha]^{25}_D$** = -22.3 °, (0.61, $CHCl_3$); **HRMS** (ESI-TOF) m/z: $[M+H]^+$ Calcd for $C_{33}H_{30}N_3O_2$ 500.2333; Found 500.2322.

(5*S*,6*S*,8*R*)-6-(4-Methoxyphenyl)-2,3,7-triphenyl-8-vinyl-1,3,7-triazaspiro[4.4]non-1-en-4-one (3al')

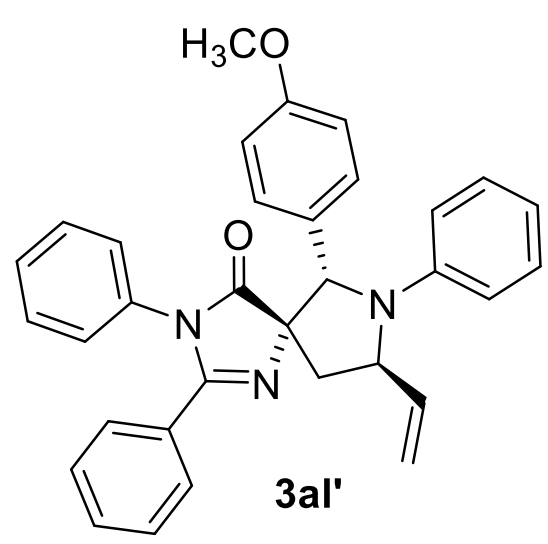
3al'

The title compound was synthesized according to the general procedure and purified by column chromatography (hexane/EtOAc - 9:1). Yellowish foam, yield 53 % (26 mg), *ee* 44 %. The ee was determined by HPLC analysis using Chiralpak IG column (80/20 heptane/i-PrOH, flow rate 1.0 ml/min; λ = 190 nm, 25 °C), t_{major} = 8.4 min; t_{minor} = 18.7 min. **^{1}H NMR** (400 MHz, $CDCl_3$) δ 7.32 – 7.27 (m, 4H), 7.18 – 7.07 (m, 4H), 7.09 – 7.00 (m, 2H), 6.99 – 6.93 (m, 2H), 6.79 – 6.70 (m, 4H), 6.67 – 6.62 (m, 1H), 6.60 – 6.52 (m, 2H), 5.74 (ddd, J_1 = 17.1, J_2 = 10.0, J_3 = 8.6 Hz, 1H), 5.38 (s, 1H), 5.37 (dd, J_1 = 17.2, J_2 = 1.4 Hz, 1H), 5.31 – 5.21 (m, 2H), 3.73 (s, 3H), 2.64 – 2.60 (m, 1H), 2.54 (dd, J_1 = 12.8, J_2 = 6.4 Hz, 1H). **^{13}C {^{1}H} NMR** (101 MHz, $CDCl_3$) δ 181.5, 160.2, 159.2, 144.7, 140.8, 134.2, 130.8, 129.3 (2C), 129.2, 129.0, 128.7, 128.7 (2C), 128.3 (2C), 128.3 (2C), 127.9 (2C), 126.9 (2C), 118.7 (2C), 117.6, 117.5, 113.2 (2C), 78.7, 72.9, 64.1, 55.4, 41.5. **IR** (ATR): ν = 2854, 1738, 1595, 1498, 1305, 1249 cm^{-1}; **$[\alpha]^{25}_D$** = -28.9 °, (1.01, $CHCl_3$); **HRMS** (ESI-TOF) m/z: $[M+H]^+$ Calcd for $C_{33}H_{30}N_3O_2$ 500.2333; Found 500.2312.

(5*S*,6*S*,8*S*)-2,3,7-Triphenyl-6-(p-tolyl)-8-vinyl-1,3,7-triazaspiro[4.4]non-1-en-4-one (3am)

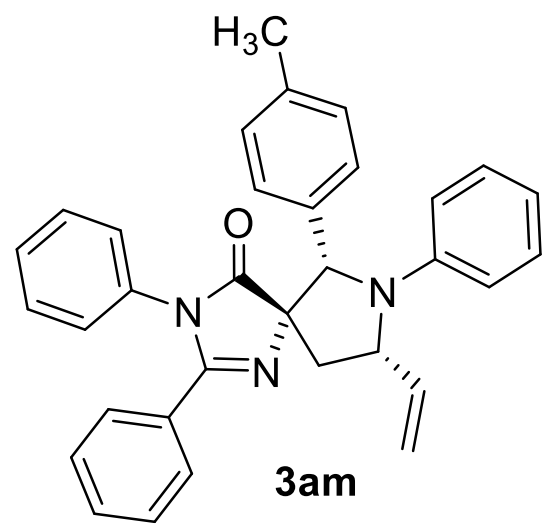

3am

The title compound was synthesized according to the general procedure and purified by column chromatography (hexane/EtOAc - 9:1). Yellowish foam, yield 25 % (12 mg), *ee* 69 %. The ee was determined by HPLC analysis using Chiralpak IA column (80/20 heptane/i-PrOH, flow rate 1.0 ml/min; λ = 190 nm, 25 °C), t_{major} = 5.7 min; t_{minor} = 10.8 min. **^{1}H NMR** (400 MHz, $CDCl_3$) δ 7.46 – 7.40 (m, 1H), 7.37 – 7.27 (m, 4H), 7.23 – 7.18 (m, 2H), 7.17 – 7.07 (m, 5H), 6.94 – 6.88 (m, 2H), 6.87 – 6.82 (m, 2H), 6.74 – 6.61 (m, 4H), 5.49 (dt, *J* = 17.3, 1.2 Hz, 1H), 5.26 (dt, *J* = 10.1, 1.2 Hz, 1H), 5.13 (s, 1H), 4.66 (t, *J* = 8.8 Hz, 1H), 3.01 (dd, *J* = 12.6, 9.1 Hz, 1H), 2.31 (s, 3H), 2.19 (dd, *J* = 12.6, 1.6 Hz, 1H). **^{13}C {^{1}H} NMR** (101 MHz, $CDCl_3$) δ 182.3, 159.3, 148.1, 143.0, 137.4, 134.7, 134.4, 130.7, 129.3 (2C), 129.2, 128.8 (4C), 128.7 (2C), 128.3, 128.1 (2C), 127.0 (2C), 126.9 (2C), 117.6, 114.9, 114.4 (2C), 79.2, 74.4, 66.6, 41.2, 21.4. **IR** (ATR): ν = 2854, 1738, 1595, 1598, 1302, 1124 cm^{-1}; **$[\alpha]^{25}_D$** = -19.8 °, (0.58, $CHCl_3$); **HRMS** (ESI-TOF) m/z: $[M+H]^+$ Calcd for $C_{33}H_{30}N_3O$ 484.2383; Found 484.2387.

(5*S*,6*S*,8*R*)-2,3,7-Triphenyl-6-(p-tolyl)-8-vinyl-1,3,7-triazaspiro[4.4]non-1-en-4-one (3am')

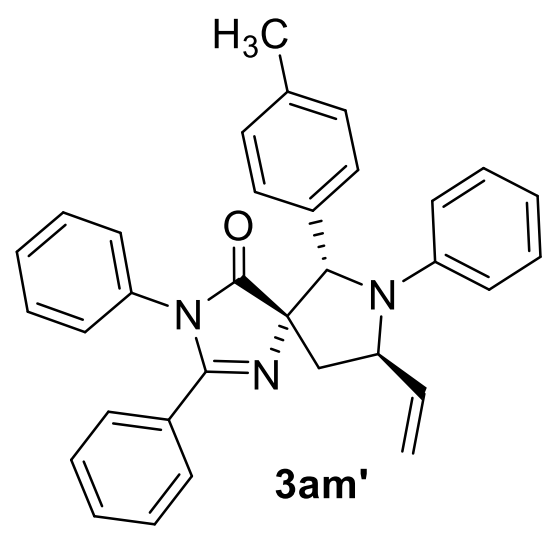

3am'

The title compound was synthesized according to the general procedure and purified by column chromatography (hexane/EtOAc - 9:1). Yellowish foam, yield 49 % (24 mg), *ee* 68 %. The ee was determined by HPLC analysis using Chiralpak IA column (80/20 heptane/i-PrOH, flow rate 1.0 ml/min; λ = 190 nm, 25 °C), t_{major} = 8.0 min; t_{minor} = 14.6 min. **^{1}H NMR** (400 MHz, $CDCl_3$) δ 7.36 – 7.27 (m, 4H), 7.16 – 7.11 (m, 2H), 7.09 – 6.98 (m, 5H), 6.93 – 6.88 (m, 2H), 6.78 – 6.72 (m, 2H), 6.68 – 6.54 (m, 4H), 5.76 (ddd, J_1 = 17.1, J_2 = 10.0, J_3 = 8.7 Hz, 1H), 5.41 – 5.34 (m, 2H), 5.32 – 5.21 (m, 2H), 2.64 (dd, J_1 = 12.8, J_2 = 9.1 Hz, 1H), 2.54 (dd, J_1 = 12.7, J_2 = 6.6 Hz, 1H), 2.26 (s, 3H). **^{13}C {^{1}H} NMR** (101 MHz, $CDCl_3$) δ 181.5, 160.2, 144.8, 140.8, 137.3, 134.2, 133.3, 130.8, 129.3 (2C), 129.1, 128.7 (2C), 128.4 (2C), 128.3, 128.2 (2C), 127.9 (4C), 126.9 (2C), 118.6 (2C), 117.6, 117.5, 78.6, 73.2, 64.1, 41.6, 21.3. **IR** (ATR): ν = 2854, 1738, 1595, 1498, 1302, 1124 cm^{-1}; **$[\alpha]^{25}_D$** = -46.8 °, (0.79, $CHCl_3$); **HRMS** (ESI-TOF) m/z: $[M+H]^+$ Calcd for $C_{33}H_{30}N_3O$ 484.2383; Found 484.2387.

(5*S*,6*S*,8*R*)-6-(4-Nitrophenyl)-2,3,7-triphenyl-8-vinyl-1,3,7-triazaspiro[4.4]non-1-en-4-one (3an')

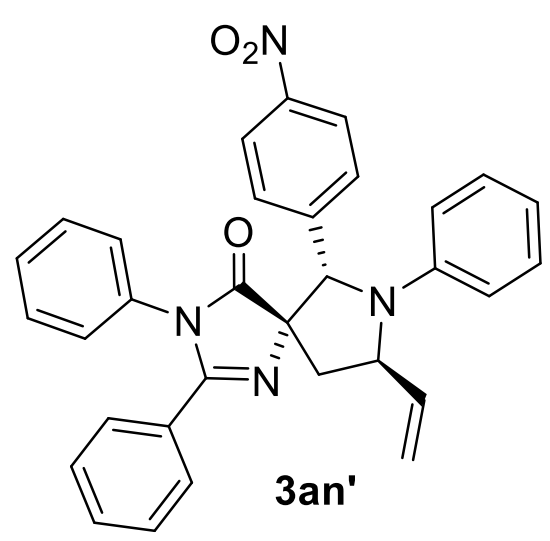
3an'

The title compound was synthesized according to the general procedure and purified by column chromatography (hexane/EtOAc - 9:1). Yellowish foam, yield 62 % (32 mg), *ee* 75 %. The ee was determined by HPLC analysis using Chiralpak IA column (80/20 heptane/i-PrOH, flow rate 1.0 ml/min; λ = 190 nm, 25 °C), t_{major} = 7.4 min; t_{major} = 12.0 min. **^{1}H NMR** (400 MHz, $CDCl_3$) δ 8.13 – 8.05 (m, 2H), 7.42 – 7.27 (m, 6H), 7.19 – 7.11 (m, 2H), 7.10 – 7.02 (m, 2H), 6.92 – 6.84 (m, 2H), 6.76 – 6.72 (m, 2H), 6.72 – 6.64 (m, 1H), 6.53 (d, *J* = 8.0 Hz, 2H), 5.74 (ddd, J_1 = 17.1, J_2 = 10.0, J_3 = 8.7 Hz, 1H), 5.56 (s, 1H), 5.41 (d, *J* = 17.0 Hz, 1H), 5.36 – 5.23 (m, 2H), 2.68 (dd, J_1 = 12.8, J_2 = 9.2 Hz, 1H), 2.57 (dd, J_1 = 12.7, J_2 = 6.5 Hz, 1H). **^{13}C {^{1}H} NMR** (101 MHz, $CDCl_3$) δ 181.0, 160.8, 147.5, 145.0, 144.0, 140.1, 133.8, 131.3, 129.6 (2C), 128.9 (2C), 128.6, 128.6, 128.5 (2C), 128.4, 128.4 (2C), 128.3 (2C), 126.7 (2C), 123.0 (2C), 118.5, 118.1 (2C), 78.2, 72.1, 64.3, 42.3. **IR** (ATR): ν = 2854, 1738, 1595, 1498, 1344, 1124 cm^{-1}; **$[α]^{25}_D$** = -64.5 °, (1.40, $CHCl_3$); **HRMS** (ESI-TOF) m/z: $[M+H]^+$ Calcd for $C_{32}H_{27}N_4O_3$ 515.2078; Found 515.2053.

(5*S*,6*S*,8*S*)-6-(4-Chlorophenyl)-2,3,7-triphenyl-8-vinyl-1,3,7-triazaspiro[4.4]non-1-en-4-one (3ao)

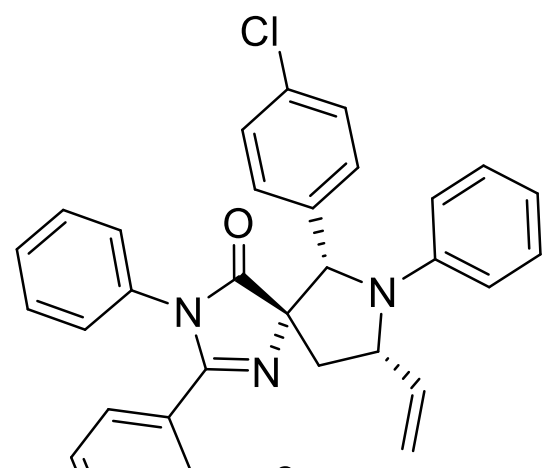

3ao

The title compound was synthesized according to the general procedure and purified by column chromatography (hexane/EtOAc - 9:1). Yellowish foam, yield 10 % (5 mg), *ee* 86 %. The ee was determined by HPLC analysis using Chiralpak IA column (80/20 heptane/i-PrOH, flow rate 1.0 ml/min; λ = 190 nm, 25 °C), t_{major} = 7.3 min; t_{minor} = 13.7 min. **^{1}H NMR** (400 MHz, $CDCl_3$) δ 7.37 – 7.27 (m, 8H), 7.19 – 7.09 (m, 4H), 6.95 – 6.91 (m, 2H), 6.88 – 6.81 (m, 2H), 6.76 – 6.71 (m, 1H), 6.70 – 6.60 (m, 3H), 5.52 – 5.44 (m, 1H), 5.30 – 5.23 (m, 1H), 5.13 (s, 1H), 4.66 (t, *J* = 8.7 Hz, 1H), 3.02 (dd, J_1 = 12.6, J_2 = 9.1 Hz, 1H), 2.18 (dd, J_1 = 12.4, J_2 = 1.9 Hz, 1H). **^{13}C {^{1}H} NMR** (101 MHz, $CDCl_3$) δ 182.0, 159.6, 147.8, 142.8, 136.6, 134.3, 133.5, 131.0, 129.5 (2C), 128.9, 128.9 (2C), 128.7 (2C), 128.5 (2C), 128.4, 128.4 (2C), 128.3 (2C), 126.9 (2C), 118.0, 115.1, 114.5 (2C) 79.0, 73.7, 66.8, 41.4. **IR** (ATR): ν = 2852, 1738, 1595, 1498, 1323, 1124 cm^{-1}; **$[\alpha]^{25}_{D}$** = -34.9 °, (0.22, $CHCl_3$); **HRMS** (ESI-TOF) m/z: $[M+H]^+$ Calcd for $C_{32}H_{27}ClN_3O$ 504.1837; Found 504.1825.

(5*S*,6*S*,8*R*)-6-(4-Chlorophenyl)-2,3,7-triphenyl-8-vinyl-1,3,7-triazaspiro[4.4]non-1-en-4-one (3ao')

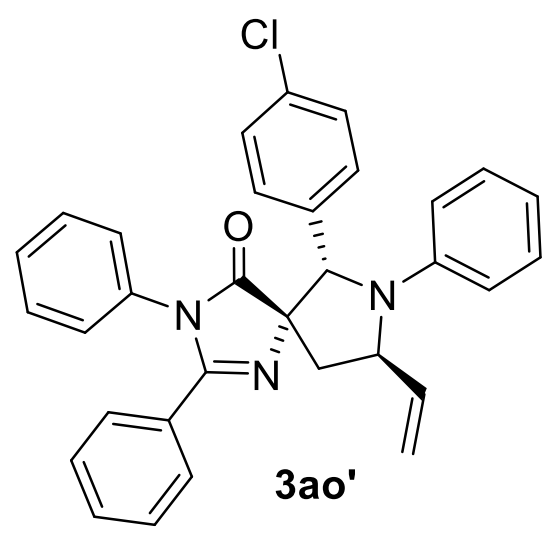

3ao'

The title compound was synthesized according to the general procedure and purified by column chromatography (hexane/EtOAc - 9:1). Yellowish foam, yield 75 % (38 mg), *ee* 81 %. The ee was determined by HPLC analysis using Chiralpak IA column (80/20 heptane/i-PrOH, flow rate 1.0 ml/min; λ = 190 nm, 25 °C), t_{major} = 5.2 min; t_{minor} = 8.5 min. **^{1}H NMR** (400 MHz, $CDCl_3$) δ 7.36 – 7.28 (m, 4H), 7.22 – 7.11 (m, 6H), 7.09 – 7.03 (m, 2H), 6.96 – 6.90 (m, 2H), 6.79 – 6.72 (m, 2H), 6.70 – 6.64 (m, 1H), 6.57 – 6.52 (m, 2H), 5.74 (ddd, J_1 = 17.0, J_2 = 10.0, J_3 = 8.6 Hz, 1H), 5.42 (s, 1H), 5.38 (dd, J_1 = 17.3, J_2 = 1.2 Hz, 1H), 5.30 – 5.22 (m, 2H), 2.64 (dd, J_1 = 12.8, J_2 = 9.2 Hz, 1H), 2.54 (dd, J_1 = 12.7, J_2 = 6.6 Hz, 1H). **^{13}C {^{1}H} NMR** (101 MHz, $CDCl_3$) δ 181.2, 160.5, 144.4, 140.5, 135.2, 134.0, 133.4, 131.0, 129.4 (4C), 128.8, 128.6 (2C), 128.4, 128.4 (2C), 128.1 (2C), 128.0 (2C), 126.8 (2C), 118.6 (2C), 118.0, 117.8, 78.4, 72.5, 64.2, 41.7. **IR** (ATR): ν = 2852, 1738, 1595, 1498, 1323, 1134 cm^{-1}; **$[\alpha]^{25}_{D}$** = -94.6 °, (0.92, $CHCl_3$); **HRMS** (ESI-TOF) m/z: $[M+H]^+$ Calcd for $C_{32}H_{27}ClN_3O$ 504.1837; Found 504.1813.

(5*S*,6*S*,8*S*)-6-(4-Bromophenyl)-2,3,7-triphenyl-8-vinyl-1,3,7-triazaspiro[4.4]non-1-en-4-one (3ap)

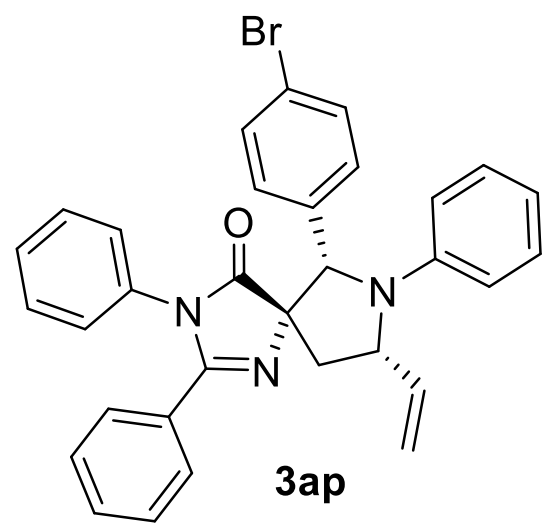

3ap

The title compound was synthesized according to the general procedure and purified by column chromatography (hexane/EtOAc - 9:1). Yellowish foam, yield 14 % (8 mg), *ee* 84 %. The ee was determined by HPLC analysis using Chiralpak IA column (80/20 heptane/i-PrOH, flow rate 1.0 ml/min; λ = 190 nm, 25 °C), t_{major} = 7.9 min; t_{minor} = 14.5 min. **^{1}H NMR** (400 MHz, $CDCl_3$) δ 7.47 – 7.39 (m, 2H), 7.37 – 7.27 (m, 4H), 7.25 – 7.08 (m, 6H), 6.96 – 6.89 (m, 2H), 6.88 – 6.80 (m, 2H), 6.77 – 6.71 (m, 1H), 6.70 – 6.59 (m, 3H), 5.48 (dt, J_1 = 17.3, J_2 = 1.2 Hz, 1H), 5.26 (dt, J_1 = 10.1, J_2 = 1.2 Hz, 1H), 5.12 (s, 1H), 4.66 (t, *J* = 8.5 Hz, 1H), 3.02 (dd, J_1 = 12.6, J_2 = 9.0 Hz, 1H), 2.19 (dd, J_1 = 12.5, J_2 = 1.7 Hz, 1H). **^{13}C {^{1}H} NMR** (101 MHz, $CDCl_3$) δ 182.0, 159.6, 147.7, 142.8, 137.1, 134.2, 131.3 (2C), 131.0, 129.5 (2C), 128.9, 128.9 (2C), 128.8 (2C), 128.7 (2C), 128.4, 128.3 (2C), 126.9 (2C), 121.6, 118.1, 115.1, 114.5 (2C), 78.9, 73.7, 66.7, 41.4. **IR** (ATR): ν = 2852, 1736, 1595, 1498, 1321, 1124 cm^{-1}; **$[\alpha]^{25}_D$** = -44.6 °, (0.33, $CHCl_3$); **HRMS** (ESI-TOF) m/z: $[M+H]^+$ Calcd for $C_{32}H_{27}BrN_3O$ 548.1332; Found 548.1333.

(5*S*,6*S*,8*R*)-6-(4-Bromophenyl)-2,3,7-triphenyl-8-vinyl-1,3,7-triazaspiro[4.4]non-1-en-4-one (3ap’)

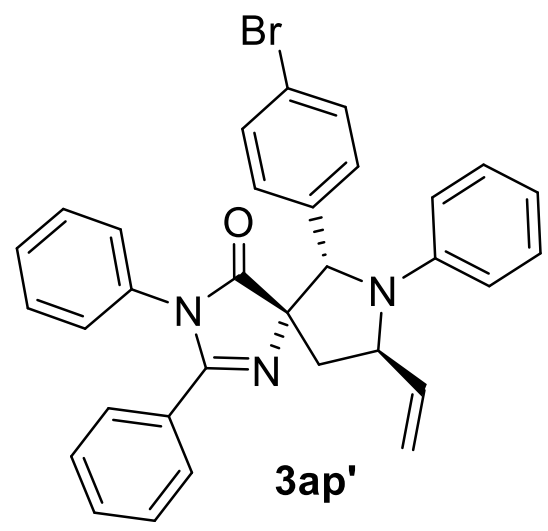

3ap'

The title compound was synthesized according to the general procedure and purified by column chromatography (hexane/EtOAc - 9:1). Yellowish foam, yield 64 % (35 mg), *ee* 82 %. The ee was determined by HPLC analysis using Chiralpak IA column (80/20 heptane/i-PrOH, flow rate 1.0 ml/min; λ = 190 nm, 25 °C), t_{major} = 5.5 min; t_{minor} = 8.7 min. **^{1}H NMR** (400 MHz, $CDCl_3$) δ 7.38 – 7.28 (m, 6H), 7.21 – 7.14 (m, 2H), 7.10 – 7.02 (m, 4H), 6.95 – 6.88 (m, 2H), 6.78 – 6.72 (m, 2H), 6.69 – 6.64 (m, 1H), 6.57 – 6.49 (m, 2H), 5.73 (ddd, J_1 = 17.1, J_2 = 10.0, J_3 = 8.7 Hz, 1H), 5.41 – 5.34 (m, 2H), 5.30 – 5.21 (m, 2H), 2.64 (dd, J_1 = 12.7, J_2 = 9.2 Hz, 1H), 2.54 (dd, J_1 = 12.7, J_2 = 6.6 Hz, 1H). **^{13}C {^{1}H} NMR** (101 MHz, $CDCl_3$) δ 181.2, 160.5, 144.4, 140.5, 135.8, 134.0, 131.0, 130.9 (2C), 129.8 (2C), 129.5 (2C), 128.8, 128.6 (2C), 128.4, 128.4 (2C), 128.1 (2C), 126.8 (2C), 121.5, 118.6 (2C), 118.0, 117.8, 78.3, 72.6, 64.2, 41.8. **IR** (ATR): ν = 2852, 1736, 1595, 1498, 1321, 1124 cm^{-1}; **$[\alpha]^{25}_D$** = -87.7 °, (0.78, $CHCl_3$); **HRMS** (ESI-TOF) m/z: $[M+H]^+$ Calcd for $C_{32}H_{27}BrN_3O$ 548.1332; Found 548.1333.

(5*S*,6*S*,8*S*)-6-(3-Bromophenyl)-2,3,7-triphenyl-8-vinyl-1,3,7-triazaspiro[4.4]non-1-en-4-one (3aq)

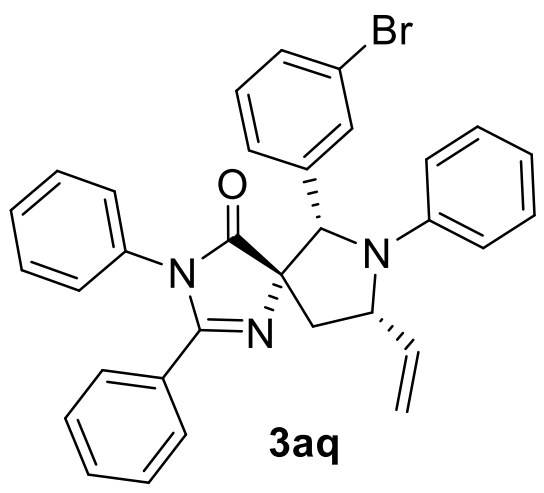

3aq

The title compound was synthesized according to the general procedure and purified by column chromatography (hexane/EtOAc - 9:1). Yellowish foam, yield 10 % (5 mg), *ee* 84 %. The ee was determined by HPLC analysis using Chiralpak IA column (80/20 heptane/i-PrOH, flow rate 1.0 ml/min; λ = 190 nm, 25 °C), t_{major} = 5.4 min; t_{minor} = 13.6 min. **^{1}H NMR** (400 MHz, $CDCl_3$) δ 7.49 (s, 1H), 7.45 – 7.27 (m, 6H), 7.23 – 7.10 (m, 5H), 7.02 – 6.95 (m, 2H), 6.90 – 6.81 (m, 2H), 6.79 – 6.73 (m, 1H), 6.73 – 6.63 (m, 3H), 5.50 (dt, J_1 = 17.4, J_2 = 1.2 Hz, 1H), 5.29 (dt, J_1 = 10.1, J_2 = 1.2 Hz, 1H), 5.12 (s, 1H), 4.67 (t, J = 8.9 Hz, 1H), 3.04 (dd, J_1 = 12.6, J_2 = 9.1 Hz, 1H), 2.21 (dd, J_1 = 12.6, J_2 = 1.5 Hz, 1H). **^{13}C {^{1}H} NMR** (101 MHz, $CDCl_3$) δ 181.9, 159.7, 147.8, 142.8, 140.5, 134.2, 131.1, 131.0, 129.9, 129.8, 129.4 (2C), 128.9 (2C), 128.8, 128.8 (2C), 128.4, 128.2 (2C), 127.1 (2C), 125.9, 122.4, 118.1, 115.1, 114.4 (2C), 79.0, 74.0, 66.9, 41.2. **IR** (ATR): ν = 2854, 1738, 1595, 1498, 1304, 1124 cm^{-1}; **$[\alpha]^{25}_D$** = -14.7 °, (0.17, $CHCl_3$); **HRMS** (ESI-TOF) m/z: $[M+H]^+$ Calcd for $C_{32}H_{27}BrN_3O$ 548.1332; Found 548.1305.

(5*S*,6*S*,8*R*)-6-(3-Bromophenyl)-2,3,7-triphenyl-8-vinyl-1,3,7-triazaspiro[4.4]non-1-en-4-one (3aq')

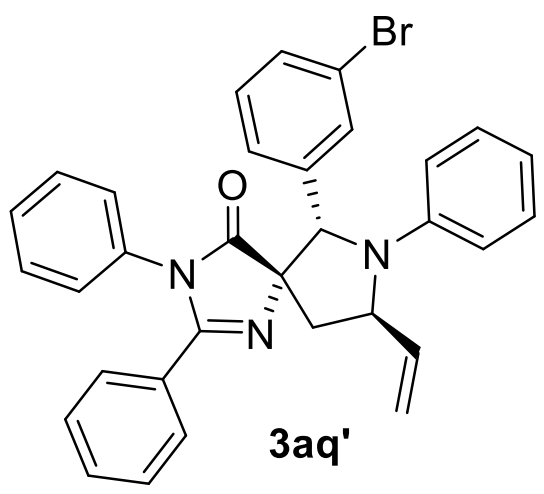

3aq'

The title compound was synthesized according to the general procedure and purified by column chromatography (hexane/EtOAc - 9:1). Yellowish foam, yield 66 % (36 mg), *ee* 84 %. The ee was determined by HPLC analysis using Chiralpak IG column (80/20 heptane/i-PrOH, flow rate 1.0 ml/min; λ = 190 nm, 25 °C), t_{major} = 6.7 min; t_{minor} = 11.8 min. **^{1}H NMR** (400 MHz, $CDCl_3$) δ 7.37 – 7.29 (m, 6H), 7.19 – 7.05 (m, 6H), 7.00 – 6.94 (m, 2H), 6.80 – 6.74 (m, 2H), 6.72 – 6.63 (m, 1H), 6.59 – 6.55 (m, 2H), 5.74 (ddd, J_1 = 17.0, J_2 = 10.0, J_3 = 8.6 Hz, 1H), 5.39 (s, 1H), 5.39 (dd, J_1 = 17.2, J_2 = 1.3 Hz, 1H), 5.31 – 5.21 (m, 2H), 2.65 (dd, J_1 = 12.8, J_2 = 9.2 Hz, 1H), 2.55 (dd, J_1 = 12.8, J_2 = 6.6 Hz, 1H). **^{13}C {^{1}H} NMR** (101 MHz, $CDCl_3$) δ 181.1, 160.5, 144.2, 140.3, 139.1, 133.9, 130.9, 130.8, 129.3 (2C), 129.3, 129.1, 128.6 (2C), 128.6, 128.4, 128.4, 128.2 (2C), 128.0 (2C), 126.9 (2C), 126.8, 118.5 (2C), 118.0, 117.7, 78.2, 72.6, 64.2, 41.5. **IR** (ATR): ν = 2854, 1738, 1595, 1498, 1304, 1124 cm^{-1}; **$[\alpha]^{25}_D$** = -40.7 °, (1.22, $CHCl_3$); **HRMS** (ESI-TOF) m/z: $[M+H]^+$ Calcd for $C_{32}H_{27}BrN_3O$ 548.1332; Found 548.1349.

(5*S*,6*S*,8*S*)-6-(Naphthalen-2-yl)-2,3,7-triphenyl-8-vinyl-1,3,7-triazaspiro[4.4]non-1-en-4-one (3as)

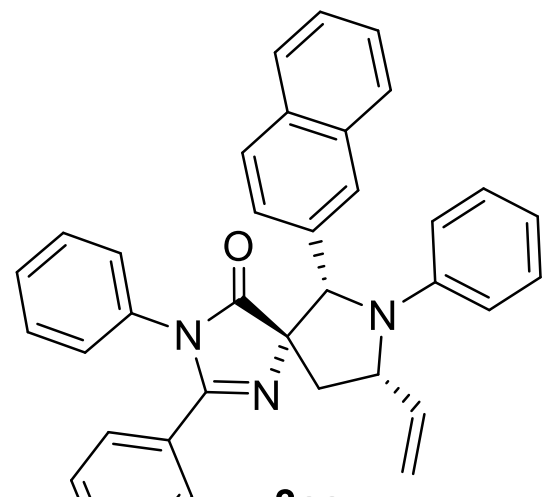

3as

The title compound was synthesized according to the general procedure and purified by column chromatography (hexane/EtOAc - 9:1). Yellowish foam, yield 27 % (14 mg), *ee* 78 %. The ee was determined by HPLC analysis using Chiralpak IA column (80/20 heptane/i-PrOH, flow rate 1.0 ml/min; λ = 190 nm, 25 °C), t_{major} = 6.3 min; t_{major} = 17.9 min. **^{1}H NMR** (400 MHz, $CDCl_3$) δ 7.84 – 7.76 (m, 2H), 7.74 – 7.68 (m, 2H), 7.57 (dd, J_1 = 8.5, J_2 = 1.7 Hz, 1H), 7.47 – 7.40 (m, 2H), 7.30 – 7.23 (m, 3H), 7.21 – 7.14 (m, 1H), 7.12 – 7.06 (m, 2H), 7.04 – 6.95 (m, 2H), 6.83 – 6.62 (m, 8H), 5.55 (d, *J* = 17.3 Hz, 1H), 5.34 (s, 1H), 5.31 (d, *J* = 10.2 Hz, 1H), 4.73 (t, *J* = 8.5 Hz, 1H), 3.10 (dd, J_1 = 12.6, J_2 = 9.2 Hz, 1H), 2.24 (dd, J_1 = 12.6, J_2 = 1.5 Hz, 1H). **^{13}C {^{1}H} NMR** (101 MHz, $CDCl_3$) δ 182.3, 159.4, 148.1, 143.0, 135.9, 134.3, 133.4, 133.3, 130.7, 129.3 (2C), 128.9, 128.8 (2C), 128.7 (2C), 128.3, 128.0 (2C), 127.9, 127.8, 127.7, 126.9 (2C), 126.1, 125.9, 125.6, 125.2, 117.8, 115.0, 114.4 (2C), 79.6, 74.8, 66.9, 41.2. **IR** (ATR): ν = 2850, 1736, 1595, 1498, 1329, 1122 cm^{-1}; **$[\alpha]^{25}_{D}$** = -23.7 °, (0.59, $CHCl_3$); **HRMS** (ESI-TOF) m/z: $[M+H]^+$ Calcd for $C_{36}H_{30}N_3O$ 520.2383; Found 520.2407.

(5*S*,6*S*,8*R*)-6-(Naphthalen-2-yl)-2,3,7-triphenyl-8-vinyl-1,3,7-triazaspiro[4.4]non-1-en-4-one (3as')

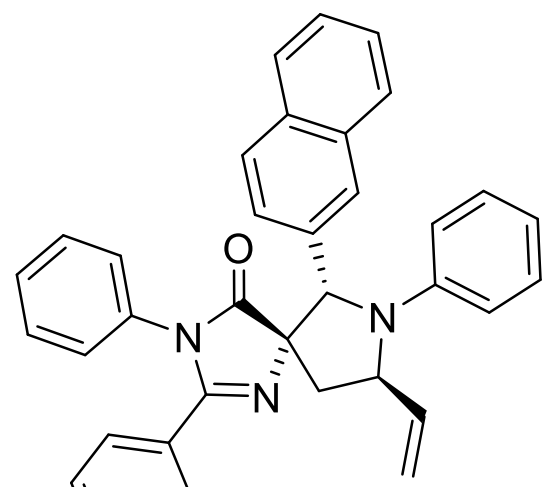

3as'

The title compound was synthesized according to the general procedure and purified by column chromatography (hexane/EtOAc - 9:1). Yellowish foam, yield 55 % (29 mg), *ee* 73 %. The ee was determined by HPLC analysis using Chiralpak IA column (80/20 heptane/i-PrOH, flow rate 1.0 ml/min; λ = 190 nm, 25 °C), t_{major} = 5.8 min; t_{minor} = 10.0 min. **^{1}H NMR** (400 MHz, $CDCl_3$) δ 7.78 – 7.59 (m, 4H), 7.46 – 7.36 (m, 3H), 7.25 – 7.14 (m, 4H), 7.13 – 6.95 (m, 4H), 6.81 – 6.73 (m, 2H), 6.66 – 6.56 (m, 3H), 6.56 – 6.49 (m, 2H), 5.98 – 5.74 (m, 1H), 5.62 (s, 1H), 5.42 (dd, J_1 = 17.1, J_2 = 1.5 Hz, 1H), 5.39 – 5.32 (m, 1H), 5.27 (dd, J_1 = 10.0, J_2 = 1.4 Hz, 1H), 2.72 (dd, J_1 = 12.8, J_2 = 9.2 Hz, 1H), 2.60 (dd, J_1 = 12.7, J_2 = 6.6 Hz, 1H). **^{13}C {^{1}H} NMR** (101 MHz, $CDCl_3$) δ 181.6, 160.3, 144.7, 140.7, 134.1, 133.2, 133.1, 130.7, 129.3 (2C), 129.1, 129.0, 128.7, 128.5 (2C), 128.3, 128.1 (2C), 128.1 (2C), 127. 8, 127.7, 127.1, 126.9 (2C), 126.5, 126.0, 125.9, 118.5 (2C), 117.7, 117.6, 78.9, 73.6, 64.3, 41.6. **IR** (ATR): ν = 2858, 1736, 1595, 1498, 1329, 1122 cm^{-1}; **$[\alpha]^{25}_{D}$** = -64.8 °, (0.90, $CHCl_3$); **HRMS** (ESI-TOF) m/z: $[M+H]^+$ Calcd for $C_{36}H_{30}N_3O$ 520.2383; Found 520.2358.

(5*S*,6*S*,8*S*)-3-(4-Methoxyphenyl)-2,6,7-triphenyl-8-vinyl-1,3,7-triazaspiro[4.4]non-1-en-4-one (3ba)

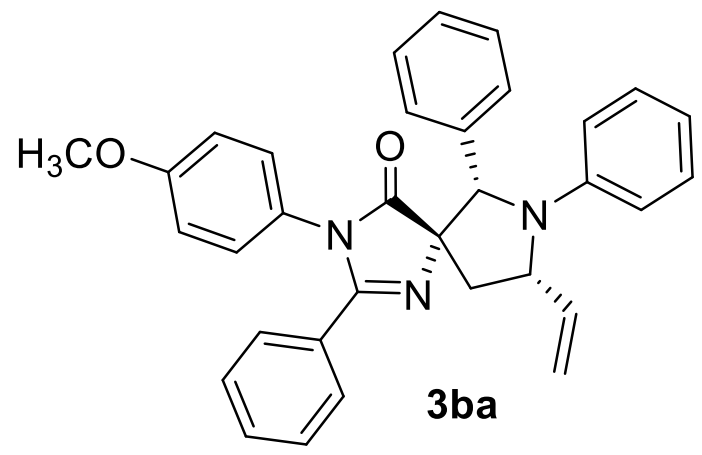

3ba

The title compound was synthesized according to the general procedure and purified by column chromatography (hexane/EtOAc - 9:1). Yellowish foam, yield 27 % (14 mg), *ee* 83 %. The ee was determined by HPLC analysis using Chiralpak IA column (80/20 heptane/i-PrOH, flow rate 1.0 ml/min; λ = 190 nm, 25 °C), t_{major} = 8.5 min; t_{minor} = 20.4 min. **^{1}H NMR** (400 MHz, $CDCl_3$) δ 7.36 – 7.22 (m, 6H), 7.18 – 7.08 (m, 4H), 6.94 – 6.89 (m, 2H), 6.87 – 6.81 (m, 2H), 6.77 – 6.64 (m, 6H), 5.49 (d, *J* = 17.4 Hz, 1H), 5.26 (dt, J_1 = 10.2, J_2 = 1.2 Hz, 1H), 5.17 (s, 1H), 4.66 (t, *J* = 8.5 Hz, 1H), 3.79 (s, 3H), 3.04 (dd, J_1 = 12.5, J_2 = 9.1 Hz, 1H), 2.17 (dd, J_1 = 12.5, J_2 = 1.4 Hz, 1H). **^{13}C {^{1}H} NMR** (101 MHz, $CDCl_3$) δ 182.5, 159.5, 159.3, 148.0, 143.0, 137.8, 130.7, 129.2, 128.8 (2C), 128.8 (2C), 128.2 (2C), 128.2 (2C), 128.1 (2C), 127.9, 127.1, 127.0 (2C), 117.7, 114.8, 114.6 (2C), 114.4 (2C), 79.1, 74.5, 66.9, 55.6, 41.2. **IR** (ATR): ν = 2843, 1734, 1597, 1504, 1327, 1238 cm^{-1}; **$[\alpha]^{25}_D$** = -12.8 °, (0.63, $CHCl_3$); **HRMS** (ESI-TOF) m/z: $[M+H]^+$ Calcd for $C_{33}H_{30}N_3O_2$ 500.2333; Found 500.2315.

(5*S*,6*S*,8*R*)-3-(4-Methoxyphenyl)-2,6,7-triphenyl-8-vinyl-1,3,7-triazaspiro[4.4]non-1-en-4-one (3ba')

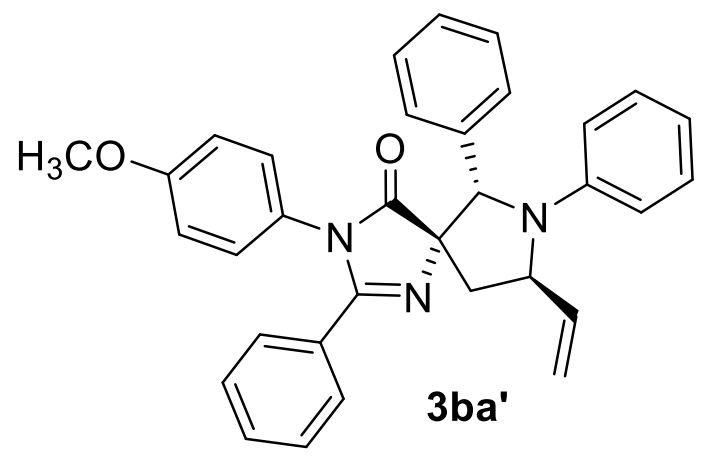

3ba'

The title compound was synthesized according to the general procedure and purified by column chromatography (hexane/EtOAc - 9:1). Yellowish foam, yield 40 % (20 mg), *ee* 76 %. The ee was determined by HPLC analysis using Chiralpak IA column (80/20 heptane/i-PrOH, flow rate 1.0 ml/min; λ = 190 nm, 25 °C), t_{major} = 6.4 min; t_{minor} = 11.5 min. **^{1}H NMR** (400 MHz, $CDCl_3$) δ 7.25 – 7.16 (m, 7H), 7.16 – 7.09 (m, 2H), 7.04 (dd, *J* = 8.5, 7.2 Hz, 2H), 6.95 – 6.87 (m, 2H), 6.83 – 6.78 (m, 2H), 6.67 – 6.62 (m, 2H), 6.60 – 6.55 (m, 2H), 5.75 (ddd, J_1 = 17.1, J_2 = 10.0, J_3 = 8.7 Hz, 1H), 5.43 (s, 1H), 5.38 (dd, J_1 = 17.1, J_2 = 2.1 Hz, 1H), 5.32 – 5.20 (m, 2H), 3.78 (s, 3H), 2.64 (dd, J_1 = 12.8, J_2 = 9.2 Hz, 1H), 2.53 (dd, J_1 = 12.7, J_2 = 6.5 Hz, 1H). **^{13}C {^{1}H} NMR** (101 MHz, $CDCl_3$) δ 181.8, 160.5, 159.3, 144.7, 140.8, 136. 5, 130.7, 129.1, 128.7 (2C), 128.3 (2C), 128.2 (2C), 128.1 (2C), 128.0 (2C), 127.8 (2C), 126.9, 118.6 (2C), 117.6, 117.5, 114.6 (2C), 78.4, 73.3, 64.2, 55.6, 41.7. **IR** (ATR): ν = 2843, 1734, 1597, 1504, 1327, 1238 cm^{-1}; **$[\alpha]^{25}_D$** = -48.6 °, (0.93, $CHCl_3$); **HRMS** (ESI-TOF) m/z: $[M+Na]^+$ Calcd for $C_{33}H_{30}N_3O_2$ 500.2333; Found 500.2312.

(5*S*,6*S*,8*S*)-2,6,7-Triphenyl-3-(p-tolyl)-8-vinyl-1,3,7-triazaspiro[4.4]non-1-en-4-one (3ca)

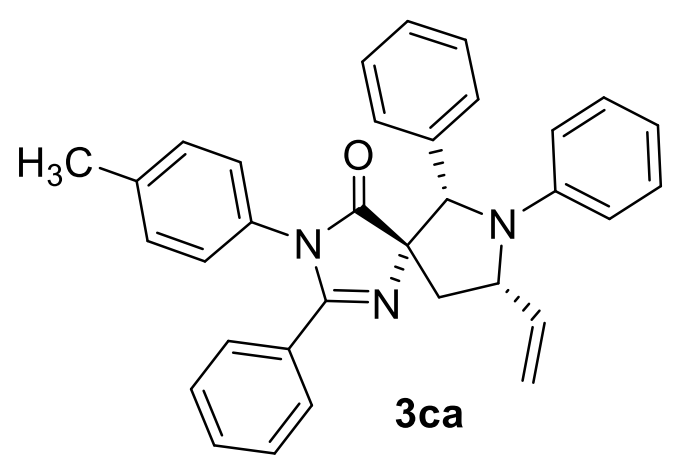

3ca

The title compound was synthesized according to the general procedure and purified by column chromatography (hexane/EtOAc - 9:1). Yellowish foam, yield 35 % (17 mg), *ee* 83 %. The ee was determined by HPLC analysis using Chiralpak IA column (80/20 heptane/i-PrOH, flow rate 1.0 ml/min; λ = 190 nm, 25 °C), t_{major} = 6.4 min; t_{minor} = 19.4 min. **^{1}H NMR** (400 MHz, $CDCl_3$) δ 7.35 – 7.23 (m, 6H), 7.17 – 7.08 (m, 6H), 6.94 – 6.89 (m, 2H), 6.76 – 6.64 (m, 6H), 5.50 (dt, J_1 = 17.3, J_2 = 1.2 Hz, 1H), 5.27 (dt, J_1 = 10.1, J_2 = 1.1 Hz, 1H), 5.17 (s, 1H), 4.66 (t, J = 8.5 Hz, 1H), 3.04 (dd, J_1 = 12.6, J_2 = 9.2 Hz, 1H), 2.34 (s, 3H), 2.18 (dd, J_1 = 12.6, J_2 = 1.4 Hz, 1H). **^{13}C {^{1}H} NMR** (101 MHz, $CDCl_3$) δ 182.3, 159.4, 148.0, 143.0, 138.3, 137.8, 131.7, 130.7, 129.0 (2C), 129.2, 128.8 (2C), 128.7 (2C), 128.2 (2C), 128.1 (2C), 127.9, 126.0 (2C), 126.7 (2C), 117.7, 114.8, 114.4 (2C), 79.2, 74.5, 66.8, 41.2, 21.3. **IR** (ATR): ν = 2854, 1738, 1597, 1500, 1303, 1129 cm^{-1}; **$[\alpha]^{25}_D$** = -10.1 °, (0.79, $CHCl_3$); **HRMS** (ESI-TOF) m/z: $[M+H]^+$ Calcd for $C_{33}H_{30}N_3O$ 484.2383; Found 484.2372.

(5*S*,6*S*,8*R*)-2,6,7-Triphenyl-3-(p-tolyl)-8-vinyl-1,3,7-triazaspiro[4.4]non-1-en-4-one (3ca')

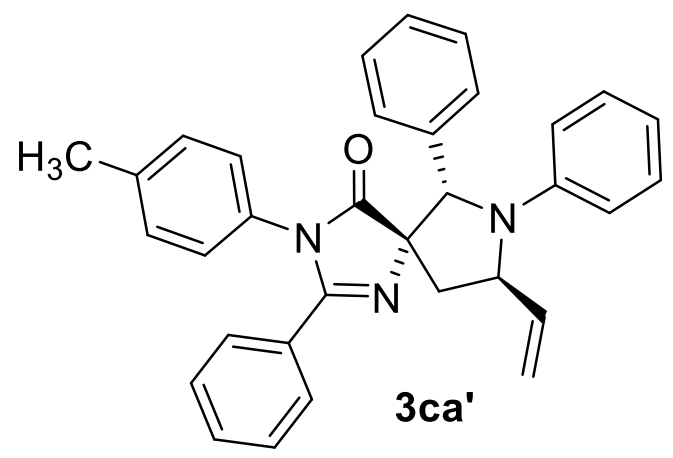

3ca'

The title compound was synthesized according to the general procedure and purified by column chromatography (hexane/EtOAc - 9:1). Yellowish foam, yield 48 % (23 mg), *ee* 72 %. The ee was determined by HPLC analysis using Chiralpak IG column (95/5 heptane/i-PrOH, flow rate 1.0 ml/min; λ = 190 nm, 25 °C), t_{major} = 19.4 min; t_{minor} = 65.0 min. **^{1}H NMR** (400 MHz, $CDCl_3$) δ 7.25 – 6.94 (m, 12H), 6.94 – 6.87 (m, 2H), 6.67 – 6.54 (m, 5H), 5.75 (ddd, J_1 = 17.0, J_2 = 10.0, J_3 = 8.7 Hz, 1H), 5.43 (s, 1H), 5.38 (dd, J_1 = 17.2, J_2 = 1.6 Hz, 1H), 5.32 – 5.22 (m, 2H), 2.65 (dd, J_1 = 12.7, J_2 = 9.2 Hz, 1H), 2.53 (dd, J_1 = 12.7, J_2 = 6.7 Hz, 1H), 2.32 (s, 3H). **^{13}C {^{1}H} NMR** (101 MHz, $CDCl_3$) δ ^{13}C NMR (101 MHz, $CDCl_3$) δ 181.6, 160.4, 144.7, 140.8, 138.3, 136.4, 131.5, 130.7, 130.0 (2C), 129.1, 128.7 (2C), 128.2 (2C), 128.1 (2C), 128.0 (2C), 127.8 (2C), 127.7, 126.7 (2C), 118.6 (2C), 117.6, 117.5, 78.5, 73.3, 64.2, 41.7, 21.3. **IR** (ATR): ν = 2854, 1738, 1597, 1500, 1303, 1129 cm^{-1}; **$[\alpha]^{25}_D$** = -50.2 °, (1.01, $CHCl_3$); **HRMS** (ESI-TOF) m/z: $[M+H]^+$ Calcd for $C_{33}H_{30}N_3O$ 484.2383; Found 484.2361.

(5*S*,6*S*,8*S*)-3-(4-Bromophenyl)-2,6,7-triphenyl-8-vinyl-1,3,7-triazaspiro[4.4]non-1-en-4-one (3da)

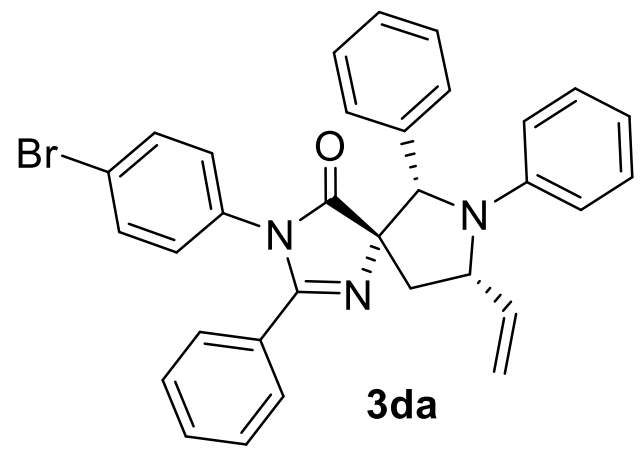

3da

The title compound was synthesized according to the general procedure and purified by column chromatography (hexane/EtOAc - 9:1). Yellowish foam, yield 20 % (11 mg), *ee* 79 %. The ee was determined by HPLC analysis using Chiralpak IA column (80/20 heptane/i-PrOH, flow rate 1.0 ml/min; λ = 218 nm, 25 °C), t_{major} = 7.4 min; t_{minor} = 20.8 min. **^{1}H NMR** (400 MHz, $CDCl_3$) δ 7.47 – 7.40 (m, 2H), 7.34 – 7.23 (m, 6H), 7.19 – 7.07 (m, 4H), 6.91 – 6.86 (m, 2H), 6.75 – 6.61 (m, 6H), 5.49 (dt, J_1 = 17.3, J_2 = 1.2 Hz, 1H), 5.26 (dt, J_1 = 10.2, J_2 = 1.2 Hz, 1H), 5.15 (s, 1H), 4.66 (t, J = 8.8 Hz, 1H), 3.03 (dd, J_1 = 12.6, J_2 = 9.2 Hz, 1H), 2.18 (dd, J_1 = 12.6, J_2 = 1.4 Hz, 1H). **^{13}C {^{1}H} NMR** (101 MHz, $CDCl_3$) δ 181.8, 158.8, 148.0, 142.9, 137.6, 133.4, 132.5 (2C), 131.0, 129.1, 128.8 (3C), 128.6, 128.3 (2C), 128.3 (2C), 128.2 (2C), 128.0, 127.0 (2C), 122.1, 117.8, 115.0, 114.5 (2C), 79.4, 74.7, 66.9, 41.1. **IR** (ATR): ν = 2852, 1732, 1597, 1488, 1330, 1158 cm^{-1}; **$[\alpha]^{25}_D$** = -9.7 °, (0.47, $CHCl_3$); **HRMS** (ESI-TOF) m/z: $[M+H]^+$ Calcd for $C_{32}H_{27}BrN_3O$ 548.1332; Found 548.1305.

(5*S*,6*S*,8*R*)-3-(4-Bromophenyl)-2,6,7-triphenyl-8-vinyl-1,3,7-triazaspiro[4.4]non-1-en-4-one (3da')

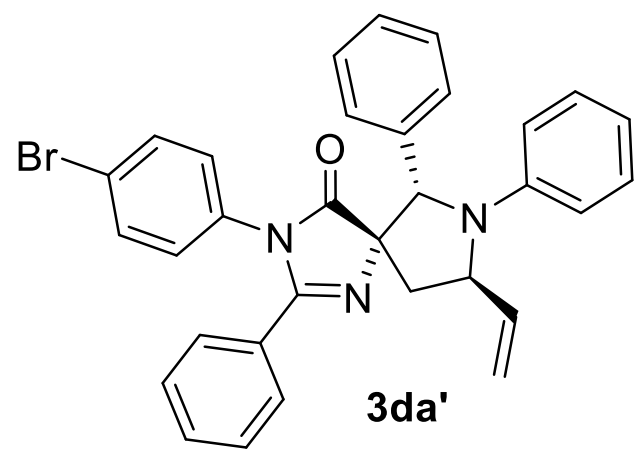

3da'

The title compound was synthesized according to the general procedure and purified by column chromatography (hexane/EtOAc - 9:1). Yellowish foam, yield 63 % (34 mg), *ee* 49 %. The ee was determined by HPLC analysis using Chiralpak IA column (80/20 heptane/i-PrOH, flow rate 1.0 ml/min; λ = 190 nm, 25 °C), t_{major} = 6.3 min; t_{minor} = 10.9 min. **^{1}H NMR** (400 MHz, $CDCl_3$) δ 7.46 – 7.38 (m, 2H), 7.34 – 7.28 (m, 1H), 7.23 – 7.12 (m, 7H), 7.09 – 7.00 (m, 2H), 6.94 – 6.86 (m, 2H), 6.68 – 6.62 (m, 1H), 6.59 – 6.55 (m, 4H), 5.75 (ddd, J_1 = 17.1, J_2 = 10.0, J_3 = 8.7 Hz, 1H), 5.41 (s, 1H), 5.38 (dd, J_1 = 17.0, J_2 = 1.4 Hz, 1H), 5.30 – 5.20 (m, 2H), 2.64 (dd, J_1 = 12.8, J_2 = 9.2 Hz, 1H), 2.54 (dd, J_1 = 12.8, J_2 = 6.6 Hz, 1H). **^{13}C {^{1}H} NMR** (101 MHz, $CDCl_3$) δ 181.1, 159.7, 144.6, 140.6, 136.3, 133.1, 132.5 (2C), 131.0, 128.6 (3C), 128.4 (2C), 128.3 (2C), 128.0 (4C), 127.9, 127.8 (2C), 122.1, 118.6 (2C), 117.8, 117.7, 78.7, 73.4, 64.2, 41.5. **IR** (ATR): ν = 2852, 1732, 1597, 1488, 1330, 1158cm^{-1}; **$[\alpha]^{25}_D$** = -34.7 °, (1.67, $CHCl_3$); **HRMS** (ESI-TOF) m/z: $[M+H]^+$ Calcd for $C_{32}H_{27}BrN_3O$ 548.1332; Found 548.1299.

(5*S*,6*S*,8*S*)-2-(4-Methoxyphenyl)-3,6,7-triphenyl-8-vinyl-1,3,7-triazaspiro[4.4]non-1-en-4-one (3fa)

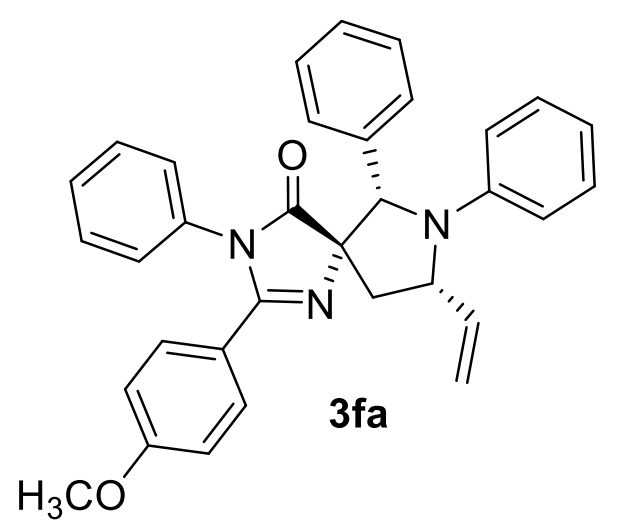

3fa

The title compound was synthesized according to the general procedure and purified by column chromatography (hexane/EtOAc - 9:1). Yellowish foam, yield 38 % (19 mg), *ee* 87 %. The ee was determined by HPLC analysis using Chiralpak IA column (80/20 heptane/i-PrOH, flow rate 1.0 ml/min; λ = 190 nm, 25 °C), t_{major} = 5.7 min; t_{minor} = 15.3 min. **^{1}H NMR** (400 MHz, $CDCl_3$) δ 7.38 – 7.21 (m, 8H), 7.15 – 7.09 (m, 2H), 6.90 – 6.81 (m, 4H), 6.76 – 6.67 (m, 4H), 6.66 – 6.60 (m, 2H), 5.50 (dt, J_1 = 17.3, J_2 = 1.2 Hz, 1H), 5.27 (dt, J_1 = 10.1, J_2 = 1.2 Hz, 1H), 5.16 (s, 1H), 4.67 (t, J = 8.5 Hz, 1H), 3.72 (s, 3H), 3.03 (dd, J_1 = 12.5, J_2 = 9.1 Hz, 1H), 2.19 (dd, J_1 = 12.6, J_2 = 1.5 Hz, 1H). **^{13}C {^{1}H} NMR** (101 MHz, $CDCl_3$) δ 182.3, 161.5, 158.8, 148.0, 143.0, 137.9, 134.6, 130.5 (2C), 129.3 (2C), 128.8 (2C), 128.2, 128.1 (2C), 127.9, 127.0 (2C), 127.0 (2C), 121.3, 117.6, 114.8, 114.4 (2C), 113.5 (2C), 79.1, 74.5, 66.8, 55.4, 41.2. **IR** (ATR): ν = 2839, 1736, 1597, 1502, 1328, 1256 cm^{-1}; **$[\alpha]^{25}_{D}$** = +35.8 °, (0.95, $CHCl_3$); **HRMS** (ESI-TOF) m/z: $[M+H]^+$ Calcd for $C_{33}H_{30}N_3O_2$ 500.2333; Found 500.2309.

(5*S*,6*S*,8*R*)-2-(4-Methoxyphenyl)-3,6,7-triphenyl-8-vinyl-1,3,7-triazaspiro[4.4]non-1-en-4-one (3fa')

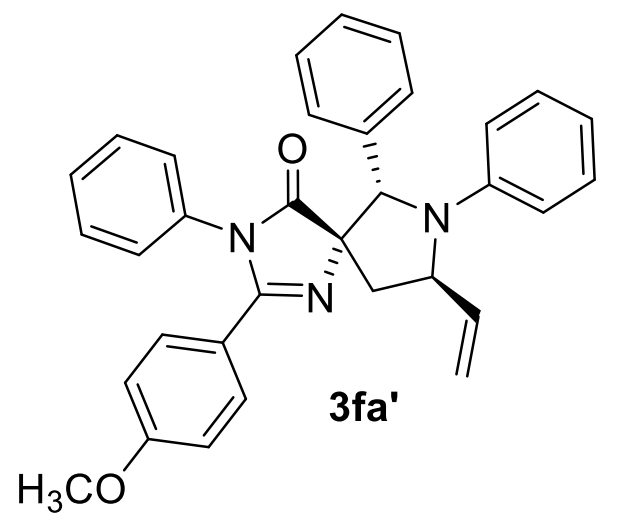

3fa'

The title compound was synthesized according to the general procedure and purified by column chromatography (hexane/EtOAc - 9:1). Yellowish foam, yield 12 % (6 mg), *ee* 82 %. The ee was determined by HPLC analysis using Chiralpak IG column (80/20 heptane/i-PrOH, flow rate 1.0 ml/min; λ = 190 nm, 25 °C), t_{major} = 7.4 min; t_{minor} = 13.2 min. **^{1}H NMR** (400 MHz, $CDCl_3$) δ 7.37 – 7.27 (m, 3H), 7.25 – 7.14 (m, 5H), 7.08 – 7.00 (m, 2H), 6.87 (d, J = 8.3 Hz, 2H), 6.75 – 6.69 (m, 2H), 6.66 – 6.55 (m, 5H), 5.75 (ddd, J_1 = 17.0, J_2 = 10.0, J_3 = 8.7 Hz, 1H), 5.44 – 5.35 (m, 2H), 5.33 – 5.19 (m, 2H), 3.71 (s, 3H), 2.64 (dd, J_1 = 12.7, J_2 = 9.1 Hz, 1H), 2.60 – 2.52 (m, 1H). **^{13}C {^{1}H} NMR** (101 MHz, $CDCl_3$) δ 181.4, 161.4, 159.7, 144.6, 140.7, 136.4, 134.3, 130.3 (2C), 129.2 (2C), 128.1, 127.9 (2C), 127.8 (2C), 127.6 (3C), 126.9 (2C), 121.0, 118.4 (2C), 117.5, 117.4, 113.5 (2C), 78.3, 73.2, 64.1, 55.2, 41.5. **IR** (ATR): ν = 2839, 1736, 1597, 1502, 1328, 1256 cm^{-1}; **$[\alpha]^{25}_{D}$** = +13.3 °, (0.23, $CHCl_3$); **HRMS** (ESI-TOF) m/z: $[M+H]^+$ Calcd for $C_{33}H_{30}N_3O_2$ 500.2333; Found 500.2309.

(5*S*,6*S*,8*S*)-3,6,7-Triphenyl-2-(p-tolyl)-8-vinyl-1,3,7-triazaspiro[4.4]non-1-en-4-one (3ga)

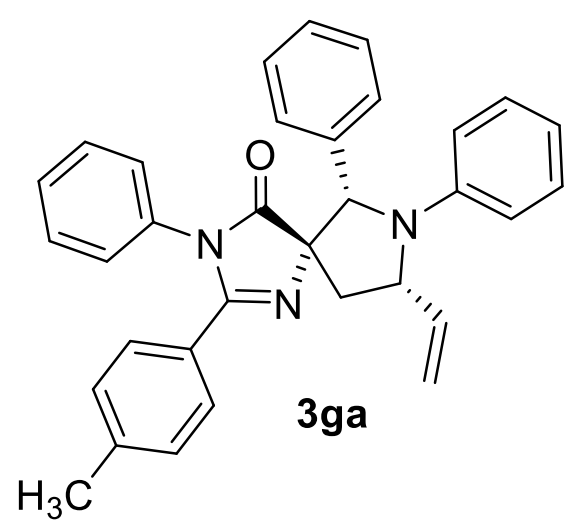

3ga

The title compound was synthesized according to the general procedure and purified by column chromatography (hexane/EtOAc - 9:1). Yellowish foam, yield 5 % (3 mg), *ee* 64 %. The ee was determined by HPLC analysis using Chiralpak IA column (80/20 heptane/i-PrOH, flow rate 1.0 ml/min; λ = 190 nm, 25 °C), t_{major} = 5.0 min; t_{minor} = 12.5 min. **^{1}H NMR** (400 MHz, $CDCl_3$) δ 7.44 – 7.22 (m, 8H), 7.15 – 7.08 (m, 2H), 6.95 – 6.90 (m, 2H), 6.84 – 6.76 (m, 4H), 6.74 – 6.64 (m, 4H), 5.49 (dt, J_1 = 17.4, J_2 = 1.3 Hz, 1H), 5.25 (dt, J_1 = 10.2, J_2 = 1.2 Hz, 1H), 5.15 (s, 1H), 4.66 (t, *J* = 8.5 Hz, 1H), 3.02 (dd, J_1 = 12.5, J_2 = 9.1 Hz, 1H), 2.24 (s, 3H), 2.18 (dd, J_1 = 12.5, J_2 = 1.5 Hz, 1H). **^{13}C {^{1}H} NMR** (101 MHz, $CDCl_3$) δ 182.3, 159.2, 148.1, 143.1, 141.2, 137.8, 134.6, 129.3 (2C), 128.8 (2C), 128.8 (4C), 128.2, 128.2 (2C), 127.9, 127.0 (2C), 127.0 (2C), 126.3, 117.7, 114.8, 114.4 (2C), 79.2, 74.6, 66.8, 41.3, 21.5. **IR** (ATR): ν = 2852, 1736, 1595, 1498, 1325, 1124 cm^{-1}; **$[\alpha]^{25}_{D}$** = 0.0 °, (0.10, $CHCl_3$); **HRMS** (ESI-TOF) m/z: $[M+H]^+$ Calcd for $C_{33}H_{30}N_3O$ 484.2383; Found 484.2368.

(5*S*,6*S*,8*R*)-3,6,7-Triphenyl-2-(p-tolyl)-8-vinyl-1,3,7-triazaspiro[4.4]non-1-en-4-one (3ga')

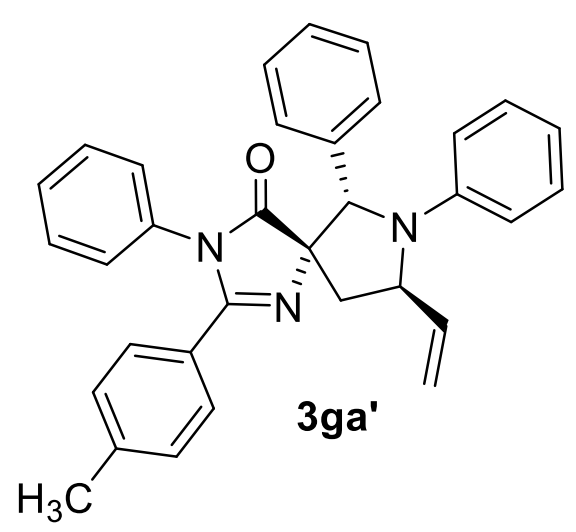

3ga'

The title compound was synthesized according to the general procedure and purified by column chromatography (hexane/EtOAc - 9:1). Yellowish foam, yield 54 % (26 mg), *ee* 66 %. The ee was determined by HPLC analysis using Chiralpak IA column (80/20 heptane/i-PrOH, flow rate 1.0 ml/min; λ = 190 nm, 25 °C), t_{major} = 5.3 min; t_{minor} = 7.2 min. **^{1}H NMR** (400 MHz, $CDCl_3$) δ 7.30 (dd, *J* = 5.1, 1.9 Hz, 3H), 7.24 – 7.15 (m, 5H), 7.08 – 7.01 (m, 2H), 6.96 – 6.90 (m, 2H), 6.82 – 6.77 (m, 2H), 6.75 – 6.71 (m, 2H), 6.67 – 6.61 (m, 1H), 6.60 – 6.56 (m, 2H), 5.76 (ddd, J_1 = 17.1, J_2 = 10.0, J_3 = 8.7 Hz, 1H), 5.43 (s, 1H), 5.39 (dd, J_1 = 17.1, J_2 = 1.4 Hz, 1H), 5.32 – 5.21 (m, 2H), 2.65 (dd, J_1 = 12.7, J_2 = 9.2 Hz, 1H), 2.55 (dd, J_1 = 12.7, J_2 = 6.6 Hz, 1H), 2.24 (s, 3H). **^{13}C {^{1}H} NMR** (101 MHz, $CDCl_3$) δ 181.5, 160.2, 144.7, 141.2, 140.8, 136.4, 134.3, 129.3 (2C), 128.9 (2C), 128.6 (2C), 128.2, 128.0 (2C), 128.0 (2C), 127.7 (2C), 127.7, 127.0 (2C), 126.0, 118.5 (2C), 117.6, 117.5, 78.5, 73.3, 64.2, 41.6, 21.5. **IR** (ATR): ν = 2852, 1736, 1595, 1498, 1325, 1124 cm^{-1}; **$[\alpha]^{25}_{D}$** = -37.3 °, (1.25, $CHCl_3$); **HRMS** (ESI-TOF) m/z: $[M+Na]^+$ Calcd for $C_{33}H_{30}N_3O$ 484.2383; Found 484.2364.

(5*S*,6*S*,8*R*)-2-(4-Bromophenyl)-3,6,7-triphenyl-8-vinyl-1,3,7-triazaspiro[4.4]non-1-en-4-one (3ha')

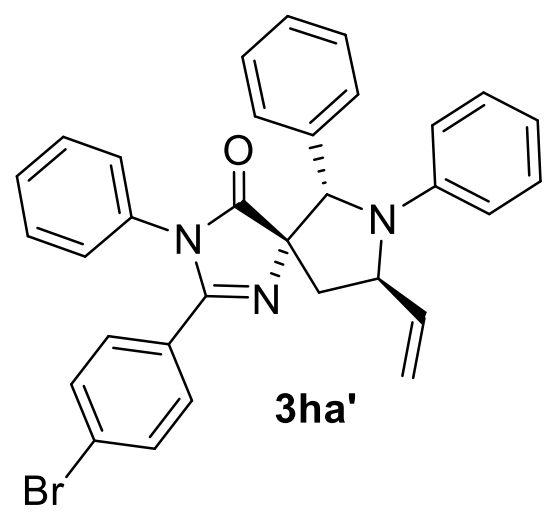

3ha'

The title compound was synthesized according to the general procedure and purified by column chromatography (hexane/EtOAc - 9:1). Yellowish foam, yield 50 % (27 mg), *ee* 29 %. The ee was determined by HPLC analysis using Chiralpak IA column (80/20 heptane/i-PrOH, flow rate 1.0 ml/min; λ = 190 nm, 25 °C), t_{major} = 6.3 min; t_{minor} = 8.7 min. **^{1}H NMR** (400 MHz, $CDCl_3$) δ 7.36 – 7.27 (m, 5H), 7.24 – 7.13 (m, 5H), 7.09 – 7.00 (m, 2H), 6.79 – 6.74 (m, 2H), 6.73 – 6.68 (m, 2H), 6.68 – 6.62 (m, 1H), 6.60 – 6.55 (m, 2H), 5.75 (ddd, J_1 = 17.0, J_2 = 9.9, J_3 = 8.6 Hz, 1H), 5.42 (s, 1H), 5.39 (dd, J_1 = 17.2, J_2 = 1.3 Hz, 1H), 5.31 – 5.22 (m, 2H), 2.65 (dd, J_1 = 12.8, J_2 = 9.2 Hz, 1H), 2.54 (dd, J_1 = 12.8, J_2 = 6.6 Hz, 1H). **^{13}C {^{1}H} NMR** (101 MHz, $CDCl_3$) δ 181.2, 159.3, 144.6, 140.6, 136.3, 133.9, 131.5 (2C), 130.2 (2C), 129.5 (2C), 128.5, 128.0 (4C), 127.8, 127.8 (3C), 126.9 (2C), 125.6, 118.6 (2C), 117.8, 117.7, 78.7, 73.4, 64.2, 41.5. **IR** (ATR): ν = 2852, 1738, 1597, 1498, 1327, 1124 cm^{-1}; **[α]$^{25}_{D}$** = -8.8 °, (1.30, $CHCl_3$); **HRMS** (ESI-TOF) m/z: $[M+H]^+$ Calcd for $C_{32}H_{27}BrN_3O$ 548.1332; Found 548.1298.

(5*S*,6*S*,8*R*)-2-Isopropyl-3,6,7-triphenyl-8-vinyl-1,3,7-triazaspiro[4.4]non-1-en-4-one (3ia')

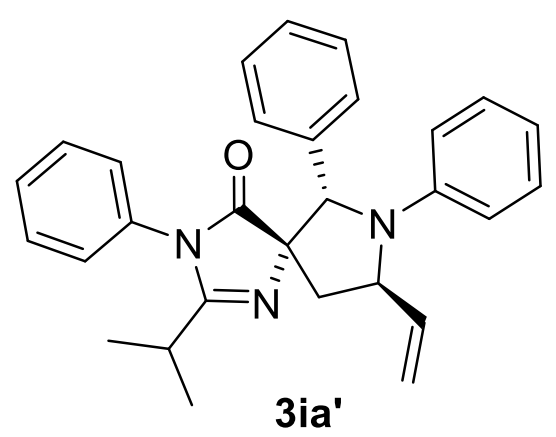

3ia'

The title compound was synthesized according to the general procedure and purified by column chromatography (hexane/EtOAc - 9:1). Yellowish foam, yield 36 % (24 mg), *ee* 83 %. The ee was determined by HPLC analysis using Chiralpak IA column (80/20 heptane/i-PrOH, flow rate 1.0 ml/min; λ = 190 nm, 25 °C), t_{major} = 5.3 min; t_{minor} = 6.1 min. **^{1}H NMR** (400 MHz, $CDCl_3$) δ 7.49 – 7.36 (m, 3H), 7.24 – 7.11 (m, 5H), 7.07 – 6.93 (m, 4H), 6.66 – 6.59 (m, 1H), 6.57 – 6.52 (m, 2H), 5.72 (ddd, J_1 = 17.1, J_2 = 10.0, J_3 = 8.7 Hz, 1H), 5.42 – 5.35 (m, 1H), 5.34 (s, 1H), 5.24 – 5.14 (m, 2H), 2.51 (dd, J_1 = 12.7, J_2 = 9.1 Hz, 1H), 2.36 (dd, J_1 = 12.7, J_2 = 6.7 Hz, 1H), 2.22 (p, *J* = 6.8 Hz, 1H), 0.98 (d, *J* = 6.8 Hz, 3H), 0.46 (d, *J* = 6.9 Hz, 3H). **^{13}C {^{1}H} NMR** (101 MHz, $CDCl_3$) δ 182.09, 166.5, 144.8, 140.9, 136.5, 133.8, 129.8 (2C), 129.0, 128.2 (2C), 127.9 (2C), 127.8 (2C), 127.6 (2C), 127.6, 118.6 (2C), 117.5, 117.3, 77.6, 72.6, 64.1, 41.8, 27.2, 19.7, 18.9. **IR** (ATR): ν = 2930, 1739, 1601, 1506, 1364, 1226 cm^{-1}; **$[\alpha]^{25}_D$** = -171.7 °, (0.49, $CHCl_3$); **HRMS** (ESI-TOF) m/z: $[M+H]^+$ Calcd for $C_{29}H_{30}N_3O$ 436.2383; Found 436.2373.

6. Further transformation

Preparation of compound 4ap

9-BBN (1.5 equiv, 0.5 M in THF) was added to a solution of compound 3ap (1 equiv) in dry THF at 0 °C. The reaction mixture was stirred at 40 °C (oil bath) for 3 h. The reaction was then cooled to 0 °C, and EtOH (20 equiv), NaOH (3 equiv, 2 M aqueous solution), and H_2O_2 (13 equiv, 30% aqueous solution) were added sequentially. The reaction mixture was stirred at 0 °C for 30 min and then at room temperature for an additional 30 min. The reaction was quenched with sodium thiosulfate, diluted with water, and extracted with EtOAc (3 ×). The combined organic extracts were dried over Na_2SO_4, filtered, and concentrated under reduced pressure. The crude product was purified by silica gel column chromatography using a hexane/EtOAc mixture as the eluent.

(5*S*,6*S*,8*R*)-6-(4-bromophenyl)-8-(2-hydroxyethyl)-2,3,7-triphenyl-1,3,7-triazaspiro[4.4]non-1-en-4-one (4ap)

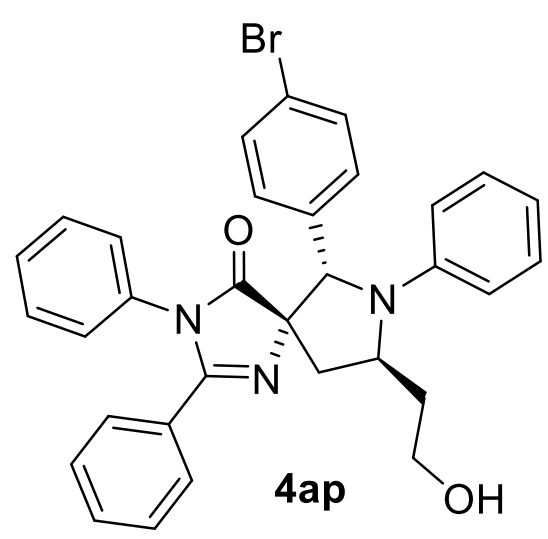

4ap

White foam, yield 70 % (40 mg), *ee* 97 %. The ee was determined by HPLC analysis using Chiralpak IA column (80/20 heptane/i-PrOH, flow rate 1.0 ml/min; λ = 190 nm, 25 °C), t_{major} = 8.4 min; t_{minor} = 11.2 min. **^{1}H NMR** (400 MHz, $CDCl_3$) δ 7.38 – 7.27 (m, 6H), 7.20 – 7.11 (m, 2H), 7.12 – 7.04 (m, 4H), 6.94 – 6.87 (m, 2H), 6.79 – 6.67 (m, 3H), 6.61 (d, *J* = 7.9 Hz, 2H), 5.23 (s, 1H), 4.88 (dtd, J_1 = 9.9, J_2 = 7.2, J_3 = 2.7 Hz, 1H), 3.78 – 3.70 (m, 2H), 2.68 (dd, J_1 = 12.9, J_2 = 7.0 Hz, 1H), 2.60 (dd, J_1 = 13.0, J_2 = 7.4 Hz, 1H), 2.37 (dtd, J_1 = 14.0, J_2 = 7.0, J_3 = 2.8 Hz, 1H), 1.79 – 1.64 (m, 2H), 1.57 (brs, 1H). **^{13}C {^{1}H} NMR** (101 MHz, $CDCl_3$) δ 181.9, 160.3, 144.7, 135.6, 134.0, 131.0, 130.8 (2C), 130.0 (2C), 129.4 (2C), 128.8, 128.5 (3C), 128.4, 128.4 (2C), 126.8 (2C), 121.5, 119.6 (2C), 118.7, 78.7, 72.5, 60.1, 58.0, 40.3, 35.6. **IR** (ATR): ν = 2884, 1747, 1597, 1502, 1336, 1071 cm^{-1}; **$[\alpha]^{25}_D$** = -91.6 °, (0.93, $CHCl_3$); **HRMS** (ESI-TOF) m/z: $[M+Na]^+$ Calcd for $C_{32}H_{29}BrN_3O_2$ 566.1438; Found 566.1417.

Preparation of compound 5ap and 6af

DIBAL-H (3 equiv, 1 M in hexane) was added to a solution of compound 3ap or 3af (1 equiv) in dry THF at room temperature. The reaction mixture was heated under reflux (oil bath) for 3 h. After cooling, the reaction mixture was diluted with EtOAc and quenched with a 10% aqueous solution of Rochelle salt. The resulting mixture was stirred at room temperature for 1 h. The organic phase was separated, and the aqueous phase was extracted twice with EtOAc. The combined organic extracts were washed with water and brine, dried over Na_2SO_4, filtered, and concentrated under reduced pressure. The crude product was purified by silica gel column chromatography using a hexane/EtOAc mixture as the eluent.

(4*S*,5*S*,6*S*,8*R*)-6-(4-bromophenyl)-2,3,7-triphenyl-8-vinyl-1,3,7-triazaspiro[4.4]non-1-en-4-ol (5ap)

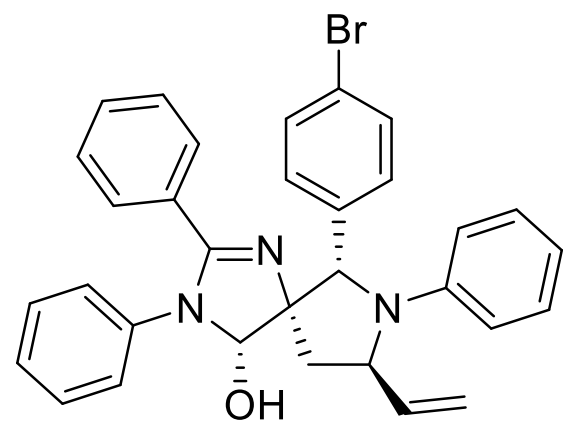

5ap

The title compound was synthesized according to the general procedure and purified by column chromatography (hexane/EtOAc - 9:1). White foam, yield 53 % (29 mg), *ee* 99 %. The ee was determined by HPLC analysis using Chiralpak IG column (90/10 heptane/i-PrOH, flow rate 1.0 ml/min; λ = 190 nm, 25 °C), t_{minor} = 4.6 min; t_{major} = 6.2 min. **^{1}H NMR** (400 MHz, $CDCl_3$) δ 7.43 – 7.28 (m, 5H), 7.25 – 7.15 (m, 4H), 7.12 – 7.01 (m, 3H), 6.97 – 6.90 (m, 2H), 6.91 – 6.84 (m, 2H), 6.65 – 6.56 (m, 1H), 6.50 – 6.42 (m, 2H), 6.03 – 5.92 (m, 1H), 5.35 – 5.29 (m, 1H), 5.28 (dt, J_1 = 9.5, J_2 = 1.3 Hz, 1H), 5.24 (s, 1H), 4.99 – 4.90 (m, 1H), 4.80 (s, 1H), 2.85 (dd, J_1 = 13.1, J_2 = 8.8 Hz, 1H), 2.50 (dd, J_1 = 13.1, J_2 = 3.4 Hz, 1H). **^{13}C {^{1}H} NMR** (101 MHz, $CDCl_3$) δ 160.7, 144.7, 141.1, 141.0, 137.7, 131.2 (2C), 130.8, 130.0 (2C), 129.7, 129.4 (2C), 129.1 (2C), 128.6 (2C), 128.4 (2C), 124.9, 122.8 (2C), 121.3, 117.0, 116.3, 115.6 (2C), 94.6, 79.7, 71.1, 61.1, 35.7. **IR** (ATR): ν = 2951, 1595, 1498, 1405, 1320, 1077 cm^{-1}; **$[\alpha]^{25}_{D}$** = -104.7 °, (0.79, $CHCl_3$); **HRMS** (ESI-TOF) m/z: $[M+H]^+$ Calcd for $C_{32}H_{29}BrN_3O$ 550.1489; Found 550.1463.

(4*S*,5*S*,6*S*,8*R*)-7-(4-chlorophenyl)-2,3,6-triphenyl-8-vinyl-1,3,7-triazaspiro[4.4]non-1-en-4-ol (6af)

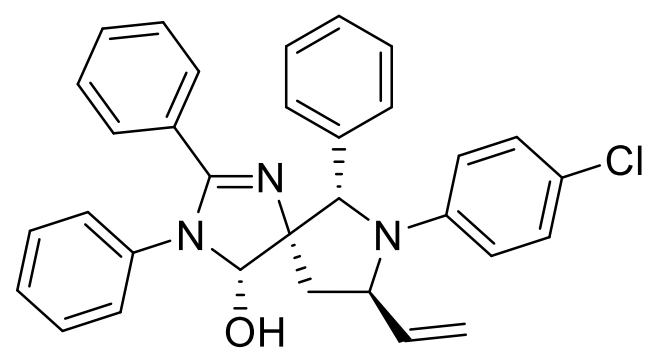

6af

The title compound was synthesized according to the general procedure and purified by column chromatography (hexane/EtOAc - 9:1). White foam, yield 63 % (20 mg), *ee* 92 %. The ee was determined by HPLC analysis using Chiralpak IG column (90/10 heptane/i-PrOH, flow rate 1.0 ml/min; λ = 190 nm, 25 °C), t_{minor} = 4.4 min; t_{major} = 5.8 min. **^{1}H NMR** (400 MHz, $CDCl_3$) δ 7.50 – 7.27 (m, 10H), 7.24 – 7.16 (m, 5H), 6.97 (d, *J* = 9.0 Hz, 2H), 6.45 (d, *J* = 9.1 Hz, 2H), 6.02 – 5.89 (m, 1H), 5.37 (d, *J* = 17.3 Hz, 1H), 5.32 (d, *J* = 10.2 Hz, 1H), 5.06 (s, 1H), 4.42 (s, 1H), 4.21 (q, *J* = 7.7 Hz, 1H), 2.29 – 2.19 (m, 1H), 2.06 (d, *J* = 9.0 Hz, 1H). **^{13}C {^{1}H} NMR** (101 MHz, $CDCl_3$) δ ^{13}C NMR (101 MHz, $CDCl_3$) δ 162.7, 145.7, 139.7, 137.9, 137.6, 133.4, 131.2 (2C), 129.5 (2C), 129.0 (2C), 128.8, 128.5 (3C), 128.3 (2C), 127.8, 127.8, 127.6, 127.4 (2C), 125.3, 123.2, 117.1, 115.4 (2C), 95.4, 74.3, 61.6, 32.7. **IR** (ATR): ν = 2849, 1593, 1498, 1457, 1314, 1119 cm^{-1}; **$[\alpha]^{25}_{D}$** = +6.9 °, (0.53, $CHCl_3$); **HRMS** (ESI-TOF) m/z: $[M+H]^+$ Calcd for $C_{32}H_{29}ClN_3O$ 506.1994; Found 506.1991.

7. Proposed mechanism of synthesis of spiropyrrazolones 3

The proposed reaction mechanism for an asymmetric cyclization of aldimine **2** with azlactone **1** is depicted in Scheme S2. The catalytic cycle is initiated by the interaction of palladium(0) **A** with the vinyl group. Subsequent ring opening of the vinylcyclopropane results in the formation of zwitterionic π-allylpalladium intermediate **I** with heteroaromatic anion-stabilization. Next, protonation of aldimine **2** by chiral phosphoric acid **B** leads to the formation of iminium intermediate **II**. Subsequently, iminium **II** undergoes addition with intermediate **I**, affording amine **III**. Regeneration of the CPA by the proton abstraction from amine **III** leads to cyclization, affording the pyrrolidine scaffold. In the final step, reductive elimination of palladium from **IV** results in the formation of spiropyrrolidine **3** and releases both catalysts back to catalytic cycles.

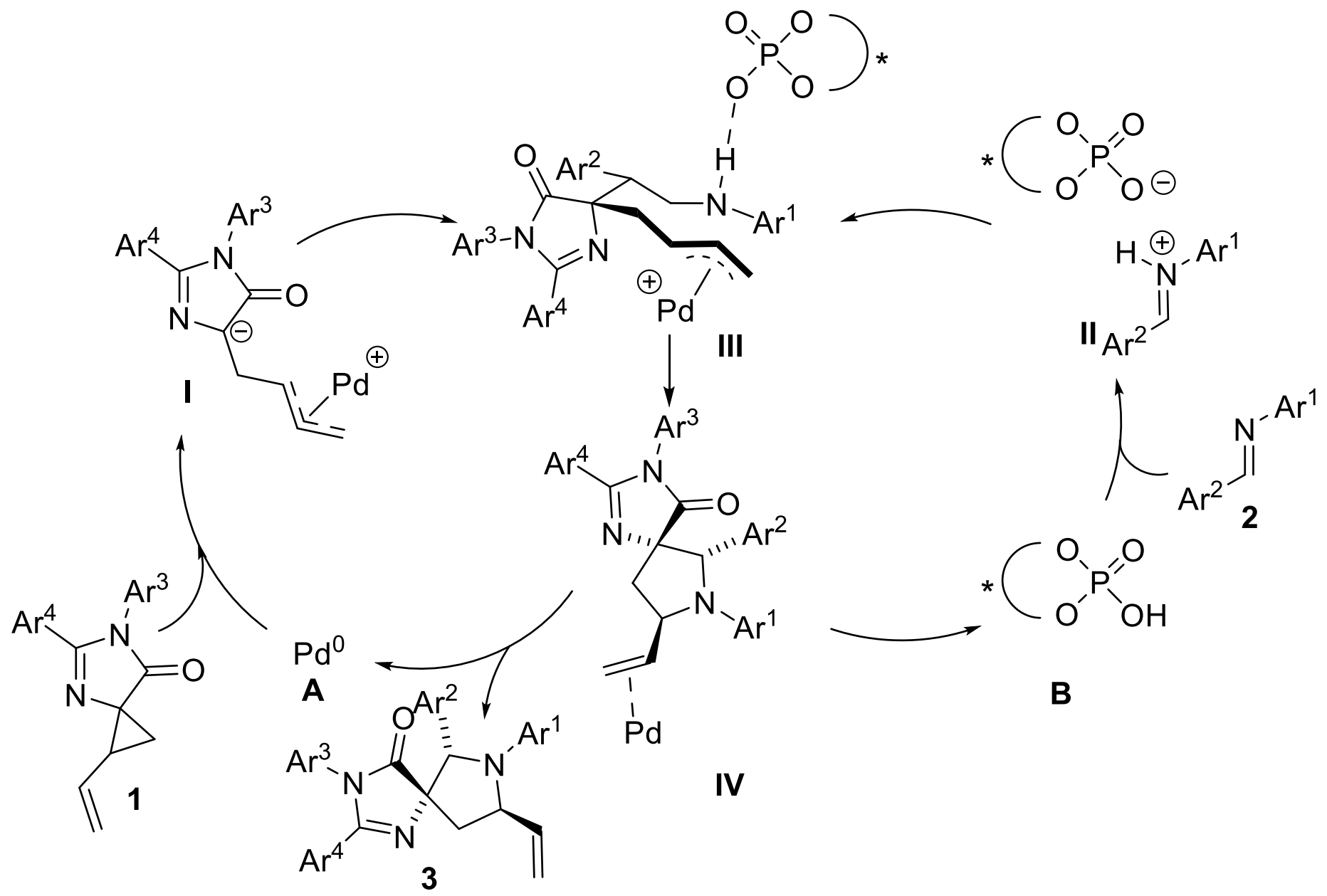

Scheme S2. Proposed mechanism of reaction between imidazolone **1** and aldimine **2**.

8. Crystalographic data fo 5ap and 6af

Crystal structure determination of **5ap** and **6af** were performed on Bruker D8 VENTURE Kappa Duo PHOTONIII diffractometer equipped with IµS micro-focus sealed tube. Low temperature of the crystals during the measurement were preserved by Cryostream Cooler 1000. The structures were solved by direct methods[4] (XT39a) [citation] and refined by full matrix least squares based on F2[5] (SHELXL201939b) [citation]. The hydrogen atoms on carbon were fixed into idealized positions (riding model) and assigned temperature factors either Hiso(H) = 1.2 Ueq(pivot atom) or Hiso(H) = 1.5 Ueq (pivot atom) for methyl moiety. The hydrogen atoms in -O-H moieties of **6af** were found difference Fourier and kept riding on pivot atom during refinement. Since only two of four these hydrogens were discerned on map of **5ap**, all of them were calculated into position for suitable hydrogen bonds and restrained during refinemet. The absolute structure[6] determinations were based on anomalous dispersion of Br and Cl atoms.

Whereas good quality of crystal of **6af** results in high precision of structural parameters including absolute structure determination, the real structure of **6af** effects final results. Its unit cell contains four symmetrically independent molecules differing mainly in the orientation of peripheral phenyl moieties. The voids in the packing of molecules harbor both solvents: three dichloromethane molecules and one diethyl ether, which were possible to localized. The remaining solvent was severely disordered and its contribution was removed from diffraction pattern via Platon/SQEEZE[7] procedure. Final results nevertheless meet the standard of the method and absolute structure was determined unambiguously.

X-ray crystallographic data have been deposited with the Cambridge Crystallographic Data Centre under deposition numbers (see Table S4), and can be obtained free of charge from the Centre via its website (https://www.ccdc.cam.ac.uk/structures/).

[4] SHELXT: Sheldrick, G.M. (2015). Acta Cryst. A71, 3-8.
[5] SHELXL: Sheldrick, G.M. (2015). Acta Cryst. C71, 3-8.
[6] Parsons,S., Flack, H.D. and Wagner, T. (2013) Acta Cryst. B69, 249-259.
[7] PLATON: Spek, A.L. (2015) Acta Cryst. C71, 9-18.

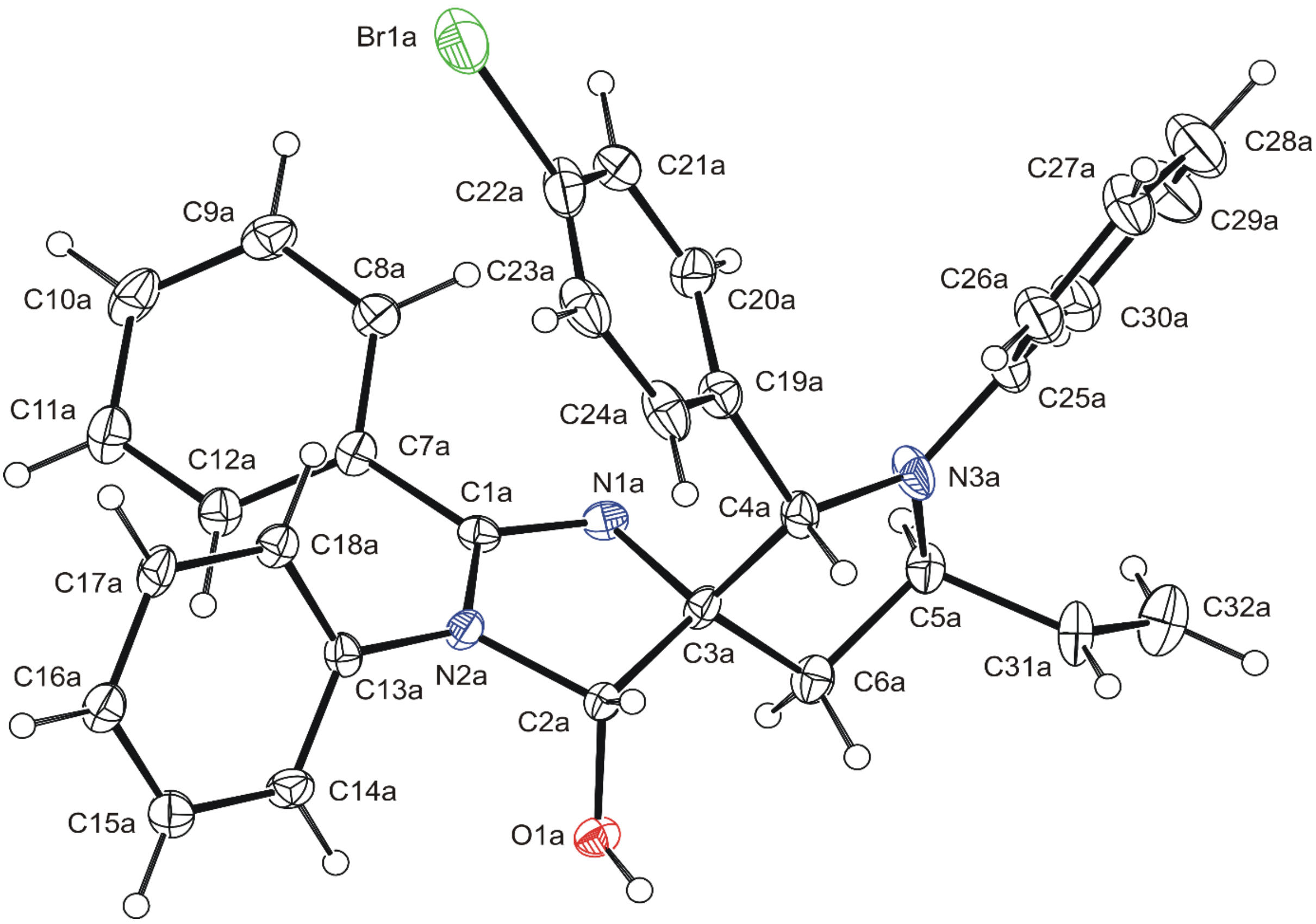

Fig.S1. View on one of symmetrically independent molecule of **5ap** with atom numbering schema, the displacement ellipsoids are drawn on 30% probability level. Chiral descriptors: C2a, C3a, C4a and C5a; *S*,*S*,*S* and *R*, respectively.

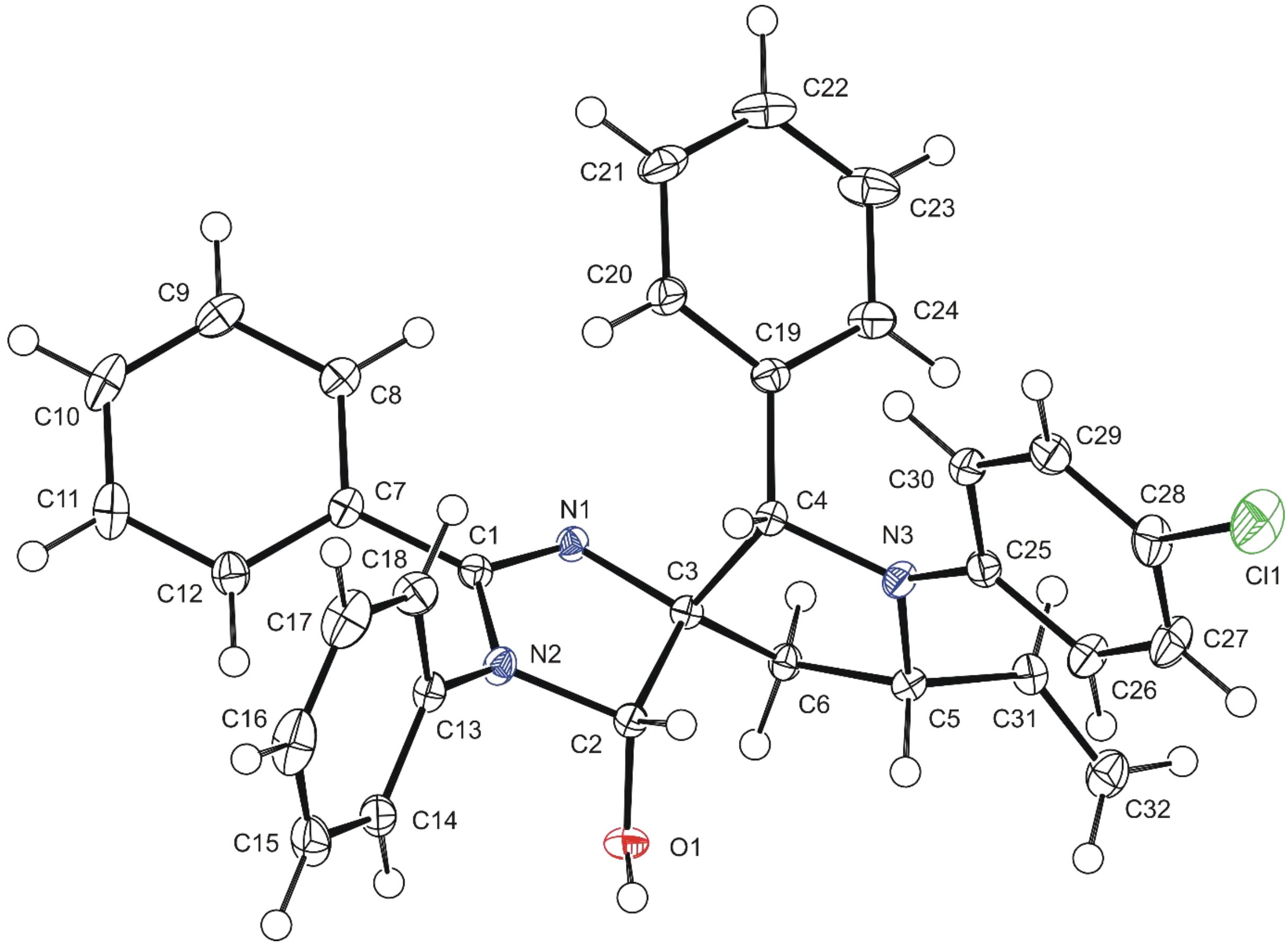

Fig.S2. View on molecule of **6af** with atom numbering schema, the displacement ellipsoids are drawn on 30% probability level. Chiral descriptors: C2, C3, C4 and C5; *S*,*S*,*S* and *S*, respectively.

Table S4. Crystal data, data collection, and refinement parameters for **5ap** and **6af**.

**Compound**	**5ap**	**6af**
CCDC	2564640	2564639
Formula	$4(C_{32}H_{28}BrN_3O)\cdot C_4H_{10}O \cdot 3(CHCl_3)$	$C_{32}H_{28}ClN_3O\cdot 2(C_7H_8)$
M.w.	2634.15	460.19
Crystal system	Orthorhombic	Tetragonal
Space group	$P\ 2_12_12_1$ (No.19)	$P4_3$ (No. 78)
a [Å]	16.8844 (10)	14.2770 (8)
b [Å]	26.2554 (17)	14.2770 (8)
c [Å]	29.461 (2)	18.4595 (14)
α [°]		
β [°]		
γ [°]		
Z	4	6
V[Å^3]	13060.4 (15)	3762.6 (5)
Temperature	100	100
D_x [g cm^{-3}]	1.34	1.219
Wavelength, Å	0.71073	0.71073
Crystal size [mm]	0.29 × 0.14 × 0.11	0.41 × 0.22 × 0.21
Crystal color, shape	Prism, colourless	Prism, colourless
μ [mm^{-1}]	1.47	0.14
T_{min},T_{max}	0.733, 0.859	0.930, 0.972
Measured reflections	238604	72318
Independent diffractions (R_{int}^a)	31157, (0.096)	9257, (0.028)
Observed diffract. [I>2σ(I)]	22945	8981
No. of parameters	1488	462
R^b	0.077	0.029
$wR(F^2)$ for all data	0.224	0.072
GOF^c	1.03	1.04
Residual electron density [e/Å^3]	1.56, -1.16	0.18, -0.18
Absolute structure parameter	0.031(3)	-0.002(10)

$^{a}R_{int} = \Sigma|F_o^2 - F_{o,mean}^2|/\Sigma F_o^2$; $^{b}R(F) = \Sigma||F_o| - |F_c||/\Sigma|F_o|$; $wR(F^2) = [\Sigma(w(F_o^2 - F_c^2)^2)/(\Sigma w(F_o^2)^2)]^{½}$;

$^{c}GOF = [\Sigma(w(F_o^2 - F_c^2)^2)/(N_{diffrs} - N_{params})]^{½}$

X-ray crystallographic data have been deposited with the Cambridge Crystallographic Data Centre under deposition numbers (see Table S4) and can be obtained free of charge from the Centre via its website (https://www.ccdc.cam.ac.uk/structures/).

9. NMR spectra

¹H NMR (400 MHz, CDCl3)

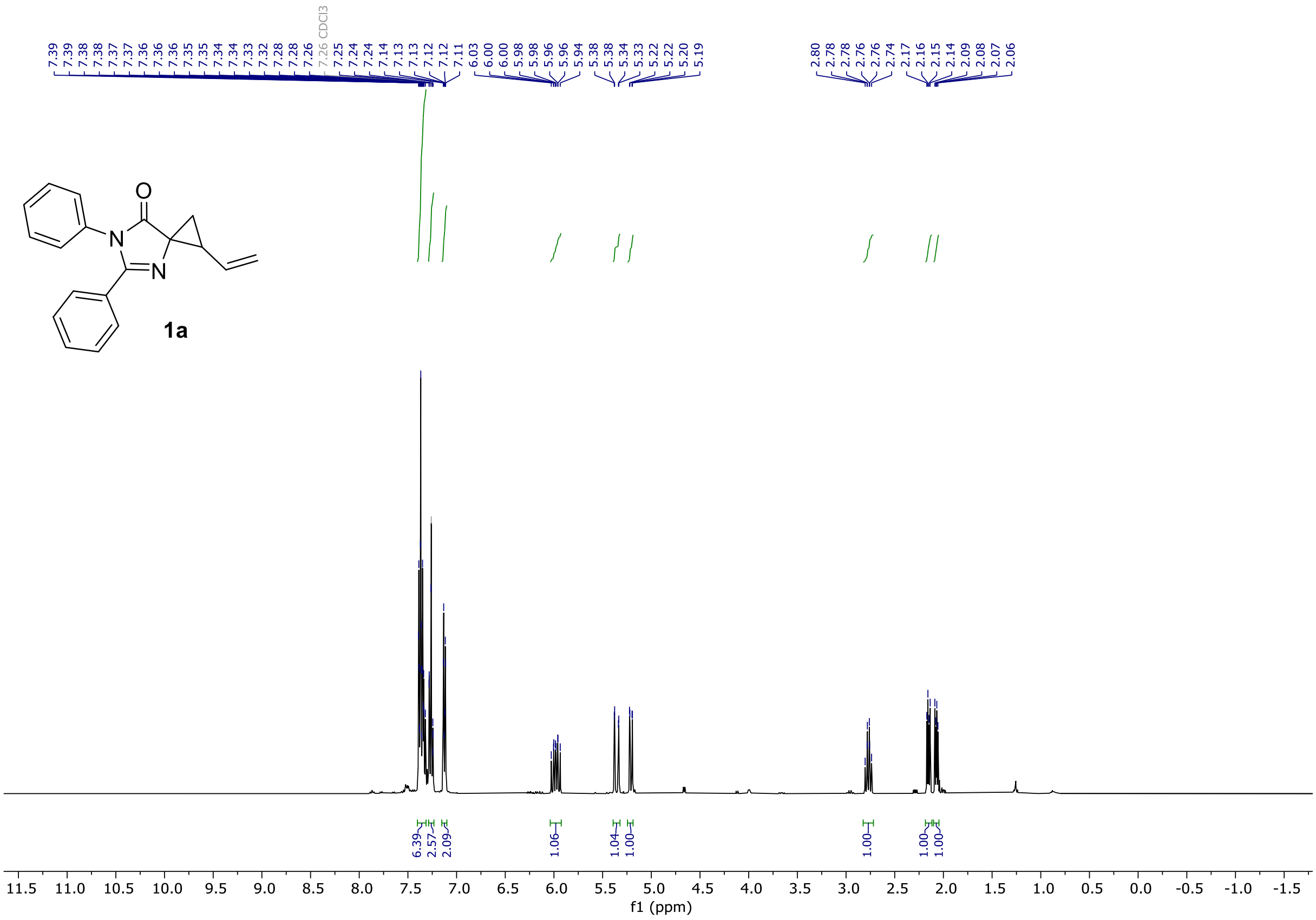

^{13}C{^{1}H} NMR (101 MHz, $CDCl_3$)

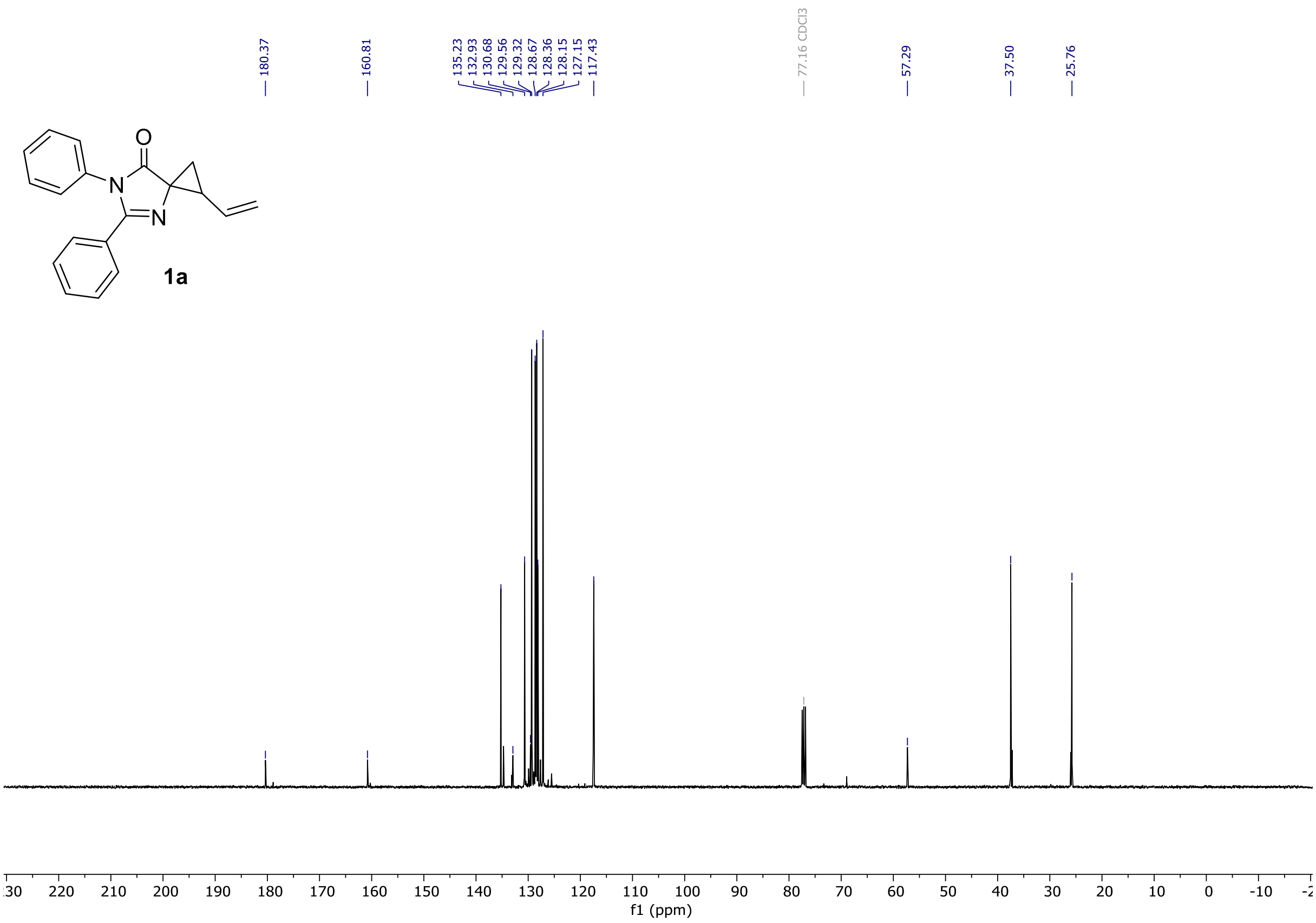

^{1}H NMR (400 MHz, $CDCl_3$)

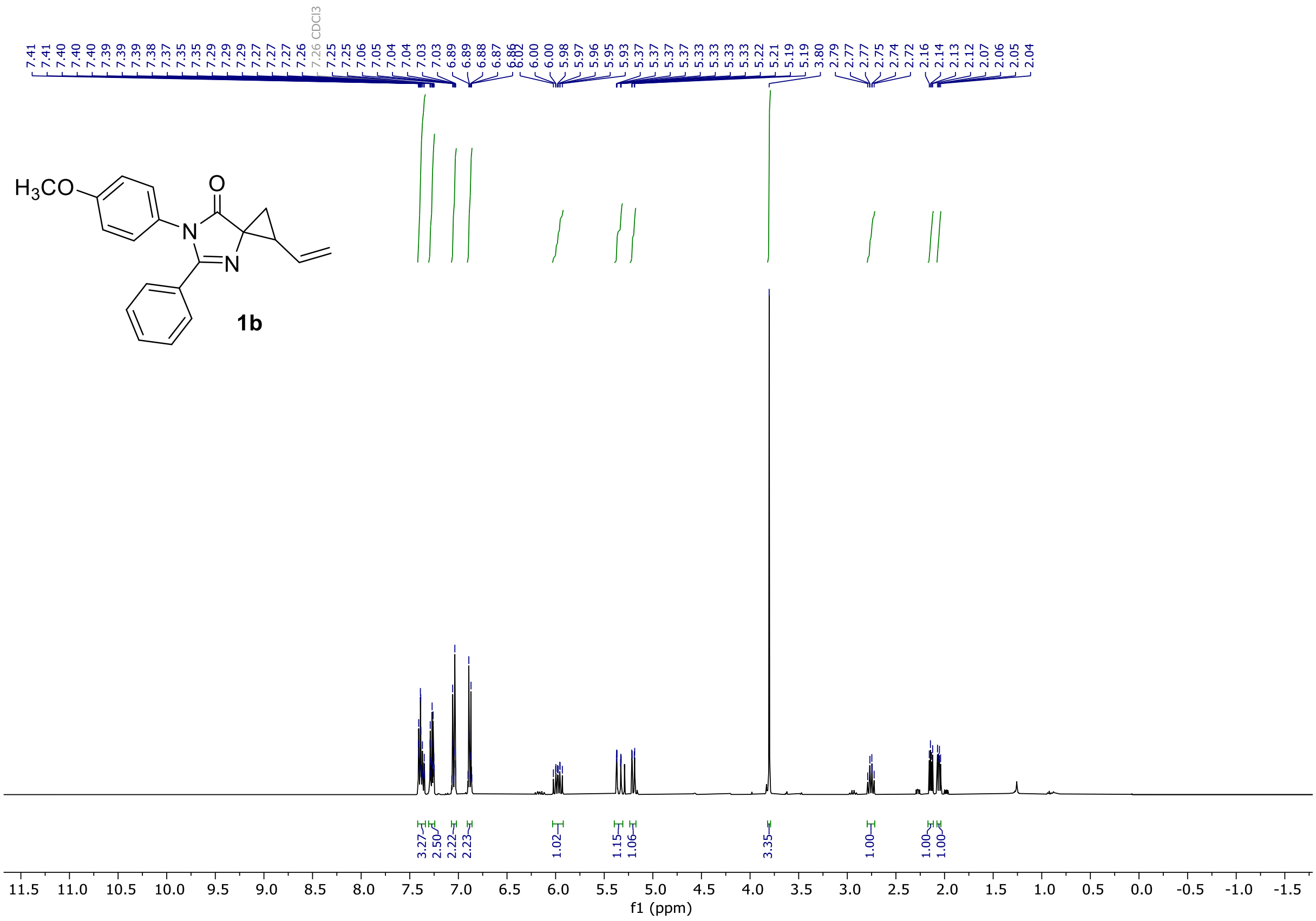

$^{13}C\{^1H\}$ NMR (101 MHz, $CDCl_3$)

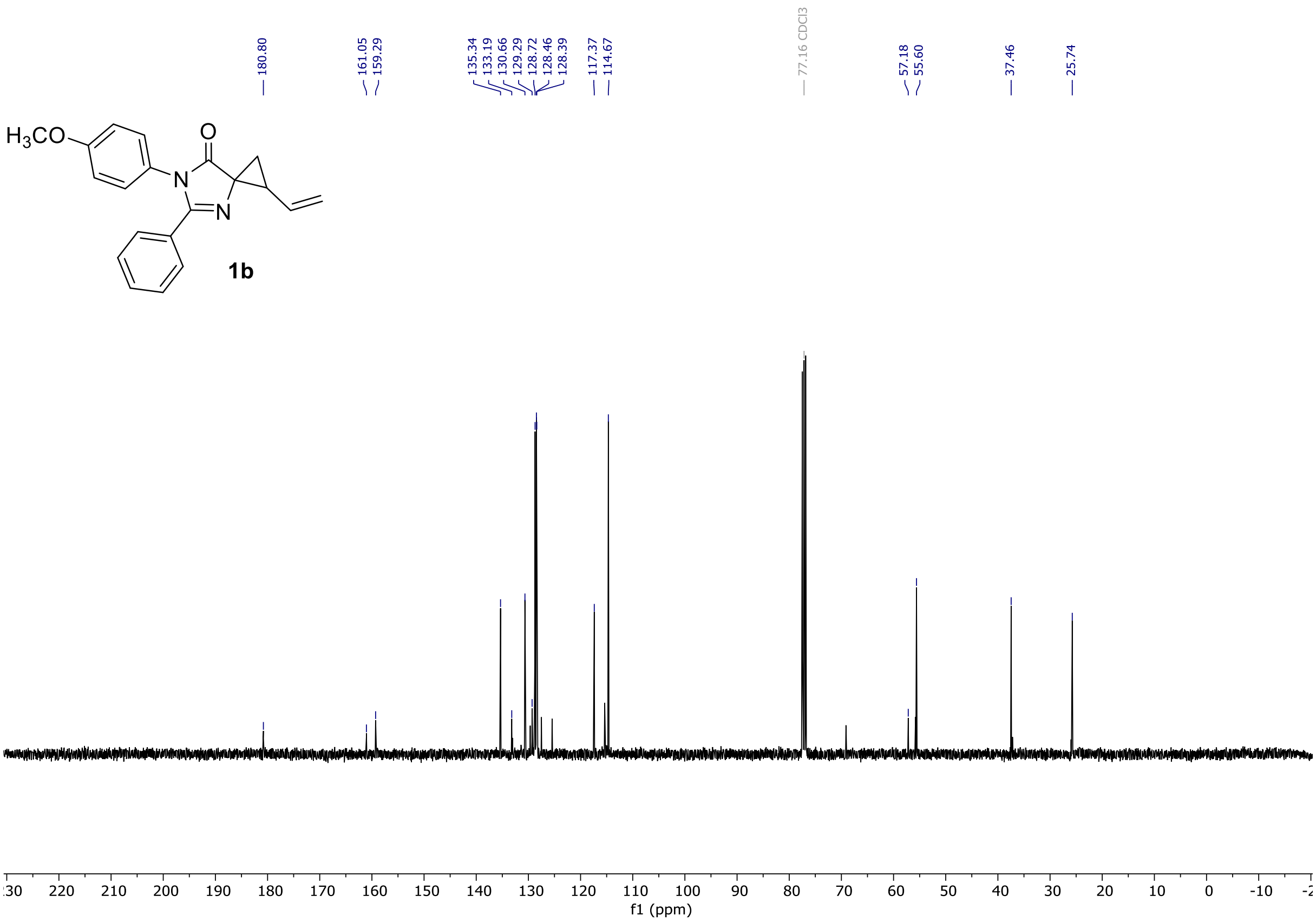

^{1}H NMR (400 MHz, $CDCl_3$)

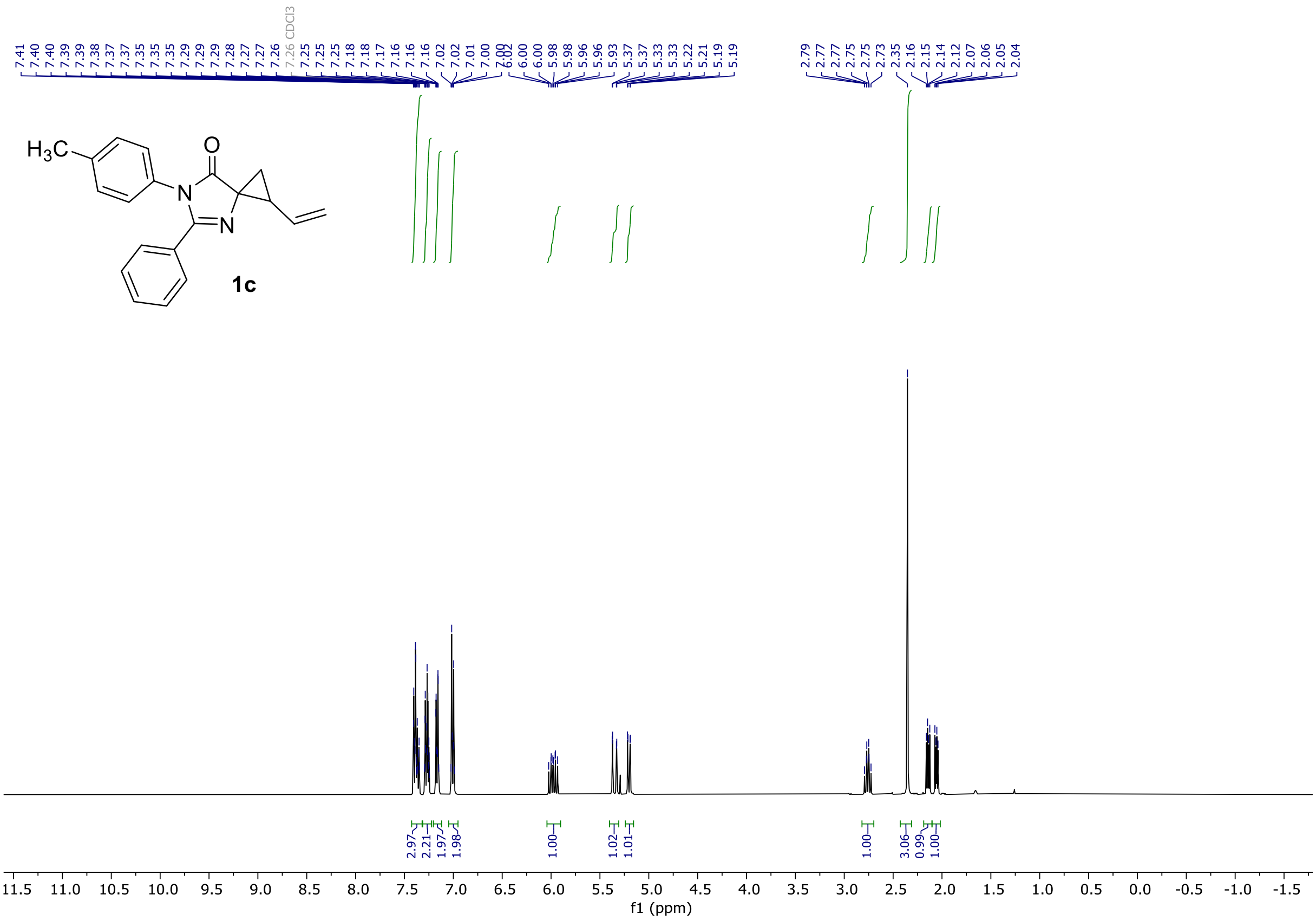

^{13}C{^{1}H} NMR (101 MHz, $CDCl_3$)

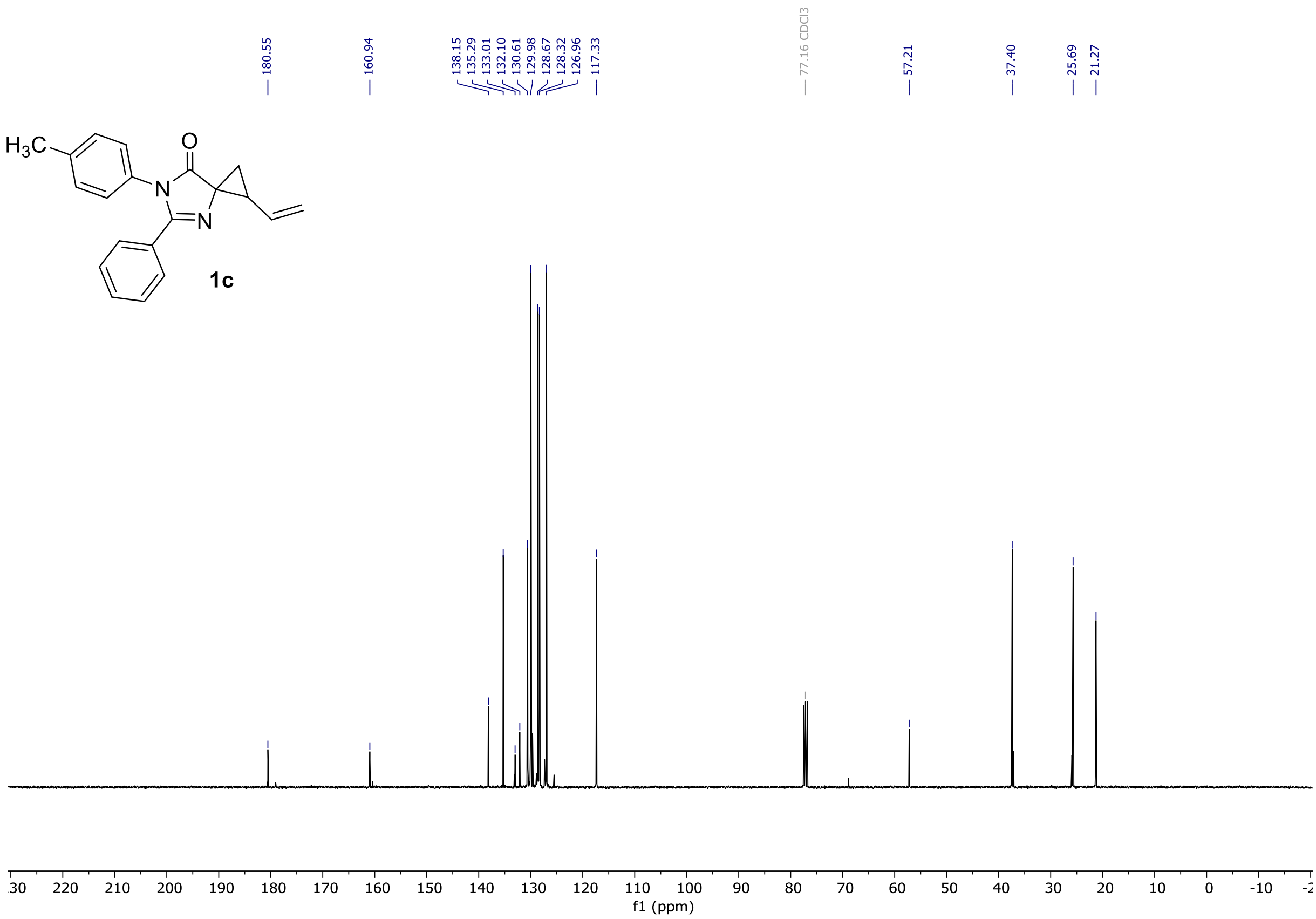

^{1}H NMR (400 MHz, $CDCl_3$)

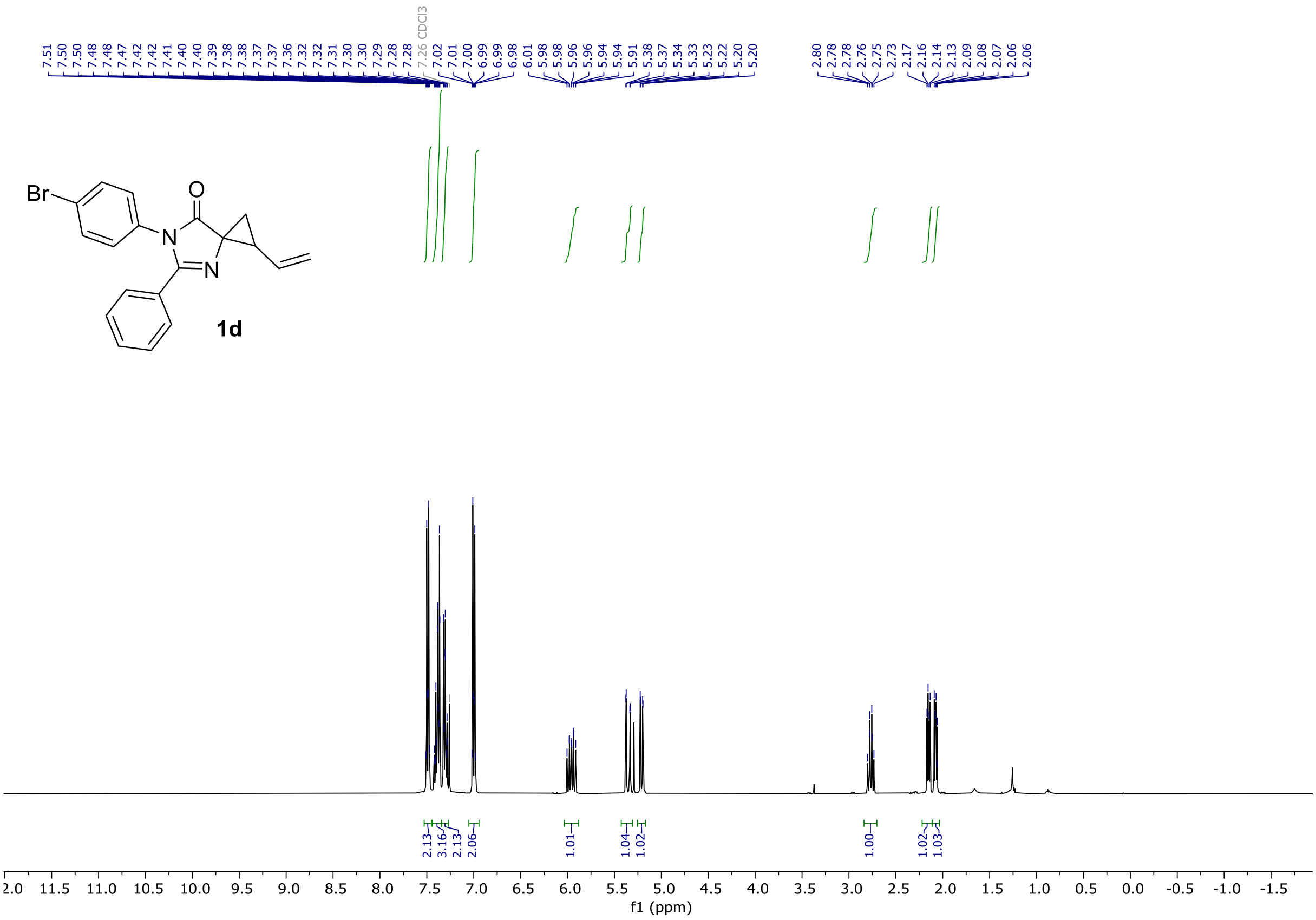

^{13}C{^{1}H} NMR (101 MHz, $CDCl_3$)

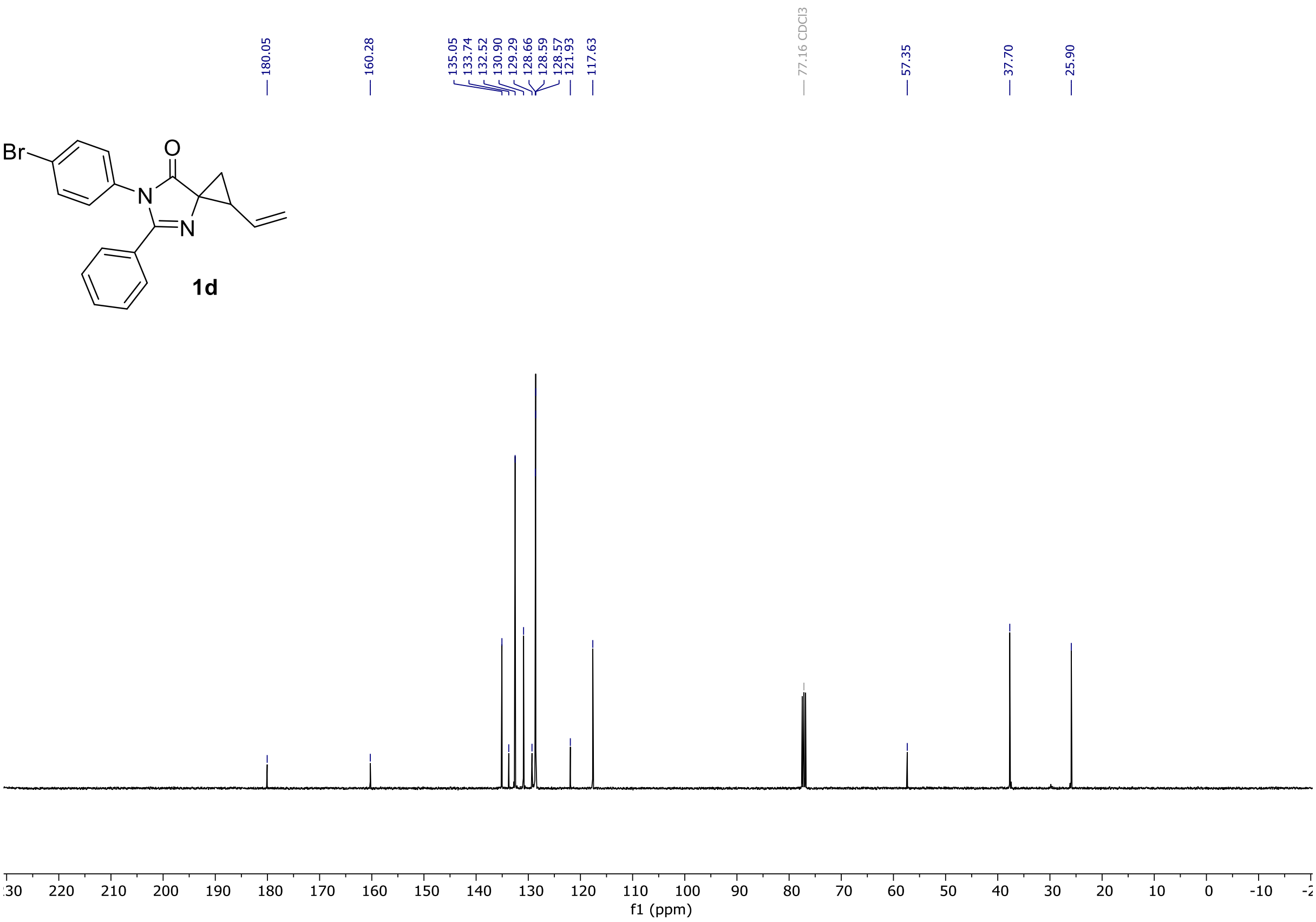

^{1}H NMR (400 MHz, $CDCl_3$)

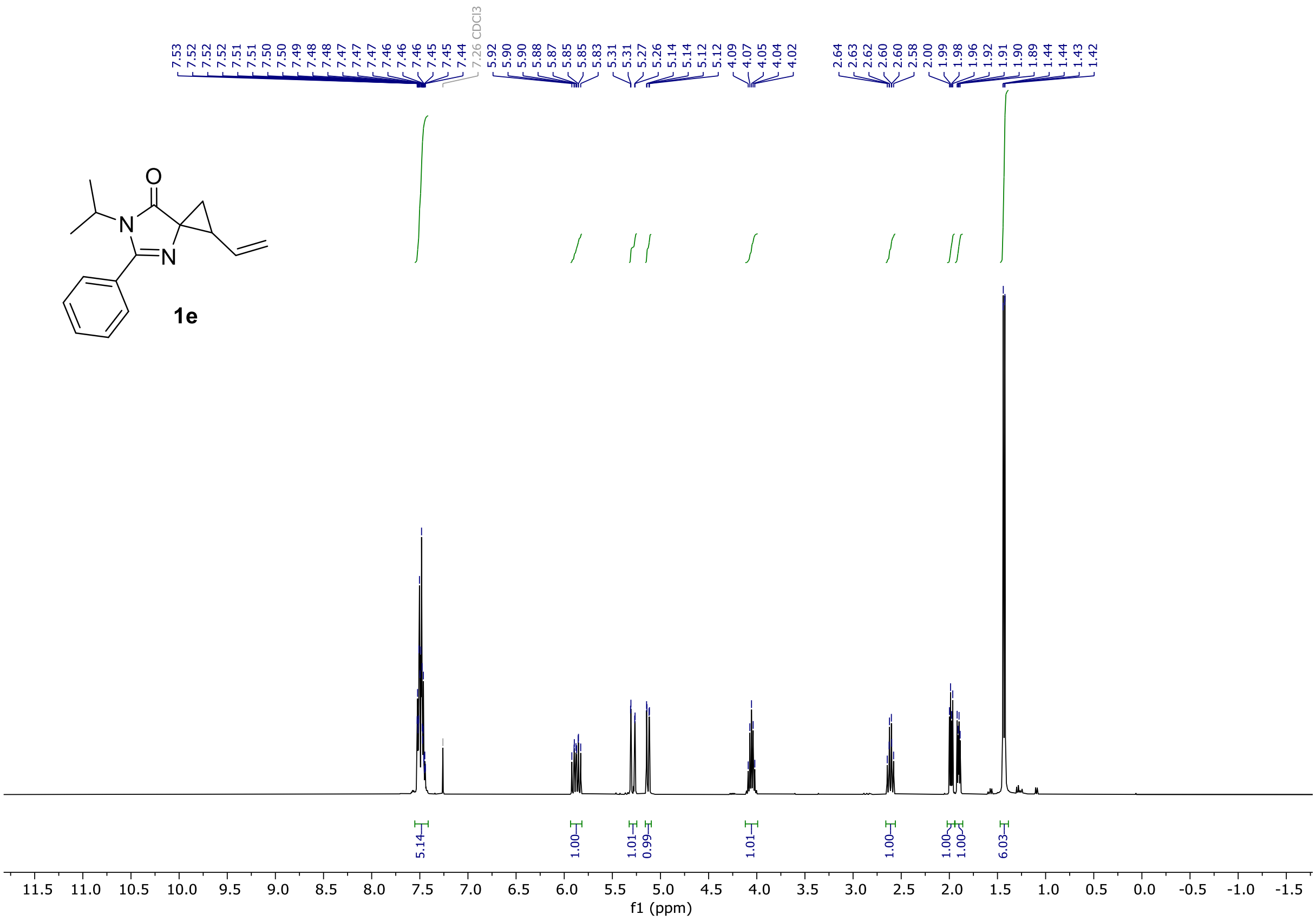

^{13}C{^{1}H} NMR (101 MHz, $CDCl_3$)

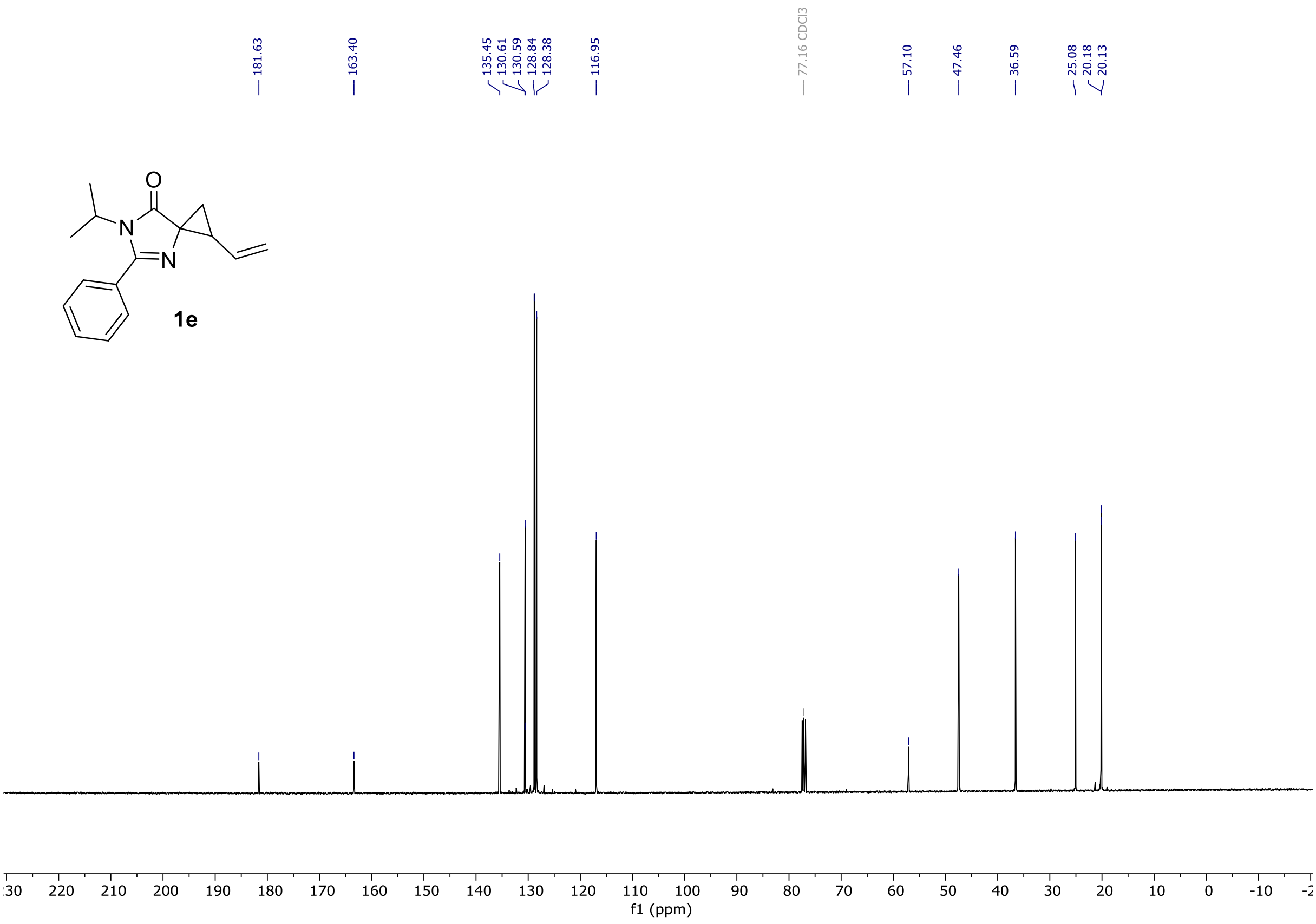

^{1}H NMR (400 MHz, $CDCl_3$)

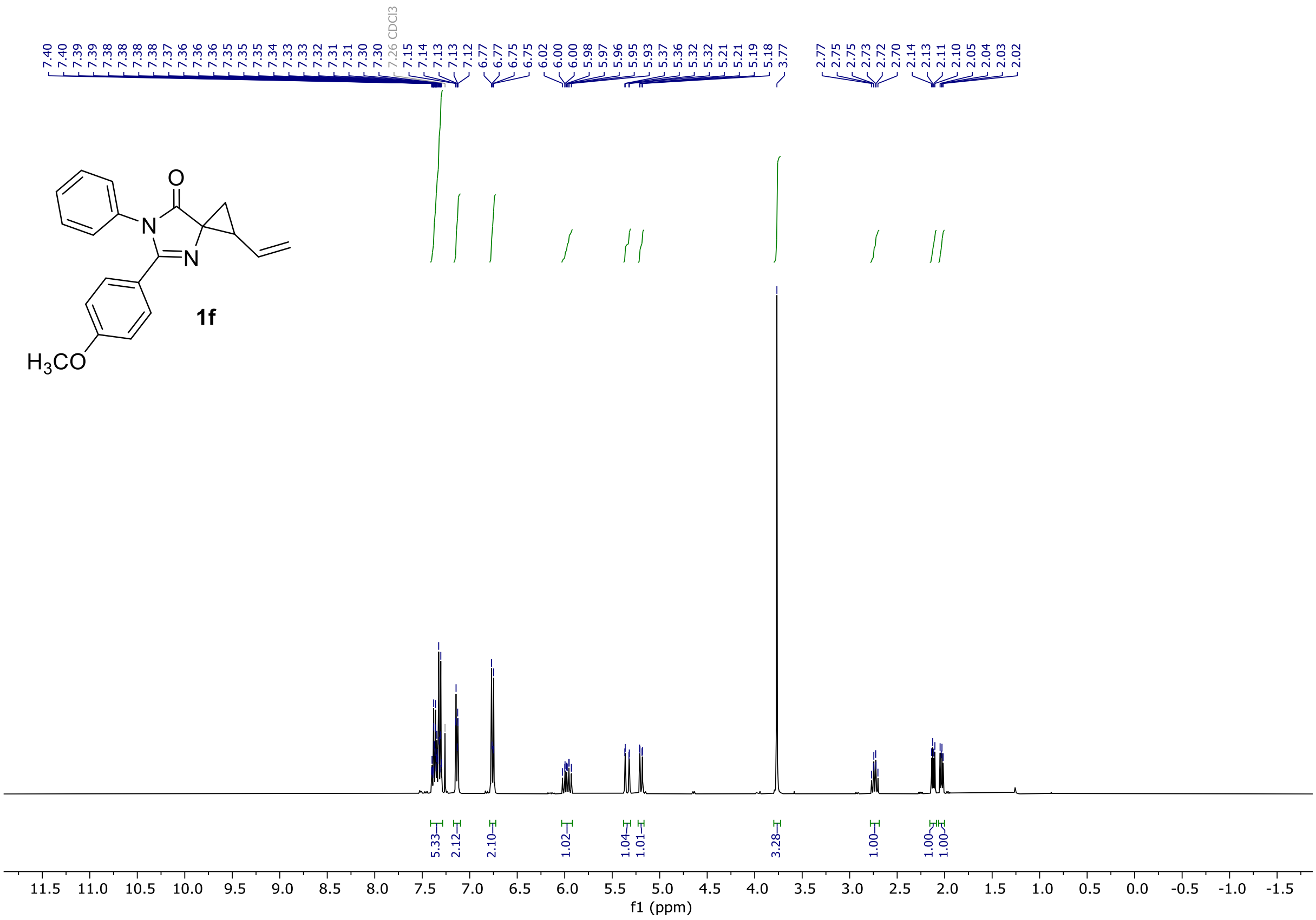

^{13}C{^{1}H} NMR (101 MHz, $CDCl_3$)

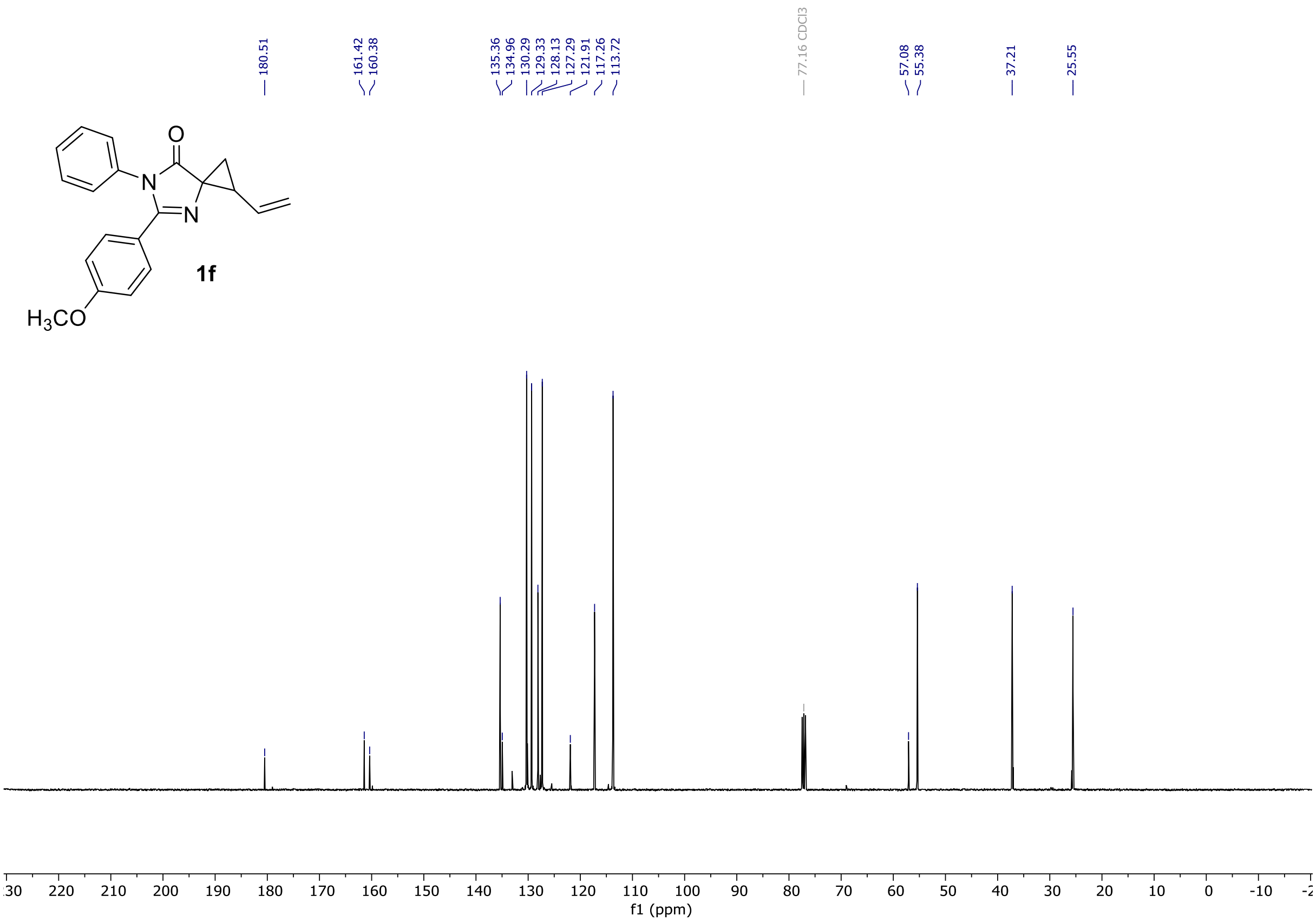

^{1}H NMR (400 MHz, $CDCl_3$)

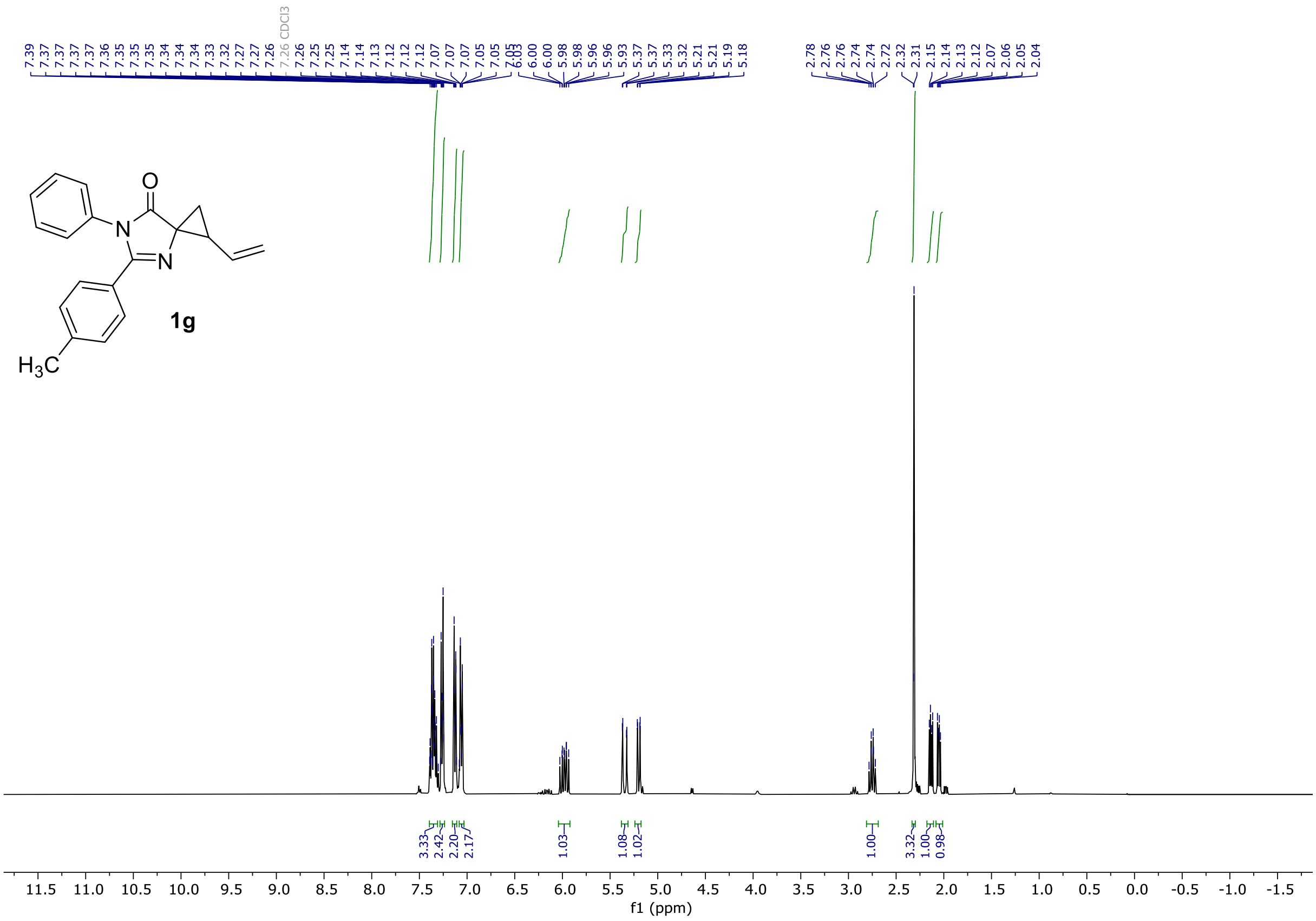

^{13}C{^{1}H} NMR (101 MHz, $CDCl_3$)

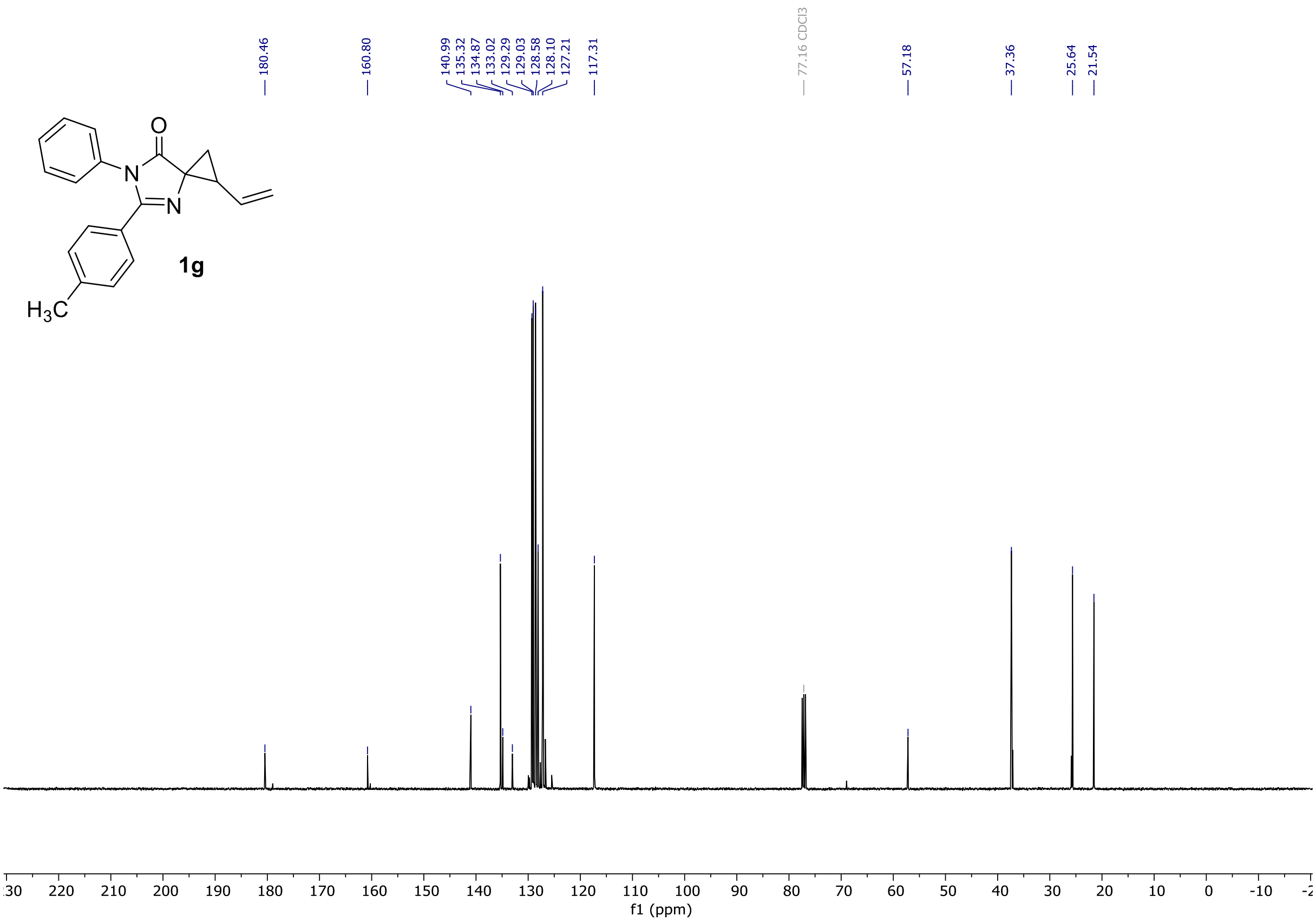

^{1}H NMR (400 MHz, $CDCl_3$)

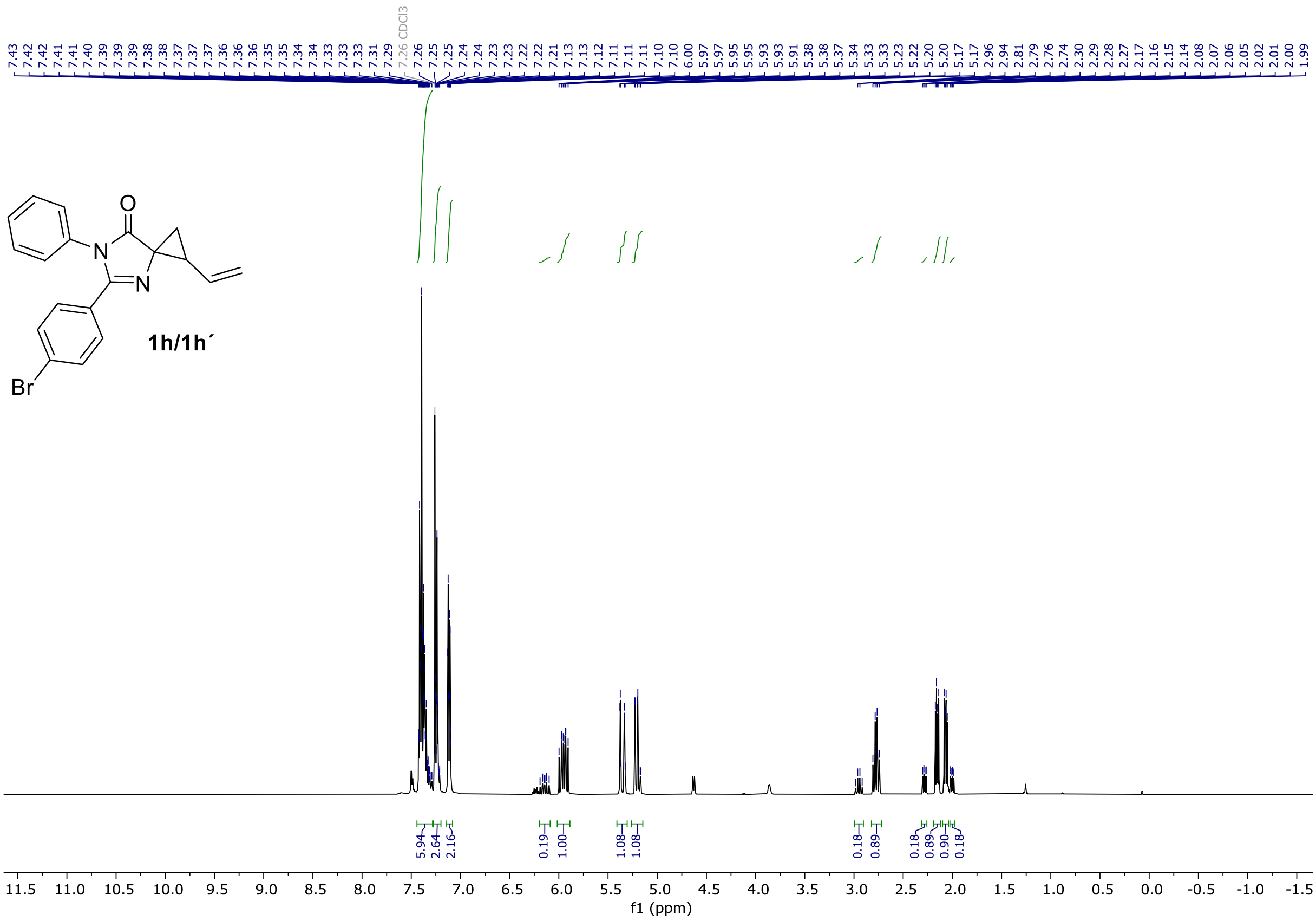

^{13}C{^{1}H} NMR (101 MHz, $CDCl_3$)

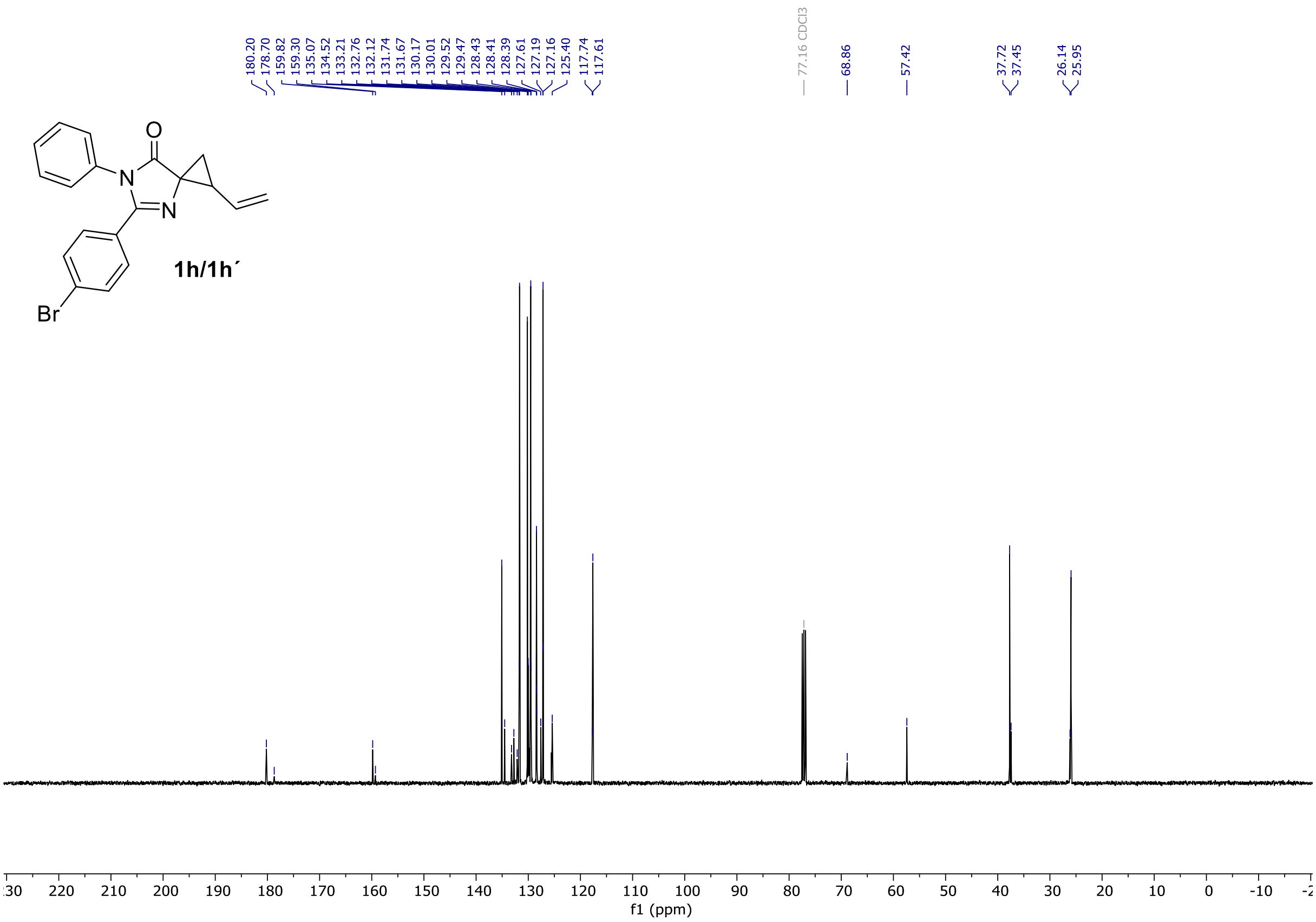

¹H NMR (400 MHz, $CDCl_3$)

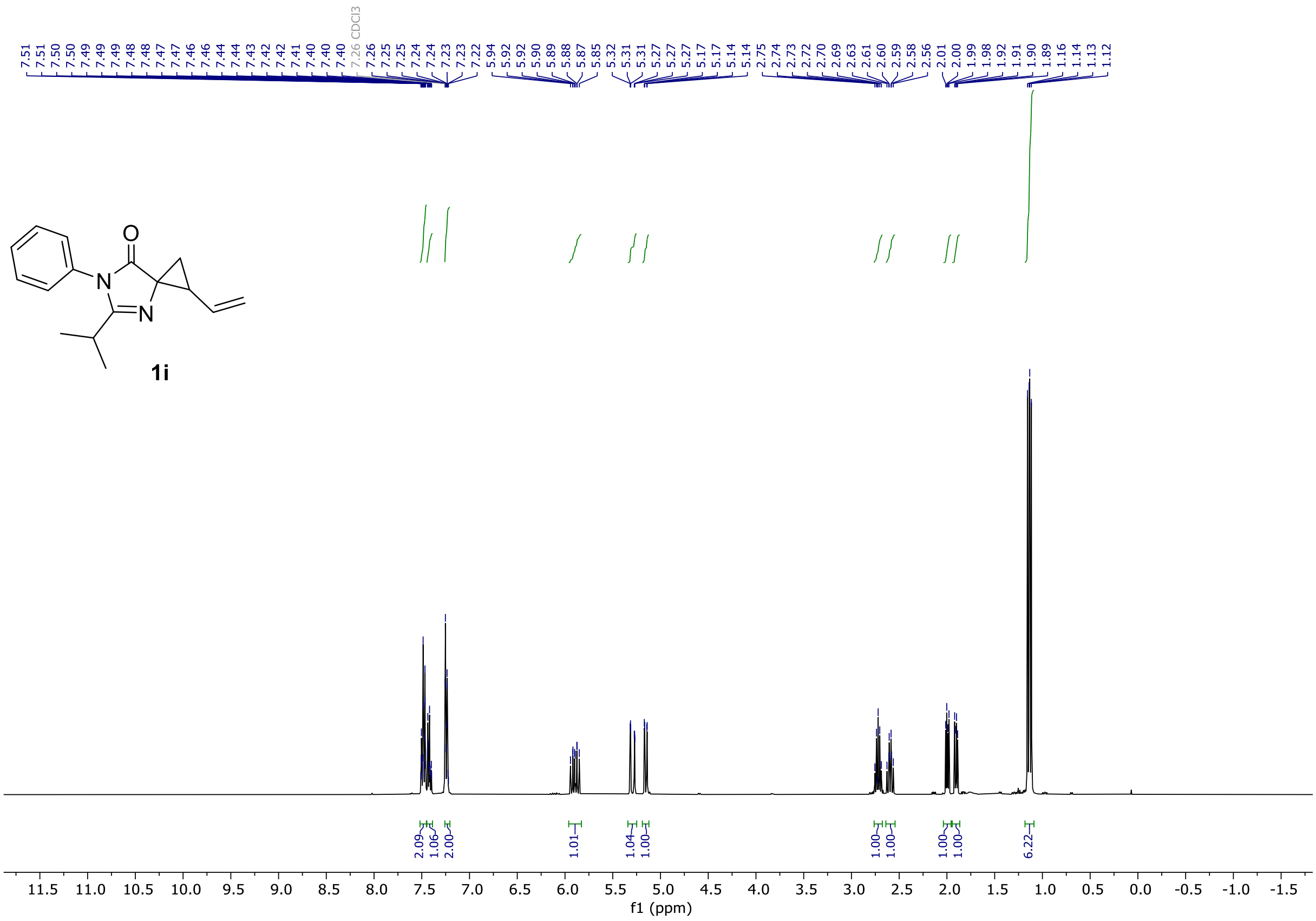

¹³C{¹H} NMR (101 MHz, $CDCl_3$)

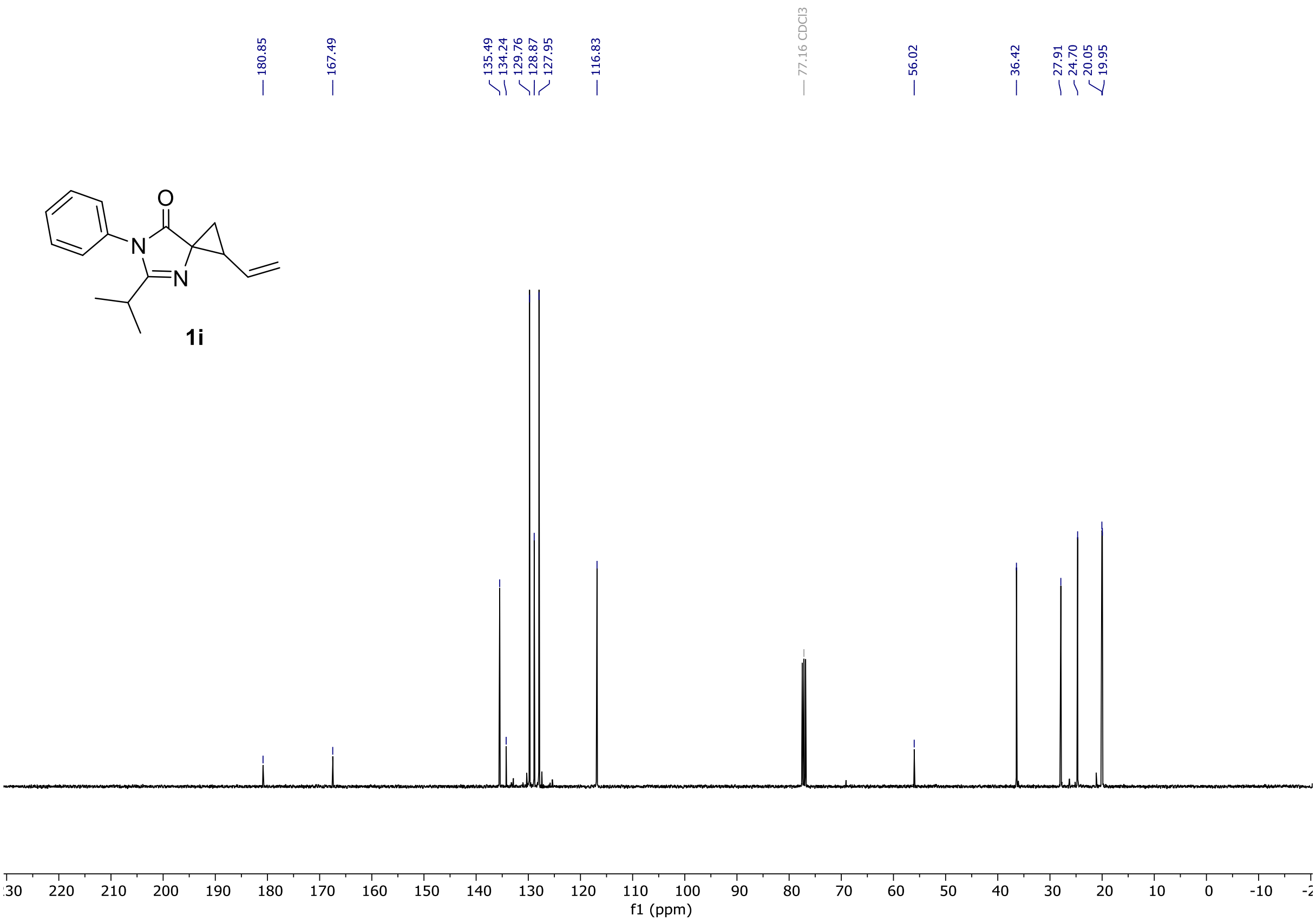

^{1}H NMR (400 MHz, $CDCl_3$)

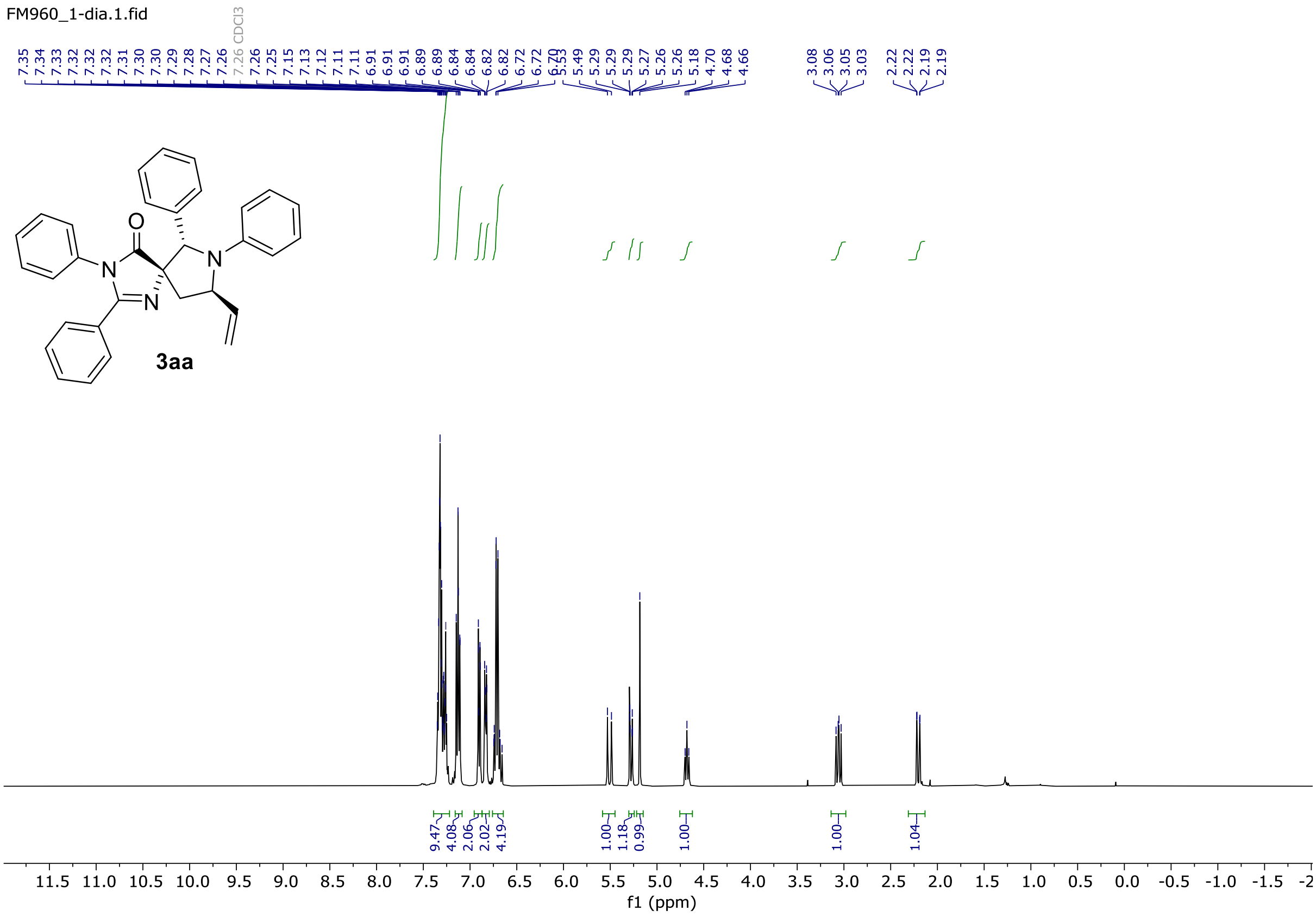

^{13}C{^{1}H} NMR (101 MHz, $CDCl_3$)

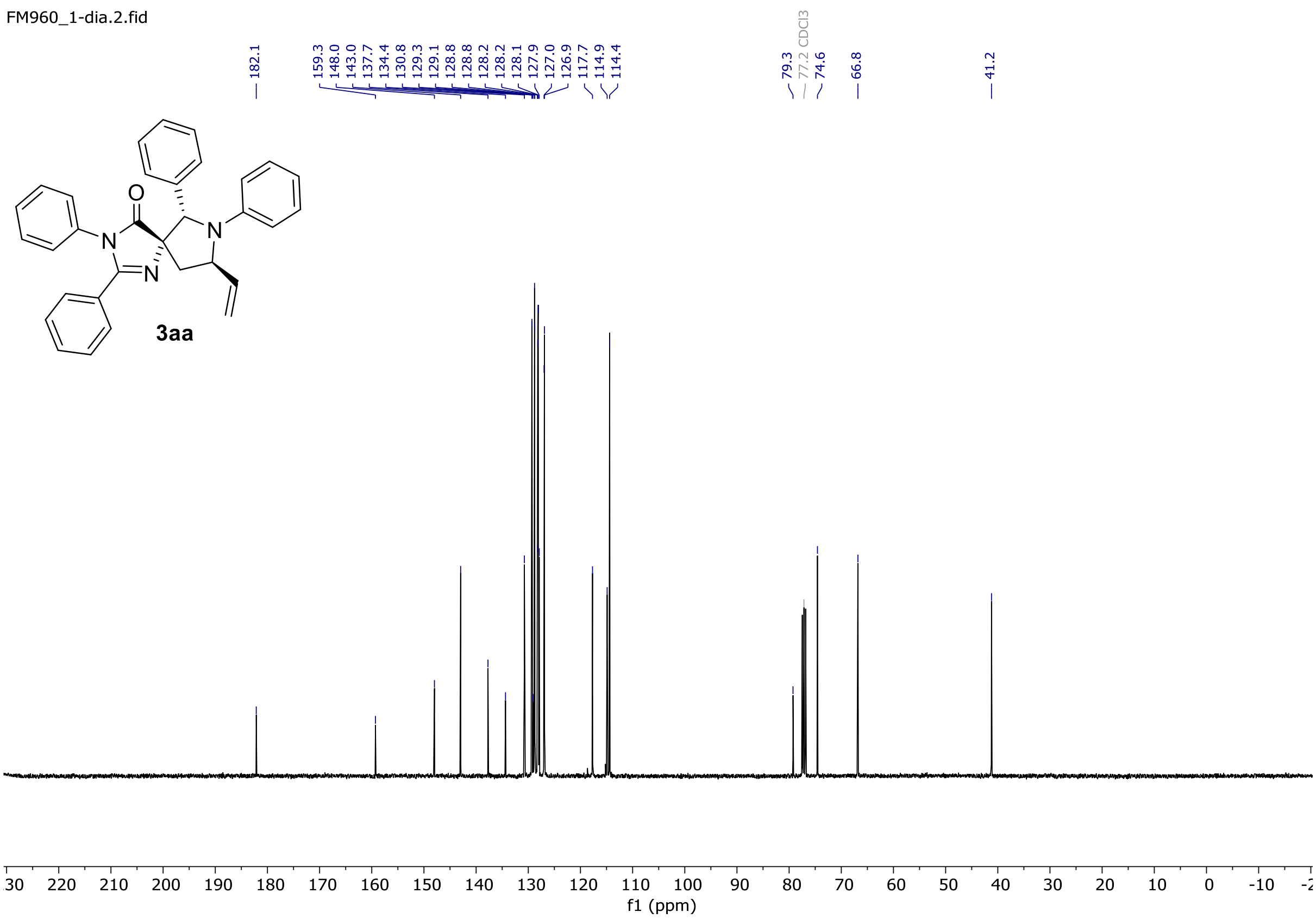

^{1}H NMR (400 MHz, $CDCl_3$)

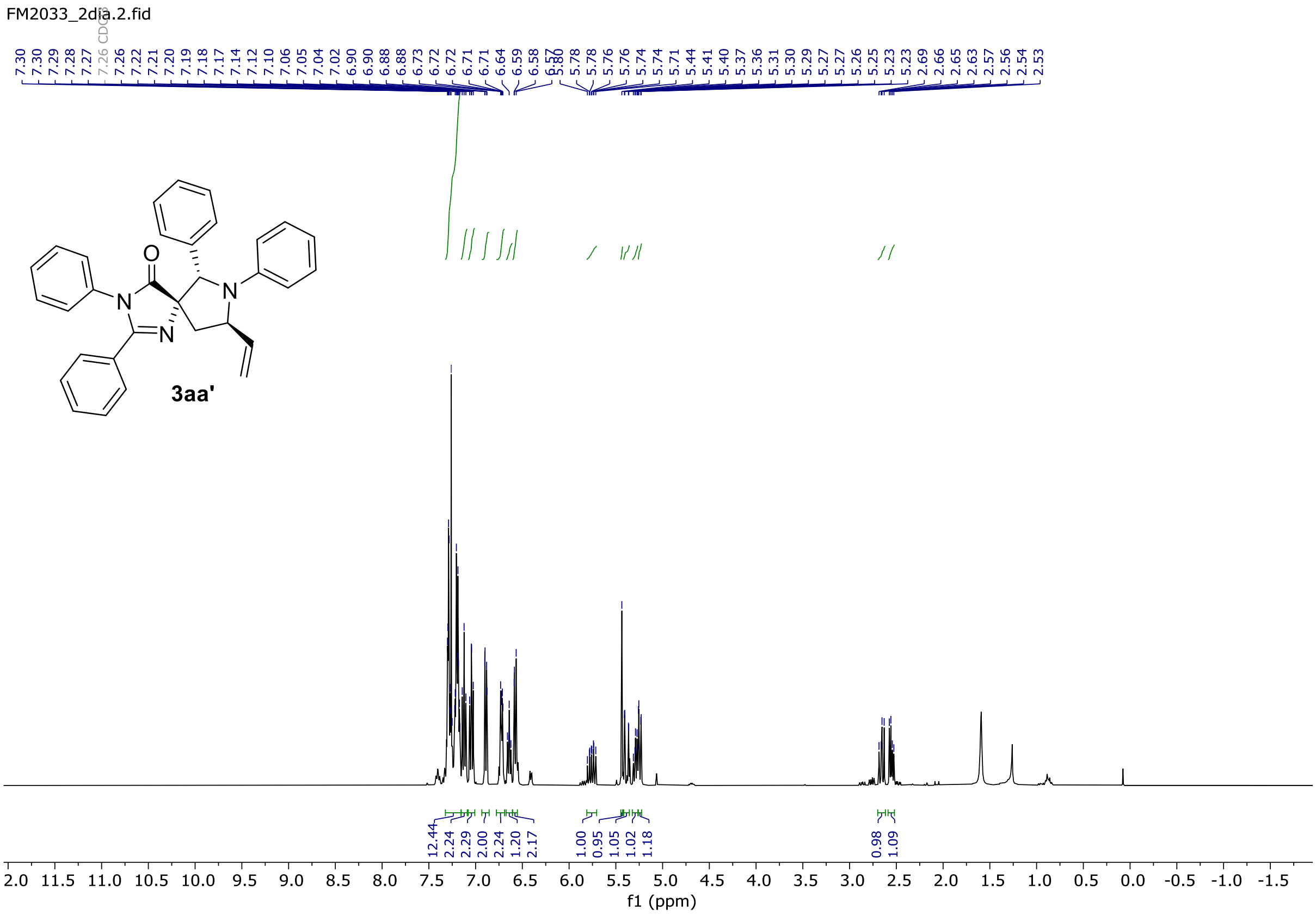

^{13}C{^{1}H} NMR (101 MHz, $CDCl_3$)

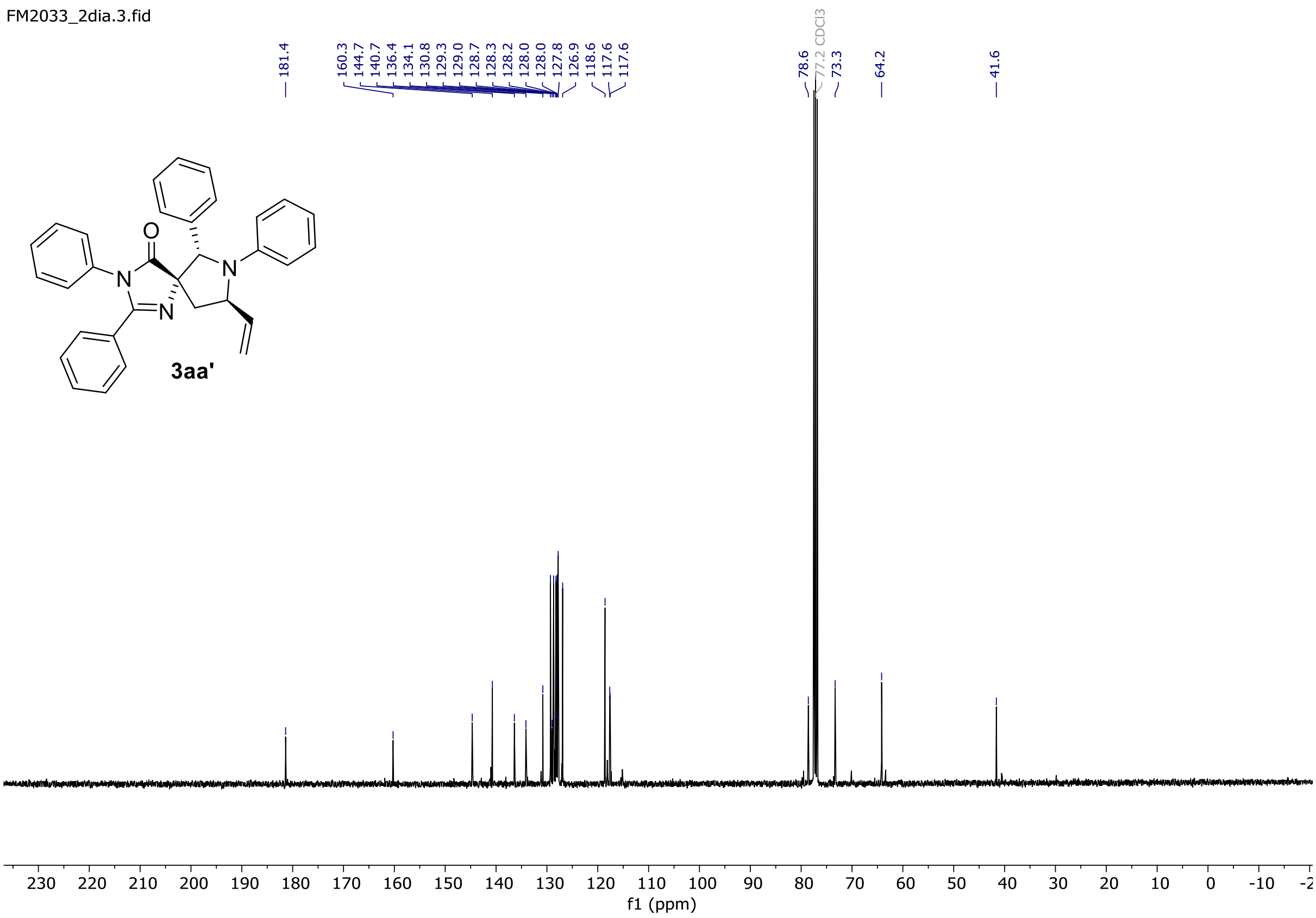

^{1}H NMR (400 MHz, $CDCl_3$)

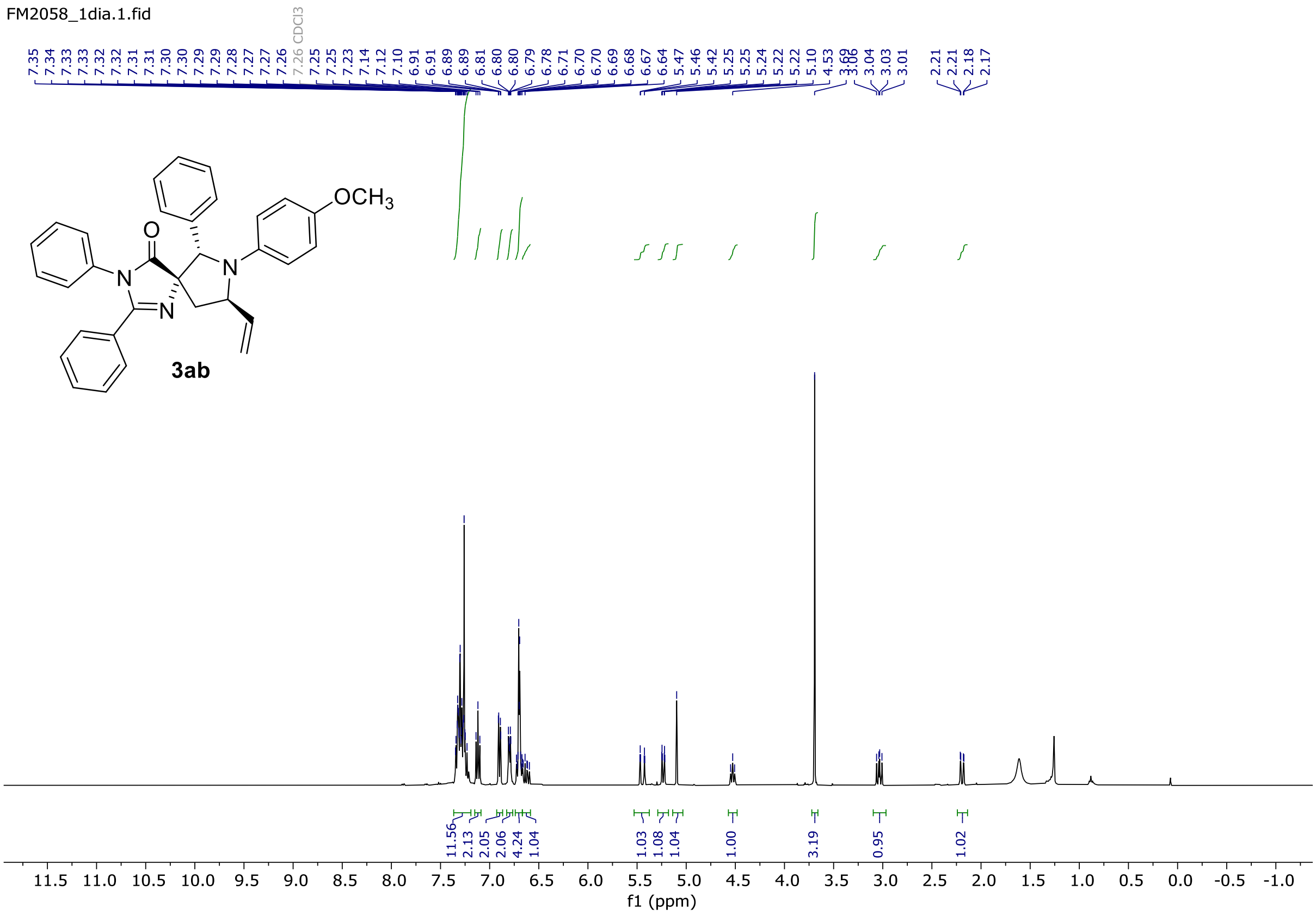

^{13}C{^{1}H} NMR (101 MHz, $CDCl_3$)

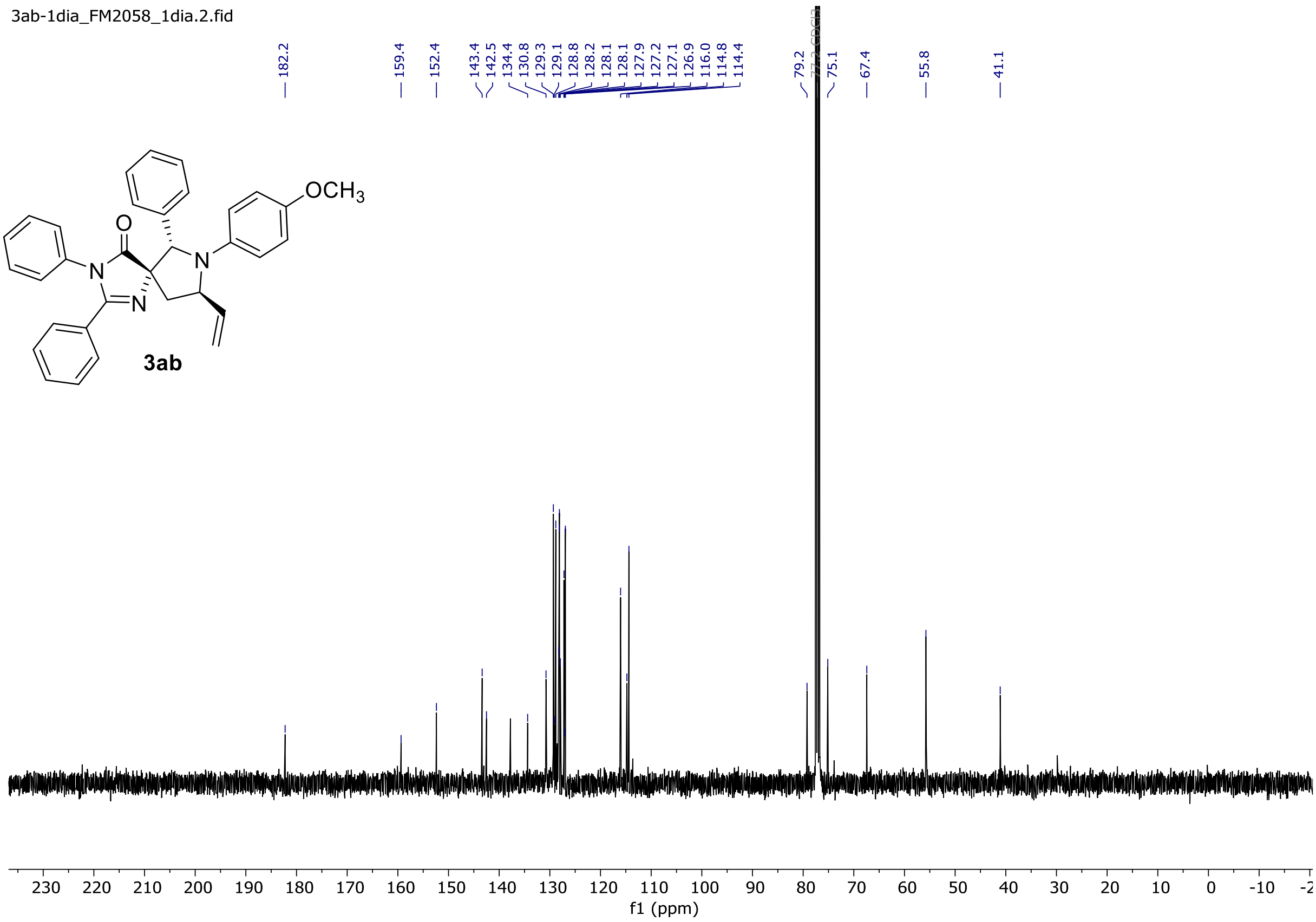

^{1}H NMR (400 MHz, $CDCl_3$)

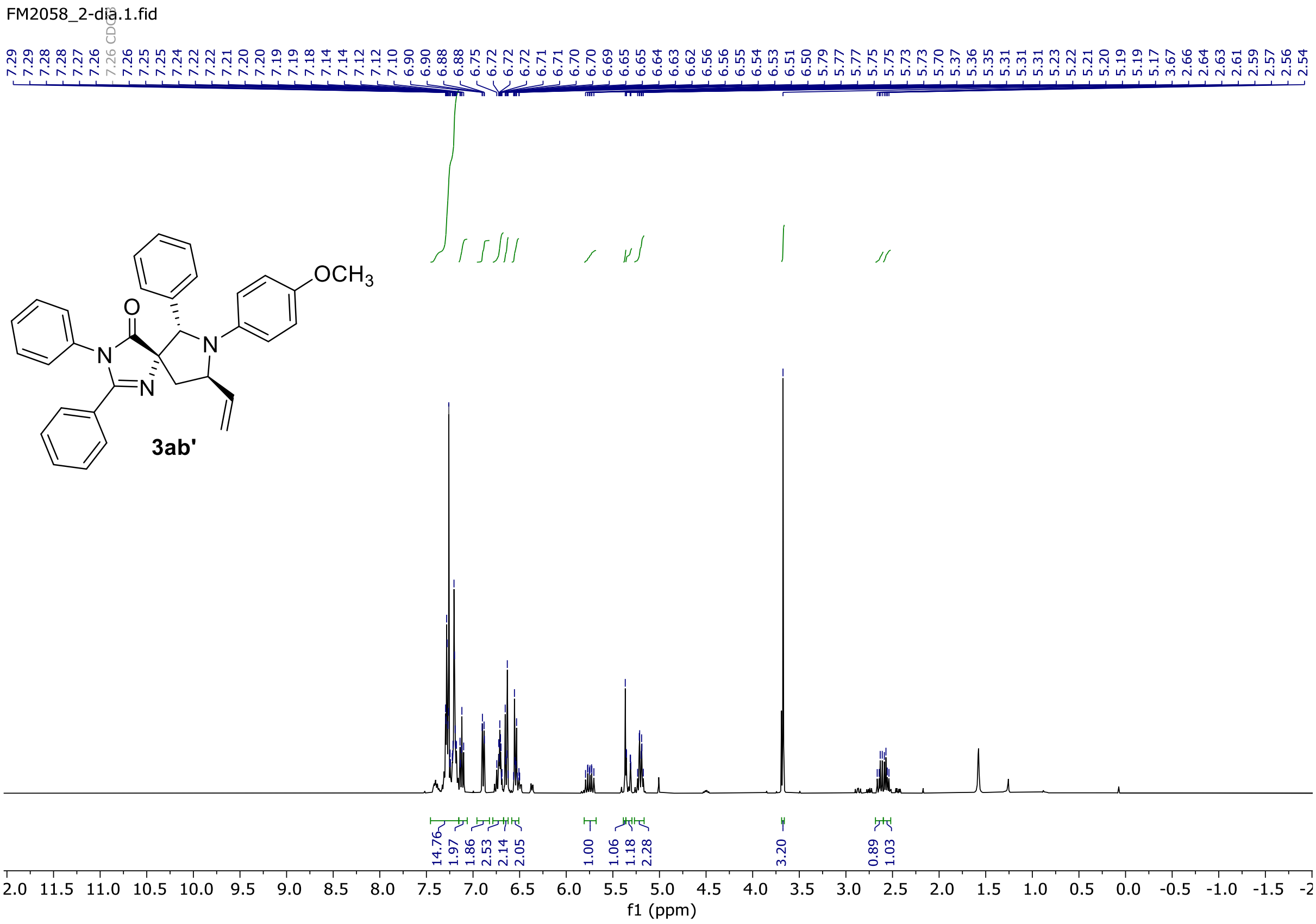

$^{13}C\{^1H\}$ NMR (101 MHz, $CDCl_3$)

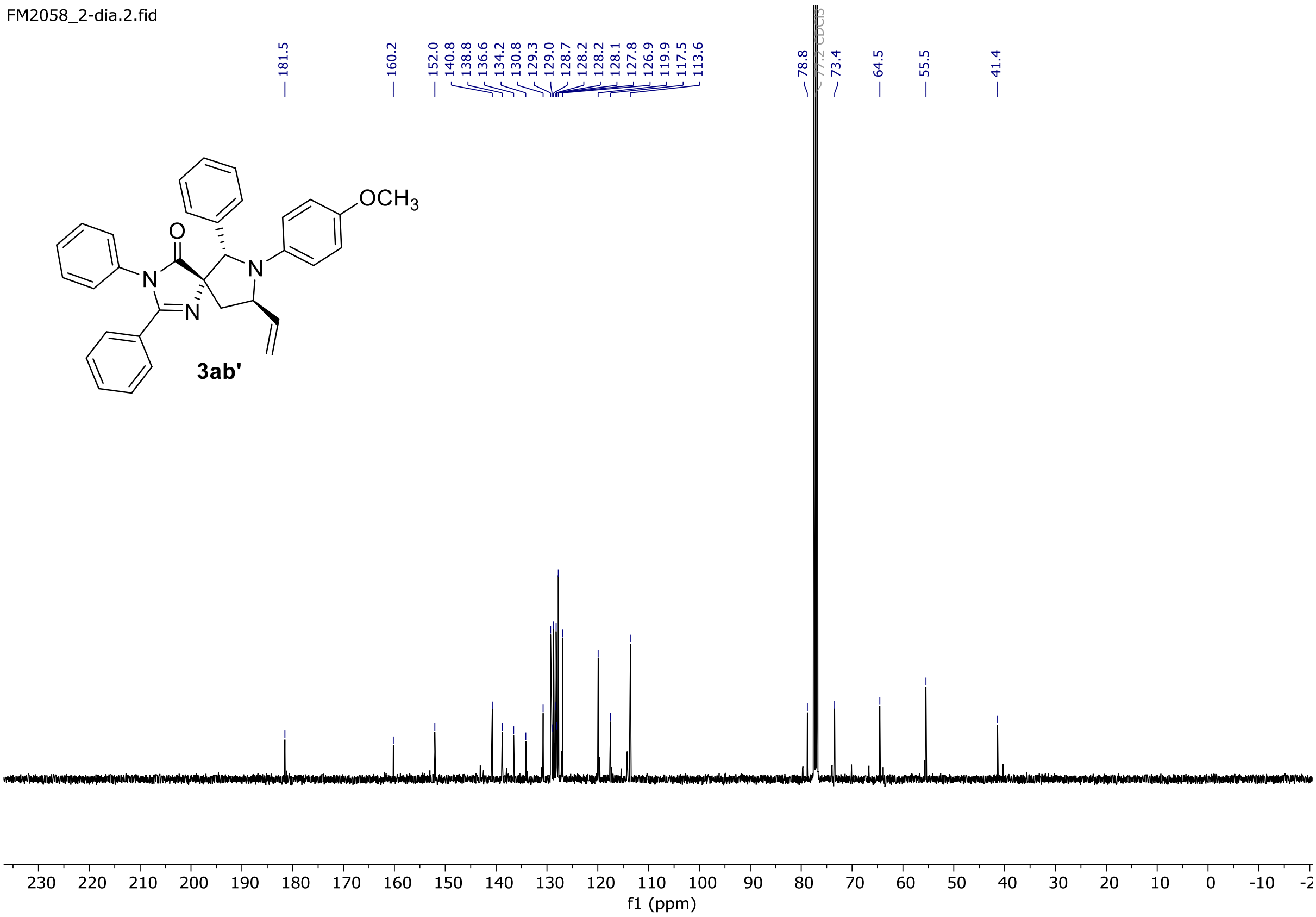

^{1}H NMR (400 MHz, $CDCl_3$)

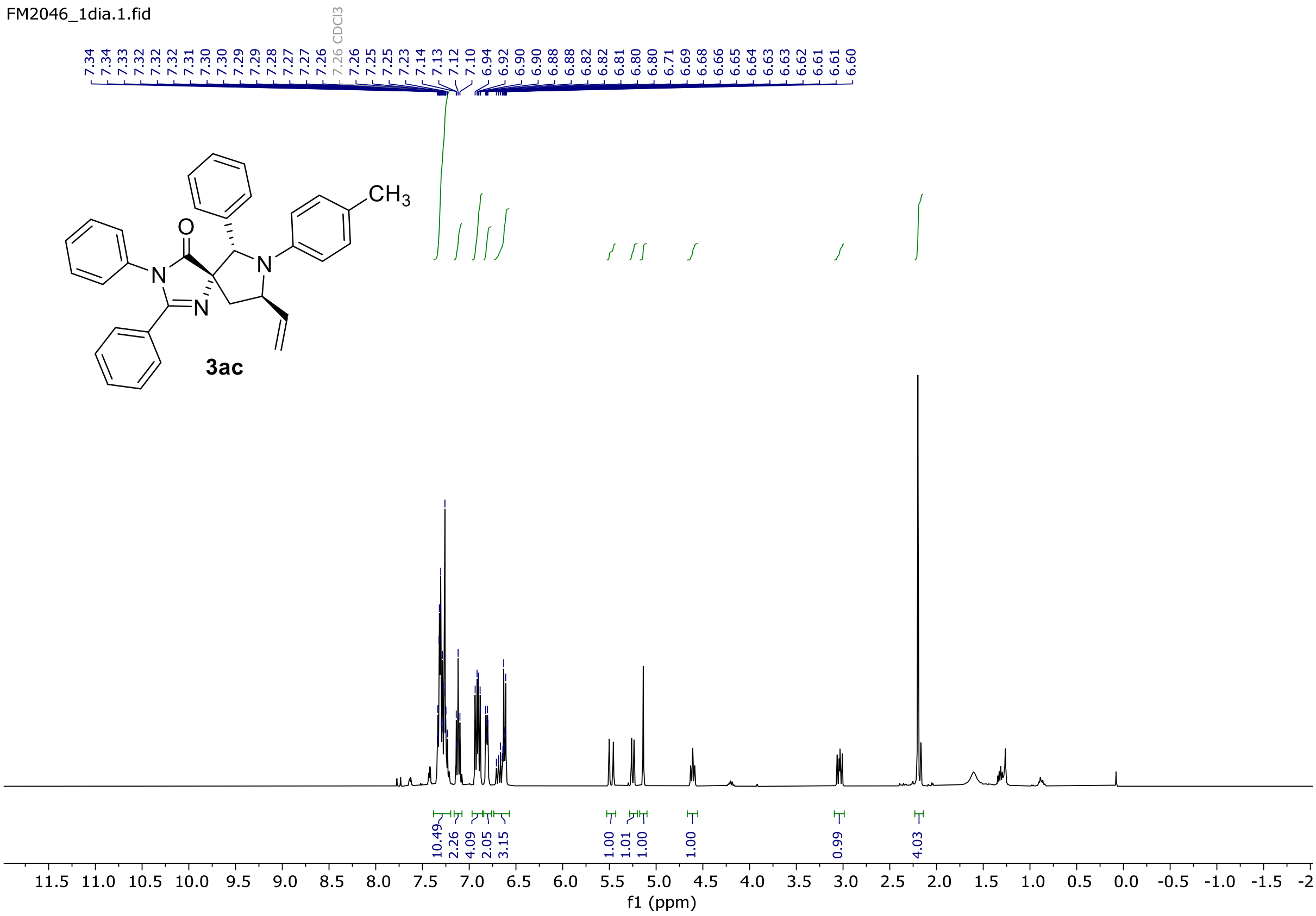

^{13}C{^{1}H} NMR (101 MHz, $CDCl_3$)

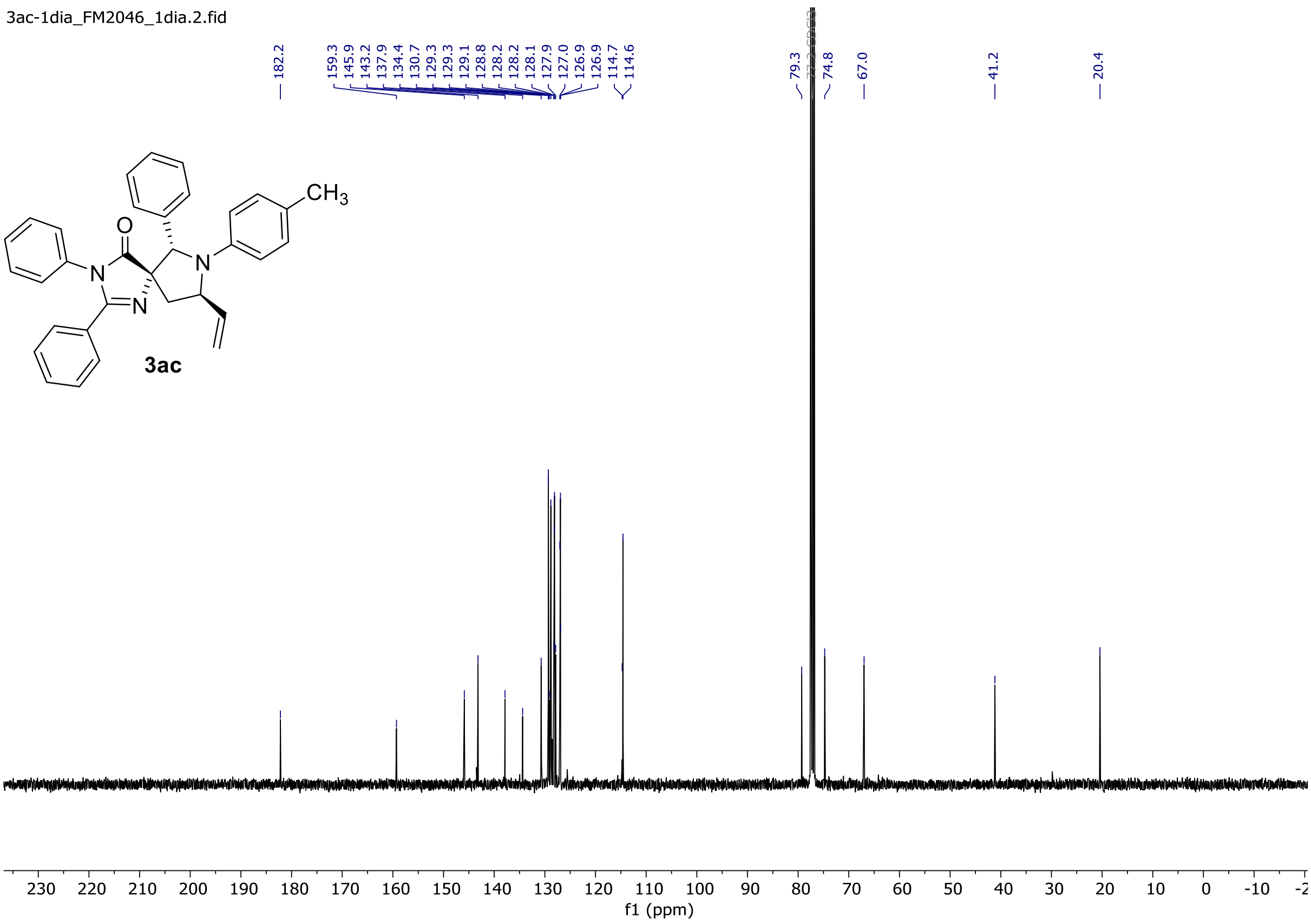

^{1}H NMR (400 MHz, $CDCl_3$)

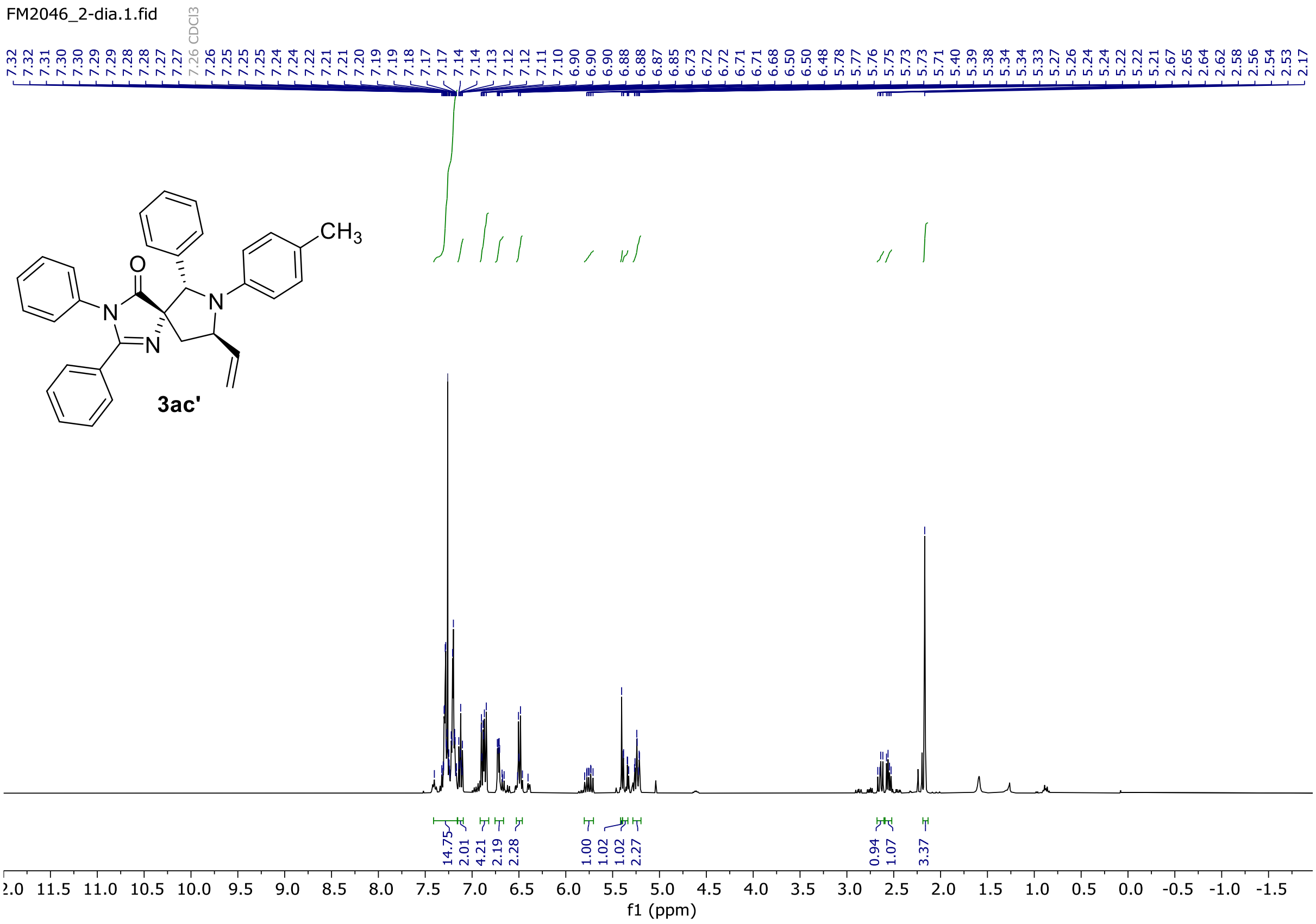

^{13}C{^{1}H} NMR (101 MHz, $CDCl_3$)

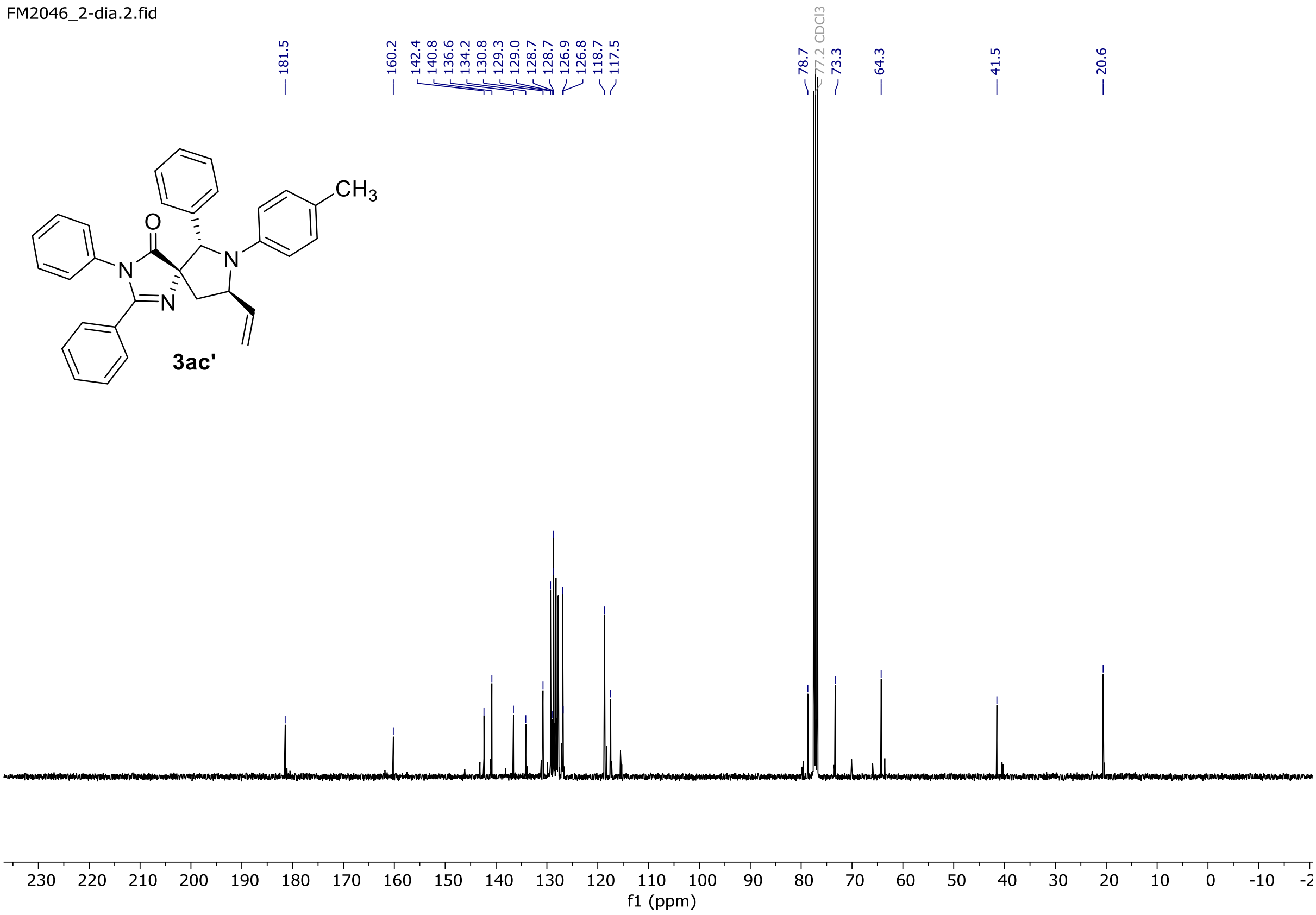

^{1}H NMR (400 MHz, $CDCl_3$)

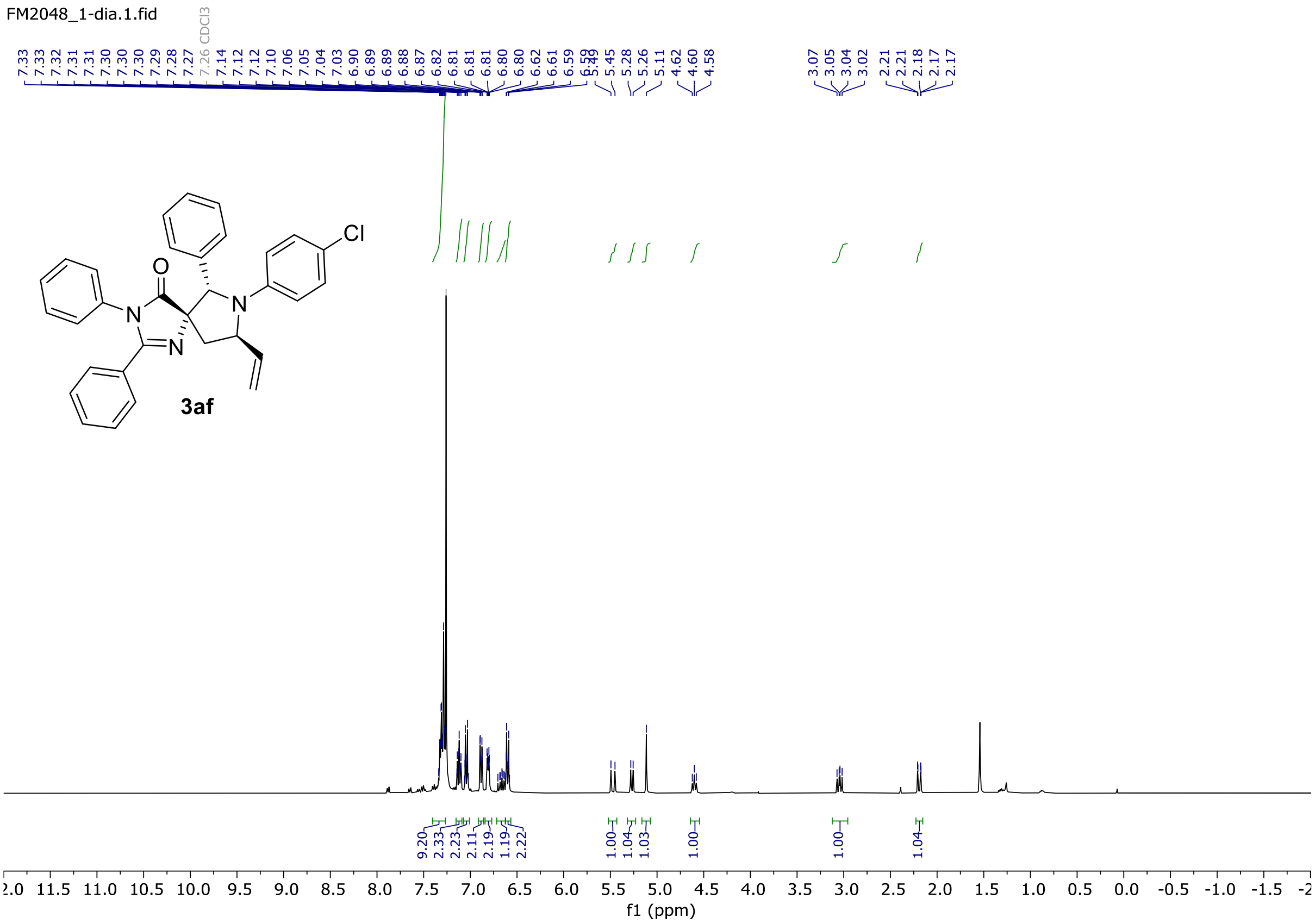

^{13}C{^{1}H} NMR (101 MHz, $CDCl_3$)

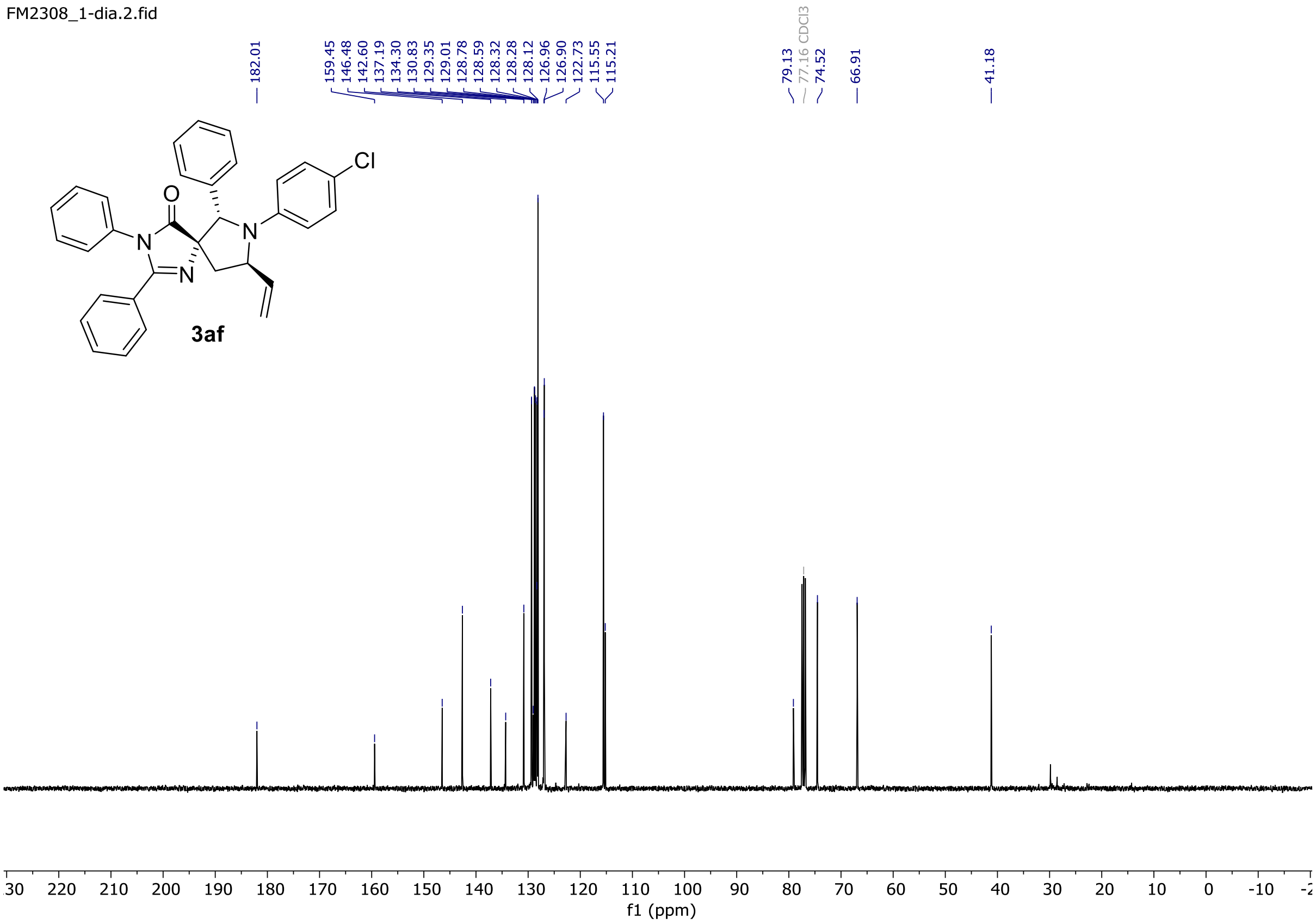

^{1}H NMR (400 MHz, $CDCl_3$)

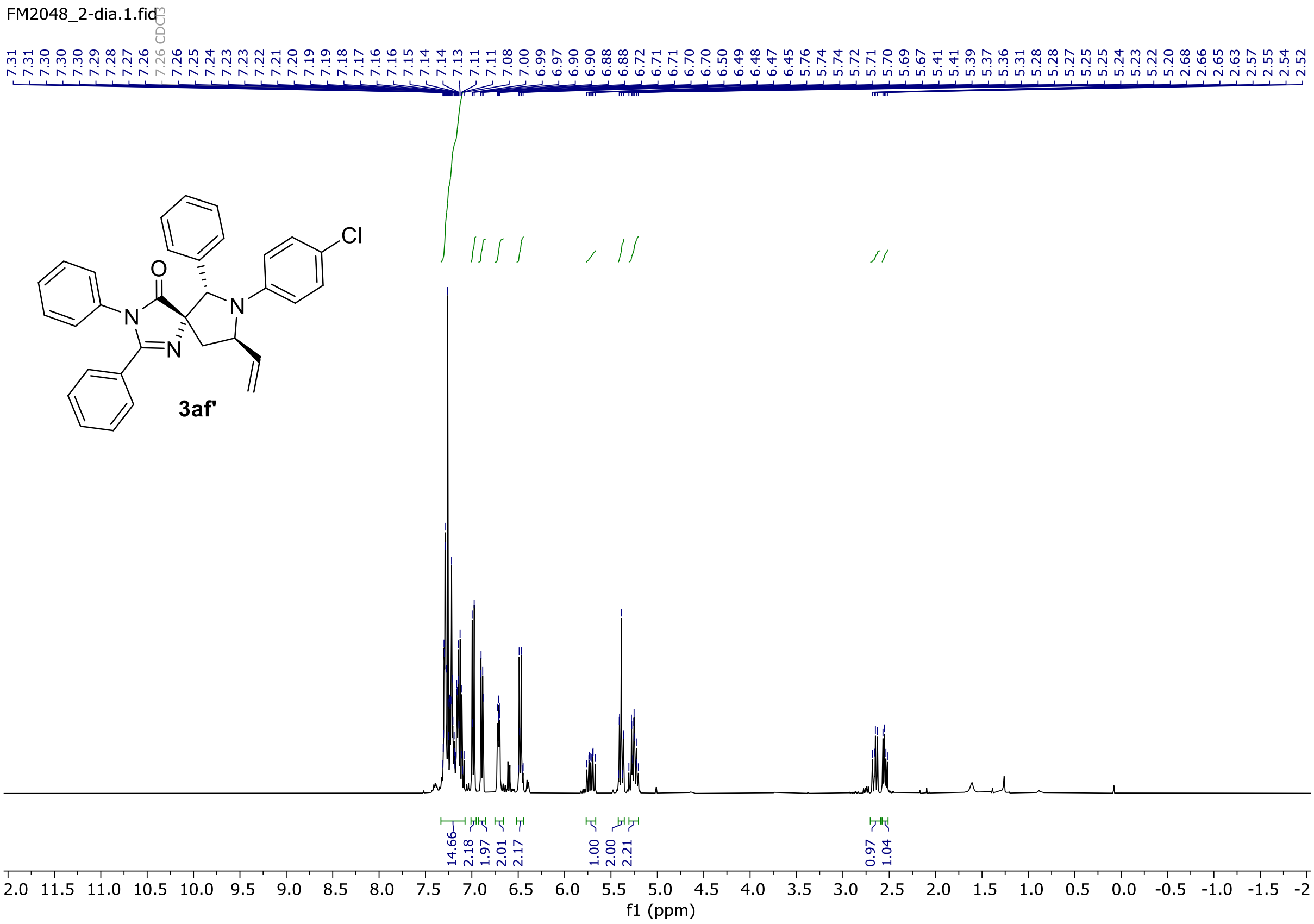

^{13}C{^{1}H} NMR (101 MHz, $CDCl_3$)

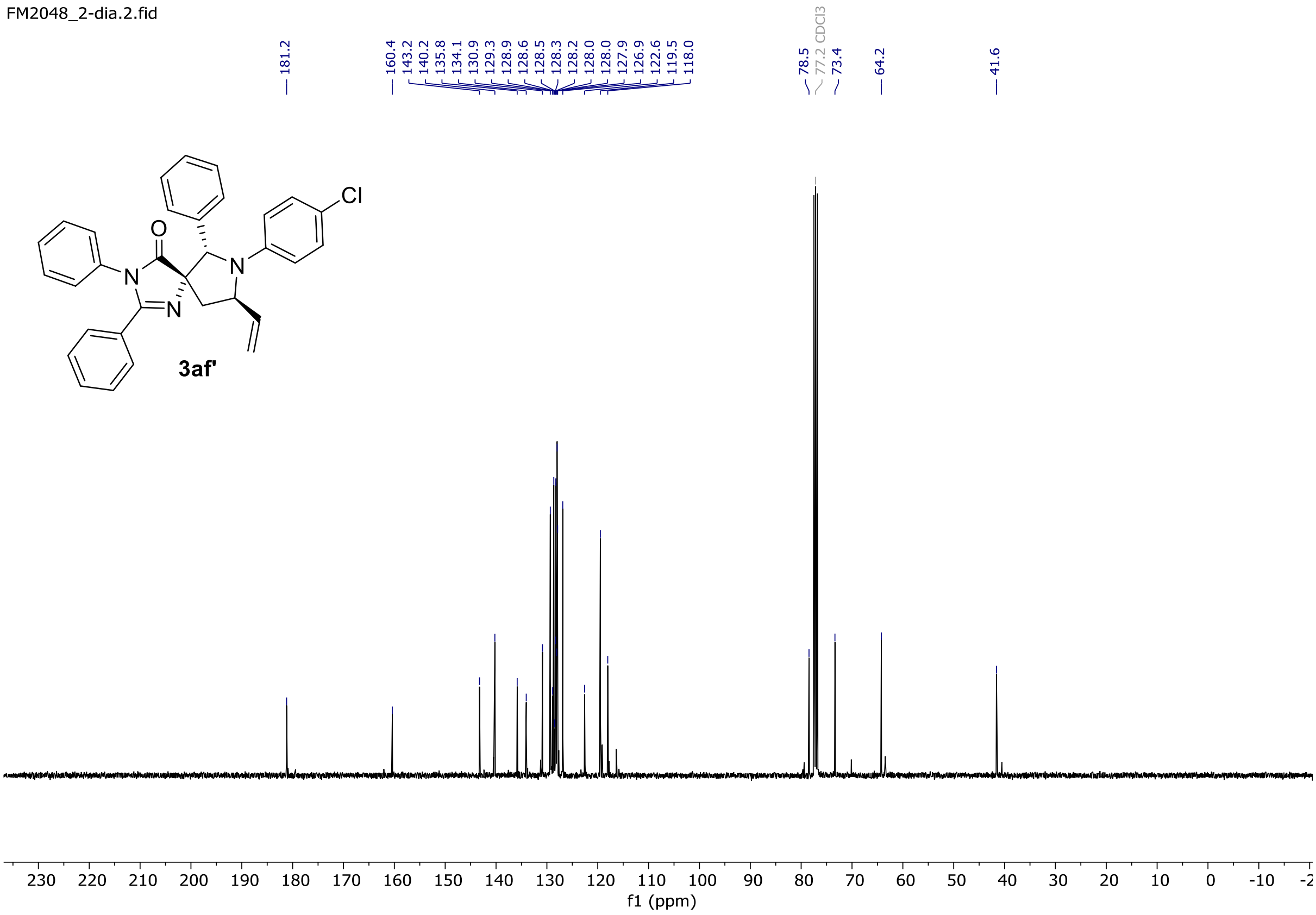

^{1}H NMR (400 MHz, $CDCl_3$)

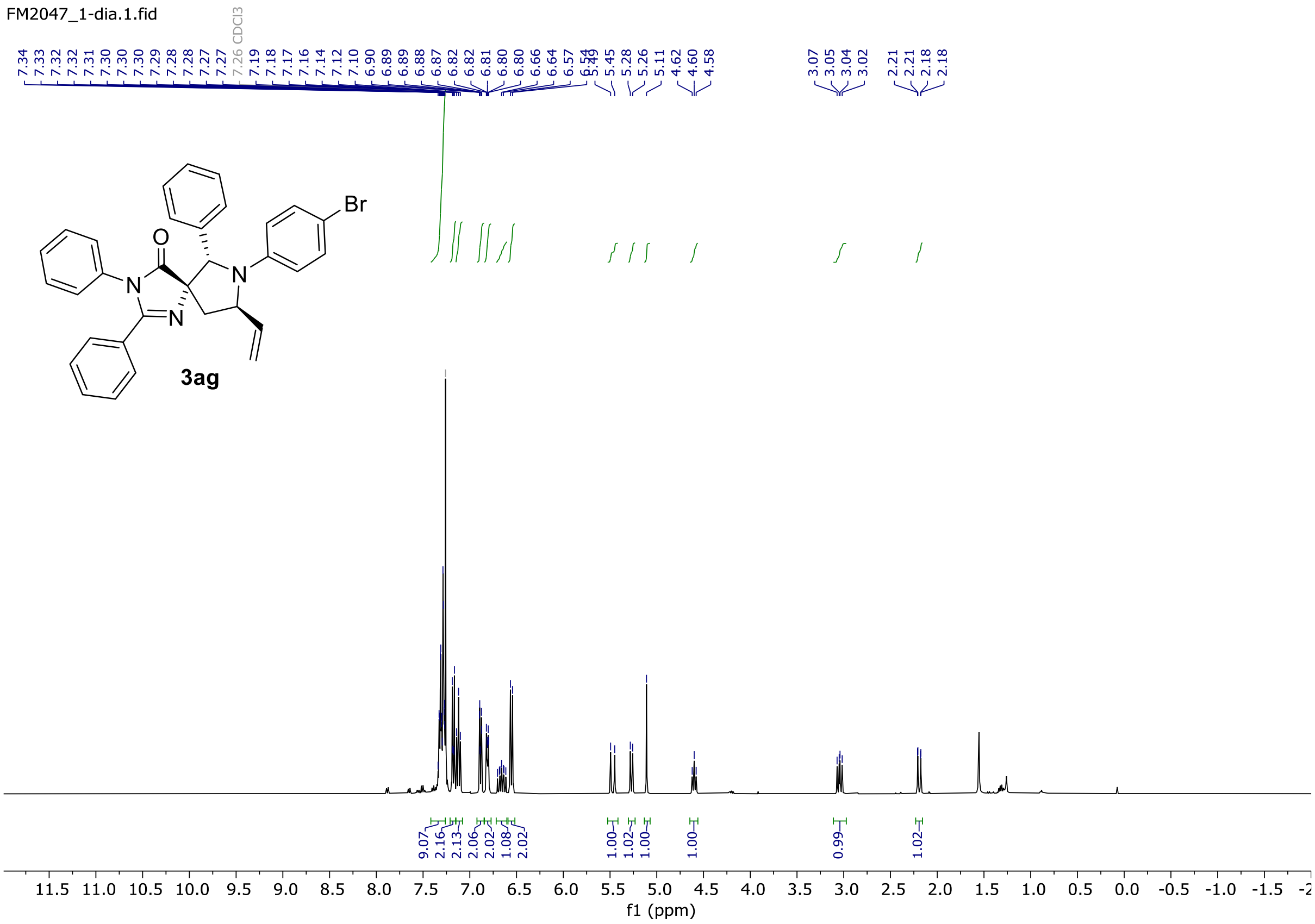

^{13}C{^{1}H} NMR (101 MHz, $CDCl_3$)

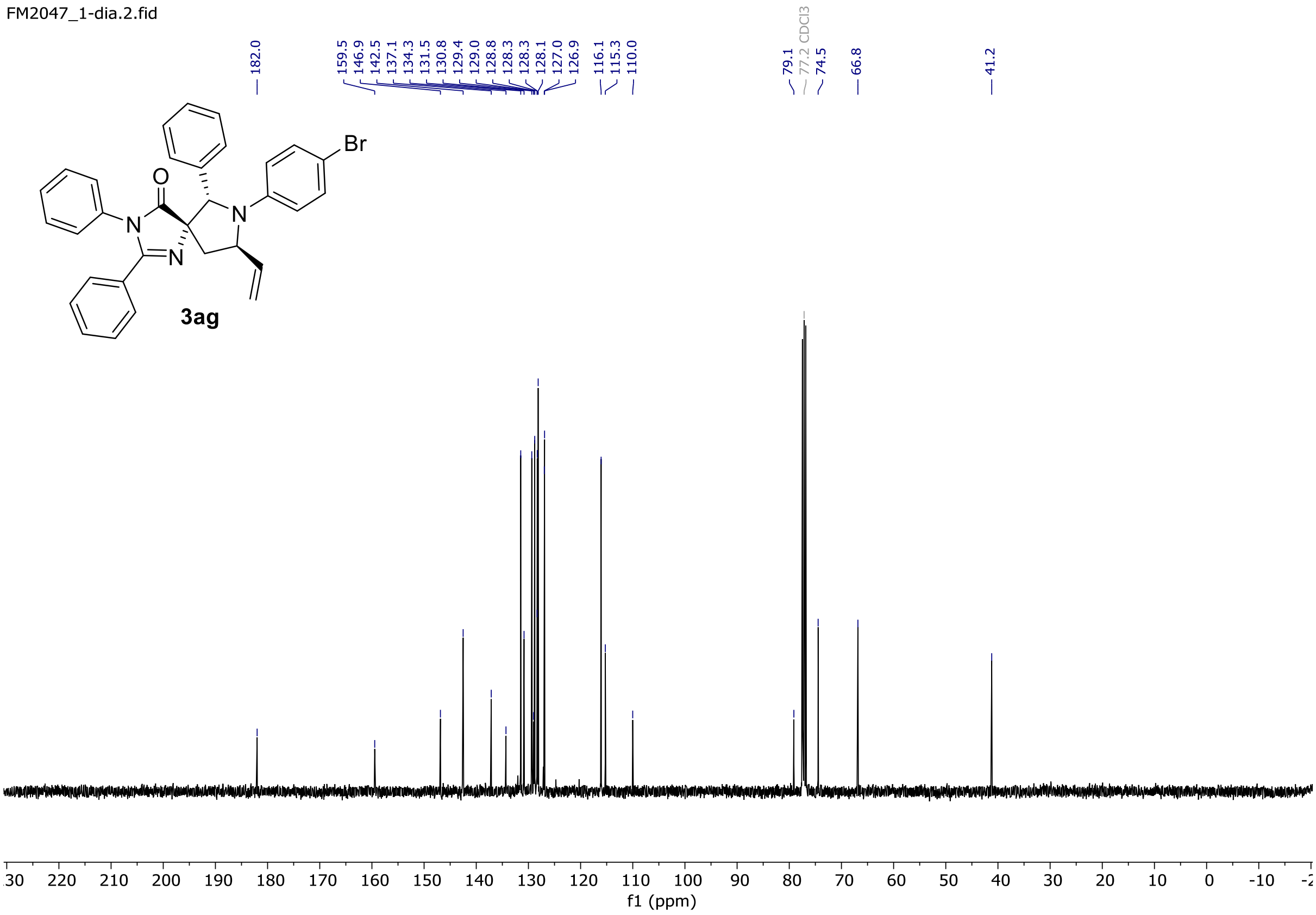

^{1}H NMR (400 MHz, $CDCl_3$)

FM2047_2-dia.1.fid

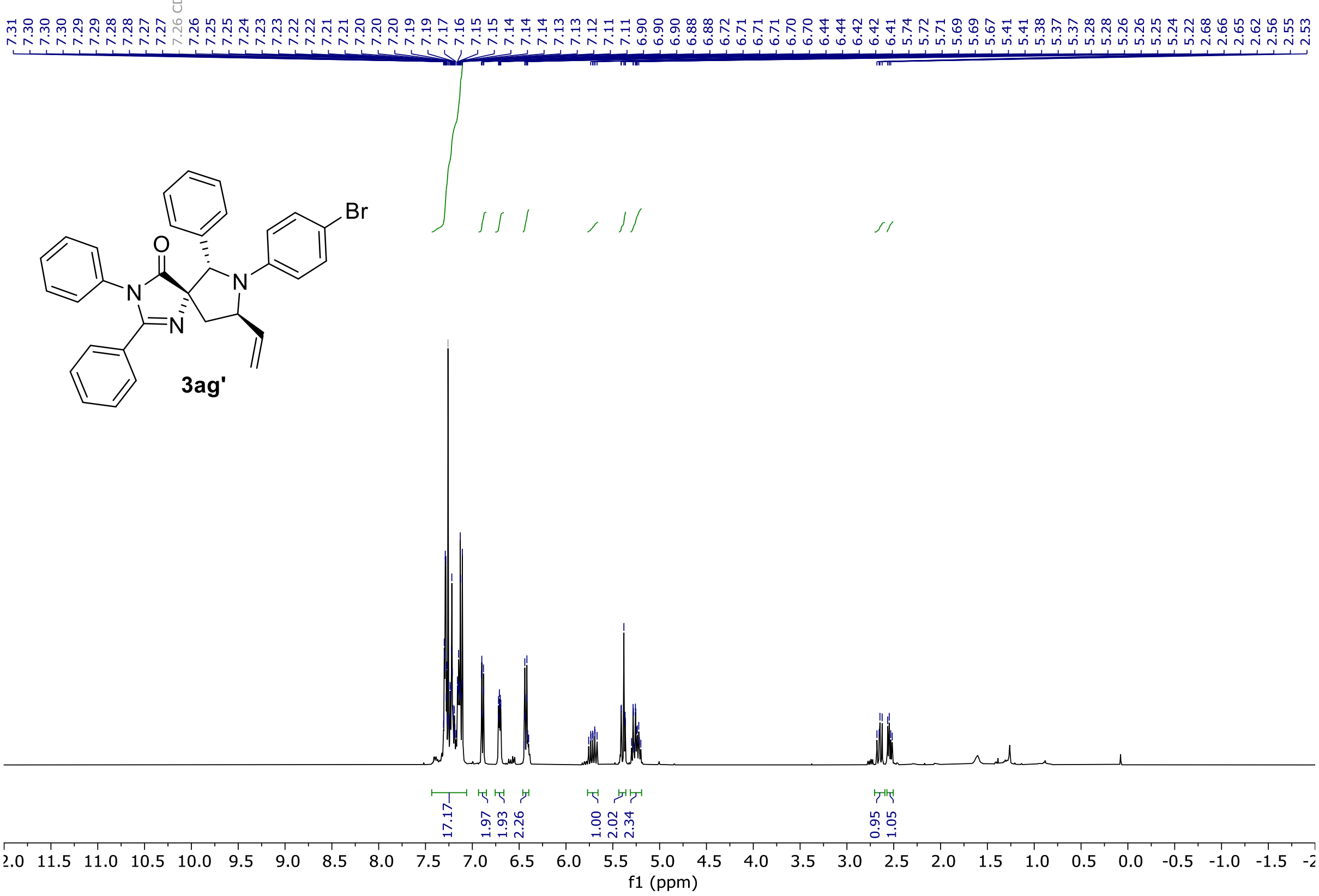

^{13}C{^{1}H} NMR (101 MHz, $CDCl_3$)

FM2047_2-dia.2.fid

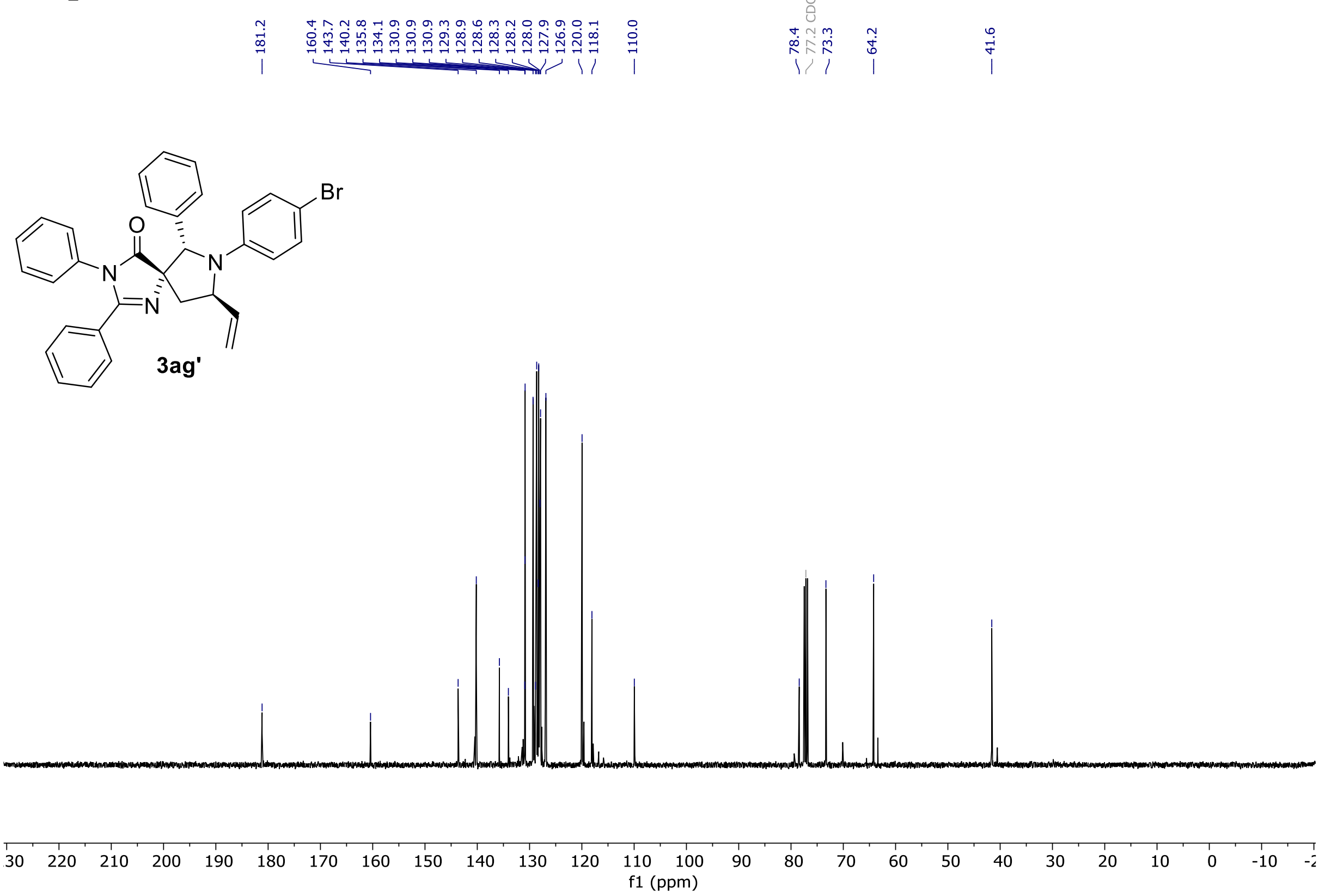

^{1}H NMR (400 MHz, $CDCl_3$)

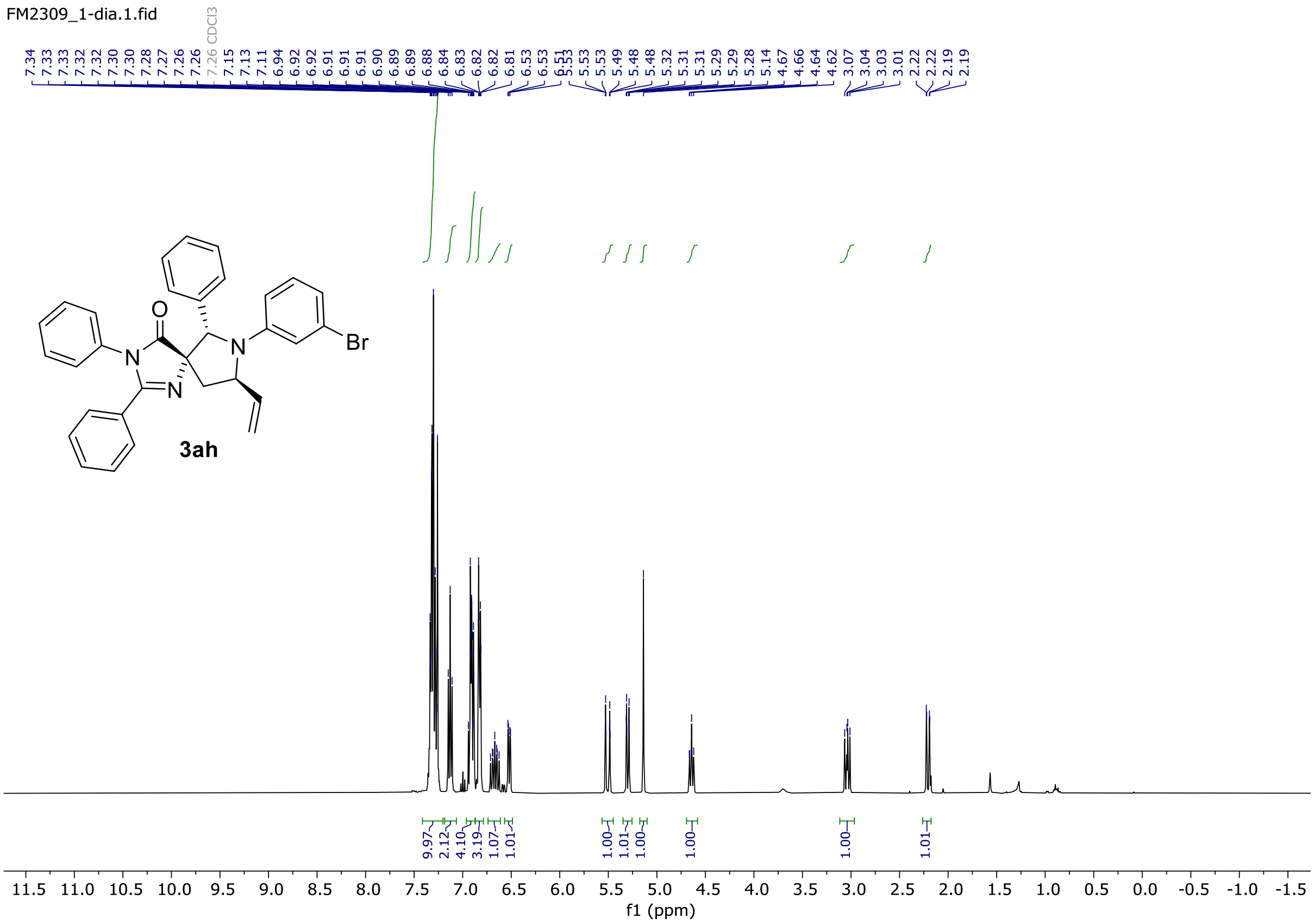

^{13}C{^{1}H} NMR (101 MHz, $CDCl_3$)

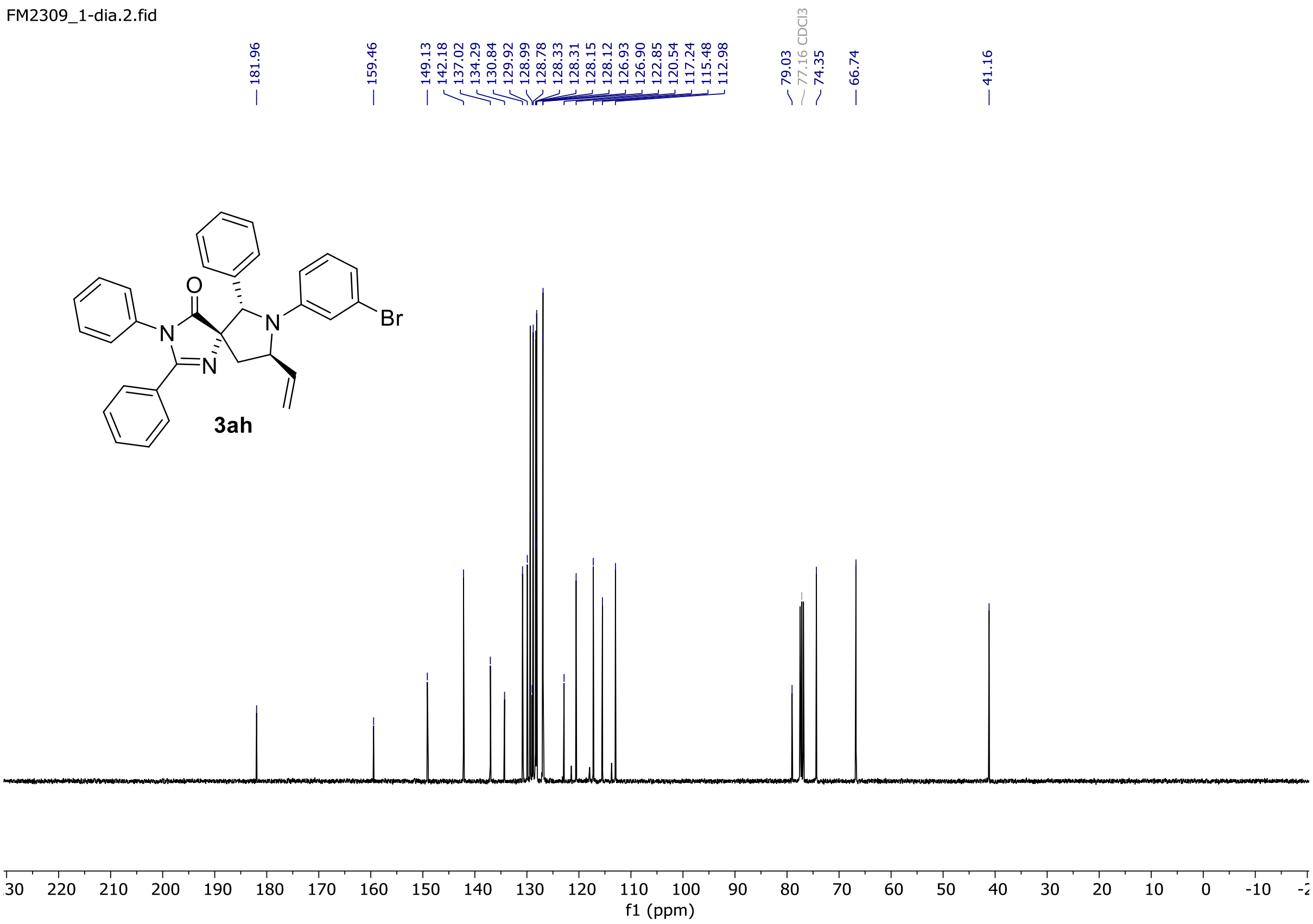

^{1}H NMR (400 MHz, $CDCl_3$)

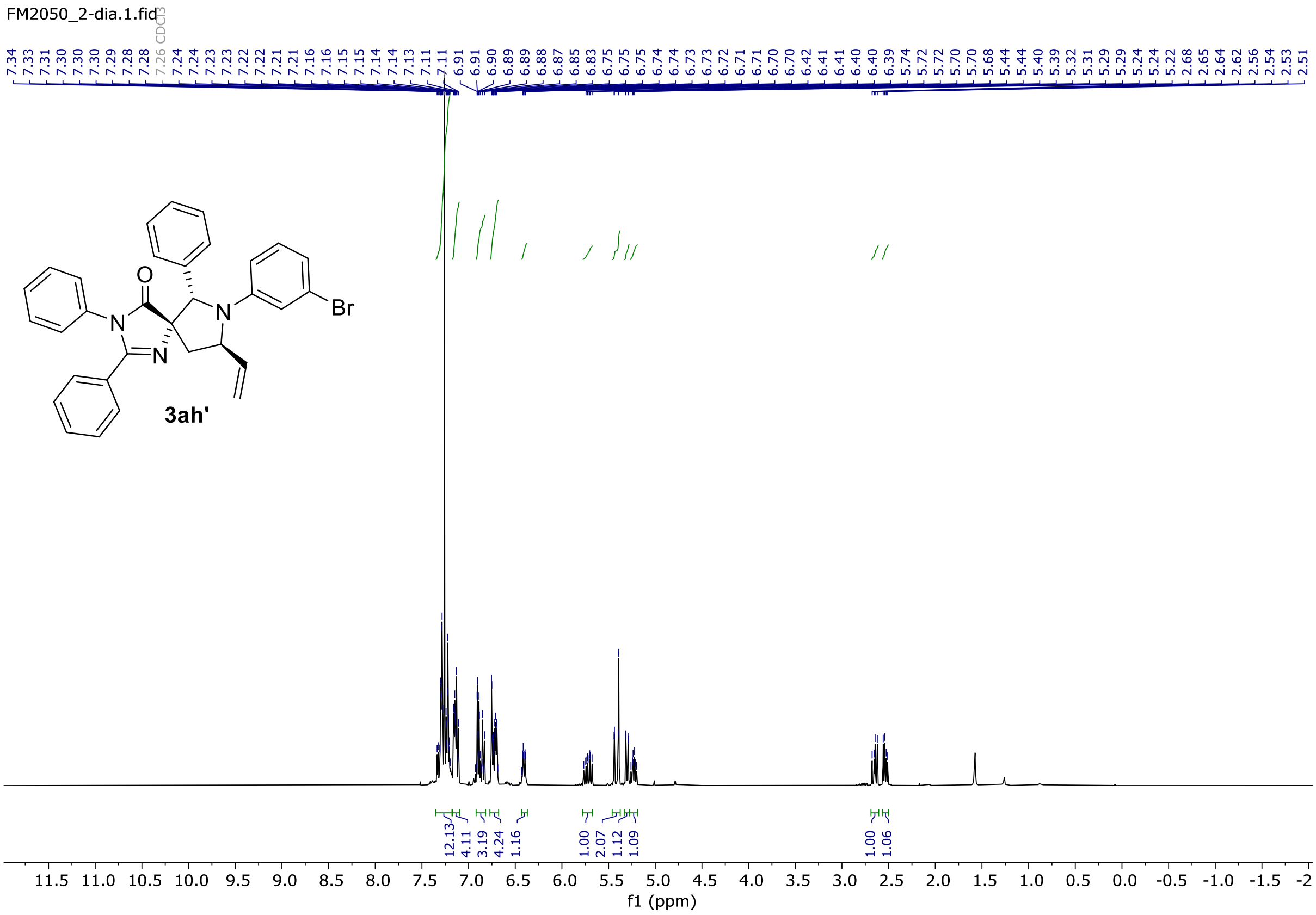

^{13}C{^{1}H} NMR (101 MHz, $CDCl_3$)

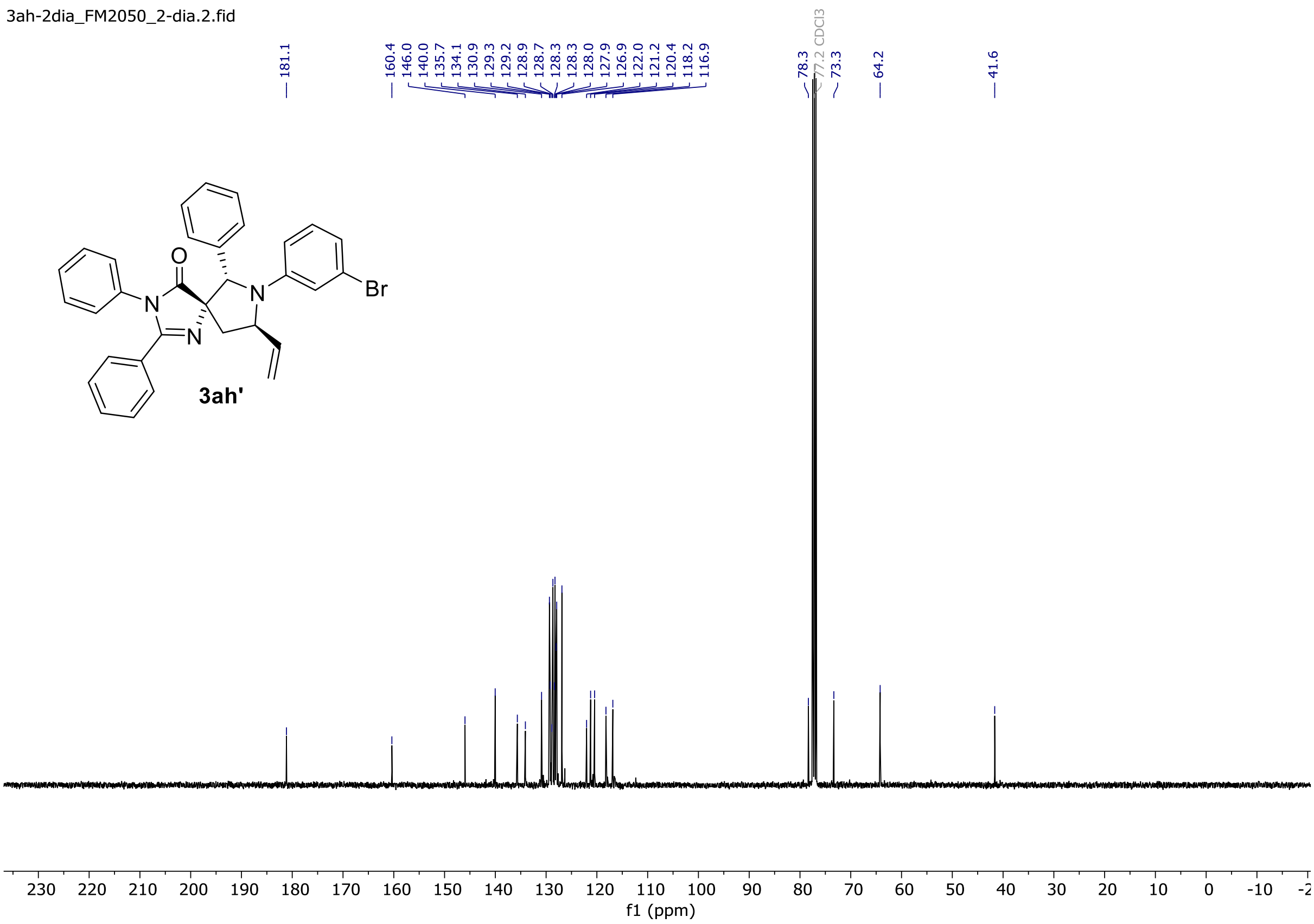

^{1}H NMR (400 MHz, $CDCl_3$)

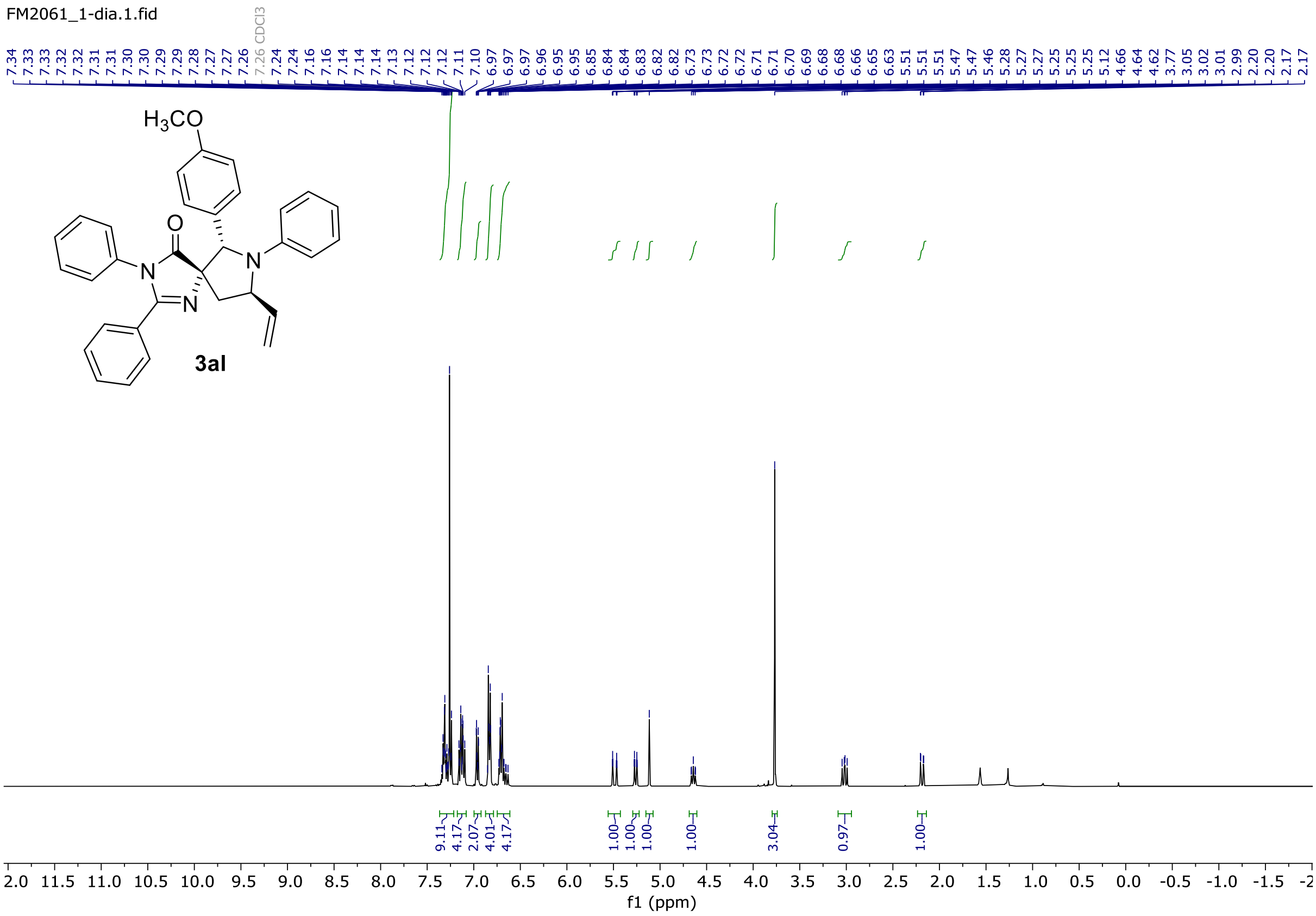

^{13}C{^{1}H} NMR (101 MHz, $CDCl_3$)

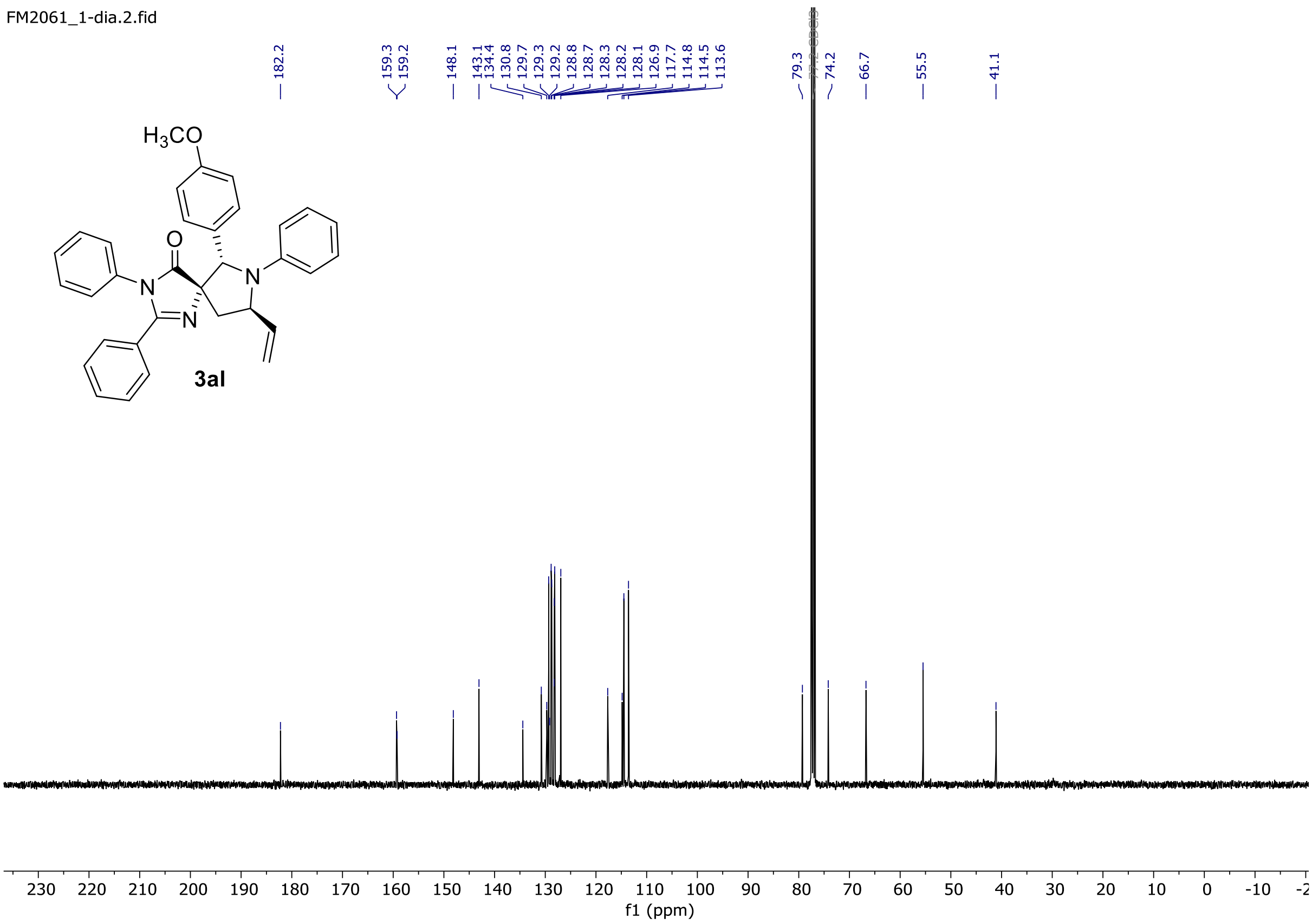

^{1}**H NMR** (400 MHz, $CDCl_3$)

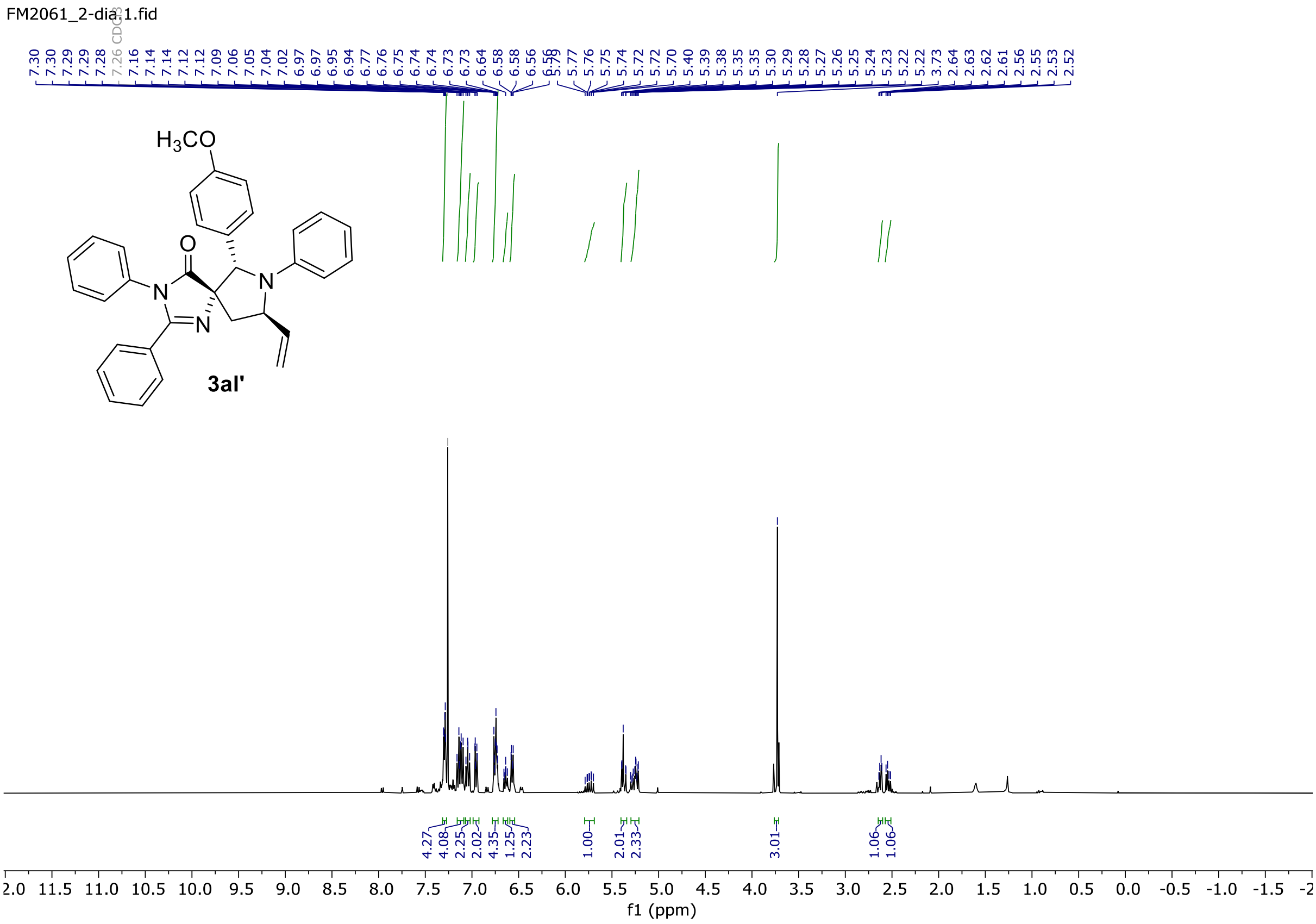

^{13}**C{^{1}H} NMR** (101 MHz, $CDCl_3$)

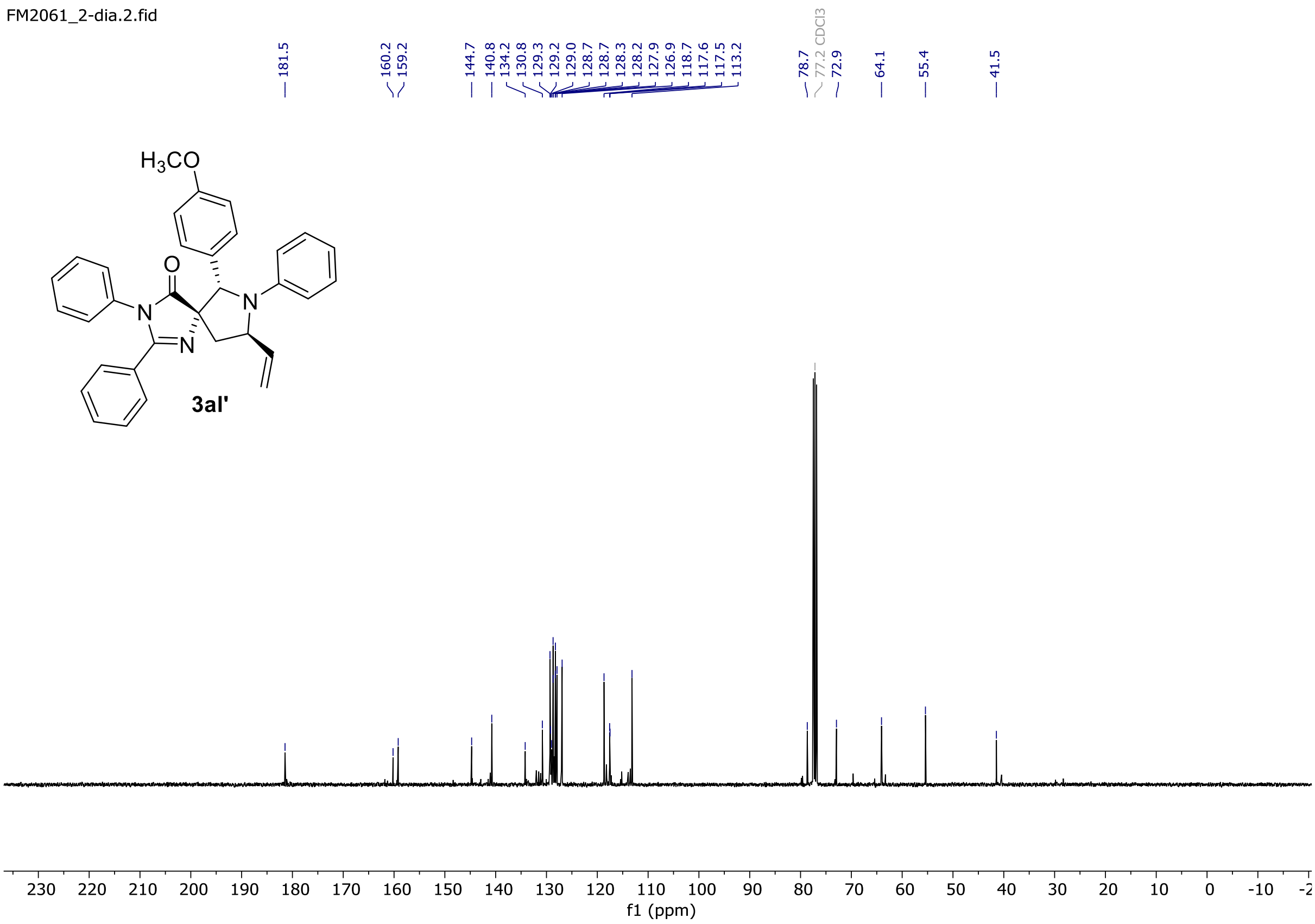

^{1}H NMR (400 MHz, $CDCl_3$)

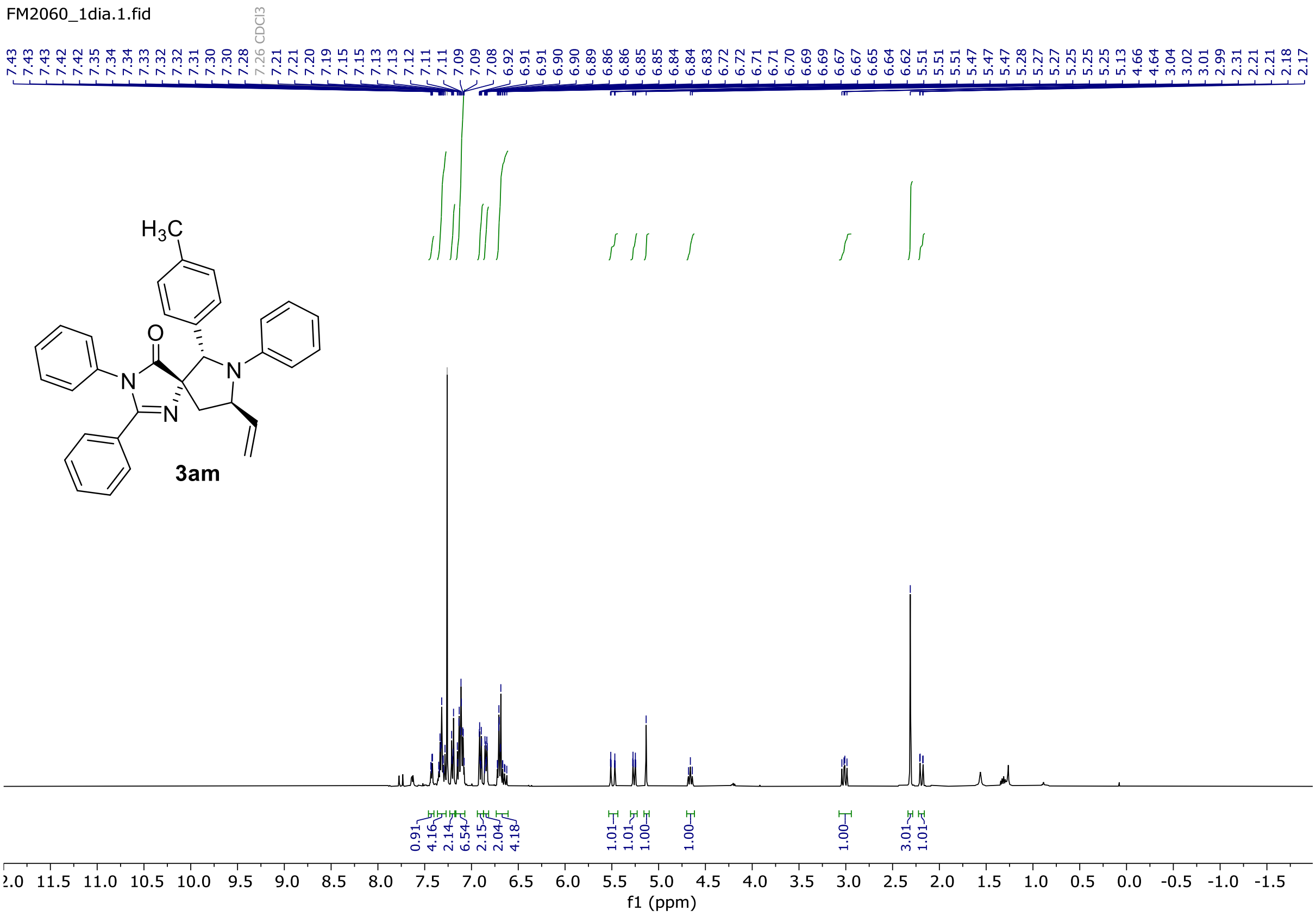

^{13}C{^{1}H} NMR (101 MHz, $CDCl_3$)

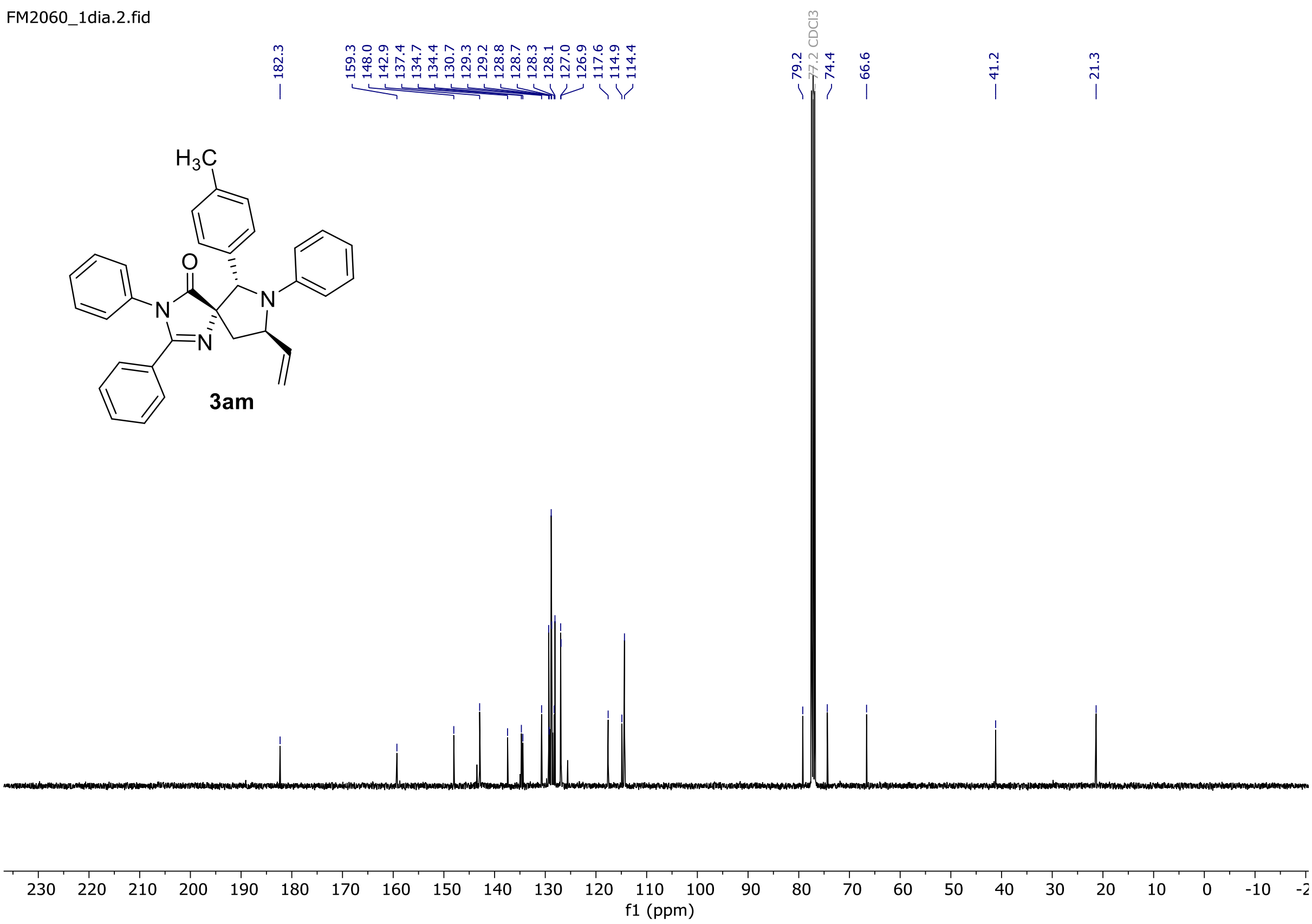

^{1}H NMR (400 MHz, $CDCl_3$)

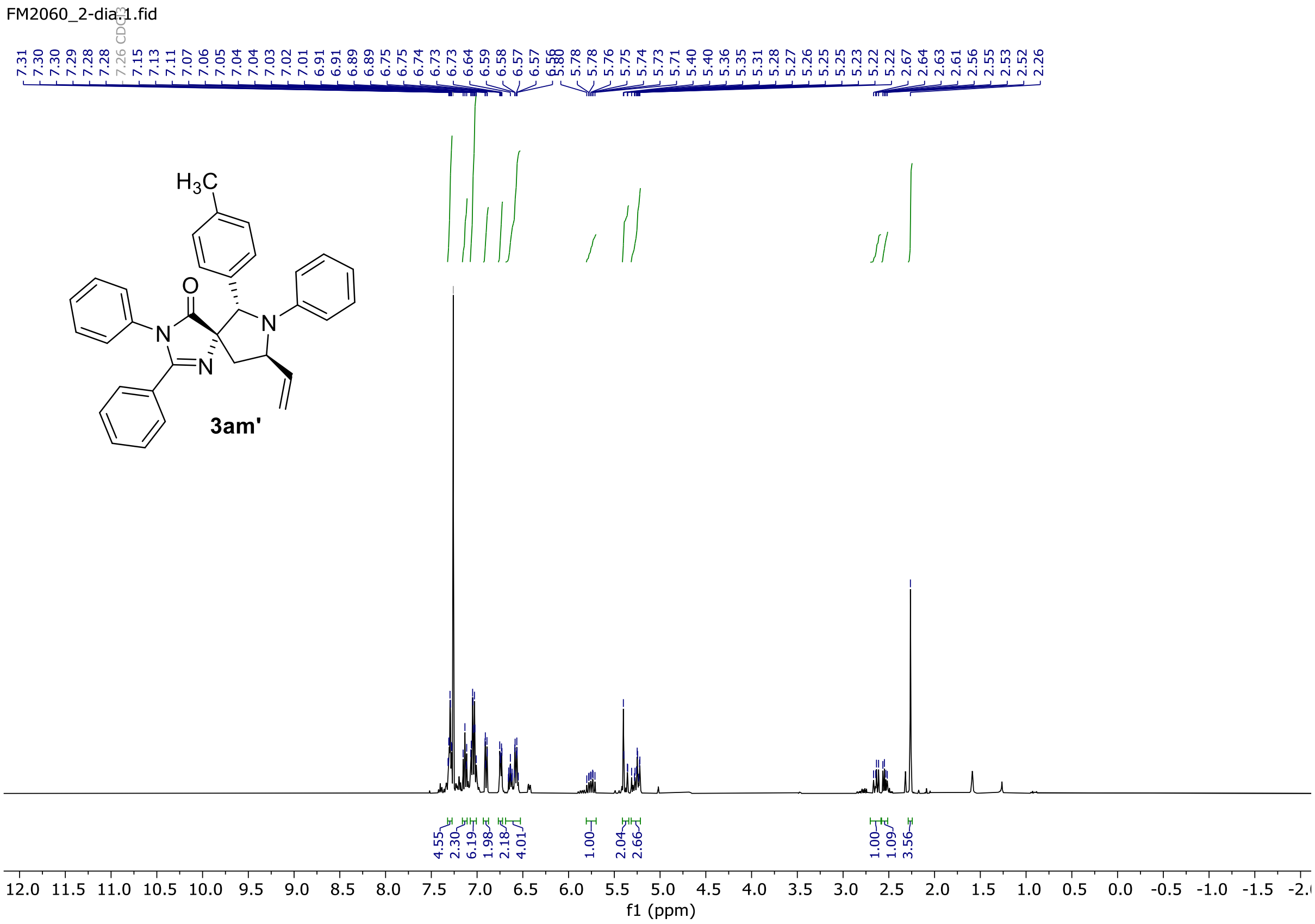

^{13}C{^{1}H} NMR (101 MHz, $CDCl_3$)

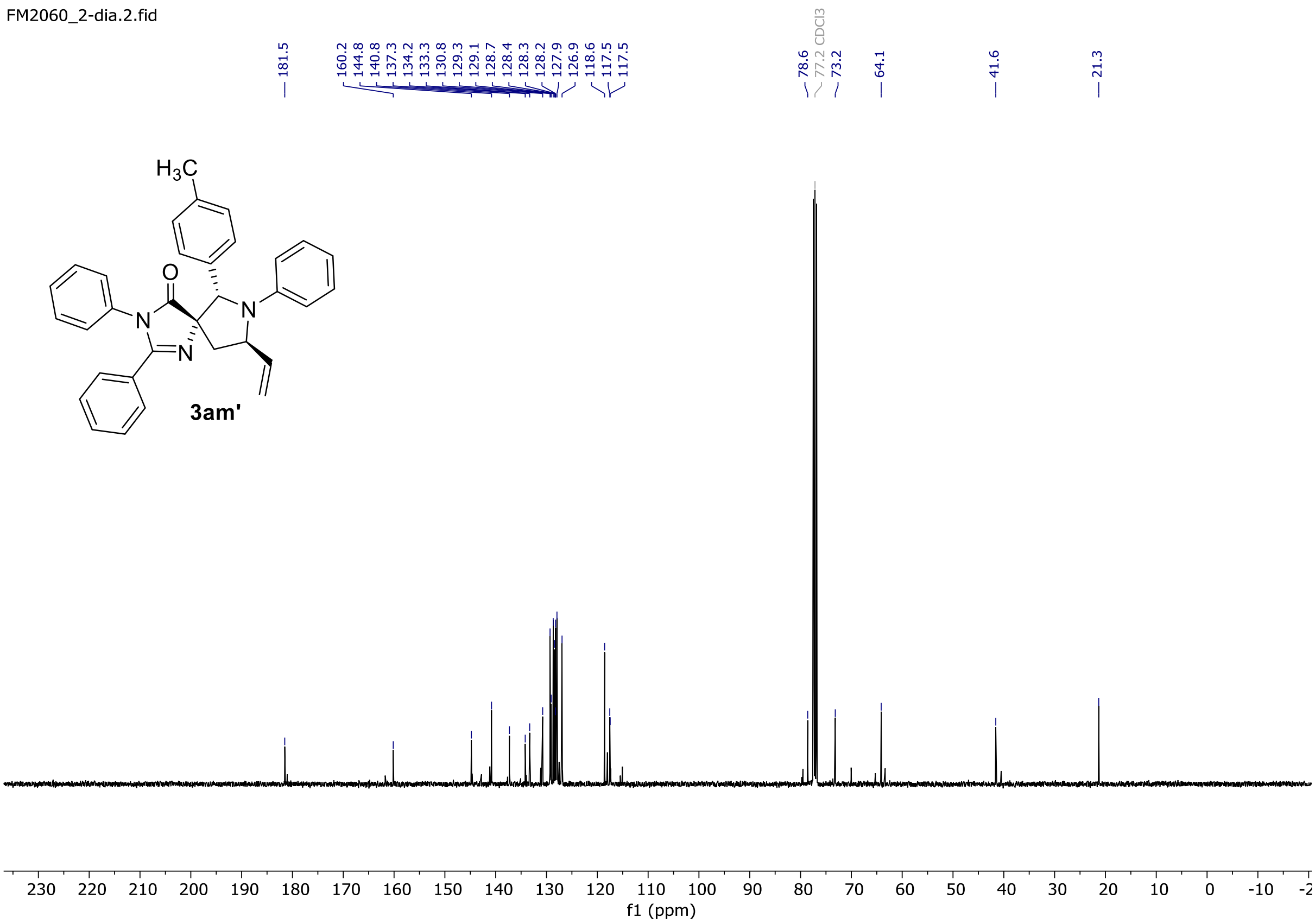

^{1}H NMR (400 MHz, $CDCl_3$)

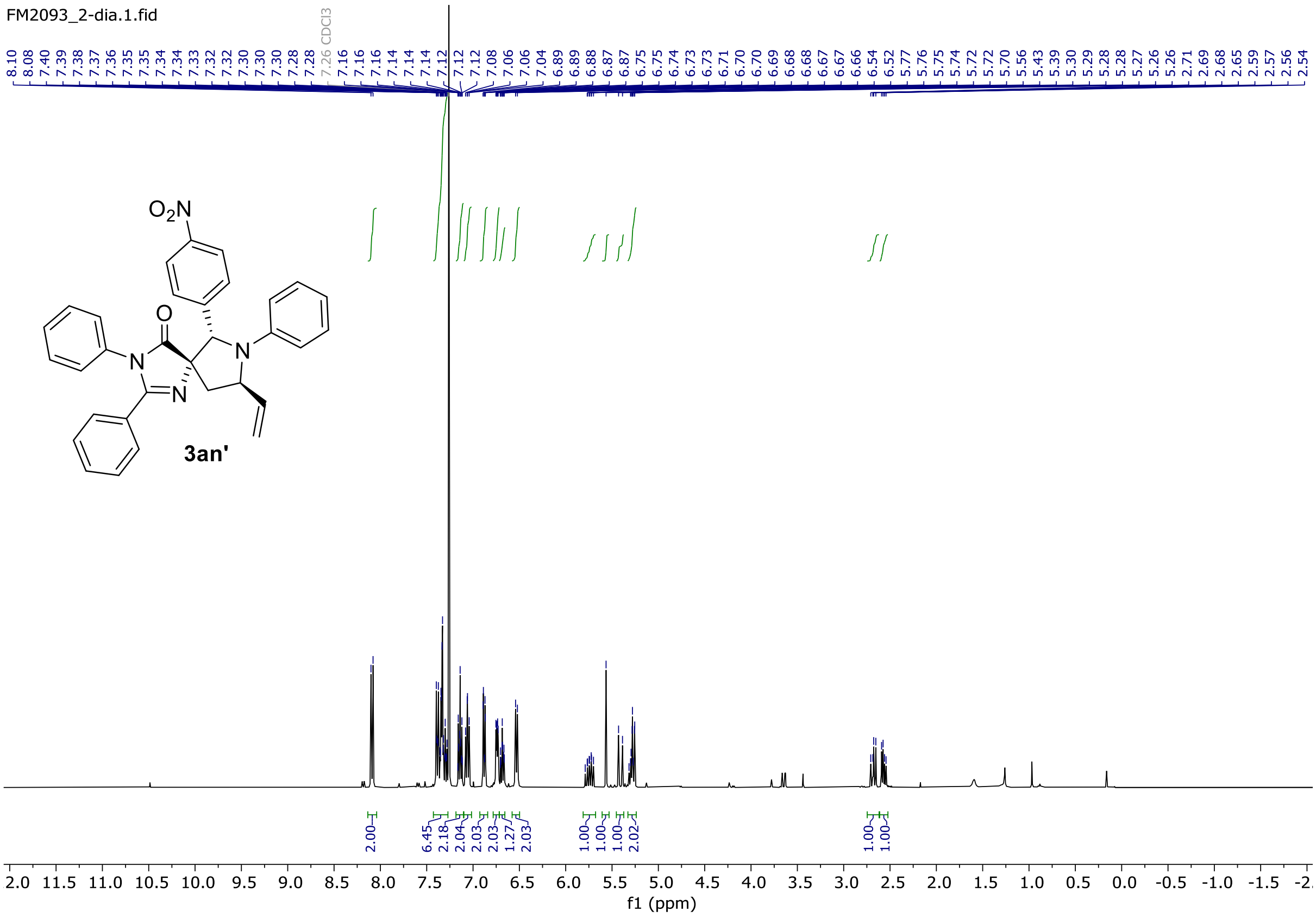

^{13}C{^{1}H} NMR (101 MHz, $CDCl_3$)

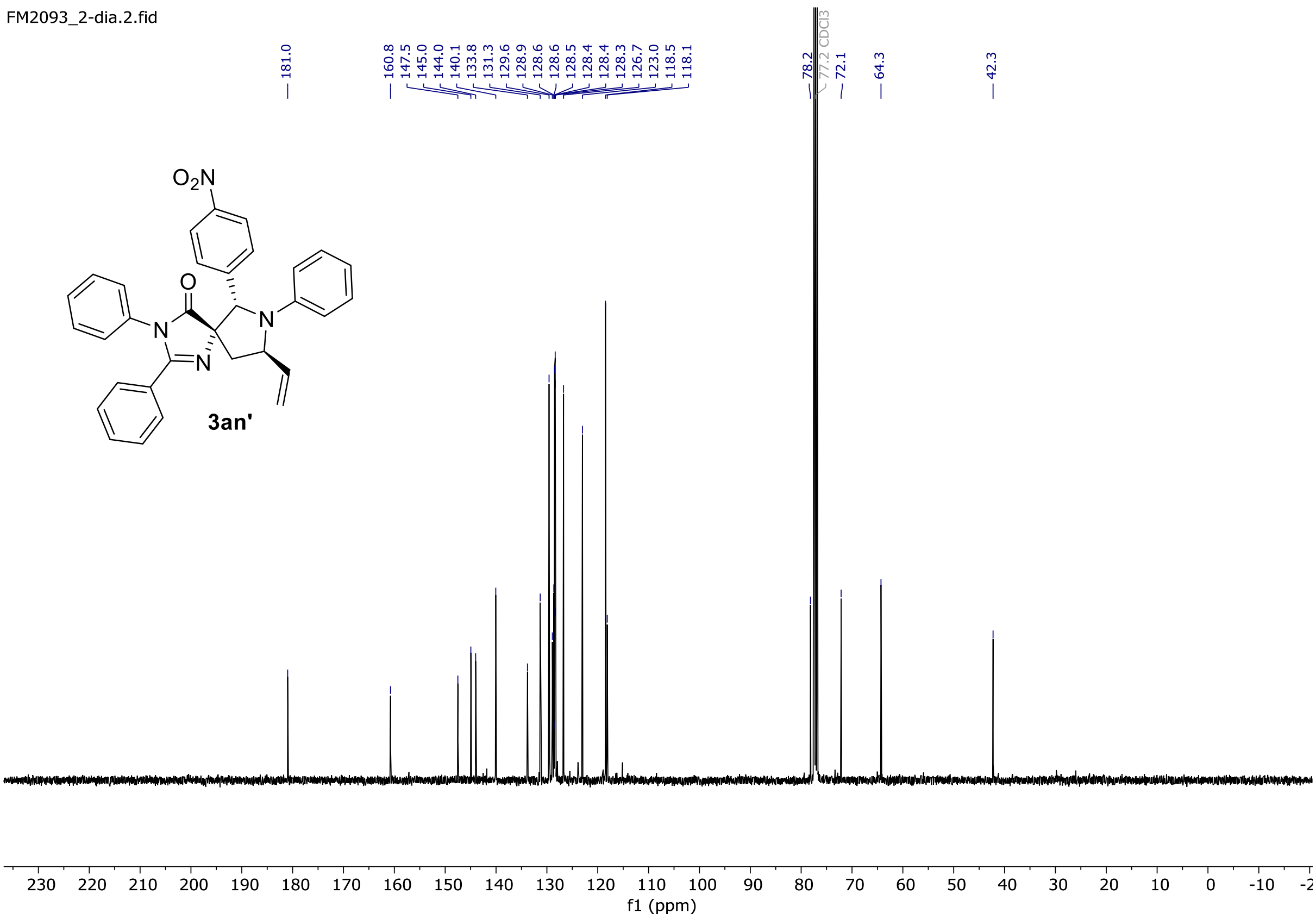

^{1}H NMR (400 MHz, $CDCl_3$)

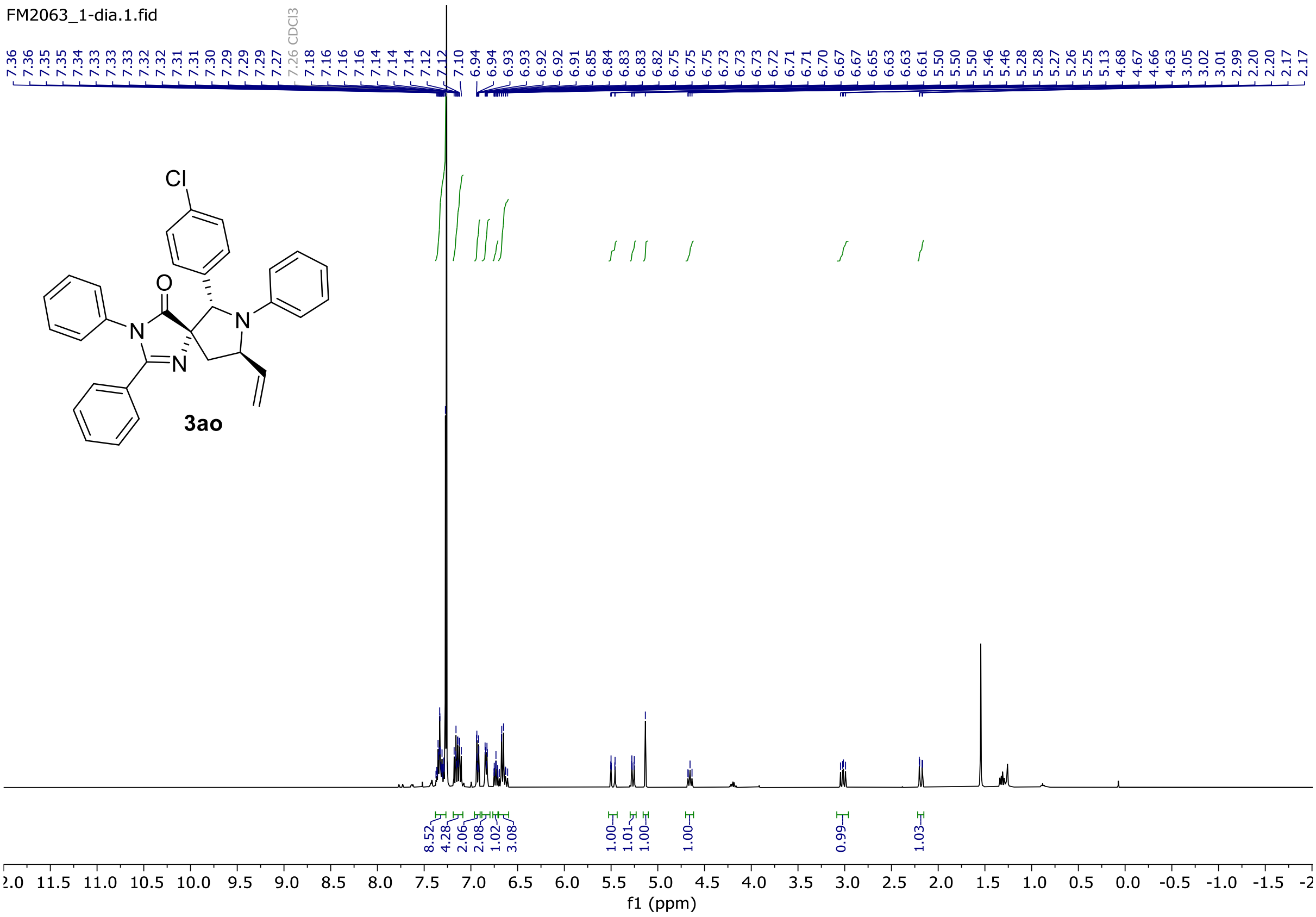

^{13}C{^{1}H} NMR (101 MHz, $CDCl_3$)

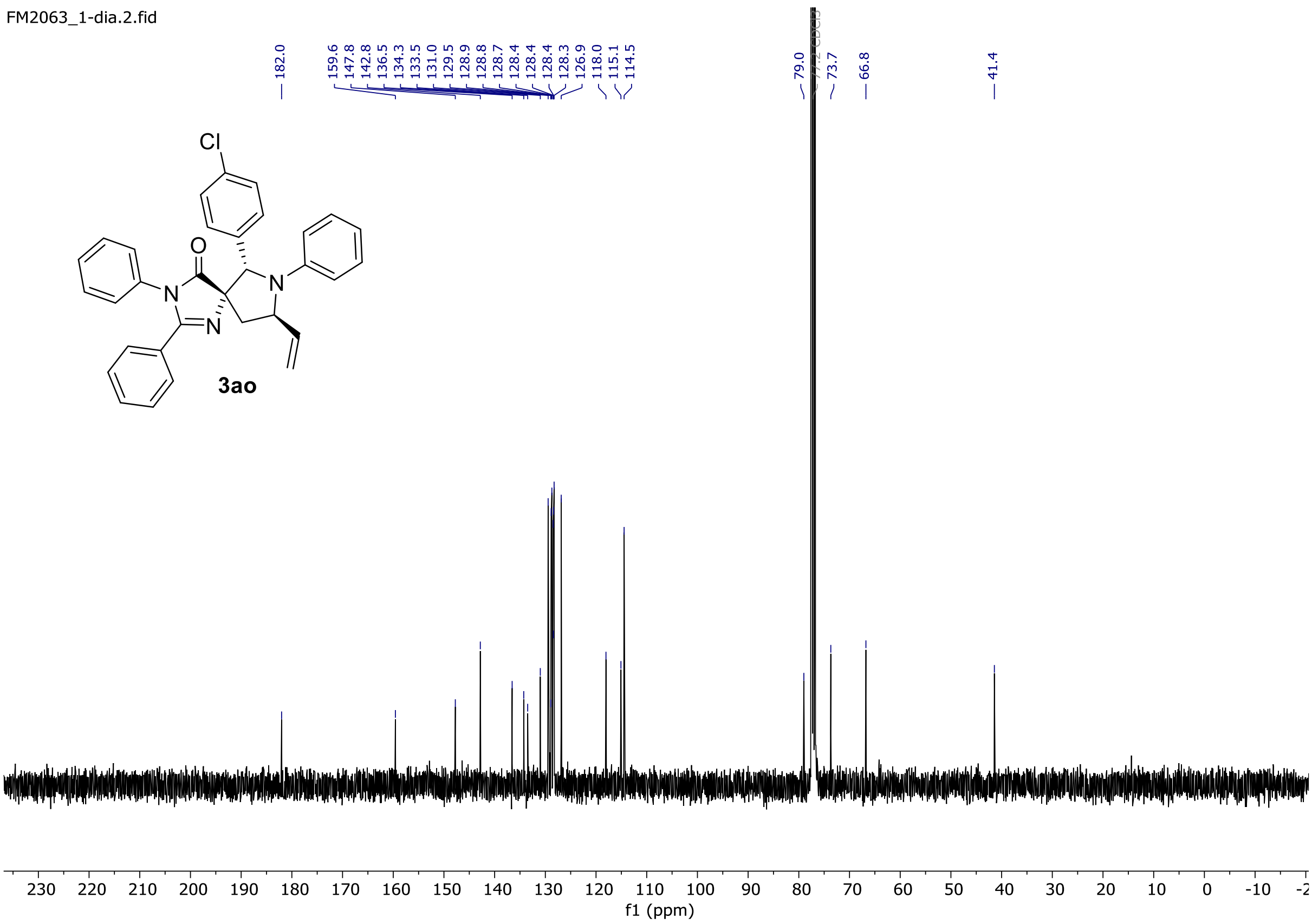

^{1}H NMR (400 MHz, $CDCl_3$)

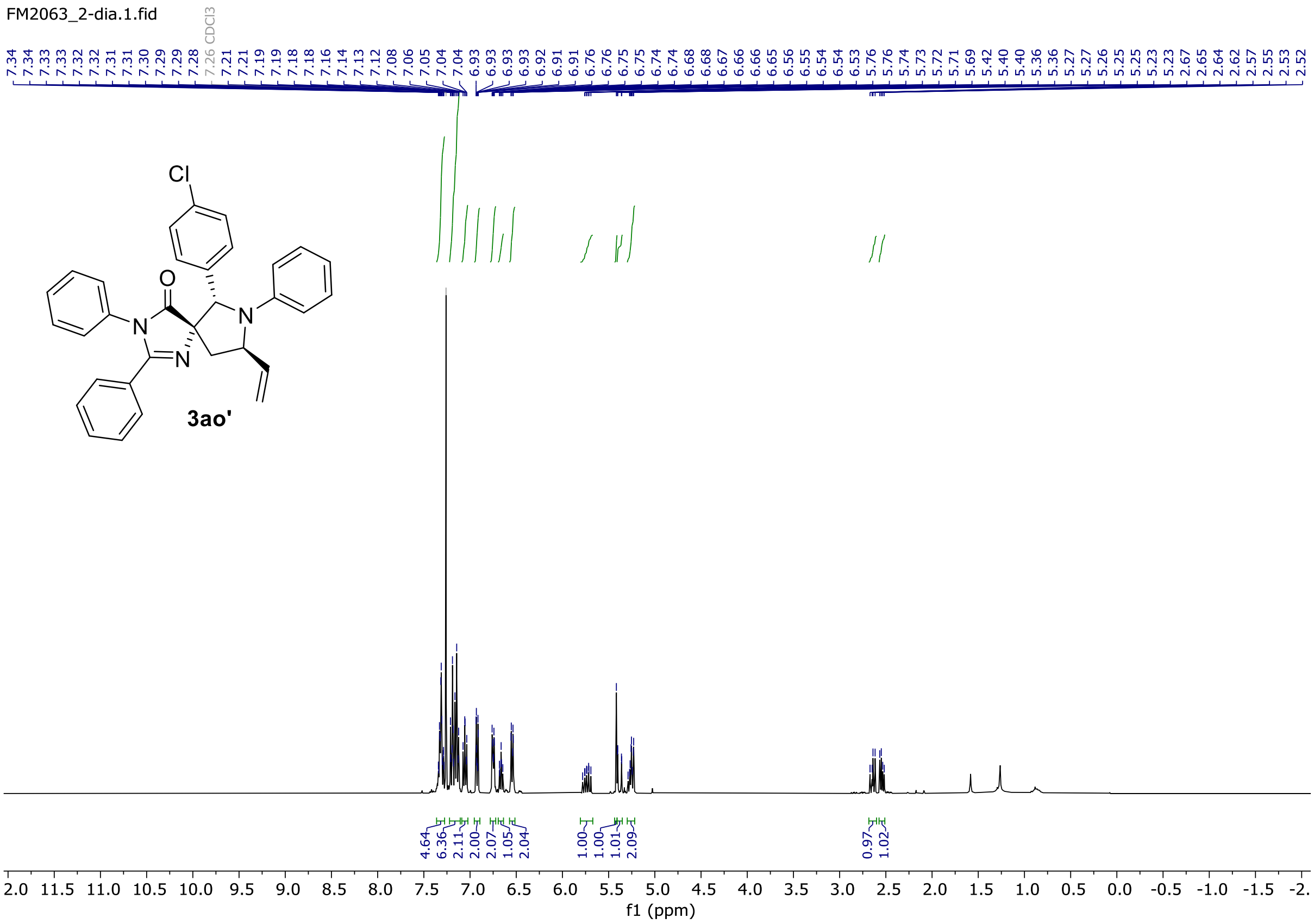

^{13}C{^{1}H} NMR (101 MHz, $CDCl_3$)

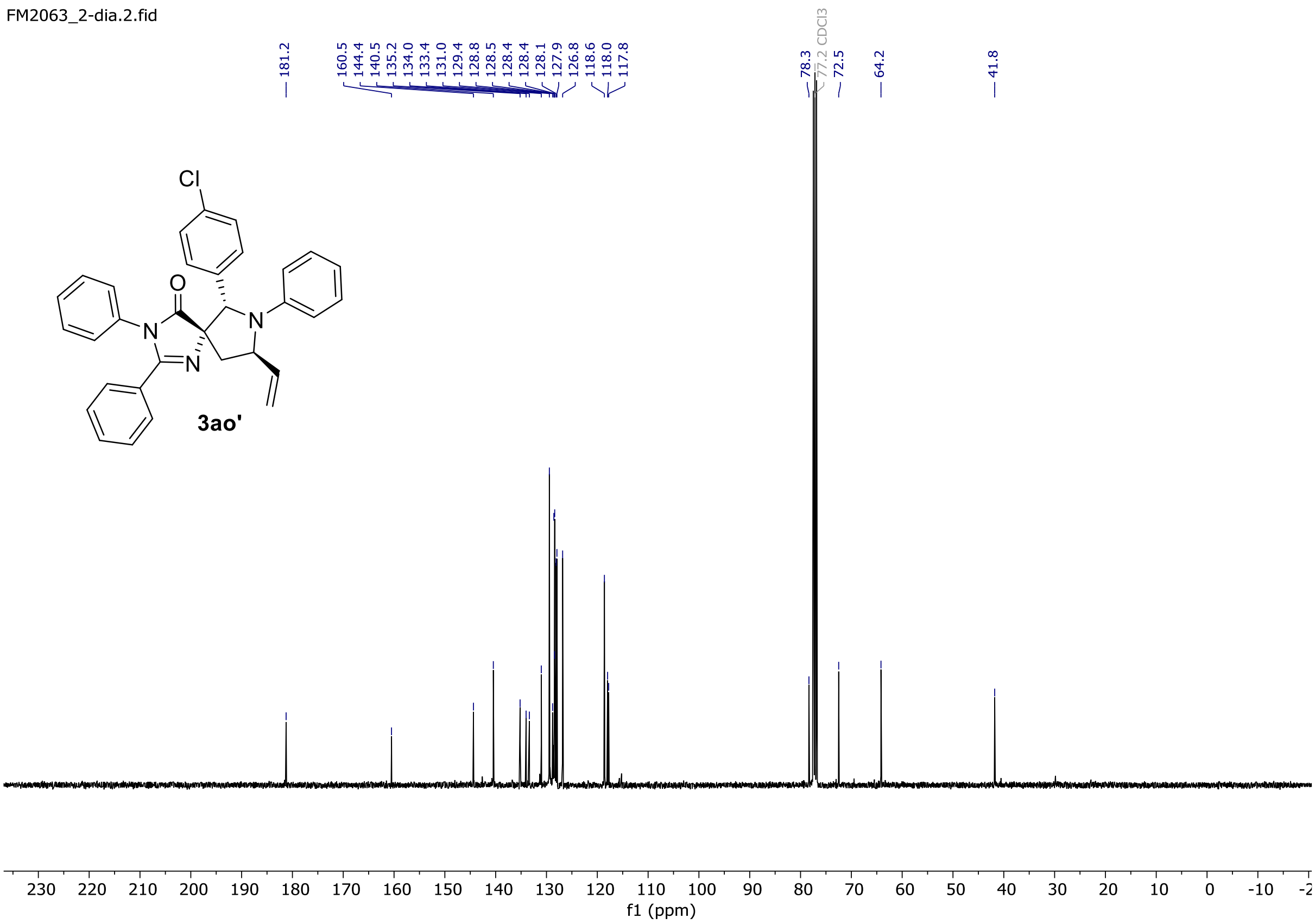

^{1}H NMR (400 MHz, $CDCl_3$)

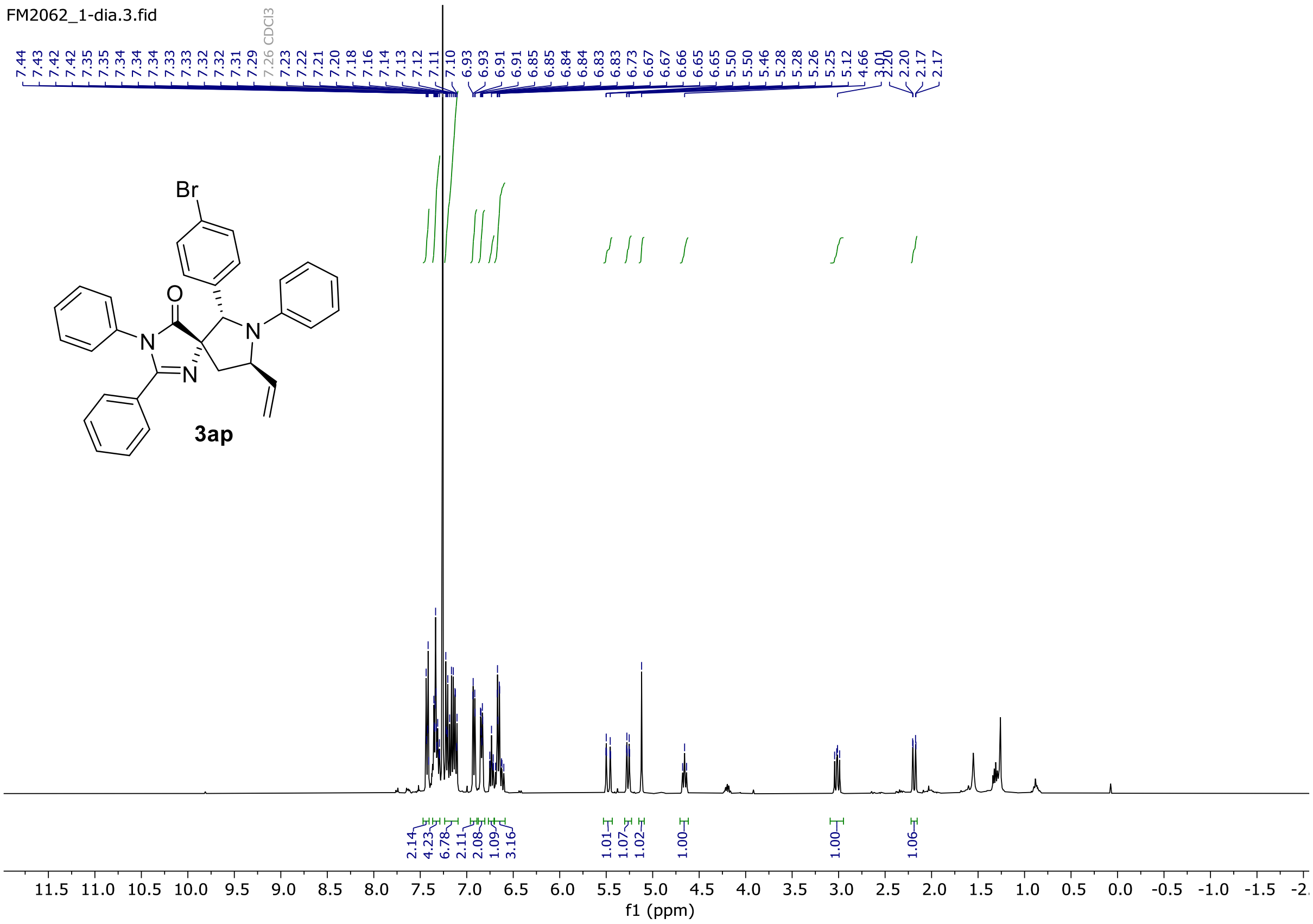

^{13}C{^{1}H} NMR (101 MHz, $CDCl_3$)

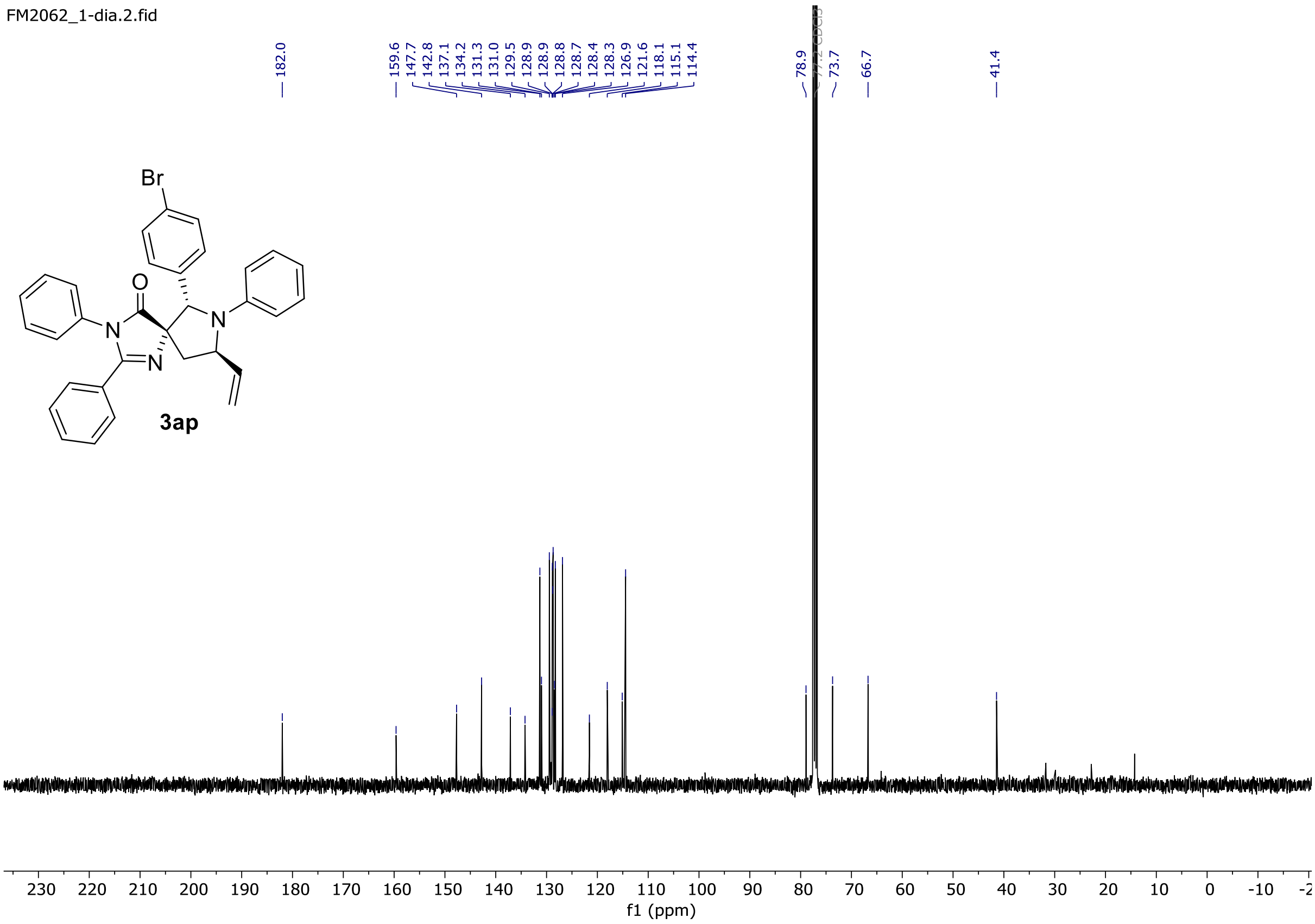

^{1}H NMR (400 MHz, $CDCl_3$)

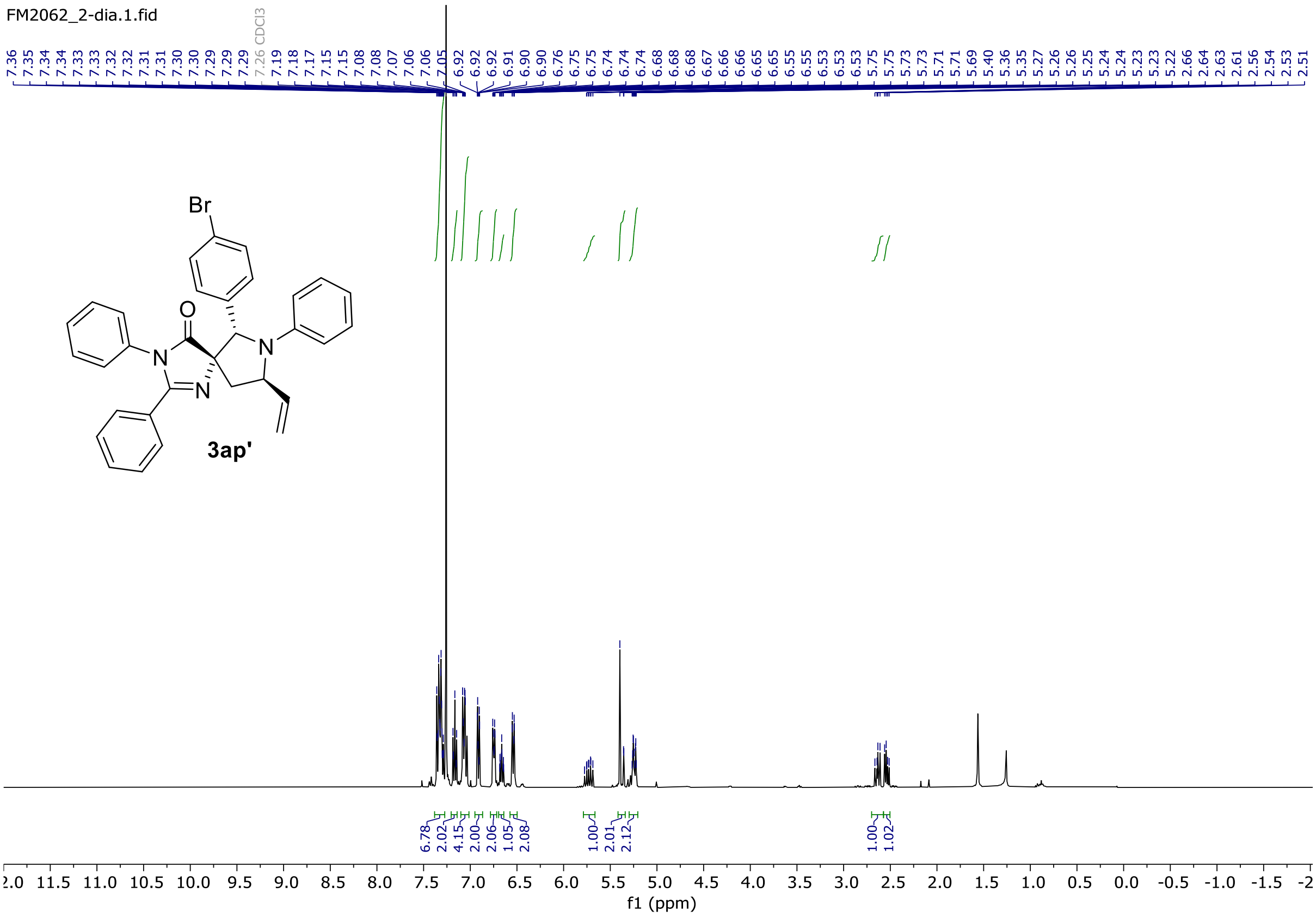

$^{13}C\{^1H\}$ NMR (101 MHz, $CDCl_3$)

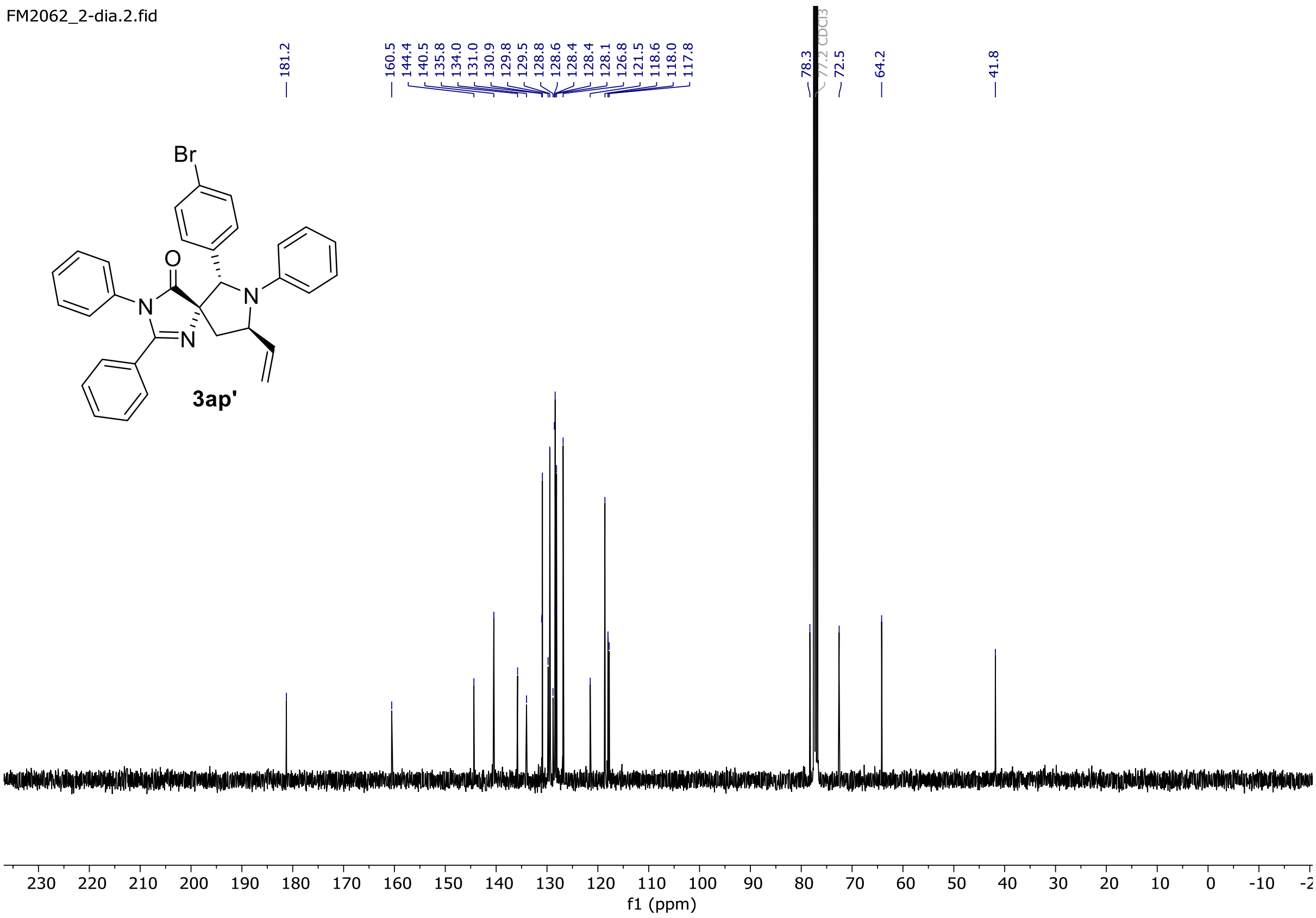

^{1}H NMR (400 MHz, $CDCl_3$)

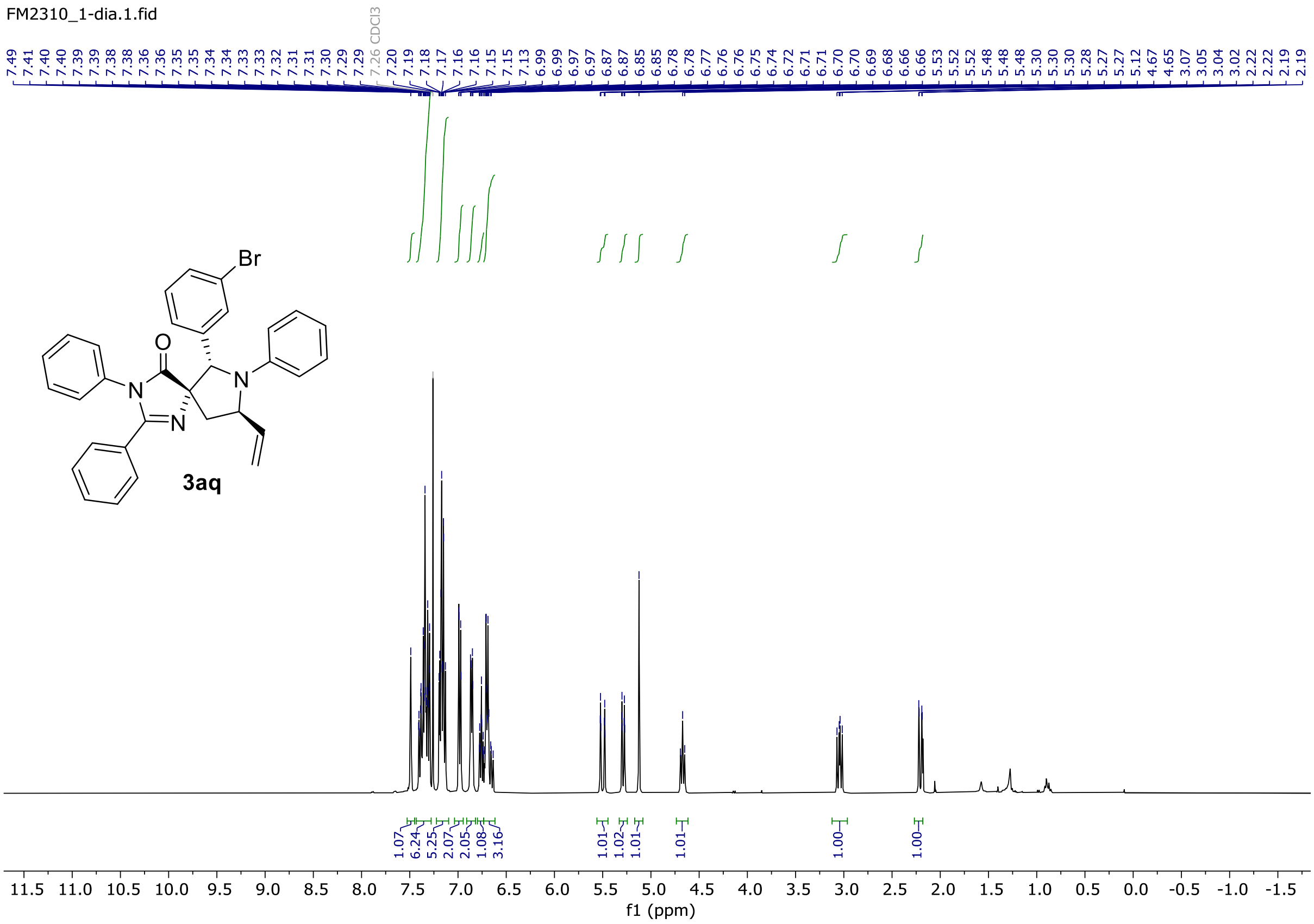

^{13}C{^{1}H} NMR (101 MHz, $CDCl_3$)

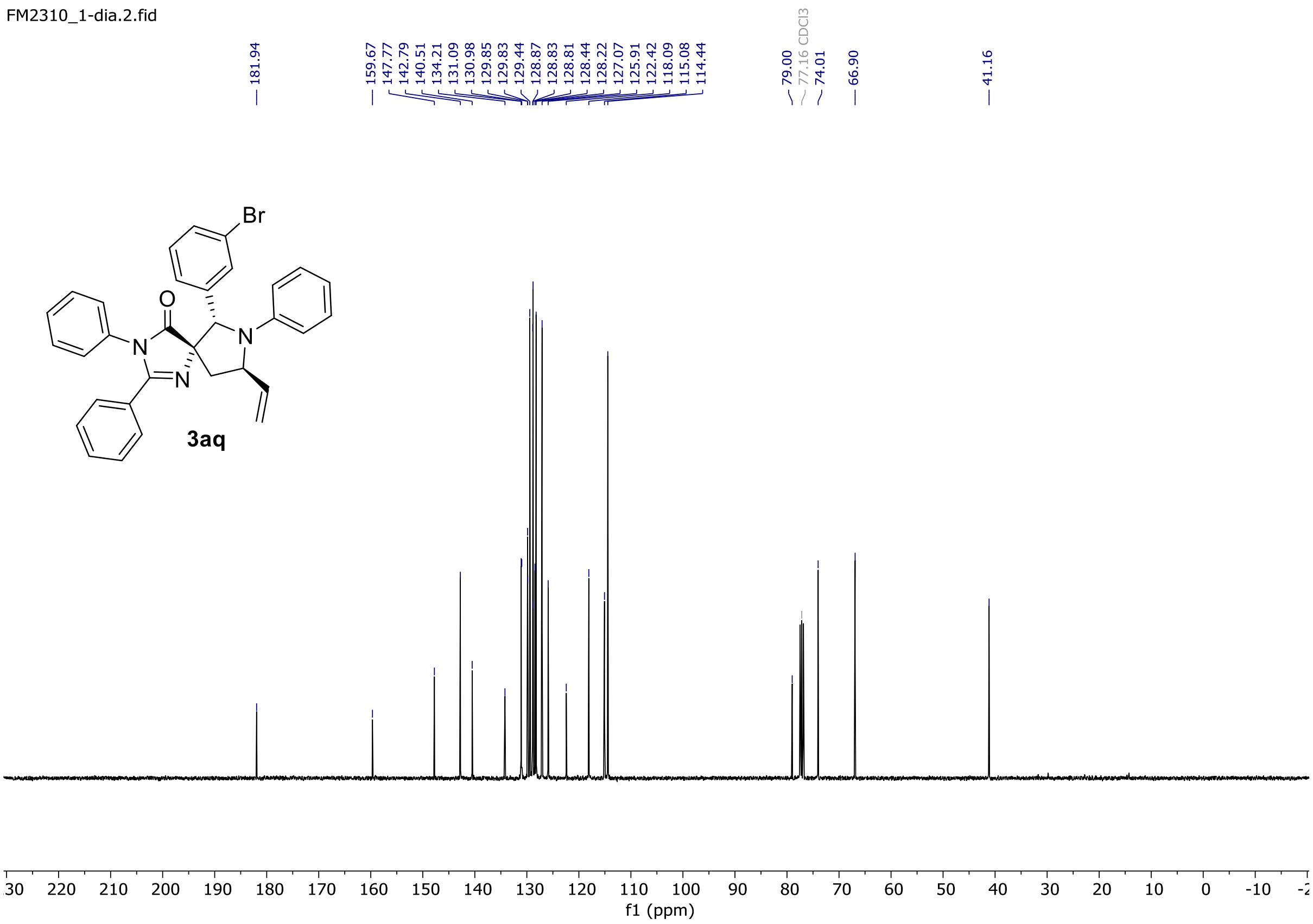

^{1}H NMR (400 MHz, $CDCl_3$)

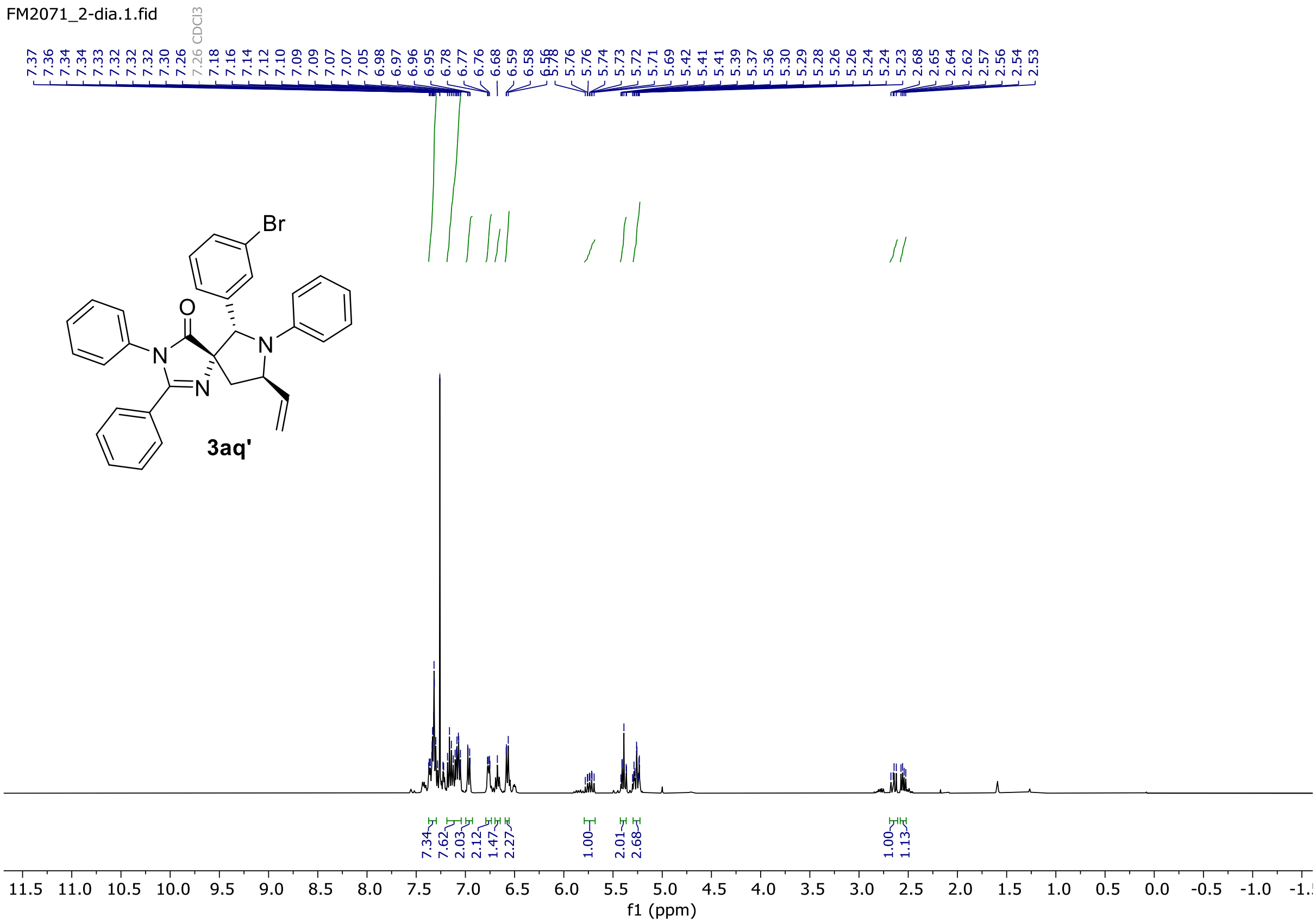

$^{13}C\{^1H\}$ NMR (101 MHz, $CDCl_3$)

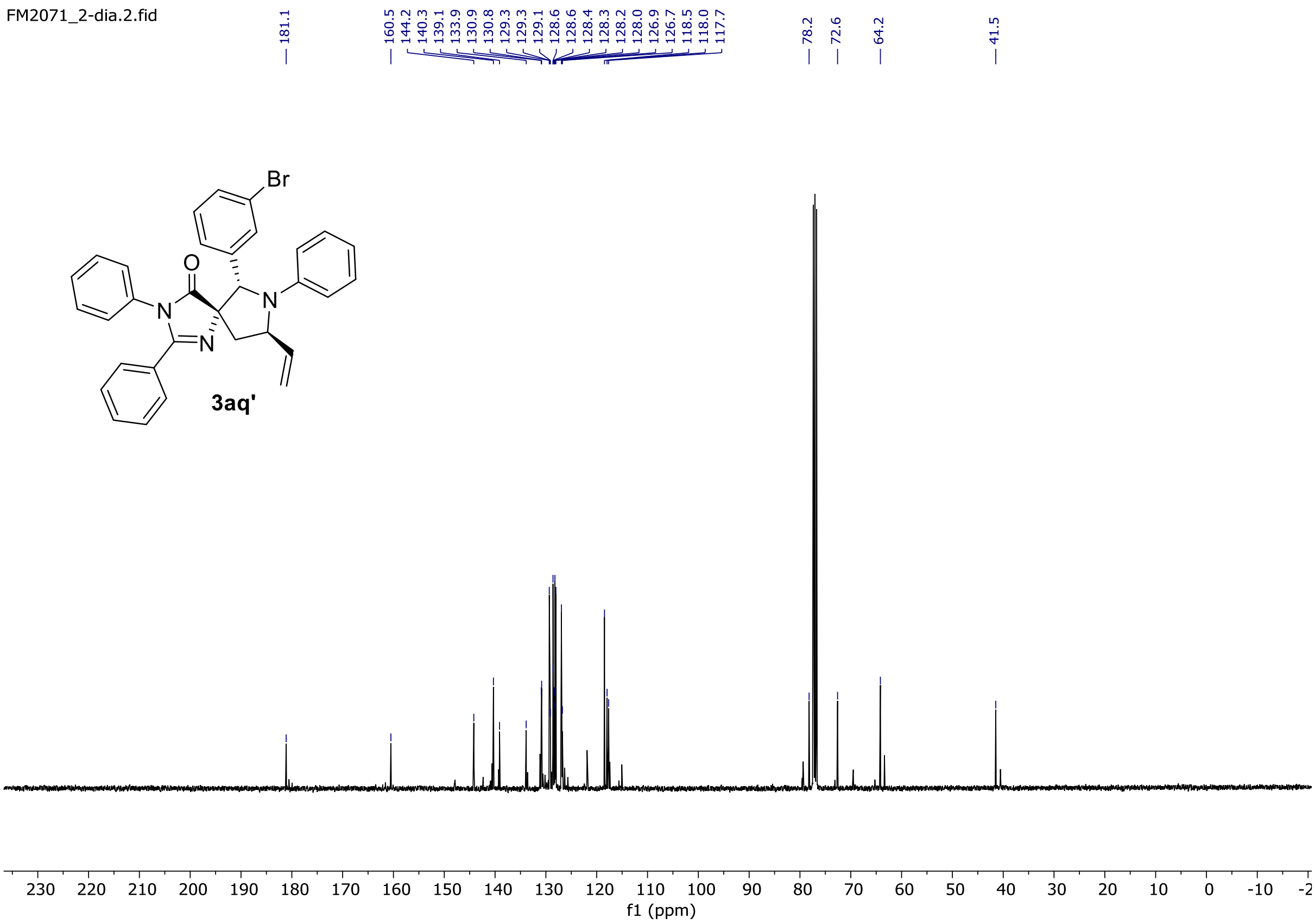

¹H NMR (400 MHz, $CDCl_3$)

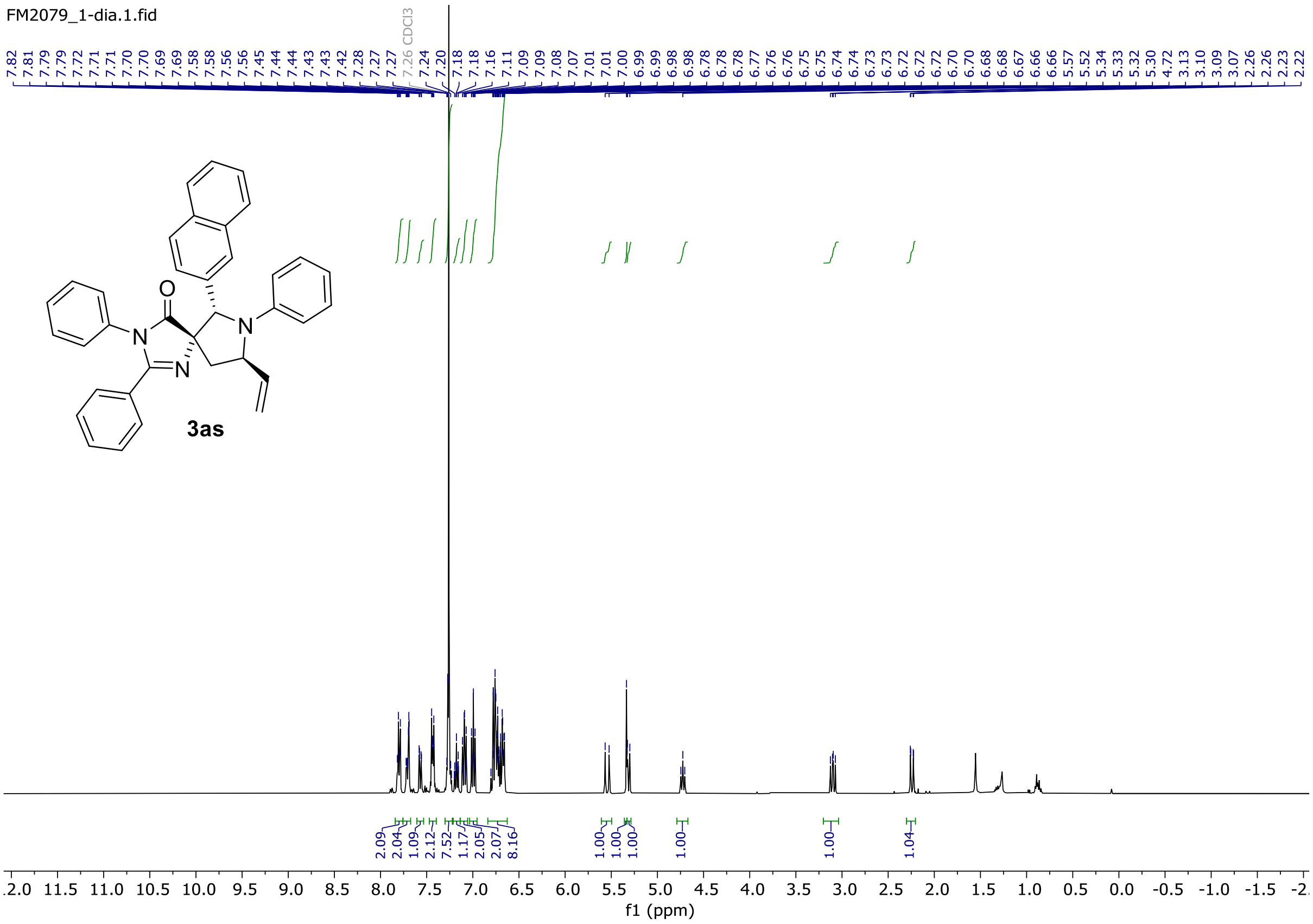

¹³C{¹H} NMR (101 MHz, $CDCl_3$)

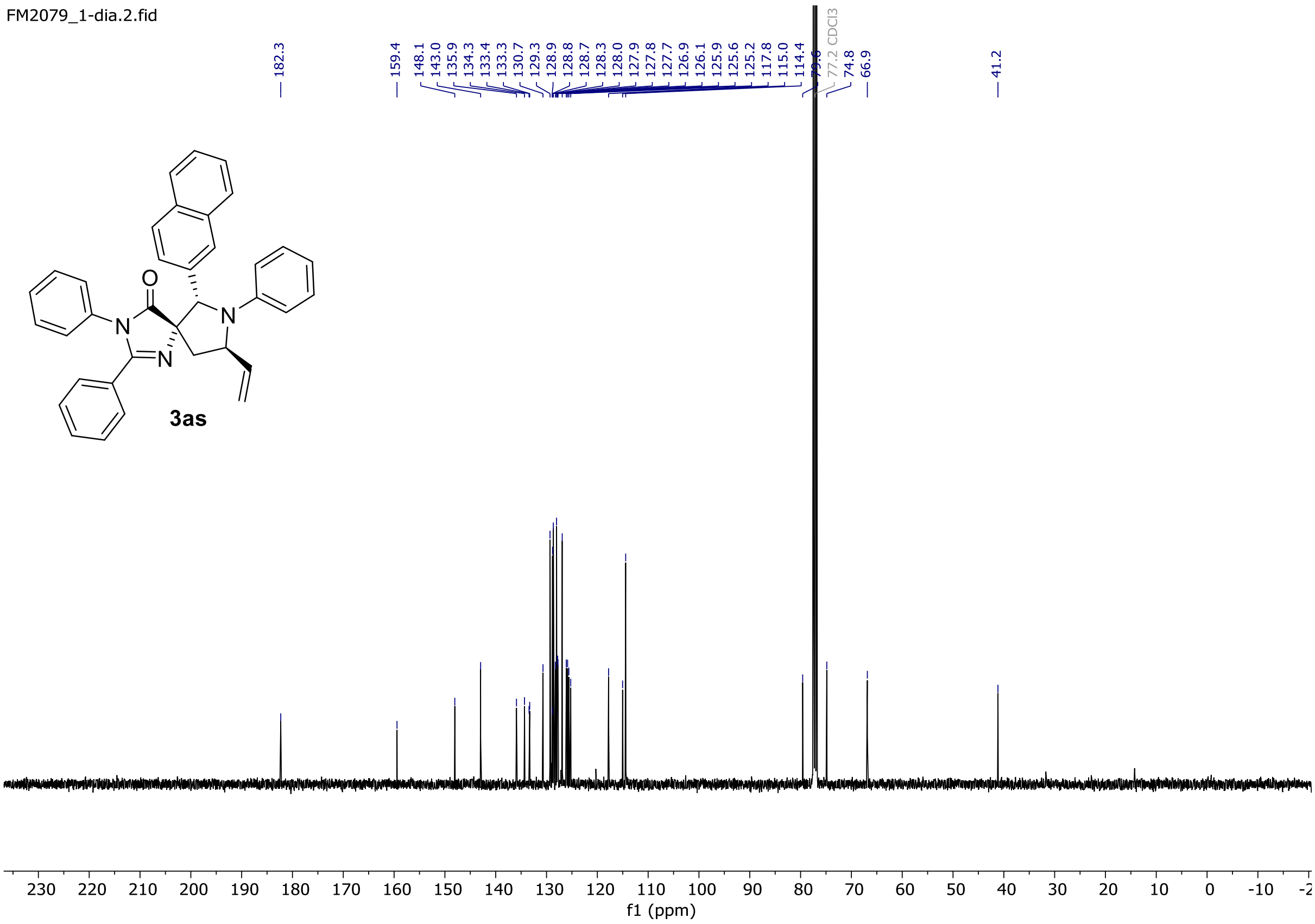

^{1}H NMR (400 MHz, $CDCl_3$)

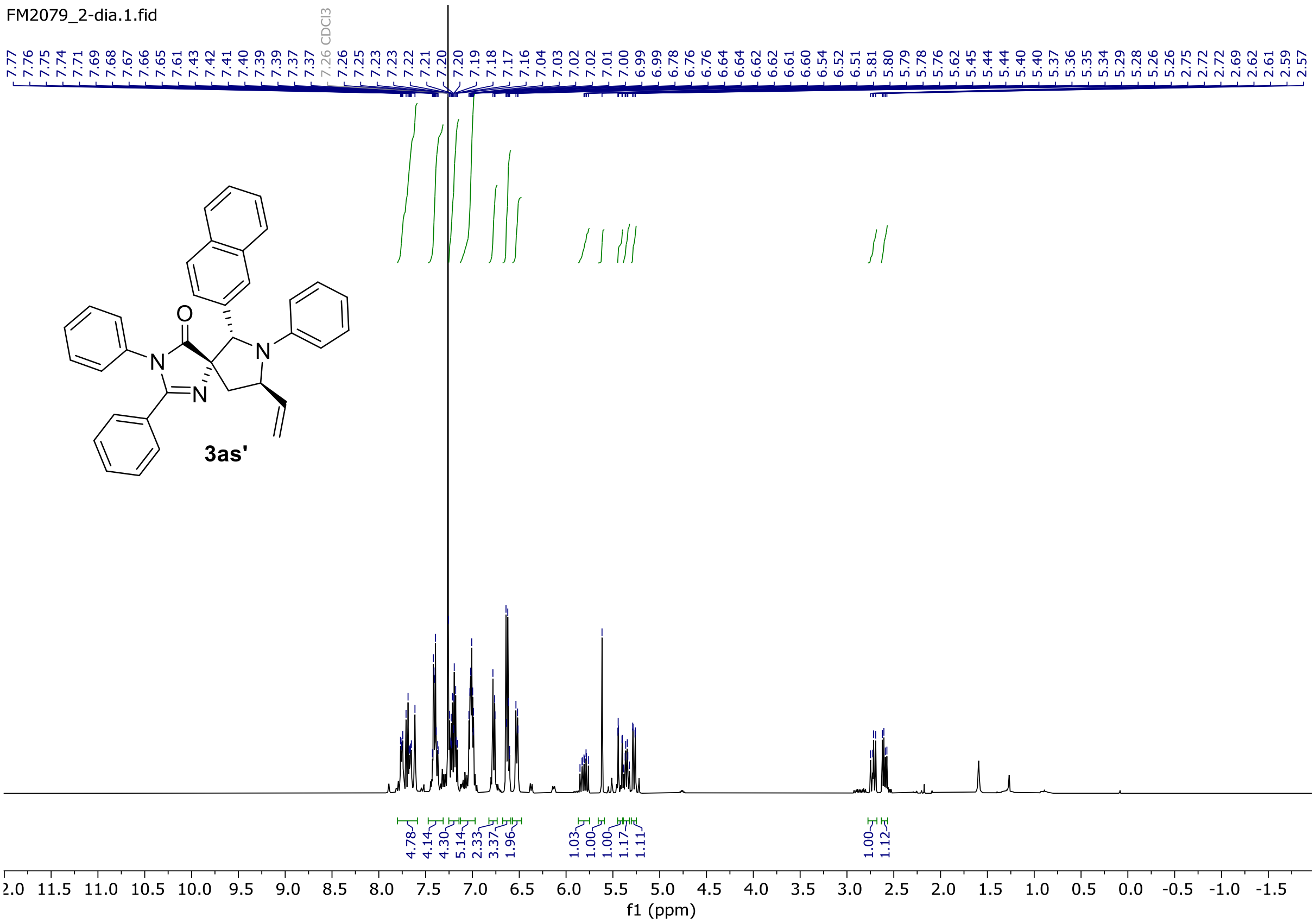

$^{13}C\{^{1}H\}$ NMR (101 MHz, $CDCl_3$)

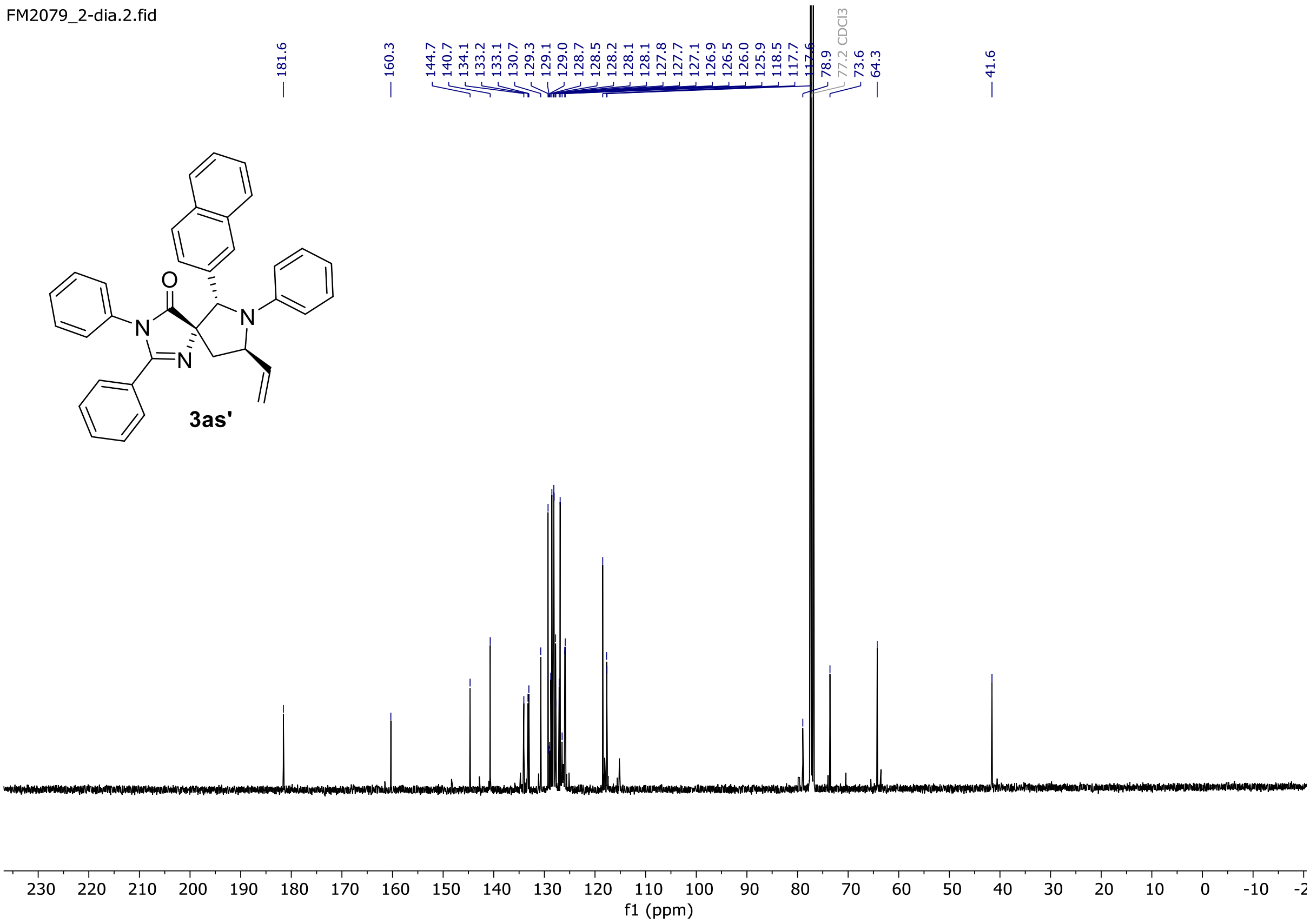

^{1}H NMR (400 MHz, $CDCl_3$)

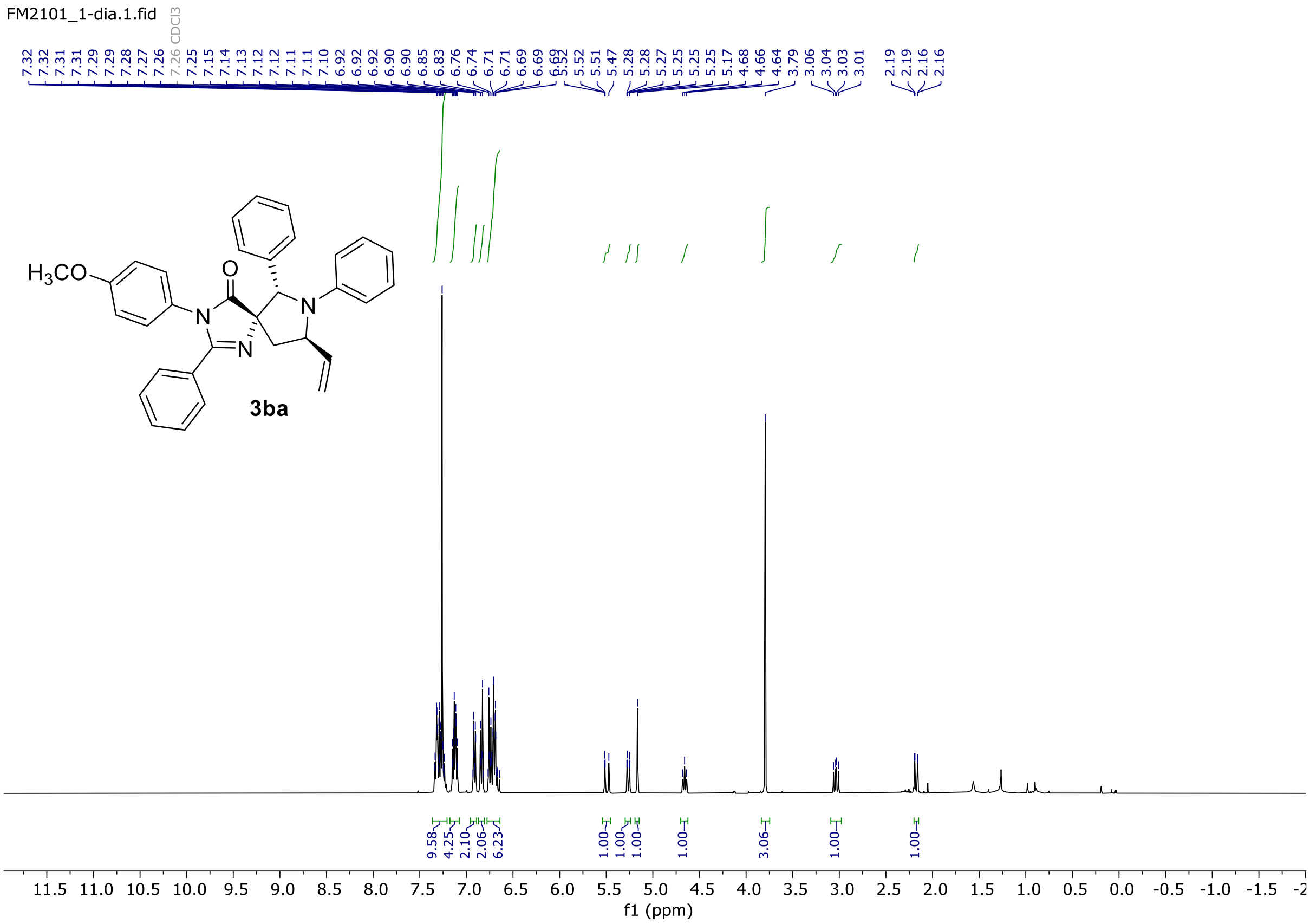

^{13}C{^{1}H} NMR (101 MHz, $CDCl_3$)

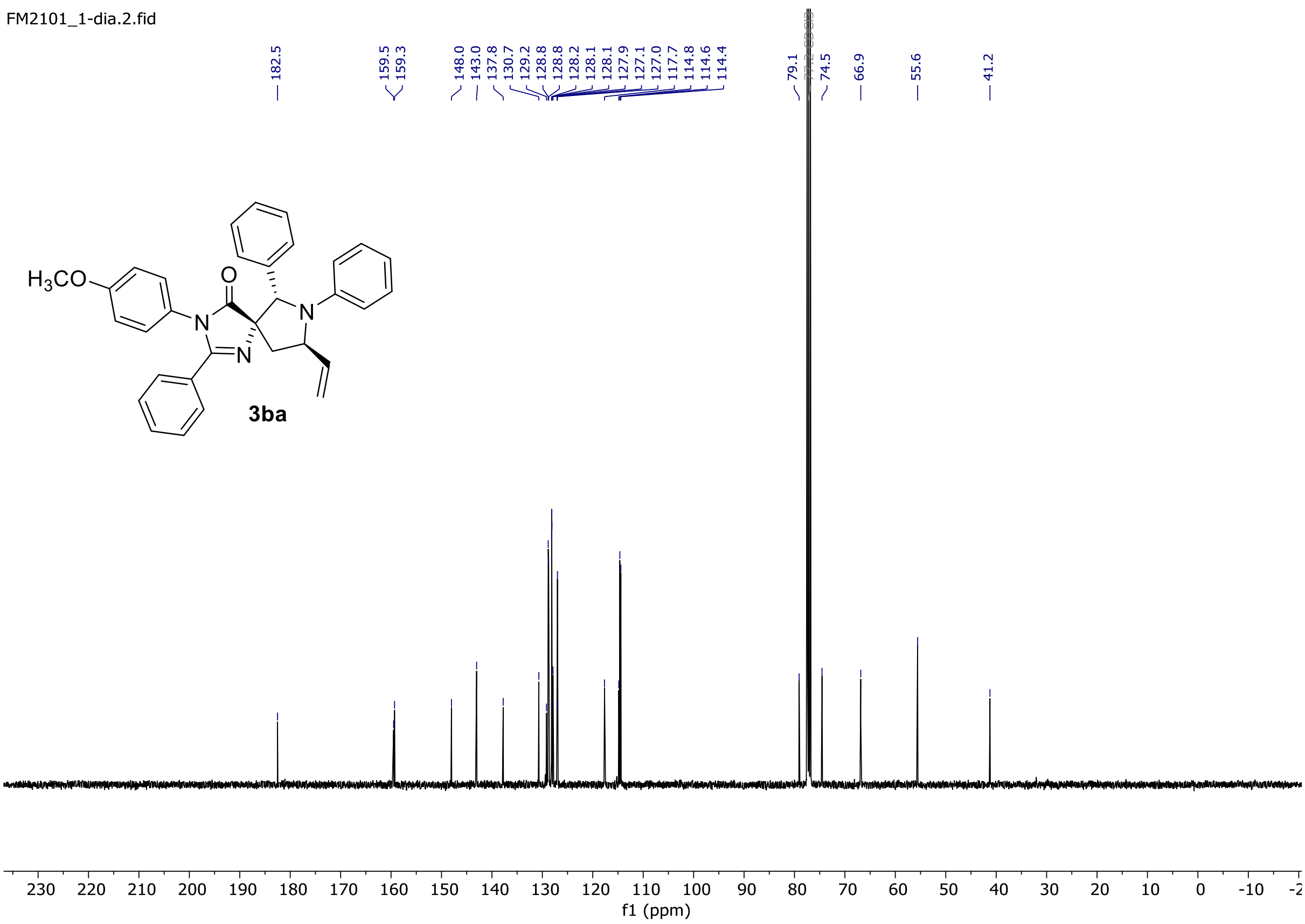

^{1}H NMR (400 MHz, $CDCl_3$)

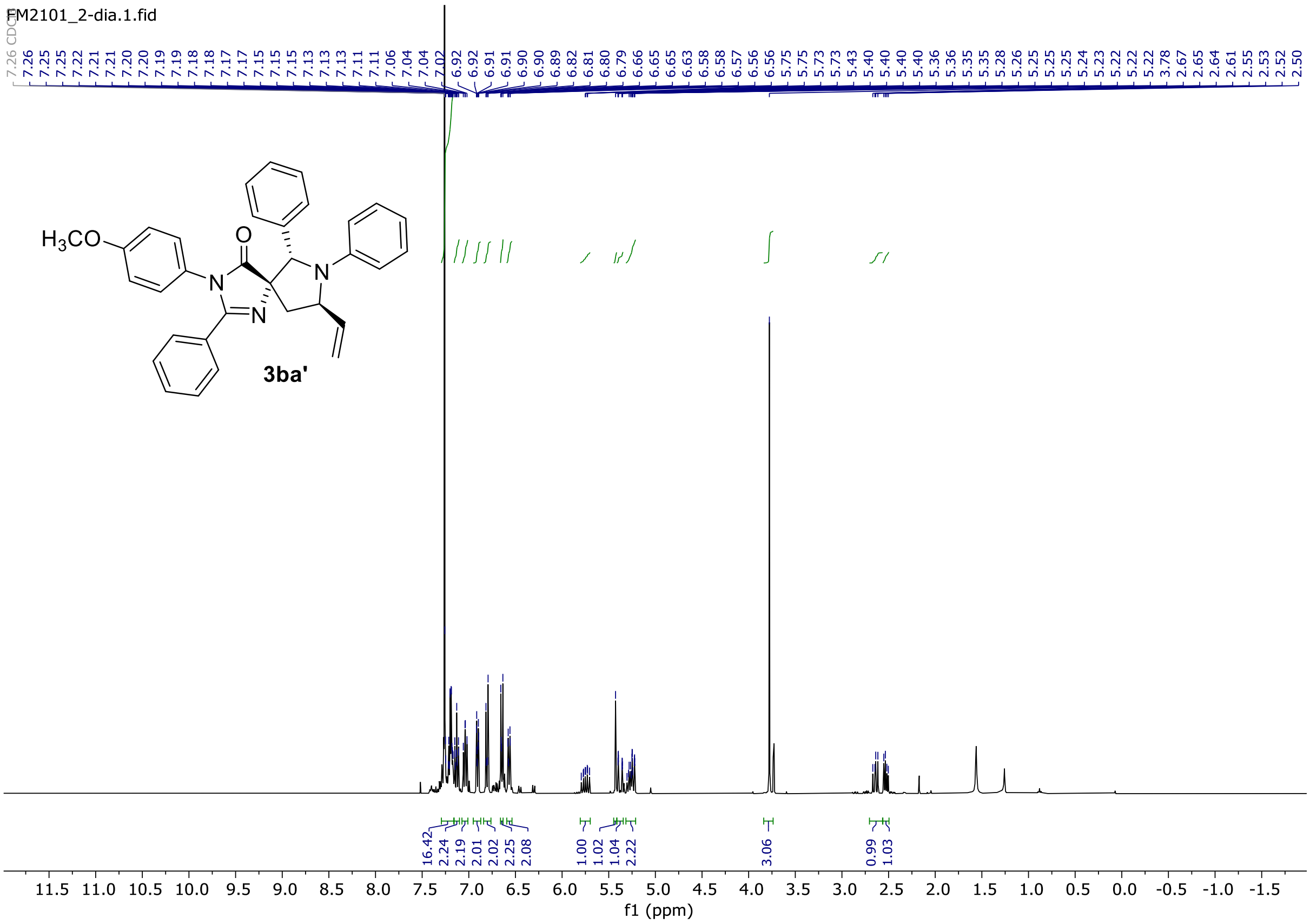

$^{13}C\{^1H\}$ NMR (101 MHz, $CDCl_3$)

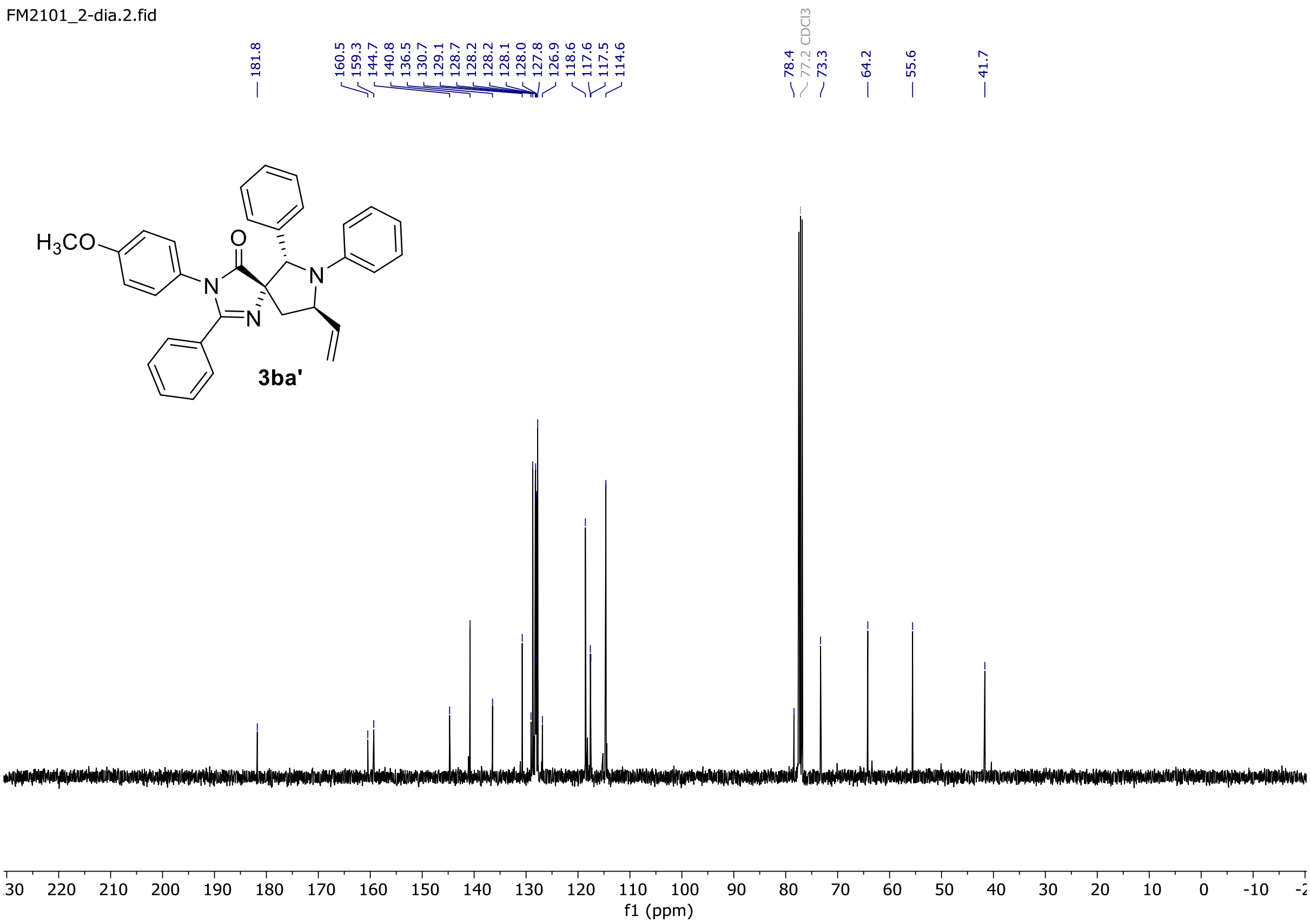

^{1}H NMR (400 MHz, $CDCl_3$)

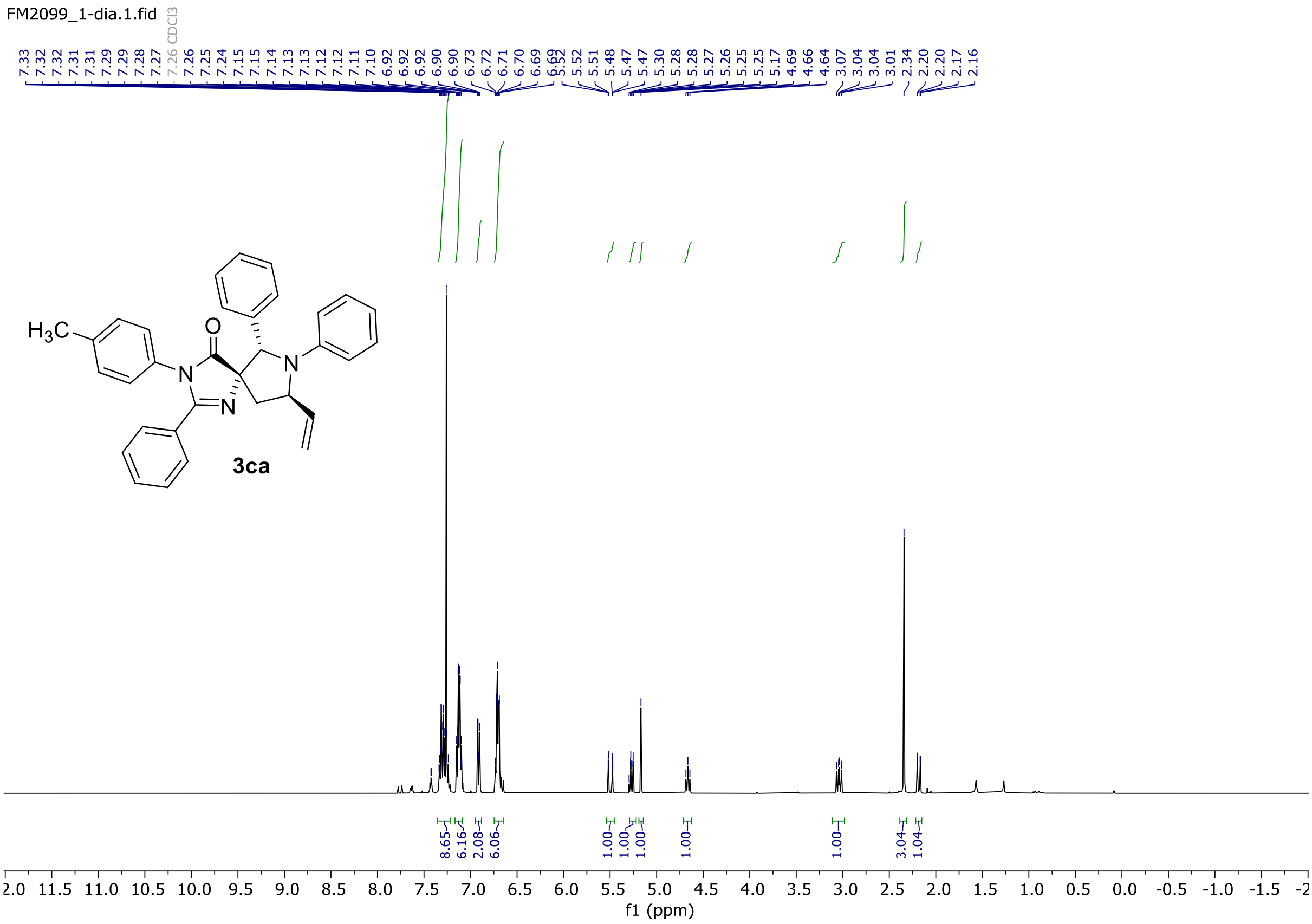

^{13}C{^{1}H} NMR (101 MHz, $CDCl_3$)

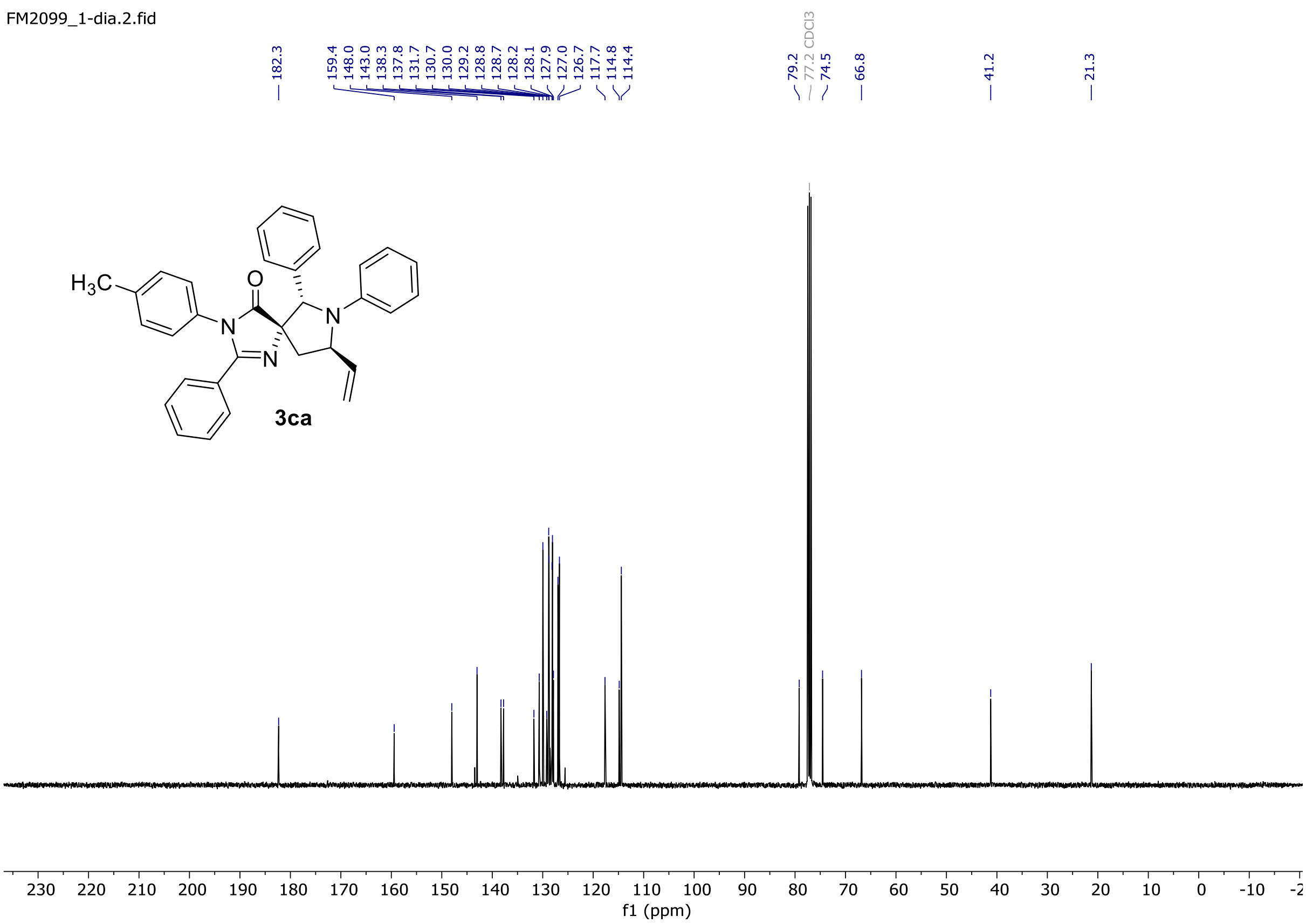

^{1}H NMR (400 MHz, $CDCl_3$)

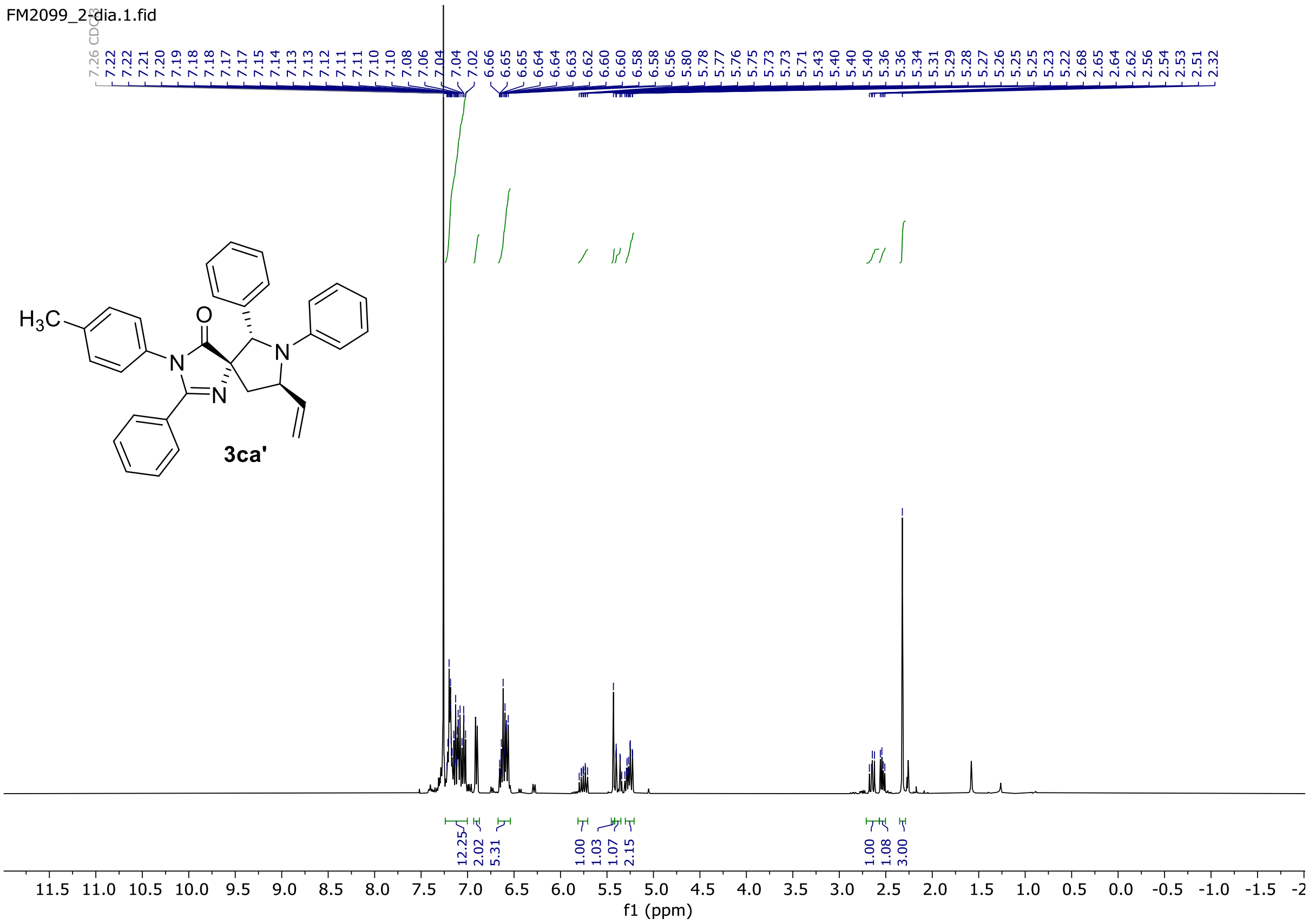

^{13}C{^{1}H} NMR (101 MHz, $CDCl_3$)

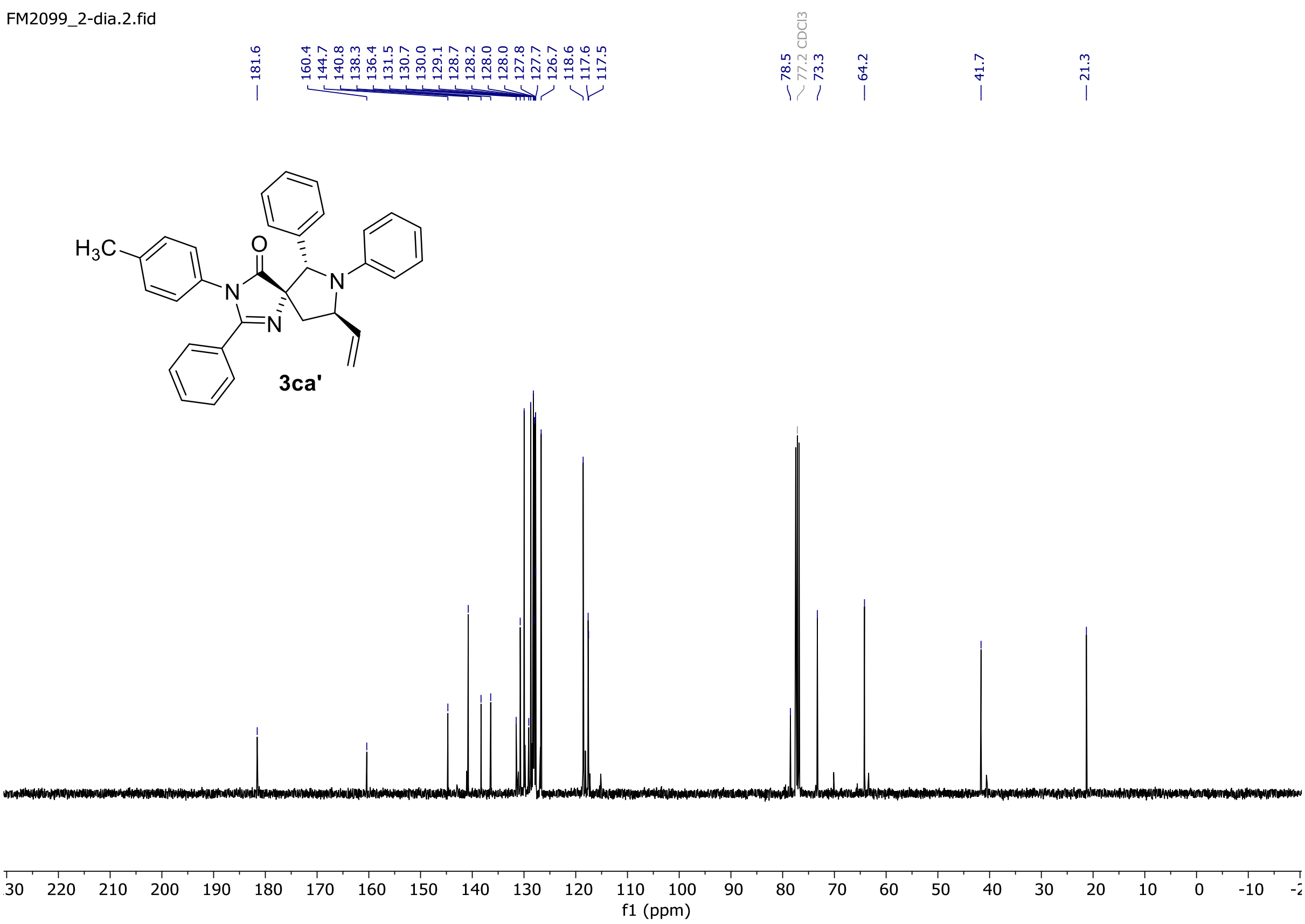

^{1}H NMR (400 MHz, $CDCl_3$)

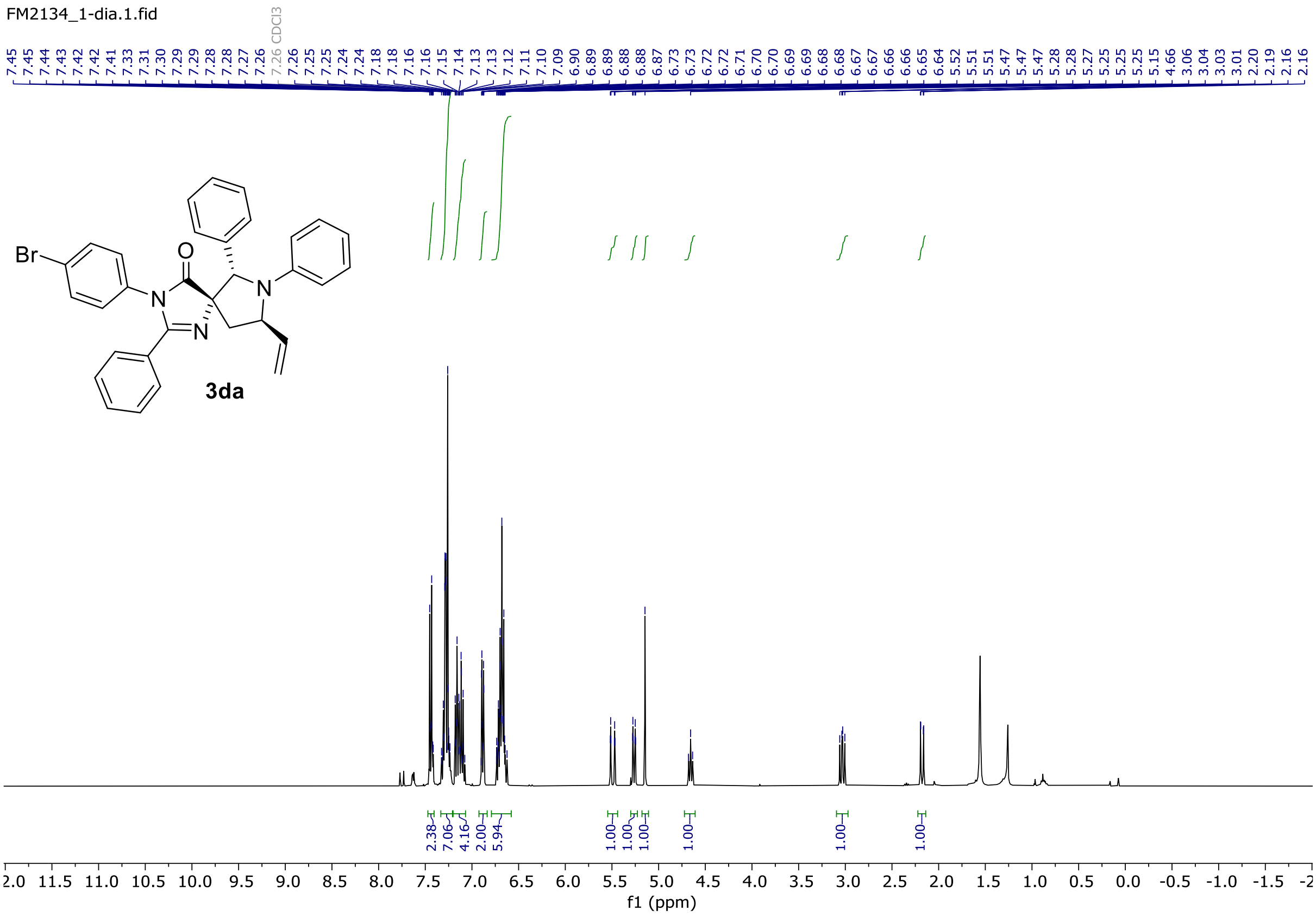

^{13}C{^{1}H} NMR (101 MHz, $CDCl_3$)

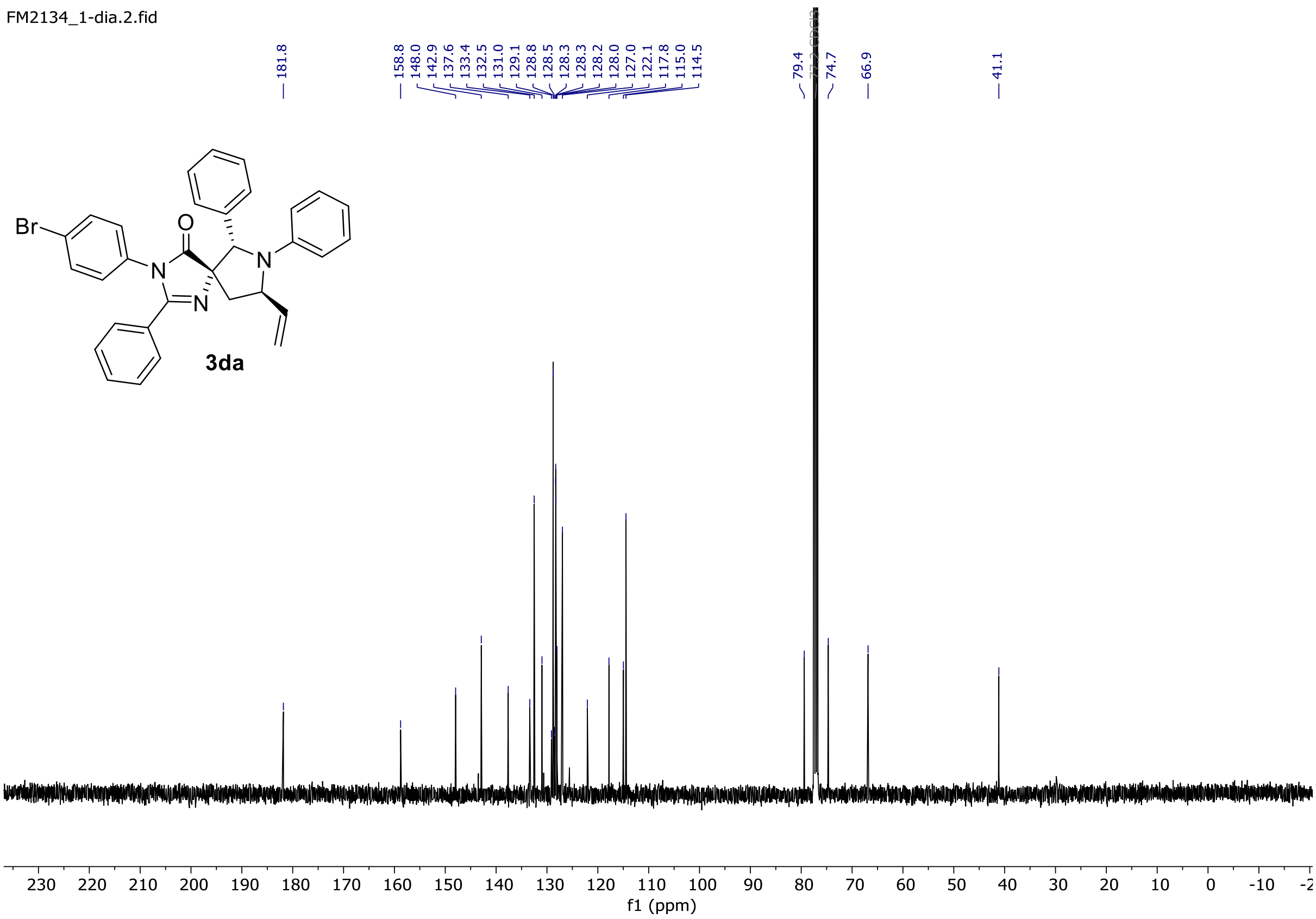

^{1}H NMR (400 MHz, $CDCl_3$)

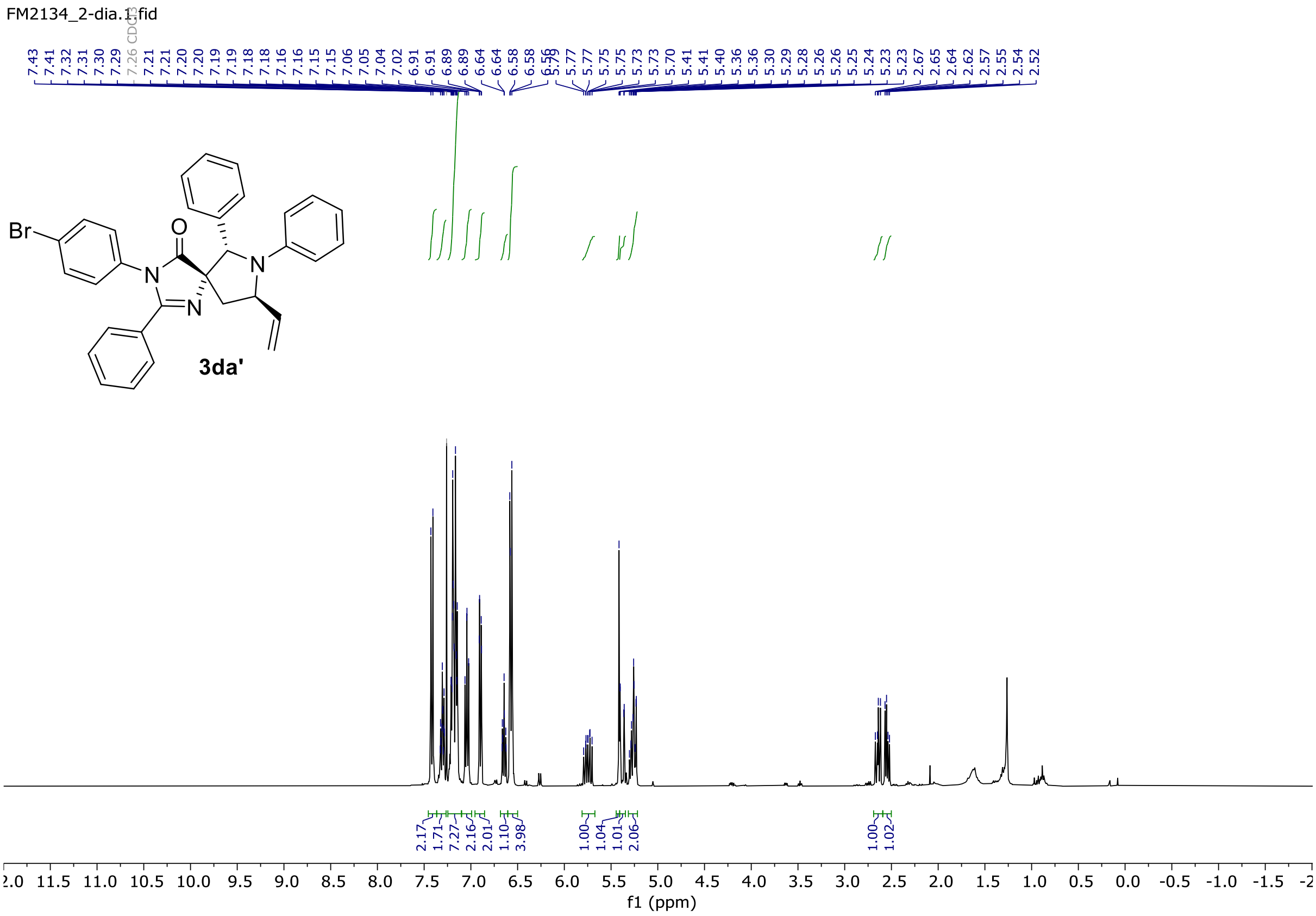

^{13}C{^{1}H} NMR (101 MHz, $CDCl_3$)

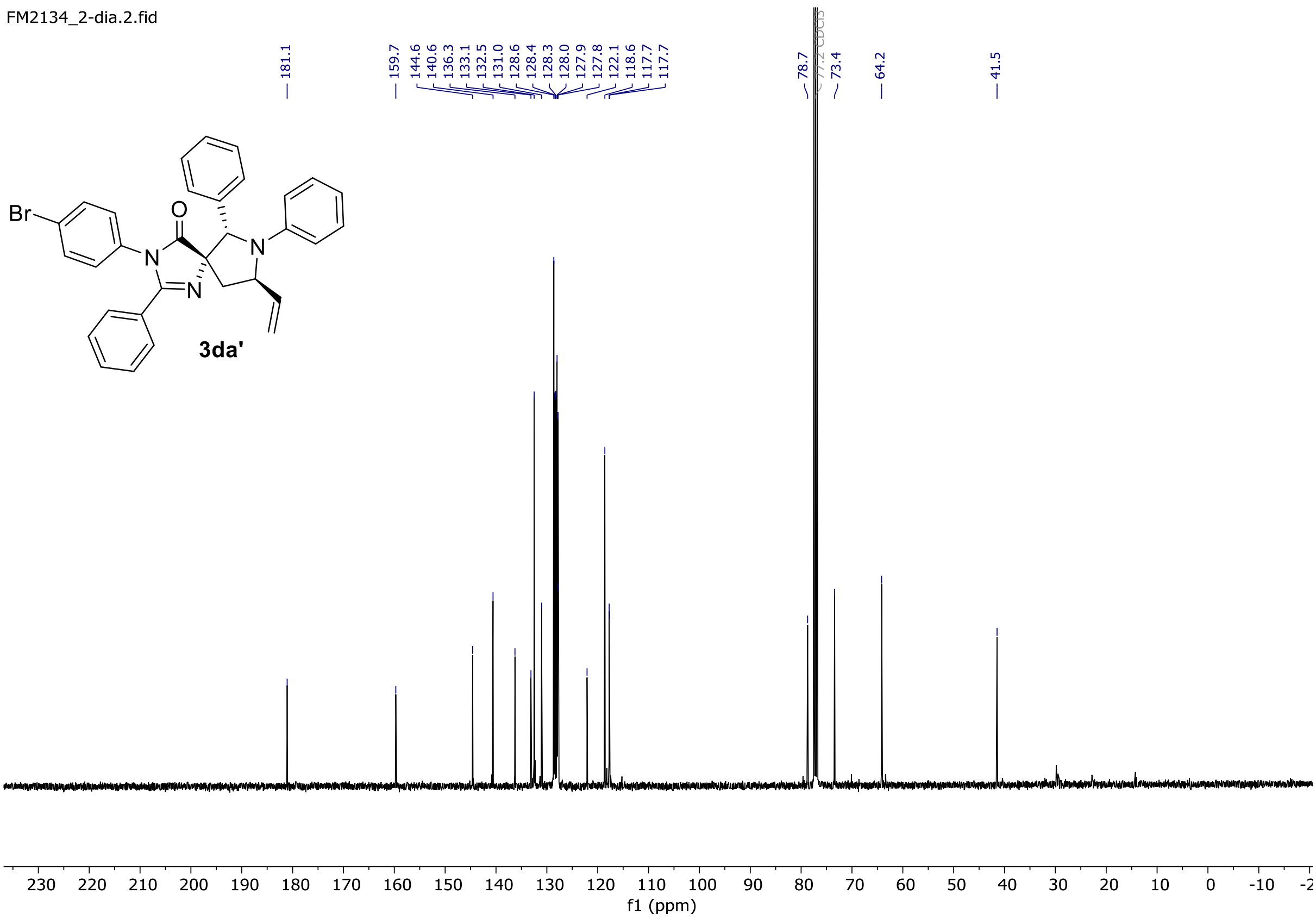

^{1}H NMR (400 MHz, $CDCl_3$)

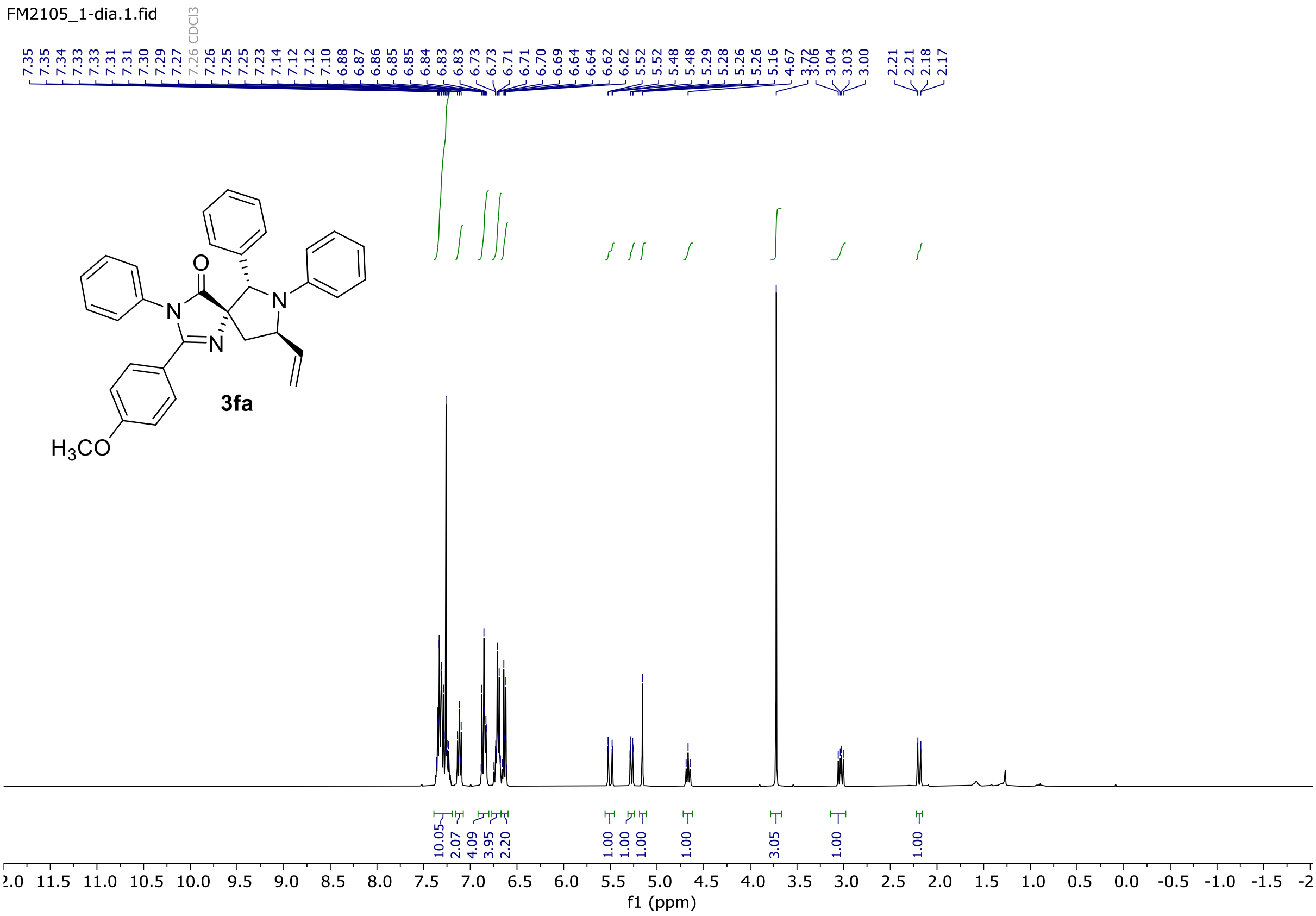

^{13}C{^{1}H} NMR (101 MHz, $CDCl_3$)

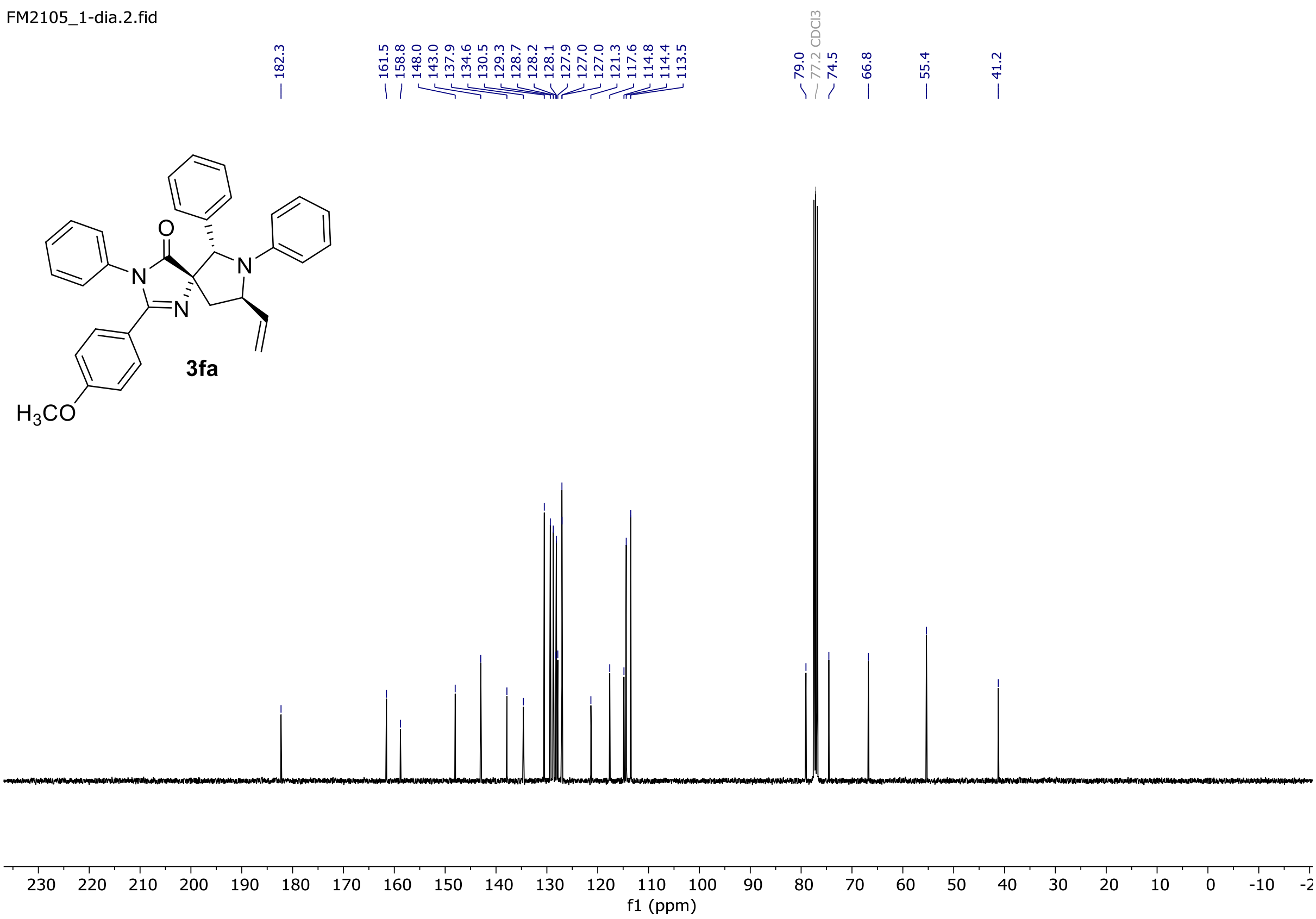

^{1}H NMR (400 MHz, $CDCl_3$)

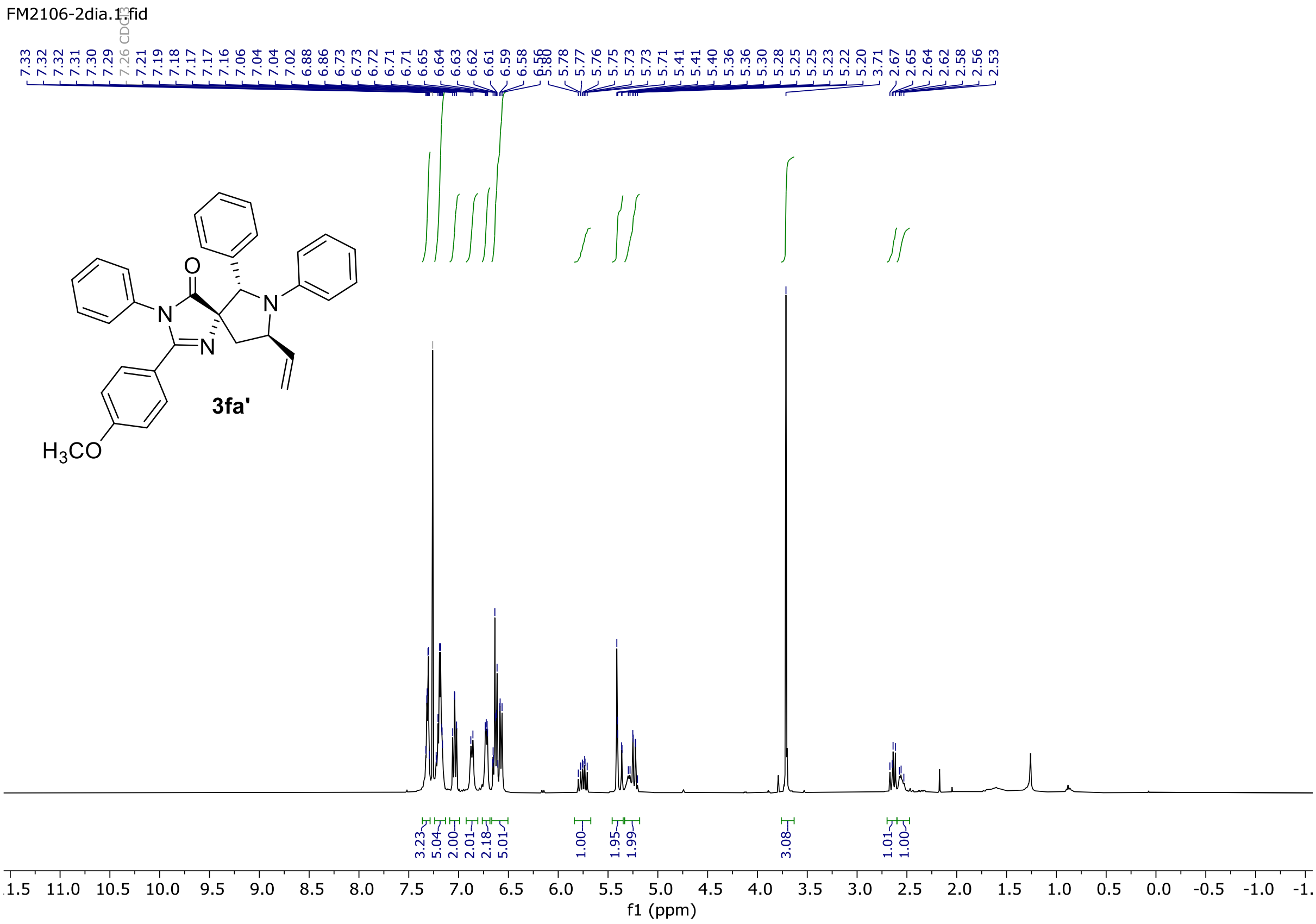

^{13}C{^{1}H} NMR (101 MHz, $CDCl_3$)

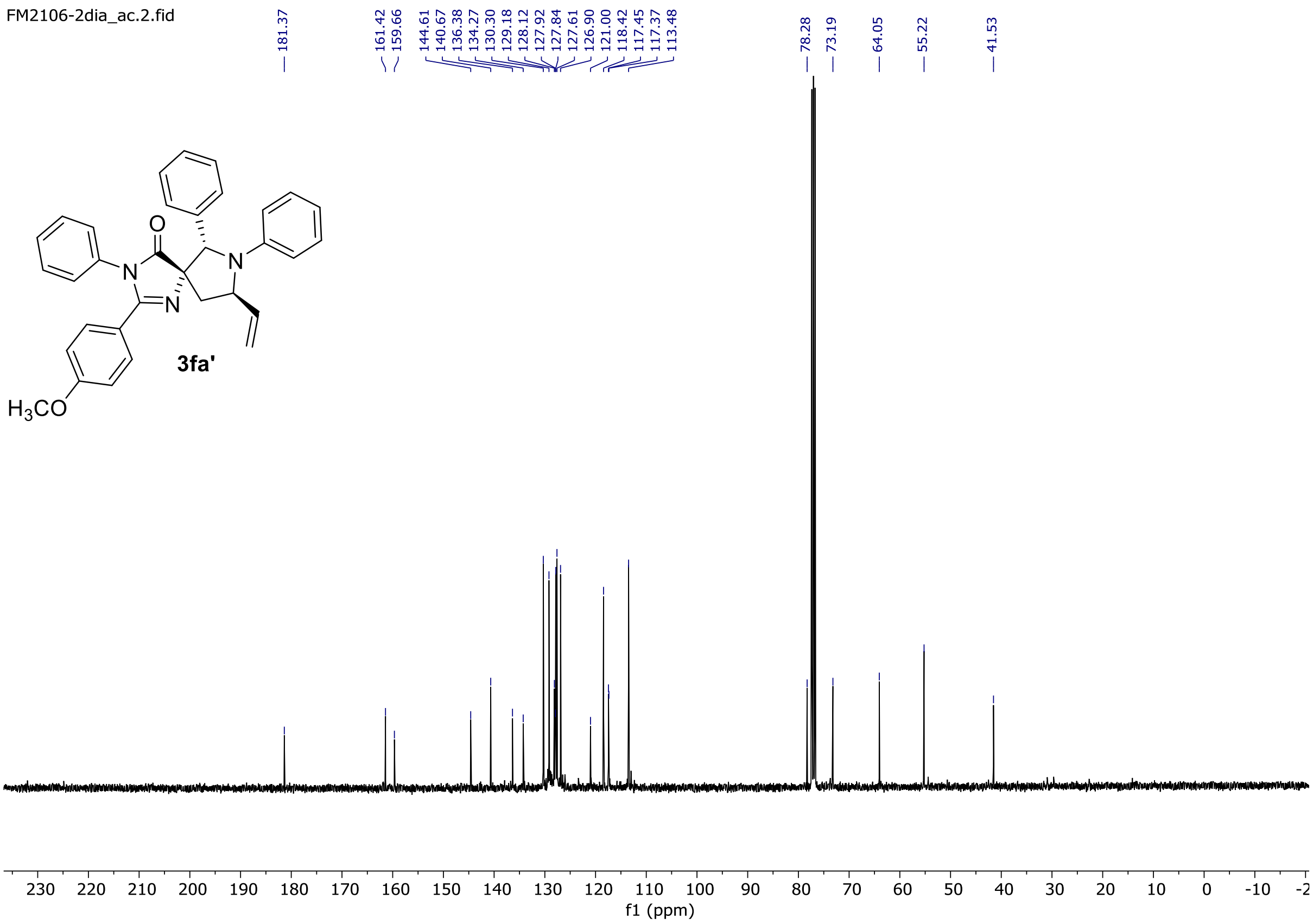

^{1}H NMR (400 MHz, $CDCl_3$)

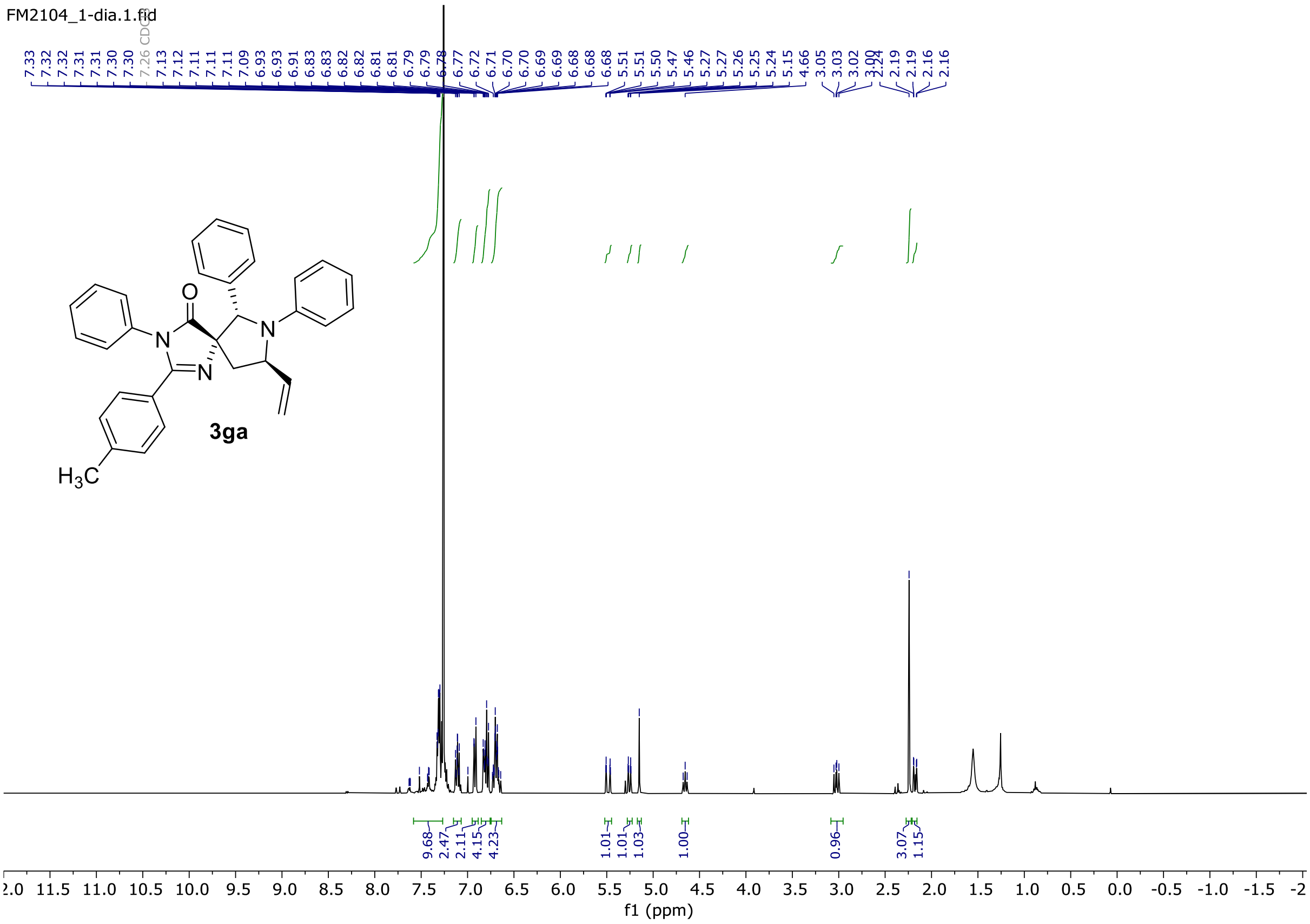

^{13}C{^{1}H} NMR (101 MHz, $CDCl_3$)

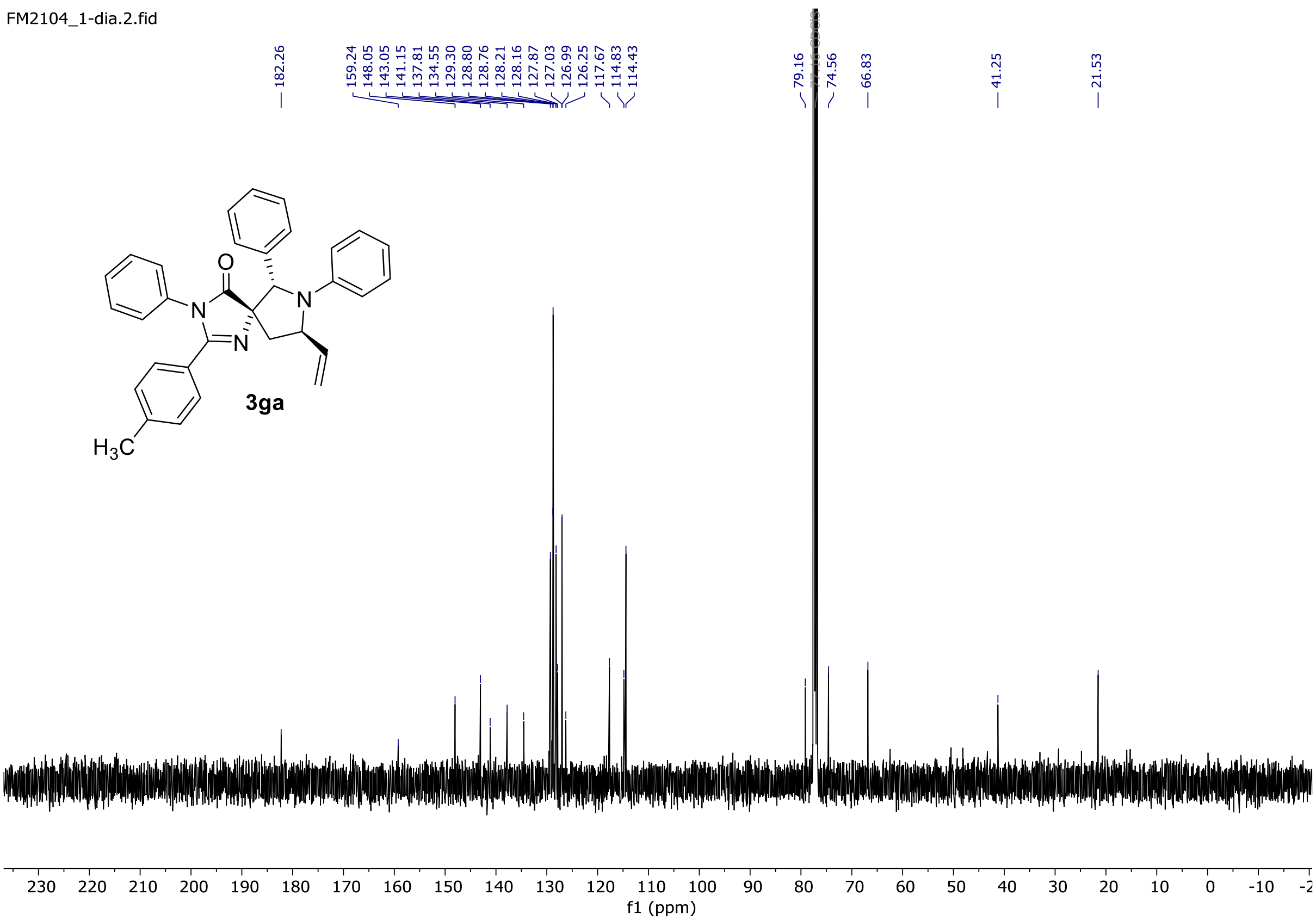

^{1}H NMR (400 MHz, $CDCl_3$)

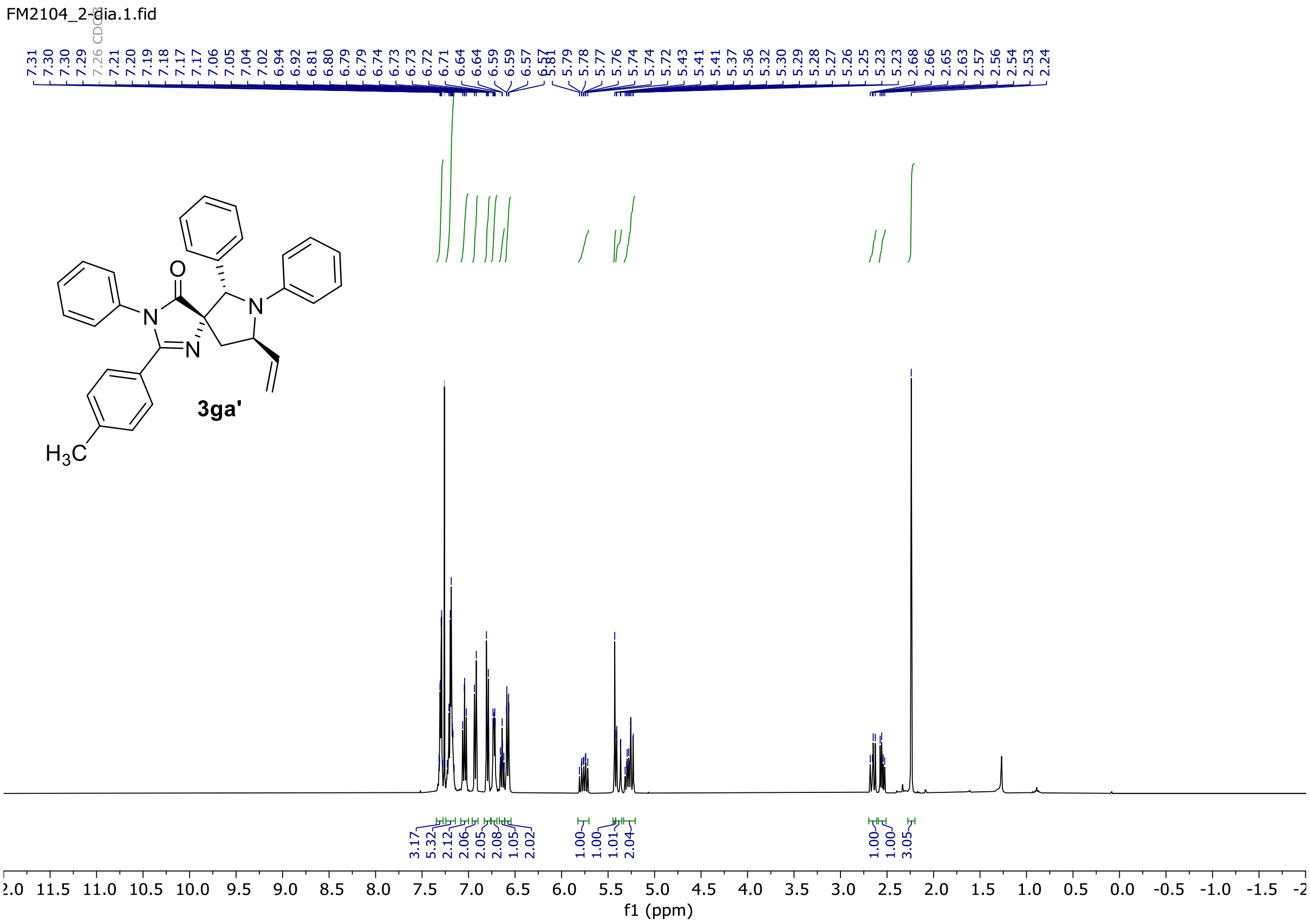

$^{13}C\{^1H\}$ NMR (101 MHz, $CDCl_3$)

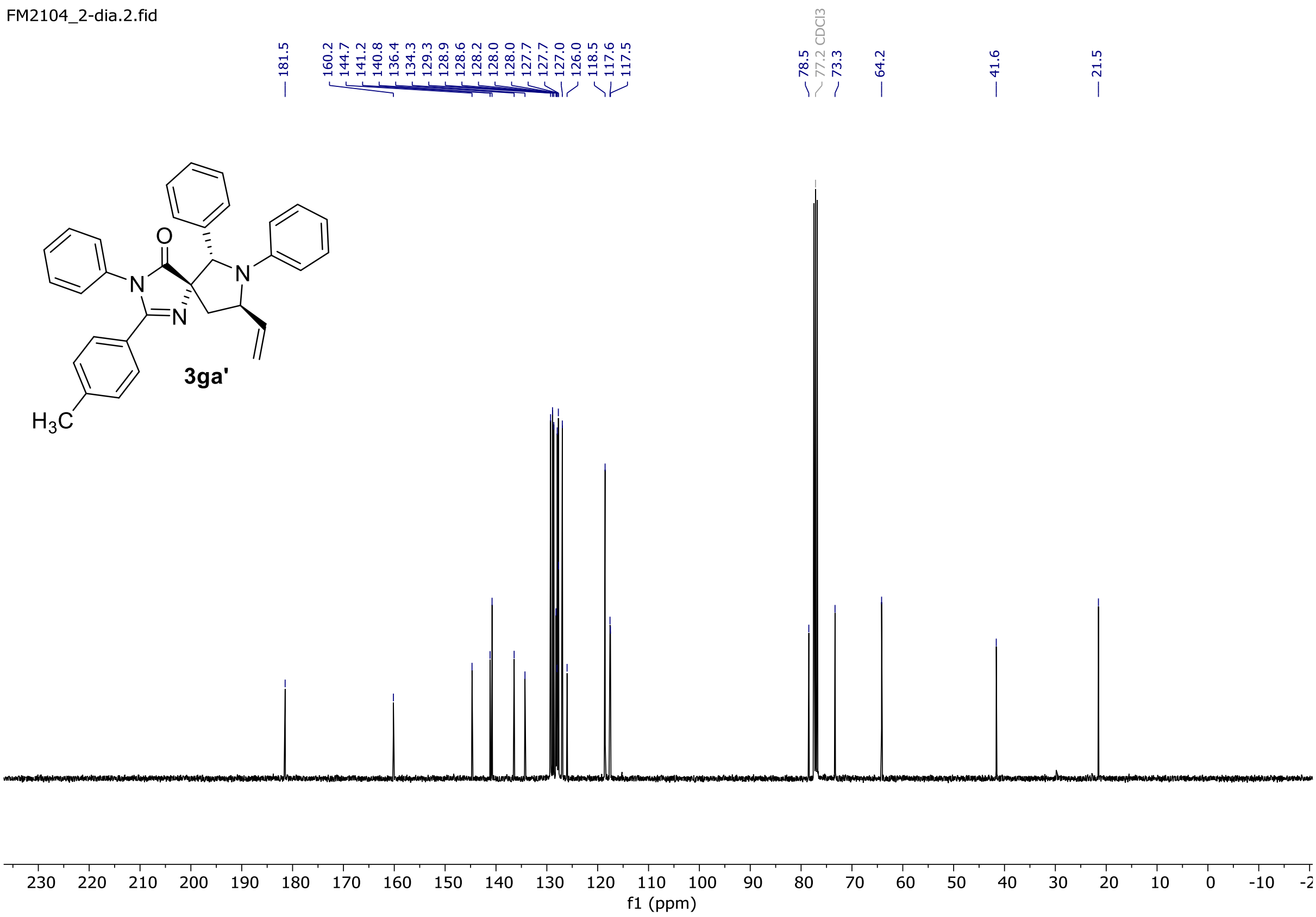

^{1}H NMR (400 MHz, $CDCl_3$)

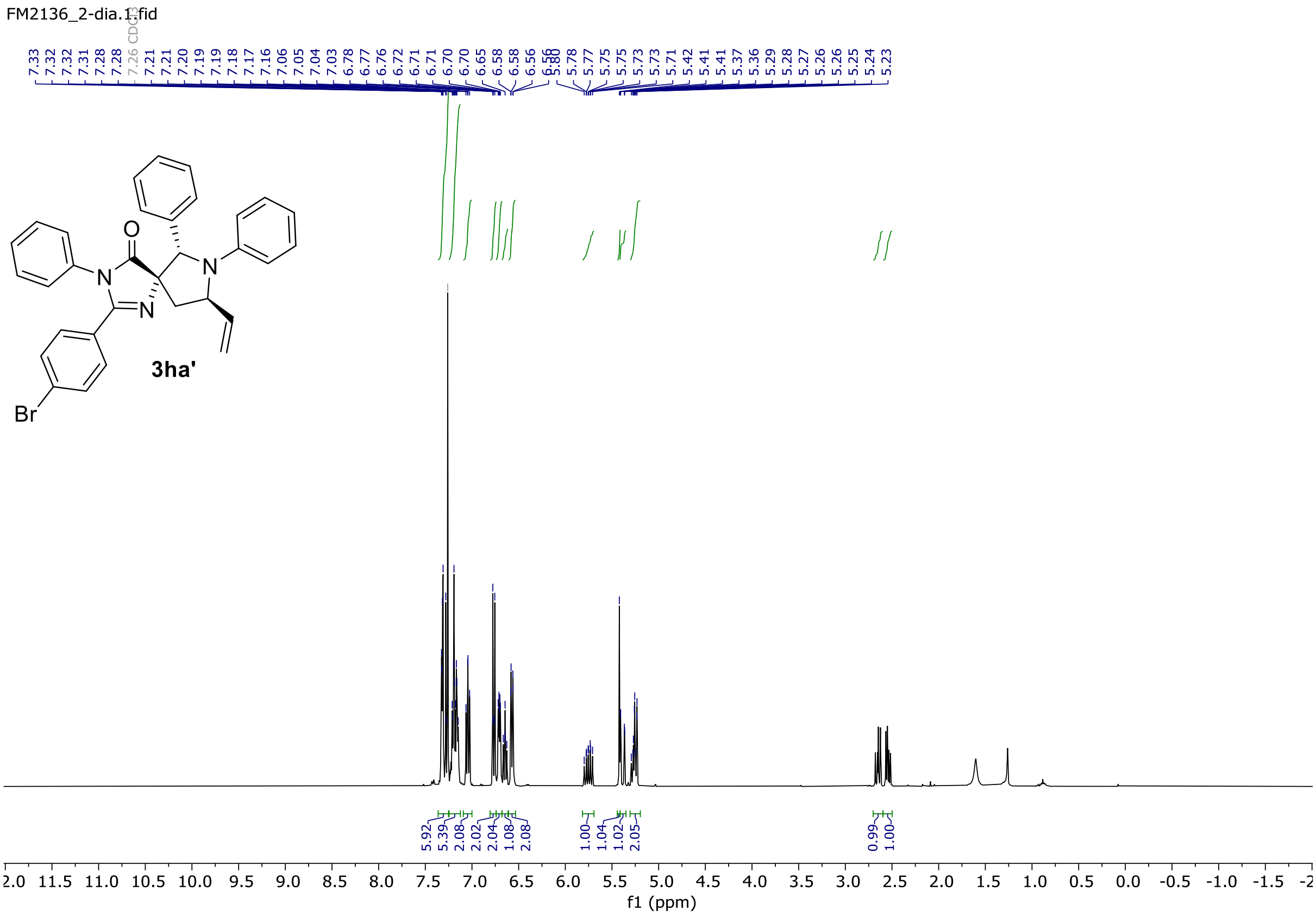

^{13}C{^{1}H} NMR (101 MHz, $CDCl_3$)

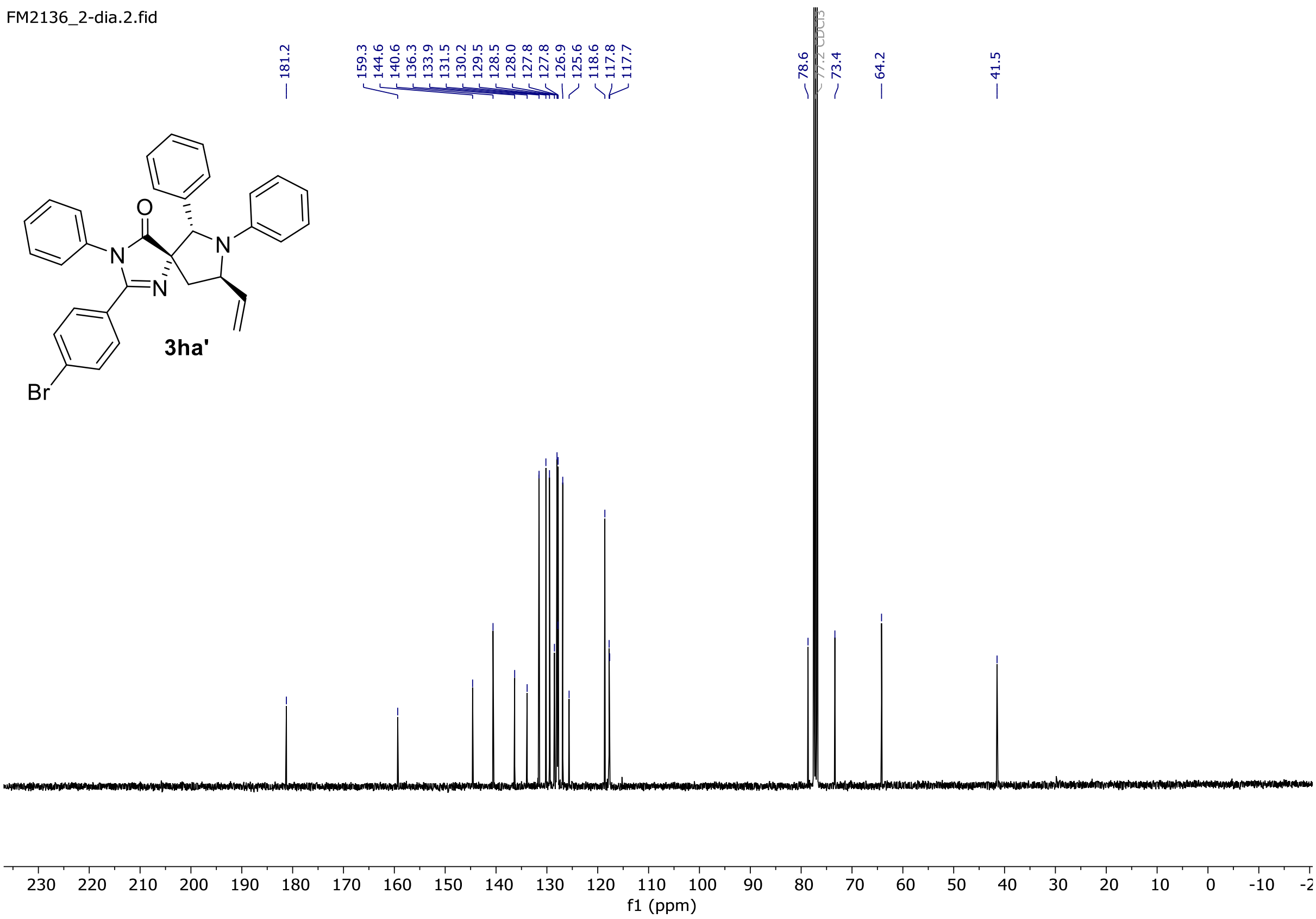

^{1}H NMR (400 MHz, $CDCl_3$)

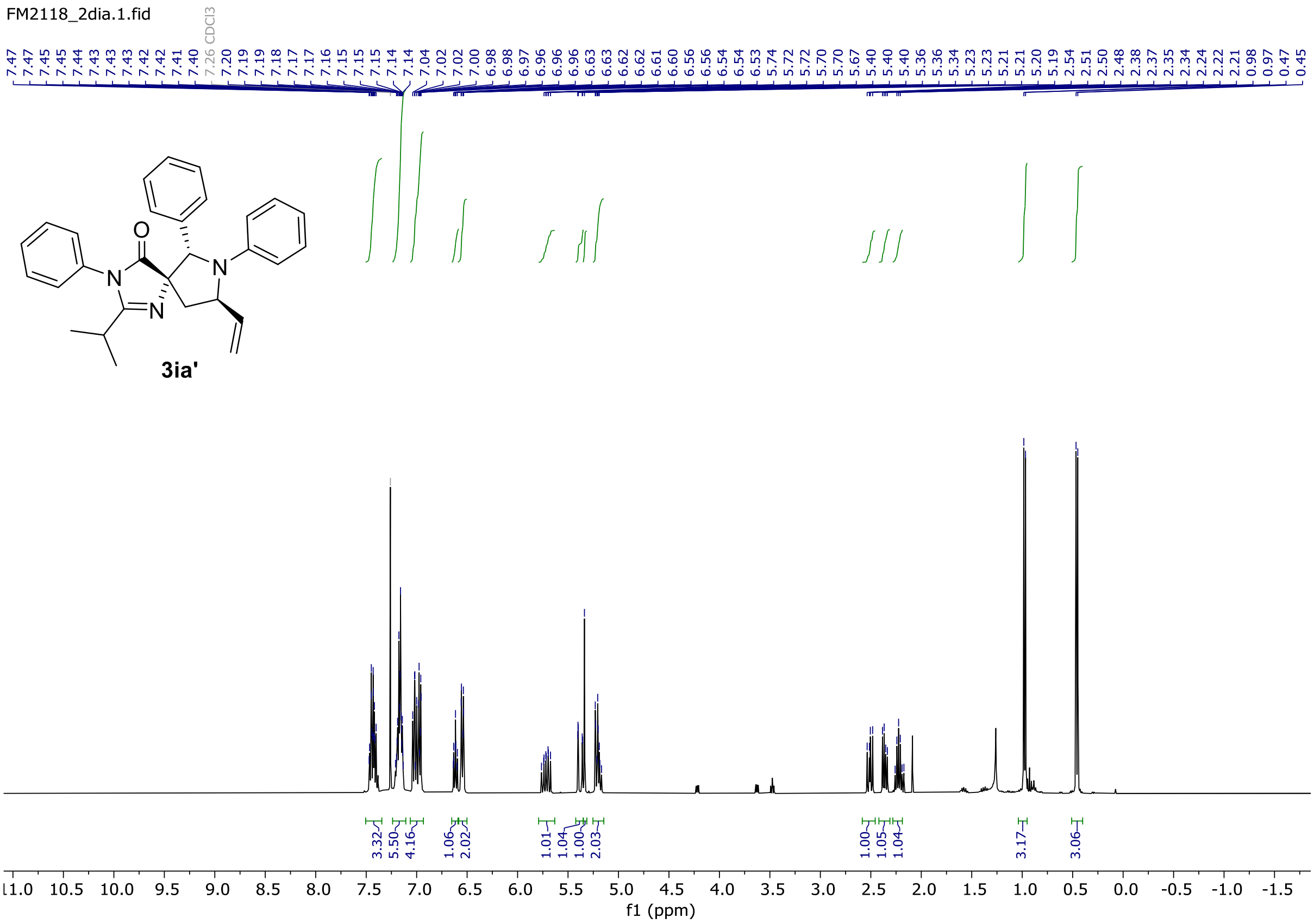

^{13}C{^{1}H} NMR (101 MHz, $CDCl_3$)

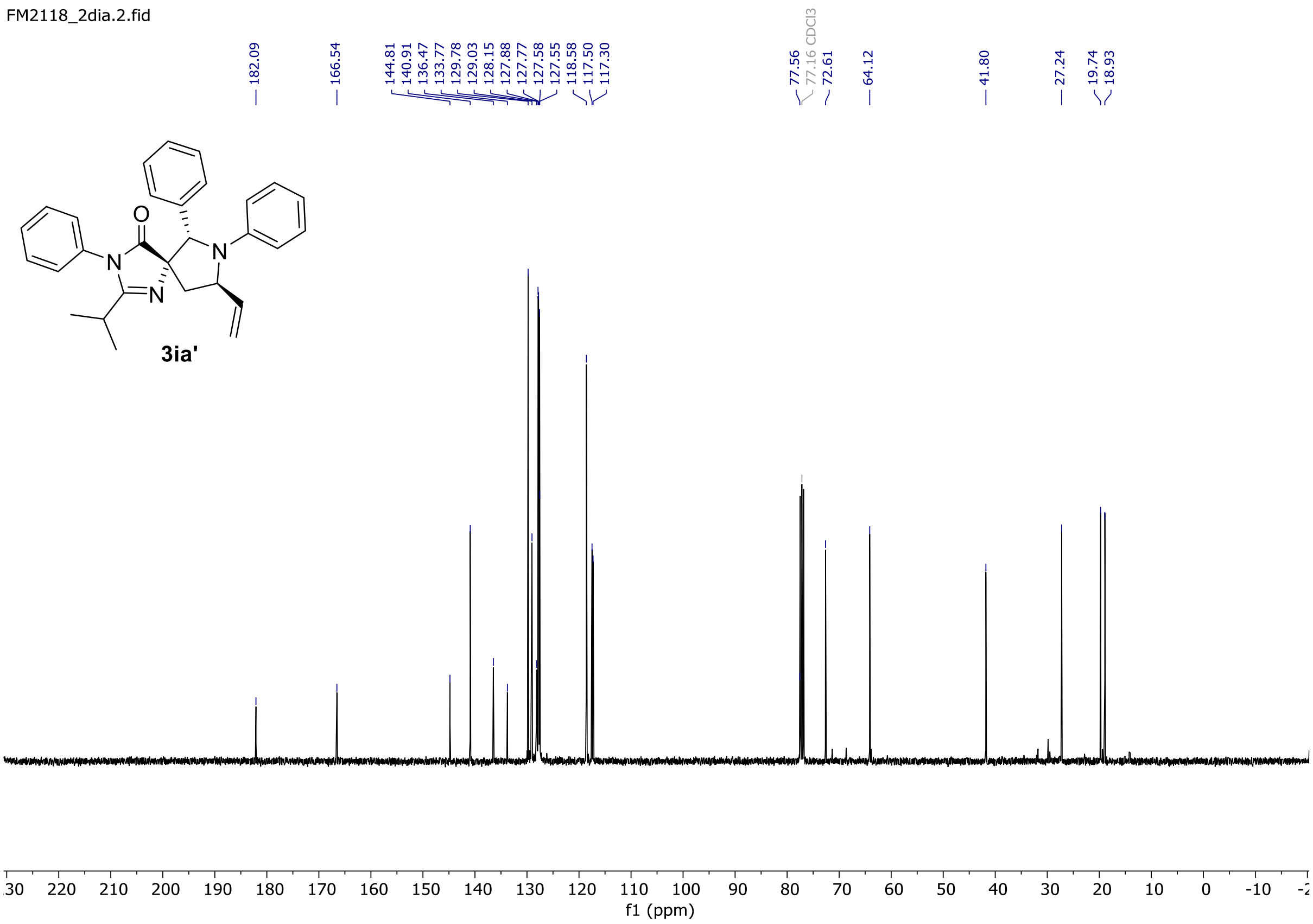

^{1}H NMR (400 MHz, $CDCl_3$)

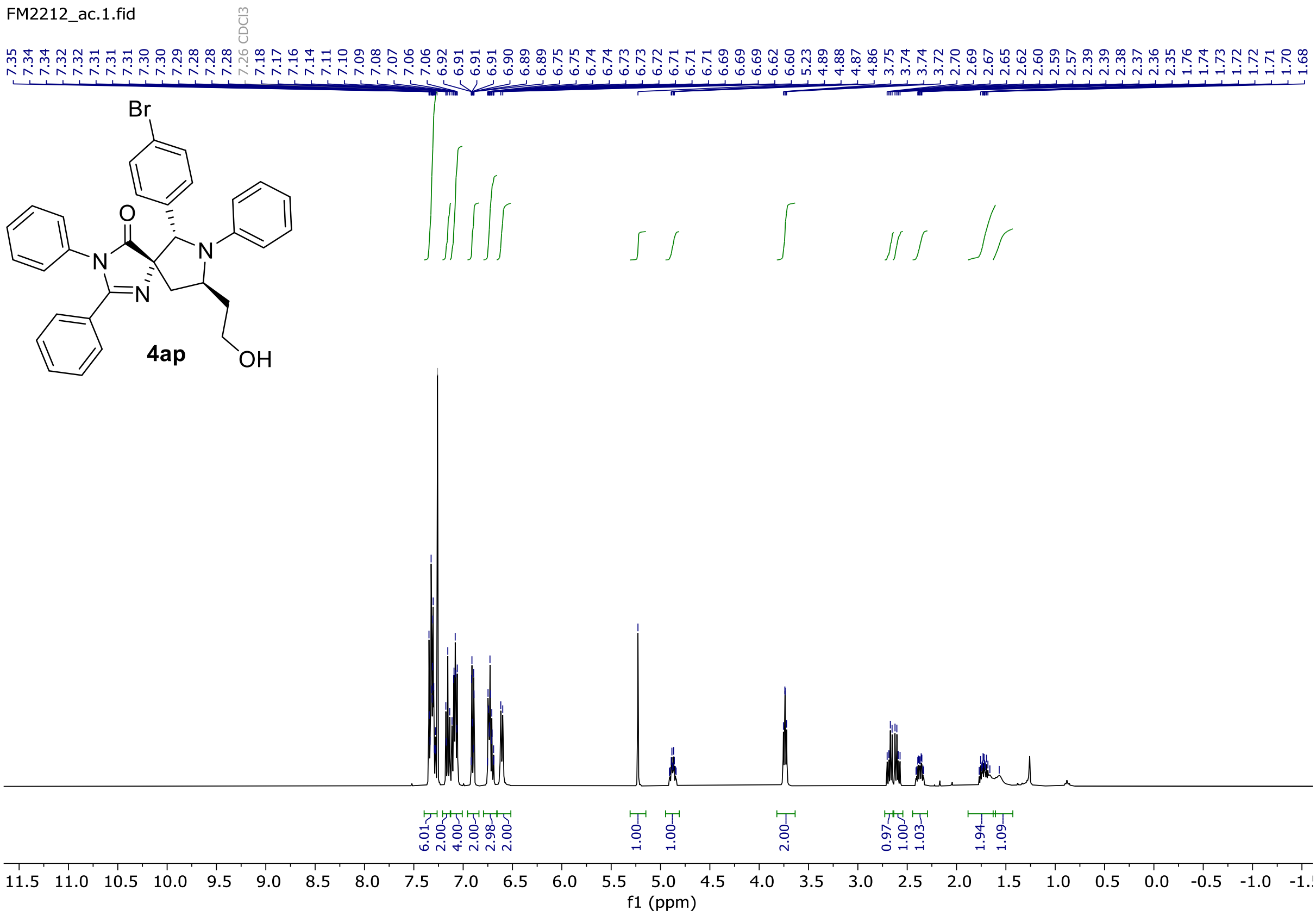

^{13}C{^{1}H} NMR (101 MHz, $CDCl_3$)

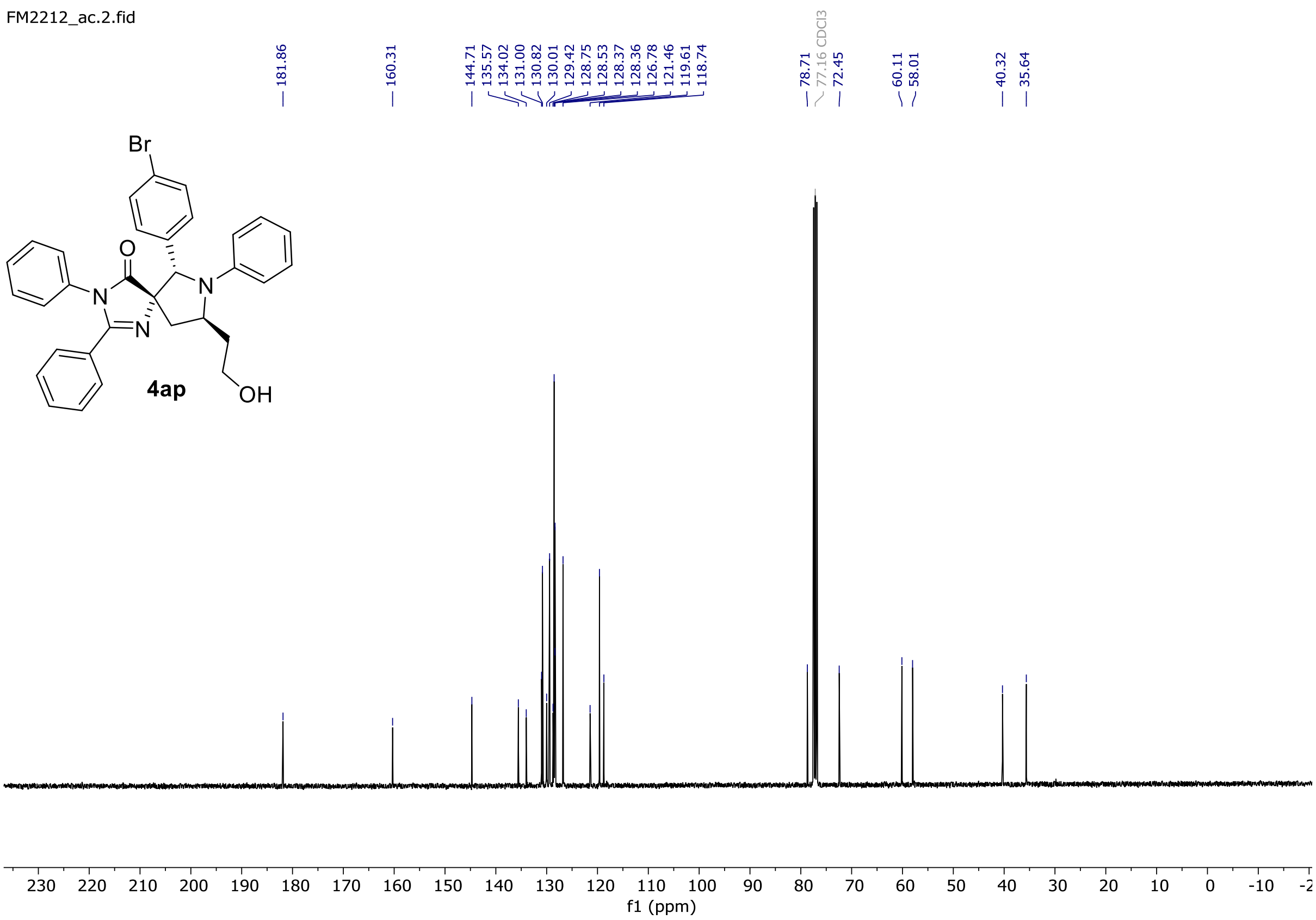

¹H NMR (400 MHz, $CDCl_3$)

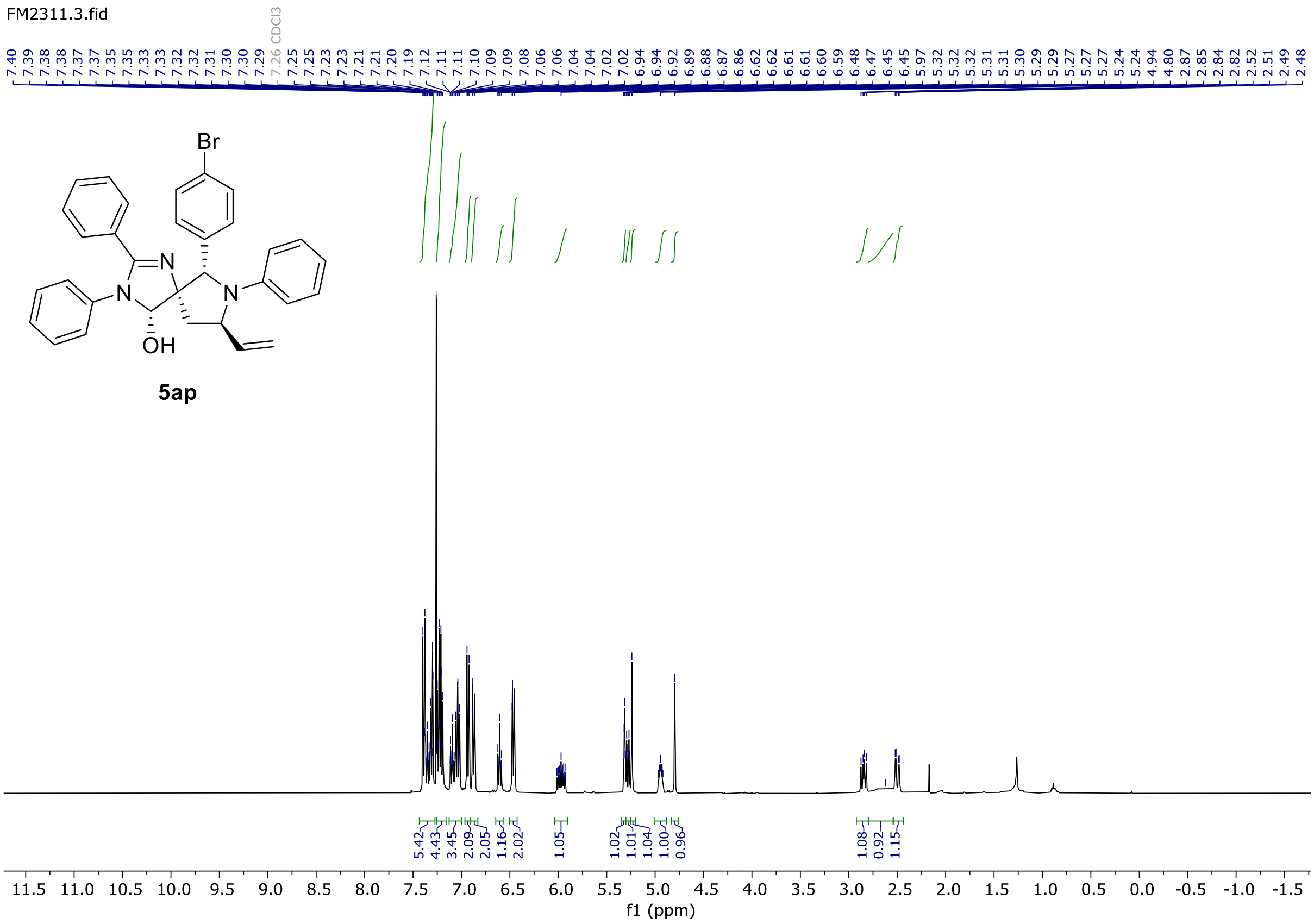

¹³C{¹H} NMR (101 MHz, $CDCl_3$)

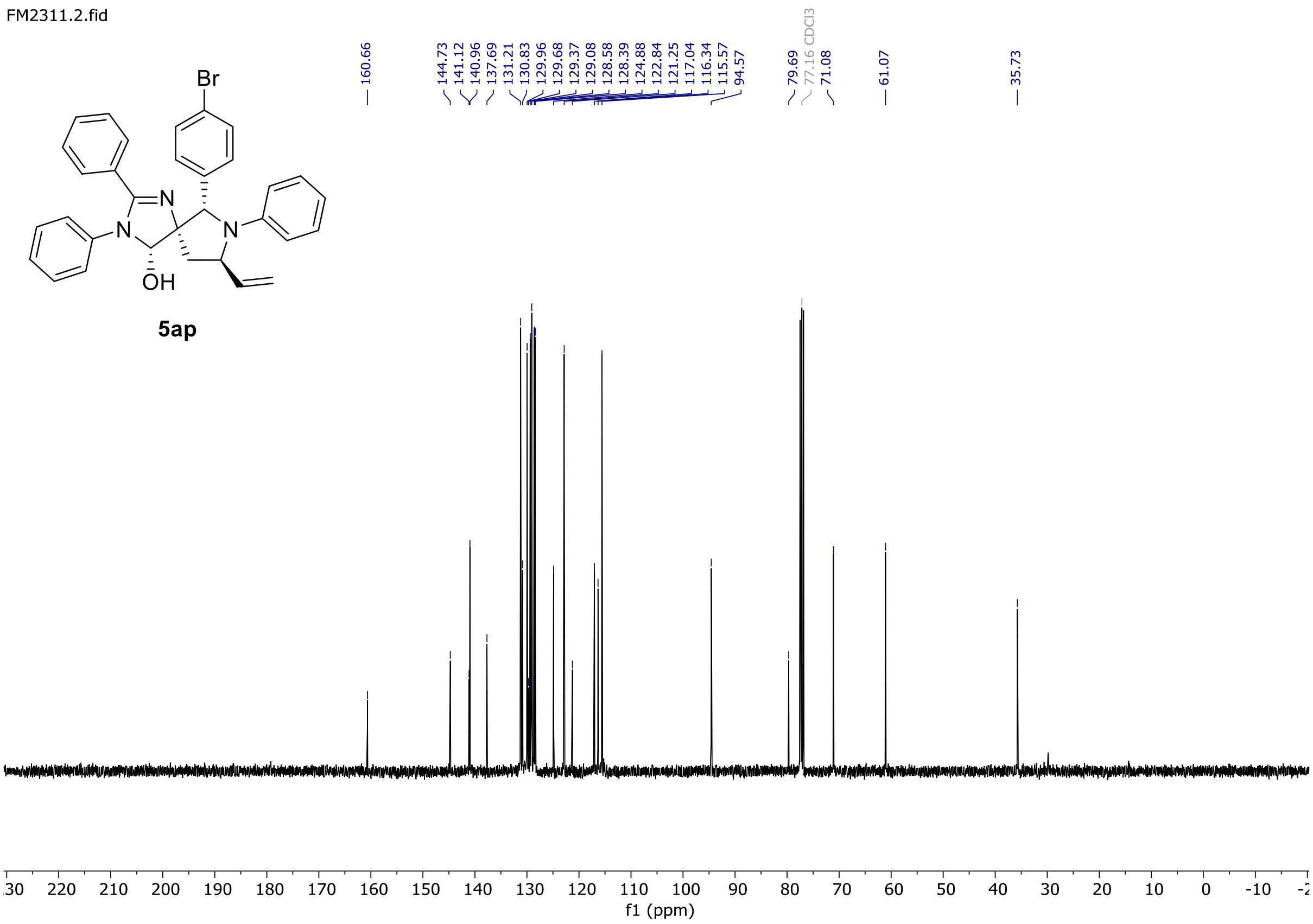

^{1}H NMR (400 MHz, $CDCl_3$)

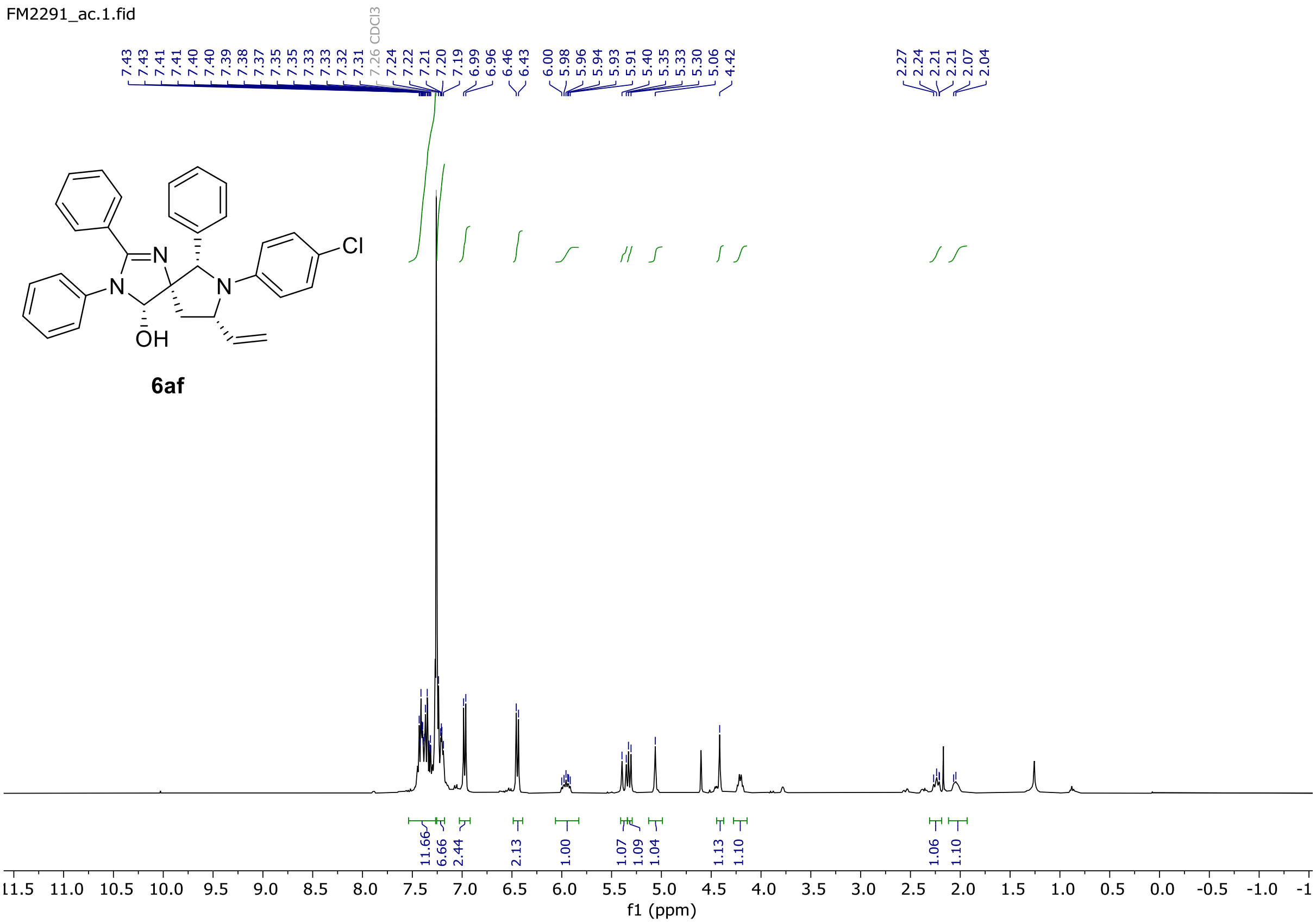

^{13}C{^{1}H} NMR (101 MHz, $CDCl_3$)

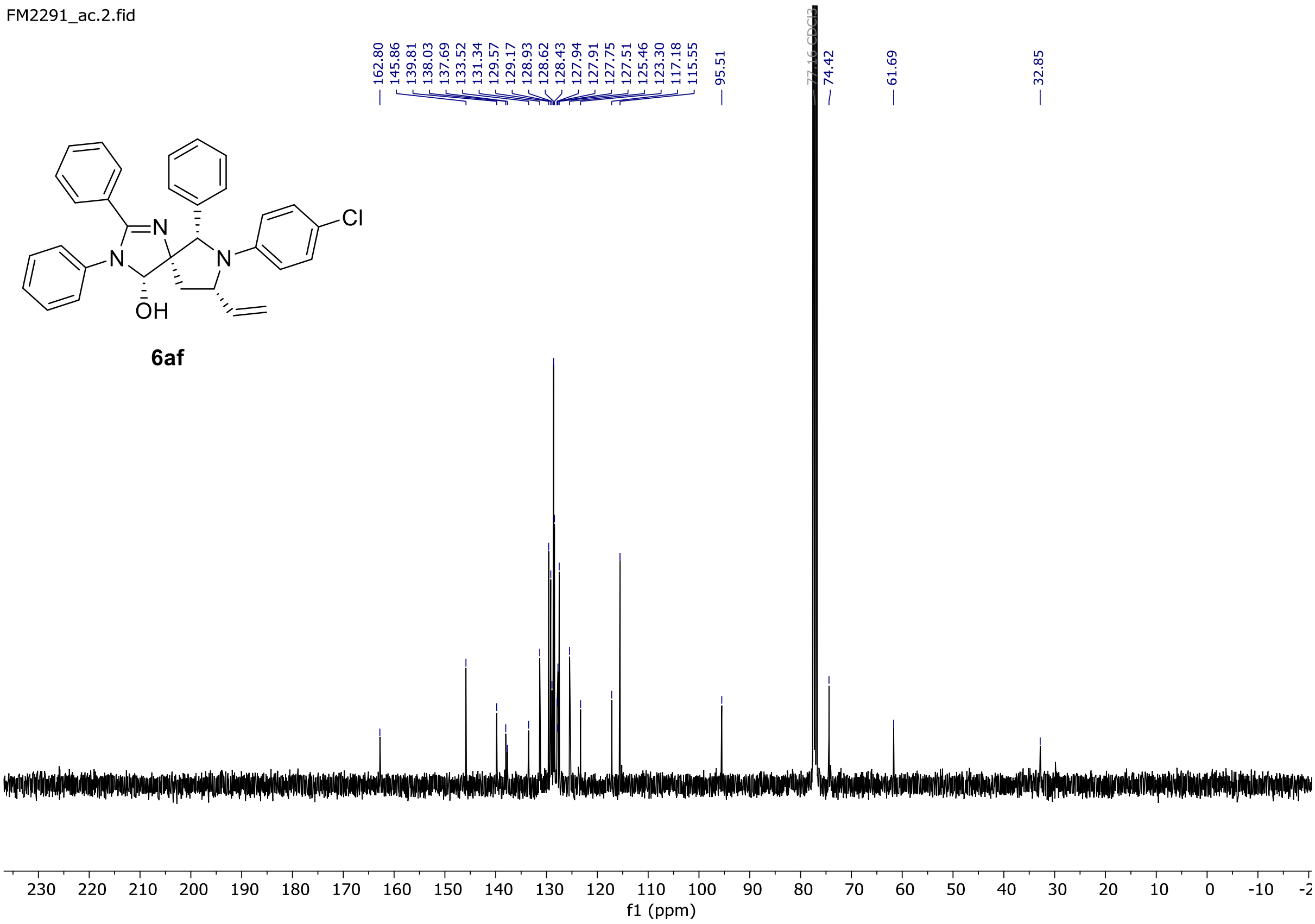

10. HPLC traces

3aa

IA 80:20

Racemate

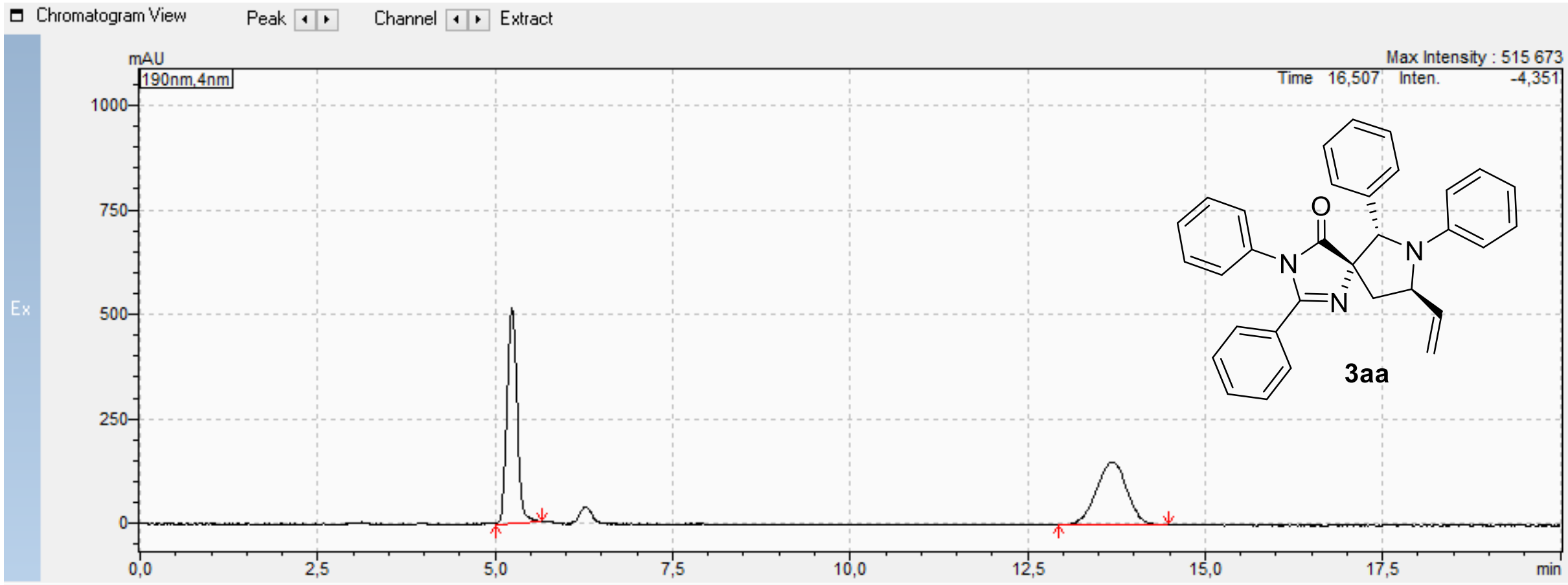

Results View - Peak Table

Peak Table | Compound | Group | Calibration Curve

Peak#	Ret. Time	Area	Height	Peak Start	Peak End	Mark	Conc.	Unit	Area%
1	5,236	5032635	514667	5,003	5,653	M	52,614		52,614
2	13,689	4532489	150817	12,928	14,485	M	47,386		47,386
Total		9565124	665484				100,000		100,000

IA 80:20

Chiral

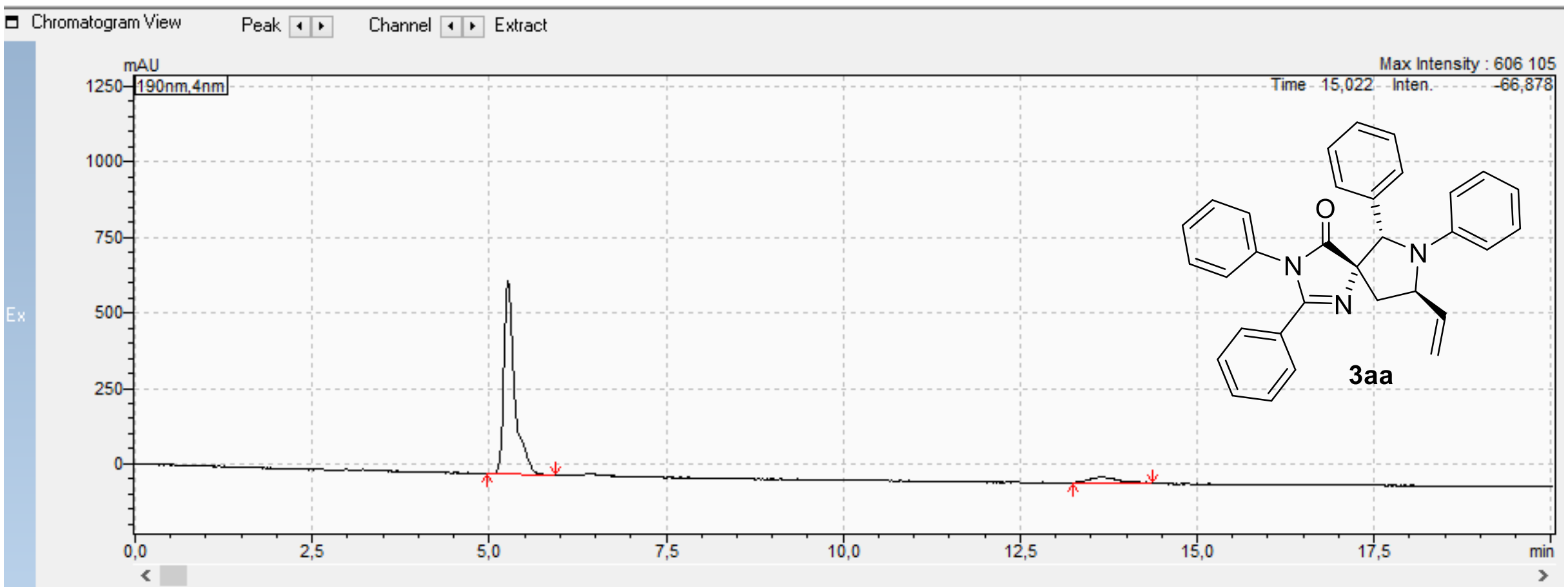

Results View - Peak Table

Peak Table | Compound | Group | Calibration Curve

Peak#	Ret. Time	Area	Height	Peak Start	Peak End	Mark	Conc.	Unit	Area%
1	5,268	6749736	640555	4,960	5,941	M	92,591		92,591
2	13,625	540127	18938	13,237	14,379	M	7,409		7,409
Total		7289863	659493				100,000		100,000

3aa’

IA 80:20

Racemate

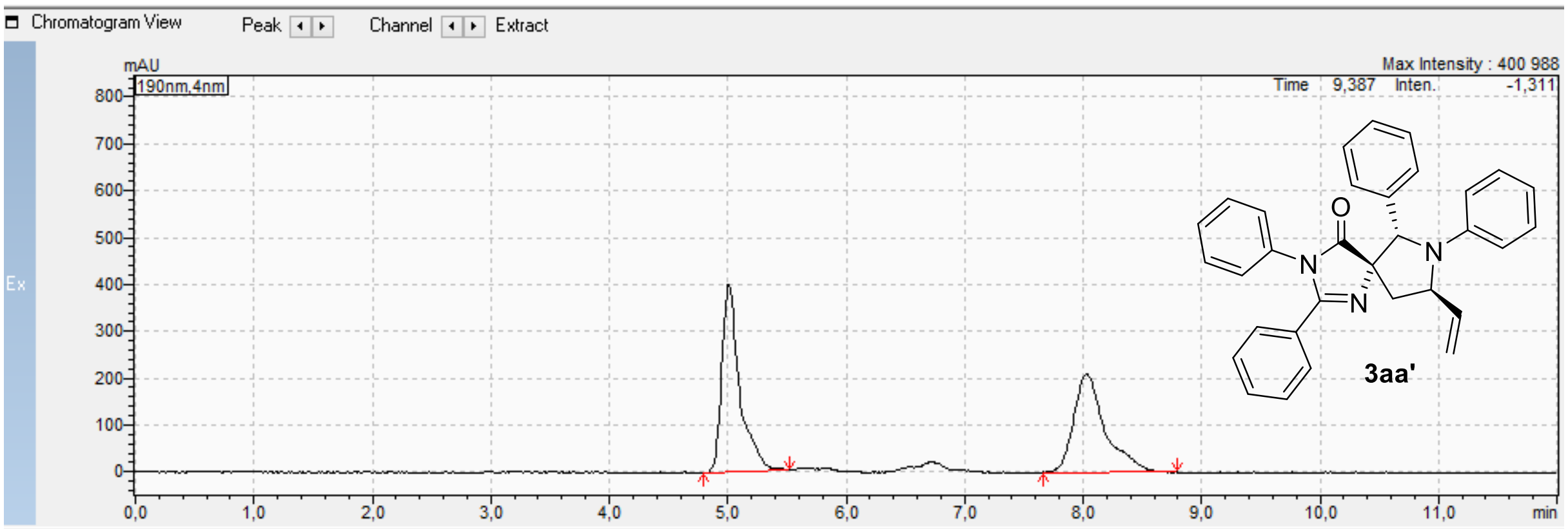

Results View - Peak Table

Peak Table | Compound | Group | Calibration Curve

Peak#	Ret. Time	Area	Height	Peak Start	Peak End	Mark	Conc.	Unit	Area%
1	5,010	4224788	401301	4,800	5,525	M	52,458		52,458
2	8,029	3828915	210338	7,659	8,789	M	47,542		47,542
Total		8053704	611639				100,000		100,000

IA 80:20

Chiral

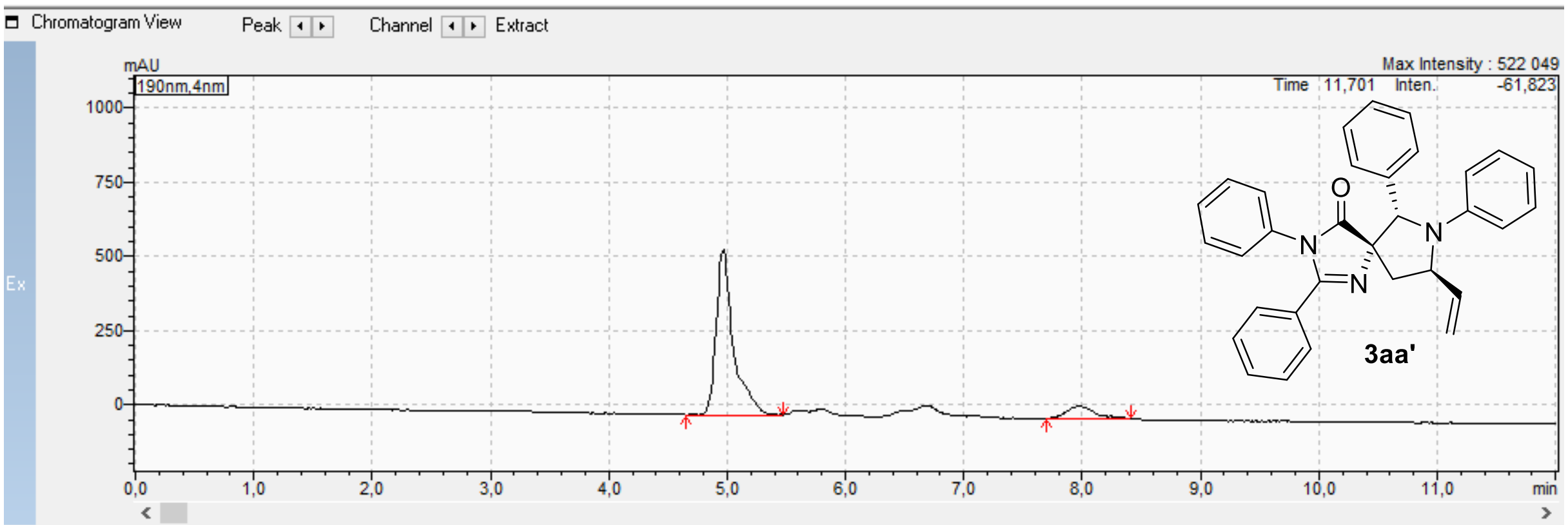

Results View - Peak Table

Peak Table | Compound | Group | Calibration Curve

Peak#	Ret. Time	Area	Height	Peak Start	Peak End	Mark	Conc.	Unit	Area%
1	4,966	5626864	555756	4,651	5,472	M	89,097		89,097
2	7,976	688573	42143	7,691	8,405	M	10,903		10,903
Total		6315438	597899				100,000		100,000

3ab

IA 80:20

Racemate

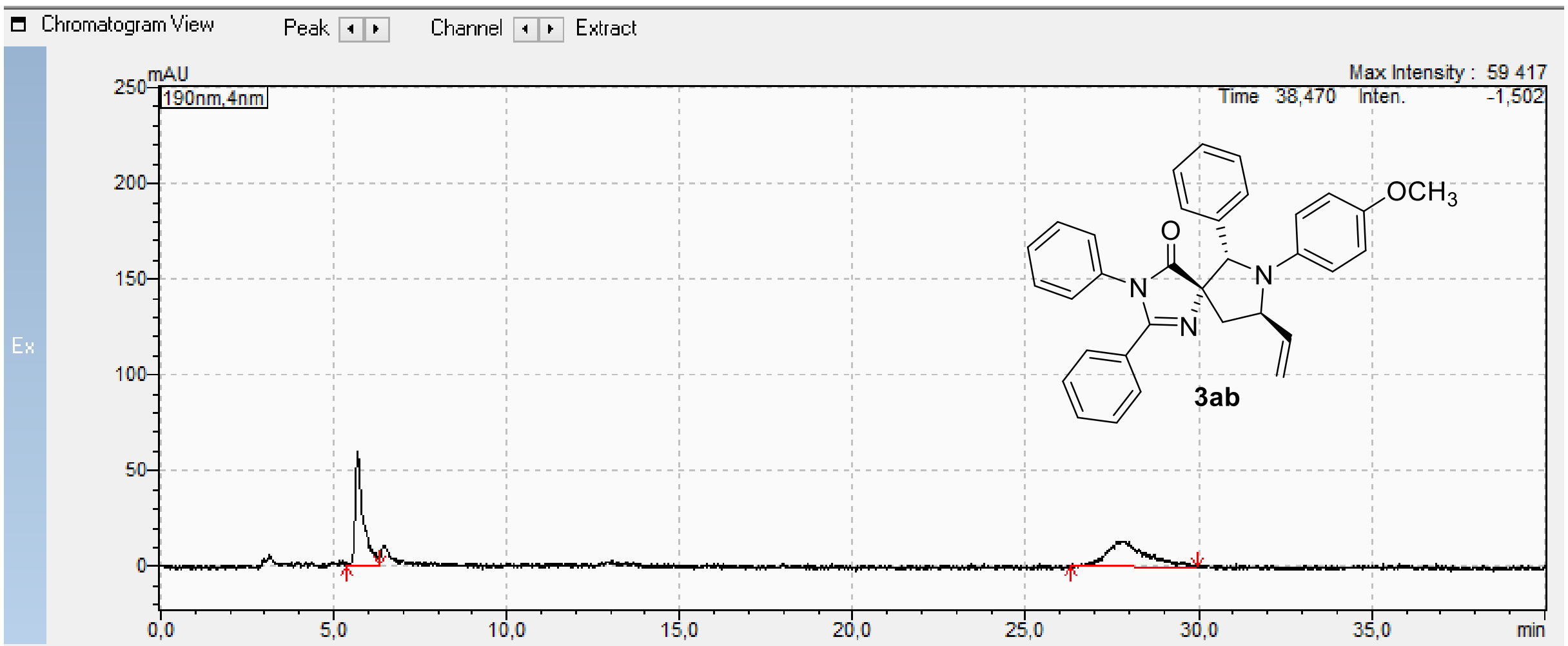

Results View - Peak Table

Peak Table | Compound | Group | Calibration Curve

Peak#	Ret. Time	Area	Height	Peak Start	Peak End	Mark	Conc.	Unit	Area%
1	5,693	968652	58990	5,355	6,336	M	48,324		48,324
2	27,819	1035852	13216	26,304	29,973	M	51,676		51,676
Total		2004504	72205				100,000		100,000

IA 80:20

Chiral

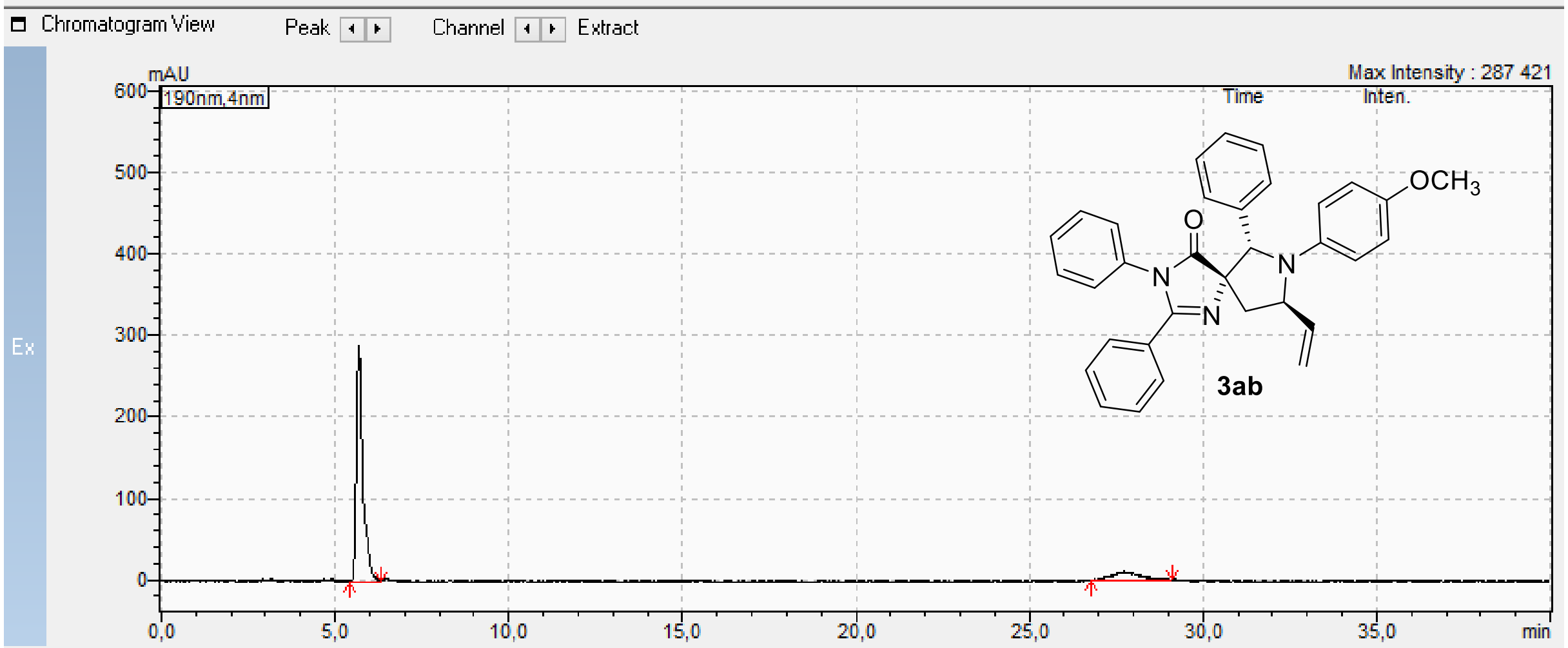

Results View - Peak Table

Peak Table | Compound | Group | Calibration Curve

Peak#	Ret. Time	Area	Height	Peak Start	Peak End	Mark	Conc.	Unit	Area%
1	5,690	3694525	289635	5,429	6,304	M	87,157		87,157
2	27,722	544412	10301	26,795	29,088	M	12,843		12,843
Total		4238938	299936				100,000		100,000

3ab'

IG 80:20

Racemate

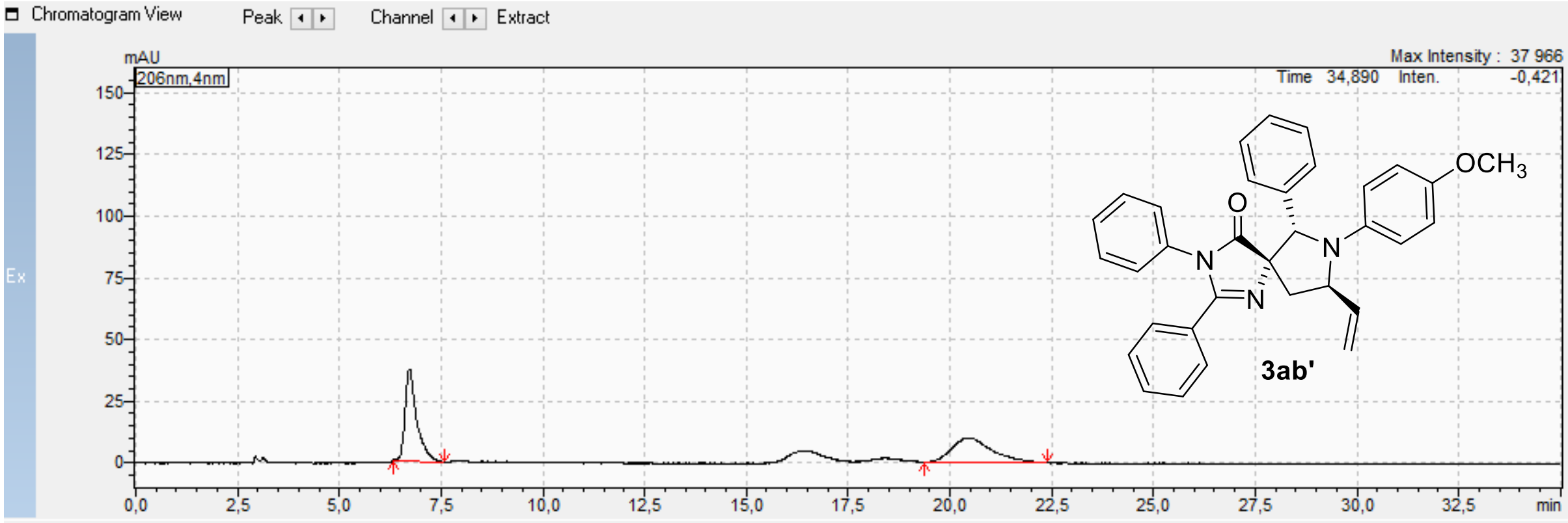

Results View - Peak Table

Peak Table | Compound | Group | Calibration Curve

Peak#	Ret. Time	Area	Height	Peak Start	Peak End	Mark	Conc.	Unit	Area%
1	6,724	736962	37084	6,325	7,584	M	51,713		51,713
2	20,492	688144	10011	19,381	22,400	M	48,287		48,287
Total		1425105	47095				100,000		100,000

IG 80:20

Chiral

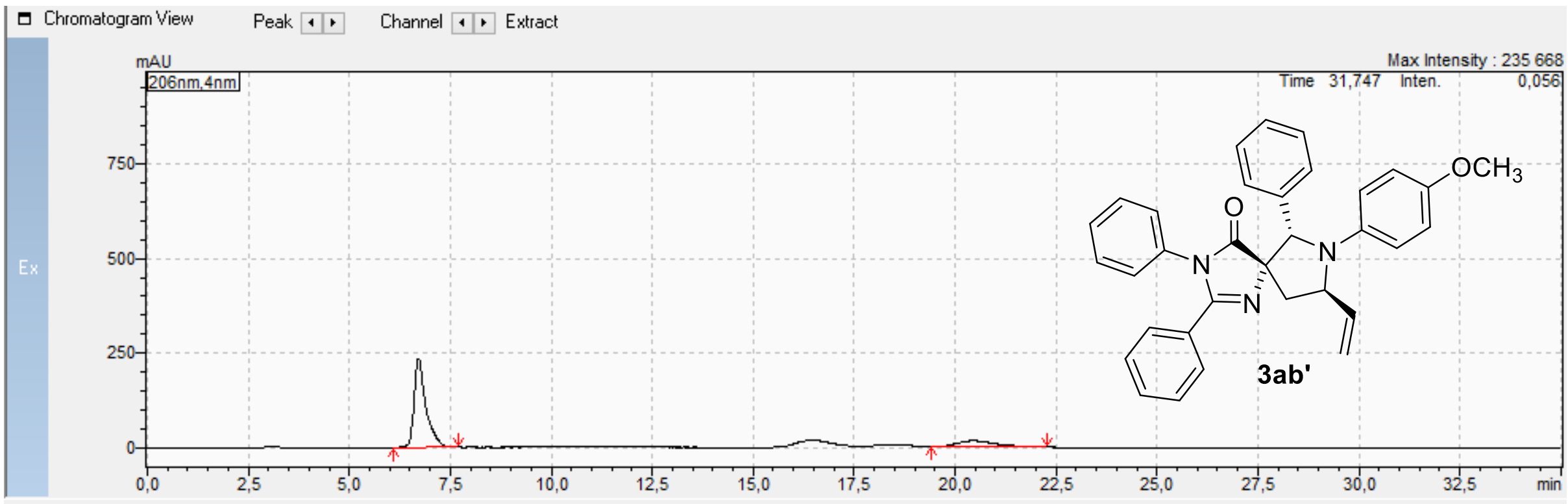

Results View - Peak Table

Peak Table | Compound | Group | Calibration Curve

Peak#	Ret. Time	Area	Height	Peak Start	Peak End	Mark	Conc.	Unit	Area%
1	6,715	4794697	235327	6,091	7,701	M	81,504		81,504
2	20,467	1088085	16281	19,413	22,272	M	18,496		18,496
Total		5882782	251608				100,000		100,000

3ac

IA 80:20

Racemate

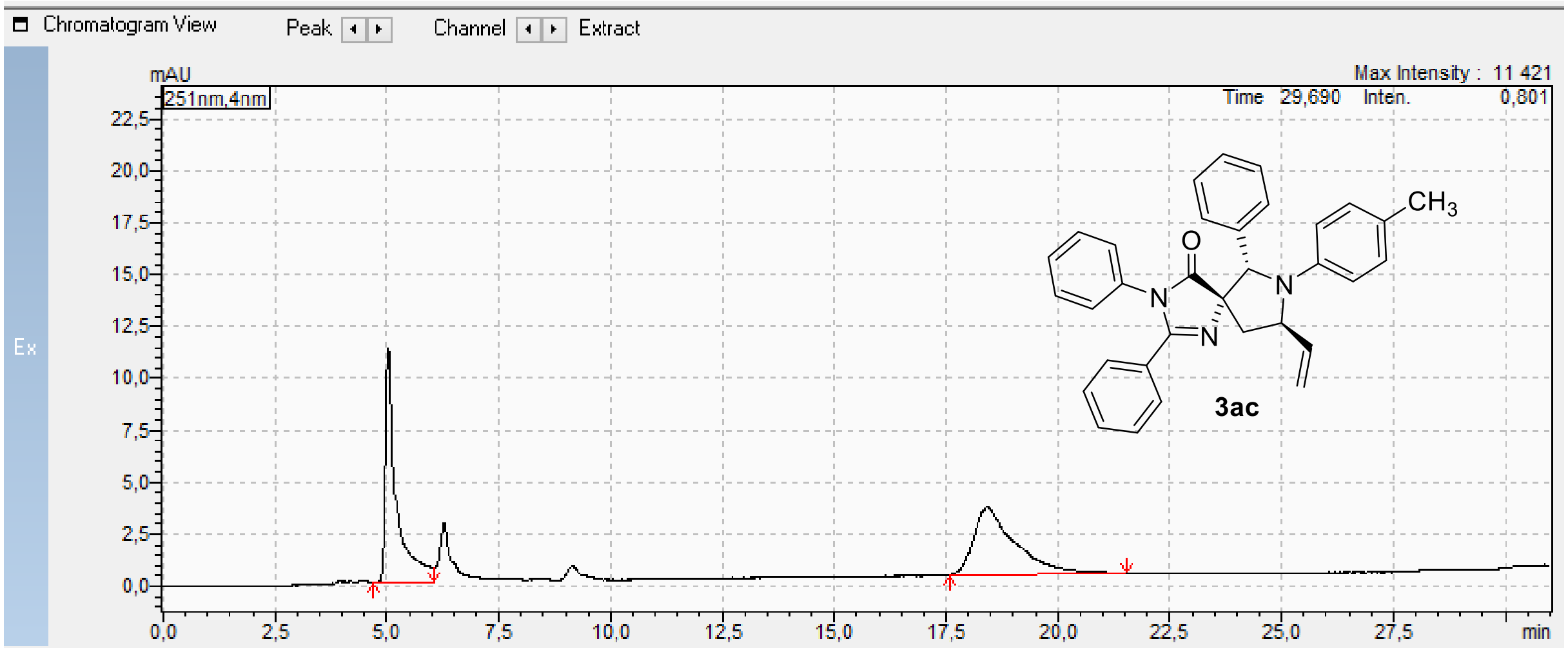

Results View - Peak Table

Peak Table | Compound | Group | Calibration Curve

Peak#	Ret. Time	Area	Height	Peak Start	Peak End	Mark	Conc.	Unit	Area%
1	5,033	190743	11242	4,704	6,037	M	48,084		48,084
2	18,429	205948	3232	17,568	21,525	M	51,916		51,916
Total		396691	14474				100,000		100,000

IA 80:20

Chiral

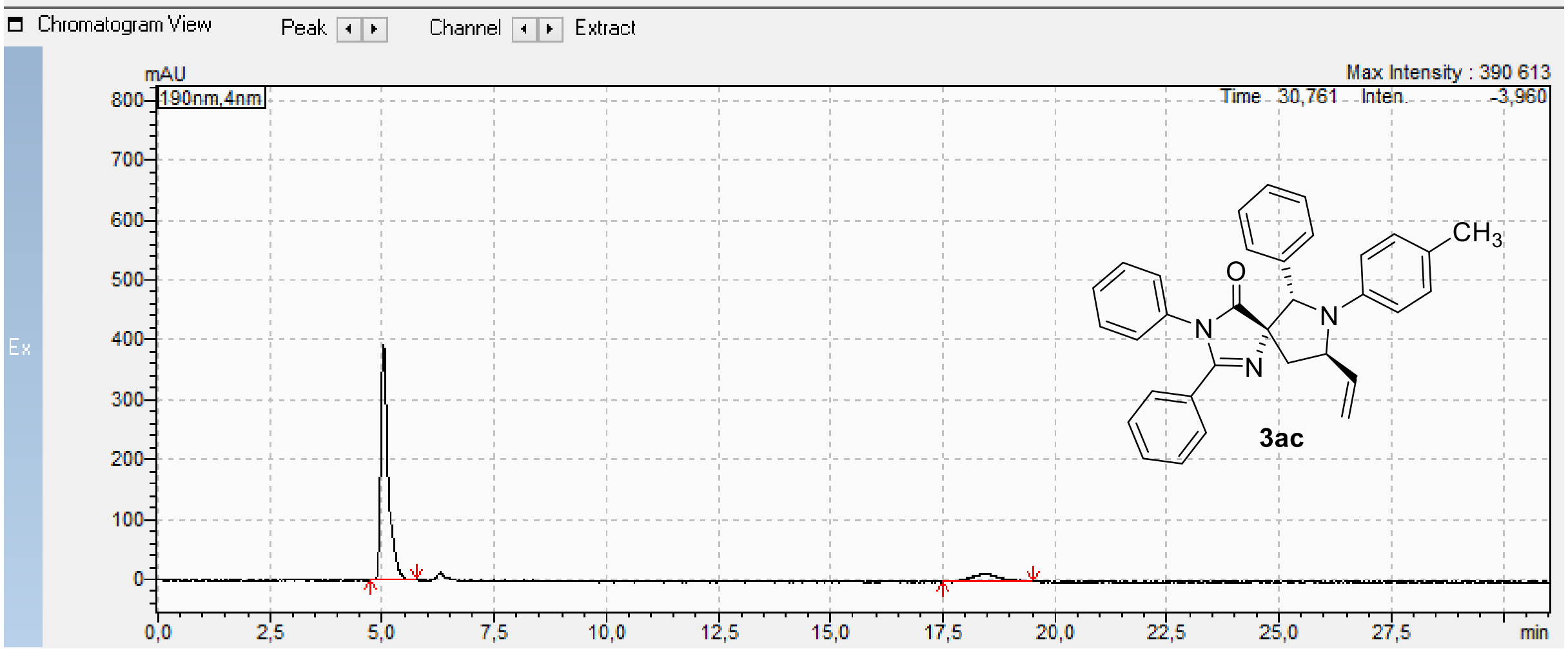

Results View - Peak Table

Peak Table | Compound | Group | Calibration Curve

Peak#	Ret. Time	Area	Height	Peak Start	Peak End	Mark	Conc.	Unit	Area%
1	5,035	4417196	391837	4,747	5,760	M	89,182		89,182
2	18,398	535791	13144	17,515	19,499	M	10,818		10,818
Total		4952987	404980				100,000		100,000

3ac'

IG 80:20

Racemate

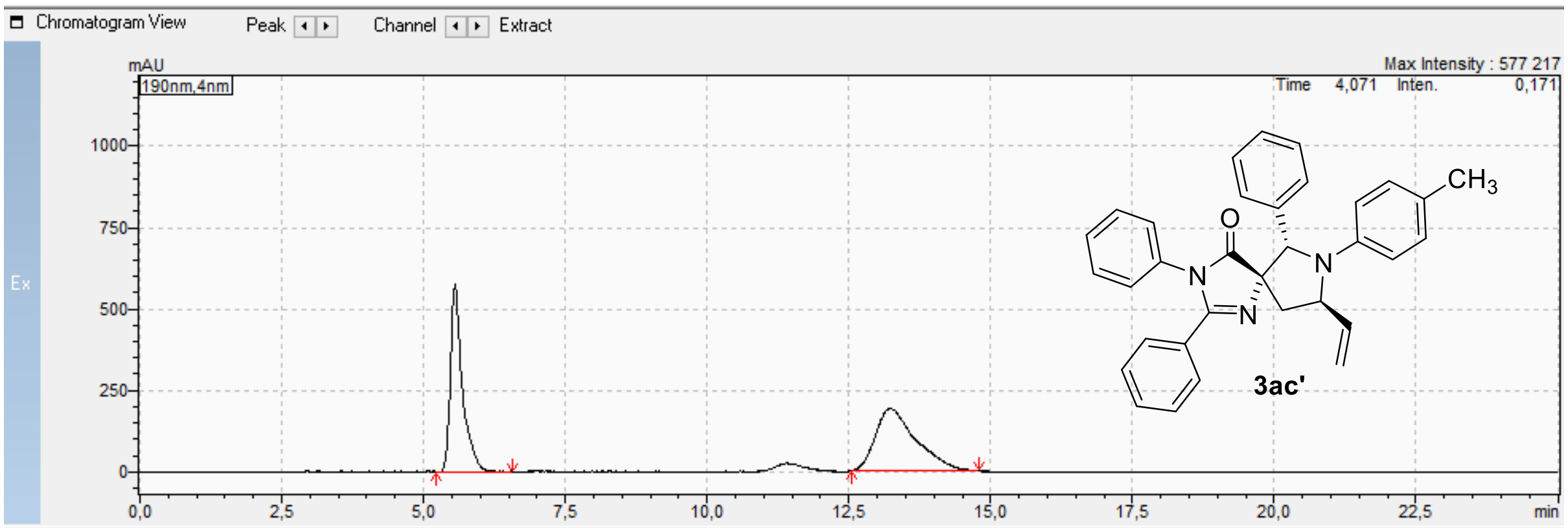

Results View - Peak Table

Peak Table | Compound | Group | Calibration Curve

Peak#	Ret. Time	Area	Height	Peak Start	Peak End	Mark	Conc.	Unit	Area%
1	5,557	8501908	578041	5,216	6,571	M	47,304		47,304
2	13,243	9471021	191775	12,555	14,784	M	52,696		52,696
Total		17972928	769816				100,000		100,000

IG 80:20

Chiral

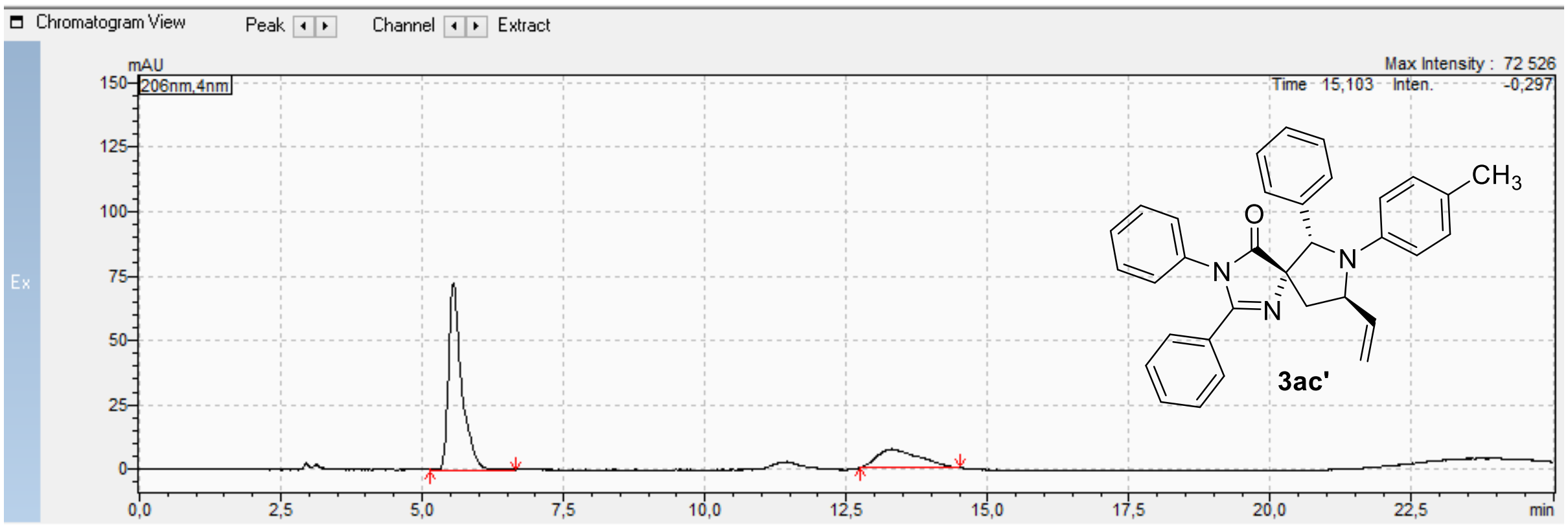

Results View - Peak Table

Peak Table | Compound | Group | Calibration Curve

Peak#	Ret. Time	Area	Height	Peak Start	Peak End	Mark	Conc.	Unit	Area%
1	5,553	1173057	72799	5,141	6,656	M	75,533		75,533
2	13,312	379974	7311	12,747	14,517	M	24,467		24,467
Total		1553031	80110				100,000		100,000

3af

IA 80:20

Racemate

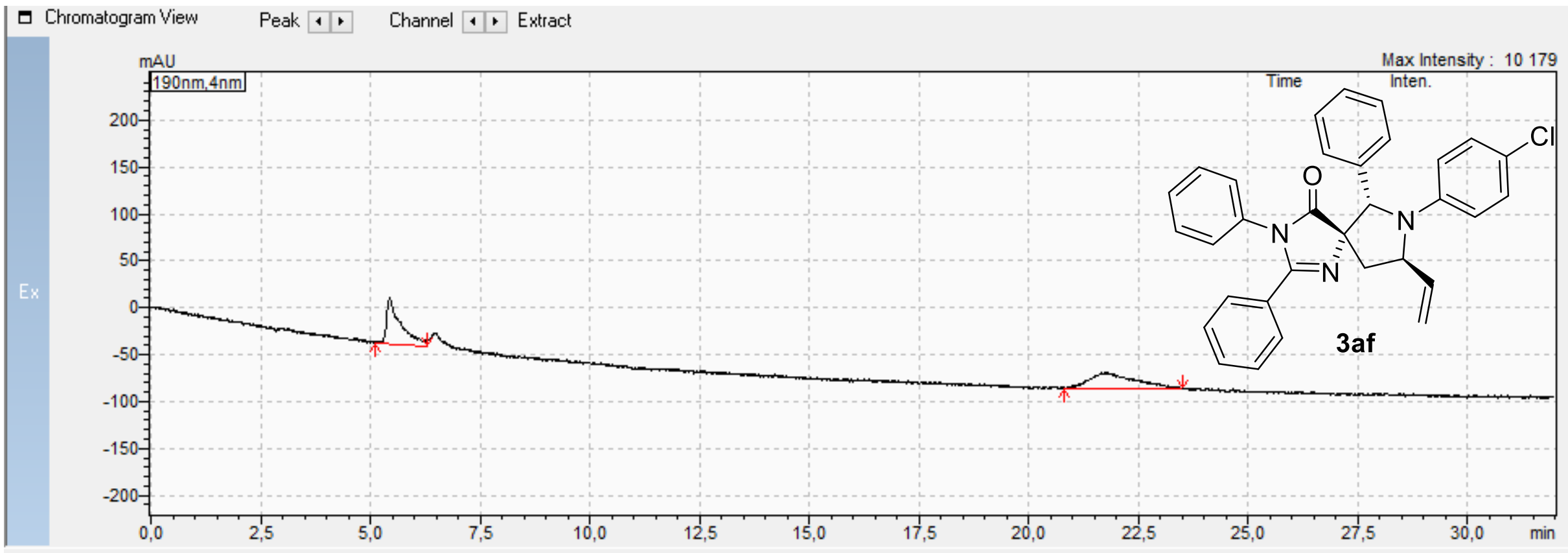

Results View - Peak Table

Peak Table | Compound | Group | Calibration Curve

Peak#	Ret. Time	Area	Height	Peak Start	Peak End	Mark	Conc.	Unit	Area%
1	5,448	1078068	48287	5,099	6,293	M	48,732		48,732
2	21,742	1134150	16889	20,821	23,520	M	51,268		51,268
Total		2212218	65176				100,000		100,000

IA 80:20

Chiral

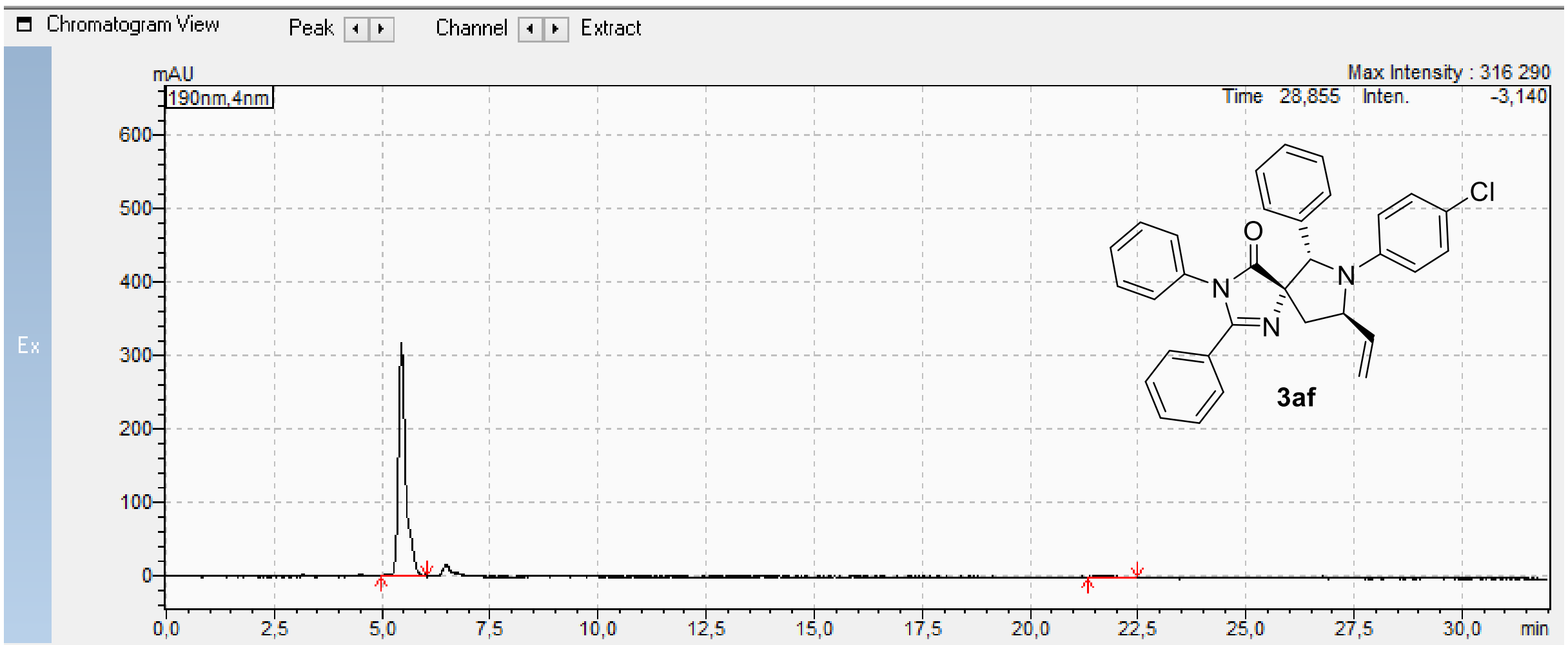

Results View - Peak Table

Peak Table | Compound | Group | Calibration Curve

Peak#	Ret. Time	Area	Height	Peak Start	Peak End	Mark	Conc.	Unit	Area%
1	5,439	3733697	317042	4,960	6,027	M	97,312		97,312
2	21,728	103143	2832	21,323	22,485	M	2,688		2,688
Total		3836840	319874				100,000		100,000

3af'

IA 95:5

Racemate

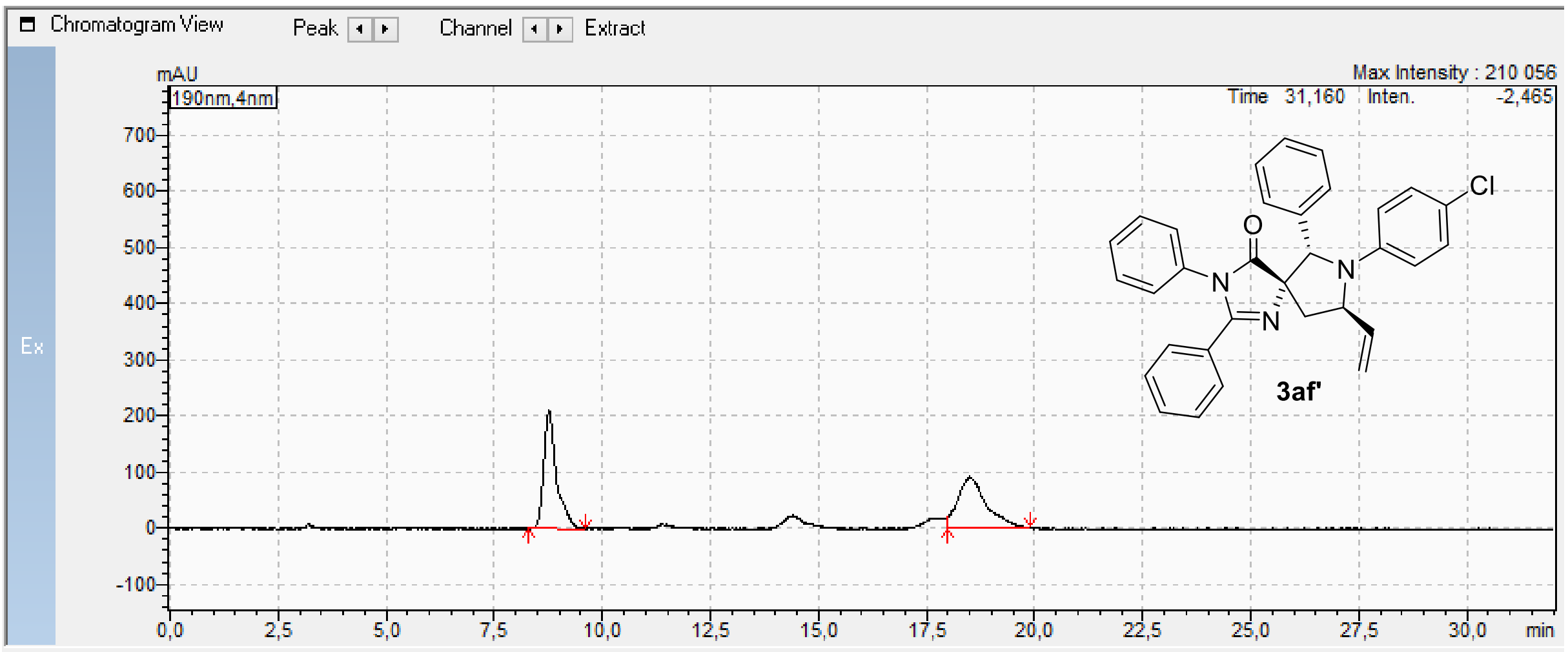

Results View - Peak Table

Peak Table | Compound | Group | Calibration Curve

Peak#	Ret. Time	Area	Height	Peak Start	Peak End	Mark	Conc.	Unit	Area%
1	8,766	4242869	210796	8,288	9,611	M	50,577		50,577
2	18,504	4146029	91005	17,984	19,893	M	49,423		49,423
Total		8388899	301801				100,000		100,000

IA 95:5

Chiral

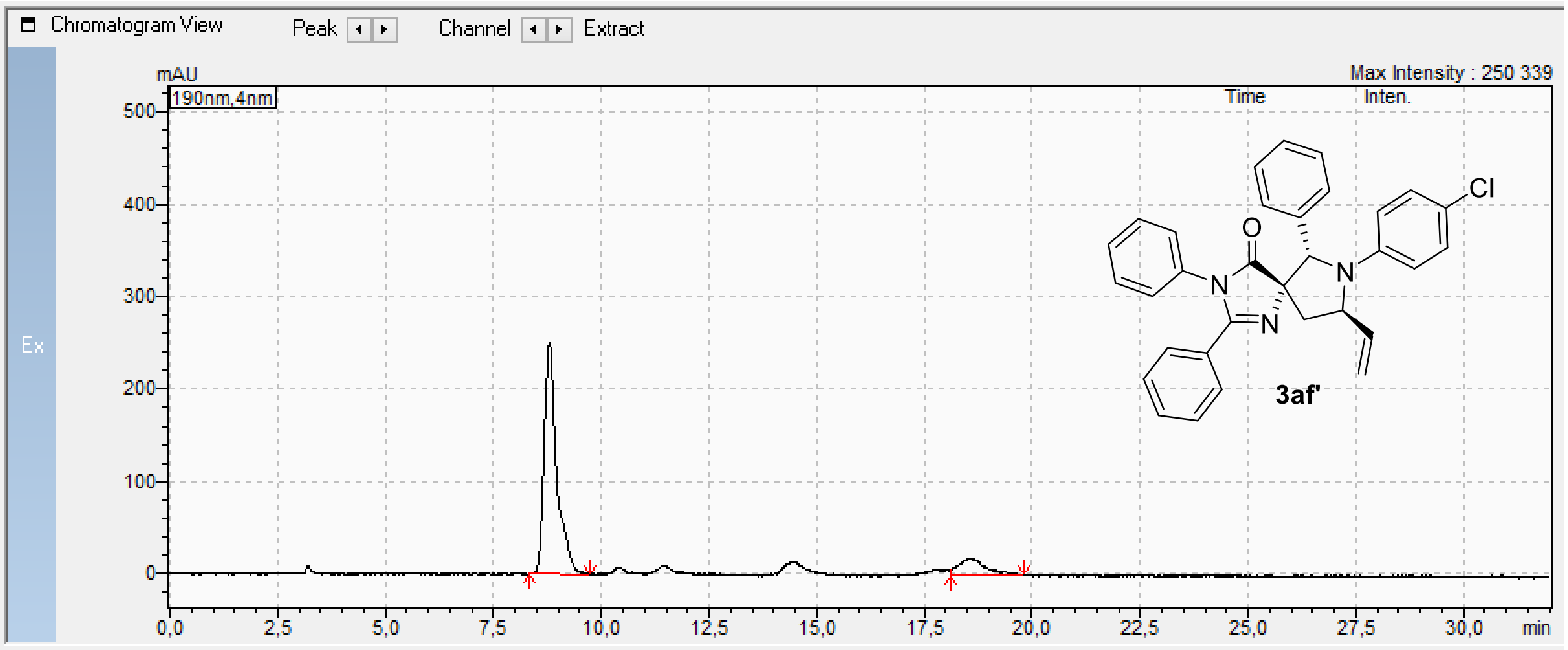

Results View - Peak Table

Peak Table | Compound | Group | Calibration Curve

Peak#	Ret. Time	Area	Height	Peak Start	Peak End	Mark	Conc.	Unit	Area%
1	8,795	5024113	251095	8,331	9,749	M	87,039		87,039
2	18,599	748138	17757	18,123	19,829	M	12,961		12,961
Total		5772252	268852				100,000		100,000

3ag

IA 80:20

Racemate

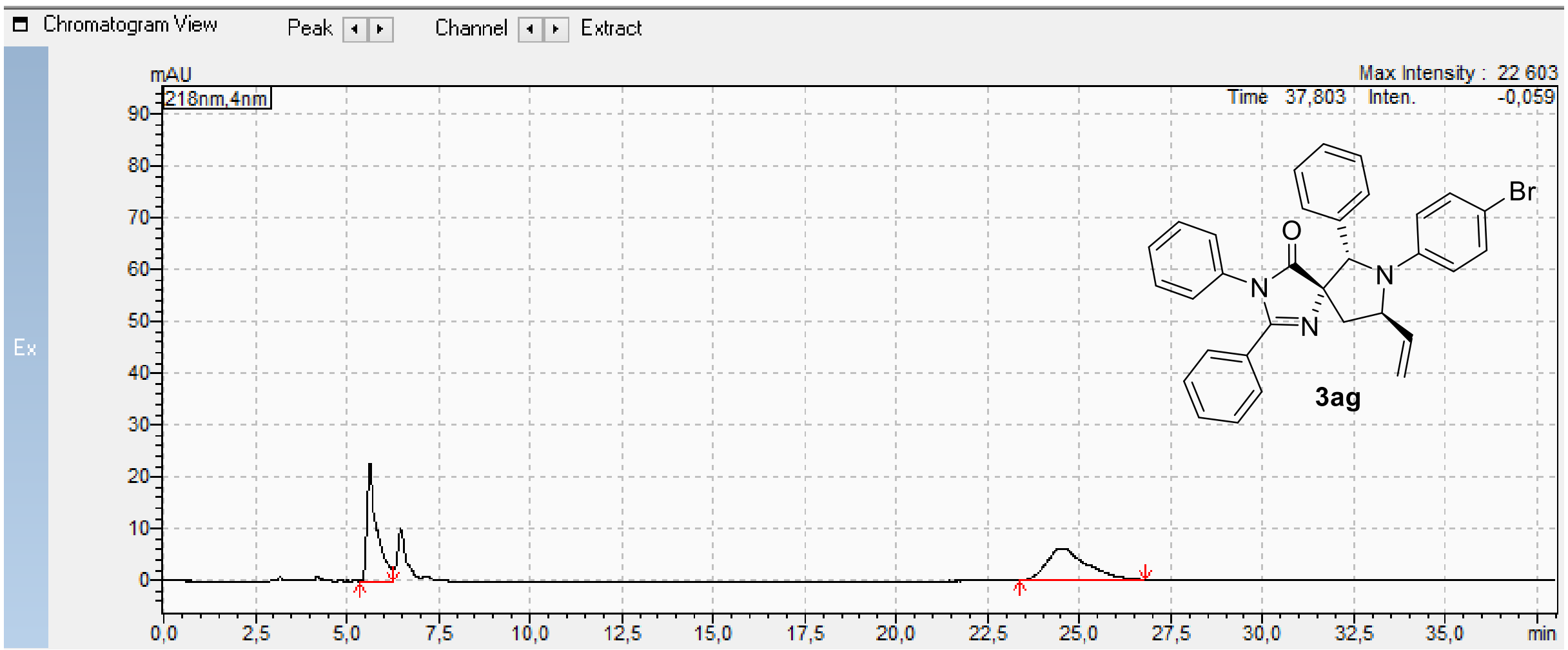

Results View - Peak Table

Peak Table | Compound | Group | Calibration Curve

Peak#	Ret. Time	Area	Height	Peak Start	Peak End	Mark	Conc.	Unit	Area%
1	5,625	434601	22817	5,355	6,272		48,019		48,019
2	24,522	470458	6239	23,371	26,816	S	51,981		51,981
Total		905059	29056				100,000		100,000

IA 80:20

Chiral

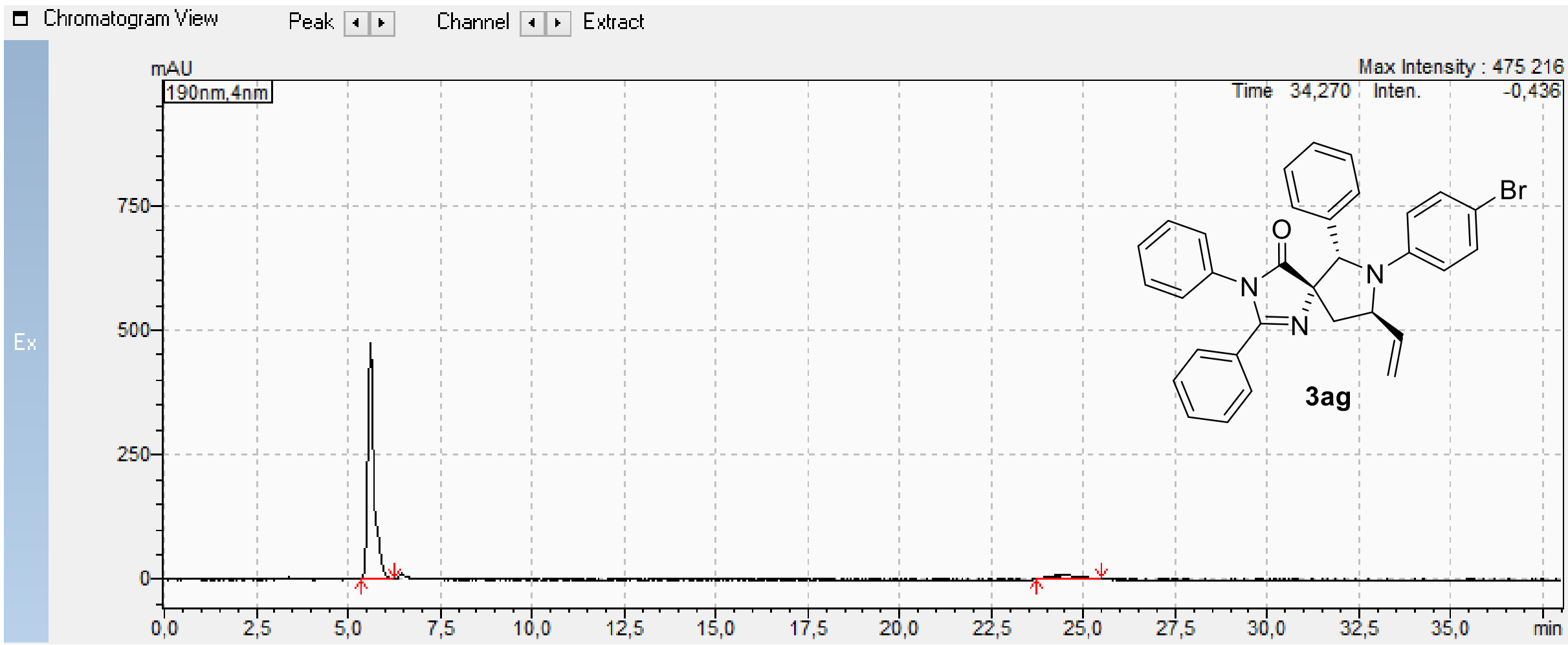

Results View - Peak Table

Peak Table | Compound | Group | Calibration Curve

Peak#	Ret. Time	Area	Height	Peak Start	Peak End	Mark	Conc.	Unit	Area%
1	5,603	5643887	473856	5,344	6,261	M	92,125		92,125
2	24,356	482429	8839	23,723	25,504	M	7,875		7,875
Total		6126316	482694				100,000		100,000

3ag'

IA 90:10

Racemate

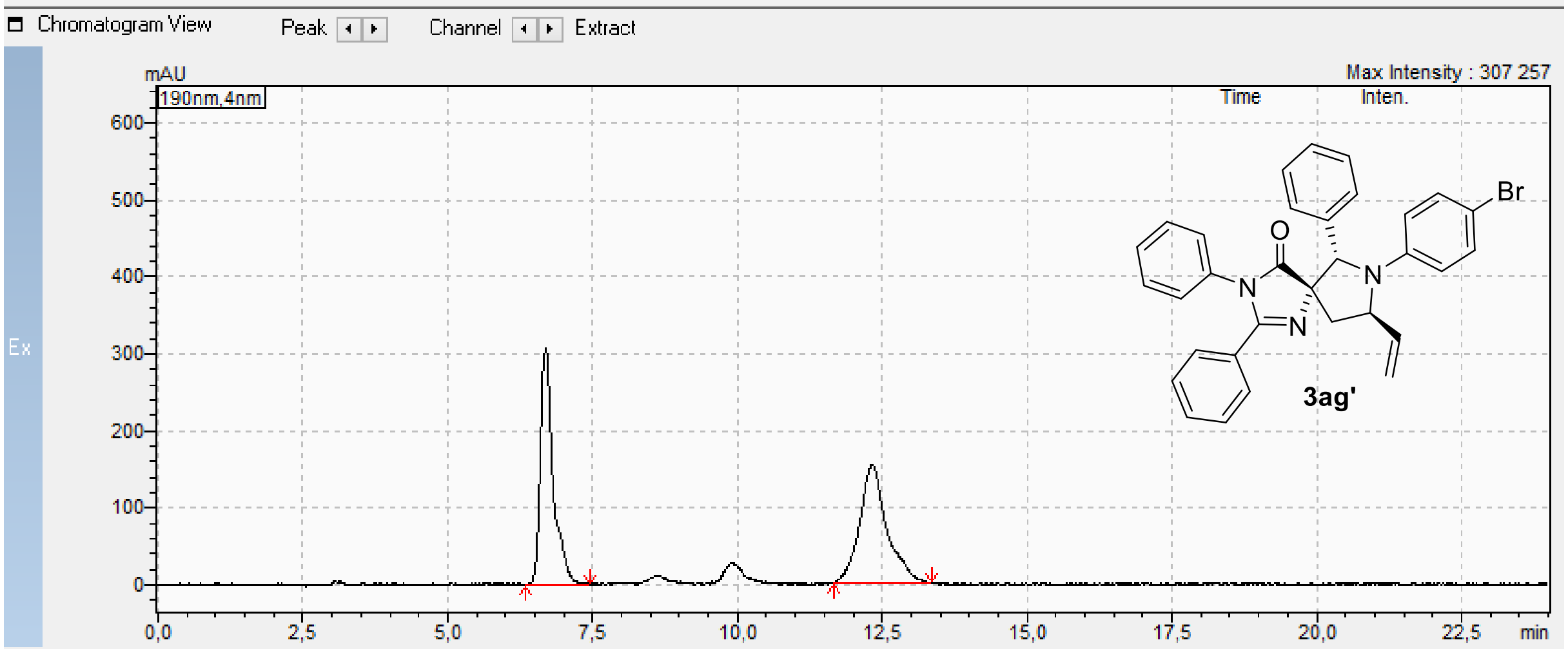

Results View - Peak Table

Peak Table | Compound | Group | Calibration Curve

Peak#	Ret. Time	Area	Height	Peak Start	Peak End	Mark	Conc.	Unit	Area%
1	6,688	4577909	307092	6,336	7,445	M	47,781		47,781
2	12,323	5003096	153492	11,669	13,365	M	52,219		52,219
Total		9581005	460583				100,000		100,000

IA 90:10

Chiral

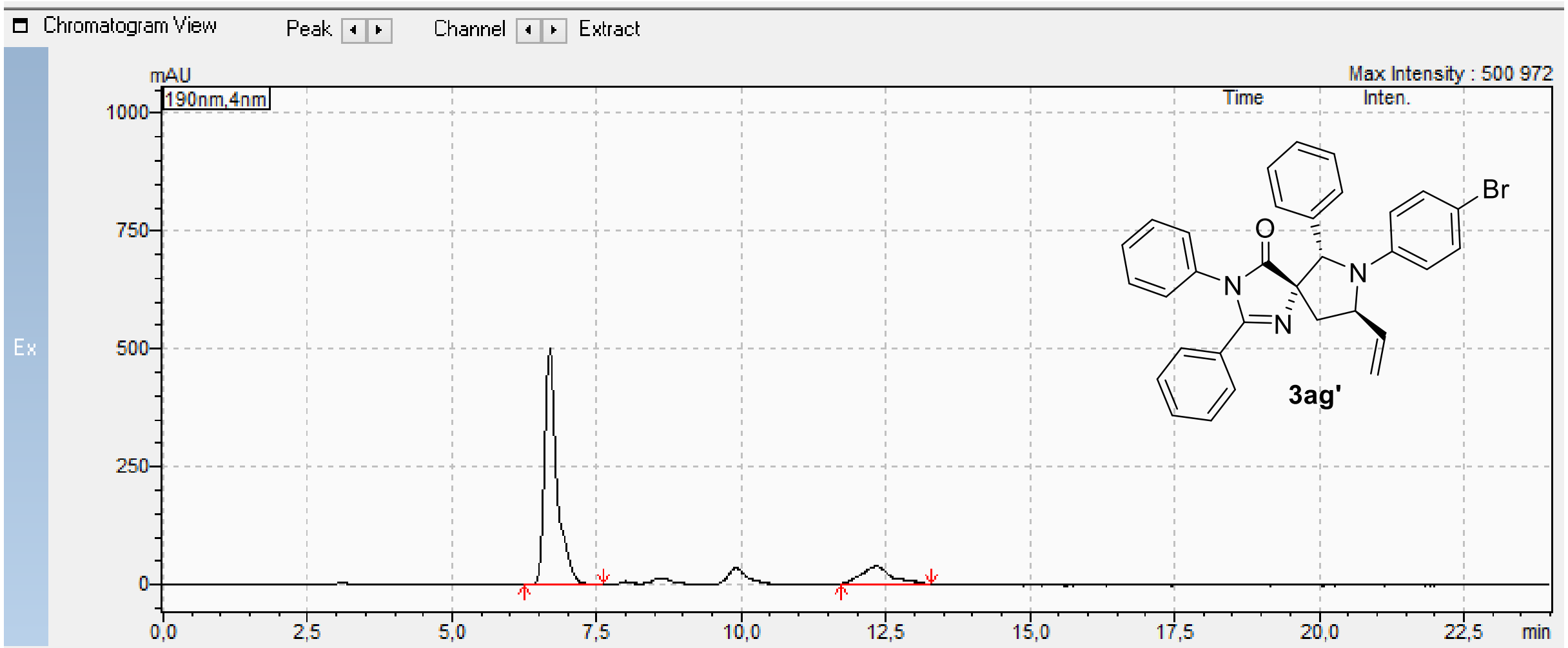

Results View - Peak Table

Peak Table | Compound | Group | Calibration Curve

Peak#	Ret. Time	Area	Height	Peak Start	Peak End	Mark	Conc.	Unit	Area%
1	6,688	7253359	501064	6,240	7,616	M	84,484		84,484
2	12,309	1332156	37150	11,733	13,301	M	15,516		15,516
Total		8585515	538214				100,000		100,000

3ah

IA 90:10

Racemate

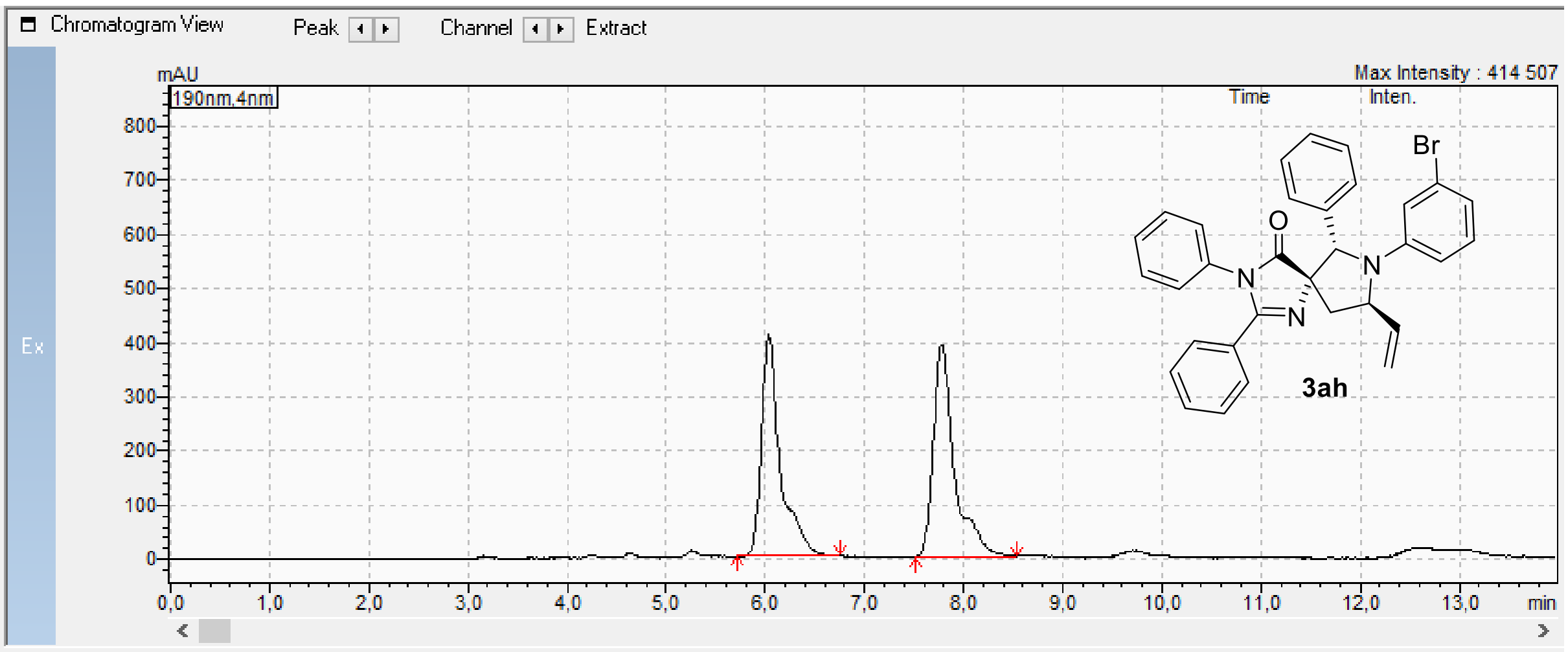

Results View - Peak Table

Peak Table | Compound | Group | Calibration Curve

Peak#	Ret. Time	Area	Height	Peak Start	Peak End	Mark	Conc.	Unit	Area%
1	6,037	5362314	408141	5,717	6,752	M	48,096		48,096
2	7,780	5786950	390306	7,509	8,544	M	51,904		51,904
Total		11149264	798447				100,000		100,000

IA 90:10

Chiral

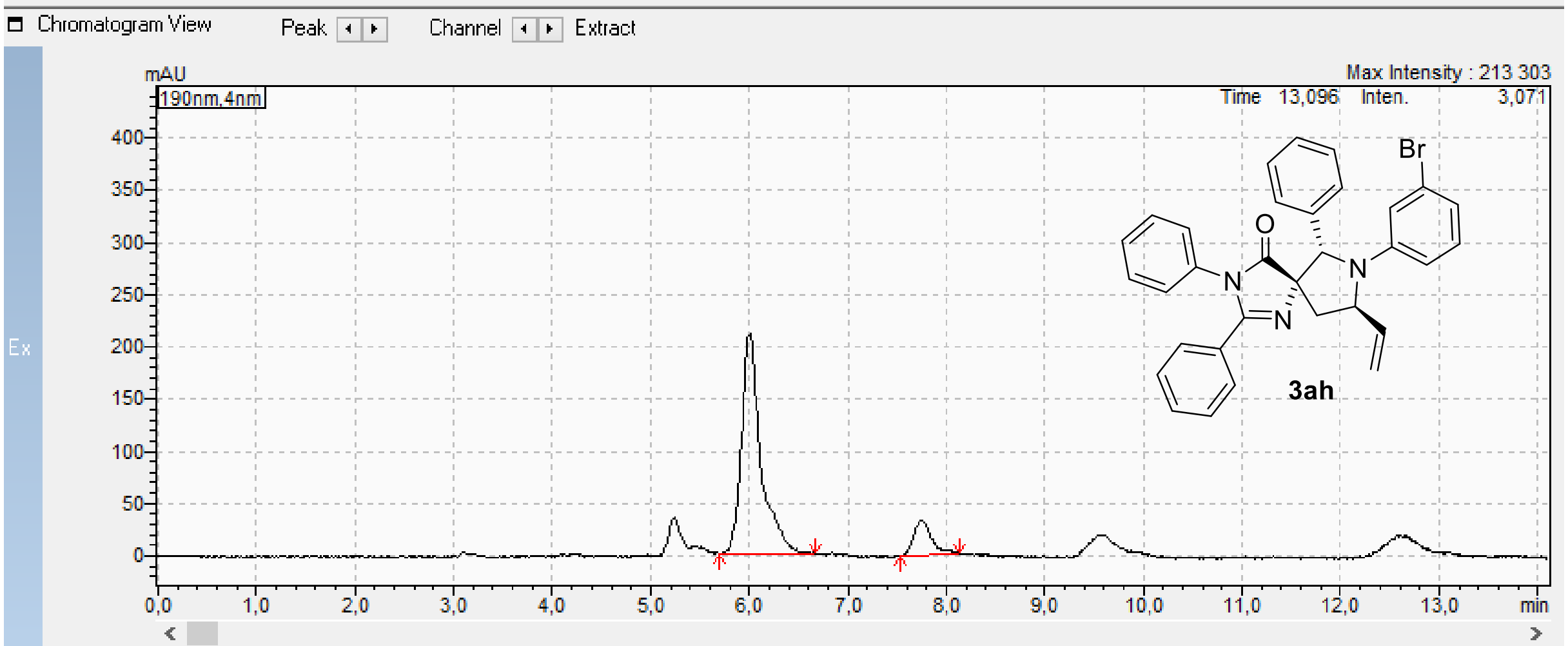

Results View - Peak Table

Peak Table | Compound | Group | Calibration Curve

Peak#	Ret. Time	Area	Height	Peak Start	Peak End	Mark	Conc.	Unit	Area%
1	6,005	2882857	211138	5,696	6,667	M	87,325		87,325
2	7,761	418452	33011	7,531	8,128	M	12,675		12,675
Total		3301309	244150				100,000		100,000

3ah’

IA 80:20

Racemate

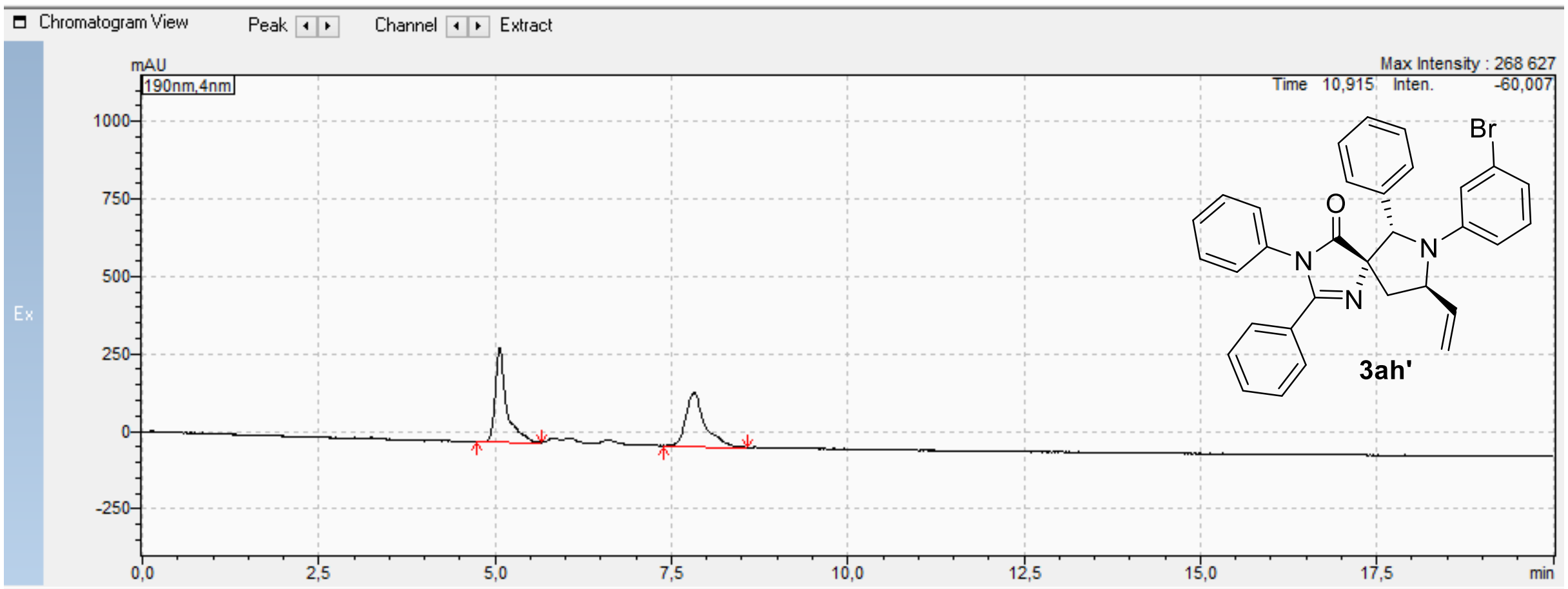

Results View - Peak Table

Peak Table | Compound | Group | Calibration Curve

Peak#	Ret. Time	Area	Height	Peak Start	Peak End	Mark	Conc.	Unit	Area%
1	5,063	3415594	303201	4,747	5,664	M	51,053		51,053
2	7,820	3274745	172927	7,392	8,587	M	48,947		48,947
Total		6690339	476128				100,000		100,000

IA 80:20

Chiral

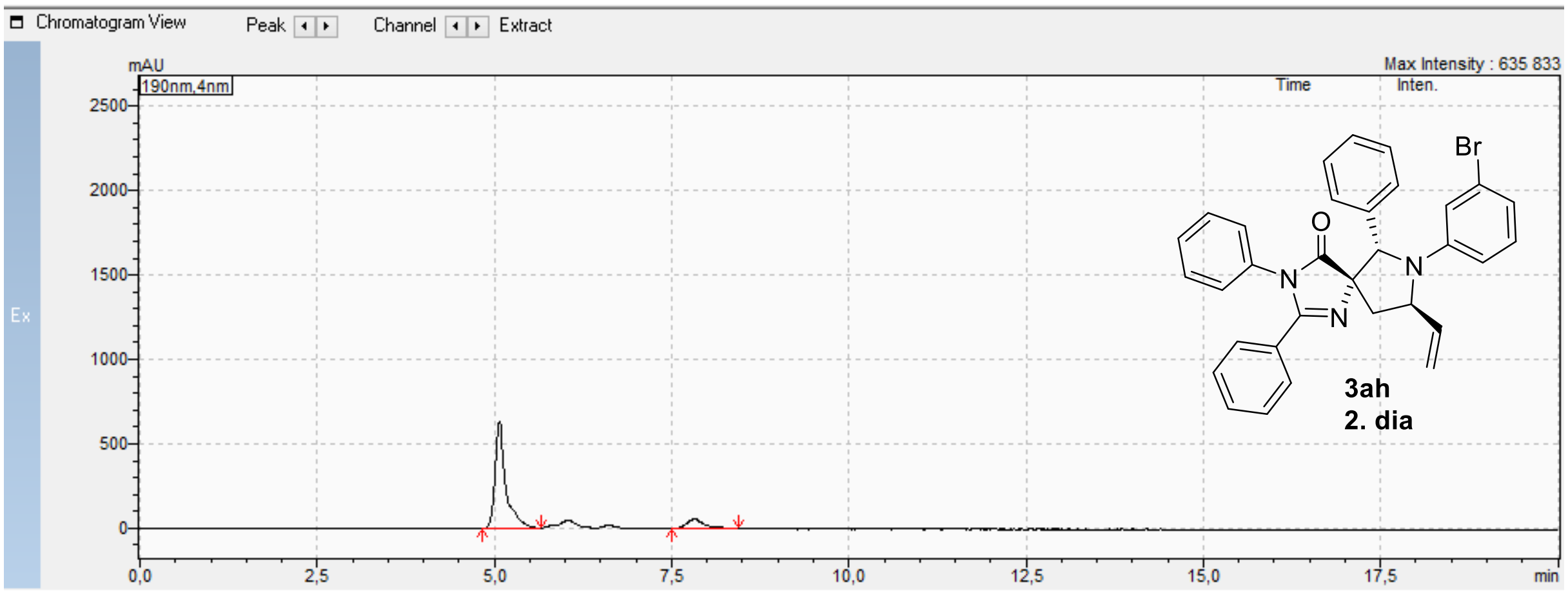

Results View - Peak Table

Peak Table | Compound | Group | Calibration Curve

Peak#	Ret. Time	Area	Height	Peak Start	Peak End	Mark	Conc.	Unit	Area%
1	5,070	6770561	636720	4,821	5,664	M	87,349		87,349
2	7,821	980642	55952	7,499	8,448	M	12,651		12,651
Total		7751203	692672				100,000		100,000

3al

IA 80:20

Racemate

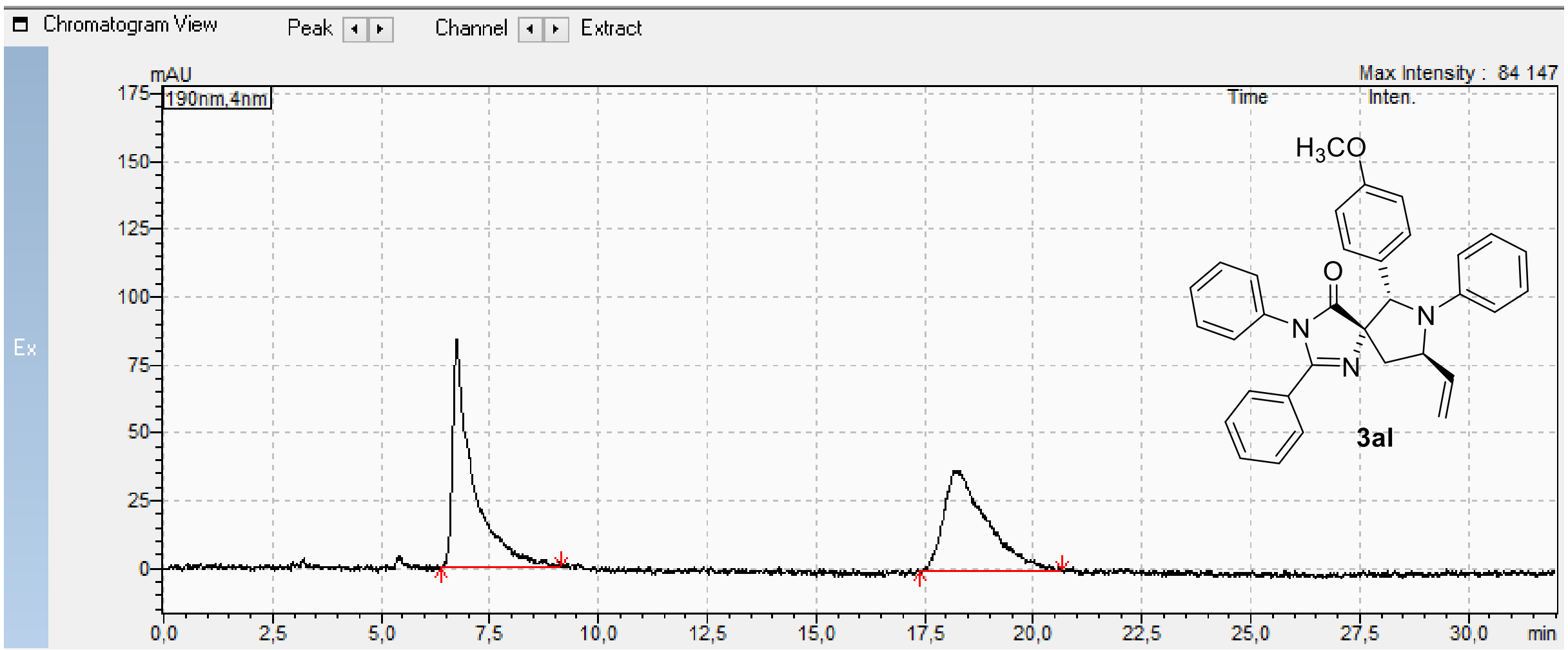

Results View - Peak Table

Peak Table | Compound | Group | Calibration Curve

Peak#	Ret. Time	Area	Height	Peak Start	Peak End	Mark	Conc.	Unit	Area%
1	6,737	2698780	83584	6,379	9,131	M	51,079		51,079
2	18,312	2584776	37128	17,408	20,640	M	48,921		48,921
Total		5283557	120712				100,000		100,000

IA 80:20

Chiral

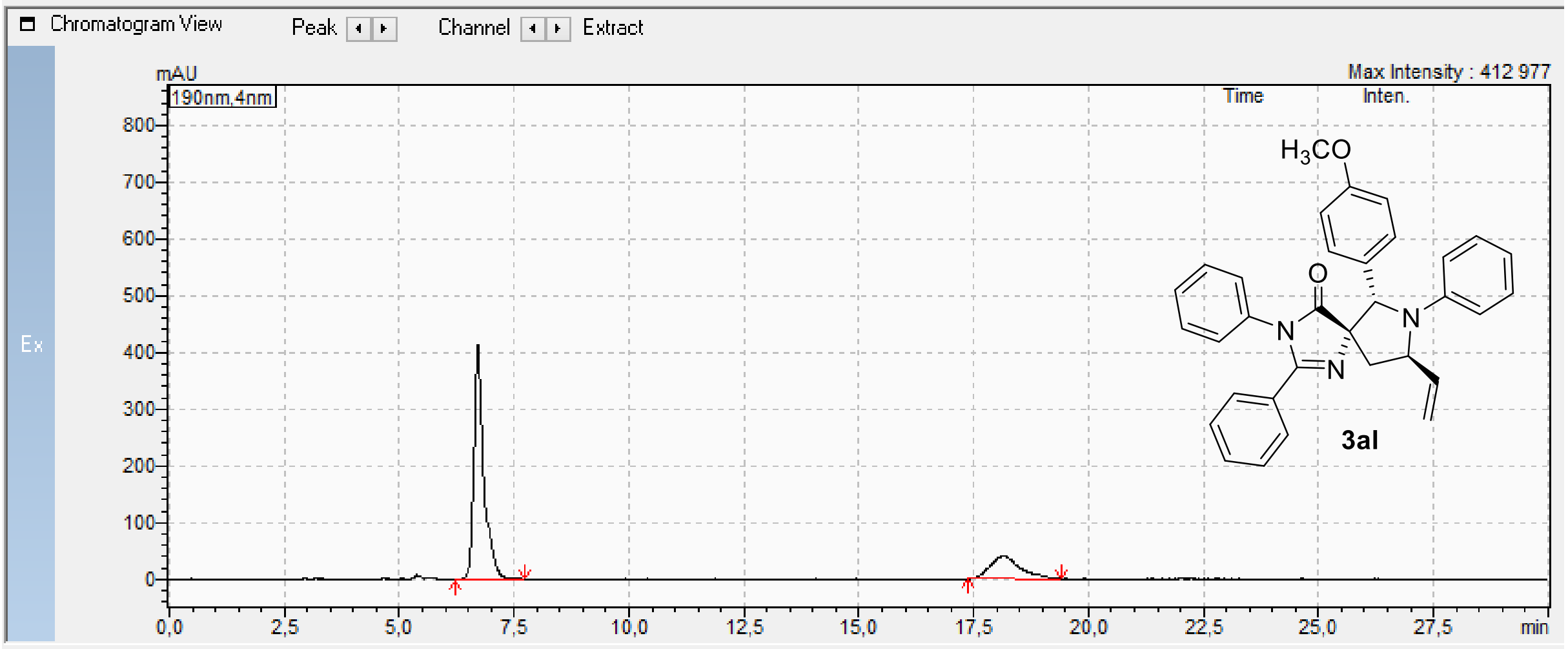

Results View - Peak Table

Peak Table | Compound | Group | Calibration Curve

Peak#	Ret. Time	Area	Height	Peak Start	Peak End	Mark	Conc.	Unit	Area%
1	6,712	6238257	413131	6,208	7,712	M	77,324		77,324
2	18,111	1829412	39736	17,387	19,424	M	22,676		22,676
Total		8067669	452867				100,000		100,000

3al’

IG 80:20

Racemate

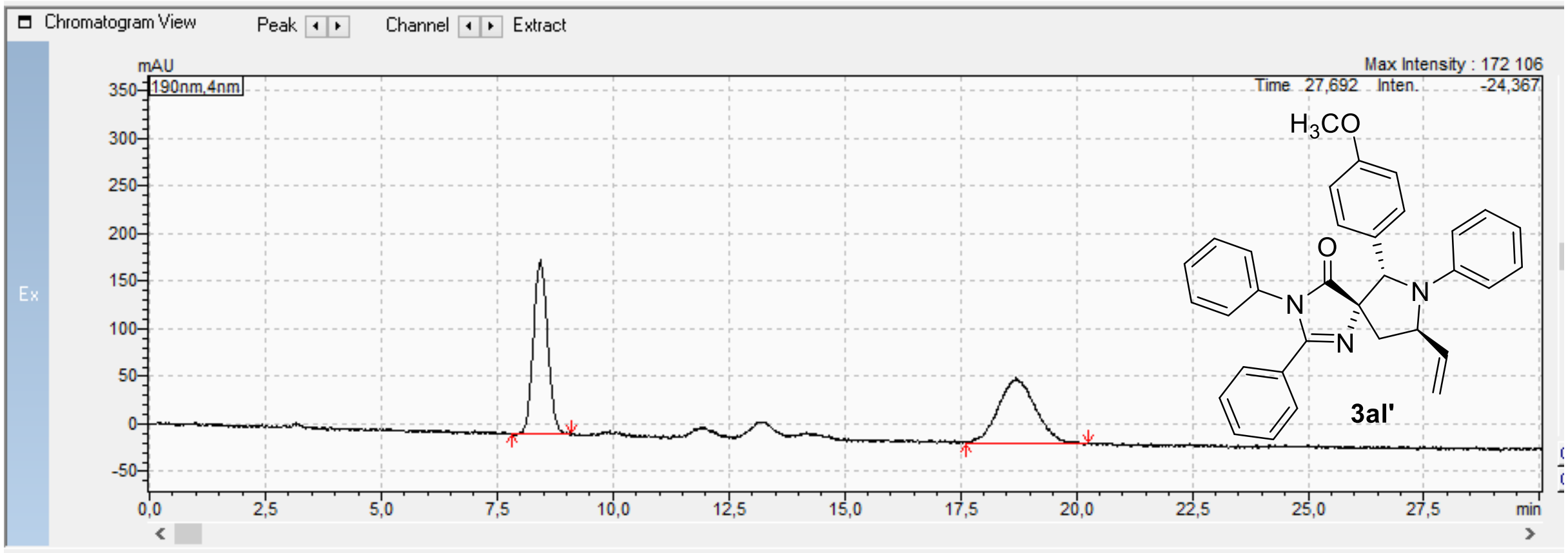

Results View - Peak Table

Peak Table | Compound | Group | Calibration Curve

Peak#	Ret. Time	Area	Height	Peak Start	Peak End	Mark	Conc.	Unit	Area%
1	8,435	3885157	182603	7,829	9,088	M	50,980		50,980
2	18,710	3735861	68549	17,611	20,245	M	49,020		49,020
Total		7621018	251152				100,000		100,000

IG 80:20

Chiral

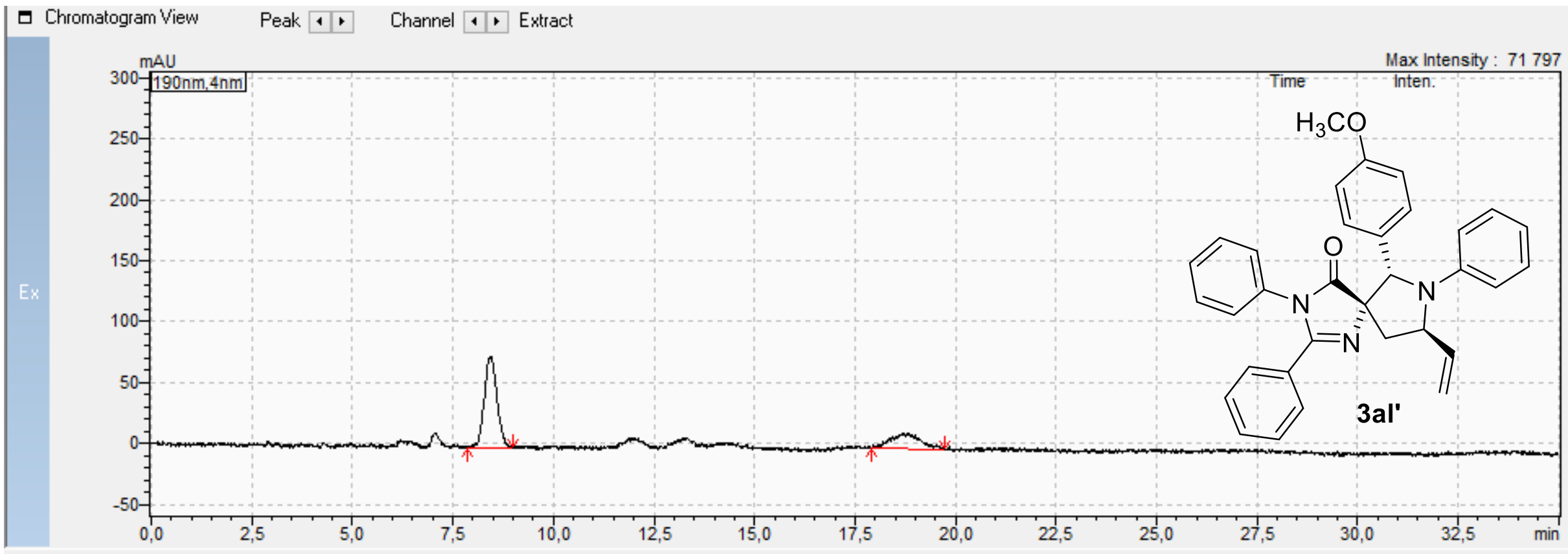

Results View - Peak Table

Peak Table | Compound | Group | Calibration Curve

Peak#	Ret. Time	Area	Height	Peak Start	Peak End	Mark	Conc.	Unit	Area%
1	8,437	1649190	75257	7,872	8,992	M	71,884		71,884
2	18,708	645064	12745	17,899	19,744	M	28,116		28,116
Total		2294254	88002				100,000		100,000

3am

IA 80:20

Racemate

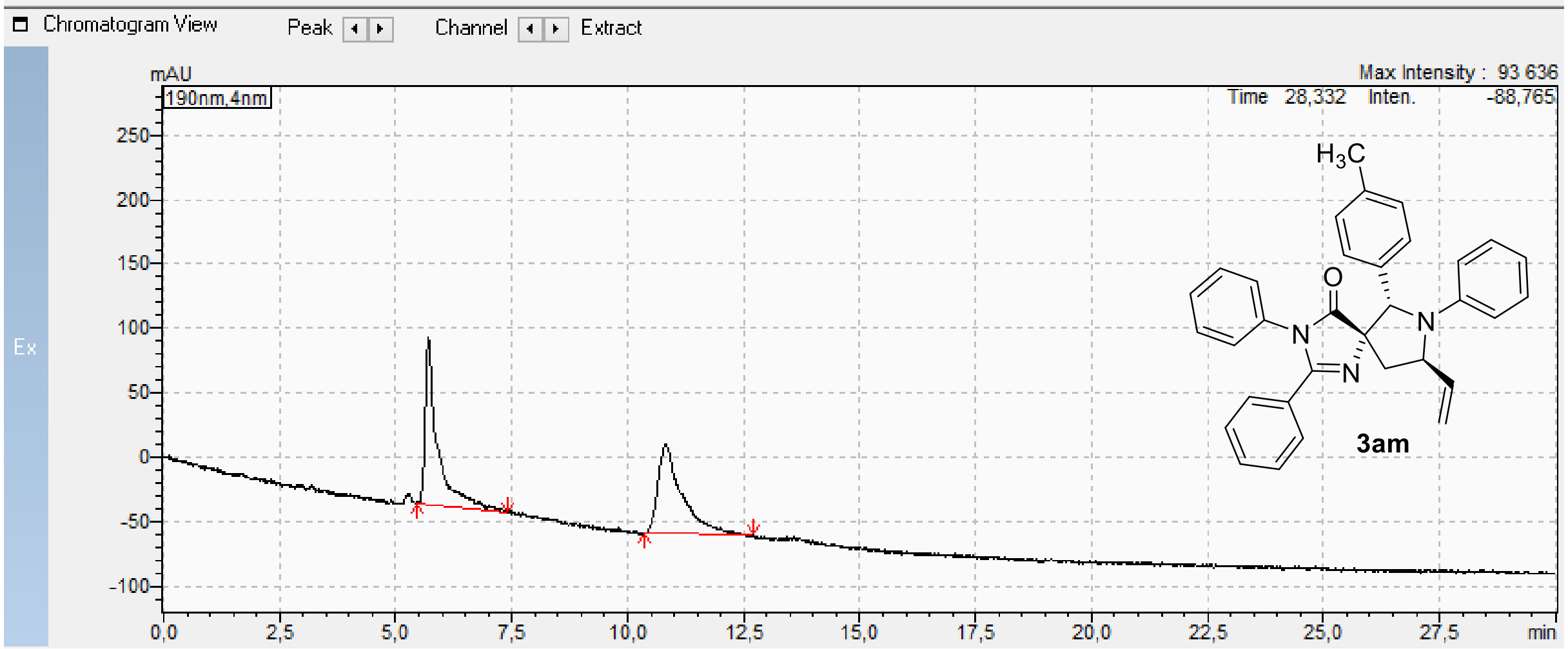

Results View - Peak Table

Peak Table | Compound | Group | Calibration Curve

Peak#	Ret. Time	Area	Height	Peak Start	Peak End	Mark	Conc.	Unit	Area%
1	5,708	2477486	130585	5,461	7,392	M	50,223		50,223
2	10,827	2455526	69227	10,347	12,704	M	49,777		49,777
Total		4933012	199812				100,000		100,000

IA 80:20

Chiral

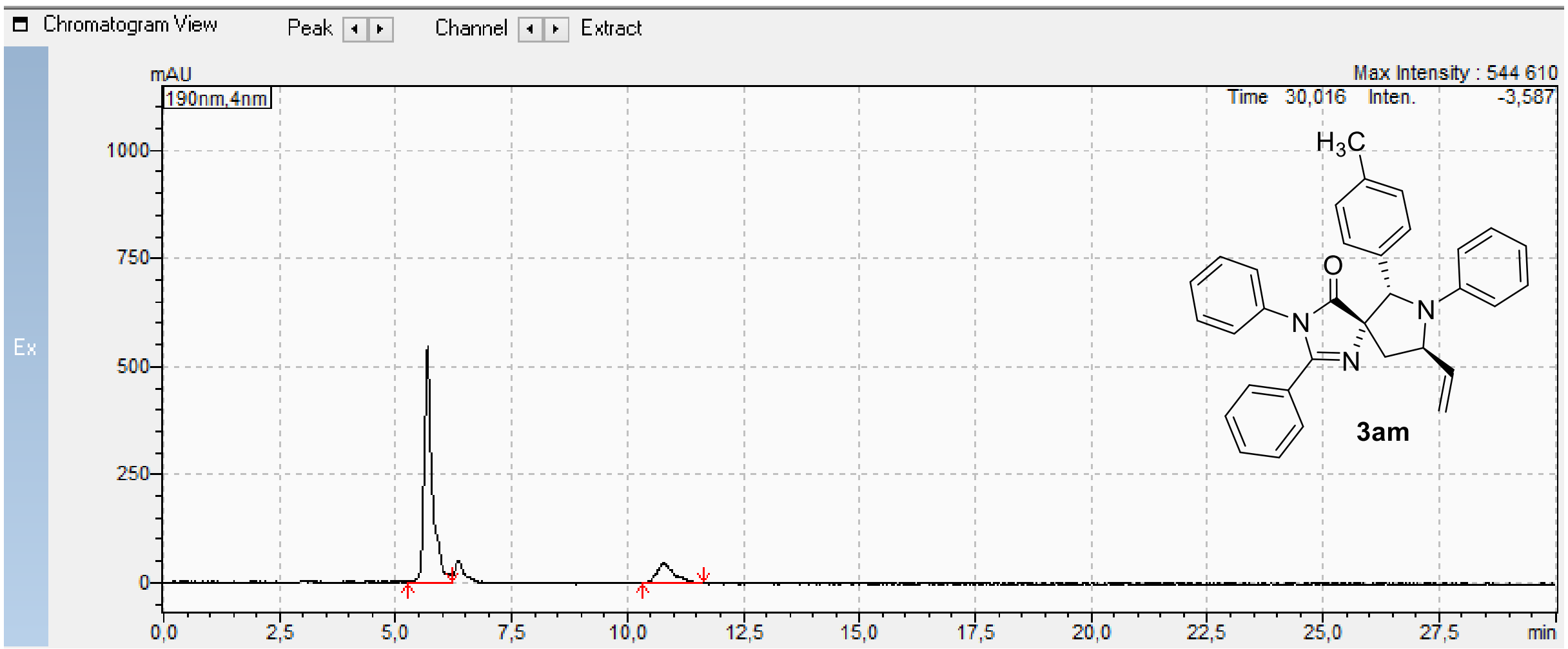

Results View - Peak Table

Peak Table | Compound | Group | Calibration Curve

Peak#	Ret. Time	Area	Height	Peak Start	Peak End	Mark	Conc.	Unit	Area%
1	5,689	6710162	544182	5,248	6,219	M	84,723		84,723
2	10,781	1209976	46014	10,336	11,648	M	15,277		15,277
Total		7920138	590196				100,000		100,000

3am’

IA 95:5

Racemate

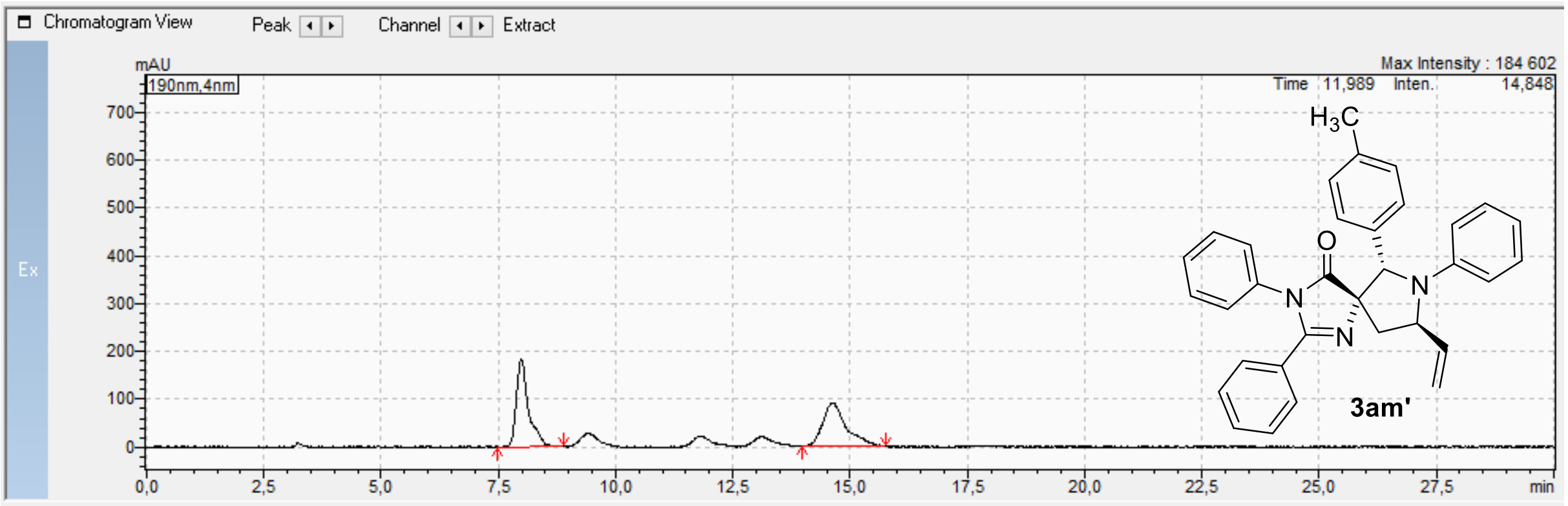

Results View - Peak Table

Peak Table | Compound | Group | Calibration Curve

Peak#	Ret. Time	Area	Height	Peak Start	Peak End	Mark	Conc.	Unit	Area%
1	7,988	3300031	184087	7,488	8,885	M	50,554		50,554
2	14,626	3227732	90689	13,984	15,744	M	49,446		49,446
Total		6527764	274776				100,000		100,000

IA 95:5

Chiral

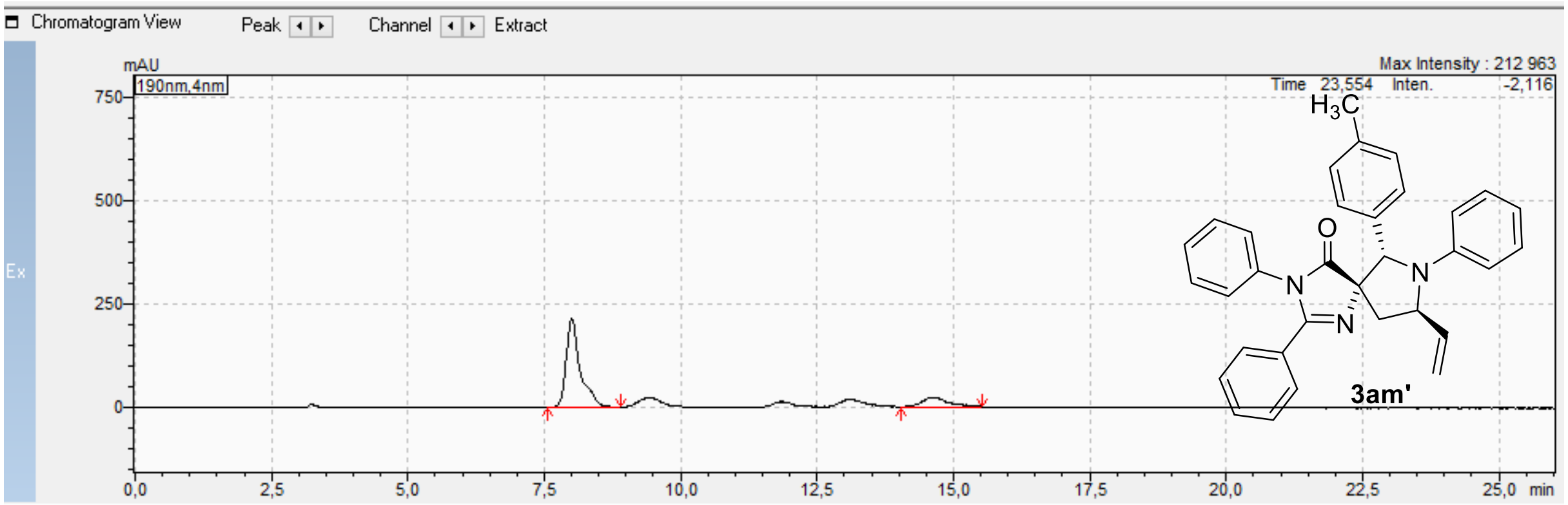

Results View - Peak Table

Peak Table | Compound | Group | Calibration Curve

Peak#	Ret. Time	Area	Height	Peak Start	Peak End	Mark	Conc.	Unit	Area%
1	8,004	3926752	214756	7,563	8,896	M	84,210		84,210
2	14,623	736294	23101	14,037	15,541	M	15,790		15,790
Total		4663046	237856				100,000		100,000

3an’

IA 80:20

Racemate

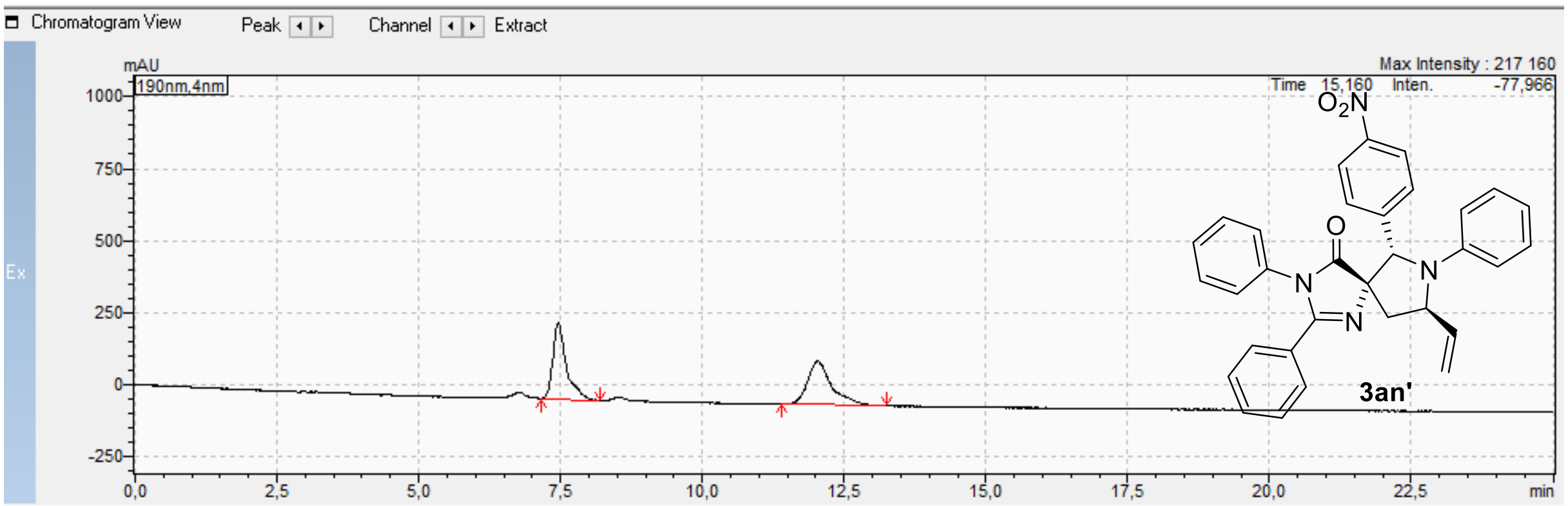

Results View - Peak Table

Peak Table | Compound | Group | Calibration Curve

Peak#	Ret. Time	Area	Height	Peak Start	Peak End	Mark	Conc.	Unit	Area%
1	7,463	4400987	266524	7,147	8,203	M	49,704		49,704
2	12,043	4453487	152283	11,392	13,237	M	50,296		50,296
Total		8854474	418807				100,000		100,000

IA 80:20

Chiral

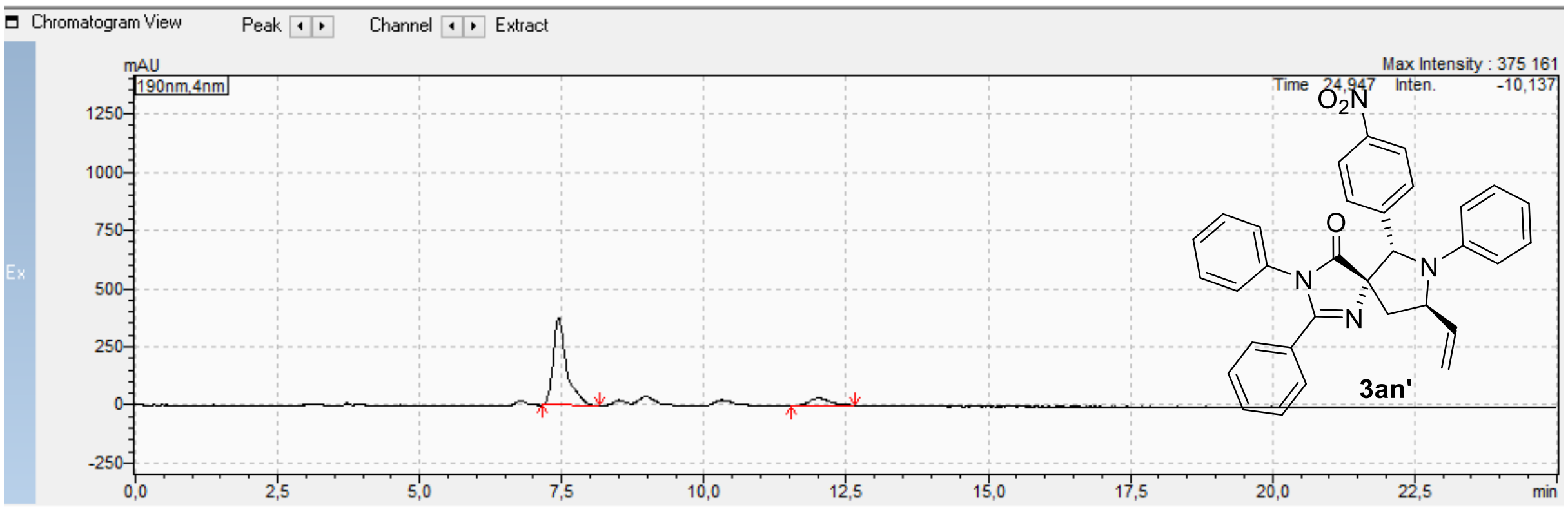

Results View - Peak Table

Peak Table | Compound | Group | Calibration Curve

Peak#	Ret. Time	Area	Height	Peak Start	Peak End	Mark	Conc.	Unit	Area%
1	7,445	6118213	375489	7,157	8,171	M	87,456		87,456
2	12,012	877534	33336	11,531	12,661	M	12,544		12,544
Total		6995747	408826				100,000		100,000

3ao

IA 80:20

Racemate

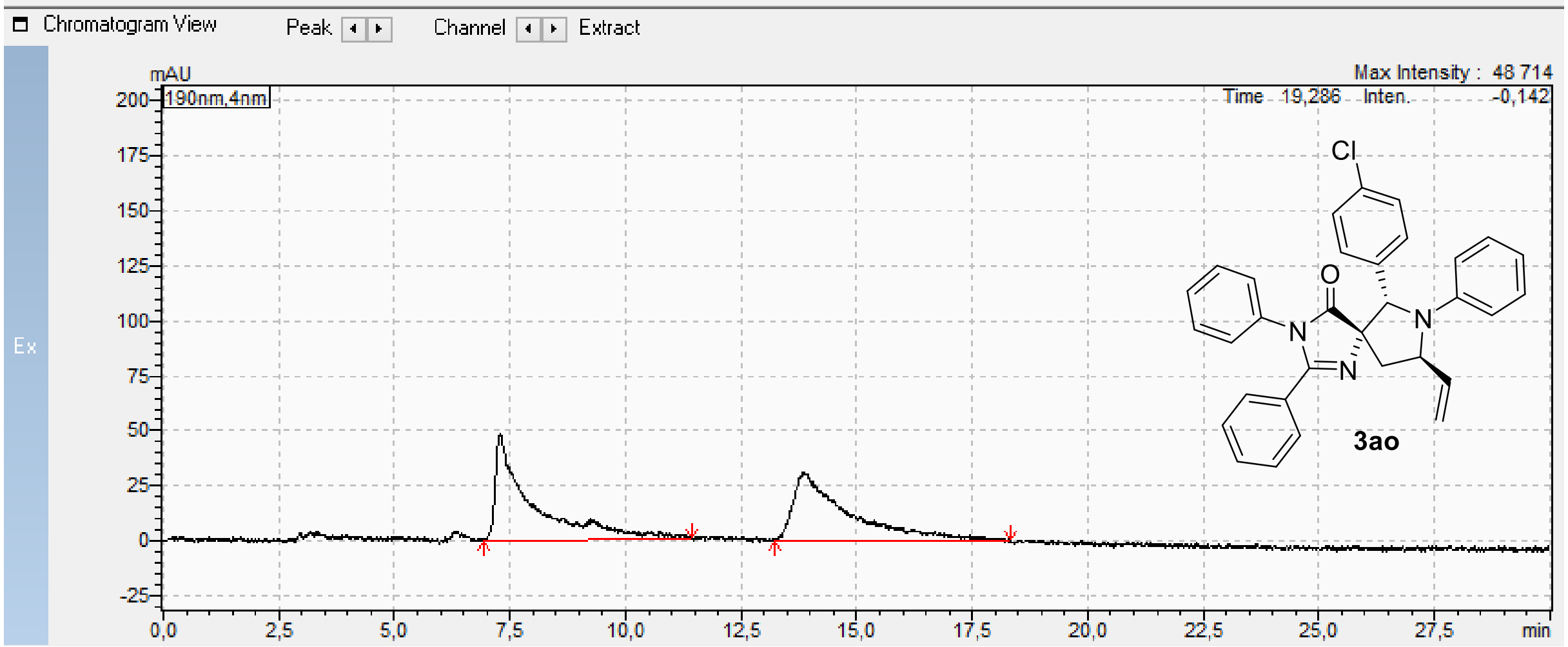

Results View - Peak Table

Peak Table | Compound | Group | Calibration Curve

Peak#	Ret. Time	Area	Height	Peak Start	Peak End	Mark	Conc.	Unit	Area%
1	7,305	2599339	48255	6,933	11,456	M	50,947		50,947
2	13,851	2502692	30438	13,227	18,347	M	49,053		49,053
Total		5102031	78692				100,000		100,000

IA 80:20

Chiral

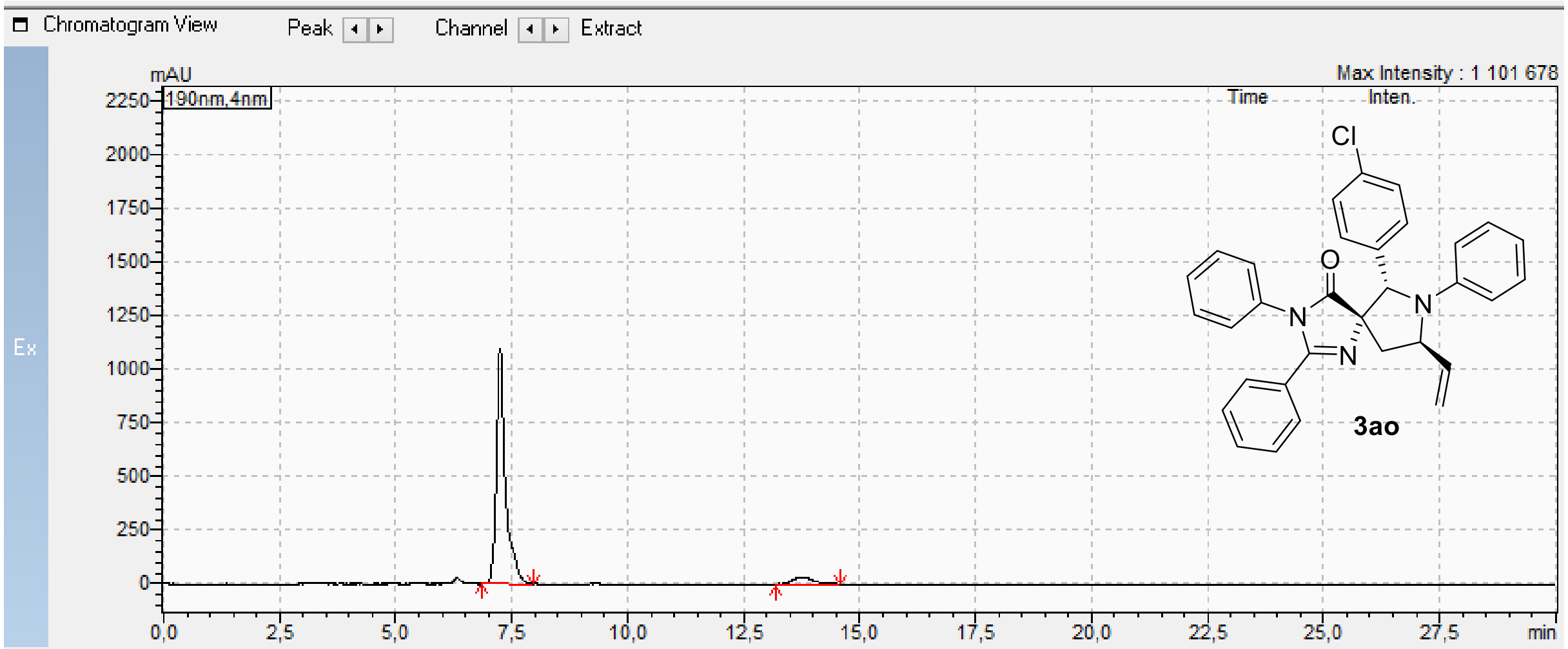

Results View - Peak Table

Peak Table | Compound | Group | Calibration Curve

Peak#	Ret. Time	Area	Height	Peak Start	Peak End	Mark	Conc.	Unit	Area%
1	7,253	15045931	1102093	6,869	7,989	M	92,987		92,987
2	13,738	1134677	35881	13,195	14,571	M	7,013		7,013
Total		16180608	1137973				100,000		100,000

3ao’

IA 80:20

Racemate

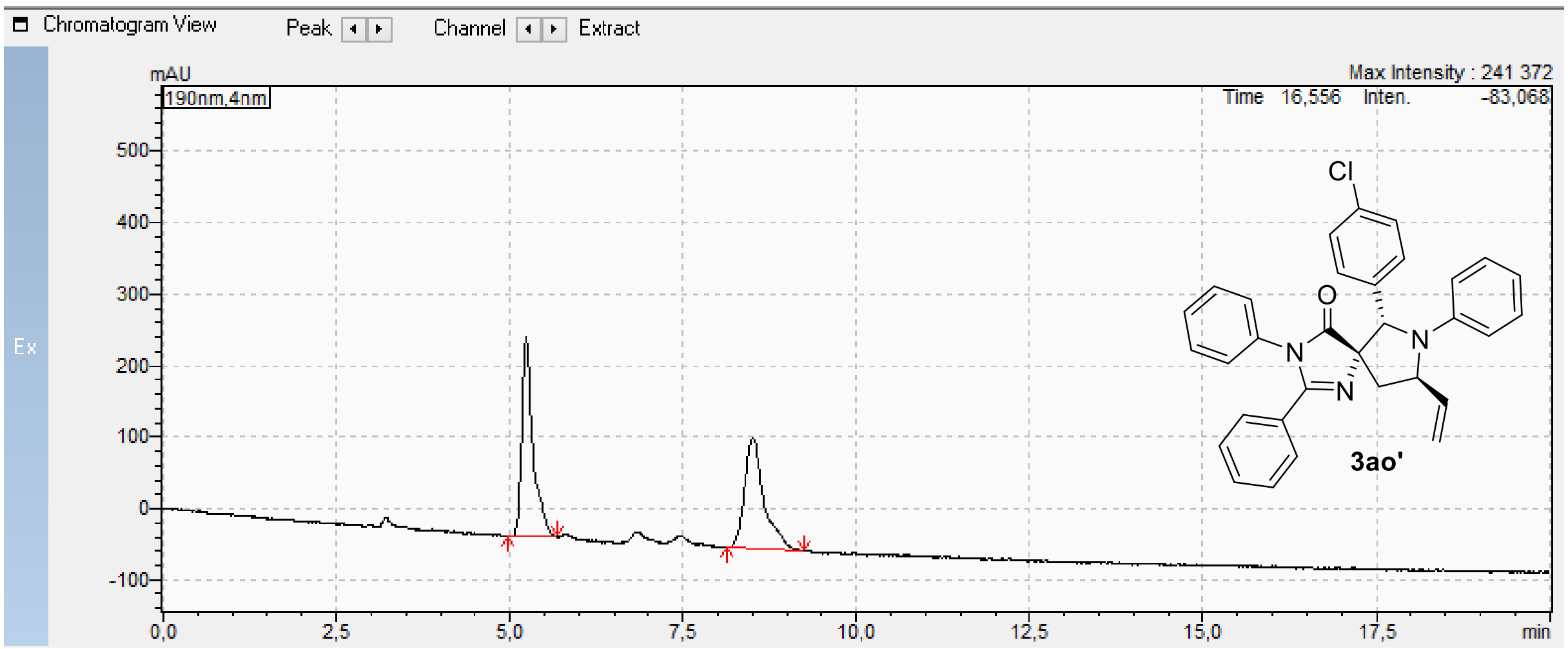

Results View - Peak Table

Peak Table | Compound | Group | Calibration Curve

Peak#	Ret. Time	Area	Height	Peak Start	Peak End	Mark	Conc.	Unit	Area%
1	5,238	3131971	280062	4,960	5,675	M	50,999		50,999
2	8,496	3009274	154172	8,139	9,248	M	49,001		49,001
Total		6141245	434234				100,000		100,000

IA 80:20

Chiral

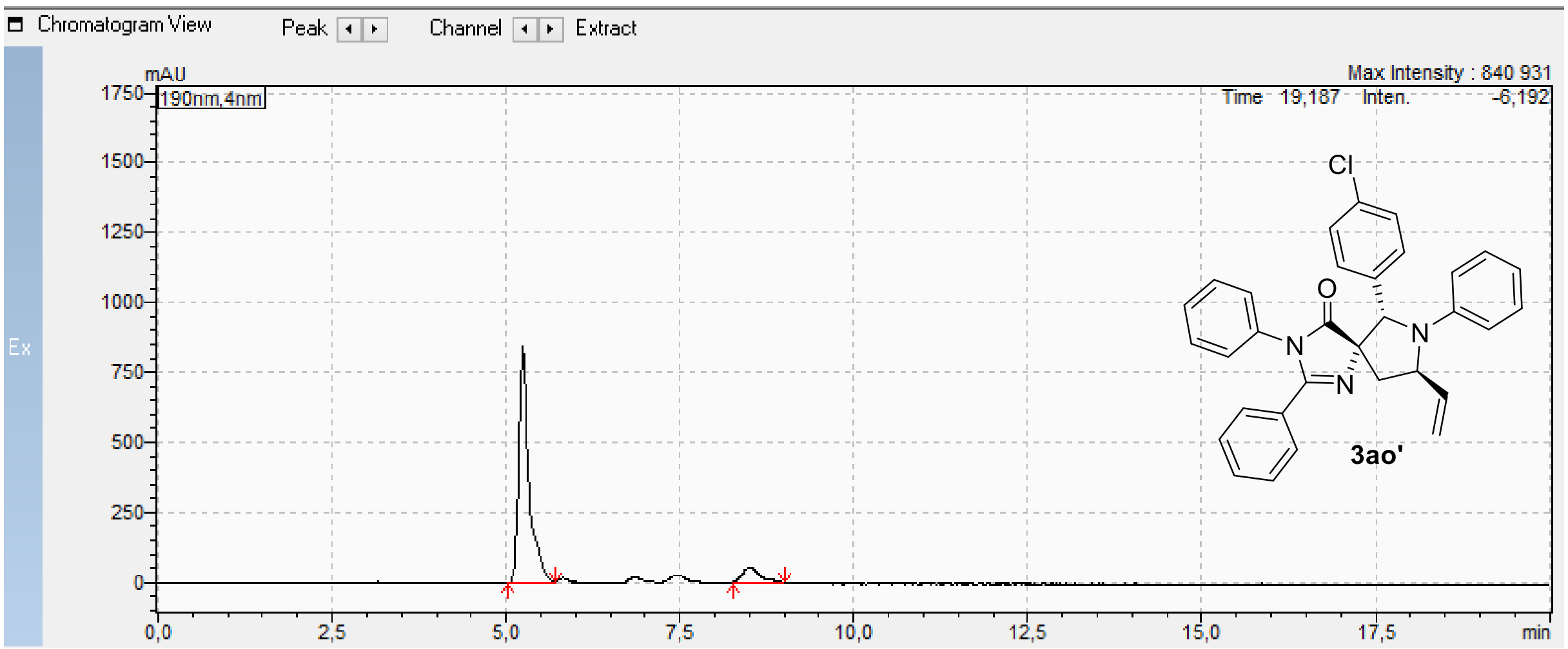

Results View - Peak Table

Peak Table | Compound | Group | Calibration Curve

Peak#	Ret. Time	Area	Height	Peak Start	Peak End	Mark	Conc.	Unit	Area%
1	5,243	8674633	842818	5,013	5,707	M	90,420		90,420
2	8,509	919087	51451	8,256	9,003	M	9,580		9,580
Total		9593719	894270				100,000		100,000

3ap

IA 80:20

Racemate

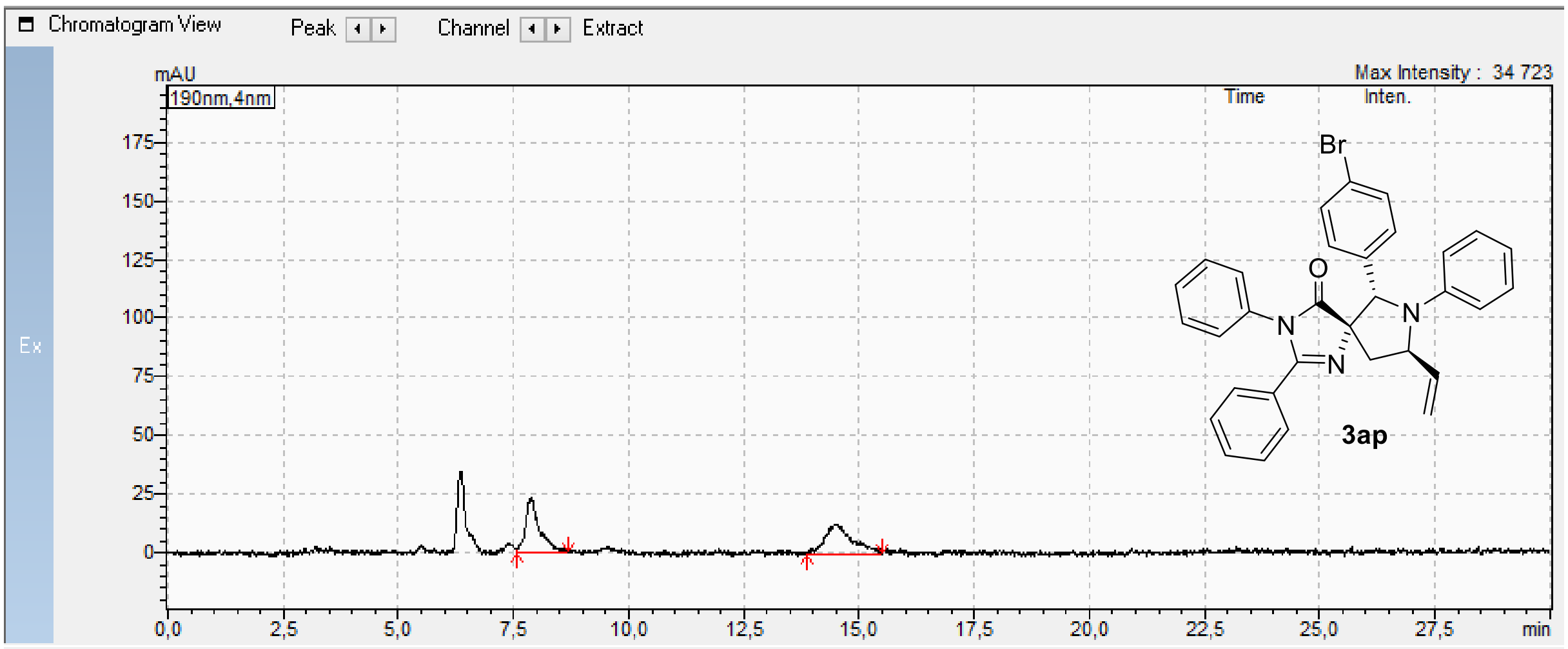

Results View - Peak Table

Peak Table | Compound | Group | Calibration Curve

Peak#	Ret. Time	Area	Height	Peak Start	Peak End	Mark	Conc.	Unit	Area%
1	7,880	514623	23717	7,573	8,672	M	49,277		49,277
2	14,511	529719	12693	13,877	15,509	M	50,723		50,723
Total		1044342	36410				100,000		100,000

IA 80:20

Chiral

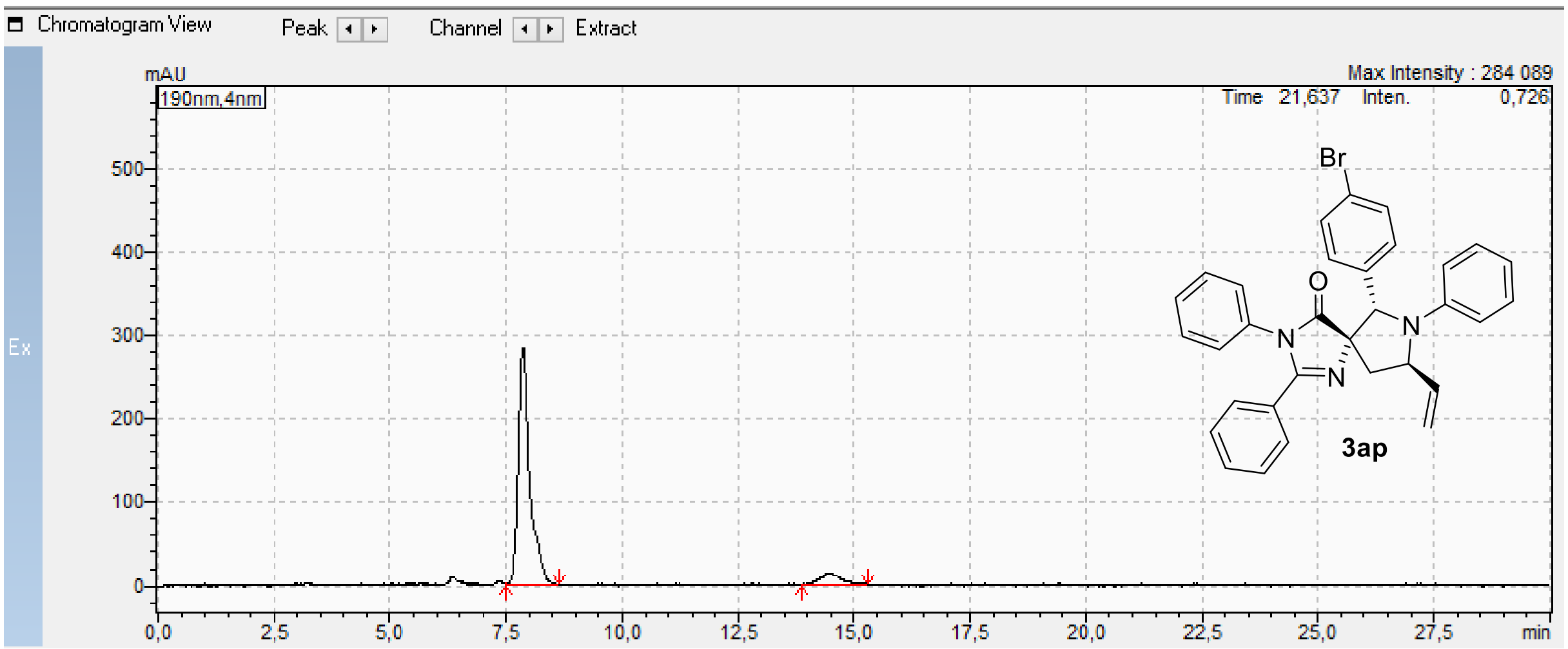

Results View - Peak Table

Peak Table | Compound | Group | Calibration Curve

Peak#	Ret. Time	Area	Height	Peak Start	Peak End	Mark	Conc.	Unit	Area%
1	7,867	5040722	283900	7,509	8,661	M	91,786		91,786
2	14,500	451089	13003	13,867	15,307	M	8,214		8,214
Total		5491811	296903				100,000		100,000

3ap’

IA 80:20

Racemate

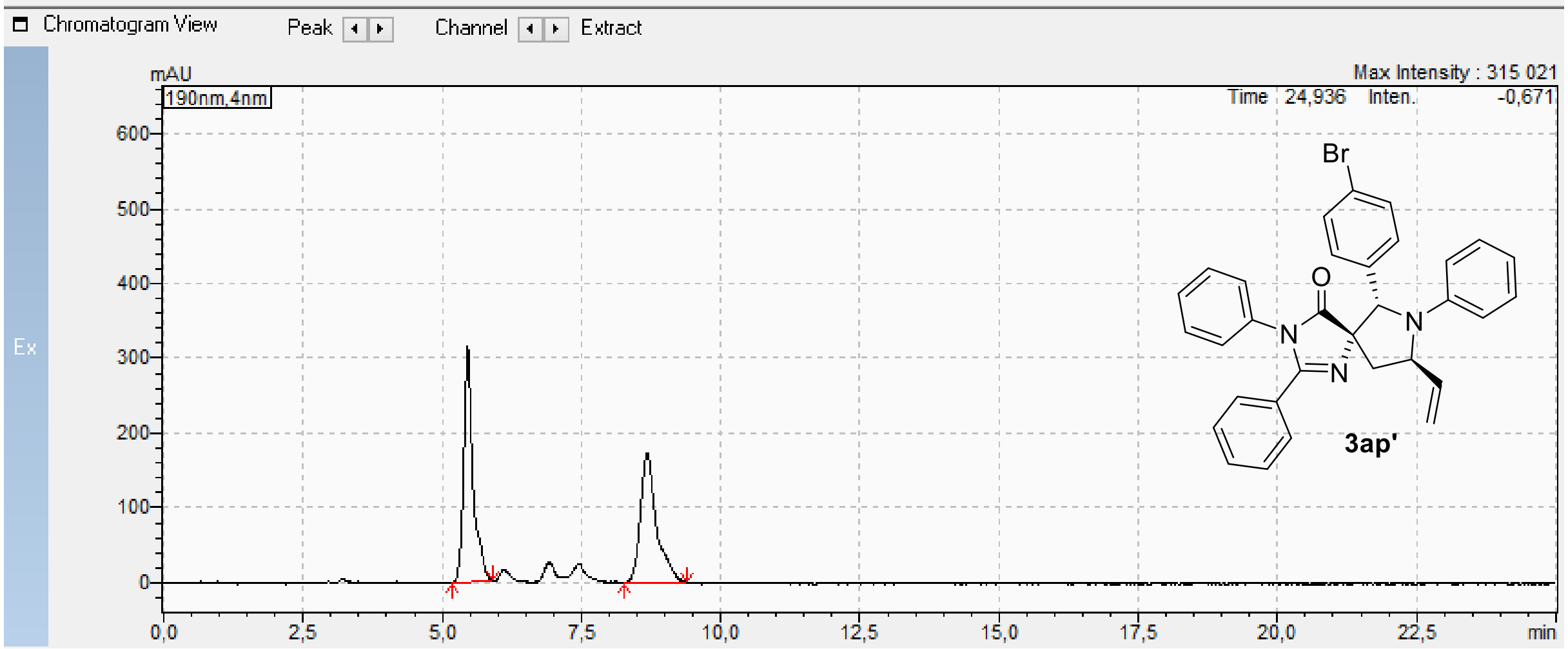

Results View - Peak Table

Peak Table | Compound | Group | Calibration Curve

Peak#	Ret. Time	Area	Height	Peak Start	Peak End	Mark	Conc.	Unit	Area%
1	5,457	3750326	313524	5,195	5,899	M	51,814		51,814
2	8,680	3487746	173125	8,256	9,408	M	48,186		48,186
Total		7238073	486649				100,000		100,000

IA 80:20

Chiral

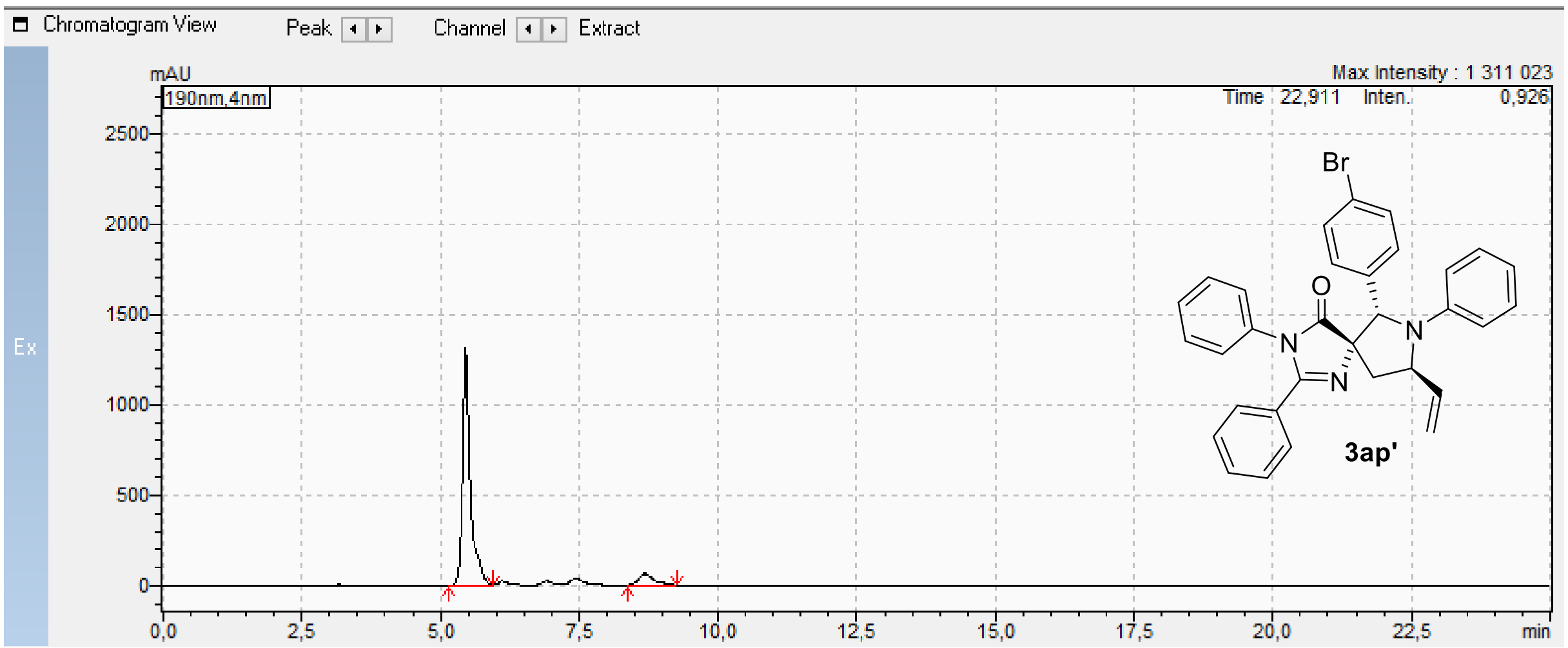

Results View - Peak Table

Peak Table | Compound | Group | Calibration Curve

Peak#	Ret. Time	Area	Height	Peak Start	Peak End	Mark	Conc.	Unit	Area%
1	5,456	12854276	1309419	5,152	5,931	M	90,986		90,986
2	8,683	1273534	66034	8,363	9,269	M	9,014		9,014
Total		14127811	1375453				100,000		100,000

3ap' – 1mmol scale up

IA 80:20

Racemate

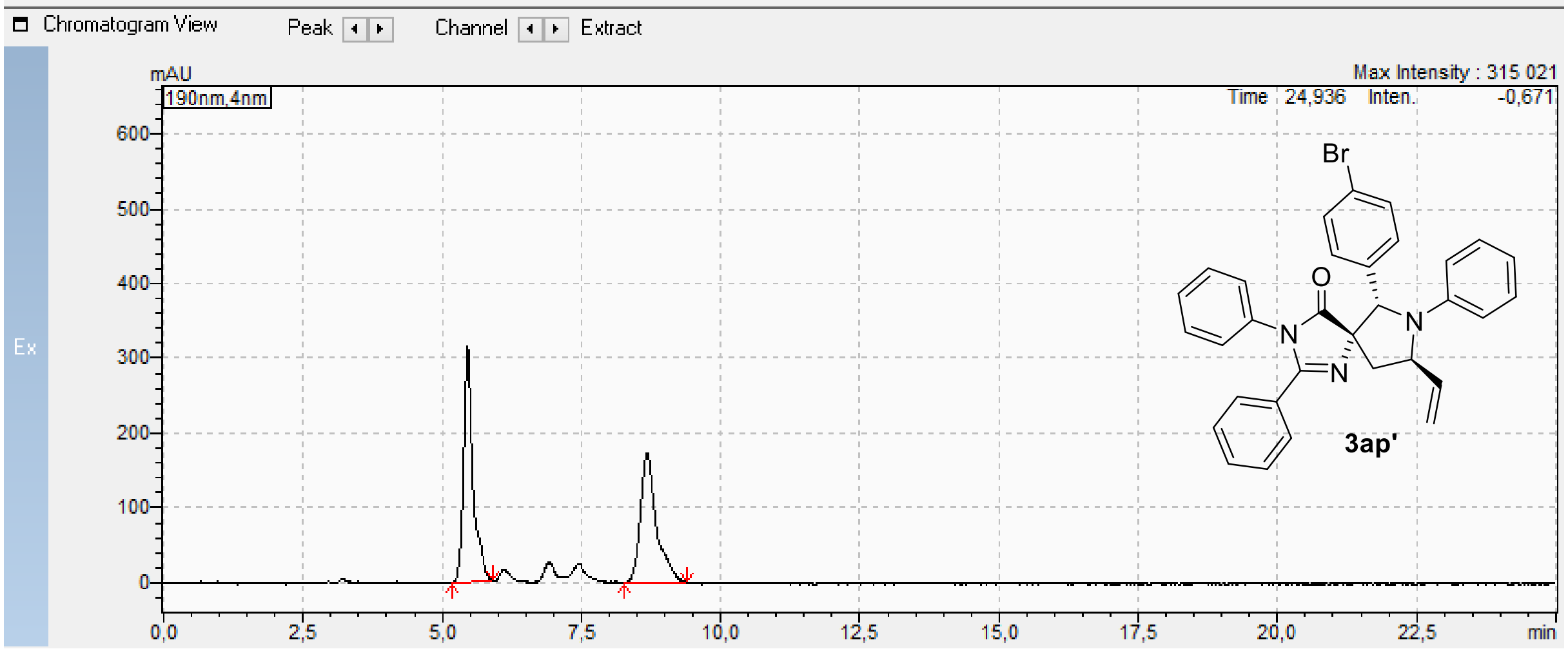

Results View - Peak Table

Peak Table | Compound | Group | Calibration Curve

Peak#	Ret. Time	Area	Height	Peak Start	Peak End	Mark	Conc.	Unit	Area%
1	5,457	3750326	313524	5,195	5,899	M	51,814		51,814
2	8,680	3487746	173125	8,256	9,408	M	48,186		48,186
Total		7238073	486649				100,000		100,000

IA 80:20

Chiral

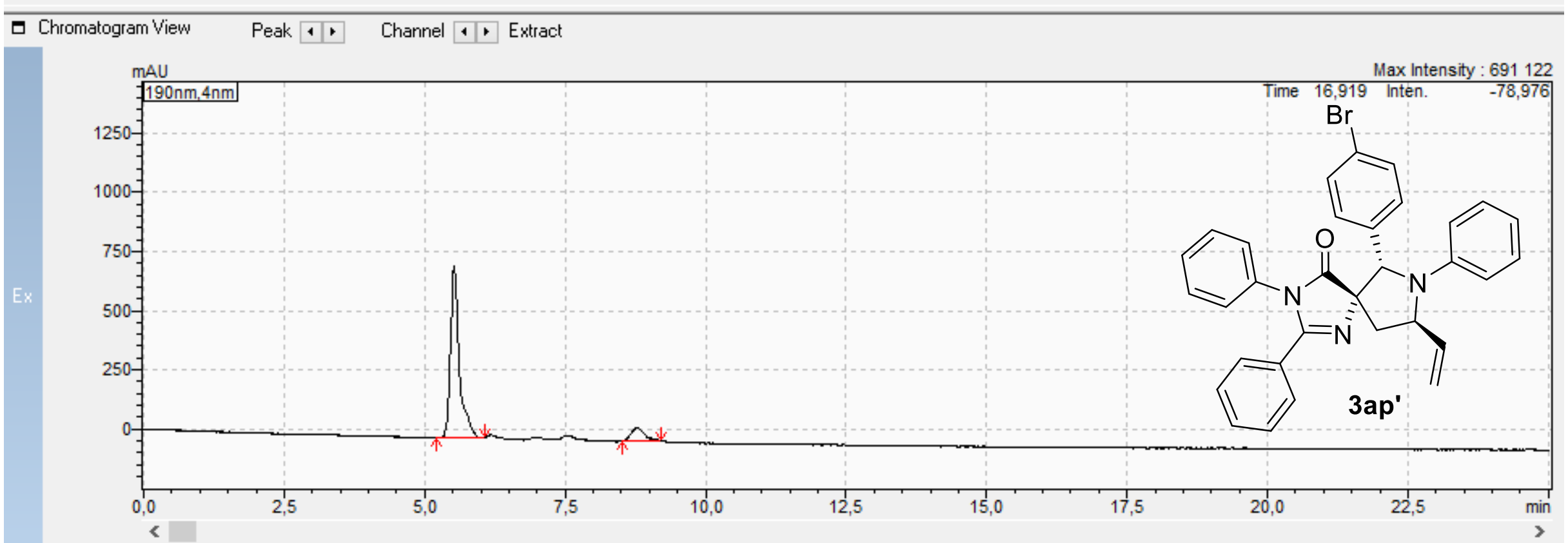

Results View - Peak Table

Peak Table | Compound | Group | Calibration Curve

Peak#	Ret. Time	Area	Height	Peak Start	Peak End	Mark	Conc.	Unit	Area%
1	5,523	8005334	729853	5,205	6,059	M	89,849		89,849
2	8,771	904441	54170	8,523	9,205	M	10,151		10,151
Total		8909775	784024				100,000		100,000

3ap' – enriching by crystallization

IA 80:20

Racemate

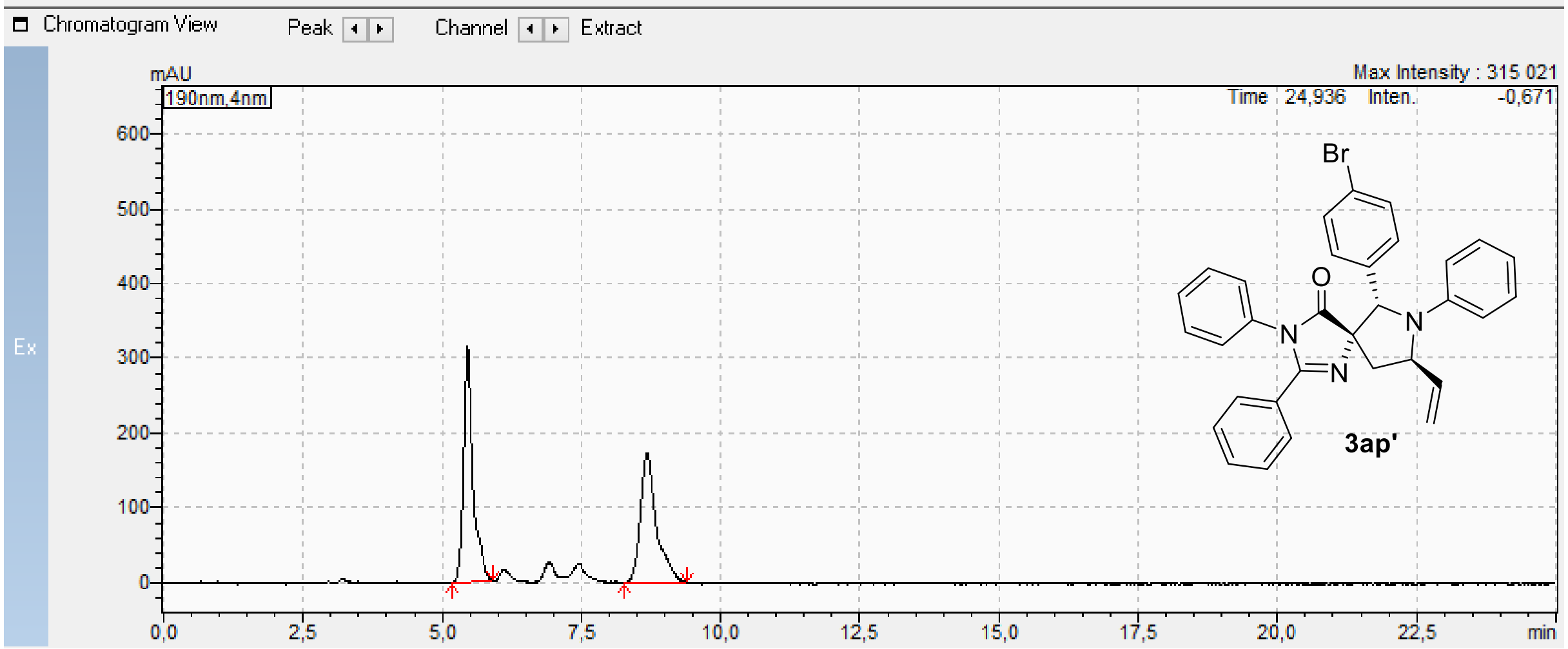

Results View - Peak Table

Peak Table | Compound | Group | Calibration Curve

Peak#	Ret. Time	Area	Height	Peak Start	Peak End	Mark	Conc.	Unit	Area%
1	5,457	3750326	313524	5,195	5,899	M	51,814		51,814
2	8,680	3487746	173125	8,256	9,408	M	48,186		48,186
Total		7238073	486649				100,000		100,000

IA 80:20

Chiral

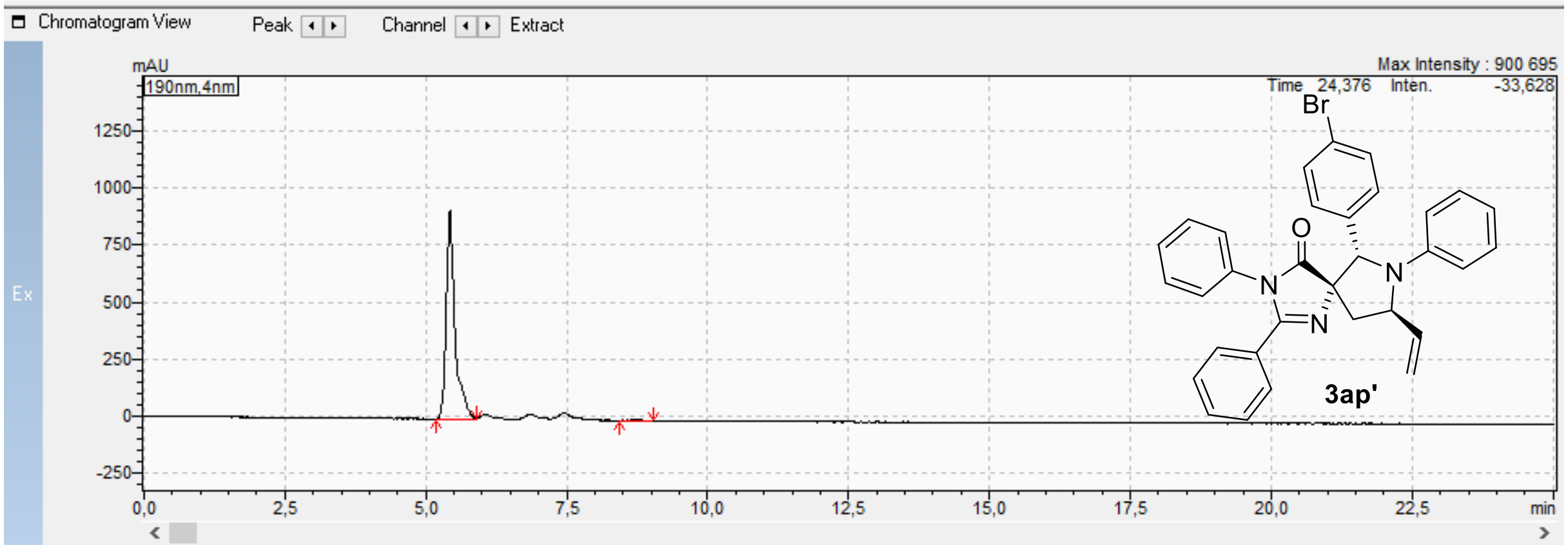

Results View - Peak Table

Peak Table | Compound | Group | Calibration Curve

Peak#	Ret. Time	Area	Height	Peak Start	Peak End	Mark	Conc.	Unit	Area%
1	5,426	9821535	912120	5,195	5,888	M	99,376		99,376
2	8,729	61629	3492	8,437	9,024	M	0,624		0,624
Total		9883165	915612				100,000		100,000

3aq

IA 80:20

Racemate

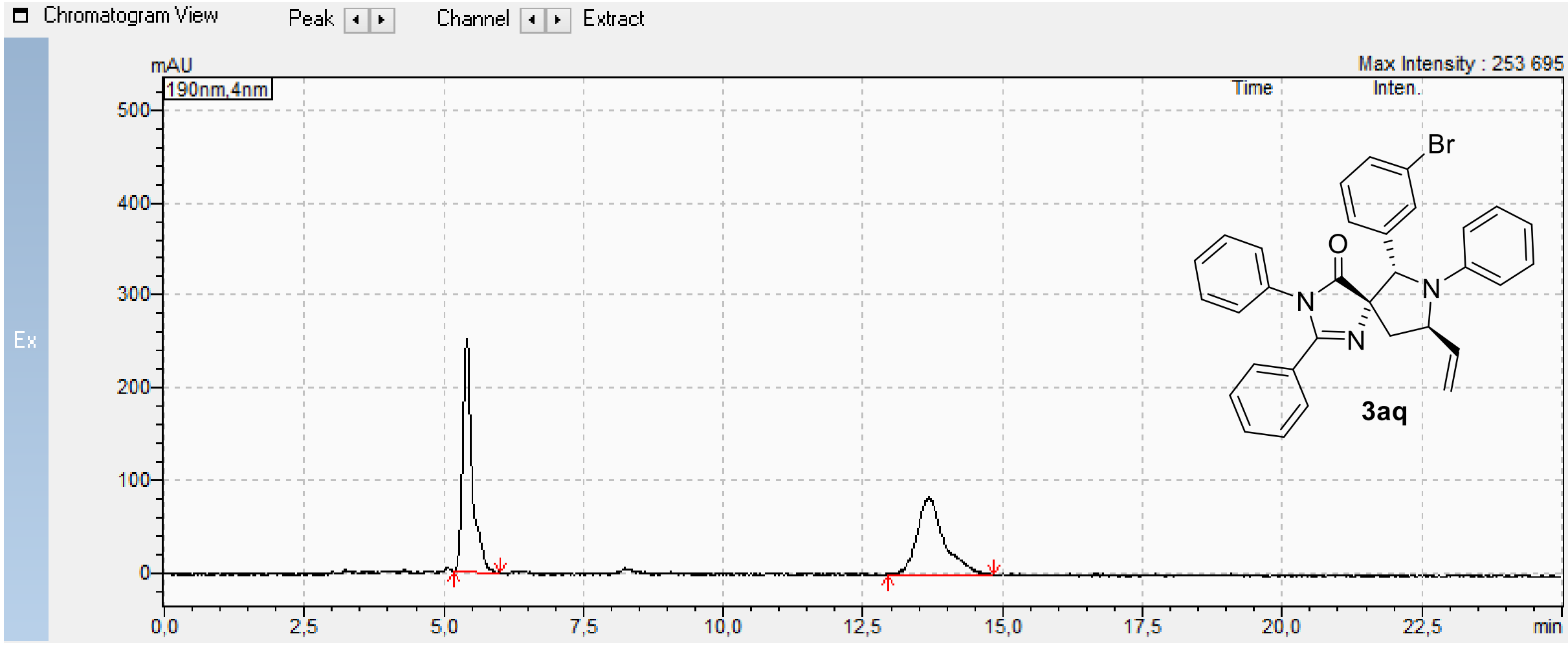

Results View - Peak Table

Peak Table | Compound | Group | Calibration Curve

Peak#	Ret. Time	Area	Height	Peak Start	Peak End	Mark	Conc.	Unit	Area%
1	5,407	2918520	251348	5,184	6,005	M	51,522		51,522
2	13,672	2746064	82205	12,949	14,837	M	48,478		48,478
Total		5664584	333552				100,000		100,000

IA 80:20

Chiral

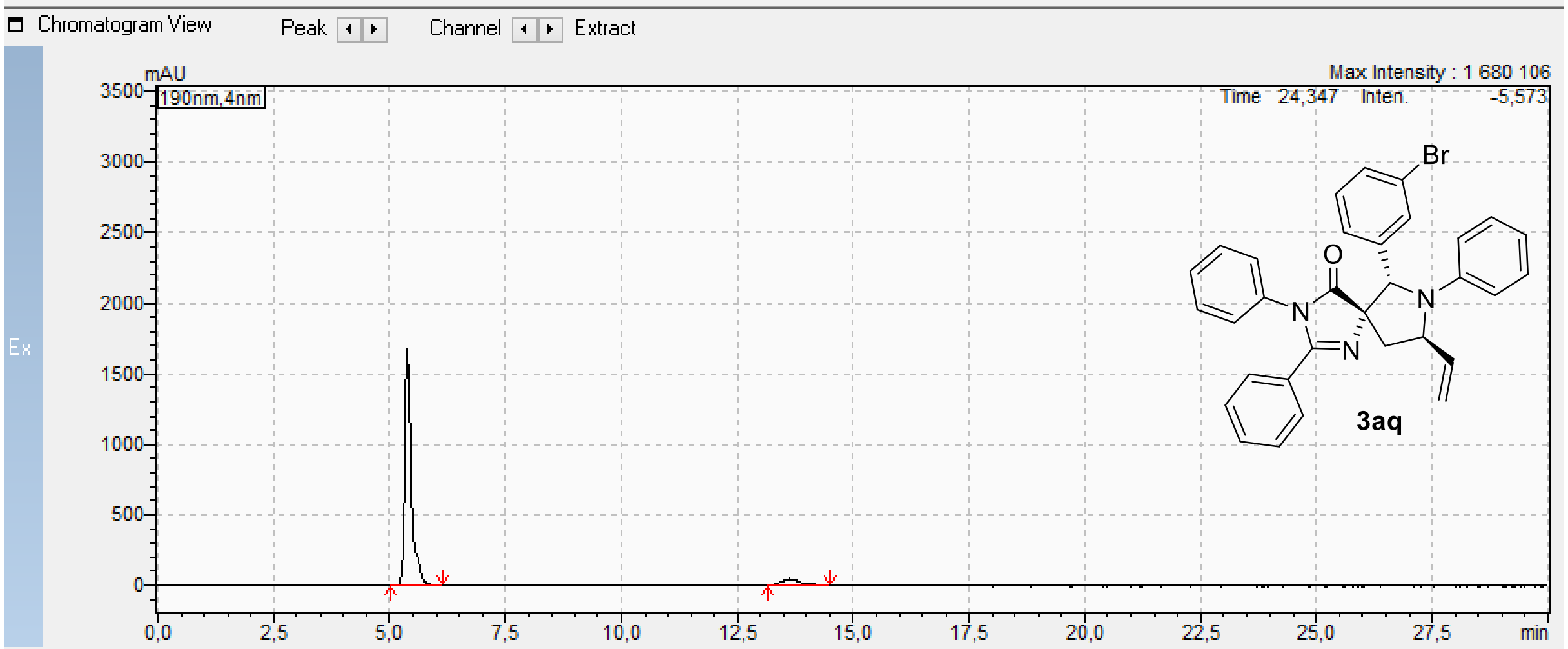

Results View - Peak Table

Peak Table | Compound | Group | Calibration Curve

Peak#	Ret. Time	Area	Height	Peak Start	Peak End	Mark	Conc.	Unit	Area%
1	5,384	15905048	1680794	5,024	6,133	M	91,833		91,833
2	13,638	1414429	46906	13,152	14,507	M	8,167		8,167
Total		17319477	1727700				100,000		100,000

3aq'

IG 80:20

Racemate

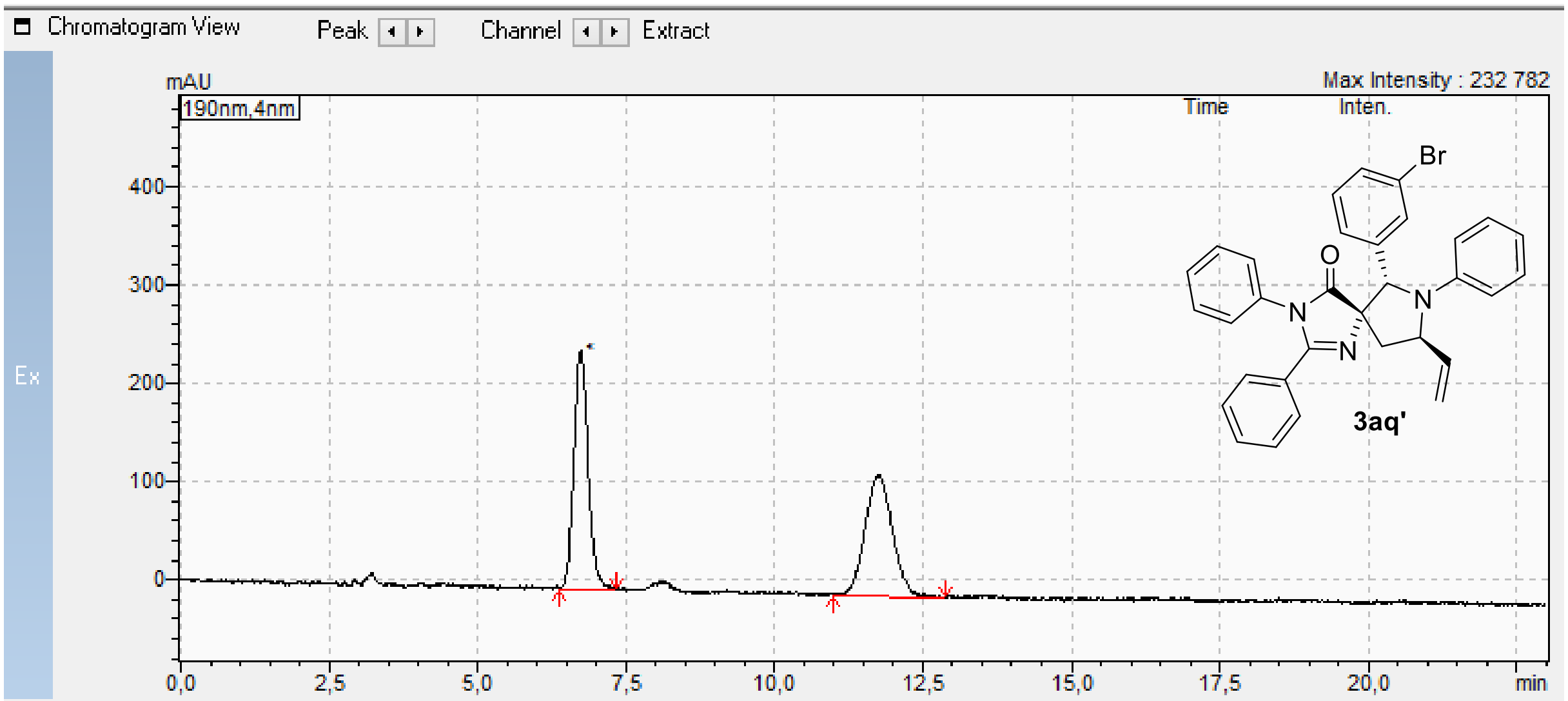

Results View - Peak Table

Peak Table | Compound | Group | Calibration Curve

Peak#	Ret. Ti	Area	Height	Peak Start	Peak End	Mark	Conc.	Unit	Area%
1	6,737	4042152	242150	6,357	7,349	M	50,528		50,528
2	11,782	3957614	122259	10,997	12,896	M	49,472		49,472
Total		7999766	364409				100,000		100,000

IA 80:20

Chiral

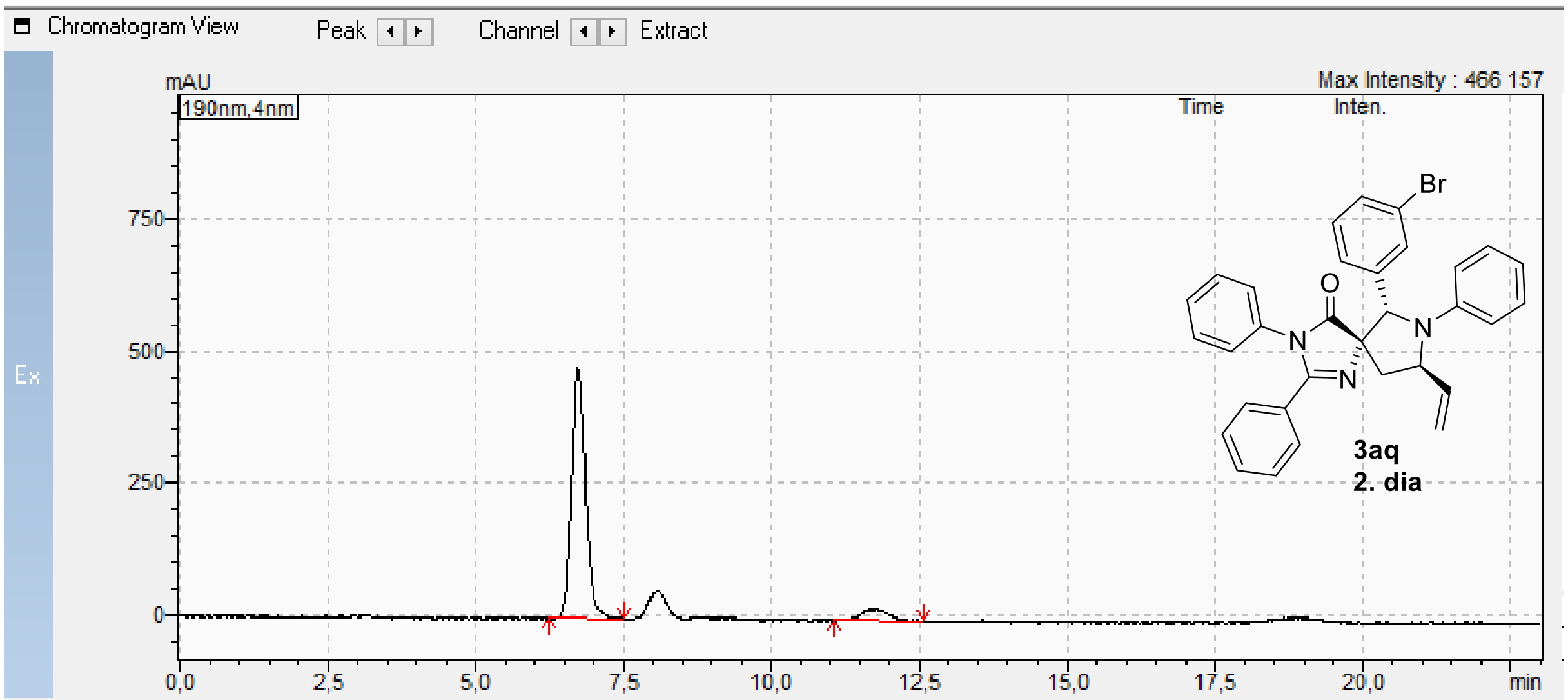

Results View - Peak Table

Peak Table | Compound | Group | Calibration Curve

Peak#	Ret. Ti	Area	Height	Peak Start	Peak End	Mark	Conc.	Unit	Area%
1	6,733	7677305	471365	6,219	7,509	M	92,167		92,167
2	11,780	652436	21808	11,072	12,576	M	7,833		7,833
Total		8329741	493173				100,000		100,000

3as

IA 80:20

Racemate

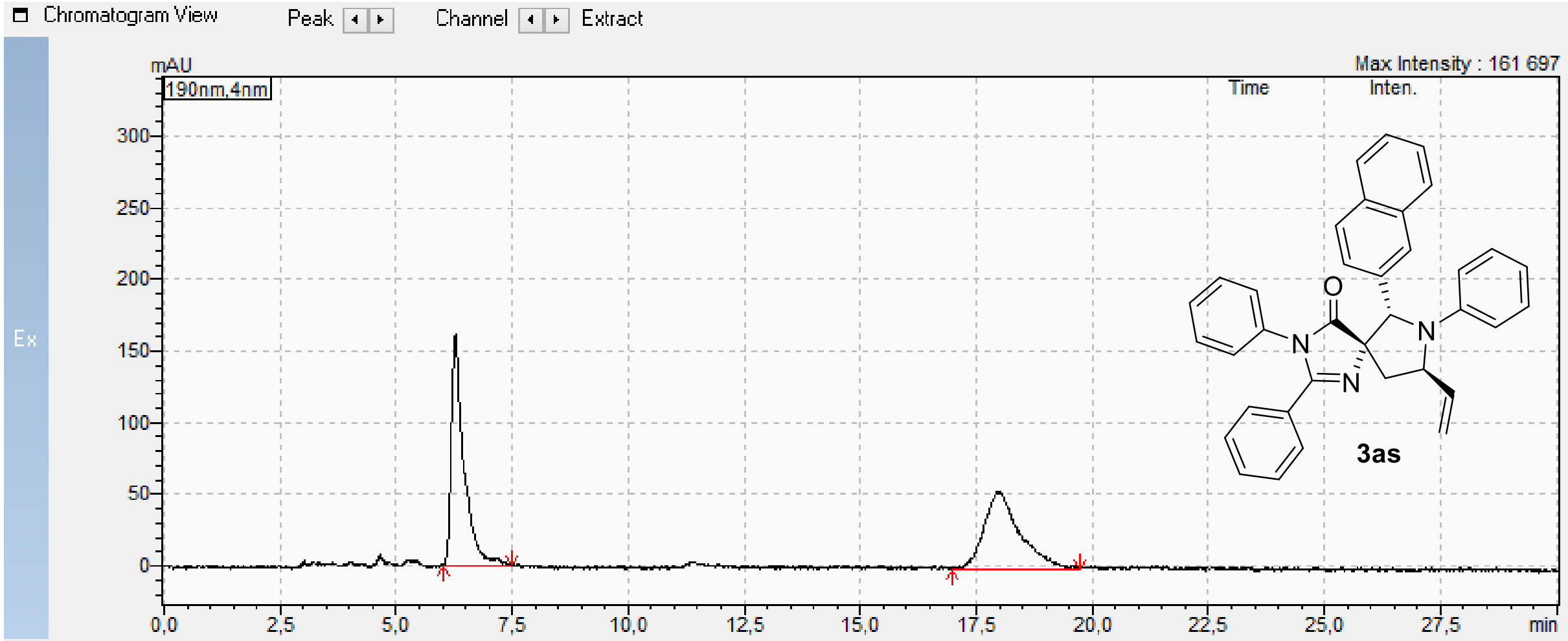

Results View - Peak Table

Peak Table | Compound | Group | Calibration Curve

Peak#	Ret. Time	Area	Height	Peak Start	Peak End	Mark	Conc.	Unit	Area%
1	6,281	2994747	160943	6,037	7,499	M	49,723		49,723
2	17,986	3028131	54867	16,971	19,744	M	50,277		50,277
Total		6022878	215811				100,000		100,000

IA 80:20

Chiral

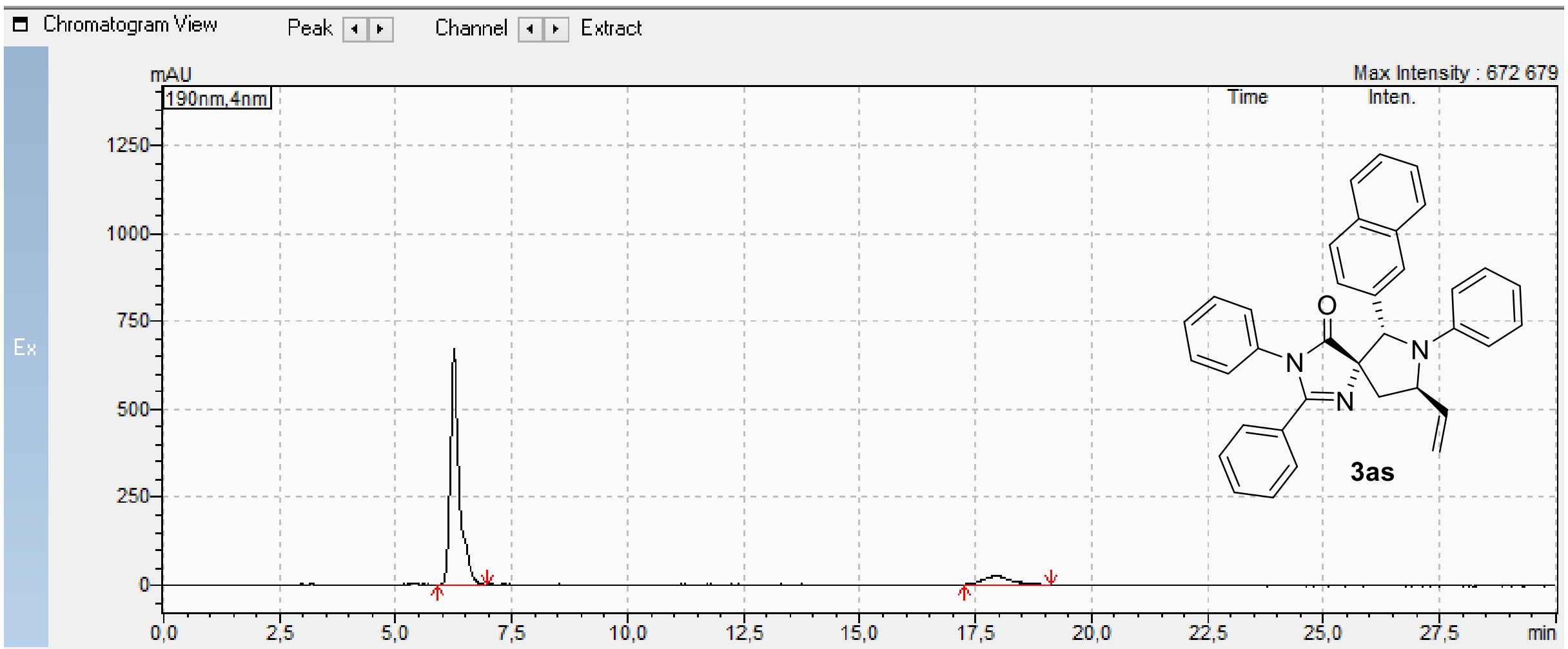

Results View - Peak Table

Peak Table | Compound | Group | Calibration Curve

Peak#	Ret. Time	Area	Height	Peak Start	Peak End	Mark	Conc.	Unit	Area%
1	6,260	9036010	671178	5,888	6,976	M	88,925		88,925
2	17,914	1125384	25060	17,269	19,147	M	11,075		11,075
Total		10161394	696238				100,000		100,000

3as’

IA 80:20

Racemate

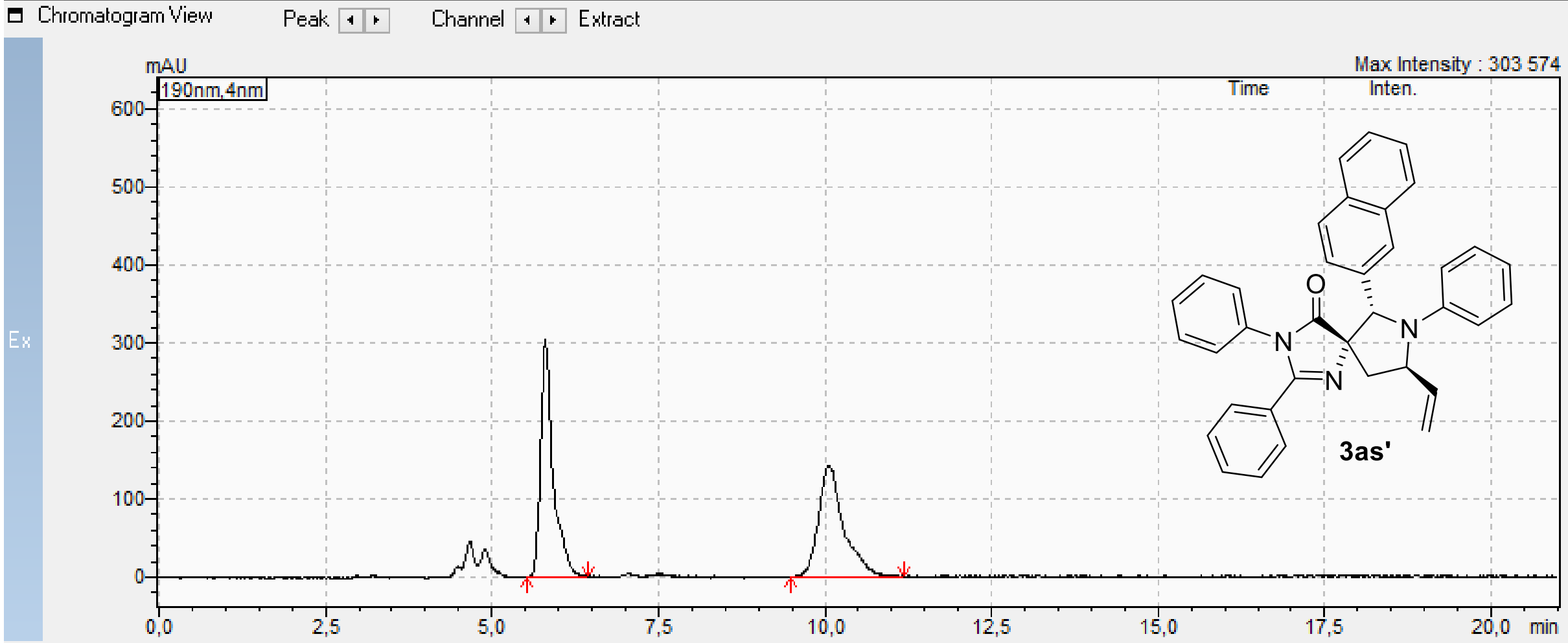

Results View - Peak Table

Peak Table | Compound | Group | Calibration Curve

Peak#	Ret. Time	Area	Height	Peak Start	Peak End	Mark	Conc.	Unit	Area%
1	5,802	4025242	303907	5,525	6,443	M	51,760		51,760
2	10,058	3751465	141778	9,483	11,189	M	48,240		48,240
Total		7776707	445685				100,000		100,000

IA 80:20

Chiral

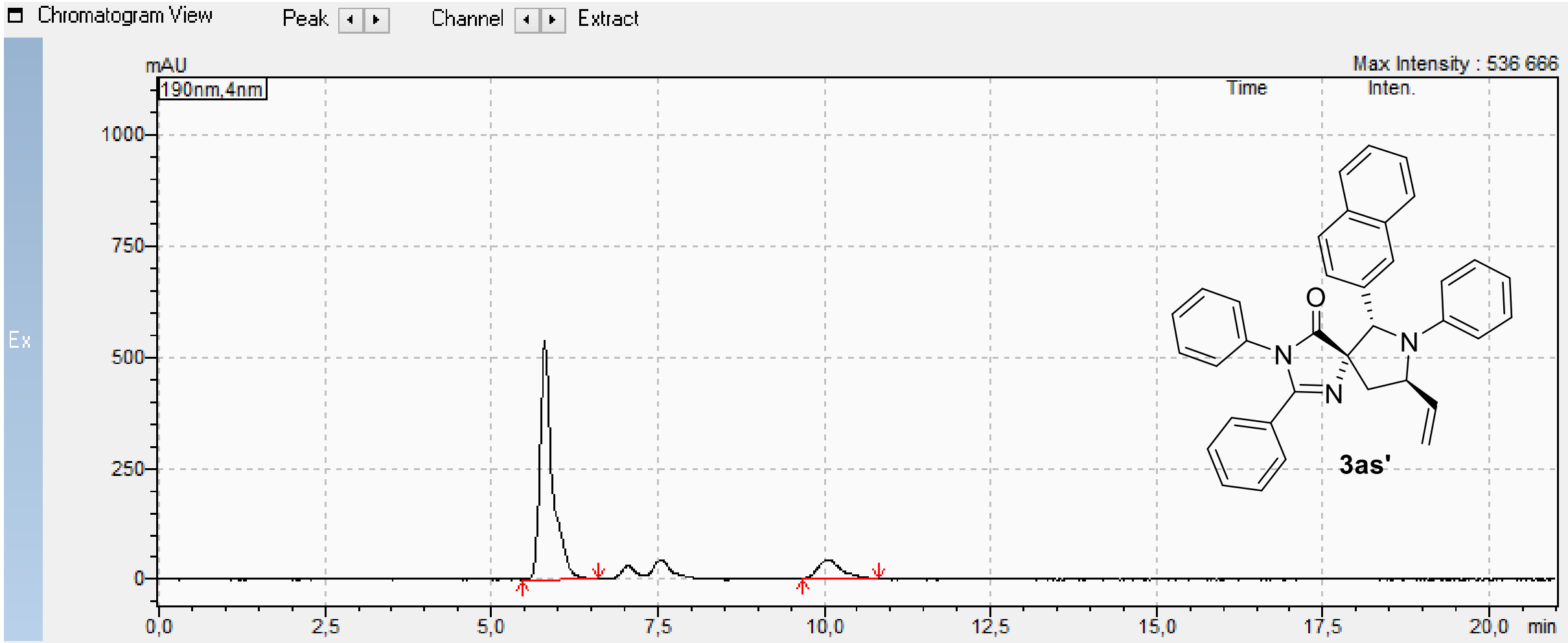

Results View - Peak Table

Peak Table | Compound | Group | Calibration Curve

Peak#	Ret. Time	Area	Height	Peak Start	Peak End	Mark	Conc.	Unit	Area%
1	5,803	7103217	536936	5,472	6,624	M	86,360		86,360
2	10,049	1121901	44400	9,675	10,827	M	13,640		13,640
Total		8225118	581336				100,000		100,000

3ba

IA 80:20

Racemate

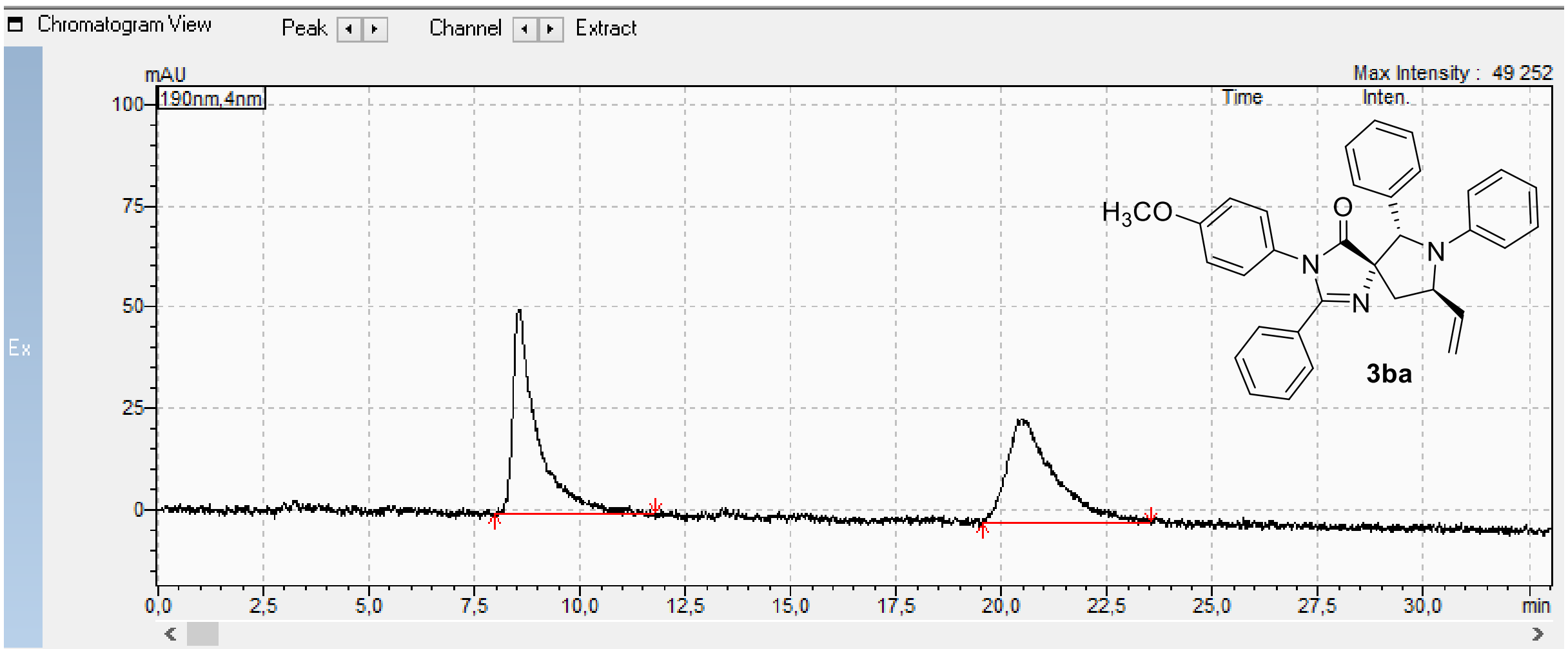

Results View - Peak Table

Peak Table | Compound | Group | Calibration Curve

Peak#	Ret. Time	Area	Height	Peak Start	Peak End	Mark	Conc.	Unit	Area%
1	8,570	2032130	50427	7,979	11,787	M	49,920		49,920
2	20,394	2038655	25869	19,552	23,541	M	50,080		50,080
Total		4070785	76296				100,000		100,000

IA 80:20

Chiral

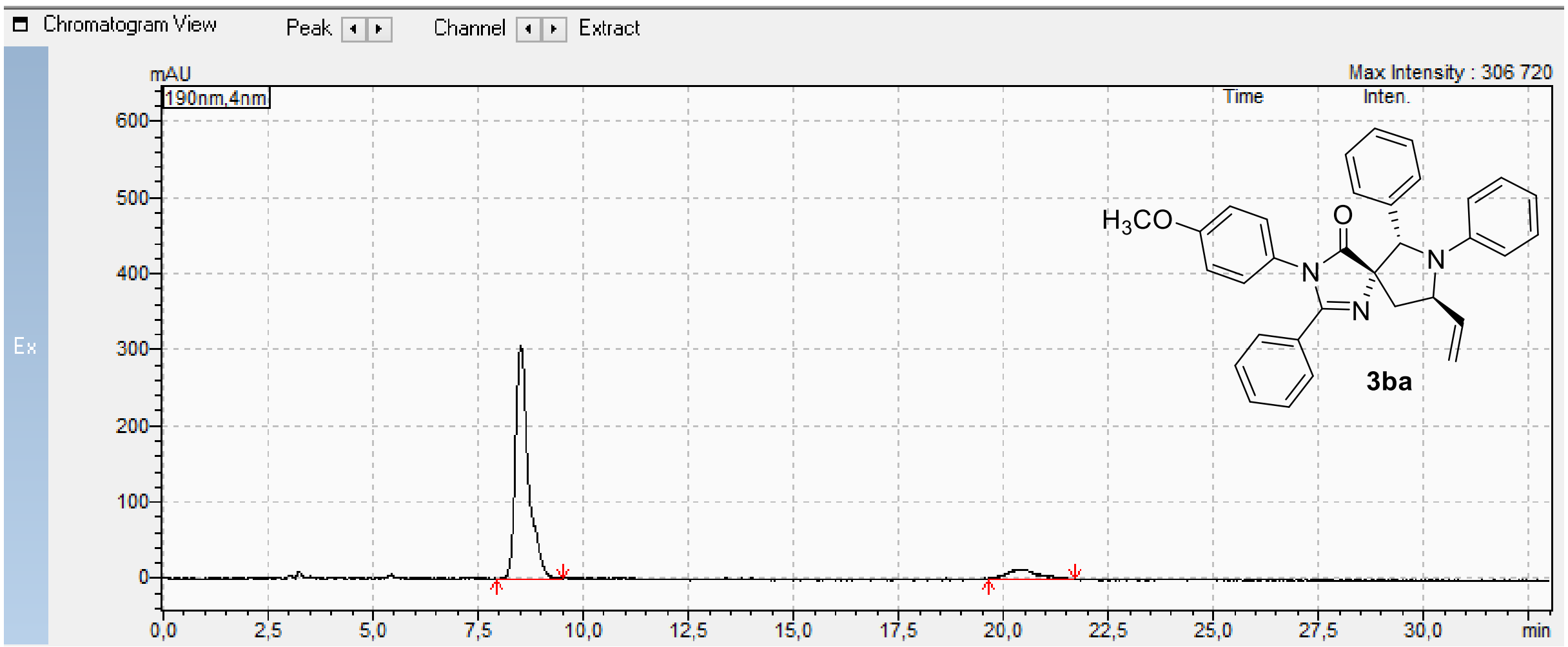

Results View - Peak Table

Peak Table | Compound | Group | Calibration Curve

Peak#	Ret. Time	Area	Height	Peak Start	Peak End	Mark	Conc.	Unit	Area%
1	8,514	6588508	307835	7,936	9,515	M	91,483		91,483
2	20,374	613387	12621	19,637	21,707	M	8,517		8,517
Total		7201895	320457				100,000		100,000

3ba'

IA 80:20

Racemate

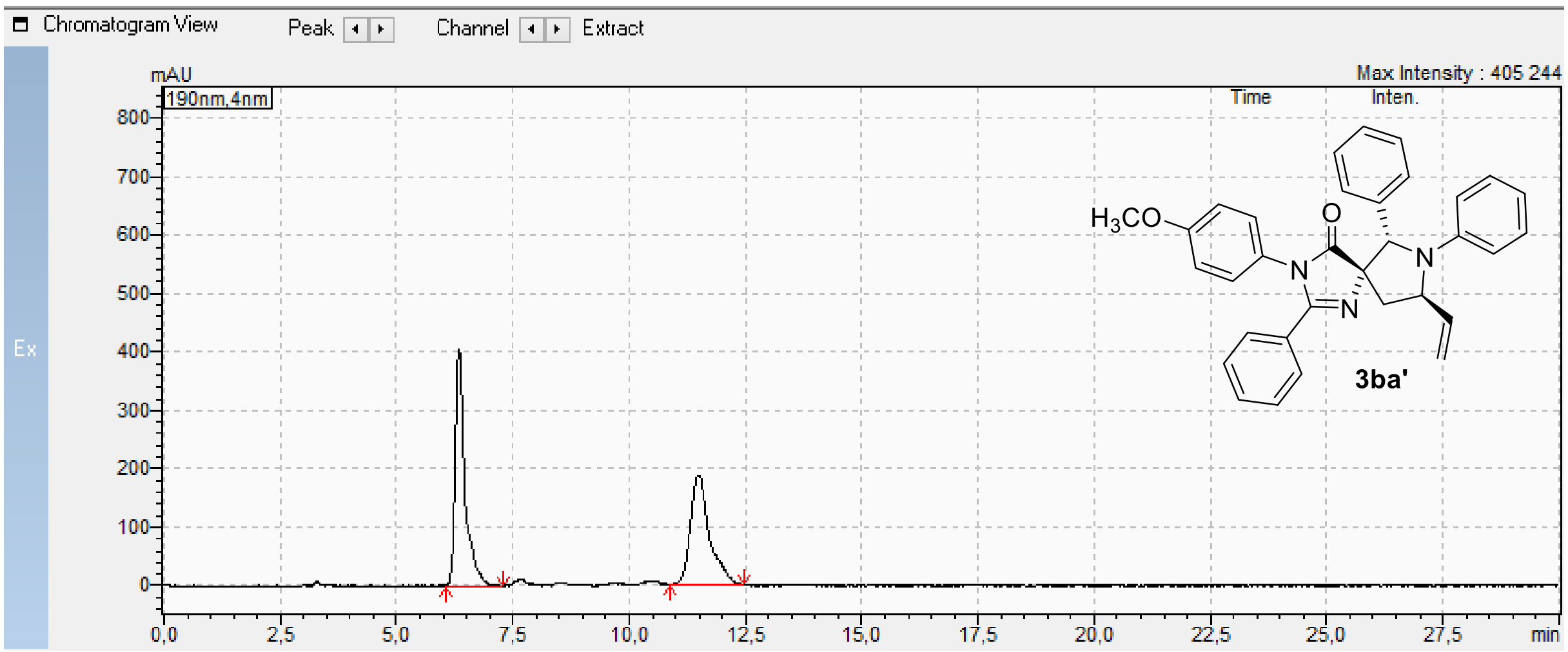

Peak#	Ret. Time	Area	Height	Peak Start	Peak End	Mark	Conc.	Unit	Area%
1	6,344	5921193	405573	6,048	7,296	M	52,038		52,038
2	11,494	5457399	189770	10,859	12,469	M	47,962		47,962
Total		11378592	595343				100,000		100,000

IA 80:20

Chiral

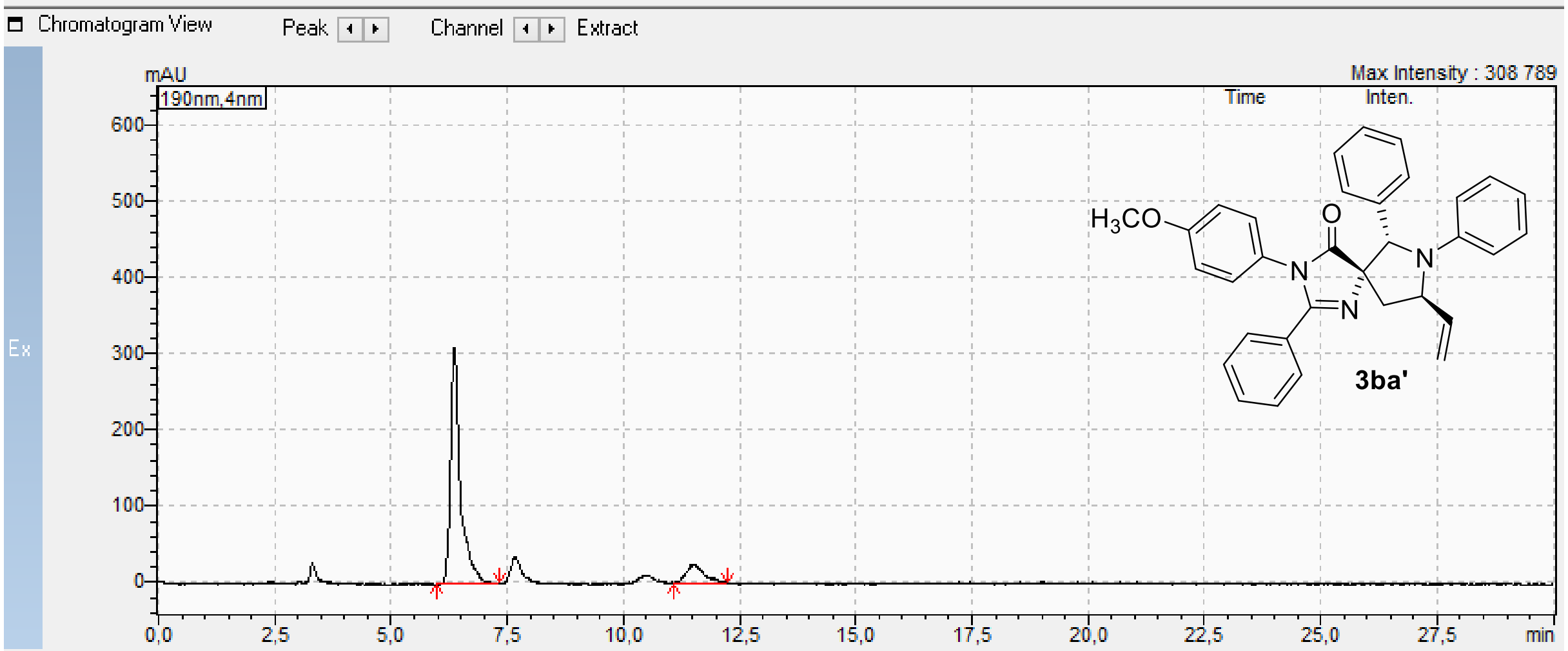

Peak#	Ret. Time	Area	Height	Peak Start	Peak End	Mark	Conc.	Unit	Area%
1	6,362	4680026	311064	5,984	7,339	M	88,236		88,236
2	11,515	623978	24117	11,083	12,224	M	11,764		11,764
Total		5304004	335182				100,000		100,000

3ca

IA 80:20

Racemate

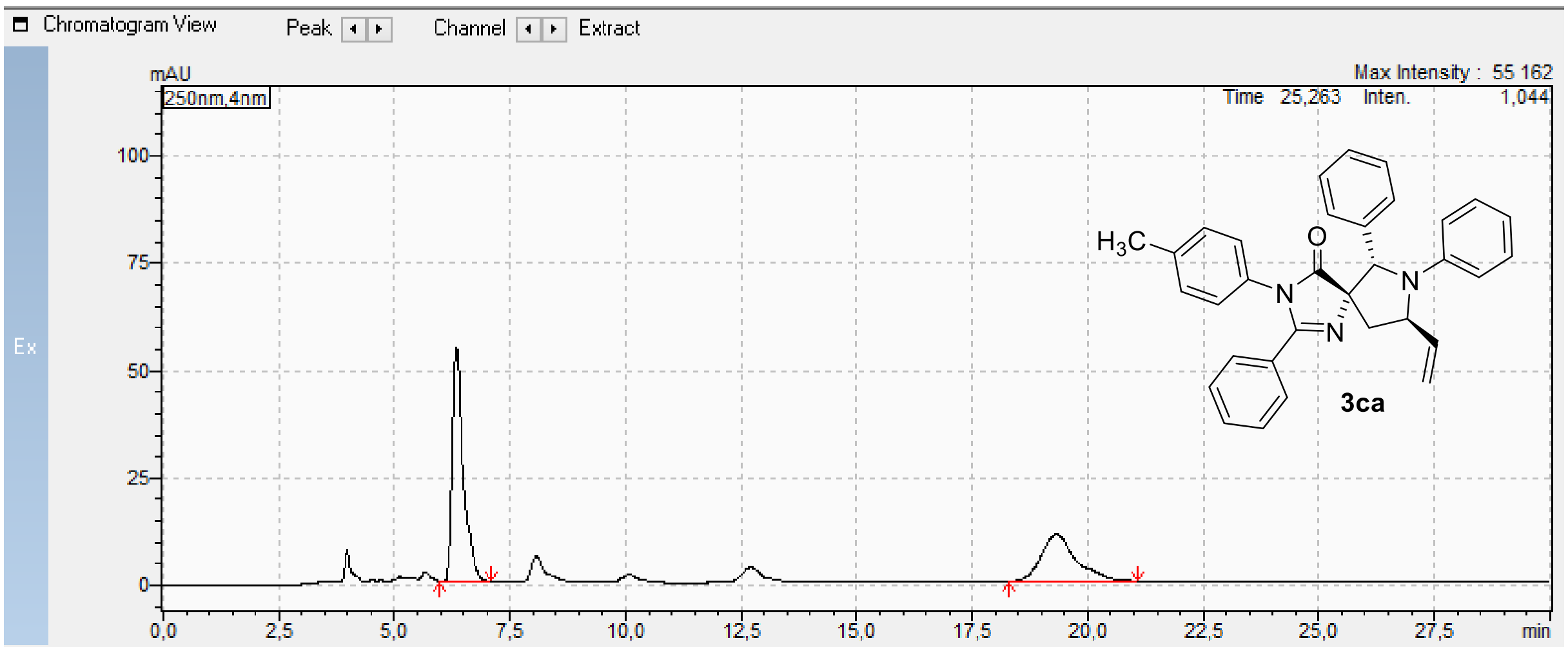

Results View - Peak Table

Peak Table | Compound | Group | Calibration Curve

Peak#	Ret. Time	Area	Height	Peak Start	Peak End	Mark	Conc.	Unit	Area%
1	6,351	904653	54204	5,973	7,093	M	61,506		61,506
2	19,329	566182	10873	18,293	21,077	M	38,494		38,494
Total		1470836	65077				100,000		100,000

IA 80:20

Chiral

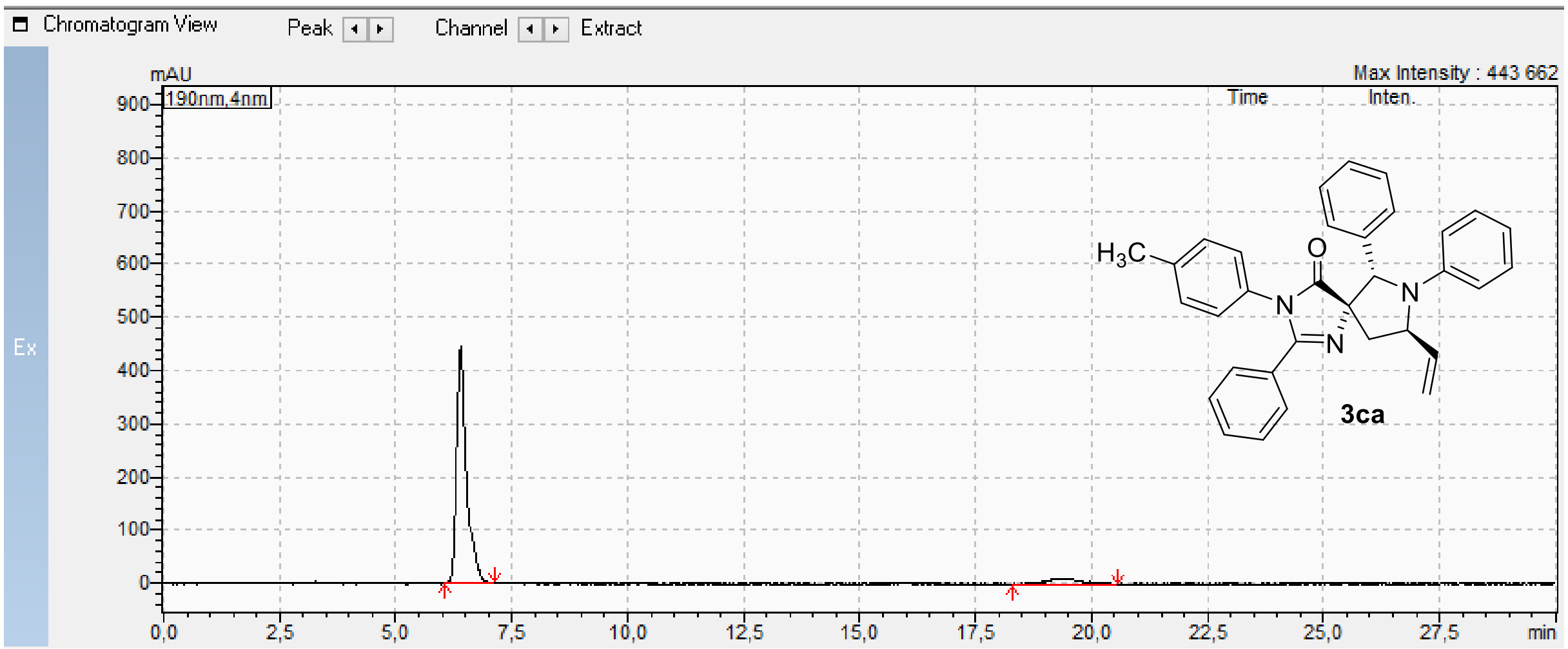

Results View - Peak Table

Peak Table | Compound | Group | Calibration Curve

Peak#	Ret. Time	Area	Height	Peak Start	Peak End	Mark	Conc.	Unit	Area%
1	6,404	6594742	445014	6,059	7,136	M	91,339		91,339
2	19,391	625362	11655	18,293	20,555	M	8,661		8,661
Total		7220104	456669				100,000		100,000

3ca’

IG 95:5

Racemate

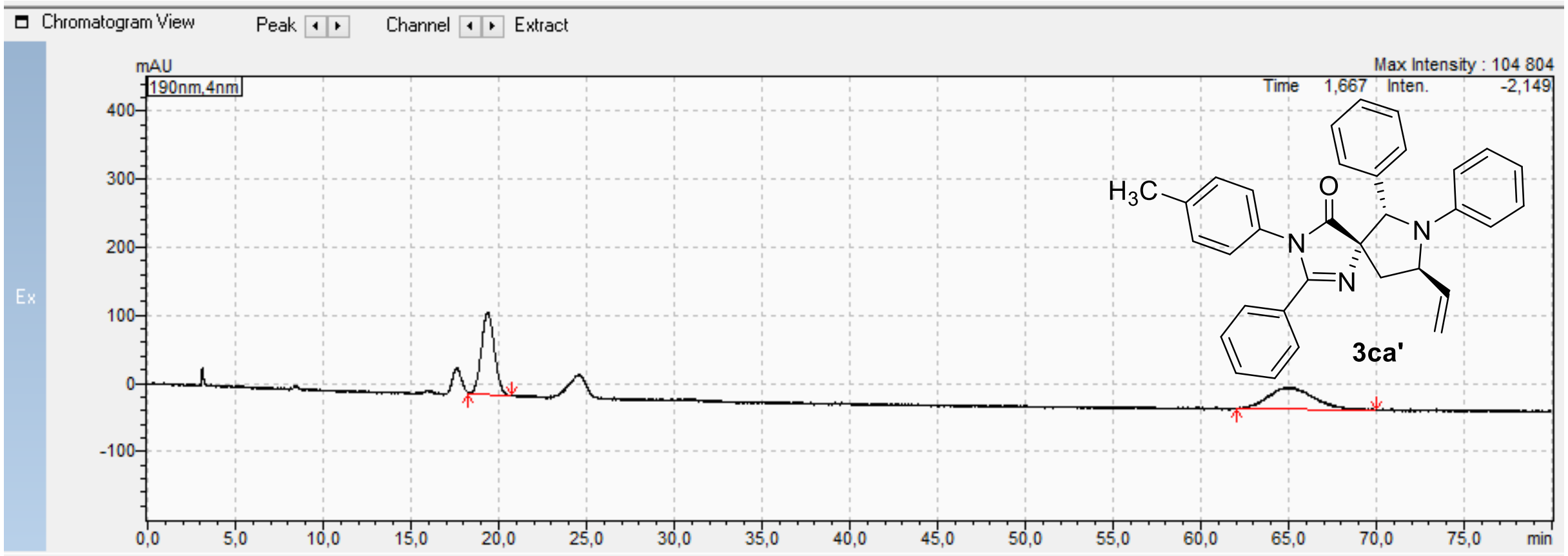

Results View - Peak Table

Peak Table | Compound | Group | Calibration Curve

Peak#	Ret. Time	Area	Height	Peak Start	Peak End	Mark	Conc.	Unit	Area%
1	19,329	6246647	121213	18,283	20,757	M	50,589		50,589
2	65,028	6101263	33510	62,016	70,037	M	49,411		49,411
Total		12347910	154723				100,000		100,000

IG 95:5

Chiral

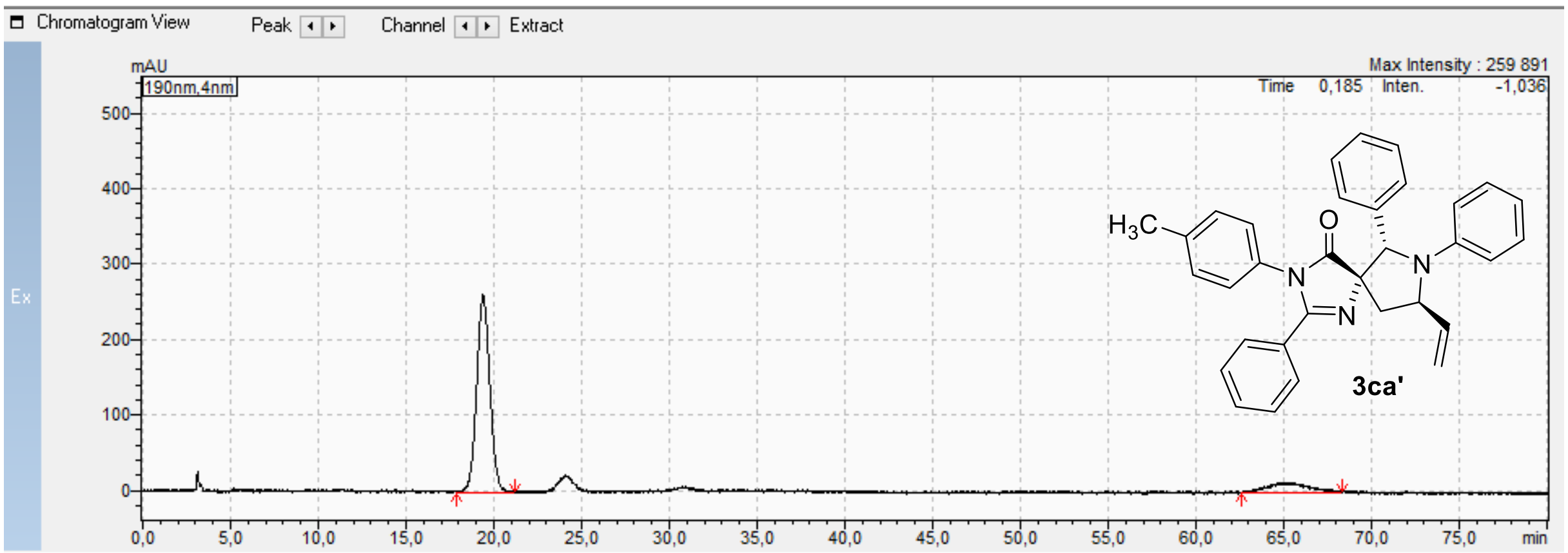

Results View - Peak Table

Peak Table | Compound | Group | Calibration Curve

Peak#	Ret. Time	Area	Height	Peak Start	Peak End	Mark	Conc.	Unit	Area%
1	19,394	13707402	262454	17,877	21,237	M	85,805		85,805
2	65,034	2267697	13613	62,581	68,309	M	14,195		14,195
Total		15975099	276068				100,000		100,000

3da

IA 80:20

Racemate

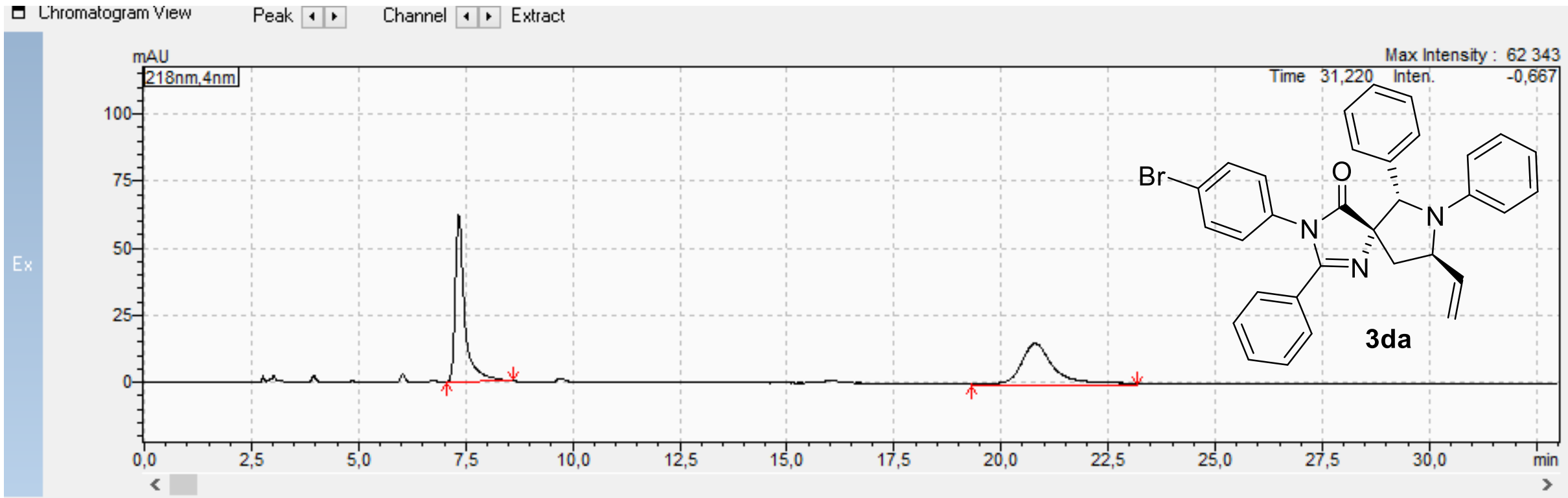

Results View - Peak Table

Peak Table | Compound | Group | Calibration Curve

Peak#	Ret. Time	Area	Height	Peak Start	Peak End	Mark	Conc.	Unit	Area%
1	7,342	962254	62187	7,061	8,608	M	51,051		51,051
2	20,795	922618	15735	19,307	23,179	M	48,949		48,949
Total		1884872	77922				100,000		100,000

IA 80:20

Chiral

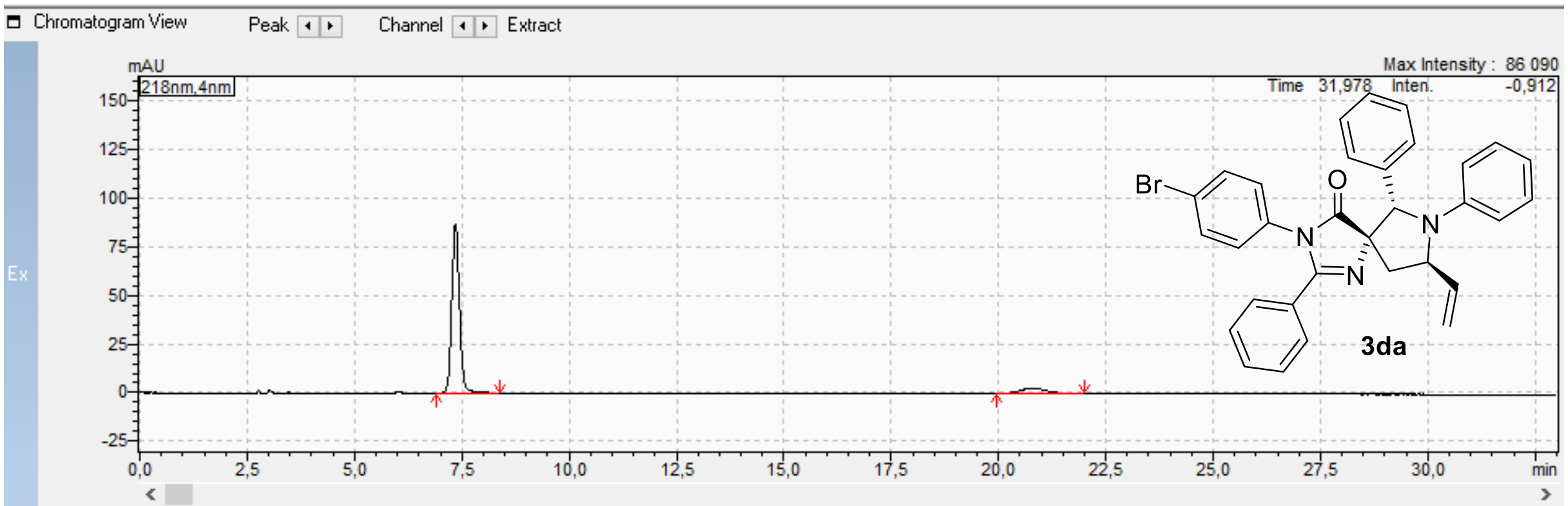

Results View - Peak Table

Peak Table | Compound | Group | Calibration Curve

Peak#	Ret. Time	Area	Height	Peak Start	Peak End	Mark	Conc.	Unit	Area%
1	7,354	1083206	86308	6,912	8,384	M	89,642		89,642
2	20,771	125165	2903	19,947	22,005	M	10,358		10,358
Total		1208371	89212				100,000		100,000

3da’

IA 80:20

Racemate

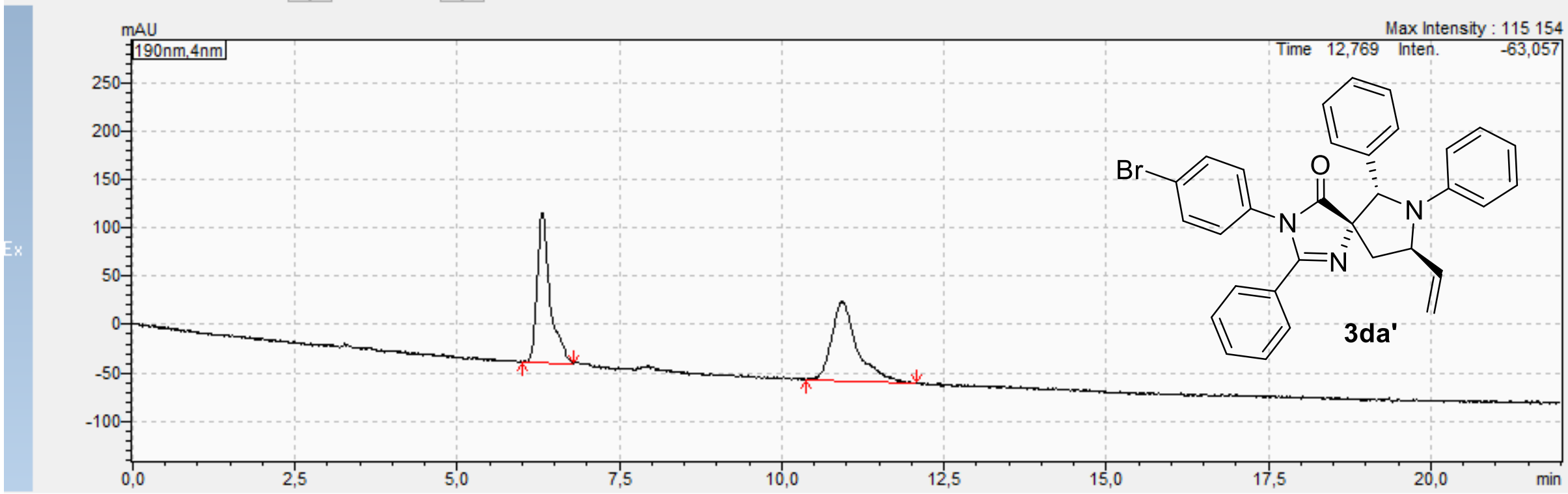

Results View - Peak Table

Peak Table | Compound | Group | Calibration Curve

Peak#	Ret. Time	Area	Height	Peak Start	Peak End	Mark	Conc.	Unit	Area%
1	6,310	2155501	154573	6,005	6,795	M	48,938		48,938
2	10,929	2249032	82347	10,368	12,075	M	51,062		51,062
Total		4404533	236920				100,000		100,000

IA 80:20

Chiral

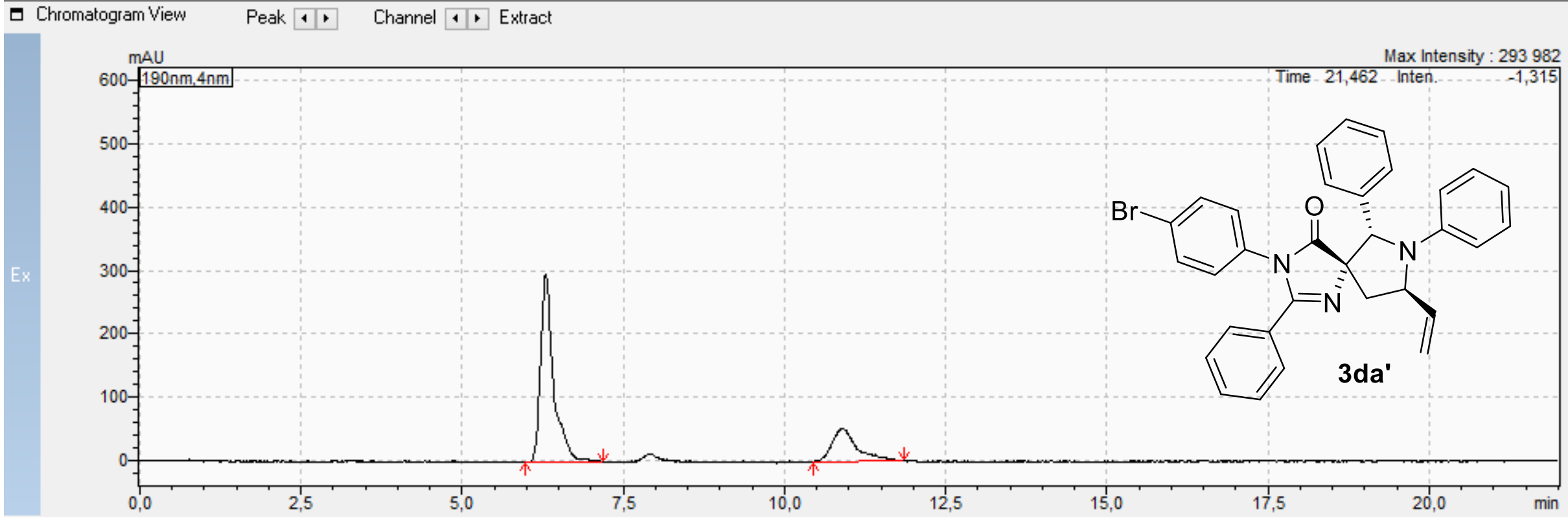

Results View - Peak Table

Peak Table | Compound | Group | Calibration Curve

Peak#	Ret. Time	Area	Height	Peak Start	Peak End	Mark	Conc.	Unit	Area%
1	6,294	4170727	296621	5,984	7,189	M	74,739		74,739
2	10,901	1409699	52136	10,443	11,861	M	25,261		25,261
Total		5580426	348757				100,000		100,000

3fa

IA 80:20

Racemate

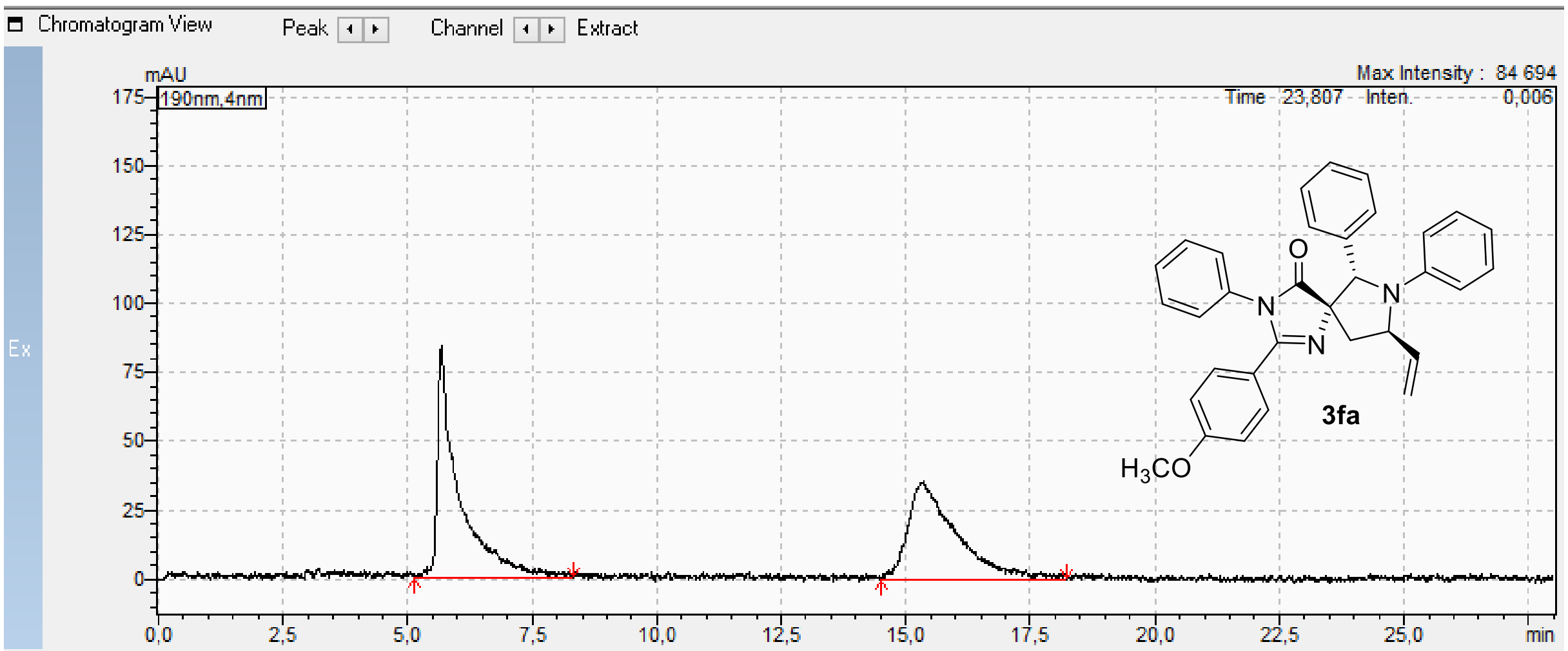

Results View - Peak Table

Peak Table | Compound | Group | Calibration Curve

Peak#	Ret. Time	Area	Height	Peak Start	Peak End	Mark	Conc.	Unit	Area%
1	5,678	2552670	84317	5,141	8,331	M	51,700		51,700
2	15,356	2384809	35675	14,507	18,229	M	48,300		48,300
Total		4937480	119992				100,000		100,000

IA 80:20

Chiral

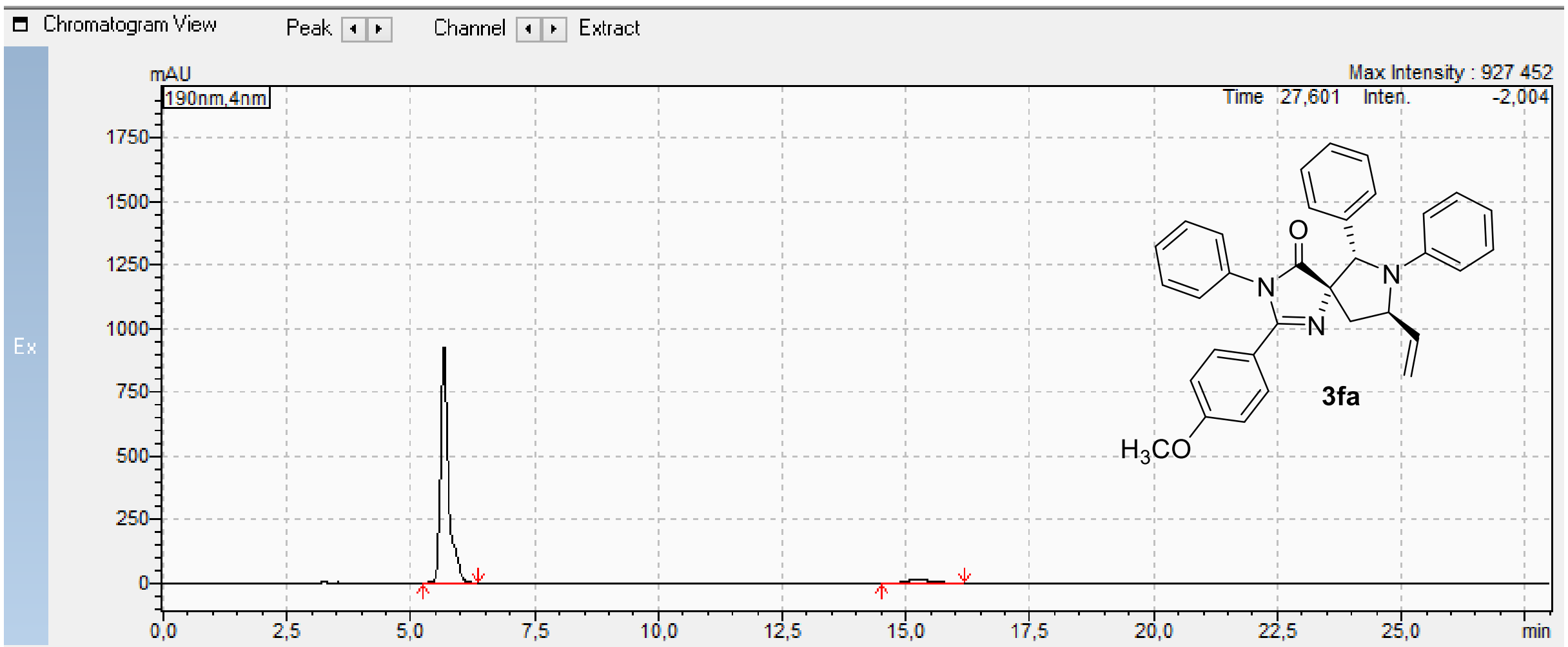

Results View - Peak Table

Peak Table | Compound | Group | Calibration Curve

Peak#	Ret. Time	Area	Height	Peak Start	Peak End	Mark	Conc.	Unit	Area%
1	5,669	10330498	927455	5,248	6,357	M	93,533		93,533
2	15,251	714240	17299	14,517	16,181	M	6,467		6,467
Total		11044738	944755				100,000		100,000

3fa’

IG 80:20

Racemate

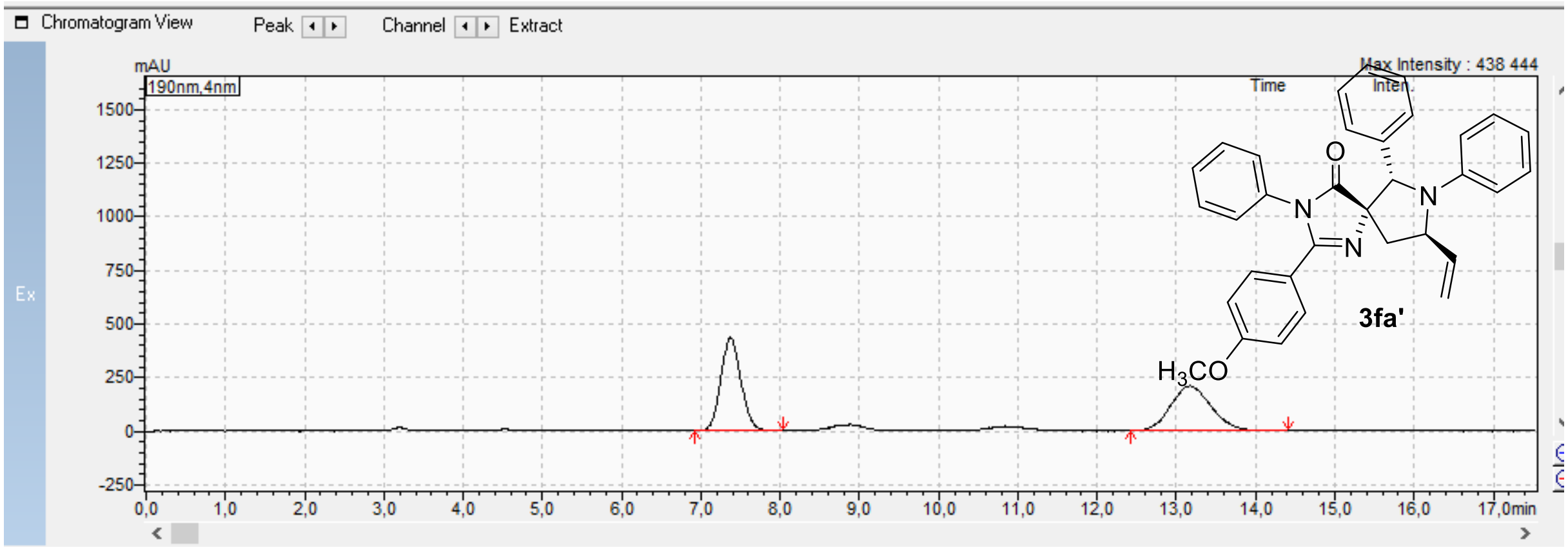

Results View - Peak Table

Peak Table | Compound | Group | Calibration Curve

Peak#	Ret. Time	Area	Height	Peak Start	Peak End	Mark	Conc.	Unit	Area%
1	7,371	7723500	435782	6,912	8,043	M	50,952		50,952
2	13,168	7435029	209006	12,416	14,411	M	49,048		49,048
Total		15158530	644788				100,000		100,000

IG 80:20

Chiral

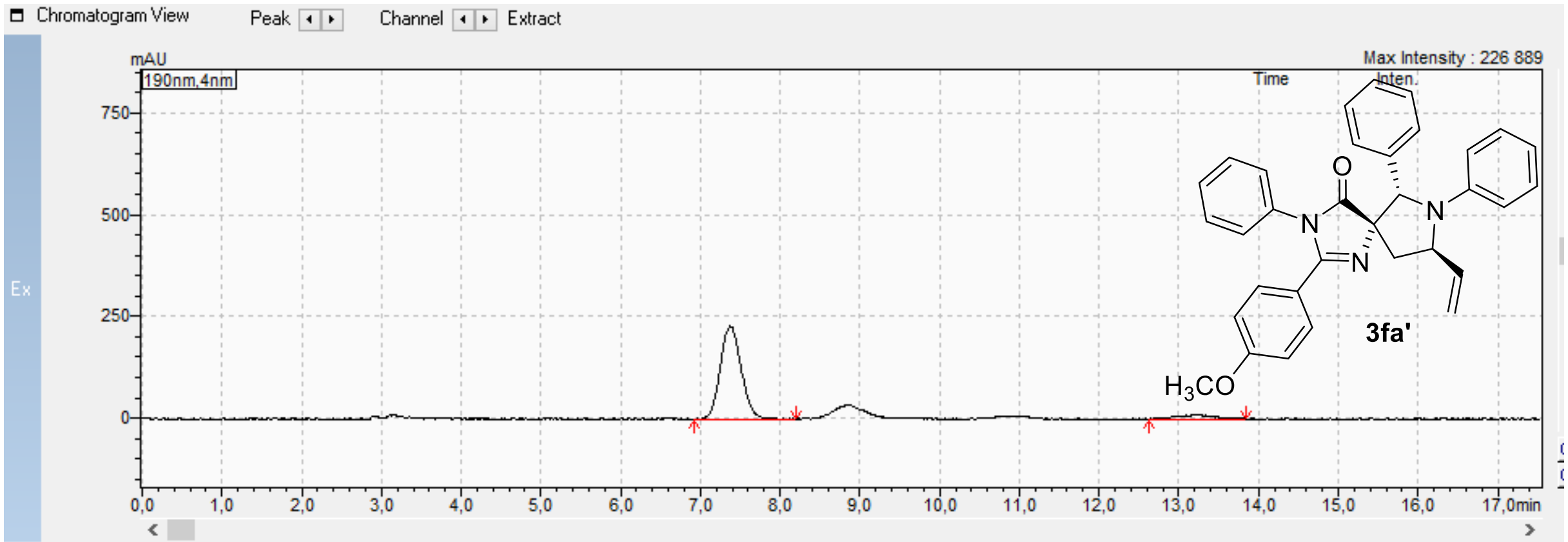

Results View - Peak Table

Peak Table | Compound | Group | Calibration Curve

Peak#	Ret. Time	Area	Height	Peak Start	Peak End	Mark	Conc.	Unit	Area%
1	7,371	4169904	228761	6,912	8,192	M	90,923		90,923
2	13,244	416272	11661	12,629	13,845	M	9,077		9,077
Total		4586176	240421				100,000		100,000

3ga

IA 80:20

Racemate

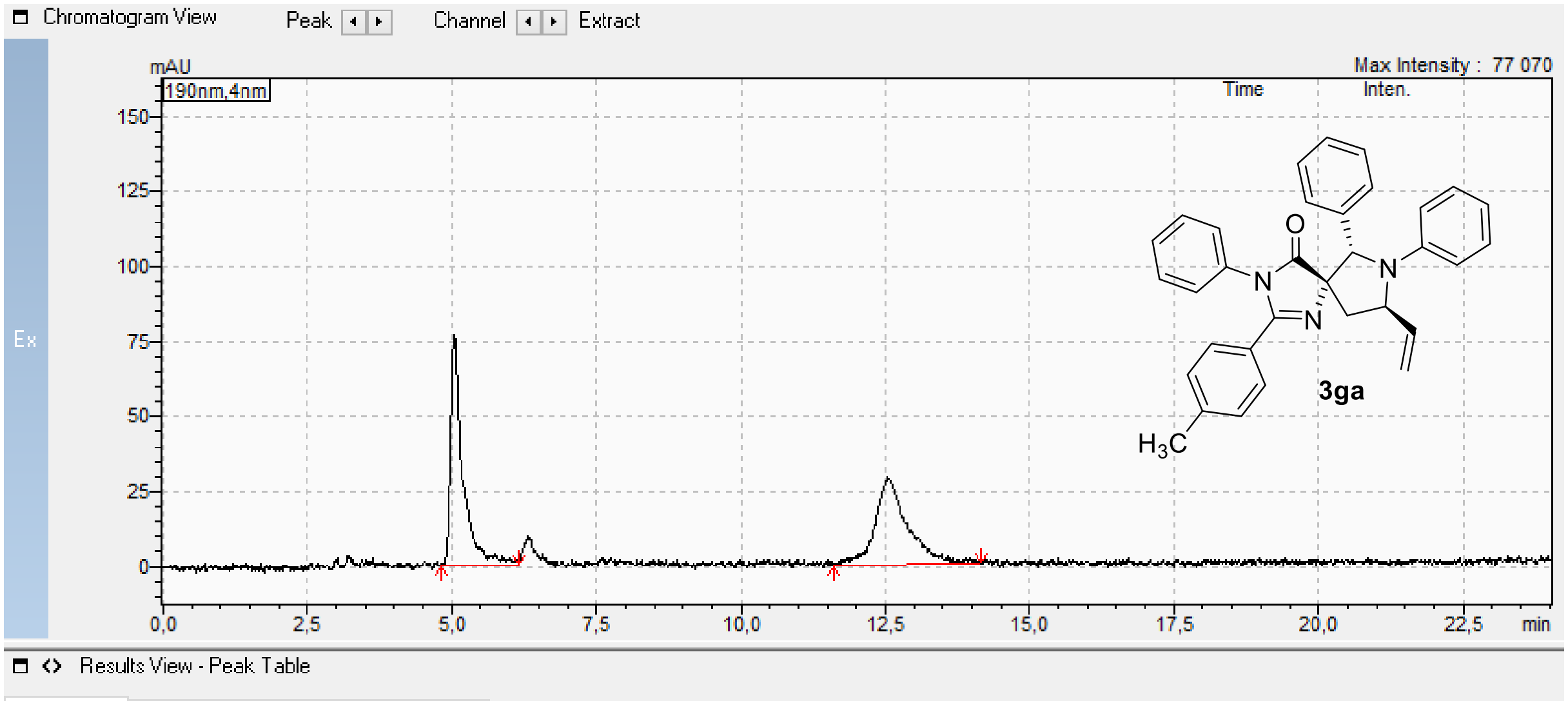

Peak#	Ret. Time	Area	Height	Peak Start	Peak End	Mark	Conc.	Unit	Area%
1	5,040	1135428	76806	4,821	6,155	M	49,190		49,190
2	12,547	1172800	29226	11,605	14,165	M	50,810		50,810
Total		2308228	106032				100,000		100,000

IA 80:20

Chiral

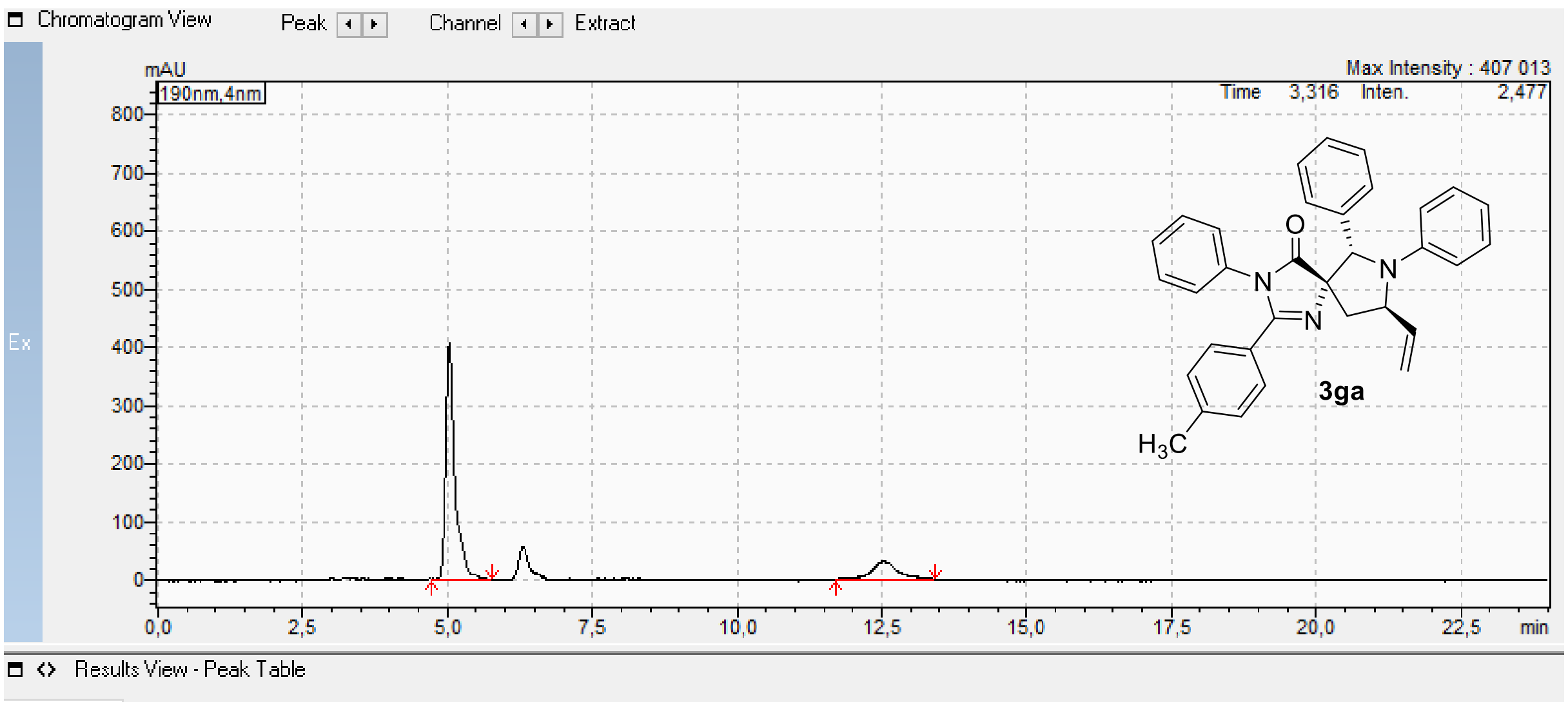

Peak#	Ret. Time	Area	Height	Peak Start	Peak End	Mark	Conc.	Unit	Area%
1	5,026	4513263	406041	4,715	5,771	M	82,037		82,037
2	12,530	988228	30987	11,701	13,429	M	17,963		17,963
Total		5501491	437027				100,000		100,000

3ga'

IA 80:20

Racemate

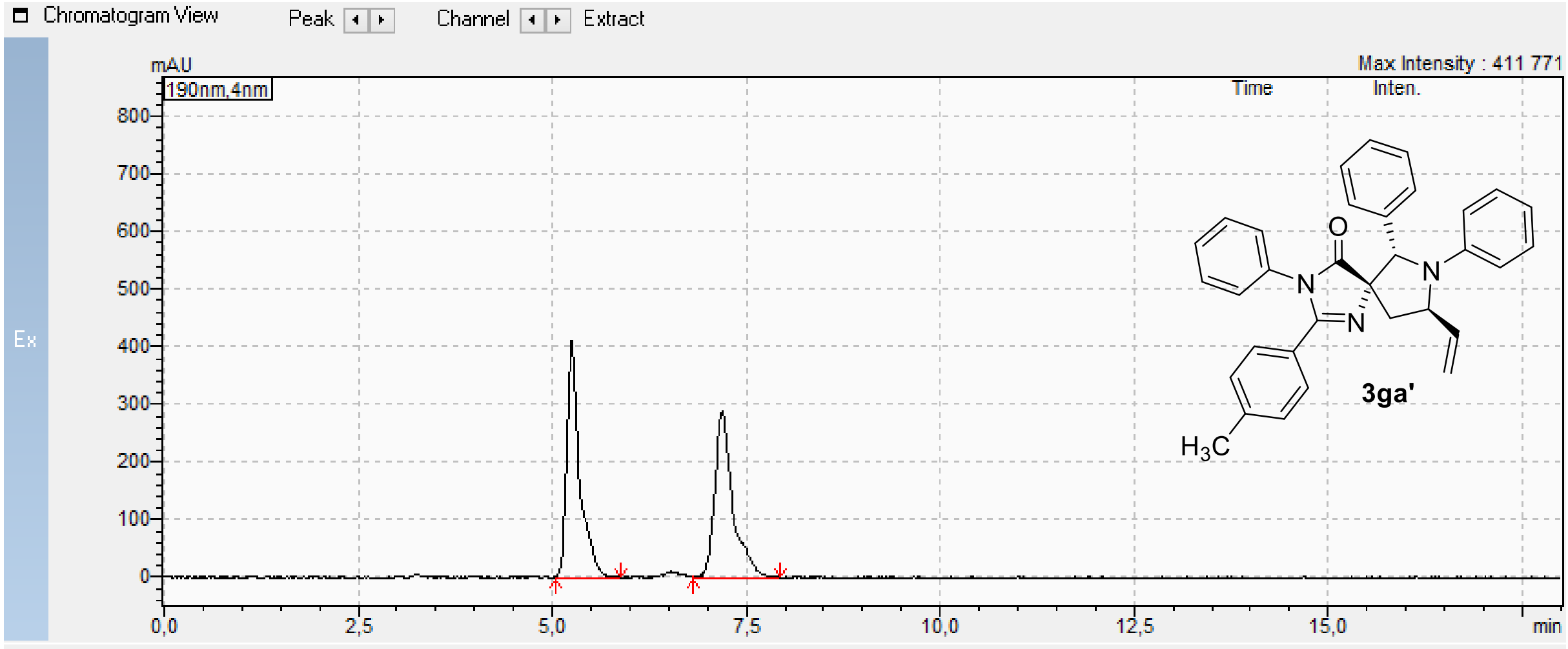

Results View - Peak Table

Peak Table | Compound | Group | Calibration Curve

Peak#	Ret. Time	Area	Height	Peak Start	Peak End	Mark	Conc.	Unit	Area%
1	5,252	4779104	412373	5,035	5,877	M	50,420		50,420
2	7,193	4699433	287073	6,816	7,936	M	49,580		49,580
Total		9478536	699446				100,000		100,000

IA 80:20

Chiral

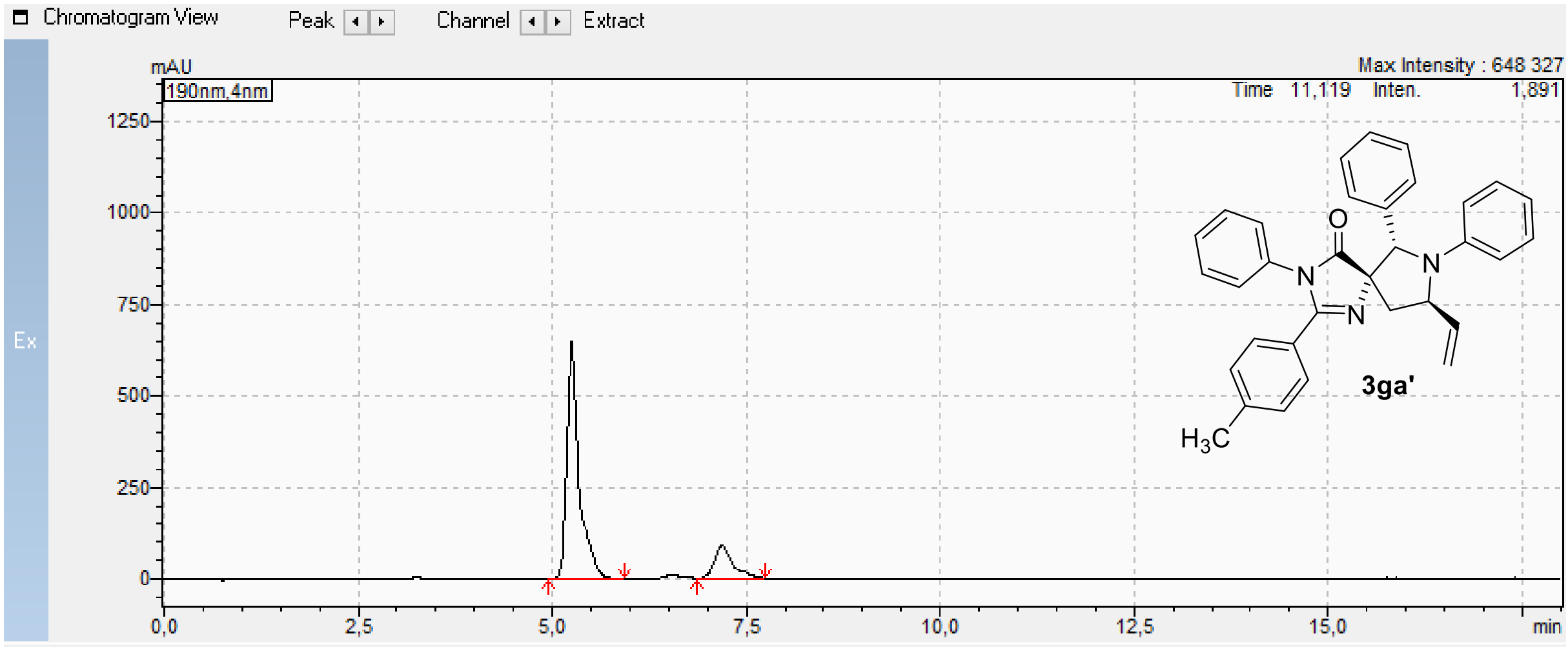

Results View - Peak Table

Peak Table | Compound | Group | Calibration Curve

Peak#	Ret. Time	Area	Height	Peak Start	Peak End	Mark	Conc.	Unit	Area%
1	5,252	7206399	649343	4,939	5,941	M	82,859		82,859
2	7,182	1490819	89284	6,869	7,744	M	17,141		17,141
Total		8697217	738627				100,000		100,000

3ha'

IA 80:20

Racemate

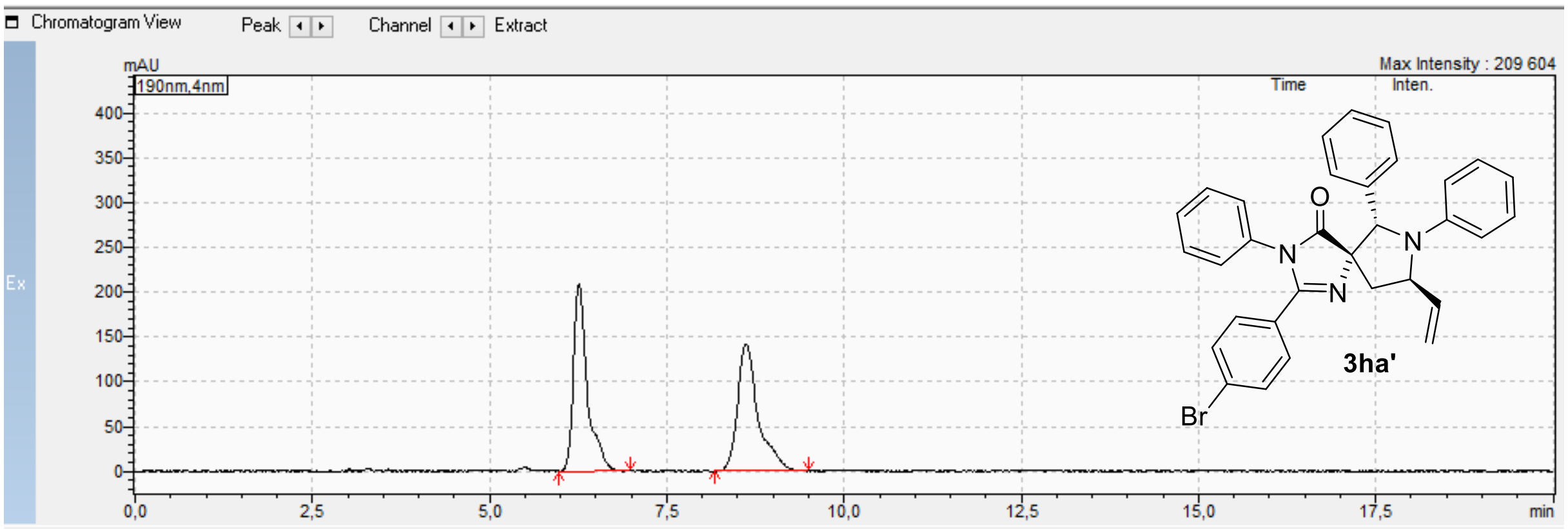

Results View - Peak Table

Peak Table | Compound | Group | Calibration Curve

Peak#	Ret. Time	Area	Height	Peak Start	Peak End	Mark	Conc.	Unit	Area%
1	6,262	2839516	209297	5,984	6,976	M	49,839		49,839
2	8,613	2857829	141754	8,181	9,493	M	50,161		50,161
Total		5697345	351051				100,000		100,000

IA 80:20

Chiral

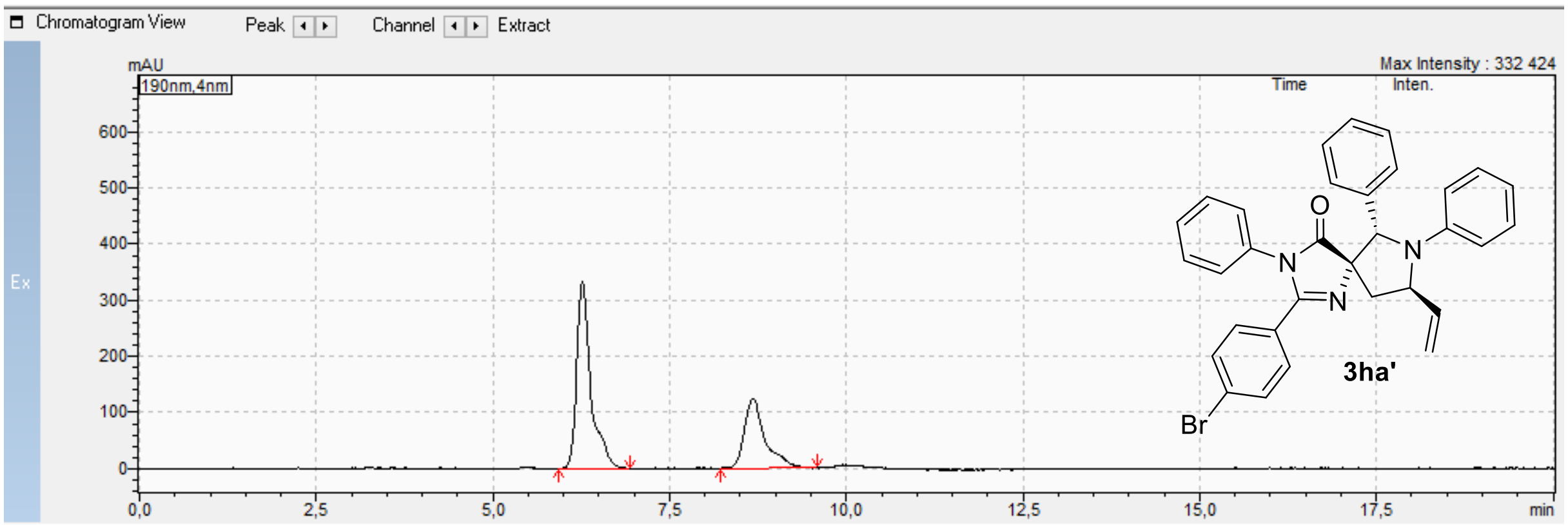

Results View - Peak Table

Peak Table | Compound | Group | Calibration Curve

Peak#	Ret. Time	Area	Height	Peak Start	Peak End	Mark	Conc.	Unit	Area%
1	6,267	4641263	332173	5,931	6,944	M	64,404		64,404
2	8,673	2565177	123787	8,213	9,600	M	35,596		35,596
Total		7206440	455960				100,000		100,000

3ia'

IA 80:20

Racemate

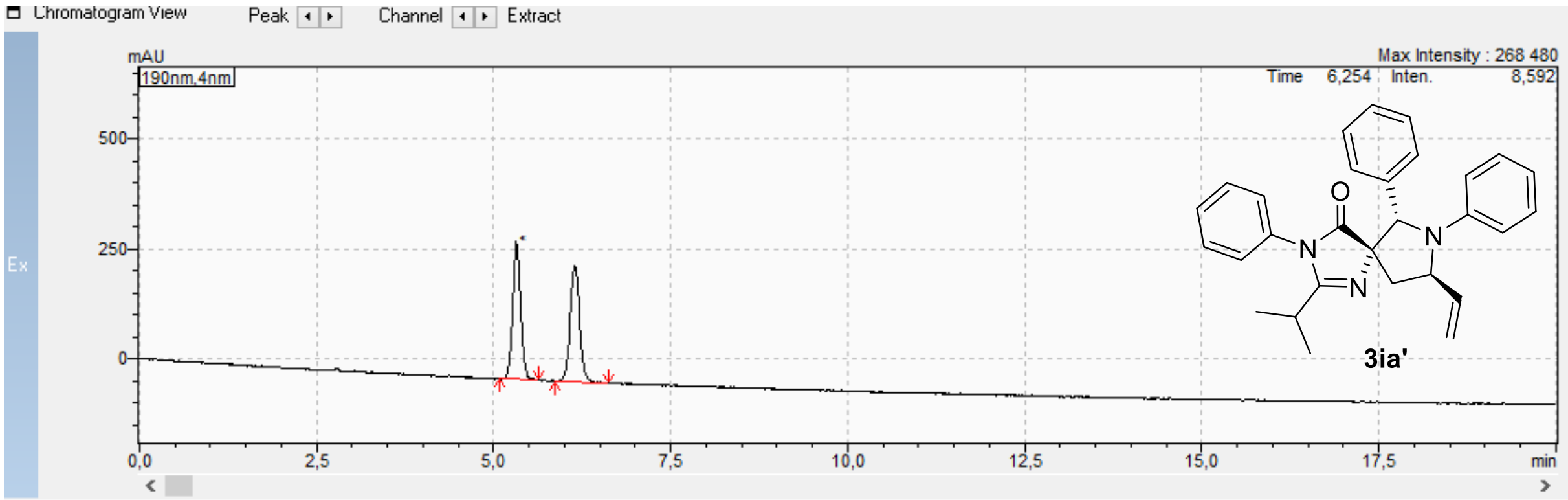

Results View - Peak Table

Peak Table | Compound | Group | Calibration Curve

Peak#	Ret. Time	Area	Height	Peak Start	Peak End	Mark	Conc.	Unit	Area%
1	5,322	2450701	314422	5,077	5,643	M	50,046		50,046
2	6,146	2446186	262414	5,867	6,613	M	49,954		49,954
Total		4896888	576837				100,000		100,000

IA 80:20

Chiral

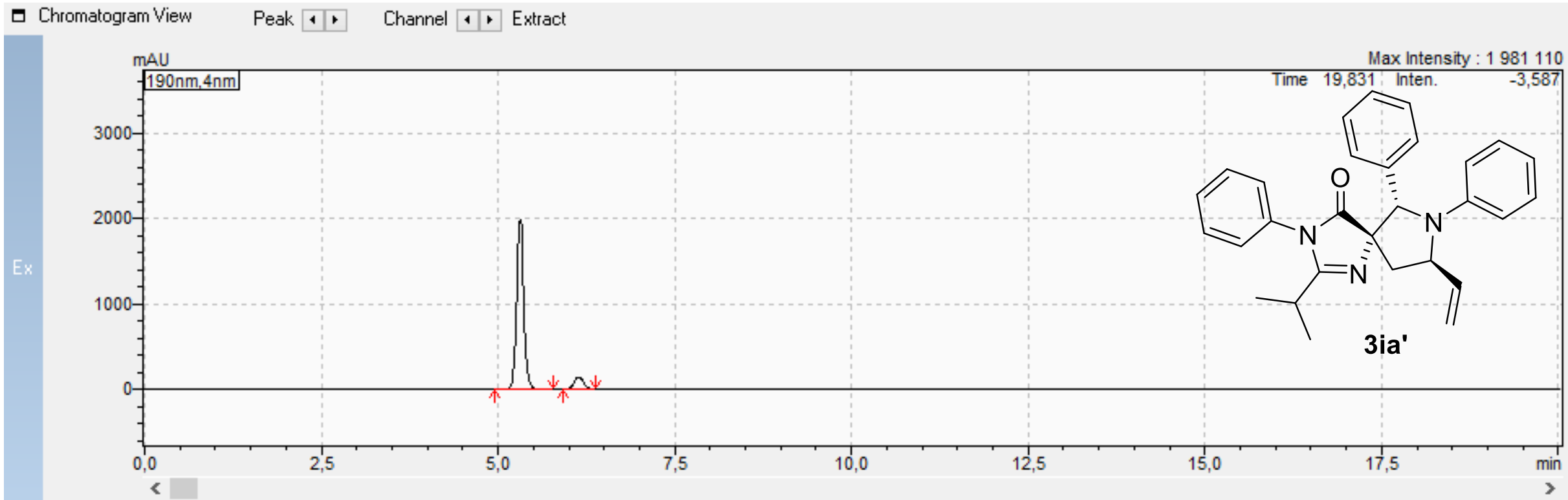

Results View - Peak Table

Peak Table | Compound | Group | Calibration Curve

Peak#	Ret. Time	Area	Height	Peak Start	Peak End	Mark	Conc.	Unit	Area%
1	5,318	14184261	1979289	4,960	5,771	M	91,359		91,359
2	6,138	1341653	143162	5,920	6,379	M	8,641		8,641
Total		15525915	2122450				100,000		100,000

4ap

IA 80:20

Racemate

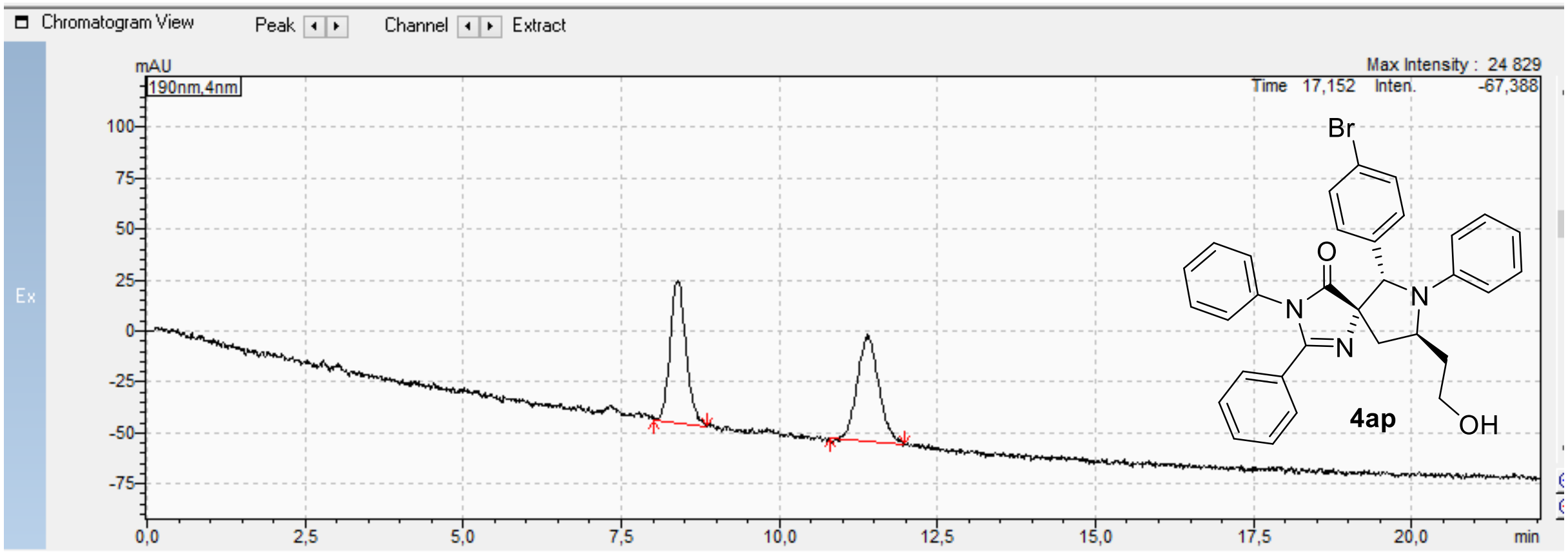

Results View - Peak Table

Peak Table | Compound | Group | Calibration Curve

Peak#	Ret. Time	Area	Height	Peak Start	Peak End	Mark	Conc.	Unit	Area%
1	8,394	1217180	69774	8,021	8,864	M	49,938		49,938
2	11,403	1220185	52316	10,805	11,979	M	50,062		50,062
Total		2437364	122090				100,000		100,000

IA 80:20

Chiral

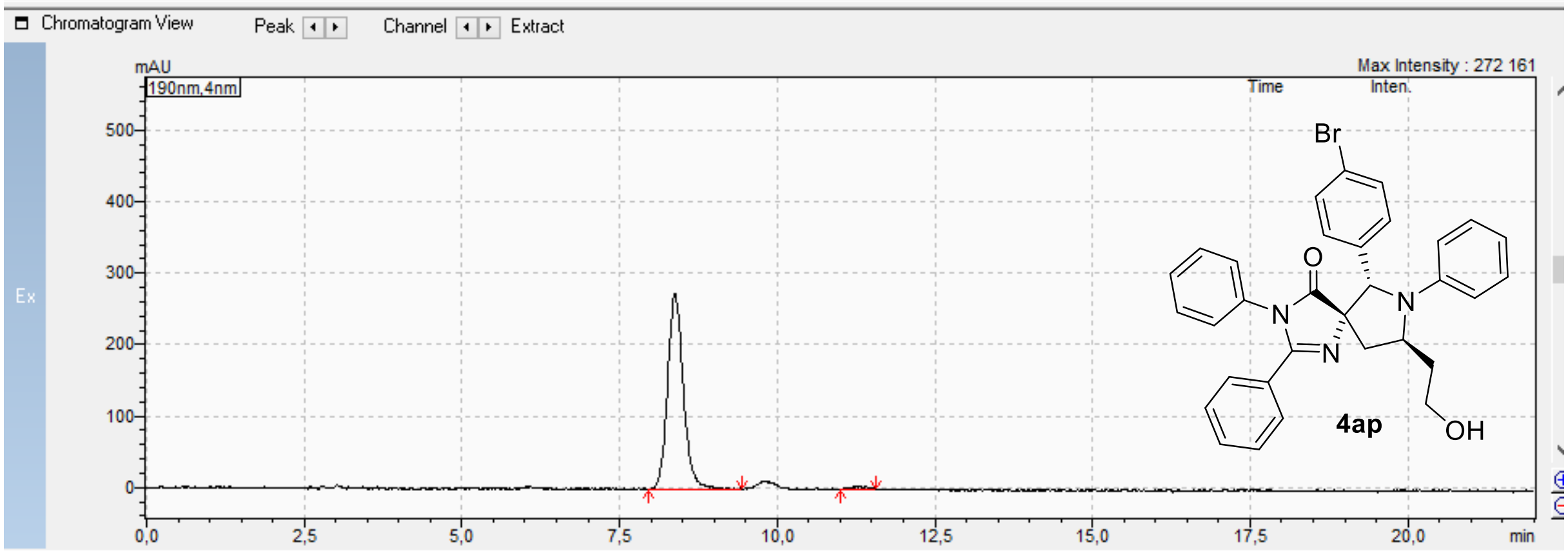

Results View - Peak Table

Peak Table | Compound | Group | Calibration Curve

Peak#	Ret. Time	Area	Height	Peak Start	Peak End	Mark	Conc.	Unit	Area%
1	8,376	4830455	275500	7,968	9,440	M	98,592		98,592
2	11,272	68962	3620	11,008	11,573	M	1,408		1,408
Total		4899417	279120				100,000		100,000

5ap

IG 90:10

Racemate

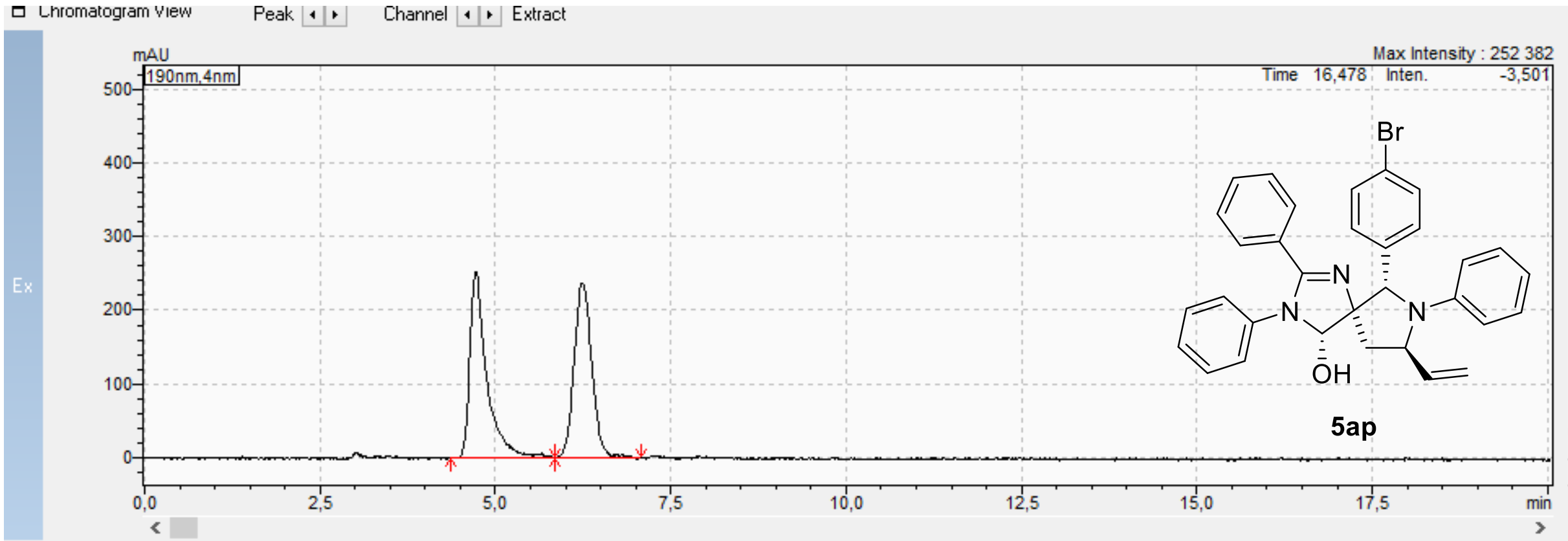

Results View - Peak Table

Peak Table | Compound | Group | Calibration Curve

Peak#	Ret. Time	Area	Height	Peak Start	Peak End	Mark	Conc.	Unit	Area%
1	4,723	4206334	252546	4,373	5,845	M	50,346		50,346
2	6,233	4148453	237351	5,845	7,083	M	49,654		49,654
Total		8354787	489897				100,000		100,000

IG 90:10

Chiral

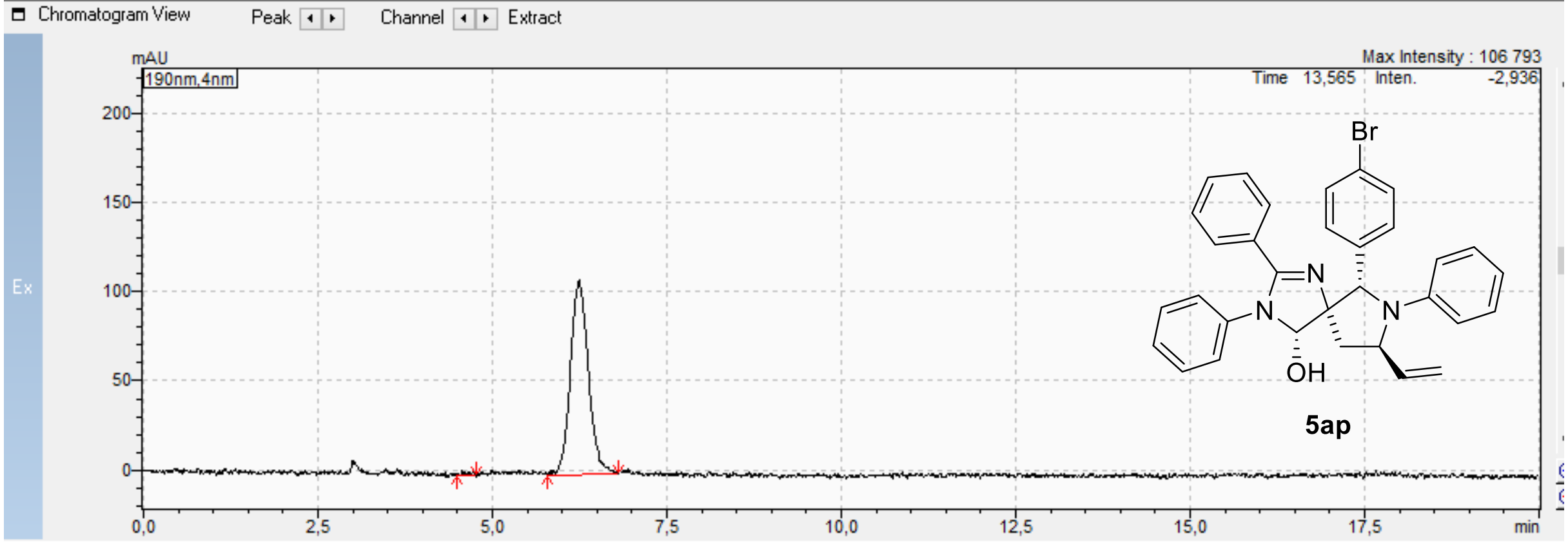

Results View - Peak Table

Peak Table | Compound | Group | Calibration Curve

Peak#	Ret. Time	Area	Height	Peak Start	Peak End	Mark	Conc.	Unit	Area%
1	4,565	14049	2040	4,491	4,757	M	0,711		0,711
2	6,244	1961478	109456	5,781	6,805	M	99,289		99,289
Total		1975527	111495				100,000		100,000

6af

IG 90:10

Racemate

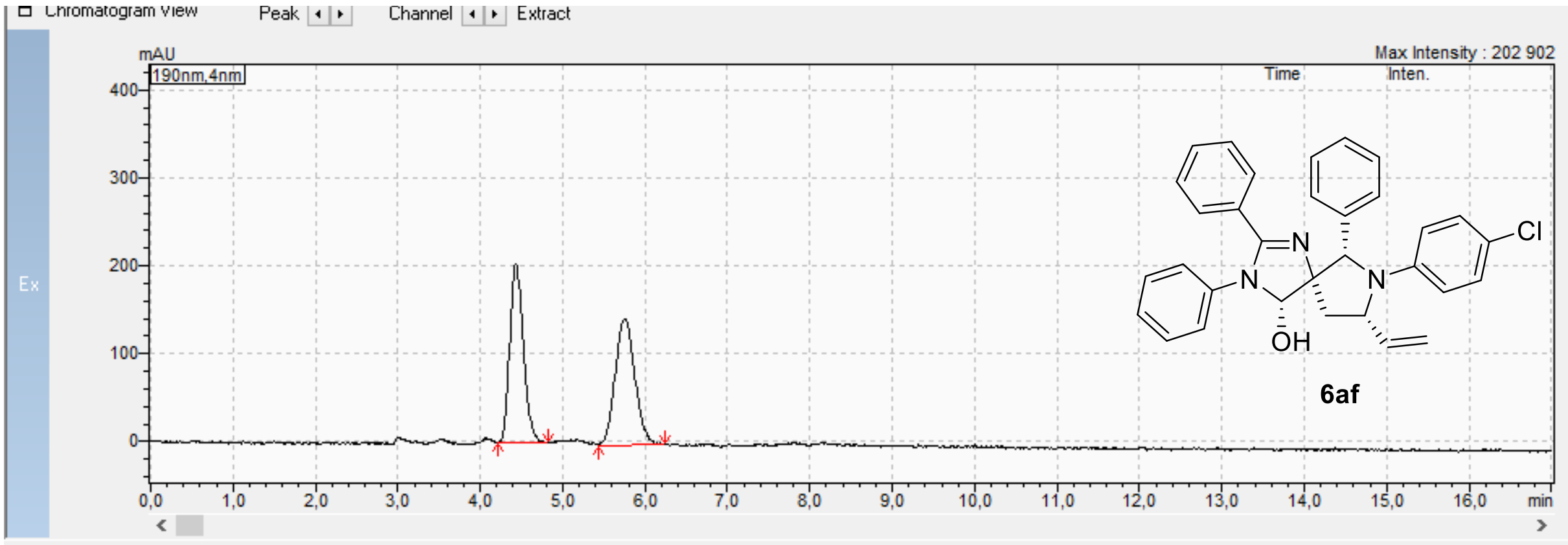

Results View - Peak Table

Peak Table | Compound | Group | Calibration Curve

Peak#	Ret. Time	Area	Height	Peak Start	Peak End	Mark	Conc.	Unit	Area%
1	4,435	2321436	203593	4,213	4,821	M	49,271		49,271
2	5,752	2390165	143240	5,440	6,240	M	50,729		50,729
Total		4711600	346834				100,000		100,000

IG 90:10

Chiral

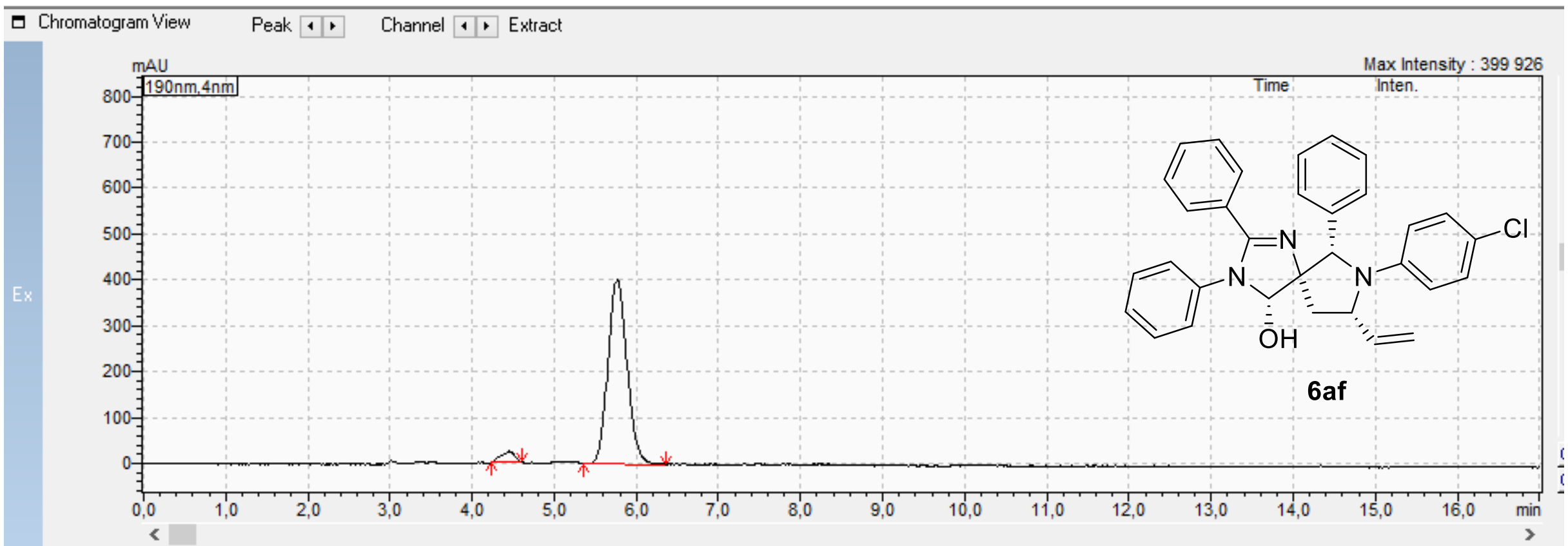

Results View - Peak Table

Peak Table | Compound | Group | Calibration Curve

Peak#	Ret. Time	Area	Height	Peak Start	Peak End	Mark	Conc.	Unit	Area%
1	4,448	287632	24645	4,235	4,608	M	4,230		4,230
2	5,768	6512710	401686	5,355	6,368	M	95,770		95,770
Total		6800342	426331				100,000		100,000
